# Supplementary material for: Photosensitizer-free visible-light-promoted glycosylation enabled by 2-glycosyloxy tropone donors
Source: Nat Commun. 2023 Dec 4;14:8025. doi: 10.1038/s41467-023-43786-y (PMC10695961; doi:10.1038/s41467-023-43786-y)
Supplement: Supplementary file 1 — Supplementary Information [file 41467_2023_43786_MOESM1_ESM.pdf]

Supplementary Information

Photosensitizer-Free Visible Light-Promoted Glycosylation Enabled by 2-Glycosyloxy Tropone Donors

Jing Zhang, Zhao-Xiang Luo, Xia Wu, Chen-Fei Gao, Peng-Yu Wang, Jin-Ze Chai, Miao Liu, Xin-Shan Ye, and De-Cai Xiong*

State Key Laboratory of Natural and Biomimetic Drugs, School of Pharmaceutical Sciences, Peking University, Beijing, China

Ningbo Institute of Marine Medicine, Peking University, Ningbo 315010, China

*** Correspondence:** decai@bjmu.edu.cn

Table of Contents

1. General information..3

2. Reaction optimization and control experiments 4

3. Preparation of 2-glycosyloxy tropone donors 9

4. Preparation of the acceptor **2r-x**...22

5. Glycosylation reactions using 2-glycosyloxy tropone donors..28

6. 2-Glycosyloxytropone as the acceptor 72

6.1 Synthesis of compound **S27**...72

6.2 Glycosylation reaction between 2-glycosyloxytropone with thioglycoside 73

6.3 Glycosylation reaction between 2-glycosyloxytropone with glycosyl imidates.....................74

7. Mechanism experiments...75

7.1 The effect of 1,1-diphenylethylene 75

7.2 Absorption and fluorescence spectra of compound **1a**...75

7.3 Decomposition experiments of the glycosyl donor in the presence of light...........................76

7.4 Light on and Light off experiments 77

7.5 Proposed Mechanism..82

8. References 82

8. NMR spectra 85

9. References 185

1. General information

All the reagents and solvents were purchased from commercial suppliers and used directly without further purification unless otherwise stated. Dry $ClCH_2CH_2Cl$ was bought from J&K Scientific in China. Visualization on thin-layer chromatography (TLC) was achieved by use of UV light (254 nm), a solution of concentrated sulfuric acid (5.0 mL) in CH_3CH_2OH (95.0 mL) or a solution of $(NH_4)_6Mo_7O_{24}$•$4H_2O$ (12.00 g, 9.7 mmol) and $Ce(NH_4)_2(NO_3)_6$ (0.25 g, 0.45 mmol) in sulfuric acid (5%, 250 mL). Column chromatography was conducted on silica gel (200-300 mesh). NMR spectras were recorded using $CDCl_3$ or CD_3OD as the solvent on a Bruker AV 400 or Bruker AV 600 at 400 or 600 MHz for ^1H NMR, 101 or 151 MHz for ^{13}C NMR, 162 MHz for ^{31}P NMR, 376 MHz for ^{19}F NMR, respectively. Chemical shifts were reported in ppm and coupling constants of ^1H NMR were expressed in Hz. The multiplicity of data for ^1H NMR was recorded as follows: s = singlet, d = doublet, t = triplet, q = quartet, m = multiplet, dd = doublet of doublets, and br = broad. High-resolution mass spectra data was obtained using a Waters Xevo G2 Q-TOF mass spectrometer.

Standard setup (3 × 5 w)

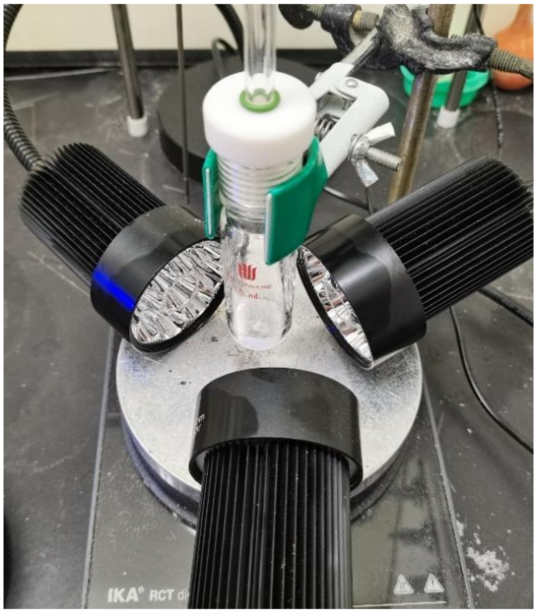

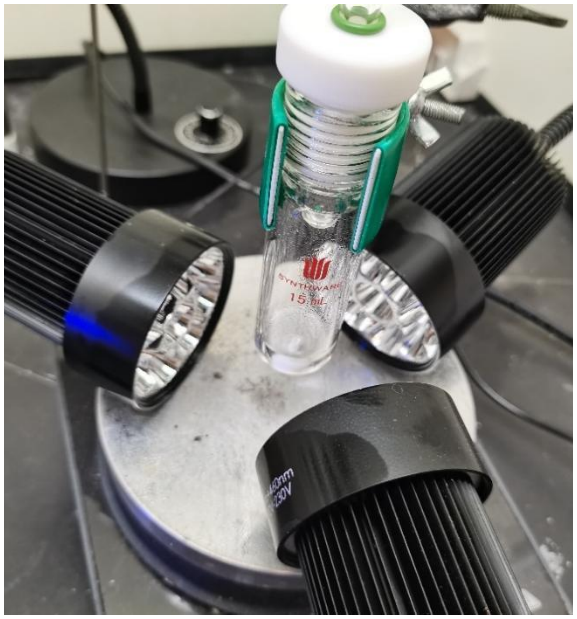

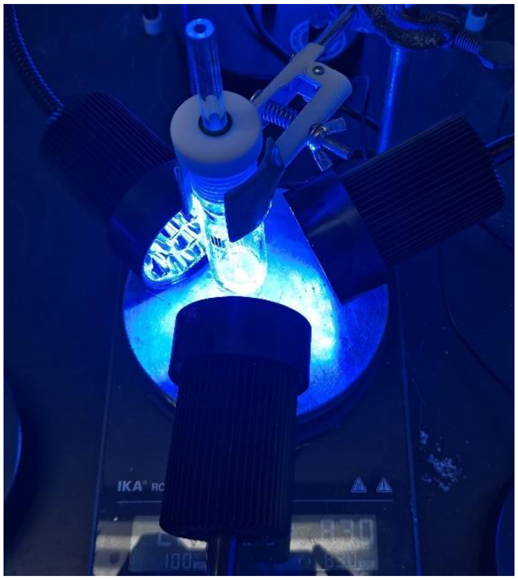

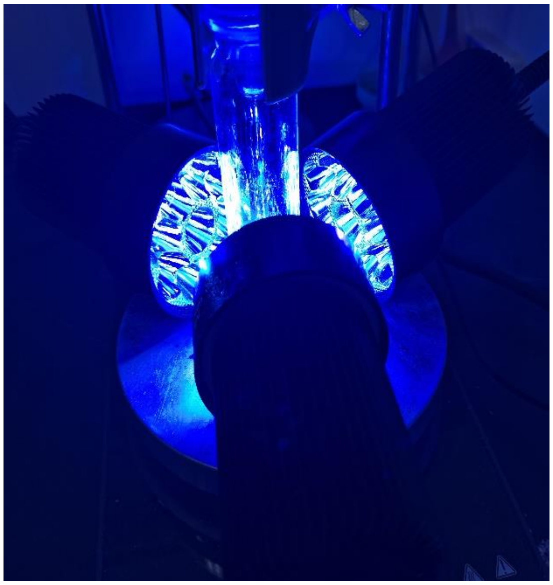

2. Reaction optimization and control experiments

Supplementary Table 1. Effect of various irradiation wavelengths.[a]

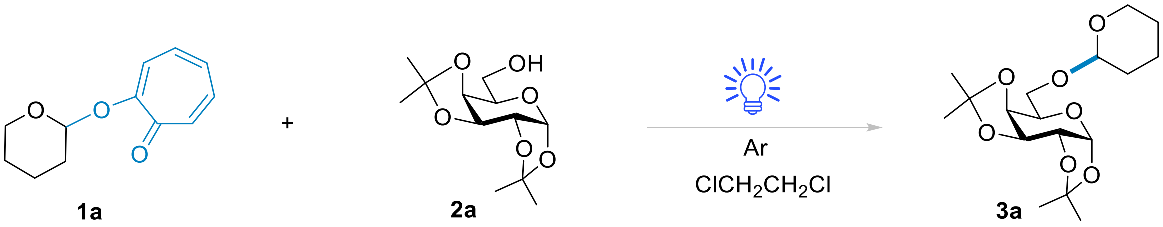

Entry	Wavelengths	Time (h)	Yield (%)[b]
1	none	3	0
2	310-320 nm	3	22
3	365-370 nm	3	20
4	385-390 nm	3	65
5	415-420 nm	3	66
6	430-435 nm	3	65
**8**	**blue LEDs**	**3**	**67**

[a] Reaction conditions: **1a** (0.075 mmol), **2a** (0.05 mmol), irradiation wavelengths, $ClCH_2CH_2Cl$ (2.0 mL), argon atmosphere. [b] Isolated yields.

Supplementary Table 2. Effect of illumination intensity.[a]

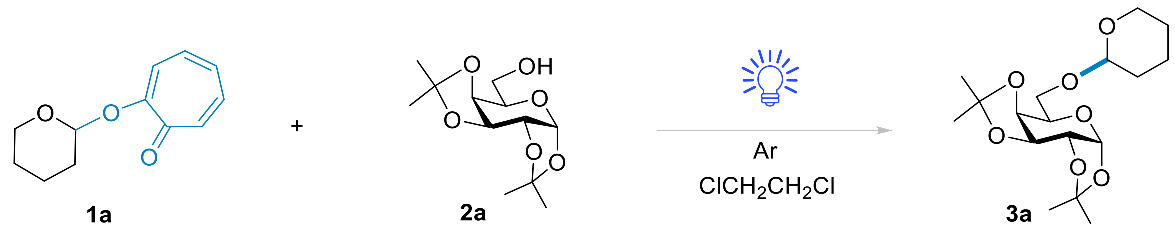

Entry	Illumination intensity	Time (h)	Yield (%)[b]
**1**	**15 W**	**3**	**67**
2	10 W	3	59
3	5 W	3	20
4	3 W	3	10

[a] Reaction conditions: **1a** (0.075 mmol), **2a** (0.05 mmol), blue LEDs, $ClCH_2CH_2Cl$ (2.0 mL), argon atmosphere. [b] Isolated yields.

Supplementary Table 3. Effect of different solvents.[a]

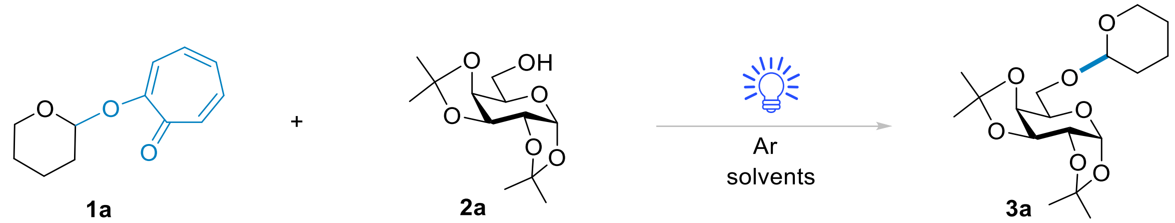

Entry	solvents	Time (h)	Yield (%)[b]
1	DMF	3	40
2	CH_2Cl_2	3	65
3	CH_3CN	3	44
4	Toluene	3	30
5	THF	3	20

6	DME	3	NR
**8**	**$ClCH_2CH_2Cl$**	**3**	**67**

[a] Reaction conditions: **1a** (0.075 mmol), **2a** (0.05 mmol), blue LEDs (15 W), solvents (2.0 mL), argon atmosphere. [b] Isolated yields.

Supplementary Table 4. Effect of additives and molecular sieves on this reaction.[a]

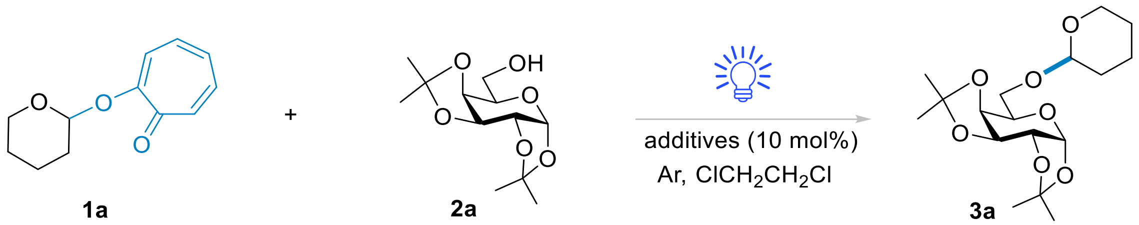

1a **2a** **3a**

Entry	Additive	Time	Yield (%)[b]
1	-	3 h	67
2	NaB(3,5- $(CF_3)_2Ph)_4$	2 h	66
3	$NaSO_4Me$	1 h	66
4	NaOTf	1 h	77
5	Bu_4NOTf	3 h	71
6	$Cu(OTf)_2$	10 min	75
7	TMSOTf	10 min	83
8[c]	-	5 h	82
**9[c]**	**NaOTF**	**20 min**	**91**
10	4 Å MS (200 mg)	3 h	NR

[a] Reaction conditions: **1a** (0.075 mmol), **2a** (0.05 mmol), additive (10 mol%), blue LEDs (15 W), $ClCH_2CH_2Cl$ (2.0 mL), argon atmosphere. [b] Isolated yields. [c] **1a** (0.10 mmol).

Supplementary Table 5. Optimization of the glycosylation reaction between donor 1b with acceptor 2b.[a]

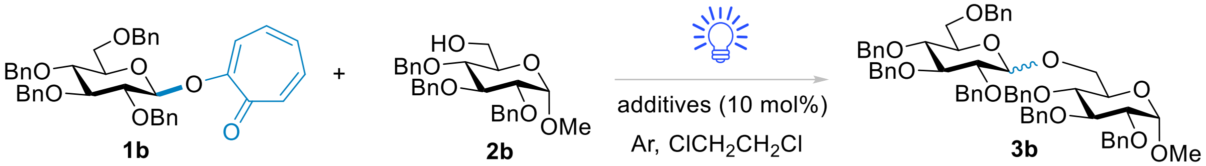

Entry	Additive	Time (h)	Yield (%)[b]	α/β ratio[b]
1	no additive	12	13	1.7/1
2	NaOTf	2.5	71	1.8/1
3	NaB(3,5- $(CF_3)_2Ph)_4$	6	63	1.7/1
4	$NaSO_2CF_3$	2.5	60	1.7/1
5	$NaBF_4$	12	30	2.2/1
6	NaOAc	12	10	1.6/1
7	Sodium *p*-toluenesulfinate	12	23	1.4/1
8	NaI	12	18	1.7/1
9	$NaClO_4$	12	10	1.7/1
10	$NaNTf_2$	8	41	1.7/1
11	NaOTs	12	10	1.7/1
12	$NaSO_4Me$	2	50	3.0/1
13	$NaSO_3Me$	10	10	1.7/1
14	Na_2SO4	12	10	1.7/1
15	Bu_4NOTf	8	42	2.0/1
16	KOTf	8	54	1.9/1
17	TfOH	0.5	84	1.9/1
18	$BF_3{\cdot}Et_2O$	0.5	17	3.4/1

19	$HOPO(OPh)_2$	12	10	1.7/1
20	H_3PO_4	1	17	1.1/1
21	TMSOTf	0.5	92	2.0/1
22	no Blue LEDs, no additive	24	NR	-
23	NaOTf, in dark	12	NR	-
24	TMSOTf, in dark	0.5	44	2.0/1
25	TMSOTf, in dark	12	44	2.0/1
26	TMSOTf, 4Å MS (200 mg)	12	13	2.0/1
27	TMSOTf, 4Å MS (200 mg), in dark	12	NR	-

[a] Reaction conditions: **1b** (0.075 mmol), **2b** (0.05 mmol), additive (10 mol%), blue LEDs (15 W), $ClCH_2CH_2Cl$ (2.0 mL), argon atmosphere. [b] Isolated yields, ratios of α/β determined by ^1H NMR.

3. Preparation of 2-glycosyloxy tropone donors

Donor list:

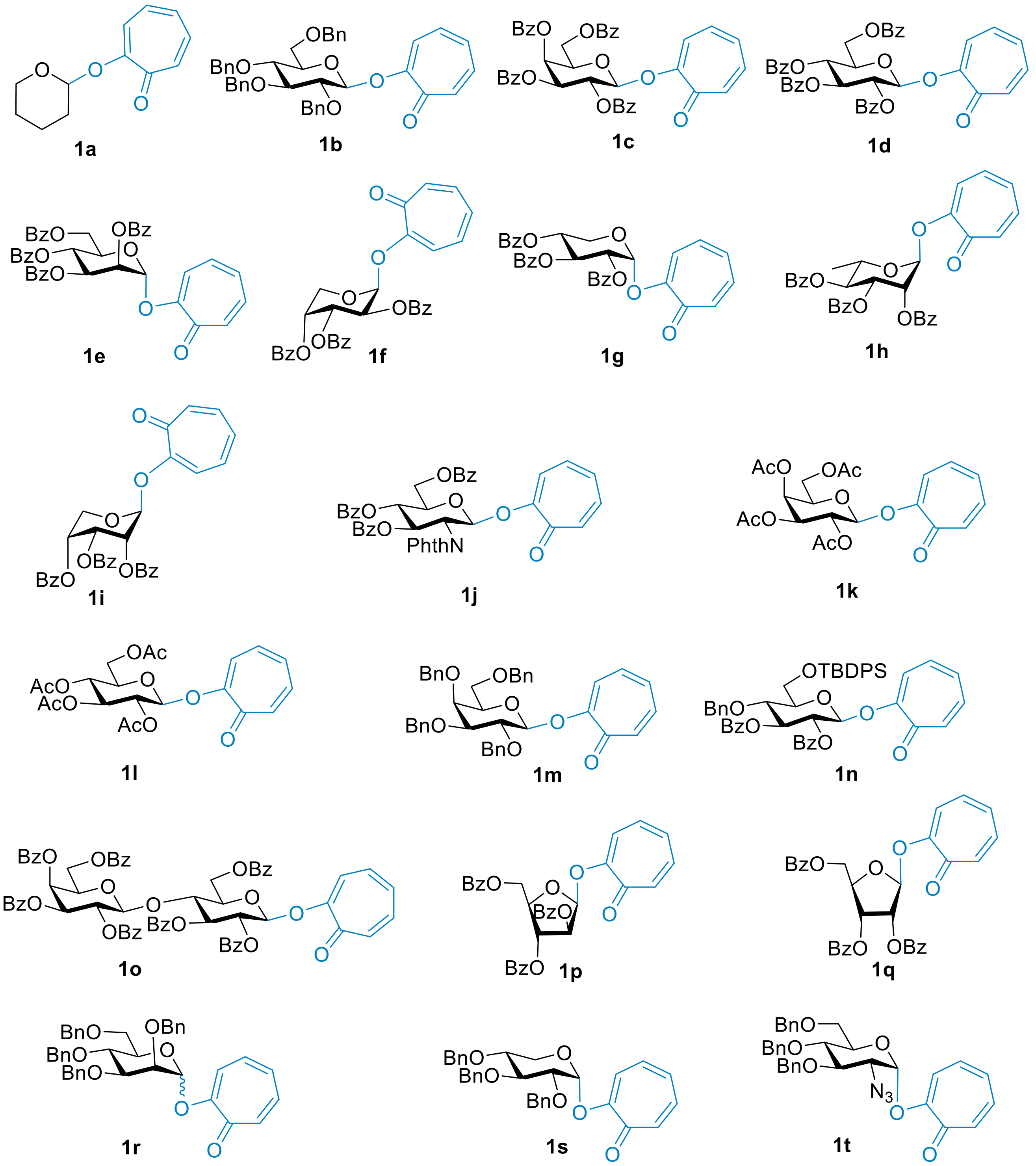

Compounds **1a-t** were synthesized as follows.

7-Oxocyclohepta-1,3,5-trien-1-yl tetrahydro-2*H*-pyran (1a)

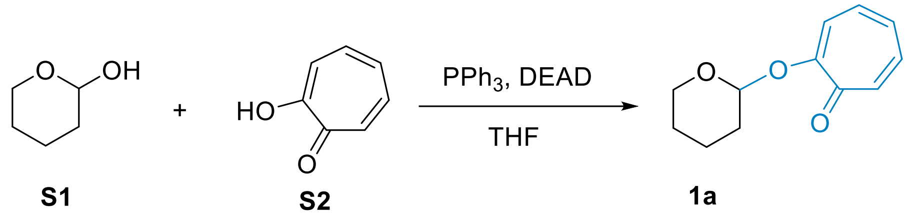

To a solution of **S1** (2.04 g, 20.0 mmol), **S2** (1.22 g, 10.0 mmol) and PPh_3 (5.02 g, 20.0 mmol) in dry THF (30.0 mL), diethylazodicarboxylate (DEAD, 3.20 mL, 20.0 mmol) was added slowly at 0°C under argon atmosphere. Then the mixture was stirred at room temperature and the progress of the reaction was monitored by TLC. Upon completion, the reaction mixture was concentrated in vacuo. The residue was purified by flash column chromatography on silica gel (petroleum ether/EtOAc = 2/1) to obtained **1a** (885.0 mg, 43%) as a yellow oil. **^{1}H NMR** (400 MHz, $CDCl_3$) δ 7.23 - 7.19 (m, 3H), 7.04 (t, *J* = 10.4 Hz, 1H), 6.91 - 6.86 (m, 1H), 5.61 (t, *J* = 2.9 Hz, 1H), 3.87 (td, *J* = 10.9, 3.0 Hz, 1H), 3.68 - 3.60 (m, 1H), 2.24 - 1.97 (m, 2H), 1.96 - 1.83 (m, 1H), 1.81 - 1.60 (m, 3H). **^{13}C NMR** (101 MHz, $CDCl_3$) δ 181.0, 163.1, 138.2, 136.0, 132.7, 129.0, 118.3, 96.7, 62.1, 30.0, 25.0, 18.2. **HRMS** (ESI) Calculated for $C_{12}H_{14}NaO_3$ $[M+Na]^+$: 229.0841 Found: 229.0839.

7-Oxocyclohepta-1,3,5-trien-1-yl 2,3,4,6-tetra-*O*-benzyl-β-D-glucopyranoside (1b)

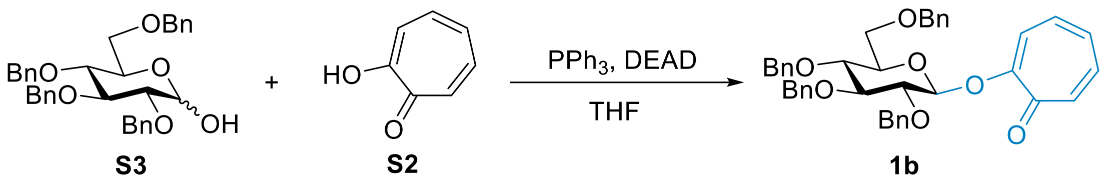

To a solution of **S3** (3.24 g, 6.00 mmol), **S2** (1.09 g, 9.00 mmol) and PPh_3 (3.12 g, 12.0 mmol) in dry THF (30.0 mL), diethylazodicarboxylate (DEAD, 1.89 mL, 12.0 mmol) was added slowly at 0°C under argon atmosphere. Then the mixture was stirred at room temperature and the progress of the reaction was monitored by TLC. Upon completion, the reaction mixture was concentrated in vacuo. The residue was purified by flash column chromatography on silica gel (petroleum ether/EtOAc = 4/1) to obtain product **1b** (2.89 g, 75%) as a white solid. $[\alpha]_D^{25}$: -92.67 (*c*: 0.15 $CHCl_3$). **^{1}H NMR** (400 MHz, $CDCl_3$) δ 7.40 - 7.37 (m, 2H), 7.34 - 7.27 (m, 14H), 7.25 - 7.16 (m, 6H), 7.12 - 7.10 (m, 1H), 6.89 - 6.86 (m, 2H), 5.30 (d, *J* = 10.7 Hz, 1H), 5.06 (d, *J* = 7.6 Hz, 1H), 5.01 (d, *J* = 11.0 Hz, 1H), 4.89 - 4.80 (m, 3H), 4.58 - 4.49 (m, 3H), 3.88 (dd, *J* = 9.1, 7.6 Hz, 1H), 3.80 - 3.71 (m, 2H), 3.67 - 3.61 (m, 3H). **^{13}C NMR** (101 MHz, $CDCl_3$) δ 180.1, 163.3, 138.6, 138.4, 138.2, 138.0, 137.9, 136.0, 132.4, 129.6, 128.6, 128.42, 128.40, 128.35, 128.31, 127.98, 127.86, 127.85, 127.67, 127.65, 127.61, 117.6, 101.3, 84.3, 81.2, 77.4, 75.7, 75.5, 75.1, 74.8, 73.4, 68.9. **HRMS** (ESI) Calculated for $C_{41}H_{40}O_7Na$ $[M+Na]^+$: 667.2672 Found: 667.2668.

General Procedure A:

To a solution of carbohydrate in pyridine, benzoyl chloride (BzCl) or acetic anhydride (Ac_2O) was added dropwise at 0°C. After stirring overnight at room temperature, the solvent was removed in vacuo. The residue was diluted with EtOAc, washed with water (3×100 mL), and saturated NH_4Cl aqueous solution (3×100 mL). The organic phase was dried over Na_2SO_4 and filtered. The solvent was removed in vacuo to get the crude product. Then 33% HBr/AcOH was added dropwise to a solution of the crude product in CH_2Cl_2 at 0°C. The reaction was monitored by TLC. After completion, the mixture was diluted with EtOAc, washed with water (3×50 mL), and saturated $NaHCO_3$ aqueous solution (3×50 mL). The organic layer was dried over Na_2SO_4 and filtered. The solvent was removed in vacuo to obtain the glycosyl bromides without further purification. The glycosyl bromides were used to synthesize glycosyl donors in the next step.

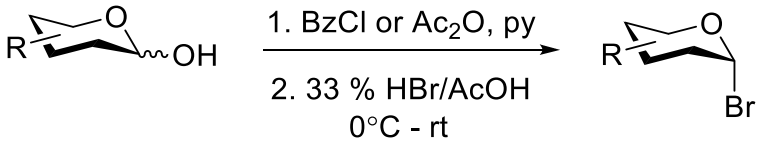

7-Oxocyclohepta-1,3,5-trien-1-yl 2,3,4,6-tetra-*O*-benzoyl-β-D-galactopyranoside (1c)

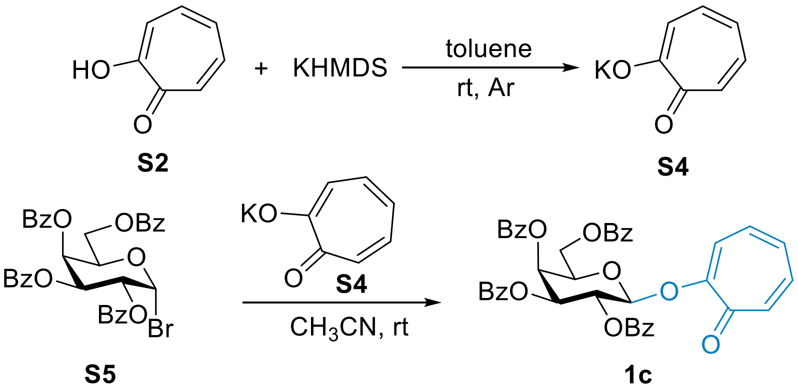

To a solution of **S2** (2.50 g, 20.0 mmol) in anhydrous toluene (20.0 mL) was added KHMDS (20.0 mL, 20.0 mmol) slowly under argon atmosphere. The mixture was stirred at room temperature for one hour. The reaction solution was filtered and washed with toluene to obtain **S4** as a yellow solid.

S5 was prepared from D-galactose (4.50 g, 25.0 mmol) using **General Procedure A**. The crude product **S5** (14.47 g, 22.00 mmol) and **S4** (4.22 g, 26.4 mmol) were dissolved in CH_3CN (50.0 mL), and the mixture was stirred at room temperature for 24 h [1]. The solvent was removed in vacuo. The residue was purified by flash column chromatography on silica gel (petroleum ether/EtOAc = 4/1) to obtain **1c** (14.00 g, 80%, over three steps) as a white solid. $[\alpha]_D^{25}$: 20.00 (*c*: 0.14 $CHCl_3$). **^{1}H NMR** (400 MHz, $CDCl_3$) δ 8.11 (d, *J* = 7.6 Hz, 2H), 8.02 - 7.96 (m, 4H), 7.82 (d, *J* = 7.7 Hz, 2H), 7.64 - 7.33 (m, 10H), 7.29 - 7.23 (m, 3H), 7.10 - 7.05 (m, 2H), 6.89 - 6.79 (m, 2H),

6.10 - 6.02 (m, 2H), 5.90 (d, *J* = 7.9 Hz, 1H), 5.70 (dd, *J* = 10.3, 3.5 Hz, 1H), 4.67 - 4.61 (m, 1H), 4.44 - 4.39 (m, 2H). **^{13}C NMR** (101 MHz, $CDCl_3$) δ 180.5, 165.8, 165.55, 165.52, 165.4, 161.3, 139.7, 135.8, 133.6, 133.3, 133.2, 133.1, 131.9, 131.4, 130.0, 129.9, 129.8, 129.7, 129.3, 128.9, 128.7, 128.6, 128.4, 128.3, 124.7, 98.0, 71.9, 71.6, 69.7, 68.0, 61.9. **HRMS** (ESI) Calculated for $C_{41}H_{32}O_{11}Na$ $[M+Na]^+$: 723.1842 Found: 723.1862.

7-Oxocyclohepta-1,3,5-trien-1-yl 2,3,4,6-tetra-*O*-benzoyl-β-D-glucopyranoside (1d)

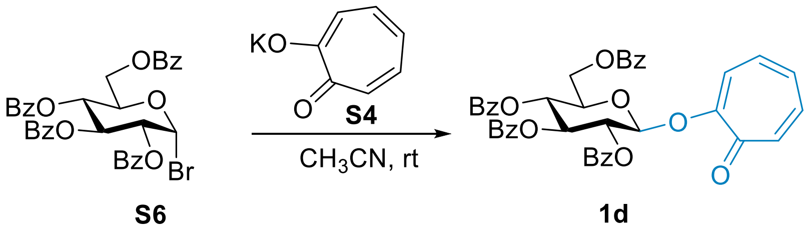

S6 was prepared from D-glucose (1.08 g, 6.00 mmol) using **General Procedure A**. The crude product **S6** (2.79 g, 4.24 mmol) and **S4** (0.81 g, 5.1 mmol) were dissolved in CH_3CN (12.5 mL). The mixture was stirred at room temperature for 24 h. The solvent was removed in vacuo and the residue was purified by flash column chromatography on silica gel (petroleum ether/EtOAc = 4/1) to obtain product **1d** (2.20 g, 53%, over three steps) as a pale-yellow solid. $[\alpha]_D^{25}$: -60.00 (*c*: 0.13 $CHCl_3$). **^{1}H NMR** (400 MHz, $CDCl_3$) δ 8.03 - 7.85 (m, 8H), 7.56 - 7.28 (m, 12H), 7.22 - 7.21 (m, 1H), 7.08 - 7.04 (m, 2H), 6.88 - 6.78 (m, 2H), 6.03 - 5.97 (m, 2H), 5.80 - 5.69 (m, 2H), 4.57 (dd, *J* = 12.1, 3.2 Hz, 1H), 4.49 (dd, *J* = 12.2, 5.8 Hz, 1H), 4.26 - 4.21 (m, 1H). **^{13}C NMR** (101 MHz, $CDCl_3$) δ 180.7, 165.9, 165.7, 165.3, 165.2, 161.0, 139.8, 135.9, 133.5, 133.2, 133.19, 133.12, 132.0, 131.6, 130.0, 129.88, 129.82, 129.7, 129.5, 129.2, 128.8, 128.6, 128.4, 128.35, 128.33, 125.4, 97.3, 72.7, 72.6, 72.0, 69.6, 62.8. **HRMS** (ESI) Calculated for $C_{41}H_{32}O_{11}Na$ $[M+Na]^+$: 723.1842 Found: 723.1844.

7-Oxocyclohepta-1,3,5-trien-1-yl 2,3,4,6-tetra-*O*-benzoyl-α-D-mannopyranoside (1e)

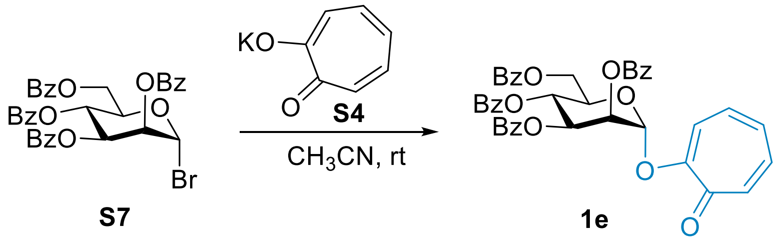

S7 was prepared from D-mannose (0.54 g, 3.0 mmol) using **General Procedure A**. The crude product **S7** (1.57 g, 2.39 mmol) and **S4** (0.46 g, 2.9 mmol) were dissolved in CH_3CN (7.0 mL). The mixture was stirred at room temperature for 24 h. The solvent was removed in vacuo and the residue was purified by flash column chromatography on silica gel (petroleum ether/EtOAc = 4/1) to obtain product **1e** (1.40 g, 66%, over three steps) as a white solid. $[\alpha]_D^{25}$: 33.85 (*c*: 0.13 $CHCl_3$). **^{1}H NMR** (400 MHz, $CDCl_3$) δ 8.09 - 8.07 (m, 2H), 8.04 - 7.97 (m,

4H), 7.86 - 7.84 (m, 2H), 7.62 - 7.49 (m, 3H), 7.45 - 7.35 (m, 7H), 7.28 (d, *J* = 7.4 Hz, 2H), 7.25 - 7.15 (m, 3H), 6.93 - 6.89 (m, 2H), 6.22 - 6.13 (m, 2H), 6.06 - 6.02 (m, 2H), 4.71 - 4.63 (m, 2H), 4.47 (dd, *J* = 12.2, 5.0 Hz, 1H). **^{13}C NMR** (101 MHz, $CDCl_3$) δ 180.6, 165.9, 165.5, 165.2, 161.3, 139.4, 136.1, 133.58, 133.54, 133.1, 133.0, 131.8, 131.2, 129.9, 129.77, 129.74, 129.1, 129.0, 128.8, 128.6, 128.48, 128.40, 128.3, 121.8, 96.2, 70.3, 70.1, 69.6, 66.7, 62.7. **HRMS** (ESI) Calculated for $C_{41}H_{32}O_{11}Na$ $[M+Na]^+$: 723.1842 Found: 723.1842.

7-Oxocyclohepta-1,3,5-trien-1-yl 2,3,4-tri-*O*-benzoyl-β-D-arabinopyranoside (1f)

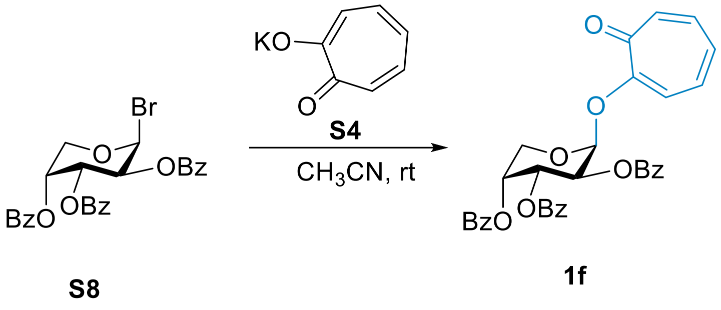

S8 was prepared from D-arabinose (0.60 g, 4.00 mmol) using **General Procedure A**. The crude product **S8** (1.52 g, 2.90 mmol) and **S4** (0.56 g, 3.5 mmol) were dissolved in CH_3CN (10.0 mL), and the mixture was stirred at room temperature for 24 h. The solvent was removed in vacuo and the residue was purified by flash column chromatography on silica gel (petroleum ether/EtOAc = 3/1) to obtain product **1f** (1.42 g, 63%, over three steps) as a white solid. $[\alpha]_D^{25}$: -311.11 (*c*: 0.14 $CHCl_3$). **^{1}H NMR** (400 MHz, $CDCl_3$) δ 8.12 - 8.09 (m, 2H), 8.05 - 8.02 (m, 2H), 7.88 - 7.86 (m, 2H), 7.63 - 7.58 (m, 1H), 7.52 - 7.43 (m, 4H), 7.39 - 7.35 (m, 2H), 7.30 - 7.26 (m, 2H), 7.18 - 7.16 (m, 3H), 7.00 - 6.89 (m, 2H), 6.21 (d, *J* = 3.6 Hz, 1H), 6.21 (dd, *J* = 10.6, 3.5 Hz, 1H), 5.93 (dd, *J* = 10.6, 3.6 Hz, 1H), 5.88 - 5.87 (m, 1H), 4.44 (dd, *J* = 13.3, 1.4 Hz, 1H), 4.06 (dd, *J* = 13.2, 2.0 Hz, 1H). **^{13}C NMR** (101 MHz, $CDCl_3$) δ 180.5, 166.2, 165.7, 165.4, 162.3, 139.2, 136.0, 133.49, 133.40, 133.2, 132.0, 130.8, 130.0, 129.8, 129.7, 129.5, 129.2, 129.1, 128.6, 128.4, 128.3, 121.3, 96.0, 69.8, 68.7, 68.0, 62.1. **HRMS** (ESI) Calculated for $C_{33}H_{26}O_9Na$ $[M+Na]^+$: 589.1475 Found: 589.1467.

7-Oxocyclohepta-1,3,5-trien-1-yl 2,3,4-tri-*O*-benzoyl-α-D-xylopyranoside (1g)

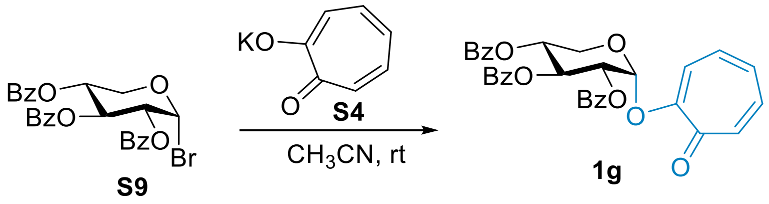

S9 was prepared from D-xylose (0.60 g, 4.0 mmol) using **General Procedure A**. The crude product **S9** (1.54 g, 2.94 mmol) and **S4** (0.56 g, 3.5 mmol) were dissolved in CH_3CN (10.0 mL), and the mixture was stirred at room temperature for 24 h. The solvent was removed in vacuo and the residue was purified by flash column

chromatography on silica gel (petroleum ether/EtOAc = 3/1) to obtain product **1g** (1.45 g, 64%, over three steps) as a white solid. $[\alpha]_D^{25}$: 164.67 (*c*: 0.15 $CHCl_3$). **^{1}H NMR** (400 MHz, $CDCl_3$) δ 8.05 - 7.93 (m, 6H), 7.55 - 7.30 (m, 9H), 7.20 - 7.14 (m, 3H), 6.98 - 6.87 (m, 2H), 6.42 (t, *J* = 9.9 Hz, 1H), 6.16 (d, *J* = 3.6 Hz, 1H), 5.52 - 5.46 (m, 2H), 4.20 - 4.09 (m, 2H). **^{13}C NMR** (101 MHz, $CDCl_3$) δ 180.5, 166.0, 165.6, 165.5, 162.1, 139.4, 136.0, 133.5, 133.3, 133.2, 131.9, 131.1, 130.0, 129.9, 129.7, 129.2, 129.0, 128.9, 128.5, 128.4, 128.3, 122.3, 95.1, 71.2, 69.9, 69.7, 60.1. **HRMS** (ESI) Calculated for $C_{33}H_{26}O_9Na$ $[M+Na]^+$: 589.1475 Found: 589.1475.

7-Oxocyclohepta-1,3,5-trien-1-yl 2,3,4-tri-*O*-benzoyl-α-L-rhamnopyranoside (1h)

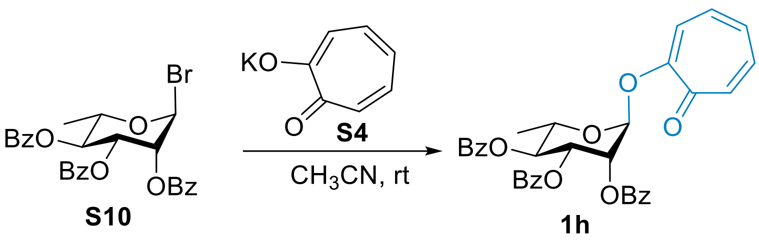

S10 was prepared from L-rhamnose (0.98 g, 6.0 mmol) using **General Procedure A**. The crude product **S10** (2.62 g, 4.87 mmol) and **S4** (0.93 g, 5.8 mmol) were dissolved in CH_3CN (10.0 mL), and the mixture was stirred at room temperature for 24 h. The solvent was removed in vacuo and the residue was purified by flash column chromatography on silica gel (petroleum ether/EtOAc = 3/1) to obtain product **1h** (2.54 g, 73%, over three steps) as a white solid. $[\alpha]_D^{25}$: 1.25 (*c*: 0.16 $CHCl_3$). **^{1}H NMR** (400 MHz, $CDCl_3$) δ 8.14 - 8.11 (m, 2H), 8.01 - 7.98 (m, 2H), 7.85 - 7.83 (m, 2H), 7.63 - 7.36 (m, 7H), 7.27 - 7.19 (m, 5H), 7.05 - 6.92 (m, 2H), 6.12 (dd, *J* = 10.1, 3.5 Hz, 1H), 6.02 (dd, *J* = 3.5, 1.9 Hz, 1H), 5.97 (d, *J* = 1.8 Hz, 1H), 5.77 (t, *J* = 10.0 Hz, 1H), 4.46 - 4.39 (m, 1H), 1.35 (d, *J* = 6.3 Hz, 3H). **^{13}C NMR** (101 MHz, $CDCl_3$) δ 180.6, 165.8, 165.4, 165.2, 161.6, 139.2, 136.2, 133.6, 133.4, 133.1, 132.1, 130.9, 129.9, 129.8, 129.7, 129.24, 129.20, 129.1, 128.6, 128.4, 128.2, 121.0, 96.1, 71.5, 70.4, 69.6, 68.3, 17.7. **HRMS** (ESI) Calculated for $C_{34}H_{28}O_9Na$ $[M+Na]^+$: 603.1631 Found: 603.1627.

7-Oxocyclohepta-1,3,5-trien-1-yl 2,3,4-tri-*O*-benzoyl-β-D-ribopyranoside (1i)

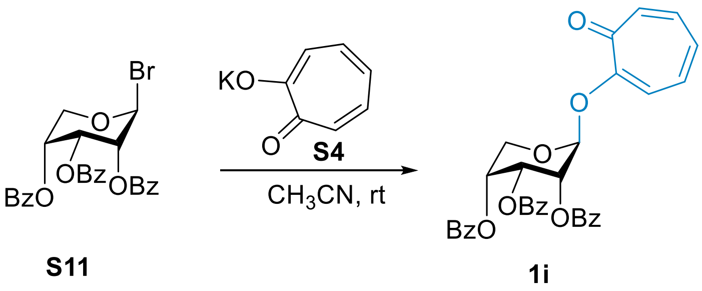

S11 was prepared from D-ribose (0.60 g, 4.0 mmol) using **General Procedure A**. The crude product **S11** (1.45 g, 2.76 mmol) and **S4** (0.53 g, 3.3 mmol) were dissolved in CH_3CN (10.0 mL), and the mixture was stirred at room

temperature for 24 h. The solvent was removed in vacuo and the residue was purified by flash column chromatography on silica gel (petroleum ether/EtOAc = 3/1) to obtain product **1i** (1.31 g, 58%, over three steps) as a white solid. $[\alpha]_D^{25}$: -127.67 (*c*: 0.15 $CHCl_3$). **^{1}H NMR** (400 MHz, $CDCl_3$) δ 8.08 - 8.05 (m, 2H), 8.02 - 8.00 (m, 2H), 7.89 - 7.87 (m, 2H), 7.57 - 7.46 (m, 3H), 7.32 - 7.22 (m, 9H), 7.05 - 6.94 (m, 2H), 6.10 (d, *J* = 2.3 Hz, 1H), 6.06 (d, *J* = 3.9 Hz, 1H), 5.86 - 5.84 (m, 1H), 5.75 - 5.72 (m, 1H), 4.42 (dd, *J* = 13.2, 2.1 Hz, 1H), 4.16 (dd, *J* = 13.2, 2.6 Hz, 1H). **^{13}C NMR** (101 MHz, $CDCl_3$) δ 180.6, 166.1, 165.8, 165.0, 161.5, 139.2, 136.2, 133.3, 133.2, 133.1, 132.1, 130.9, 130.0, 129.9, 129.8, 129.7, 129.5, 129.3, 128.39, 128.35, 121.1, 96.8, 68.2, 67.3, 65.9, 62.5. **HRMS** (ESI) Calculated for $C_{33}H_{26}O_9Na$ $[M+Na]^+$: 589.1475 Found: 589.1466.

7-Oxocyclohepta-1,3,5-trien-1-yl 3,4,6-*O*-tri-benzoyl-2-deoxy-2-phthalimido-β-D-glucopyranoside (1j)

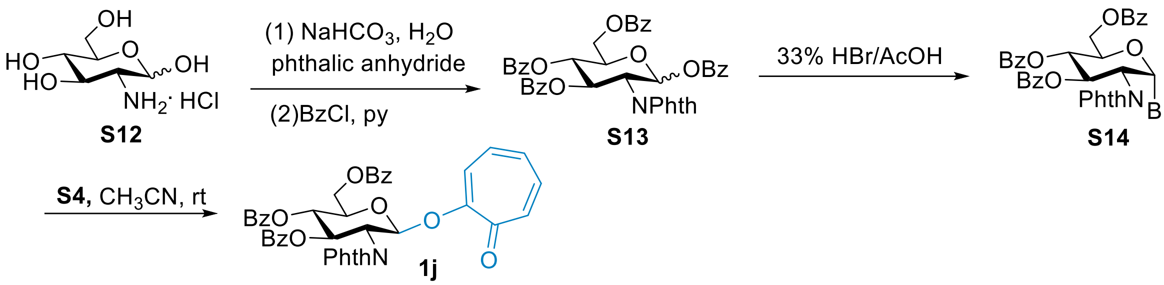

To a solution of **S12** (2.50 g, 11.5 mmol) in H_2O (15.0 mL) was added $NaHCO_3$ (4.00 g, 47.5 mmol). After stirring for 30 min at room temperature, phthalic anhydride (4.00 g, 27.0 mmol) was added. The mixture was kept at 40°C until giving a clear yellow solution. Then the yellow solution was concentrated. The residue was co-evaporated with toluene, and dissolved in pyridine (15.0 mL). BzCl (2.30 mL) was added, and the mixture was kept at room temperature while monitoring the progress of the reaction using TLC (petroleum ether/EtOAc = 1/1) until its completion. The mixture was diluted with EtOAc, washed with water (3×30 mL), saturated NH_4Cl aqueous solution (3×30 mL). The organic phase was dried over Na_2SO_4 and filtered. The solvent was removed in vacuo to get **S13**. 33% HBr/AcOH (5.0 mL) was added dropwise at 0°C to a solution of the crude product in CH_2Cl_2 (3.0 mL), after stirring for 1h. The mixture was diluted with EtOAc, washed with water (3×30 mL), saturated $NaHCO_3$ aqueous solution (3×30 mL). The organic layer was collected, dried over Na_2SO_4, and filtered. The solvent was removed in vacuo to give **S14** without further purification.

The crude product **S14** and **S4** (1.30 g, 8.00 mmol) were dissolved in CH_3CN (20.0 mL). The mixture was stirred at room temperature for 36 h. The solvent was removed in vacuo and the residue was purified by flash column chromatography on silica gel (petroleum ether/EtOAc = 1/1) to obtain **1j** (3.90 g, 60%, over four steps) as a yellow solid. $[\alpha]_D^{25}$: 64.00 (*c*: 0.13 $CHCl_3$). **^{1}H NMR** (400 MHz, $CDCl_3$) δ 8.01 - 7.99 (m, 2H), 7.93 - 7.90 (m, 2H), 7.83 - 7.78 (m, 4H), 7.68 - 7.66 (m, 2H), 7.58 - 7.54 (m, 1H), 7.51 - 7.47 (m, 1H), 7.44 - 7.39 (m, 3H), 7.36 - 7.32 (m, 2H), 7.29 - 7.27 (m, 2H), 7.23 - 7.21 (m, 1H), 7.09 - 6.98 (m, 2H), 6.86 - 6.81 (m, 1H), 6.74 (t, *J* = 10.2 Hz, 1H), 6.49 (dd, *J* = 10.7, 9.2 Hz, 1H), 6.13 (d, *J* = 8.4 Hz, 1H), 5.73 (t, *J* = 9.7 Hz, 1H), 4.89 (dd, *J* = 10.7, 8.4 Hz, 1H),

4.62 - 4.51 (m, 2H), 4.34 - 4.29 (m, 1H). **^{13}C NMR** (101 MHz, $CDCl_3$) δ 180.5, 165.9, 165.5, 165.2, 161.5, 140.0, 135.8, 134.0, 133.4, 133.2, 133.1, 131.79, 131.75, 129.9, 129.8, 129.7, 129.6, 128.7, 128.6, 128.42, 128.40, 128.3, 125.3, 123.6, 96.5, 72.3, 70.6, 70.2, 62.9, 54.7. **HRMS** (ESI) Calculated for $C_{42}H_{32}NO_{11}$ $[M+H]^+$: 726.1975 Found: 726.1965.

7-Oxocyclohepta-1,3,5-trien-1-yl 2,3,4,6-tetra-*O*-acetyl-β-D-galactopyranoside (1k)

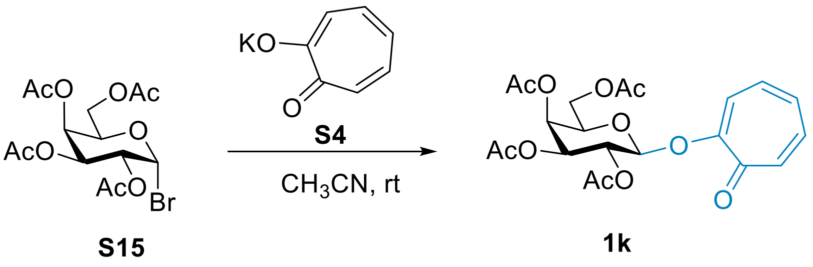

S15 was prepared from D-galactose (0.90 g, 5.0 mmol) using **General Procedure A**. The crude product **S15** (1.69 g, 4.11 mmol) and **S4** (0.79 g, 4.9 mmol) were dissolved in CH_3CN (20.0 mL), and the mixture was stirred at room temperature for 24 h. The solvent was removed in vacuo and the residue was purified by flash column chromatography on silica gel (petroleum ether/EtOAc = 2/1) to obtain product **1k** (1.67 g, 74%, over three steps) as a white solid. $[\alpha]_D^{25}$: -160.45 (*c*: 0.22 $CHCl_3$). **^{1}H NMR** (400 MHz, $CDCl_3$) δ 7.24 - 7.16 (m, 3H), 6.99 - 6.97 (m, 2H), 5.50 (dd, *J* = 10.4, 7.9 Hz, 1H), 5.44 - 5.43 (m, 1H), 5.37 (dd, *J* = 8.0, 1.0 Hz, 1H), 5.12 (dd, *J* = 10.4, 3.4 Hz, 1H), 4.21 - 4.08 (m, 2H), 3.99 - 3.96 (m, 1H), 2.17 (s, 3H), 2.12 (s, 3H), 2.02 (d, *J* = 2.2 Hz, 6H). **^{13}C NMR** (101 MHz, $CDCl_3$) δ 180.6, 170.2, 170.1, 170.0, 169.8, 161.5, 139.8, 136.1, 132.0, 131.7, 124.6, 98.6, 71.1, 70.6, 68.6, 66.8, 61.0, 20.8, 20.65, 20.62, 20.5. **HRMS** (ESI) Calculated for $C_{21}H_{24}O_{11}Na$ $[M+Na]^+$: 475.1216 Found: 475.1208.

7-Oxocyclohepta-1,3,5-trien-1-yl 2,3,4,6-tetra-*O*-acetyl-β-D-glucopyranoside (1l)

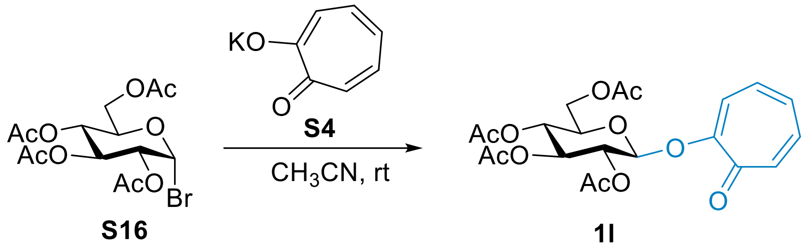

S16 was prepared from D-glucose (0.72 g, 4.0 mmol) using **General Procedure A**. The crude product **S16** (1.19 g, 2.91 mmol) and **S4** (0.56 g, 3.5 mmol) were dissolved in CH_3CN (20.0 mL), and the mixture was stirred at room temperature for 24 h. The solvent was removed in vacuo and the residue was purified by flash column chromatography on silica gel (petroleum ether/EtOAc = 2/1) to obtain product **1l** (0.92 g, 51%) as a white solid.

$[\alpha]_D^{25}$: -172.00 (*c*: 0.15 $CHCl_3$). **^{1}H NMR** (400 MHz, $CDCl_3$) δ 7.22 - 7.19 (m, 3H), 6.99 - 6.96 (m, 2H), 5.48 - 5.46 (m, 1H), 5.31 - 5.24 (m, 2H), 5.17 - 5.12 (m, 1H), 4.25 (dd, *J* = 12.3, 4.9 Hz, 1H), 4.11 (dd, *J* = 12.3, 2.5 Hz, 1H), 3.79 - 3.75 (m, 1H), 2.10 (s, 3H), 2.05 (s, 3H), 2.04 (d, *J* = 0.8 Hz, 6H). **^{13}C NMR** (101 MHz, $CDCl_3$) δ 180.7, 170.4, 170.1, 169.7, 169.4, 161.4, 139.9, 136.1, 132.0, 131.7, 124.9, 97.9, 72.4, 72.1, 71.2, 68.3, 61.7, 20.7, 20.69, 20.63, 20.60. **HRMS** (ESI) Calculated for $C_{21}H_{24}O_{11}Na$ $[M+Na]^+$: 475.1216 Found: 475.1212.

7-Oxocyclohepta-1,3,5-trien-1-yl 2,3,4,6-tetra-*O*-benzyl-β-D-galactopyranoside (1m)

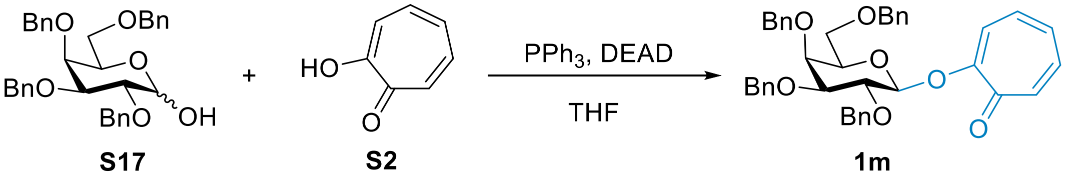

To a solution of **S17** (1.62 g, 3.00 mmol), **S2** (0.55 g, 4.5 mmol) and PPh_3 (1.56 g, 6.00 mmol) in dry THF (15.0 mL), diethylazodicarboxylate (DEAD, 0.95 mL, 6.0 mmol) was added slowly at 0°C under argon atmosphere. Then the mixture was stirred at room temperature and the progress of the reaction was monitored by TLC. Upon completion, the reaction mixture was concentrated in vacuo. The residue was purified by flash column chromatography on silica gel (petroleum ether/EtOAc = 4/1) to obtain product **1m** (0.96 g, 50%) as a yellow syrup. $[\alpha]_D^{25}$: -34.21 (*c*: 0.19 $CHCl_3$). **^{1}H NMR** (400 MHz, $CDCl_3$) δ 7.40 - 7.30 (m, 11H), 7.29 - 7.10 (m, 12H), 6.88 - 6.83 (m, 2H), 5.25 (d, *J* = 10.5 Hz, 1H), 5.04 (d, *J* = 7.6 Hz, 1H), 4.98 (d, *J* = 11.6 Hz, 1H), 4.89 (d, *J* = 10.5 Hz, 1H), 4.85 (d, *J* = 12.0 Hz, 1H), 4.76 (d, *J* = 11.9 Hz, 1H), 4.60 (d, *J* = 11.5 Hz, 1H), 4.47 - 4.39 (m, 2H), 4.26 (dd, *J* = 9.8, 7.6 Hz, 1H), 3.91 (d, *J* = 2.9 Hz, 1H), 3.71 - 3.55 (m, 4H). **^{13}C NMR** (101 MHz, $CDCl_3$) δ 180.1, 163.4, 138.6, 138.59, 138.52, 138.4, 137.9, 136.0, 132.4, 129.5, 128.7, 128.5, 128.4, 128.3, 128.2, 128.1, 127.8, 127.7, 127.66, 127.62, 127.60, 117.7, 101.7, 81.6, 78.5, 75.3, 74.6, 74.4, 73.6, 73.3, 69.0. **HRMS** (ESI) Calculated for: $C_{41}H_{40}O_7Na$ $[M+Na]^+$: 667.2672 found: 667.2664.

7-Oxocyclohepta-1,3,5-trien-1-yl 2,3-di-*O*-benzoyl-4-*O*-benzyl-6-*O*-*tert*-butyldiphenylsilyl-β-D-glucopyranoside (1n)

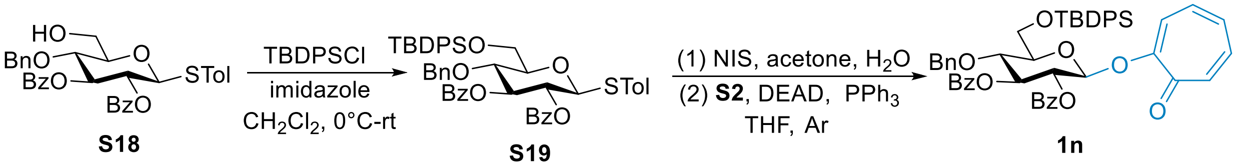

To the solution of thioglucopyranoside **S18**[2] (3.85 g, 6.60 mmol) and imidazole (0.90 g, 13 mmol) in CH_2Cl_2 was added TBDPSCl (3.40 mL, 13.2 mmol) at 0°C. Then the mixture was stirred at room temperature overnight. The reaction was quenched by MeOH, diluted with CH_2Cl_2, and washed with water. The combined organic layers were dried over Na_2SO_4 and filtered. The solvent was removed in vacuo to get the crude product **S19** (4.55 g, 5.53

mmol). To a solution of **S19** in H_2O (5.0 mL) and acetone (25.0 mL), NIS (3.73 g, 16.6 mmol) was added at 0°C. Then the reaction was stirred at room temperature and monitored by TLC. The reaction was quenched by saturated $Na_2S_2O_3$, diluted with CH_2Cl_2, and washed with water (3×100 mL). The combined organic layers were dried over Na_2SO_4 and filtered. The solvent was removed in vacuo. To a solution of the residue, **S2** (0.69 g, 5.6 mmol) and PPh_3 (2.46 g, 9.40 mmol) in THF (40.0 mL), DEAD (1.48 mL, 9.40 mmol) was added at 0°C under argon atmosphere. Then the mixture was stirred at room temperature under an argon atmosphere. After stirring for 5 h at room temperature, the solvent was removed in vacuo. The residue was purified by flash chromatography on silica gel (petroleum ether/EtOAc = 5/1) to give **1n** (2.00 g, 36%) as a pale-yellow solid. $[\alpha]_D^{25}$: 4.76 (*c*: 0.21 $CHCl_3$). **^{1}H NMR** (400 MHz, $CDCl_3$) δ 8.00 (t, *J* = 7.0 Hz, 4H), 7.66 (t, *J* = 6.7 Hz, 4H), 7.52 - 7.24 (m, 13H), 7.17 - 7.11 (m, 4H), 7.08 - 7.02 (m, 3H), 6.86 - 6.84 (m, 2H), 5.84 - 5.75 (m, 2H), 5.68 (dd, *J* = 9.5, 7.6 Hz, 1H), 4.62 (s, 2H), 4.12 (t, *J* = 9.3 Hz, 1H), 3.95 (d, *J* = 2.9 Hz, 2H), 3.71 - 3.68 (m, 1H), 1.07 (s, 9H). **^{13}C NMR** (101 MHz, $CDCl_3$) δ 180.6, 165.7, 165.5, 161.9, 139.6, 137.2, 135.8, 135.5, 133.3, 133.1, 132.9, 132.8, 132.1, 131.0, 129.9, 129.8, 129.7, 129.6, 129.56, 129.50, 128.4, 128.37, 128.32, 128.2, 127.99, 127.96, 127.8, 127.7, 127.6, 123.6, 97.8, 76.3, 75.7, 74.97, 74.94, 72.3, 62.3, 26.8, 19.3. **HRMS** (ESI) Calculated for $C_{50}H_{48}NaO_9Si$ $[M+Na]^+$: 843.2965 Found: 843.2964.

7-Oxocyclohepta-1,3,5-trien-1-yl hepta-*O*-benzoyl-β-D-lactoside (1o)

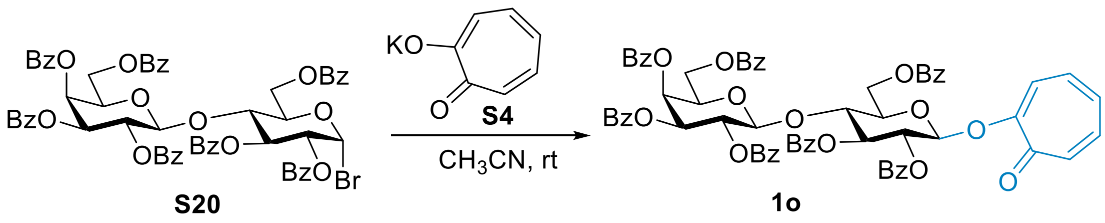

S20 was prepared from D-lactose (1.36 g, 4.00 mmol) using **General Procedure A**. The crude product **S20** (3.92 g, 3.46 mmol) and **S4** (0.66 g, 4.2 mmol) were dissolved in CH_3CN (10.0 mL), and the mixture was stirred at room temperature for 24 h. The solvent was removed in vacuo and the residue was purified by flash column chromatography on silica gel (petroleum ether/EtOAc = 3/1) to obtain **1o** (3.66 g, 78%) as a pale-yellow solid. $[\alpha]_D^{25}$: 3.04 (*c*: 0.23 $CHCl_3$). **^{1}H NMR** (400 MHz, $CDCl_3$) δ 8.07 - 7.98 (m, 6H), 7.95 - 7.88 (m, 6H), 7.73 (d, *J* = 7.8 Hz, 2H), 7.64 - 7.45 (m, 8H), 7.41 - 7.33 (m, 7H), 7.28 - 7.25 (m, 2H), 7.22 - 7.17 (m, 4H), 7.07 - 6.99 (m, 3H), 6.80 - 6.71 (m, 2H), 5.88 (t, *J* = 8.7 Hz, 1H), 5.79 (d, *J* = 7.2 Hz, 1H), 5.75 - 5.68 (m, 3H), 5.40 (dd, *J* = 10.4, 3.4 Hz, 1H), 4.91 (d, *J* = 7.9 Hz, 1H), 4.59 (dd, *J* = 12.2, 2.0 Hz, 1H), 4.46 - 4.36 (m, 2H), 3.99 - 3.95 (m, 1H), 3.91 (t, *J* = 6.7 Hz, 1H), 3.75 (d, *J* = 6.6 Hz, 2H). **^{13}C NMR** (101 MHz, $CDCl_3$) δ 180.4, 165.6, 165.5, 165.4, 165.3, 165.2, 164.8, 161.2, 139.5, 135.8, 133.5, 133.3, 133.2, 133.1, 131.8, 131.1, 130.0, 129.78, 129.75, 129.73, 129.6, 129.5, 129.4, 129.2, 128.8, 128.67, 128.64, 128.60, 128.4, 128.3, 128.2, 123.5, 101.2, 97.1, 76.0, 73.2, 73.0, 71.9, 71.7, 71.4, 69.9, 67.5, 62.0, 61.0. **HRMS** (ESI) Calculated for $C_{68}H_{54}O_{19}Na$ $[M+Na]^+$: 1197.3157 Found: 1197.3167.

7-Oxocyclohepta-1,3,5-trien-1-yl 2,3,4-tri-*O*-benzoyl-β-D-arabinofuranoside (1p)

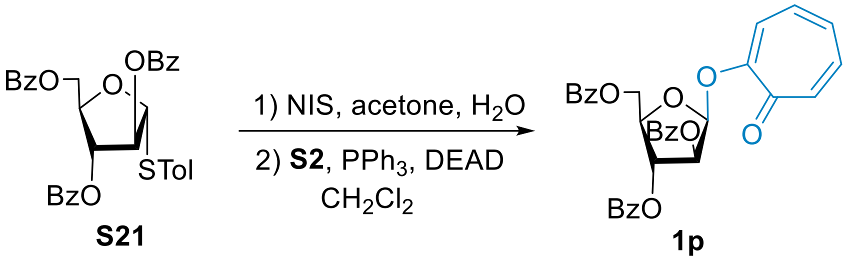

To a solution of **S21**[3] (1.13 g, 2.00 mmol) in H_2O (1.0 mL) and acetone (5.0 mL), NIS (1.35 g, 6.00 mmol) was added at 0°C. Then the mixture was stirred at room temperature and monitored by TLC. The reaction was quenched by saturated $Na_2S_2O_3$ solution, diluted with CH_2Cl_2, and washed with water (3×100 mL). The combined organic layers were dried over Na_2SO_4 and filtered. The solvent was removed in vacuo. To a solution of the residue, **S2** (0.24 g, 2.0 mmol) and PPh_3 (0.85 g, 3.3 mmol) in THF (10.0 mL), DEAD (0.51 mL, 3.3 mmol) was added at 0°C under argon atmosphere. Then the mixture was stirred at room temperature under an argon atmosphere. After stirring for 5 h at room temperature, the solvent was removed in vacuo. The residue was purified by flash chromatography on silica gel (petroleum ether/EtOAc = 6/1) to give **1p** (452.9 mg, 40%) as a white solid. $[\alpha]_D^{25}$: -72.96 (*c*: 0.27 $CHCl_3$). **^{1}H NMR** (400 MHz, $CDCl_3$) δ 8.16 - 8.13 (m, 2H), 8.07 - 8.04 (m, 2H), 8.00 - 7.98 (m, 2H), 7.60 - 7.42 (m, 7H), 7.35 (t, *J* = 7.7 Hz, 2H), 7.17 - 7.15 (m, 1H), 7.05 - 7.01 (m, 2H), 6.88 - 6.79 (m, 2H), 6.22 - 6.16 (m, 2H), 5.81 (dd, *J* = 7.3, 4.6 Hz, 1H), 4.84 (dd, *J* = 11.8, 4.3 Hz, 1H), 4.67 (dd, *J* = 11.8, 6.3 Hz, 1H), 4.61 - 4.57 (m, 1H). **^{13}C NMR** (101 MHz, $CDCl_3$) δ 180.2, 166.1, 166.0, 165.7, 162.3, 139.0, 135.7, 133.6, 133.4, 133.0, 131.9, 130.19, 130.16, 129.9, 129.7, 129.6, 129.0, 128.9, 128.5, 128.4, 128.3, 120.4, 98.9, 79.8, 76.6, 75.7, 64.9. **HRMS** (ESI) Calculated for $C_{33}H_{26}NaO_9$ $[M+Na]^+$: 589.1475 Found: 589.1470.

7-Oxocyclohepta-1,3,5-trien-1-yl 2,3,4-tri-*O*-benzoyl-β-D-ribofuranoside (1q)

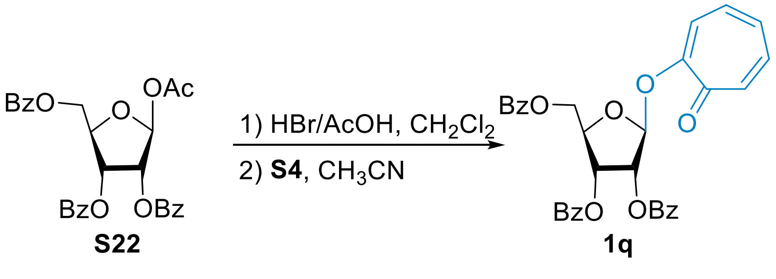

To a solution of **S22** (3.02 g, 6.00 mmol) in CH_2Cl_2, 33% HBr/AcOH (3.00 mL) was added at 0°C. The reaction mixture was stirred at room temperature (~2 h). The reaction was quenched by saturated $NaHCO_3$, diluted with CH_2Cl_2, and washed with water (3×50 mL). The combined organic layers were dried over Na_2SO_4 and filtered. The solvent was removed in vacuo. The residue and **S4** (0.58 g, 3.6 mmol) were dissolved in CH_3CN (20.0 mL), and the mixture was stirred for 24 h at room temperature. The solvent was removed in vacuo and the residue was

purified by flash column chromatography on silica gel (petroleum ether/EtOAc = 3/1) to obtain **1q** (1.53 g, 45%, over two steps) as a white solid. $[\alpha]_D^{25}$: -58.57 (*c*: 1.12 $CHCl_3$). **^{1}H NMR** (400 MHz, $CDCl_3$) δ 8.06 - 8.03 (m, 2H), 8.01 - 7.98 (m, 2H), 7.90 - 7.87 (m, 2H), 7.61 - 7.57 (m, 1H), 7.53 - 7.49 (m, 2H), 7.46 - 7.42 (m, 2H), 7.38 - 7.30 (m, 4H), 7.16 - 7.12 (m, 3H), 6.93 - 6.86 (m, 2H), 6.17 (s, 1H), 6.12 - 6.07 (m, 2H), 4.82 - 4.75 (m, 2H), 4.54 - 4.49 (m, 1H). **^{13}C NMR** (101 MHz, $CDCl_3$) δ 180.4, 166.0, 165.1, 164.9, 161.6, 139.0, 136.0, 133.5, 133.4, 133.1, 132.1, 130.3, 129.8, 129.77, 129.76, 129.4, 129.0, 128.8, 128.5, 128.39, 128.37, 120.6, 103.4, 80.3, 75.9, 71.6, 63.8. **HRMS** (ESI) Calculated for $C_{33}H_{26}NaO_9$ $[M+Na]^+$: 589.1475 Found: 589.1470.

7-Oxocyclohepta-1,3,5-trien-1-yl 2,3,4,6-tetra-*O*-benzyl-α/β-D-mannopyranoside (1r)

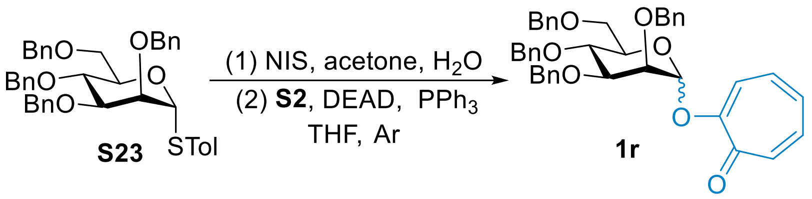

To a solution of **S23**[4] (0.65 g, 1.0 mmol) in H_2O (0.70 mL) and acetone (5.0 mL), NIS (0.67 g, 3.0 mmol) was added at 0°C. The reaction mixture was stirred at room temperature and monitored by TLC. The reaction was quenched by saturated $Na_2S_2O_3$, diluted with CH_2Cl_2, and washed with water (3×30 mL). The combined organic layers were dried over Na_2SO_4 and filtered. The solvent was removed in vacuo. To a solution of the residue, **S2** (0.15 g, 1.2 mmol) and PPh_3 (0.54 g, 2.1 mmol) in THF (5.0 mL), DEAD (0.32 mL, 2.1 mmol) was added at 0°C under argon atmosphere. Then the mixture was stirred at room temperature under an argon atmosphere. After stirring for 1 h at room temperature, the solvent was removed in vacuo. The residue was purified by flash chromatography on silica gel (petroleum ether/EtOAc = 6/1) to give **1r** (α/β = 1/3.2, 368.5 mg, 57%) as a pale-yellow solid. **^{1}H NMR** (400 MHz, $CDCl_3$) δ 7.61 - 7.59 (m, 6H), 7.43 - 7.25 (m, 71H), 7.22 - 7.15 (m, 20H), 6.90 - 6.88 (m, 8H), 5.73 (d, *J* = 1.8 Hz, 1H), 5.19 (d, *J* = 12.3 Hz, 3H), 5.15 - 5.12 (m, 6H), 4.94 (d, *J* = 10.9 Hz, 3H), 4.90 (d, *J* = 10.8 Hz, 1H), 4.79 (s, 2H), 4.66 - 4.40 (m, 21H), 4.26 (d, *J* = 3.0 Hz, 3H), 4.21 - 4.16 (m, 2H), 4.06 (t, *J* = 9.3 Hz, 1H), 3.92 (t, *J* = 9.5 Hz, 4H), 3.83 (dd, *J* = 10.9, 1.9 Hz, 3H), 3.77 - 3.67 (m, 5H), 3.59 - 3.53 (m, 6H). **^{13}C NMR** (101 MHz, $CDCl_3$) δ 180.9, 180.7, 162.9, 162.3, 138.93, 138.90, 138.7, 138.3, 138.26, 138.23, 138.1, 137.9, 136.2, 136.1, 132.5, 132.4, 130.3, 130.2, 128.6, 128.3, 128.29, 128.26, 128.1, 128.0, 127.9, 127.8, 127.7, 127.68, 127.66, 127.64, 127.61, 127.56, 127.54, 127.46, 127.40, 121.2, 120.6, 99.3, 96.9, 81.7, 79.6, 76.3, 75.1, 75.0, 74.5, 74.49, 74.41, 74.2, 73.5, 73.3, 73.2, 73.1, 72.7, 72.3, 71.3, 69.4, 69.0. **HRMS** (ESI) Calculated for $C_{41}H_{40}O_7Na$ $[M+Na]^+$: 667.2672 Found: 667.2665.

7-Oxocyclohepta-1,3,5-trien-1-yl 2,3,4-tri-*O*-benzyl-α-D-xylopyranoside (1s)

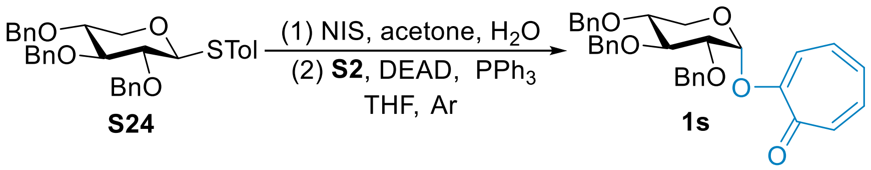

To a solution of **S24**[5] (0.51 g, 1.0 mmol) in H_2O (0.50 mL) and acetone (3.0 mL), NIS (0.67 g, 3.0 mmol) was added at 0°C. The reaction mixture was stirred at room temperature and monitored by TLC. The reaction was quenched by saturated $Na_2S_2O_3$, diluted with CH_2Cl_2, and washed with water (3×30 mL). The combined organic layers were dried over Na_2SO_4 and filtered. The solvent was removed in vacuo. To a solution of the residue, **S2** (0.14 g, 1.2 mmol) and PPh_3 (0.51 g, 1.9 mmol) in THF (5.0 mL), DEAD (0.31 mL, 1.9 mmol) was added at 0°C under argon atmosphere. Then the mixture was stirred at room temperature under an argon atmosphere. After stirring for 1 h at room temperature, the solvent was removed in vacuo. The residue was purified by flash chromatography on silica gel (petroleum ether/EtOAc = 6/1) to obtain **1s** (312.5 mg, 60%) as a pale-yellow syrup. $[\alpha]_D^{25}$: 4.34 (*c*: 0.23 $CHCl_3$). **^{1}H NMR** (400 MHz, $CDCl_3$) δ 7.41 - 7.30 (m, 8H), 7.29 - 7.19 (m, 9H), 7.11 - 7.08 (m, 1H), 6.95 - 6.92 (m, 2H), 5.93 (d, *J* = 3.5 Hz, 1H), 5.01 (d, *J* = 10.9 Hz, 1H), 4.91 (d, *J* = 11.2 Hz, 2H), 4.83 (d, *J* = 11.9 Hz, 1H), 4.78 (d, *J* = 11.6 Hz, 1H), 4.63 (d, *J* = 11.6 Hz, 1H), 4.18 (t, *J* = 9.0 Hz, 1H), 3.69 - 3.61 (m, 4H). **^{13}C NMR** (101 MHz, $CDCl_3$) δ 181.0, 162.3, 139.2, 138.9, 138.2, 138.1, 135.9, 132.2, 130.7, 128.4, 128.37, 128.34, 128.2, 128.0, 127.8, 127.78, 127.71, 127.5, 123.2, 94.8, 80.8, 79.2, 77.5, 75.8, 73.6, 72.9, 61.4. **HRMS** (ESI) Calculated for $C_{33}H_{32}O_6Na$ $[M+Na]^+$: 547.2097 Found: 547.2090.

7-Oxocyclohepta-1,3,5-trien-1-yl 2-azido-3,4,6-tri-*O*-benzyl-2-deoxy-α-D-glucopyranoside (1t)

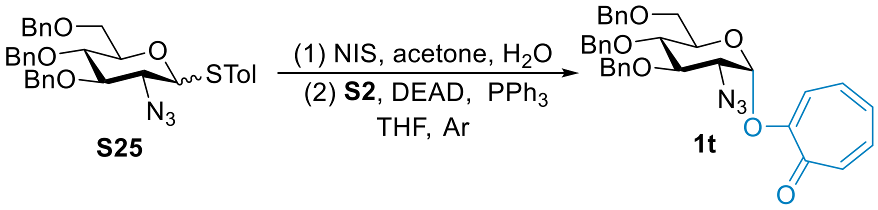

To a solution of **S25**[4] (0.58 g, 1.0 mmol) in H_2O (0.60 mL) and acetone (3.0 mL), NIS (0.67 g, 3.0 mmol) was added at 0°C. The reaction mixture was stirred at room temperature and monitored by TLC. The reaction was quenched by saturated $Na_2S_2O_3$, diluted with CH_2Cl_2, and washed with water (3×100 mL). The combined organic layers were dried over Na_2SO_4 and filtered. The solvent was removed in vacuo. To a solution of the residue, **S2** (139.1 mg, 1.140 mmol) and PPh_3 (498.2 mg, 1.900 mmol) in THF (5.0 mL), DEAD (0.29 mL, 1.9 mmol) was added at 0°C under argon atmosphere. Then the mixture was stirred at room temperature under an argon atmosphere. After stirring for 1 h at room temperature, the solvent was removed in vacuo. The residue was purified by flash chromatography on silica gel (petroleum ether/EtOAc = 5/1) to obtain **1t** (332.5 mg, 58%) as a pale-brown syrup. $[\alpha]_D^{25}$: 71.82 (*c*: 0.44 $CHCl_3$). **^{1}H NMR** (400 MHz, $CDCl_3$) δ 7.39 - 7.24 (m, 13H), 7.21 - 7.13 (m, 5H), 6.95 - 6.85 (m, 2H), 5.79 (d, *J* = 3.4 Hz, 1H), 4.99 - 4.91 (m, 2H), 4.84 (d, *J* = 11.0 Hz, 1H), 4.57 - 4.52 (m, 2H),

4.42 (d, J = 11.9 Hz, 1H), 4.34 - 4.29 (m, 1H), 4.00 - 3.96 (m, 1H), 3.81 (t, J = 9.5 Hz, 1H), 3.73 (dd, J = 11.0, 3.6 Hz, 1H), 3.63 - 3.57 (m, 2H). **^{13}C NMR** (101 MHz, $CDCl_3$) δ 180.4, 161.9, 139.1, 137.9, 137.8, 137.7, 136.0, 132.0, 130.5, 128.5, 128.45, 128.41, 128.1, 127.9, 127.84, 127.81, 127.6, 120.2, 96.5, 80.1, 77.9, 75.6, 75.0, 73.4, 72.2, 68.0, 63.4. **HRMS** (ESI) Calculated for $C_{34}H_{33}N_3O_6Na$ $[M+Na]^+$: 602.2267 Found: 602.2260.

4. Preparation of the acceptor 2r-x

The acceptors 2a-x and S17

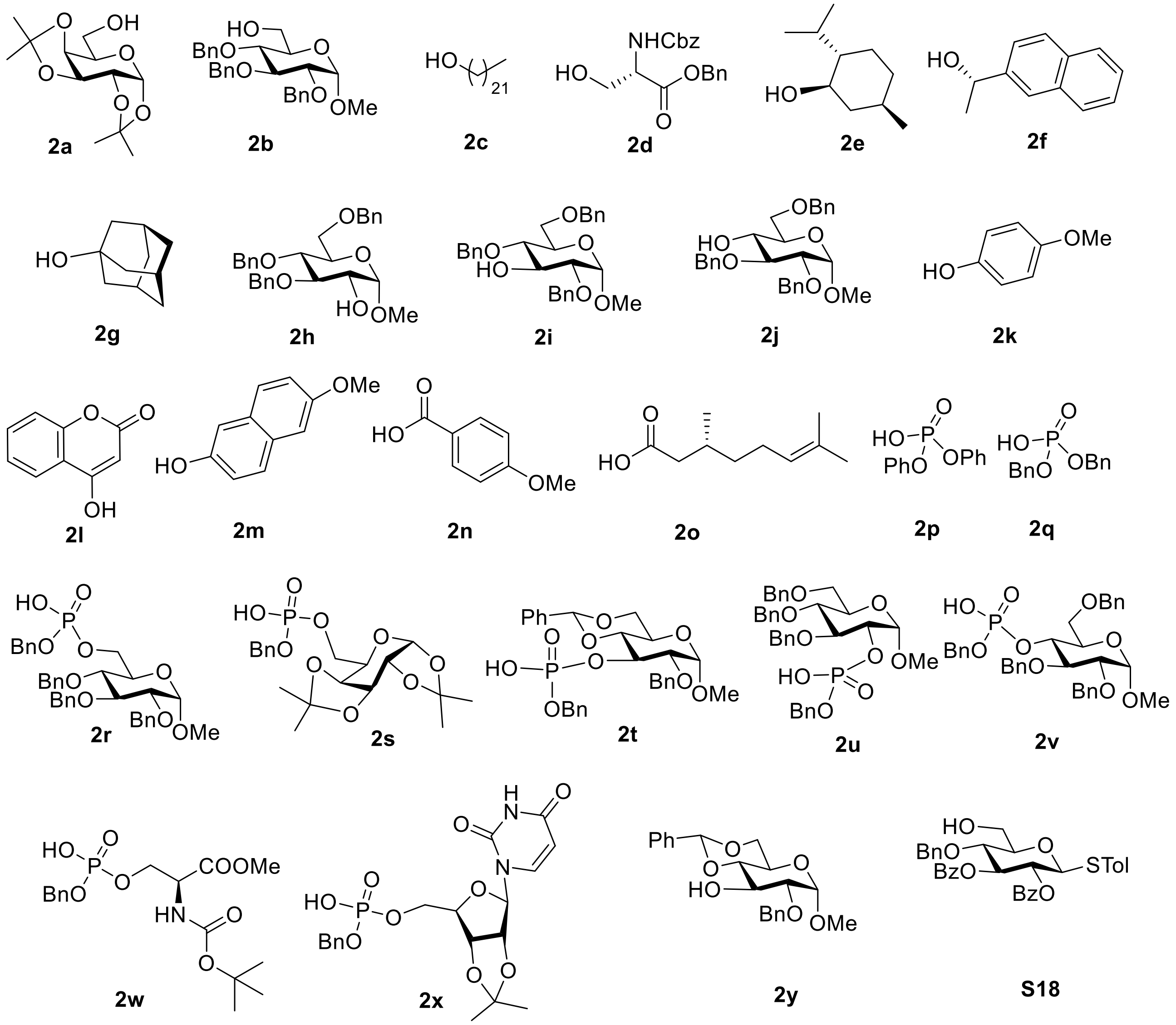

Compounds **2b**, **2h**, **2i**, **2j**, **2y**, **S17** were synthesized following the literature procedures[2,6,7,8]. Compounds **2a**, **2c-g**, **2k-q**, epiandrosterone, estradiol benzoate, β-estradiol, cholesterol, simvastatin, pyrimidine, purine and phosphoric acid are commercially available. Compounds **2r-x** were synthesized as the following methods [9].

Preparation acceptors of 2r-x

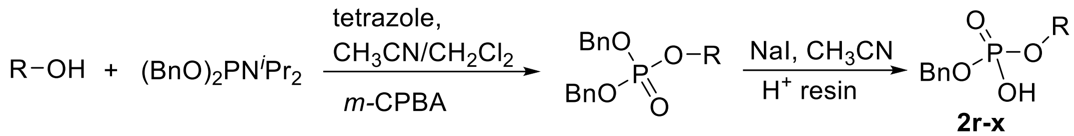

Methyl 2,3,4-tri-*O*-benzoyl-6-*O*-(benzyloxy(hydroxy)phosphoryl)-α-D-glucopyranoside (2r)

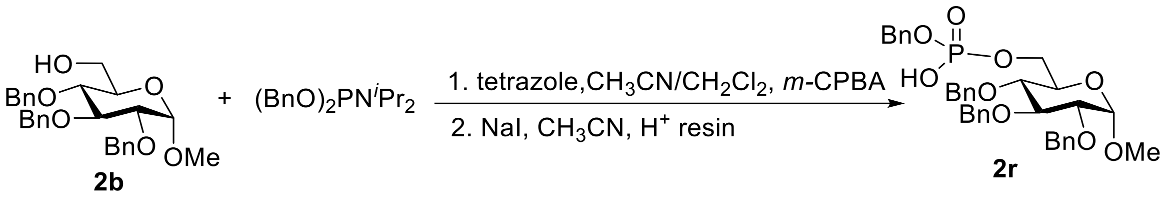

To a solution of **2b** (232.1 mg, 0.5000 mmol) in CH_3CN and CH_2Cl_2 (CH_3CN/CH_2Cl_2 = 1/4, 0.2 M) were added $(BnO)_2PN^iPr_2$ (0.25 mL, 0.75 mmol) and tetrazole (63.0 mg, 0.900 mmol), and the mixture was stirred at room temperature for 2 h. Then 3-chloroperoxybenzoic acid (*m*-CPBA, 345.1 mg, 2.000 mmol) was added to the mixture, and the reaction was stirred for 0.5 h, followed by quenching with $Na_2S_2O_3$ solution. After removing the CH_3CN by evaporation, the mixture was diluted with CH_2Cl_2, washed with H_2O, saturated $NaHCO_3$ and brine. The combined organic layers were dried over Na_2SO_4 and filtered. The filtrate was concentrated, and then purified via recrystallization to get the crude product.

The above crude product and NaI (300.0 mg, 2.000 mmol) were dissolved in CH_3CN (0.2 M), and the mixture was stirred at 60°C for 3 h. Then the solvent was removed by evaporation, and the mixture was extracted with EtOAc, washed with H_2O. The combined organic layers were dried over Na_2SO_4, filtered and concentrated. The residue was loaded to silica gel column which was neutralized by using hexanes/Et_3N (100/1) and purified by eluting with solvents (CH_2Cl_2/MeOH = 20/1) to give the product in the form of a phosphate salt. The phosphate salt in a mixed solvent of CH_2Cl_2/MeOH was acidified by H^+ resin, then the solution was filtered and concentrated to obtained **2r** as a colorless syrup (252.0 mg, 80%). $[\alpha]_D^{25}$: 3.63 (*c*: 0.11 $CHCl_3$). **^{1}H NMR** (400 MHz, $CDCl_3$) δ 7.33 - 7.26 (m, 16H), 7.25 - 7.19 (m, 4H), 5.00 - 4.95 (m, 3H) , 4.80 (t, *J* = 10.2 Hz, 2H), 4.73 (d, *J* = 12.1 Hz, 1H), 4.61 (d, *J* = 12.1 Hz, 1H), 4.57- 4.55 (m, 2H), 4.19 - 4.17 (m, 2H), 3.96 (t, *J* = 9.3 Hz, 1H), 3.72 (dd, *J* = 10.4, 2.6 Hz, 1H), 3.52 - 3.44 (m, 2H), 3.31 (s, 3H). **^{31}P NMR** (162 MHz, $CDCl_3$) δ 1.31. **^{13}C NMR** (101 MHz, $CDCl_3$) δ 138.7, 138.0, 137.9, 135.6 (d, *J* = 8.0 Hz), 128.5, 128.46, 128.43, 128.3, 128.07, 128.04, 127.9, 127.85, 127.80, 127.7, 127.5, 98.0, 81.8, 79.8, 75.6, 75.0, 73.3, 69.2 (d, *J* = 8.0 Hz), 69.1 (d, *J* = 5.0 Hz), 66.2 (d, *J* = 6.0 Hz), 55.2. **HRMS** (ESI) Calculated for $C_{35}H_{39}O_9PNa$ $[M+Na]^+$: 657.2229 Found:657.2225.

1,2:3,4-Di-*O*-isopropylidene-6-*O*-(benzyloxy(hydroxy)phosphoryl)-α-D-galactopyranoside (2s)

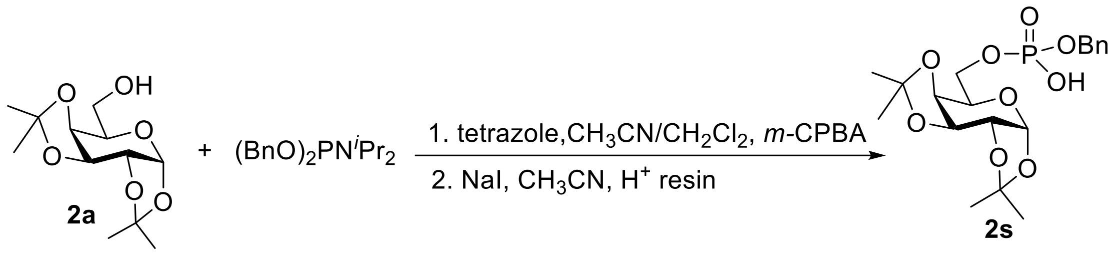

To a solution of **2a** (260.1 mg, 1.000 mmol) in CH_3CN and CH_2Cl_2 (CH_3CN/CH_2Cl_2 = 1/4, 0.2 M) were added $(BnO)_2PN^iPr_2$ (0.50 mL, 1.5 mmol) and tetrazole (126.0 mg, 1.800 mmol), and the mixture was stirred at room temperature for 3 h. Then *m*-CPBA (690.2 mg, 4.000 mmol) was added to the mixture, and the reaction was stirred for 0.5 h, followed by quenching with $Na_2S_2O_3$ solution. After removing the CH_3CN by evaporation, the mixture was diluted with CH_2Cl_2, washed with H_2O, saturated $NaHCO_3$ and brine. The combined organic layers were dried over Na_2SO_4 and filtered. The filtrate was concentrated, and then purified via recrystallization to get the crude product.

The above crude product and NaI (600.0 mg, 4.000 mmol) were dissolved in CH_3CN (0.2 M), and the mixture was stirred at 60°C for 3 h. Then the solvent was removed by evaporation, and the mixture was extracted with EtOAc, washed with H_2O. The combined organic layers were dried over Na_2SO_4, filtered, and concentrated. The residue was loaded to a silica gel column which was neutralized by using hexanes/Et_3N (100/1) and purified by eluting with solvents (CH_2Cl_2/MeOH = 20/1) to give the product in the form of a phosphate salt. The phosphate salt in a mixed solvent of CH_2Cl_2/MeOH was acidified by H^+ resin, then the solution was filtered and concentrated to obtain **2s** as a yellow syrup (252.0 mg, 59%). ^{1}**H NMR** (400 MHz, $CDCl_3$) δ 7.41 - 7.29 (m, 5H), 5.51 (d, *J* = 5.0 Hz, 1H), 5.07 (d, *J* = 7.1 Hz, 2H), 4.59 (dd, *J* = 7.9, 2.5 Hz, 1H), 4.30 (dd, *J* = 5.0, 2.5 Hz, 1H), 4.22 (dd, *J* = 7.9, 1.9 Hz, 1H), 4.19 - 4.13 (m, 2H), 4.06 (td, *J* = 6.4, 1.8 Hz, 1H), 1.50 (s, 3H), 1.41 (s, 3H), 1.31 (s, 3H), 1.30 (s, 3H). ^{31}**P NMR** (162 MHz, $CDCl_3$) δ 0.59. The ^{1}H NMR data coincide with the reported data[9].

Methyl 2-*O*-benzyl-4,6-*O*-benzylidene-3-*O*-(benzyloxy(hydroxy)phosphoryl)-α-D-glucopyranoside (2t)

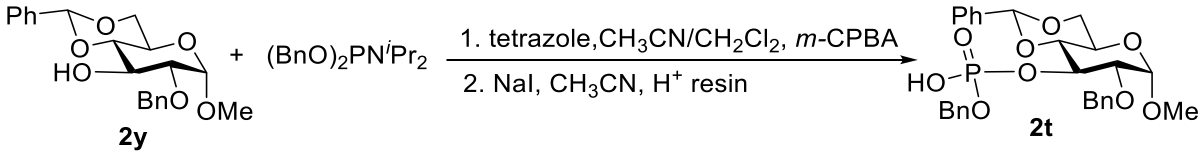

To a solution of **2y** (186.1 mg, 0.5000 mmol) in CH_3CN and CH_2Cl_2 (CH_3CN/CH_2Cl_2 = 1/4, 0.2 M) were added $(BnO)_2PN^iPr_2$ (0.25 mL, 0.75 mmol) and tetrazole (63.0 mg, 0.900 mmol), and the mixture was stirred at room temperature for 2 h. Then *m*-CPBA (345.1 mg, 2.00 mmol) was added to the mixture, and the reaction was stirred for 2 h, followed by quenching with $Na_2S_2O_3$ solution. After removing the CH_3CN by evaporation, the mixture

was diluted with CH_2Cl_2, washed with H_2O, saturated $NaHCO_3$ and brine. The combined organic layers were dried over Na_2SO_4 and filtered. The filtrate was concentrated, and then purified via recrystallization to get the crude product.

The above crude product and NaI (300.0 mg, 2.000 mmol) were dissolved in CH_3CN (0.2 M), and the mixture was stirred at 60°C for 3 h. Then the solvent was removed by evaporation, and the mixture was extracted with EtOAc, washed with H_2O. The combined organic layers were dried over Na_2SO_4, filtered and concentrated. The residue was loaded to silica gel column which was neutralized by using hexanes/Et_3N (100/1) and purified by eluting with solvents (CH_2Cl_2/MeOH = 20/1) to give the product in the form of a phosphate salt. The phosphate salt in a mixed solvent of CH_2Cl_2/MeOH was acidified by H^+ resin, then the solution was filtered and concentrated to obtain **2t** as a white solid (160.0 mg, 61%). $[\alpha]_D^{25}$: 3.08 (*c*: 0.13 $CHCl_3$). **^{1}H NMR** (400 MHz, $CDCl_3$) δ 7.47 - 7.44 (m, 2H), 7.38 - 7.26 (m, 8H), 7.23 - 7.15 (m, 3H), 7.15 - 7.06 (m, 2H), 5.47 (s, 1H), 4.96 - 4.88 (m, 2H), 4.84 - 4.77 (m, 2H), 4.58 (d, *J* = 12.4 Hz, 1H), 4.47 - 4.45 (m, 1H), 4.23 (dd, *J* = 10.1, 4.7 Hz, 1H), 3.81 - 3.75 (m, 1H), 3.74 - 3.47 (m, 3H), 3.28 (s, 3H). **^{31}P NMR** (162 MHz, $CDCl_3$) δ -0.41. **^{13}C NMR** (101 MHz, $CDCl_3$) δ 137.8, 136.8, 136.2 (d, *J* = 9.0 Hz), 128.9, 128.46, 128.40, 128.23, 128.20, 128.0, 127.8, 127.3, 126.2, 101.6, 99.1, 80.0 (d, *J* = 3.0 Hz), 78.2 (d, *J* = 3.0 Hz), 73.7, 68.8, 68.79, 68.74, 62.0, 55.3. **HRMS** (ESI) Calculated for $C_{28}H_{31}O_9PNa$ $[M+Na]^+$: 565.1603 Found 565.1607.

Methyl 3,4,6-tri-*O*-benzyl-2-*O*-(benzyloxy(hydroxy)phosphoryl)-α-D-glucopyranoside (2u)

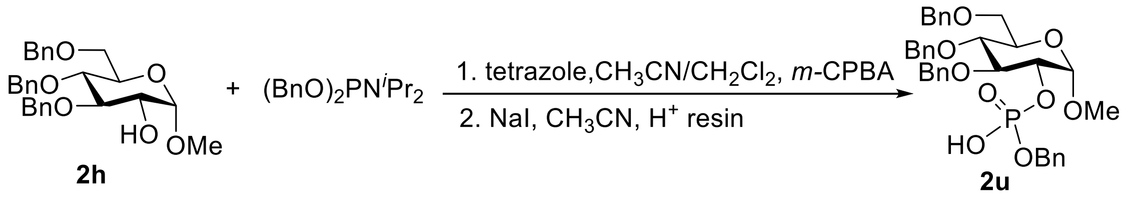

To a solution of **2h** (464.2 mg, 1.000 mmol) in CH_3CN and CH_2Cl_2 (CH_3CN/CH_2Cl_2 = 1/4, 0.2 M) were added $(BnO)_2PN^iPr_2$ (0.50 mL, 1.5 mmol) and tetrazole (126.0 mg, 1.800 mmol), and the mixture was stirred at room temperature for 2 h. Then *m*-CPBA (690.2 mg, 4.000 mmol) was added to the mixture, and the reaction was stirred for 1 h, followed by quenching with $Na_2S_2O_3$ solution. After removing the CH_3CN by evaporation, the mixture was diluted with CH_2Cl_2, washed with H_2O, saturated $NaHCO_3$ and brine. The combined organic layers were dried over Na_2SO_4 and filtered. The filtrate was concentrated, and then purified via recrystallization to get the crude product.

The above crude product and NaI (600.0 mg, 4.000 mmol) were dissolved in CH_3CN (0.2 M), and the mixture was stirred at 60°C for 3 h. Then the solvent was removed by evaporation, and the mixture was extracted with EtOAc, washed with H_2O. The combined organic layers were dried over Na_2SO_4, filtered, and concentrated. The residue was loaded to silica gel column which was neutralized by using hexanes/Et_3N (100/1) and purified by eluting with solvents (CH_2Cl_2/MeOH = 20/1) to give the product in the form of a phosphate salt. The phosphate salt in a mixed

solvent of CH_2Cl_2/MeOH was acidified by H^+ resin, then the solution was filtered and concentrated to obtain **2u** as a colorless oil (228.0 mg, 72%). $[\alpha]_D^{25}$: 44.62 (*c*: 0.13 $CHCl_3$). **^{1}H NMR** (400 MHz, $CDCl_3$) δ 7.38 - 7.21 (m, 18H), 7.13 (dd, *J* = 6.6, 2.9 Hz, 2H), 5.07 - 4.97 (m, 3H), 4.90 (d, *J* = 10.8 Hz, 1H), 4.77 (d, *J* = 10.8 Hz, 1H), 4.72 (d, *J* = 10.8 Hz, 1H), 4.59 (d, *J* = 12.1 Hz, 1H), 4.49 - 4.43 (m, 2H), 4.35 - 4.29 (m, 1H), 3.99 (t, *J* = 9.2 Hz, 1H), 3.79 - 3.75 (m, 1H), 3.72 - 3.59 (m, 3H), 3.34 (s, 3H). **^{31}P NMR** (162 MHz, $CDCl_3$) δ -0.12. **^{13}C NMR** (101 MHz, $CDCl_3$) δ 138.3, 138.1, 137.9, 135.8 (d, *J* = 9.0 Hz), 128.4, 128.38, 128.34, 128.31, 128.2, 128.1, 127.86, 127.83, 127.68, 127.62, 127.5, 97.8, 80.6 (d, *J* = 8.0 Hz), 77.5, 75.6, 75.0, 73.4, 70.1, 68.9 (d, *J* = 6.0 Hz), 68.4, 55.3. HRMS (ESI) Calculated for $C_{35}H_{39}O_9PNa$ $[M+Na]^+$: 657.2229 Found:657.2225.

Methyl 2,3,6-tri-*O*-benzyl-4-*O*-(benzyloxy(hydroxy)phosphoryl)-α-D-glucopyranoside (2v)

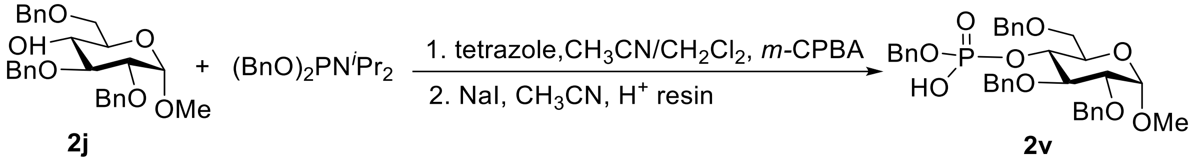

2j **2v**

To a solution of **2j** (232.1 mg, 0.5000 mmol) in CH_3CN and CH_2Cl_2 (CH_3CN/CH_2Cl_2 = 1/4, 0.2 M) were added $(BnO)_2PN^iPr_2$ (0.25 mL, 0.75 mmol) and tetrazole (63.0 mg, 0.900 mmol), and the mixture was stirred at room temperature for 3 h. Then *m*-CPBA (345.1 mg, 2.000 mmol) was added to the mixture, and the reaction was stirred for 2 h, followed by quenching with $Na_2S_2O_3$ solution. After removing the CH_3CN by evaporation, the mixture was diluted with CH_2Cl_2, washed with H_2O, saturated $NaHCO_3$ and brine. The combined organic layers were dried over Na_2SO_4 and filtered. The filtrate was concentrated, and then purified via recrystallization to get the crude product.

The above crude product and NaI (300.0 mg, 2.000 mmol) were dissolved in CH_3CN (0.2 M), and the mixture was stirred at 60°C for 3 h. Then the solvent was removed by evaporation, and the mixture was extracted with EtOAc, and washed with H_2O. The combined organic layers were dried over Na_2SO_4, filtered, and concentrated. The residue was loaded to a silica gel column which was neutralized by using hexanes/Et_3N (100/1) and purified by eluting with solvents (CH_2Cl_2/MeOH = 20/1) to give the product in the form of a phosphate salt. The phosphate salt in a mixed solvent of CH_2Cl_2/MeOH was acidified by H^+ resin, then the solution was filtered and concentrated to obtain **2v** as a yellow syrup (228.0 mg, 72%). **^{1}H NMR** (400 MHz, $CDCl_3$) δ 7.37 (d, *J* = 7.2 Hz, 2H), 7.30 - 7.13 (m, 18H), 4.98 - 4.79 (m, 4H), 4.70 (d, *J* = 12.0 Hz, 1H), 4.58 - 4.48 (m, 3H), 4.48 - 4.31 (m, 2H), 3.93 (t, *J* = 9.3 Hz, 1H), 3.82 - 3.63 (m, 3H), 3.46 (dd, *J* = 9.6, 3.6 Hz, 1H), 3.35 (s, 3H). **^{31}P NMR** (162 MHz, $CDCl_3$) δ -0.58. The ^{1}H NMR data coincide with the reported data[9].

3-*O*-(benzyloxy(hydroxy)phosphoryl)-*N*-(*t*ert-butoxycarbonyl) -L-serine methyl ester (2w)

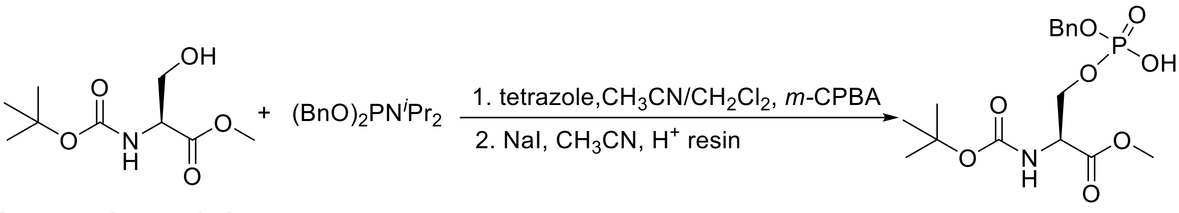

Boc-L-serine methyl ester **2w**

To a solution of Boc-L-serine methyl ester (109.6 mg, 0.5000 mmol) in CH_3CN and CH_2Cl_2 (CH_3CN/CH_2Cl_2 = 1/4, 0.2 M) were added $(BnO)_2PN^iPr_2$ (0.25 mL, 0.75 mmol) and tetrazole (63.0 mg, 0.900 mmol), and the mixture was stirred at room temperature for 2 h. Then *m*-CPBA (345.1 mg, 2.000 mmol) was added to the mixture, and the reaction was stirred for 0.5 h, followed by quenching with $Na_2S_2O_3$ solution. After removing the CH_3CN by evaporation, the mixture was diluted with CH_2Cl_2, washed with H_2O, saturated $NaHCO_3$ and brine. The combined organic layers were dried over Na_2SO_4 and filtered. The filtrate was concentrated, and then purified via recrystallization to get the crude product.

The above crude product and NaI (300.0 mg, 2.000 mmol) were dissolved in CH_3CN (0.2 M), and the mixture was stirred at 60°C for 3 h. Then the solvent was removed by evaporation, and the mixture was extracted with EtOAc, and washed with H_2O. The combined organic layers were dried over Na_2SO_4, filtered, and concentrated. The residue was loaded to a silica gel column which was neutralized by using hexanes/Et_3N (100/1) and purified by eluting with solvents (CH_2Cl_2/MeOH = 20/1) to give the product in the form of a phosphate salt. The phosphate salt in a mixed solvent of CH_2Cl_2/MeOH was acidified by H^+ resin, then the solution was filtered and concentrated to obtain **2w** as a yellow syrup (155.6 mg, 80%). $[\alpha]_D^{25}$: 2.10 (*c*: 0.19 $CHCl_3$). **^{1}H NMR** (400 MHz, $CDCl_3$) δ 7.39 - 7.31 (m, 5H), 5.03 (d, *J* = 7.8 Hz, 2H), 4.57 - 4.33 (m, 2H), 4.29 - 4.17 (m, 1H), 3.73 (s, 3H), 1.44 (s, 9H). ^{31}P NMR (162 MHz, $CDCl_3$) δ 0.39. **^{13}C NMR** (101 MHz, $CDCl_3$) δ 169.7, 155.3, 135.6 (d, *J* = 7.0 Hz), 128.57, 128.55, 127.8, 80.3, 69.3 (d, *J* = 7.0 Hz), 67.3 (d, *J* = 5.0 Hz), 53.8 (d, *J* = 8.0 Hz), 52.7, 28.2. **HRMS** (ESI) Calculated for $C_{16}H_{24}NO_8PNa$ $[M+Na]^+$: 412.1137 Found: 412.1130.

5'-*O*-(benzyloxy(hydroxy)phosphoryl)- 2',3'-*O*-isopropylideneuridine (2x)

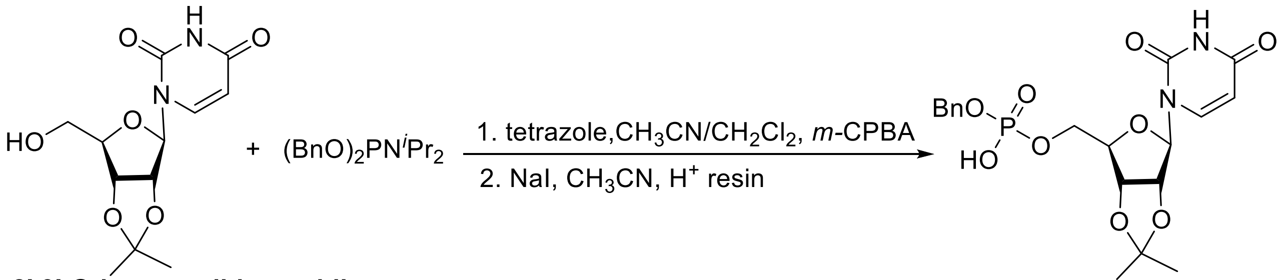

2',3'-O-isopropylideneuridine **2x**

To a solution of 2',3'-O-isopropylideneuridine (142.0 mg, 0.5000 mmol) in CH_3CN and CH_2Cl_2 (CH_3CN/CH_2Cl_2 = 1/4, 0.2 M) were added $(BnO)_2PN^iPr_2$ (0.25 mL, 0.75 mmol) and tetrazole (63.0 mg, 0.900 mmol), and the

mixture was stirred at room temperature for 4 h. Then *m*-CPBA (345.1 mg, 2.000 mmol) was added to the mixture, and the reaction was stirred for 1 h, followed by quenching with $Na_2S_2O_3$ solution. After removing the CH_3CN by evaporation, the mixture was diluted with CH_2Cl_2, washed with H_2O, saturated $NaHCO_3$ and brine. The combined organic layers were dried over Na_2SO_4 and filtered. The filtrate was concentrated, and then purified via recrystallization to get the crude product.

The above crude product and NaI (300.0 mg, 2.000 mmol) were dissolved in CH_3CN (0.2 M), and the mixture was stirred at 60°C for 3 h. Then the solvent was removed by evaporation to obtain the residue. The residue was loaded to a silica gel column which was neutralized by using hexanes/Et_3N (100/1) and purified by eluting with solvents (CH_2Cl_2/MeOH = 15/1) to give the product in the form of a phosphate salt. The phosphate salt in a mixed solvent of CH_2Cl_2/MeOH was acidified by H^+ resin, then the solution was filtered and concentrated to obtain **2x** as a yellow solid (147.6 mg, 65%). $[\alpha]_D^{25}$: 1.25 (*c*: 0.16 $CHCl_3$). **^{1}H NMR** (400 MHz, CD_3OD) δ 7.68 (d, *J* = 8.1 Hz, 1H), 7.42 - 7.32 (m, 5H), 5.85 (d, *J* = 2.6 Hz, 1H), 5.67 (d, *J* = 8.0 Hz, 1H), 5.03 (d, *J* = 7.7 Hz, 2H), 4.91 (dd, *J* = 6.4, 2.6 Hz, 1H), 4.82 (dd, *J* = 6.3, 3.4 Hz, 1H), 4.34 - 4.29 (m, 1H), 4.20 - 4.11 (m, 2H), 1.55 (s, 3H), 1.35 (s, 3H). **^{31}P NMR** (162 MHz, CD_3OD) δ -0.81. **^{13}C NMR** (101 MHz, CD_3OD) δ 164.7, 150.5, 142.4, 136.7 (d, *J* = 7.0 Hz), 128.1, 127.9, 127.3, 113.8, 101.5, 93.1, 85.3 (d, *J* = 8.0 Hz), 84.2, 80.8, 68.2 (d, *J* = 5.0 Hz), 66.2 (d, *J* = 5.0 Hz), 26.0, 24.1. **HRMS** (ESI) Calculated for $C_{19}H_{23}N_2O_9PNa$ $[M+Na]^+$: 477.1039 Found: 477.1034.

5. Glycosylation reactions using 2-glycosyloxy tropone donors

1,2:3,4-Di-*O*-isopropylidene-6-*O*-(tetrahydro-2*H*-pyran-2-yl)-α-D-galactopyranoside (3a)

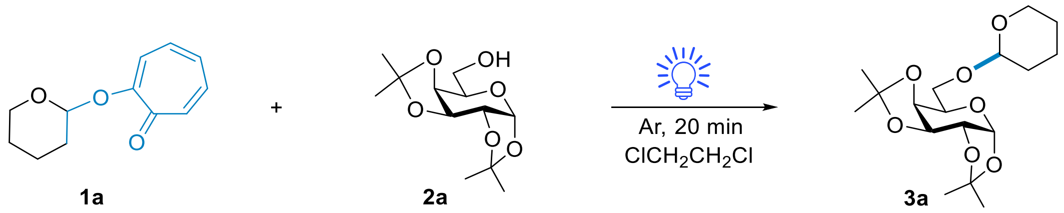

The mixture of **1a** (15.5 mg, 0.0750 mmol), acceptor **2a** (13.0 mg, 0.0500 mmol) and NaOTf (0.9 mg, 0.005 mmol) in dry $ClCH_2CH_2Cl$ (2.0 mL), was irradiated by blue LEDs at ambient temperature for 20 min. Then triethylamine (0.1 mL) was added to the reaction, the solvent was concentrated under reduced pressure. The resulting residue was eluted by flash column chromatography (petroleum ether/EtOAc = 3/1) to afford the glycosylated product **3a** as colorless oil (15.6 mg, 91%). **^{1}H NMR** (400 MHz, $CDCl_3$) δ 5.55 (dd, *J* = 9.3, 5.0 Hz, 1H), 4.73 - 4.56 (m, 2H), 4.39 - 4.21 (m, 2H), 4.05 - 3.99 (m, 1H), 3.96 - 3.59 (m, 3H), 3.56 - 3.43 (m, 1H), 1.88 - 1.59 (m, 4H), 1.55 (s, 3H), 1.51 (dd, *J* = 10.0, 4.6 Hz, 2H), 1.45 (s, 3H), 1.33 (s, 6H). **^{13}C NMR** (101 MHz, $CDCl_3$) δ 109.25, 109.20, 108.5, 99.1, 99.0, 96.4, 96.3, 71.4, 71.0, 70.73, 70.71, 70.6, 70.5, 67.4, 66.5, 66.1, 65.9, 62.4, 62.2, 30.5, 26.04, 26.00, 25.49, 25.41, 24.9, 24.5, 24.4, 19.5, 19.4. The ^{1}H NMR data coincide with the reported data[10].

Methyl 2,3,4-tri-*O*-benzyl-6-*O*-(2,3,4,6-tetra-*O*-benzyl-α/β-D-glucopyranosyl)-α-D-glucopyranoside (3b)

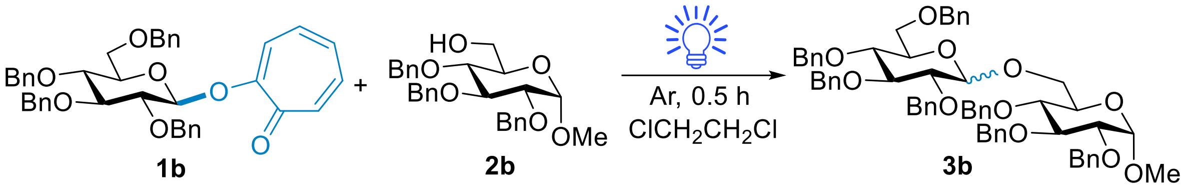

The glycosyl donor **1b** (48.3 mg, 0.0750 mmol), acceptor **2b** (23.2 mg, 0.0500 mmol) and TMSOTf (0.90 uL, 0.0050 mmol) were dissolved in dry $ClCH_2CH_2Cl$ (2.0 mL), and the mixture was irradiated by blue LEDs at ambient temperature for 0.5 h. Upon completion, the solvent was concentrated under reduced pressure. The resulting residue was eluted by flash column chromatography (petroleum ether/EtOAc = 8/1) to afford the glycosylated product **3b** as a colorless oil (45.3 mg, 92%, α/β =2.0:1). **^{1}H NMR** (400 MHz, $CDCl_3$) δ 7.38 - 7.08 (m, 65H), 4.97 - 4.89 (m, 6.6H), 4.86 - 4.48 (m, 20H), 4.48 - 4.32 (m, 3H), 4.18 (d, *J* = 10.6 Hz, 0.6H), 4.01 - 3.93 (m, 2.7H), 3.86 - 3.42 (m, 19H), 3.35 (s, 3H), 3.32 (s, 1.7H). The ^{1}H NMR data coincide with the reported data[11].

Docosyl 2,3,4,6-tetra-*O*-benzoyl-β-D-galactopyranoside (3c)

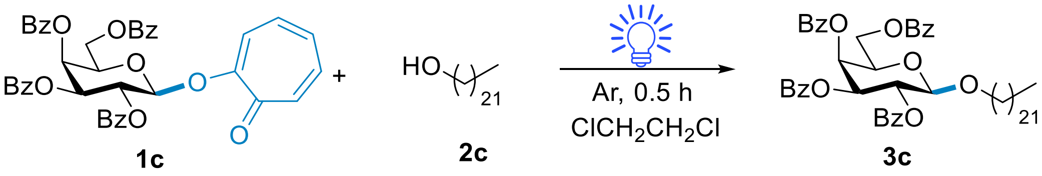

The glycosyl donor **1c** (52.5 mg, 0.0750 mmol), acceptor **2c** (16.3 mg, 0.0500 mmol) and TMSOTf (0.90 uL, 0.0050 mmol) were dissolved in dry $ClCH_2CH_2Cl$ (2.0 mL). The mixture was irradiated by blue LEDs at ambient temperature for 0.5 h. Upon completion, the solvent was concentrated under reduced pressure. The resulting residue was eluted by flash column chromatography (petroleum ether/EtOAc = 8/1) to afford the glycosylated product **3c** as a pale-yellow solid (42.9 mg, 95%). $[\alpha]_D^{25}$: 68.57 (*c*: 0.14 $CHCl_3$). **^{1}H NMR** (400 MHz, $CDCl_3$) δ 8.13 - 8.07 (m, 2H), 8.05 - 7.99 (m, 2H), 7.99 - 7.94 (m, 2H), 7.84 - 7.75 (m, 2H), 7.64 - 7.35 (m, 10H), 7.23 (d, *J* = 7.8 Hz, 2H), 5.99 (d, *J* = 3.4 Hz, 1H), 5.79 (dd, *J* = 10.4, 7.9 Hz, 1H), 5.60 (dd, *J* = 10.4, 3.5 Hz, 1H), 4.81 (d, *J* = 8.0 Hz, 1H), 4.69 (dd, *J* = 11.2, 6.5 Hz, 1H), 4.42 (dd, *J* = 11.2, 6.8 Hz, 1H), 4.32 (t, *J* = 6.6 Hz, 1H), 4.00 - 3.94 (m, 1H), 3.59 - 3.53 (m, 1H), 1.60 - 1.55 (m, 2H), 1.56 - 1.46 (m, 2H), 1.31 - 1.26 (m, 16H), 1.25 - 0.98 (m, 20H), 0.88 (t, *J* = 6.7 Hz, 3H). **^{13}C NMR** (101 MHz, $CDCl_3$) δ 166.0, 165.6, 165.5, 165.2, 133.5, 133.26, 133.24, 133.1, 130.0, 129.79, 129.78, 129.70, 129.47, 129.45, 129.0, 128.8, 128.5, 128.4, 128.3, 128.2, 101.7, 71.7, 71.2, 70.6, 69.8, 68.1, 62.0, 31.9, 29.72, 29.71, 29.68, 29.66, 29.5, 29.48, 29.43, 29.36, 29.30, 25.8, 22.7, 14.1. **HRMS** (ESI) Calculated for $C_{56}H_{76}NO_{10}$ $[M+NH_4]^+$: 922.5469 Found: 922.5481.

Benzyl *N*-(benzyloxycarbonyl)-*O*-(2,3,4,6-tetra-*O*-benzoyl-β-D-galactopyranosyl)-L-serinate (3d)

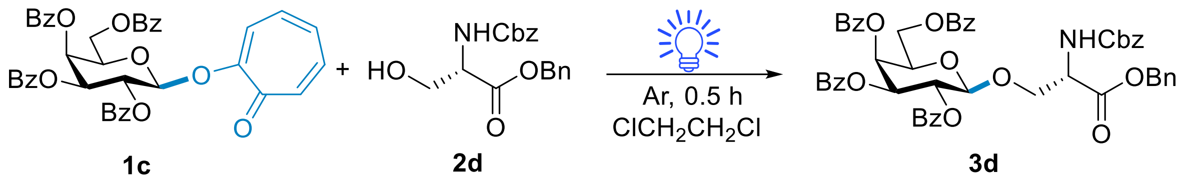

The glycosyl donor **1c** (52.5 mg, 0.0750 mmol), acceptor **2d** (16.5 mg, 0.0500 mmol) and TMSOTf (0.90 uL, 0.0050 mmol) were dissolved in dry $ClCH_2CH_2Cl$ (2.0 mL), and the mixture was irradiated by blue LEDs at ambient temperature for 0.5 h. Upon completion, the solvent was concentrated under reduced pressure. The resulting residue was eluted by flash column chromatography (petroleum ether/EtOAc = 8/1) to afford the glycosylated product **3d** as a pale-yellow solid (43.9 mg, 95%). **^{1}H NMR** (400 MHz,$CDCl_3$) δ 8.10 - 8.04 (m, 2H), 8.04 - 7.97 (m, 2H), 7.92 (d, *J* = 7.7 Hz, 2H), 7.81 - 7.74 (m, 2H), 7.65 - 7.53 (m, 2H), 7.51 - 7.39 (m, 6H), 7.39 - 7.26 (m, 12H), 7.23 (d, *J* = 7.7 Hz, 2H), 5.96 (dd, *J* = 3.4, 1.2 Hz, 1H), 5.73 (dd, *J* = 10.4, 7.9 Hz, 1H), 5.63 - 5.49 (m, 2H), 5.21 - 5.11 (m, 2H), 5.09 - 4.91 (m, 2H), 4.77 (d, *J* = 7.9 Hz, 1H), 4.60 (dd, *J* = 11.3, 6.5 Hz, 1H), 4.54 - 4.52 (m, 1H), 4.48 - 4.35 (m, 2H), 4.27 - 4.17 (m, 1H), 3.97 (dd, *J* = 10.4, 3.5 Hz, 1H). The ^{1}H NMR data coincide with the reported data[12].

Menthyl 2,3,4,6-tetra-*O*-benzoyl-β-D-galactopyranoside (3e)

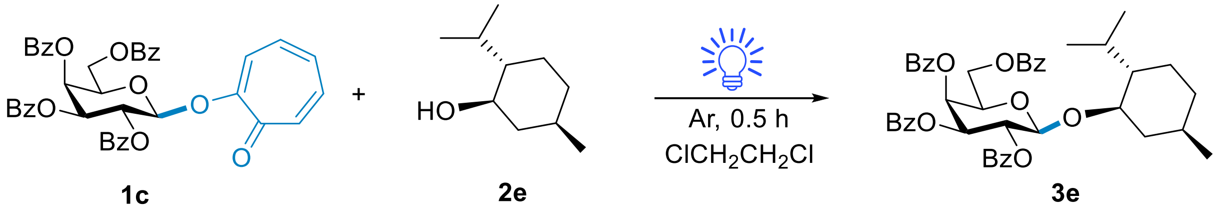

The glycosyl donor **1c** (52.5 mg, 0.0750 mmol), acceptor **2e** (7.8 mg, 0.050 mmol) and TMSOTf (0.90 uL, 0.0050 mmol) were dissolved in dry $ClCH_2CH_2Cl$ (2.0 mL), and the mixture was irradiated by blue LEDs at ambient temperature for 0.5 h. Upon completion, the solvent was concentrated under reduced pressure. The resulting residue was eluted by flash column chromatography (petroleum ether/EtOAc = 8/1) to afford the glycosylated product **3e** as a pale-yellow solid (36.3 mg, 99%). **^{1}H NMR** (400 MHz, $CDCl_3$) δ 8.12 - 8.07 (m, 2H), 8.05 - 8.00 (m, 2H), 8.00 - 7.94 (m, 2H), 7.85 - 7.76 (m, 2H), 7.64 -7.58 (m, 1H), 7.58 - 7.34 (m, 9H), 7.26 - 7.21 (m, 2H), 5.98 (dd, *J* = 3.5, 1.1 Hz, 1H), 5.74 (dd, *J* = 10.4, 7.9 Hz, 1H), 5.58 (dd, *J* = 10.4, 3.4 Hz, 1H), 4.87 (d, *J* = 7.9 Hz, 1H), 4.62 (dd, *J* = 11.3, 6.7 Hz, 1H), 4.42 (dd, *J* = 11.3, 6.3 Hz, 1H), 4.30 - 4.27 (m, 1H), 3.48 (td, *J* = 10.7, 4.3 Hz, 1H), 2.39 - 2.32 (m, 1H), 1.97 - 1.91 (m, 1H), 1.62 - 1.54 (m, 2H), 1.32 - 1.18 (m, 2H), 0.97 -0.89 (m, 1H),

0.87 (d, *J* = 7.1 Hz, 3H), 0.76 (d, *J* = 6.8 Hz, 3H), 0.73 (d, *J* = 6.5 Hz, 3H), 0.72 - 0.65 (m, 2H). The ^{1}H NMR data coincide with the reported data[11].

(*S*)-2-(1-*O*-(2,3,4,6-tetra-*O*-benzoyl-β-D-galactopyranosyloxy) ethyl)naphthalene (3f)

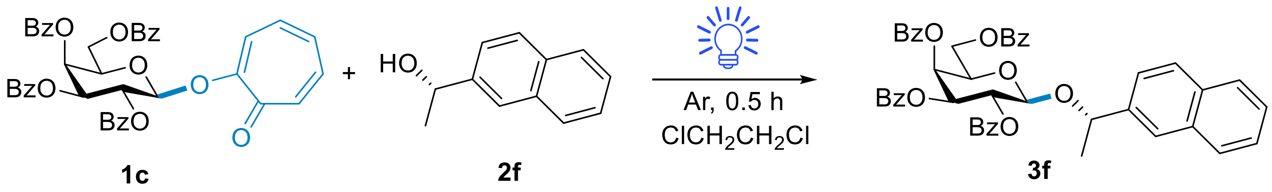

The glycosyl donor **1c** (52.5 mg, 0.0750 mmol), acceptor **2f** (8.6 mg, 0.050 mmol) and TMSOTf (0.90 uL, 0.0050 mmol) were dissolved in dry $ClCH_2CH_2Cl$ (2.0 mL), and the mixture was irradiated by blue LEDs at ambient temperature for 0.5 h. Upon completion, the solvent was concentrated under reduced pressure. The resulting residue was eluted by flash column chromatography (petroleum ether/EtOAc = 8/1) to afford the glycosylated product **3f** as a pale-yellow solid (33.8 mg, 90%). $[\alpha]_D^{25}$: 111.67 (*c*: 0.18 $CHCl_3$). **^{1}H NMR** (400 MHz, $CDCl_3$) δ 8.13 - 8.06 (m, 4H), 7.90 (d, *J* = 7.8 Hz, 2H), 7.76 (t, *J* = 7.7 Hz, 3H), 7.65 - 7.35 (m, 14H), 7.28 - 7.18 (m, 4H), 5.92 (d, *J* = 3.4 Hz, 1H), 5.87 (dd, *J* = 10.3, 8.0 Hz, 1H), 5.38 (dd, *J* = 10.3, 3.5 Hz, 1H), 5.14 (q, *J* = 6.5 Hz, 1H), 4.73 (dd, *J* = 11.4, 6.8 Hz, 1H), 4.65 (d, *J* = 8.0 Hz, 1H), 4.45 (dd, *J* = 11.3, 6.4 Hz, 1H), 4.17 (t, *J* = 6.6 Hz, 1H), 1.57 (d, *J* = 6.5 Hz, 3H). **^{13}C NMR** (101 MHz, $CDCl_3$) δ 166.0, 165.6, 165.4, 165.1, 139.2, 133.5, 133.3, 133.2, 133.17, 133.13, 133.0, 130.0, 129.9, 129.8, 129.7, 129.5, 129.4, 129.0, 128.7, 128.59, 128.53, 128.51, 128.3, 128.2, 127.8, 127.6, 126.1, 126.0, 125.7, 124.2, 98.5, 76.2, 71.8, 71.3, 69.7, 68.1, 62.1, 24.0. **HRMS** (ESI) Calculated for $C_{46}H_{42}NO_{10}$ $[M+NH_4]^+$: 768.2809 Found: 768.2806.

1-Adamantyl 2,3,4,6-tetra-*O*-benzoyl-β-D-galactopyranoside (3g)

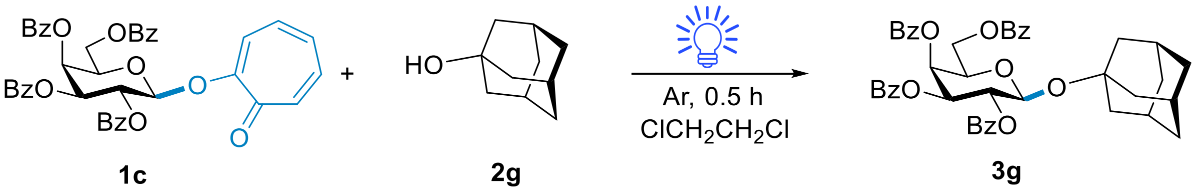

The glycosyl donor **1c** (52.5 mg, 0.0750 mmol), acceptor **2g** (7.6 mg, 0.050 mmol) and TMSOTf (0.90 uL, 0.0050 mmol) were dissolved in dry $ClCH_2CH_2Cl$ (2.0 mL), and the mixture was irradiated by blue LEDs at ambient temperature for 0.5 h. Upon completion, the solvent was concentrated under reduced pressure. The resulting residue was eluted by flash column chromatography (petroleum ether/EtOAc = 8/1) to afford the glycosylated product **3g** as a white solid (35.8 mg, 98%). **^{1}H NMR** (400 MHz, $CDCl_3$) δ 8.15 - 8.08 (m, 2H), 8.06 - 8.01 (m, 2H), 7.99 - 7.93 (m, 2H), 7.82 - 7.75 (m, 2H), 7.64 - 7.53 (m, 2H), 7.52 - 7.35 (m, 8H), 7.26 - 7.20 (m, 2H), 5.95 (dd, *J* = 3.6, 1.2 Hz, 1H), 5.77 (dd, *J* = 10.4, 8.0 Hz, 1H), 5.60 (dd, *J* = 10.3, 3.6 Hz, 1H), 5.09 (d, *J* = 7.9 Hz, 1H), 4.60 (dd, *J* = 11.4, 7.7 Hz, 1H), 4.46 (dd, *J* = 11.4, 5.5 Hz, 1H), 4.33 - 4.30 (m, 1H), 2.04 (br, 3H), 1.90 - 1.81 (m,

3H), 1.70 - 1.67 (m, 3H), 1.58 - 1.55 (m, 3H), 1.51 - 1.48 (m, 3H). The ^{1}H NMR data coincide with the reported data[13].

Methyl 2,3,4-tri-*O*-benzyl-6-*O*-(2,3,4,6-tetra-*O*-benzoyl-β-D-galactopyranosyl)-α-D-glucopyranoside (3h)

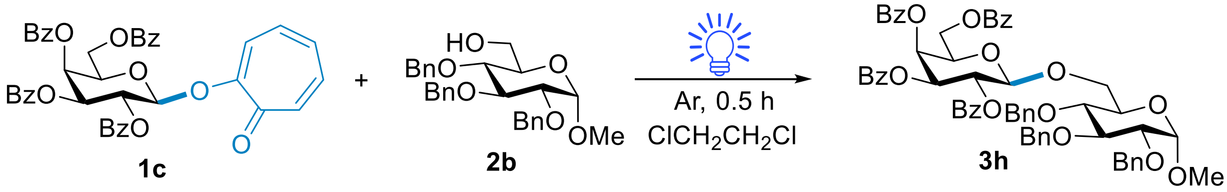

The glycosyl donor **1c** (52.5 mg, 0.0750 mmol), acceptor **2b** (23.2 mg, 0.0500 mmol) and TMSOTf (0.90 uL, 0.0050 mmol) were dissolved in dry $ClCH_2CH_2Cl$ (2.0 mL), and the mixture was irradiated by blue LEDs at ambient temperature for 0.5 h. Upon completion, the solvent was concentrated under reduced pressure. The resulting residue was eluted by flash column chromatography (petroleum ether/EtOAc = 6/1) to afford the glycosylated product **3h** as a pale-yellow oil (50.0 mg, 96%). **^{1}H NMR** (400 MHz,$CDCl_3$) δ 8.13 - 7.99 (m, 4H), 7.93 - 7.85 (m, 2H), 7.82 - 7.72 (m, 2H), 7.64 - 7.36 (m, 9H), 7.32 - 7.25 (m, 11H), 7.24 - 7.17 (m, 5H), 7.17 - 7.09 (m, 2H), 5.97 (d, *J* = 3.5 Hz, 1H), 5.92 - 5.79 (m, 1H), 5.59 (dd, *J* = 10.4, 3.5 Hz, 1H), 4.90 (d, *J* = 10.9 Hz, 1H), 4.83 - 4.63 (m, 4H), 4.61 - 4.54 (m, 2H), 4.50 (d, *J* = 3.5 Hz, 1H), 4.45 - 4.33 (m, 2H), 4.30 - 4.16 (m, 2H), 3.90 (t, *J* = 9.2 Hz, 1H), 3.82 - 3.71 (m, 2H), 3.45 - 3.31 (m, 2H), 3.20 (s, 3H). The ^{1}H NMR data coincide with the reported data[14].

1,2:3,4-Di-*O*-isopropylidene-6-*O*-(2,3,4,6-tetra-*O*-benzoyl-β-D-galactopyranosyl)-α-D-galactopyranoside (3i)

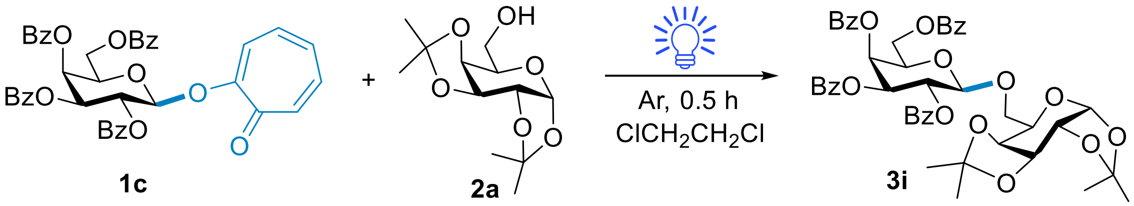

The glycosyl donor **1c** (52.5 mg, 0.0750 mmol), acceptor **2a** (13.0 mg, 0.0500 mmol) and TMSOTf (0.90 uL, 0.0050 mmol) were dissolved in dry $ClCH_2CH_2Cl$ (2.0 mL), and the mixture was irradiated by blue LEDs at ambient temperature for 0.5 h. Upon completion, the solvent was concentrated under reduced pressure. The resulting residue was eluted by flash column chromatography (petroleum ether/EtOAc = 6/1) to afford the glycosylated product **3i** as a pale-yellow oil (39.4 mg, 94%). **^{1}H NMR** (400 MHz, $CDCl_3$) δ 8.10 - 8.05 (m, 2H), 8.06 - 8.01 (m, 2H), 7.99 - 7.94 (m, 2H), 7.80 - 7.76 (m, 2H), 7.64 - 7.53 (m, 2H), 7.50 - 7.33 (m, 8H), 7.24 (t, *J* = 7.7 Hz, 2H), 5.99 (d, *J* = 3.5 Hz, 1H), 5.80 (dd, *J* = 10.4, 7.9 Hz, 1H), 5.60 (dd, *J* = 10.4, 3.5 Hz, 1H), 5.41 (d, *J* =

5.0 Hz, 1H), 5.01 (d, *J* = 8.0 Hz, 1H), 4.67 (dd, *J* = 11.3, 6.6 Hz, 1H), 4.48 - 4.38 (m, 2H), 4.34 (t, *J* = 6.6 Hz, 1H), 4.21 (dd, *J* = 5.0, 2.4 Hz, 1H), 4.15 - 4.02 (m, 2H), 3.94 - 3.87 (m, 2H), 1.39 (s, 3H), 1.24 (s, 3H), 1.22 (s, 3H), 1.20 (s, 3H). The ^{1}H NMR data coincide with the reported data[15].

Methyl 3,4,6-tri-*O*-benzyl-2-*O*-(2,3,4,6-tetra-*O*-benzoyl-β-D-galactopyranosyl)-α-D-glucopyranoside (3j)

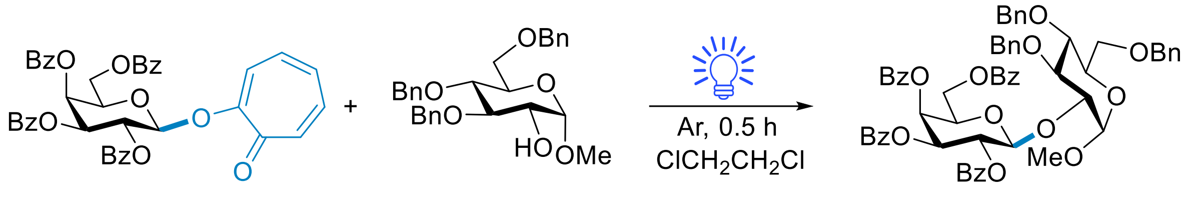

1c **2h** **3j**

The glycosyl donor **1c** (52.5 mg, 0.0750 mmol), acceptor **2h** (23.2 mg, 0.0500 mmol) and TMSOTf (0.90 uL, 0.0050 mmol) were dissolved in dry $ClCH_2CH_2Cl$ (2.0 mL), and the mixture was irradiated by blue LEDs at ambient temperature for 0.5 h. Upon completion, the solvent was concentrated under reduced pressure. The resulting residue was eluted by flash column chromatography (petroleum ether/EtOAc = 7/1) to afford the glycosylated product **3j** as a pale-yellow oil (47.4 mg, 91%). **^{1}H NMR** (400 MHz, $CDCl_3$) δ 8.14 - 8.07 (m, 2H), 8.05 - 7.99 (m, 2H), 7.75 - 7.72 (m, 4H), 7.65 - 7.59 (m, 1H), 7.55 - 7.45 (m, 3H), 7.44 - 7.27 (m, 9H), 7.25 - 7.17 (m, 5H), 7.16 - 7.07 (m, 5H), 6.99 - 6.95 (m, 4H), 6.04 - 5.94 (m, 2H), 5.58 (dd, *J* = 10.3, 3.5 Hz, 1H), 5.15 (d, *J* = 8.0 Hz, 1H), 5.08 (d, *J* = 3.4 Hz, 1H), 4.68 - 4.56 (m, 4H), 4.54 - 4.43 (m, 3H), 4.41 - 4.31 (m, 2H), 3.97 (t, *J* = 9.2 Hz, 1H), 3.84 (dd, *J* = 9.7, 3.4 Hz, 1H), 3.81 - 3.58 (m, 4H), 3.43 (s, 3H). The ^{1}H NMR data coincide with the reported data[15].

Methyl 2,4,6-tri-*O*-benzyl-3-*O*-(2,3,4,6-tetra-*O*-benzoyl-β-D-galactopyranosyl)-α- -glucopyranoside (3k)

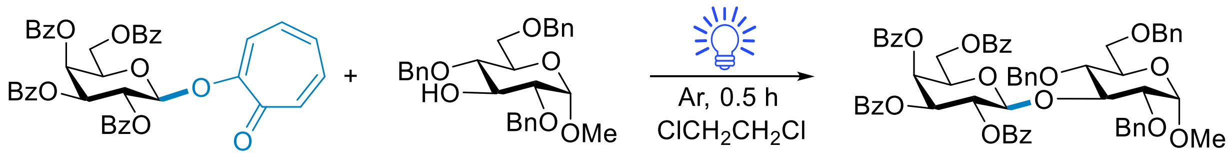

1c **2i** **3k**

The glycosyl donor **1c** (52.5 mg, 0.0750 mmol), acceptor **2i** (23.2 mg, 0.0500 mmol) and TMSOTf (0.90 uL, 0.0050 mmol) were dissolved in dry $ClCH_2CH_2Cl$ (2.0 mL), and the mixture was irradiated by blue LEDs at ambient temperature for 0.5 h. Upon completion, the solvent was concentrated under reduced pressure. The resulting residue was eluted by flash column chromatography (petroleum ether/EtOAc = 7/1) to afford the

glycosylated product **3k** as a pale-yellow oil (46.3 mg, 89%). **^{1}H NMR** (400 MHz, $CDCl_3$) δ 8.09 - 7.71 (m, 8H), 7.58 - 7.26 (m, 18H), 7.26 - 7.15 (m, 7H), 7.09 - 7.07 (m, 2H), 5.99 (dd, *J* = 3.6, 1.0 Hz, 1H), 5.86 (dd, *J* = 10.5, 8.0 Hz, 1H), 5.67 (dd, *J* = 10.4, 3.5 Hz, 1H), 5.49 (d, *J* = 8.0 Hz, 1H), 5.28 (d, *J* = 10.5 Hz, 1H), 4.69 - 4.26 (m, 9H), 4.17 (d, *J* = 12.3 Hz, 1H), 3.77 - 3.51 (m, 4H), 3.36 (dd, *J* = 9.7, 3.5 Hz, 1H), 3.25 (s, 3H). The ^{1}H NMR data coincide with the reported data[15].

Methyl 2,3,6-tri-*O*-benzyl-4-*O*-(2,3,4,6-tetra-*O*-benzoyl-β-D-galactopyranosyl)-α-D-glucopyranoside (3l)

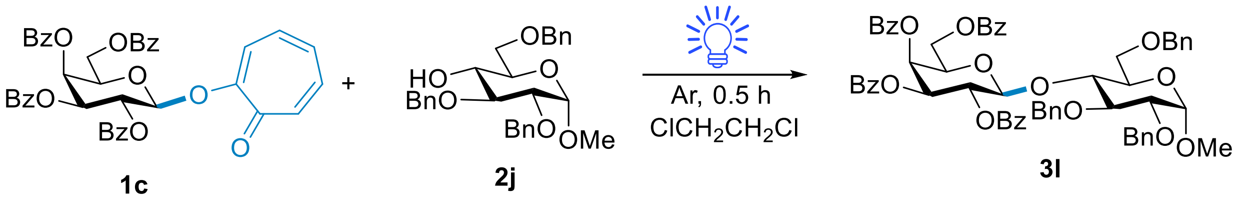

The glycosyl donor **1c** (52.5 mg, 0.0750 mmol), acceptor **2j** (23.2 mg, 0.0500 mmol) and TMSOTf (0.90 uL, 0.0050 mmol) were dissolved in dry $ClCH_2CH_2Cl$ (2.0 mL), and the mixture was irradiated by blue LEDs at ambient temperature for 1 h. Upon completion, the solvent was concentrated under reduced pressure. The resulting residue was eluted by flash column chromatography (petroleum ether/EtOAc = 6/1) to afford the glycosylated product **3l** as a pale-yellow oil (45.8 mg, 88%). **^{1}H NMR** (400 MHz,$CDCl_3$) δ 8.11 - 7.71 (m, 8H), 7.59 - 7.26 (m, 22H), 7.25 - 7.13 (m, 5H), 5.84 (dd, *J* = 3.4, 1.2 Hz, 1H), 5.79 - 5.61 (m, 1H), 5.31 - 5.27 (m, 1H), 5.17 (d, *J* = 11.1 Hz, 1H), 4.90 (d, *J* = 11.1 Hz, 1H), 4.81 - 4.71 (m, 3H), 4.64 (d, *J* = 12.3 Hz, 1H), 4.57 (d, *J* = 3.7 Hz, 1H), 4.39 (dd, *J* = 11.2, 6.2 Hz, 1H), 4.31 (d, *J* = 12.2 Hz, 1H), 4.18 (dd, *J* = 11.2, 7.5 Hz, 1H), 4.02 (t, J = 9.4 Hz, 1H), 3.95 - 3.86 (m, 2H), 3.69 (dd, *J* = 10.8, 2.9 Hz, 1H), 3.61 - 3.37 (m, 3H), 3.30 (s, 3H). The ^{1}H NMR data coincide with the reported data[15].

p-Tolyl 2,3-di-*O*-benzoyl-4-*O*-benzyl-6-*O*-(2,3,4,6-tetra-*O*-benzoyl-β-D-galactopyranosyl)-β-D-thioglucopyranoside (3m)

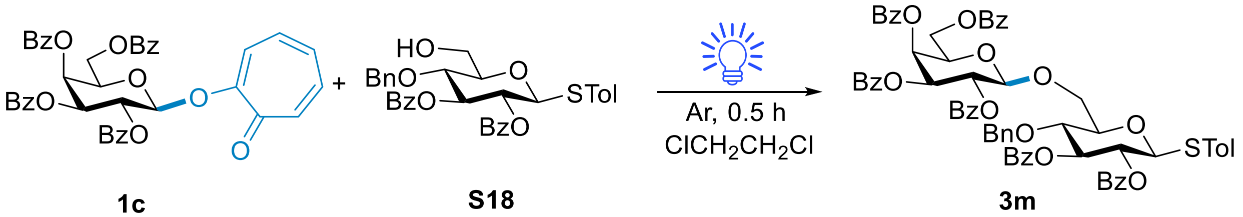

The glycosyl donor **1c** (52.5 mg, 0.0750 mmol), acceptor **S18** (29.2 mg, 0.0500 mmol) and TMSOTf (0.90 uL, 0.0050 mmol) were dissolved in dry $ClCH_2CH_2Cl$ (2.0 mL), and the mixture was irradiated by blue LEDs at

ambient temperature for 0.5 h. Upon completion, the solvent was concentrated under reduced pressure. The resulting residue was eluted by flash column chromatography (petroleum ether/EtOAc = 6/1) to afford the glycosylated product **3m** as a white solid (50.2 mg, 98%). $[\alpha]_D^{25}$: 55.00 (*c*: 0.16 $CHCl_3$). **^{1}H NMR** (400 MHz, $CDCl_3$) δ 8.18 - 8.09 (m, 2H), 8.08 - 7.99 (m, 2H), 7.93 - 7.88 (m, 4H), 7.84 - 7.79 (m, 4H), 7.74 - 7.26 (m, 21H), 7.14 - 7.10 (m, 4H), 6.96 - 6.94 (m, 2H), 6.00 (dd, *J* = 3.5, 1.2 Hz, 1H), 5.88 (dd, *J* = 10.4, 7.9 Hz, 1H), 5.67 - 5.55 (m, 2H), 5.25 (t, *J* = 9.7 Hz, 1H), 4.95 (d, *J* = 8.0 Hz, 1H), 4.78 (d, *J* = 9.9 Hz, 1H), 4.70 (dd, *J* = 11.3, 6.5 Hz, 1H), 4.45 (dd, *J* = 11.3, 6.7 Hz, 1H), 4.35 - 4.23 (m, 4H), 4.00 - 3.87 (m, 1H), 3.76 - 3.65 (m, 2H), 2.27 (s, 3H). **^{13}C NMR** (101 MHz, $CDCl_3$) δ 166.0, 165.6, 165.57, 165.54, 165.18, 165.14, 138.5, 137.0, 133.6, 133.5, 133.2, 133.19, 133.12, 130.0, 129.8, 129.79, 129.70, 129.4, 129.35, 129.31, 129.0, 128.7, 128.6, 128.5, 128.4, 128.3, 128.28, 128.24, 127.85, 127.82, 101.4, 86.2, 79.1, 76.4, 75.8, 74.7, 71.7, 71.4, 70.6, 69.7, 68.1, 67.7, 61.9, 21.1. **HRMS** (ESI) Calculated for $C_{68}H_{64}NO_{15}S$ $[M+NH_4]^+$: 1166.3997 Found: 1166.4017

p-Methoxyphenyl 2,3,4,6-tetra-*O*-benzoyl-β-D-galactopyranoside (3n)

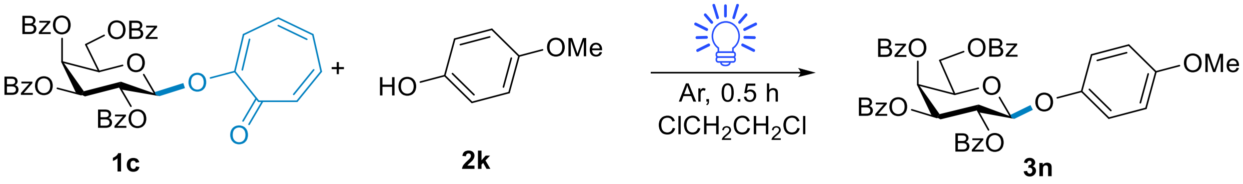

The glycosyl donor **1c** (52.5 mg, 0.0750 mmol), acceptor **2k** (6.2 mg, 0.050 mmol) and TMSOTf (0.90 uL, 0.0050 mmol) were dissolved in dry $ClCH_2CH_2Cl$ (2.0 mL), and the mixture was irradiated by blue LEDs at ambient temperature for 0.5 h. Upon completion, the solvent was concentrated under reduced pressure. The resulting residue was eluted by flash column chromatography (petroleum ether/EtOAc = 7/1) to afford the glycosylated product **3n** as a white solid (32.3 mg, 92%). **^{1}H NMR** (400 MHz,$CDCl_3$) δ 8.13 - 8.10 (m, 2H), 8.06 - 8.03 (m, 2H), 7.99 - 7.95 (m, 2H), 7.83 - 7.80 (m, 2H), 7.68 - 7.32 (m, 10H), 7.28 - 7.23 (m, 2H), 7.03 - 6.94 (m, 2H), 6.73 - 6.65 (m, 2H), 6.11 - 5.99 (m, 2H), 5.67 (dd, *J* = 10.4, 3.5 Hz, 1H), 5.26 (d, *J* = 8.0 Hz, 1H), 4.69 (dd, *J* = 11.4, 7.5 Hz, 1H), 4.54 (dd, *J* = 11.4, 5.5 Hz, 1H), 4.49 - 4.40 (m, 1H), 3.72 (s, 3H). The ^{1}H NMR data coincide with the reported data[16].

4-*O*-(2,3,4,6-Tetra-*O*-benzoyl-β-D-galactopyranosyl)-2*H*-chromen-2-one (3o)

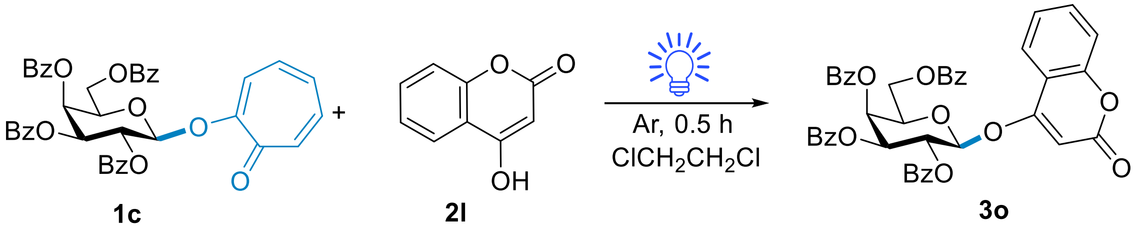

The glycosyl donor **1c** (52.5 mg, 0.0750 mmol), acceptor **2l** (8.1 mg, 0.050 mmol) and TMSOTf (0.90 uL, 0.0050 mmol) were dissolved in dry $ClCH_2CH_2Cl$ (2.0 mL), and the mixture was irradiated by blue LEDs at ambient temperature for 0.5 h. Upon completion, the solvent was concentrated under reduced pressure. The resulting residue was eluted by flash column chromatography (petroleum ether/EtOAc = 7/1) to afford the glycosylated product **3o** as a white solid (34.4 mg, 93%). $[\alpha]_D^{25}$: 78.29 (*c*: 0.35 $CHCl_3$). **^{1}H NMR** (400 MHz, $CDCl_3$) δ 8.12 (d, *J* = 7.8 Hz, 2H), 8.10 - 8.01 (m, 2H), 7.96 (d, *J* = 7.7 Hz, 2H), 7.86 - 7.76 (m, 3H), 7.70 - 7.41 (m, 9H), 7.35 (t, *J* = 7.7 Hz, 2H), 7.31 - 7.27 (m, 2H), 7.25 - 7.21 (m, 2H), 6.20 (dd, *J* = 10.4, 7.8 Hz, 1H), 6.09 - 6.08 (m, 2H), 5.85 (dd, *J* = 10.4, 3.5 Hz, 1H), 5.64 (d, *J* = 7.9 Hz, 1H), 4.62 - 4.55 (m, 3H). **^{13}C NMR** (101 MHz, $CDCl_3$) δ 166.0, 165.4, 165.3, 163.5, 162.0, 153.4, 133.8, 133.6, 133.55, 133.51, 132.7, 130.0, 129.8, 129.7, 129.0, 128.7, 128.68, 128.66, 128.5, 128.49, 128.41, 124.2, 123.2, 116.6, 114.9, 98.3, 94.1, 72.8, 70.9, 68.9, 67.7, 62.3. **HRMS** (ESI) Calculated for $C_{43}H_{33}O_{12}$ $[M+H]^+$: 741.1972 Found: 741.1976.

2-Methoxy-6-*O*-(2,3,4,6-tetra-*O*-benzoyl-β-D-galactopyranosyl)naphthalene (3p)

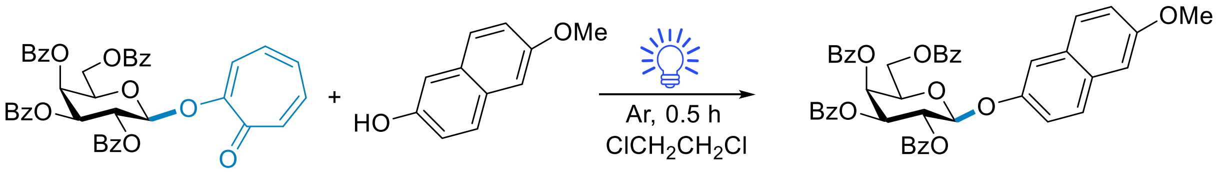

The glycosyl donor **1c** (52.5 mg, 0.0750 mmol), acceptor **2m** (8.7 mg, 0.050 mmol) and TMSOTf (0.90 uL, 0.0050 mmol) were dissolved in dry $ClCH_2CH_2Cl$ (2.0 mL), and the mixture was irradiated by blue LEDs at ambient temperature for 0.5 h. Upon completion, the solvent was concentrated under reduced pressure. The resulting residue was eluted by flash column chromatography (petroleum ether/EtOAc = 7/1) to afford the glycosylated product **3p** as white solid (36.1 mg, 96%). $[\alpha]_D^{25}$: 93.21 (*c*: 0.28 $CHCl_3$). **^{1}H NMR** (400 MHz, $CDCl_3$) δ 8.14 - 8.12 (m, 2H), 8.07 - 8.05 (m, 2H), 8.04 - 7.96 (m, 2H), 7.91 - 7.83 (m, 2H), 7.69 - 7.57 (m, 3H), 7.57 - 7.43 (m, 6H), 7.42 - 7.34 (m, 4H), 7.27 (d, *J* = 7.7 Hz, 2H), 7.19 (dd, *J* = 8.9, 2.5 Hz, 1H), 7.12 - 7.03 (m, 2H), 6.12 (dd, *J* = 10.4, 7.9 Hz, 1H), 6.07 (d, *J* = 3.4 Hz, 1H), 5.70 (dd, *J* = 10.4, 3.4 Hz, 1H), 5.46 (d, *J* = 8.0 Hz, 1H), 4.70 - 4.53 (m, 3H), 3.88 (s, 3H). **^{13}C NMR** (101 MHz, $CDCl_3$) δ 166.0, 165.5, 165.2, 156.8, 153.4, 133.7, 133.35, 133.33, 131.1, 130.0, 129.8, 129.7, 129.4, 129.26, 129.22, 128.8, 128.7, 128.67, 128.61, 128.5, 128.4, 128.3, 128.2, 119.5, 119.2, 112.1, 105.7, 100.5, 71.9, 71.7, 69.5, 68.1, 62.5, 55.3. **HRMS** (ESI) Calculated for $C_{45}H_{40}NO_{11}$ $[M+NH_4]^+$: 770.2601 Found: 770.2610

4-Methoxybenzoyl 2,3,4,6-tetra-*O*-benzoyl-β-D-galactopyranoside (3q)

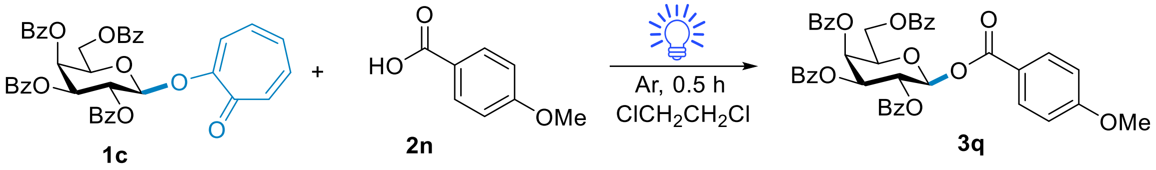

The glycosyl donor **1c** (52.5 mg, 0.0750 mmol), acceptor **2n** (7.6 mg, 0.050 mmol) and TMSOTf (0.90 uL, 0.0050 mmol) were dissolved in dry $ClCH_2CH_2Cl$ (2.0 mL), and the mixture was irradiated by blue LEDs at ambient temperature for 0.5 h. Upon completion, the solvent was concentrated under reduced pressure. The resulting residue was eluted by flash column chromatography (petroleum ether/EtOAc = 7/1) to afford the glycosylated product **3q** as a white solid (34.7 mg, 95%). $[\alpha]_D^{25}$: 42.26 (*c*: 0.31 $CHCl_3$). **^{1}H NMR** (400 MHz, $CDCl_3$) δ 8.12 (d, *J* = 7.7 Hz, 2H), 8.05 - 8.00 (m, 4H), 7.90 (d, *J* = 7.8 Hz, 2H), 7.81 (d, *J* = 7.8 Hz, 2H), 7.64 (t, *J* = 7.4 Hz, 1H), 7.56 - 7.39 (m, 7H), 7.32 (t, *J* = 7.7 Hz, 2H), 7.26 (t, *J* = 7.7 Hz, 2H), 6.97 - 6.84 (m, 2H), 6.27 (d, *J* = 8.3 Hz, 1H), 6.14 - 6.04 (m, 2H), 5.78 (dd, *J* = 10.4, 3.3 Hz, 1H), 4.68 (dd, *J* = 11.1, 6.3 Hz, 1H), 4.58 (t, *J* = 6.6 Hz, 1H), 4.45 (dd, *J* = 11.1, 6.5 Hz, 1H), 3.83 (s, 3H). **^{13}C NMR** (101 MHz, $CDCl_3$) δ 165.9, 165.47, 165.44, 165.3, 164.3, 164.0, 133.6, 133.38, 133.34, 133.2, 132.4, 130.0, 129.8, 129.79, 129.73, 129.3, 129.0, 128.8, 128.7, 128.6, 128.4, 128.39, 128.31, 120.7, 113.8, 92.8, 72.3, 71.5, 68.8, 67.9, 61.7, 55.4. **HRMS** (ESI) Calculated for $C_{42}H_{38}NO_{12}$ $[M+NH_4]^+$: 748.2394 Found: 748.2396.

(*R*)-1-*O*-(2,3,4,6-Tetra-*O*-benzoyl-β-D-galactopyranosyl)-3,7-dimethyloct-6-enoate (3r)

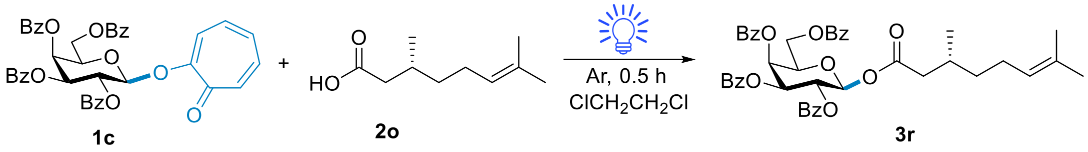

The glycosyl donor **1c** (52.5 mg, 0.0750 mmol), acceptor **2o** (8.5 mg, 0.050 mmol) and TMSOTf (0.90 uL, 0.0050 mmol) were dissolved in dry $ClCH_2CH_2Cl$ (2.0 mL), and the mixture was irradiated by blue LEDs at ambient temperature for 0.5 h. Upon completion, the solvent was concentrated under reduced pressure. The resulting residue was eluted by flash column chromatography (petroleum ether/EtOAc = 6/1) to afford the glycosylated product **3r** as a pale-yellow oil (36.3 mg, 97%). $[\alpha]_D^{25}$: 87.86 (*c*: 0.42 $CHCl_3$). **^{1}H NMR** (400 MHz, $CDCl_3$) δ 8.10 - 8.07 (m, 2H), 8.02 - 7.99 (m, 2H), 7.97 - 7.88 (m, 2H), 7.80 - 7.77 (m, 2H), 7.70 - 7.32 (m, 10H), 7.27 - 7.23 (m, 2H), 6.12 (d, *J* = 8.4 Hz, 1H), 6.04 (d, *J* = 3.4 Hz, 1H), 5.92 (dd, *J* = 10.4, 8.3 Hz, 1H), 5.66 (dd, *J* = 10.3, 3.4 Hz, 1H), 4.94 (t, *J* = 7.2 Hz, 1H), 4.64 (dd, *J* = 10.8, 6.1 Hz, 1H), 4.55 - 4.34 (m, 2H), 2.41 (dd, *J* = 15.0, 5.8 Hz, 1H), 2.22 - 2.10 (m, 1H), 1.99 - 1.72 (m, 3H), 1.63 (s, 3H), 1.54 (s, 3H), 1.18 - 1.09 (m, 1H), 0.89 - 0.86 (m, 1H), 0.75 (d, *J* = 6.6 Hz, 3H). **^{13}C NMR** (101 MHz, $CDCl_3$) δ 171.3, 165.9, 165.4, 165.1, 133.6, 133.4, 133.3, 133.2, 131.5, 130.0, 129.8, 129.79, 129.76, 129.3, 128.9, 128.8, 128.6, 128.4, 128.3, 124.0, 92.1, 72.3, 71.7, 68.7, 67.9, 61.7, 41.3, 36.5, 29.8, 25.6, 25.1, 19.1, 17.6. **HRMS** (ESI) Calculated for $C_{44}H_{48}NO_{11}$ $[M+NH_4]^+$: 766.3227 Found: 766.3231.

Methyl 2,3,4-tri-*O*-benzyl-6-*O*-(2,3,4,6-tetra-*O*-benzoyl-β-D-glucopyranosyl)-α-D-glucopyranoside (4a)

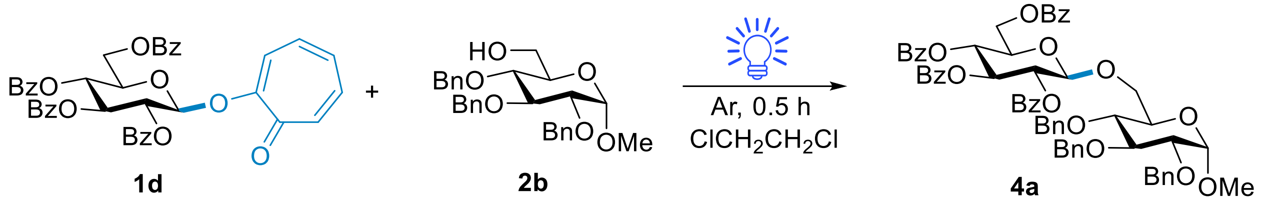

The glycosyl donor **1d** (52.5 mg, 0.0750 mmol), acceptor **2b** (23.2 mg, 0.0500 mmol) and TMSOTf (0.90 uL, 0.0050 mmol) were dissolved in dry $ClCH_2CH_2Cl$ (2.0 mL), and the mixture was irradiated by blue LEDs at ambient temperature for 0.5 h. Upon completion, the solvent was concentrated under reduced pressure. The resulting residue was eluted by flash column chromatography (petroleum ether/EtOAc = 6/1) to afford the glycosylated product **4a** as a colorless oil (51.0 mg, 98%). **^{1}H NMR** (400 MHz, $CDCl_3$) δ 8.04 - 7.95 (m, 2H), 7.90 - 7.86 (m, 4H), 7.84 - 7.79 (m, 2H), 7.55 - 7.46 (m, 2H), 7.44 - 7.26 (m, 17H), 7.26 - 7.19 (m, 6H), 7.05 - 7.03 (m, 2H), 5.88 (t, *J* = 9.6 Hz, 1H), 5.67 (t, *J* = 9.7 Hz, 1H), 5.59 (dd, *J* = 9.7, 7.8 Hz, 1H), 4.89 (d, *J* = 10.9 Hz, 1H), 4.81 (d, *J* = 7.8 Hz, 1H), 4.73 (d, *J* = 12.1 Hz, 1H), 4.68 (d, *J* = 10.9 Hz, 1H), 4.62 - 4.57 (m, 2H), 4.54 - 4.46 (m, 3H), 4.27 (d, *J* = 11.1 Hz, 1H), 4.20 - 4.04 (m, 2H), 3.88 (t, *J* = 9.3 Hz, 1H), 3.80 - 3.69 (m, 2H), 3.46 - 3.31 (m, 2H), 3.20 (s, 3H). The ^{1}H NMR data coincide with the reported data[15].

Methyl 2,3,6-tri-*O*-benzyl-4-*O*-(2,3,4,6-tetra-*O*-benzoyl-β-D-glucopyranosyl)-α-D-glucopyranoside (4b)

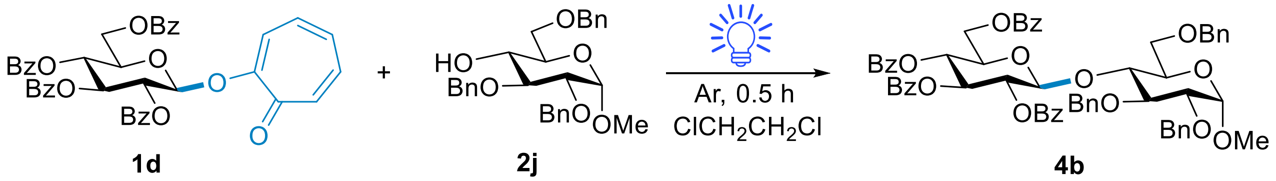

The glycosyl donor **1d** (52.5 mg, 0.0750 mmol), acceptor **2j** (23.2 mg, 0.0500 mmol) and TMSOTf (0.90 uL, 0.0050 mmol) were dissolved in dry $ClCH_2CH_2Cl$ (2.0 mL), and the mixture was irradiated by blue LEDs at ambient temperature for 0.5 h. Upon completion, the solvent was concentrated under reduced pressure. The resulting residue was eluted by flash column chromatography (petroleum ether/EtOAc = 6/1) to afford the glycosylated product **4b** as a colorless oil (42.2 mg, 81%). **^{1}H NMR** (400 MHz, $CDCl_3$) δ 7.98 - 7.92 (m, 2H), 7.92 - 7.82 (m, 4H), 7.81 - 7.73 (m, 2H), 7.58 - 7.30 (m, 17H), 7.29 - 7.24 (m, 7H), 7.22 - 7.13 (m, 3H), 5.72 - 5.37 (m, 3H), 5.06 (d, *J* = 11.2 Hz, 1H), 4.88 - 4.66 (m, 4H), 4.58 (d, *J* = 12.3 Hz, 1H), 4.54 (d, *J* = 3.7 Hz, 1H), 4.39 (dd, *J* = 12.1, 3.5 Hz, 1H), 4.34 (d, *J* = 12.1 Hz, 1H), 4.25 (dd, *J* = 12.1, 5.0 Hz, 1H), 3.96 (t, *J* = 9.4 Hz, 1H), 3.87

(t, J = 9.2 Hz, 1H), 3.73 - 3.67 (m, 2H), 3.51 - 3.40 (m, 3H), 3.27 (s, 3H). The ^{1}H NMR data coincide with the reported data[13].

Methyl 2,3,4-tri-*O*-benzyl-6-*O*-(2,3,4,6-tetra-*O*-benzoyl-α-D-mannopyranosyl)-α-D-glucopyranoside (4c)

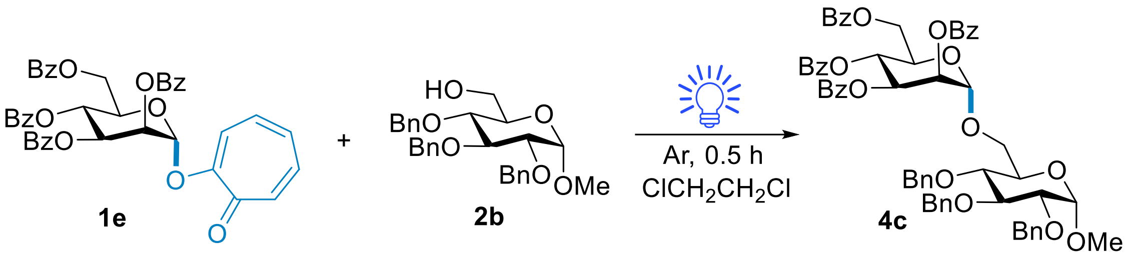

The glycosyl donor **1e** (52.5 mg, 0.0750 mmol), acceptor **2b** (23.2 mg, 0.0500 mmol) and TMSOTf (0.90 uL, 0.0050 mmol) were dissolved in dry $ClCH_2CH_2Cl$ (2.0 mL), and the mixture was irradiated by blue LEDs at ambient temperature for 0.5 h. Upon completion, the solvent was concentrated under reduced pressure. The resulting residue was eluted by flash column chromatography (petroleum ether/EtOAc = 6/1) to afford the glycosylated product **4c** as a colorless oil (50.6 mg, 97%). **^{1}H NMR** (400 MHz,$CDCl_3$) δ 8.09 - 8.03 (m, 4H), 7.91 - 7.81 (m, 4H), 7.63 - 7.47 (m, 3H), 7.46 - 7.22 (m, 24H), 6.07 (t, J = 10.1 Hz, 1H), 5.88 (dd, J = 10.1, 3.3 Hz, 1H), 5.73 (dd, J = 3.3, 1.8 Hz, 1H), 5.16 (d, J = 1.8 Hz, 1H), 5.02 (dd, J = 11.1, 2.8 Hz, 2H), 4.84 - 4.78 (m, 2H), 4.70 - 4.61 (m, 4H), 4.42 - 4.38 (m, 1H), 4.34 (dd, J = 12.0, 4.5 Hz, 1H), 4.04 (t, J = 9.3 Hz, 1H), 3.94 (dd, J = 11.0, 5.1 Hz, 1H), 3.88 - 3.79 (m, 2H), 3.64 - 3.48 (m, 2H), 3.45 (s, 3H). The ^{1}H NMR data coincide with the reported data[15].

Methyl 2,3,6-tri-*O*-benzyl-4-*O*-(2,3,4,6-tetra-*O*-benzoyl-α-D-mannopyranosyl)-α-D-glucopyranoside (4d)

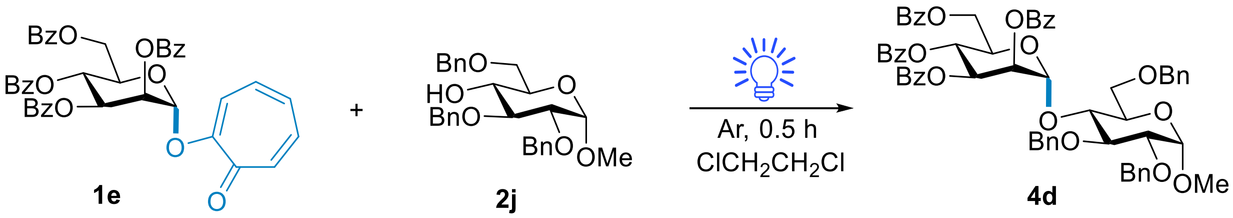

The glycosyl donor **1e** (52.5 mg, 0.0750 mmol), acceptor **2j** (23.2 mg, 0.0500 mmol) and TMSOTf (0.90 uL, 0.0050 mmol) were dissolved in dry $ClCH_2CH_2Cl$ (2.0 mL), and the mixture was irradiated by blue LEDs at ambient temperature for 0.5 h. Upon completion, the solvent was concentrated under reduced pressure. The resulting residue was eluted by flash column chromatography (petroleum ether/EtOAc = 6/1) to afford the glycosylated product **4d** as a colorless oil (42.7 mg, 82%). **^{1}H NMR** (400 MHz, $CDCl_3$) δ 8.09 - 8.03 (m, 2H), 7.93 - 7.89 (m, 4H), 7.82 - 7.76 (m, 2H), 7.64 - 7.27 (m, 20H), 7.24 - 7.18 (m, 3H), 7.16 - 7.09 (m, 1H), 7.06 - 6.93

(m, 3H), 6.02 (t, *J* = 10.1 Hz, 1H), 5.85 (dd, *J* = 10.2, 3.1 Hz, 1H), 5.72 (dd, *J* = 3.1, 1.9 Hz, 1H), 5.60 (d, *J* = 1.9 Hz, 1H), 5.06 (d, *J* = 11.0 Hz, 1H), 4.85 (d, *J* = 11.0 Hz, 1H), 4.74 (d, *J* = 12.1 Hz, 1H), 4.65 - 4.52 (m, 4H), 4.46 (dd, *J* = 12.2, 2.6 Hz, 1H), 4.36 - 4.32 (m, 1H), 4.23 (dd, *J* = 12.2, 3.7 Hz, 1H), 4.09 (t, *J* = 8.7 Hz, 1H), 3.97 - 3.83 (m, 3H), 3.77 (d, *J* = 10.4 Hz, 1H), 3.56 (dd, *J* = 9.6, 3.5 Hz, 1H), 3.43 (s, 3H). The ^{1}H NMR data coincide with the reported data[15].

Methyl 2,3,6-tri-*O*-benzyl-4-*O*-(2,3,4-tri-*O*-benzoyl-α-D-arabinopyranosyl)-α-D-glucopyranoside (4e)

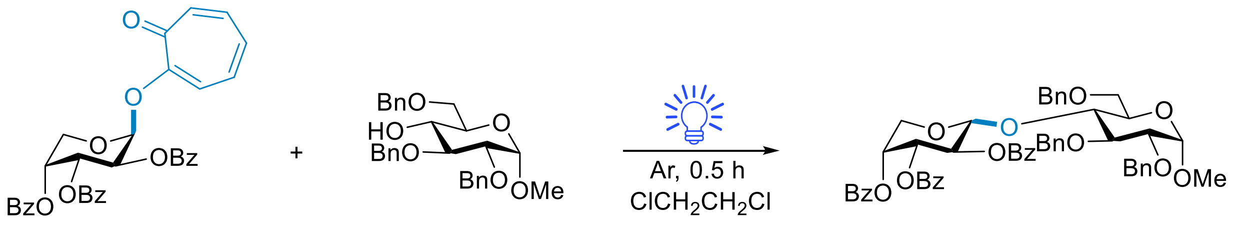

1f **2j** **4e**

The glycosyl donor **1f** (42.5 mg, 0.0750 mmol), acceptor **2j** (23.2 mg, 0.0500 mmol) and TMSOTf (0.90 uL, 0.0050 mmol) were dissolved in dry $ClCH_2CH_2Cl$ (2.0 mL), and the mixture was irradiated by blue LEDs at ambient temperature for 0.5 h. Upon completion, the solvent was concentrated under reduced pressure. The resulting residue was eluted by flash column chromatography (petroleum ether/EtOAc = 8/1) to afford the glycosylated product **4e** as a colorless oil (39.1 mg, 86%). $[\alpha]_D^{25}$: -88.75 (*c*: 0.16 $CHCl_3$). **^{1}H NMR** (400 MHz, $CDCl_3$) δ 8.11 - 8.04 (m, 2H), 7.88 - 7.84 (m, 4H), 7.65 - 7.56 (m, 1H), 7.50 - 7.37 (m, 6H), 7.35 - 7.26 (m, 12H), 7.25 - 7.19 (m, 4H), 7.18 - 7.12 (m, 1H), 5.75 (dd, *J* = 9.7, 7.2 Hz, 1H), 5.68 - 5.56 (m, 1H), 5.51 (dd, *J* = 9.6, 3.5 Hz, 1H), 5.30 (d, *J* = 7.2 Hz, 1H), 4.80 (d, *J* = 10.8 Hz, 1H), 4.66 (d, *J* = 12.0 Hz, 1H), 4.60 (d, *J* = 3.5 Hz, 1H), 4.56 - 4.47 (m, 4H), 4.11 (dd, *J* = 13.2, 3.0 Hz, 1H), 3.98 (t, *J* = 9.2 Hz, 1H), 3.87 - 3.69 (m, 5H), 3.52 (dd, *J* = 9.6, 3.5 Hz, 1H), 3.35 (s, 3H). **^{13}C NMR** (101 MHz, $CDCl_3$) δ 165.6, 165.5, 165.3, 138.5, 138.3, 137.9, 133.4, 133.28, 133.23, 129.8, 129.7, 129.6, 129.5, 129.1, 128.9, 128.58, 128.51, 128.4, 128.37, 128.34, 128.16, 128.10, 127.9, 127.6, 127.5, 127.4, 127.3, 100.8, 97.6, 81.7, 80.1, 75.2, 74.0, 73.6, 73.1, 71.0, 70.3, 69.3, 69.0, 68.8, 63.4, 55.2. **HRMS** (ESI) Calculated for $C_{54}H_{56}NO_{13}$ $[M+NH_4]^+$: 926.3752 Found: 926.3762.

Methyl 2,3,6-tri-*O*-benzyl-4-*O*-(2,3,4-tri-*O*-benzoyl-β-D-xylopyranosyl)-α-D-glucopyranoside (4f)

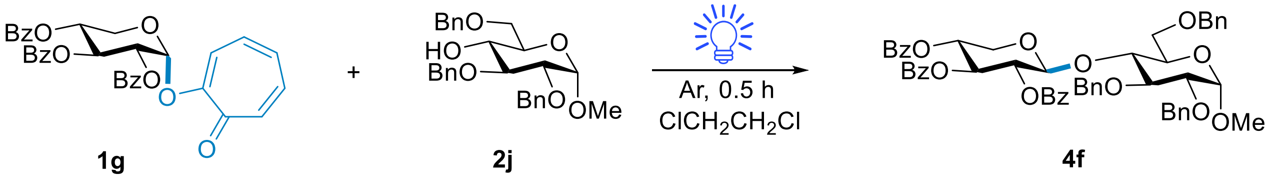

1g **2j** **4f**

The glycosyl donor **1g** (42.5 mg, 0.0750 mmol), acceptor **2j** (23.2 mg, 0.0500 mmol) and TMSOTf (0.90 uL, 0.0050 mmol) were dissolved in dry $ClCH_2CH_2Cl$ (2.0 mL), and the mixture was irradiated by blue LEDs at ambient temperature for 0.5 h. Upon completion, the solvent was concentrated under reduced pressure. The resulting residue was eluted by flash column chromatography (petroleum ether/EtOAc = 8/1) to afford the glycosylated product **4f** as a colorless oil (36.3 mg, 80%). $[\alpha]_D^{25}$: -13.33 (*c*: 0.21 $CHCl_3$). **^{1}H NMR** (400 MHz, $CDCl_3$) δ 8.06 - 7.95 (m, 2H), 7.95 - 7.84 (m, 4H), 7.59 - 7.26 (m, 24H), 5.60 (t, *J* = 8.5 Hz, 1H), 5.37 (dd, *J* = 8.6, 6.7 Hz, 1H), 5.29 - 5.23 (m, 1H), 4.95 (d, *J* = 10.5 Hz, 1H), 4.89 (d, *J* = 10.4 Hz, 1H), 4.81 (d, *J* = 12.2 Hz, 1H), 4.72 (d, *J* = 6.7 Hz, 1H), 4.66 - 4.61 (m, 2H), 4.54 (d, *J* = 3.6 Hz, 1H), 4.33 (d, *J* = 12.0 Hz, 1H), 4.27 (dd, *J* = 11.9, 4.9 Hz, 1H), 3.96 (t, *J* = 9.4 Hz, 1H), 3.88 (t, *J* = 9.2 Hz, 1H), 3.69 (dd, *J* = 10.8, 2.9 Hz, 1H), 3.53 - 3.49 (m, 2H), 3.43 (dd, *J* = 10.7, 1.9 Hz, 1H), 3.30 (s, 3H), 3.26 (dd, *J* = 12.0, 8.7 Hz, 1H). **^{13}C NMR** (101 MHz, $CDCl_3$) δ 165.5, 165.4, 164.9, 138.7, 138.2, 137.8, 133.3, 133.29, 133.25, 129.8, 129.76, 129.74, 129.2, 129.15, 129.12, 128.6, 128.4, 128.39, 128.36, 128.32, 128.1, 128.0, 127.8, 127.5, 100.2, 98.4, 79.9, 79.3, 76.6, 75.9, 73.6, 73.4, 71.6, 71.4, 69.7, 69.6, 67.6, 61.9, 55.3. **HRMS** (ESI) Calculated for $C_{54}H_{56}NO_{13}$ $[M+NH_4]^+$: 926.3752 Found: 926.3749.

Methyl 2,3,6-tri-*O*-benzyl-4-*O*-(2,3,4-tri-*O*-benzoyl-α-L-rhamnopyranosyl)-α-D-glucopyranoside (4g)

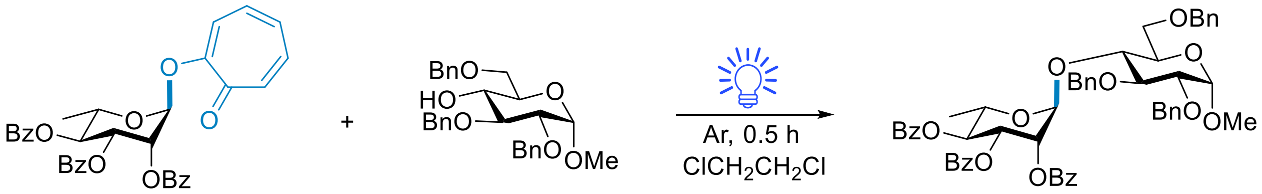

The glycosyl donor **1h** (43.5 mg, 0.0750 mmol), acceptor **2j** (23.2 mg, 0.0500 mmol) and TMSOTf (0.90 uL, 0.0050 mmol) were dissolved in dry $ClCH_2CH_2Cl$ (2.0 mL), and the mixture was irradiated by blue LEDs at ambient temperature for 0.5 h. Upon completion, the solvent was concentrated under reduced pressure. The resulting residue was eluted by flash column chromatography (petroleum ether/EtOAc = 8/1) to afford the glycosylated product **4g** as a white solid (37.8 mg, 82%). **^{1}H NMR** (400 MHz, $CDCl_3$) δ 8.08 - 8.03 (m, 2H), 7.90 - 7.83 (m, 4H), 7.62 - 7.36 (m, 9H), 7.36 - 7.26 (m, 9H), 7.22 - 7.07 (m, 6H), 5.78 (dd, *J* = 10.2, 3.4 Hz, 1H), 5.65 - 5.51 (m, 2H), 5.26 - 5.16 (m, 2H), 4.84 (d, *J* = 11.1 Hz, 1H), 4.76 (d, *J* = 12.1 Hz, 1H), 4.68 - 4.51 (m, 4H), 4.36 (dd, *J* = 10.0, 6.1 Hz, 1H), 4.05 - 3.80 (m, 4H), 3.73 (dd, *J* = 11.2, 1.8 Hz, 1H), 3.64 (dd, *J* = 9.0, 3.6 Hz, 1H), 3.41 (s, 3H), 0.88 (d, *J* = 6.1 Hz, 3H). The ^{1}H NMR data coincide with the reported data[17].

Methyl 2,3,6-tri-*O*-benzyl-4-*O*-(2,3,4-tri-*O*-benzoyl-β-D-ribopyranosyl)-α-D-glucopyranoside (4h)

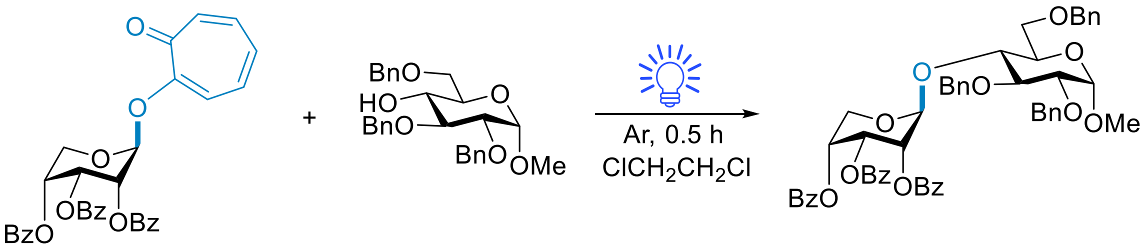

1i **2j** **4h**

The glycosyl donor **1i** (42.5 mg, 0.0750 mmol), acceptor **2j** (23.2 mg, 0.0500 mmol) and TMSOTf (0.90 uL, 0.0050 mmol) were dissolved in dry $ClCH_2CH_2Cl$ (2.0 mL), and the mixture was irradiated by blue LEDs at ambient temperature for 0.5 h. Upon completion, the solvent was concentrated under reduced pressure. The resulting residue was eluted by flash column chromatography (petroleum ether/EtOAc = 8/1) to afford the glycosylated product **4h** as a colorless oil (36.3 mg, 80%). $[\alpha]_D^{25}$: -28.00 (*c*: 0.15 $CHCl_3$). **^{1}H NMR** (400 MHz, $CDCl_3$) δ 8.00 - 7.98 (m, 2H), 7.94 - 7.91 (m, 2H), 7.88 - 7.85 (m, 2H), 7.54 - 7.45 (m, 5H), 7.42 - 7.23 (m, 15H), 7.22 - 7.09 (m, 4H), 5.79 - 5.76 (m, 1H), 5.40 - 5.29 (m, 3H), 5.12 - 5.08 (d, *J* = 10.6 Hz, 1H), 4.90 - 4.73 (m, 2H), 4.71 - 4.58 (m, 2H), 4.58 - 4.44 (m, 2H), 4.32 (dd, *J* = 13.1, 3.0 Hz, 1H), 4.06 - 3.96 (m, 2H), 3.93 - 3.82 (m, 1H), 3.78 - 3.76 (m, 1H), 3.74 - 3.64 (m, 2H), 3.62 (dd, *J* = 8.9, 3.6 Hz, 1H), 3.38 (s, 3H). **^{13}C NMR** (101 MHz, $CDCl_3$) δ 166.0, 165.9, 165.2, 138.6, 138.0, 137.8, 133.2, 133.0, 129.97, 129.95, 129.92, 129.7, 129.6, 129.4, 128.5, 128.4, 128.3, 128.25, 128.21, 128.0, 127.7, 127.5, 127.4, 98.2, 98.1, 80.3, 80.0, 75.7, 75.2, 73.4, 73.2, 70.0, 69.5, 68.3, 67.6, 66.5, 61.6, 55.3. **HRMS** (ESI) Calculated for $C_{54}H_{56}NO_{13}$ $[M+NH_4]^+$: 926.3752 Found: 926.3760

Methyl 2,3,4-tri-*O*-benzyl-6-*O*-(3,4,6-*O*-tri-benzoyl-2-deoxy-2-phthalimido-β-D-glucopyranosyl)-α-D-glucopyranoside (4i)

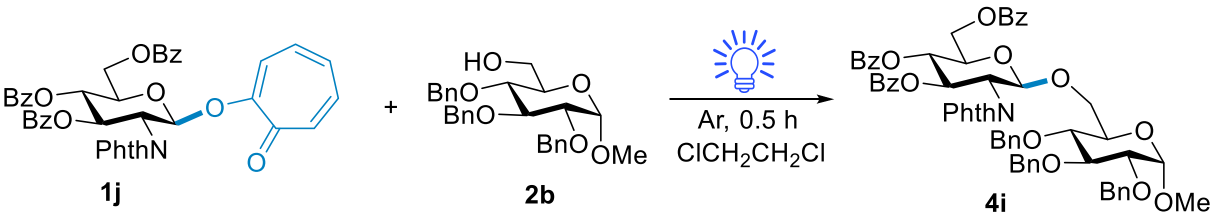

1j **2b** **4i**

The glycosyl donor **1j** (54.4 mg, 0.0750 mmol), acceptor **2b** (23.2 mg, 0.0500 mmol) and TMSOTf (0.90 uL, 0.0050 mmol) were dissolved in dry $ClCH_2CH_2Cl$ (2.0 mL), and the mixture was irradiated by blue LEDs for 0.5 h. Upon completion, the solvent was concentrated under reduced pressure. The resulting residue was eluted by flash column chromatography (petroleum ether/EtOAc = 8/1) to afford the glycosylated product **4i** as a pale-yellow solid (42.7 mg, 80%). **^{1}H NMR** (400 MHz, $CDCl_3$) δ 8.00 (d, *J* = 7.8 Hz, 2H), 7.89 (d, *J* = 7.8 Hz, 2H), 7.74 (d, *J* = 7.7 Hz, 2H),7.60 - 7.57 (m, 2H), 7.55 - 7.27 (m, 16H), 7.26 - 7.20 (m, 8H), 7.03 - 7.01 (m, 2H), 6.28 (t, *J* = 10.0 Hz, 1H), 5.70 (t, *J* = 9.6 Hz, 1H), 5.64 (d, *J* = 8.4 Hz, 1H), 4.85 (d, *J* = 10.8 Hz, 1H), 4.74 - 4.50 (m, 6H), 4.45 - 4.36 (m, 2H), 4.28 - 4.24 (m, 1H), 4.13 (d, *J* = 10.4 Hz, 2H), 3.84 (t, *J* = 9.3 Hz, 1H), 3.74 - 3.65 (m, 2H), 3.39

(dd, J = 9.6, 3.5 Hz, 1H), 3.26 (t, J = 9.4 Hz, 1H), 3.16 (s, 3H). The ^{1}H NMR data coincide with the reported data[18].

Methyl 2,3,4-tri-*O*-benzyl-6-*O*-(2,3,4,6-tetra-*O*-acetyl-β-D-galactopyranosyl)-α-D-glucopyranoside (4j)

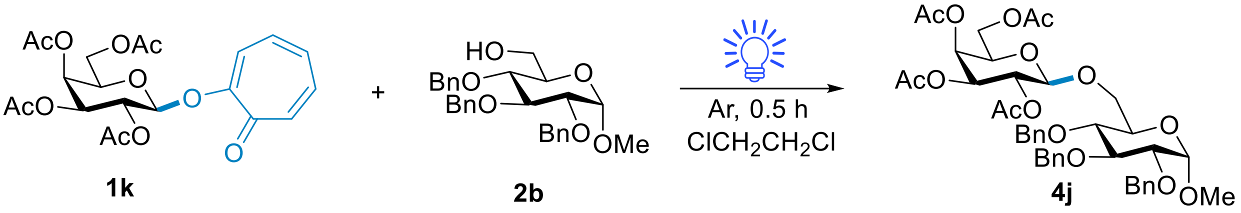

The glycosyl donor **1k** (33.9 mg, 0.0750 mmol), acceptor **2b** (23.2 mg, 0.0500 mmol) and TMSOTf (0.90 uL, 0.0050 mmol) were dissolved in dry $ClCH_2CH_2Cl$ (2.0 mL), and the mixture was irradiated by blue LEDs at ambient temperature for 0.5 h. Upon completion, the solvent was concentrated under reduced pressure. The resulting residue was eluted by flash column chromatography (petroleum ether/EtOAc = 6/1) to afford the glycosylated product **4j** as a colorless oil (30.2 mg, 76%). **^{1}H NMR** (400 MHz,$CDCl_3$) δ 7.37 - 7.27 (m, 14H), 7.24 - 7.23 (m, 1H), 5.36 (dd, J = 3.6, 1.2 Hz, 1H), 5.30 - 5.22 (m, 1H), 5.01 - 4.95 (m, 2H), 4.86 (d, J = 10.9 Hz, 1H), 4.83 - 4.76 (m, 2H), 4.65 (d, J = 12.1 Hz, 1H), 4.58 (d, J = 3.5 Hz, 1H), 4.53 (d, J = 10.9 Hz, 1H), 4.47 (d, J = 8.0 Hz, 1H), 4.17 - 4.06 (m, 3H), 3.98 (t, J = 9.2 Hz, 1H), 3.85 (t, J = 6.8 Hz, 1H), 3.78 (dd, J = 10.3, 4.7 Hz, 1H), 3.66 (dd, J = 10.7, 5.0 Hz, 1H), 3.51 (dd, J = 9.7, 3.6 Hz, 1H), 3.41 (dd, J = 9.9, 8.9 Hz, 1H), 3.36 (s, 3H), 2.12 (s, 3H), 2.02 (s, 3H), 1.97 (s, 3H), 1.96 (s, 3H). The ^{1}H NMR data coincide with the reported data[19].

Methyl 2,3,4-tri-*O*-benzyl-6-*O*-(2,3,4,6-tetra-*O*-acetyl-β-D-glucopyranosyl)-α-D-glucopyranoside (4k)

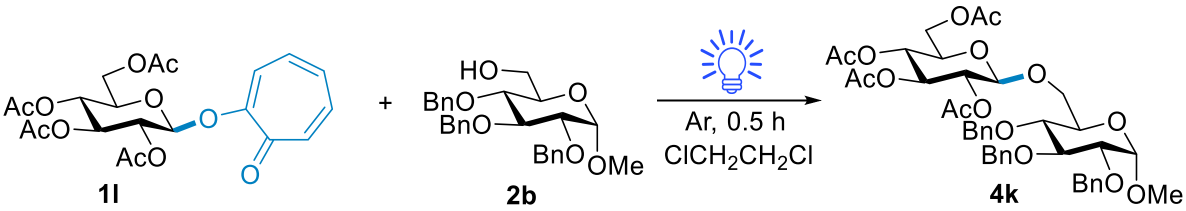

The glycosyl donor **1l** (33.9 mg, 0.0750 mmol), acceptor **2b** (23.2 mg, 0.0500 mmol) and TMSOTf (0.90 uL, 0.0050 mmol) were dissolved in dry $ClCH_2CH_2Cl$ (2.0 mL), and the mixture was irradiated by blue LEDs at ambient temperature for 0.5 h. Upon completion, the solvent was concentrated under reduced pressure. The resulting residue was eluted by flash column chromatography (petroleum ether/EtOAc = 6/1) to afford the glycosylated product **4k** as a colorless oil (27.8 mg, 70%). **^{1}H NMR** (400 MHz,$CDCl_3$) δ 7.36 - 7.26 (m, 14H), 7.24 (d, J = 1.5 Hz, 1H), 5.17 (t, J = 9.4 Hz, 1H), 5.11 - 5.02 (m, 2H), 4.98 (d, J = 10.9 Hz, 1H), 4.86 (d, J = 10.9

Hz, 1H), 4.83 - 4.75 (m, 2H), 4.65 (d, *J* = 12.1 Hz, 1H), 4.57 (d, *J* = 3.5 Hz, 1H), 4.55 - 4.49 (m, 2H), 4.23 (dd, *J* = 12.3, 4.7 Hz, 1H), 4.12 (dd, *J* = 12.3, 2.5 Hz, 1H), 4.06 (dd, *J* = 10.7, 1.8 Hz, 1H), 3.97 (t, *J* = 9.3 Hz, 1H), 3.78 - 3.74 (m, 1H), 3.71 - 3.62 (m, 2H), 3.51 (dd, *J* = 9.7, 3.6 Hz, 1H), 3.42 (dd, *J* = 10.0, 8.9 Hz, 1H), 3.36 (s, 3H), 2.05 (s, 3H), 2.02 (s, 3H), 1.99 (s, 3H), 1.95 (s, 3H). The ^{1}H NMR data coincide with the reported data[19].

Methyl 2,3,6-tri-*O*-benzyl-4-*O*-(2,3,4,6-tetra-*O*-acetyl-β-D-galactopyranosyl)-α-D-glucopyranoside (4l)

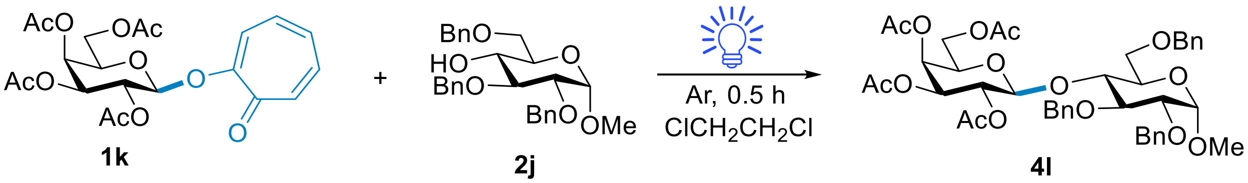

The glycosyl donor **1k** (33.9 mg, 0.0750 mmol), acceptor **2j** (23.2 mg, 0.0500 mmol) and TMSOTf (0.90 uL, 0.0050 mmol) were dissolved in dry $ClCH_2CH_2Cl$ (2.0 mL), and the mixture was irradiated by blue LEDs at ambient temperature for 0.5 h. Upon completion, the solvent was concentrated under reduced pressure. The resulting residue was eluted by flash column chromatography (petroleum ether/EtOAc = 6/1) to afford the glycosylated product **4l** as a colorless oil (24.6 mg, 62%). **^{1}H NMR** (400 MHz, $CDCl_3$) δ 7.45 - 7.26 (m, 15H), 5.24 (dd, *J* = 3.5, 1.1 Hz, 1H), 5.08 (dd, *J* = 10.4, 7.9 Hz, 1H), 4.95 (d, *J* = 10.9 Hz, 1H), 4.86 - 4.71 (m, 4H), 4.63 (d, *J* = 12.3 Hz, 1H), 4.59 (d, *J* = 3.7 Hz, 1H), 4.51 - 4.36 (m, 2H), 4.01 - 3.79 (m, 4H), 3.74 (dd, *J* = 10.6, 2.8 Hz, 1H), 3.64 - 3.57 (m, 2H), 3.56 -3.46 (m, 2H), 3.37 (s, 3H), 2.09 (s, 3H), 1.99 (s, 3H), 1.96 (s, 3H), 1.95 (s, 3H). The ^{1}H NMR data coincide with the reported data[19].

Methyl 2,3,4-tri-*O*-benzyl-6-*O*-(2,3,4,6-tetra-*O*-benzyl-α/β-D-galactopyranosyl)-α-D-glucopyranoside (4m)

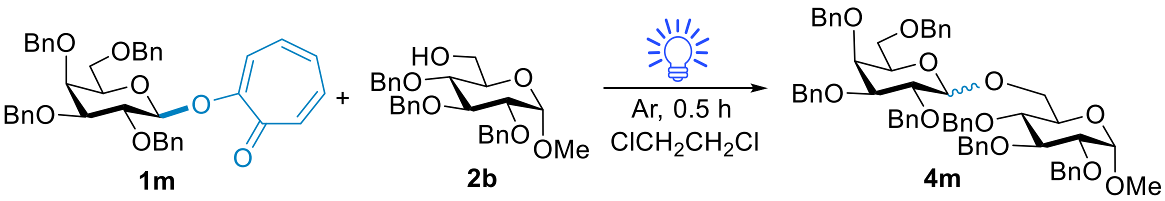

The glycosyl donor **1m** (48.3 mg, 0.0750 mmol), acceptor **2b** (23.2 mg, 0.0500 mmol) and TMSOTf (0.45 uL, 0.0025 mmol) were dissolved in dry $ClCH_2CH_2Cl$ (2.0 mL), and the mixture was irradiated by blue LEDs at ambient temperature for 0.5 h. Upon completion, the solvent was concentrated under reduced pressure. The resulting residue was eluted by flash column chromatography (petroleum ether/EtOAc = 8/1) to afford the glycosylated product **4m** as a colorless oil (42.4 mg, 86%, α/β =2.9:1). **^{1}H NMR** (400 MHz, $CDCl_3$) δ 7.35 - 7.15 (m, 60H), 4.99 - 4.91 (m, 4.7H), 4.86 - 4.65 (m, 11H), 4.63 - 4.49 (m, 6.5H), 4.45 - 4.29 (m, 4 H), 4.14 (dd, *J* =

10.8, 2.0 Hz, 0.5H), 4.04 - 3.86 (m, 7H), 3.84 - 3.71 (m, 4.4H), 3.62 - 3.38 (m, 8.7H), 3.29 (s, 3H), 3.28 (s, 3H). The ^{1}H NMR data coincide with the reported data[13].

p-Tolyl 2,3-di-*O*-benzoyl-4-*O*-benzyl-6-*O*-(2,3-di-*O*-benzoyl-4-*O*-benzyl-6-*O*-tert-butyldiphenylsilyl-β-D-glucopyranosyl)-β-D-thioglucopyranoside (4n)

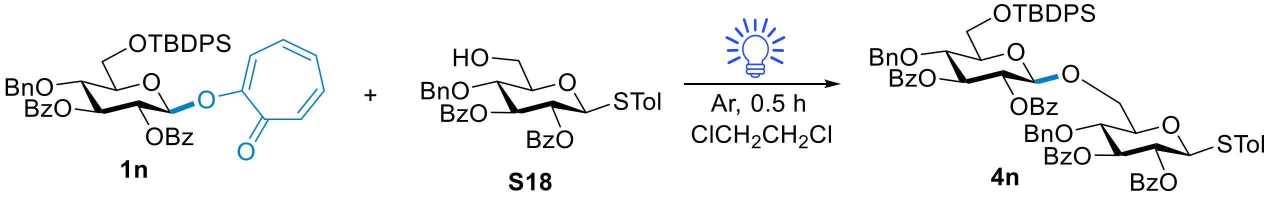

The glycosyl donor **1n** (61.5 mg, 0.0750 mmol), acceptor **S18** (29.2 mg, 0.0500 mmol) and TMSOTf (0.90 uL, 0.0050 mmol) were dissolved in dry $ClCH_2CH_2Cl$ (2.0 mL), and the mixture was irradiated by blue LEDs at ambient temperature for 0.5 h. Upon completion, the solvent was concentrated under reduced pressure. The resulting residue was eluted by flash column chromatography (petroleum ether/EtOAc = 8/1) to afford the glycosylated product **4n** as a pale-yellow solid (57.7 mg, 90%). $[\alpha]_D^{25}$: 30.95 (*c*: 0.21 $CHCl_3$). **^{1}H NMR** (400 MHz, $CDCl_3$) δ 8.02 - 7.95 (m, 2H), 7.93 - 7.88 (m, 4H), 7.81 - 7.77 (m, 4H), 7.77 - 7.71 (m, 2H), 7.51 - 7.29 (m, 18H), 7.26 - 7.21 (m, 2H), 7.15 - 7.05 (m, 10H), 6.92 - 6.89 (m, 2H), 5.75 (t, *J* = 9.5 Hz, 1H), 5.68 - 5.59 (m, 1H), 5.49 (dd, *J* = 9.8, 7.8 Hz, 1H), 5.24 (t, *J* = 9.6 Hz, 1H), 4.78 - 4.75 (m, 2H), 4.73 - 4.61 (m, 2H), 4.29 - 4.17 (m, 3H), 4.13 (t, *J* = 9.4 Hz, 1H), 4.03 (d, *J* = 2.7 Hz, 2H), 3.81 - 3.73 (m, 1H), 3.70 - 3.62 (m, 2H), 3.57 - 3.55 (m, 1H), 2.31 (s, 3H), 1.13 (s, 9H). **^{13}C NMR** (101 MHz, $CDCl_3$) δ 165.8, 165.5, 165.3, 165.1, 138.4, 137.5, 137.1, 135.9, 135.6, 133.59, 133.57, 133.17, 133.10, 132.9, 132.8, 129.86, 129.84, 129.82, 129.79, 129.76, 129.72, 129.59, 129.58, 129.53, 129.4, 128.4, 128.35, 128.33, 128.28, 128.26, 127.9, 127.84, 127.81, 127.76, 127.70, 101.0, 86.3, 79.0, 76.5, 76.1, 76.0, 75.8, 75.3, 75.0, 74.6, 72.4, 70.7, 67.3, 62.5, 27.0, 21.2, 19.4. **HRMS** (ESI) Calculated for $C_{77}H_{78}NO_{14}SSi$ $[M+NH_4]^+$: 1300.4912 Found: 1300.4929.

Methyl 2,3,6-tri-*O*-benzyl-4-*O*-(hepta-*O*-benzoyl-β-D-lactosyl)-α-D-glucopyranoside (4o)

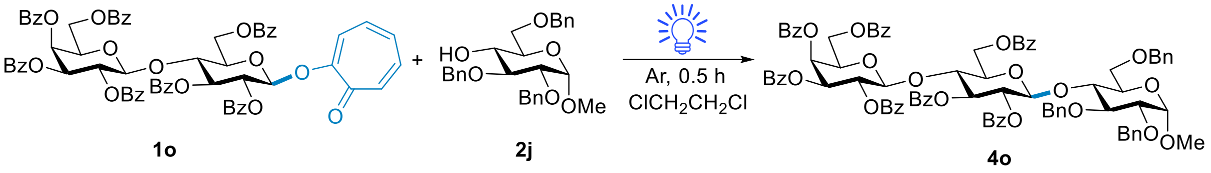

The glycosyl donor **1o** (88.1 mg, 0.0750 mmol), acceptor **2j** (23.2 mg, 0.0500 mmol) and TMSOTf (0.90 uL, 0.0050 mmol) were dissolved in dry $ClCH_2CH_2Cl$ (2.0 mL), and the mixture was irradiated by blue LEDs at ambient temperature for 0.5 h. Upon completion, the solvent was concentrated under reduced pressure. The resulting residue was eluted by flash column chromatography (petroleum ether/EtOAc = 6/1) to afford the

glycosylated product **4o** as a colorless oil (59.1 mg, 78%). $[\alpha]_D^{25}$: 28.42 (*c*: 0.19 $CHCl_3$). **^{1}H NMR** (400 MHz, $CDCl_3$) δ 8.07 (d, *J* = 7.5 Hz, 2H), 8.00 (d, *J* = 7.6 Hz, 2H), 7.96 (d, *J* = 7.8 Hz, 4H), 7.93 - 7.85 (m, 4H), 7.73 (d, *J* = 7.6 Hz, 2H), 7.64 - 7.45 (m, 9H), 7.44 - 7.29 (m, 13H), 7.23 - 7.17 (m, 9H), 7.11 (t, *J* = 7.6 Hz, 2H), 7.02 - 6.95 (m, 3H), 5.77 - 5.65 (m, 2H), 5.48 (t, *J* = 9.4 Hz, 1H), 5.38 (dd, *J* = 10.0, 7.9 Hz, 1H), 5.28 (dd, *J* = 10.3, 3.4 Hz, 1H), 5.00 (d, *J* = 11.4 Hz, 1H), 4.76 - 4.63 (m, 4H), 4.60 - 4.47 (m, 3H), 4.36 - 4.22 (m, 3H), 4.13 (t, *J* = 9.4 Hz, 1H), 3.89 - 3.77 (m, 2H), 3.74 (t, *J* = 6.7 Hz, 1H), 3.68 - 3.59 (m, 3H), 3.49 - 3.35 (m, 3H), 3.25 (s, 3H), 3.24 - 3.20 (m, 1H). **^{13}C NMR** (101 MHz, $CDCl_3$) δ 165.7, 165.5, 165.3, 165.2, 165.1, 164.9, 164.8, 139.2, 138.2, 137.6, 133.4, 133.3, 133.29, 133.26, 133.22, 133.0, 129.9, 129.8, 129.76, 129.72, 129.6, 129.59, 129.52, 129.50, 129.4, 129.2, 128.8, 128.7, 128.69, 128.67, 128.63, 128.58, 128.56, 128.55, 128.4, 128.29, 128.28, 128.23, 128.1, 128.0, 127.8, 127.6, 126.98, 126.90, 100.6, 100.3, 98.4, 80.0, 78.5, 77.2, 75.4, 75.1, 73.5, 73.4, 73.0, 72.6, 72.1, 71.7, 71.3, 69.8, 69.4, 67.5, 67.4, 62.4, 60.9, 55.2. **HRMS** (ESI) Calculated for $C_{89}H_{84}NO_{23}$ $[M+NH_4]^+$: 1534.5434 Found: 1534.5444.

2,3,4,6-Tetra-*O*-benzoyl-β-D-glucopyranosyl(dibenzyl)phosphate (5a)

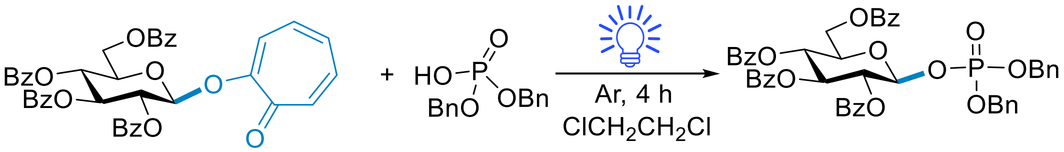

The mixture of the glycosyl donor **1d** (42.0 mg, 0.0600 mmol), acceptor **2q** (13.9 mg, 0.0500 mmol) and NaOTf (0.9 mg, 0.005 mmol) in dry $ClCH_2CH_2Cl$ (2.0 mL), was irradiated by blue LEDs at ambient temperature for 4 h. Upon completion, Et_3N (0.1 mL) was added to the reaction. The solvent was removed in vacuo, and the residue was loaded to silica gel column which was neutralized by using hexanes/Et_3N (100/1) and purified by eluting with solvents (petroleum ether/EtOAc = 4/1) to give the product **5a** (35.5 mg, 83%) as a yellow oil. **^{1}H NMR** (400 MHz, $CDCl_3$) δ 8.04 - 7.80 (m, 8H), 7.60 - 7.27 (m, 13H), 7.26 - 7.13 (m, 7H), 7.05 - 6.92 (m, 2H), 5.93 (t, *J* = 9.6 Hz, 1H), 5.79 - 5.65 (m, 3H), 5.10 - 4.98 (m, 2H), 4.84 (dd, *J* = 11.7, 6.7 Hz, 1H), 4.74 (dd, *J* = 11.7, 7.1 Hz, 1H), 4.65 (dd, *J* = 12.4, 2.8 Hz, 1H), 4.47 (dd, *J* = 12.3, 5.2 Hz, 1H), 4.32 - 4.27 (m, 1H). ^{31}P NMR (162 MHz, $CDCl_3$) δ -3.16. The ^{1}H NMR data coincide with the reported data[20].

Triethylammonium (2,3,4,6-tetra-*O*-benzoyl-β-D-glucopyranos-1-yl)(methyl 2,3,4-tri-*O*-benzyl-α-D-glucopyranosid-6-yl)phosphate (5b)

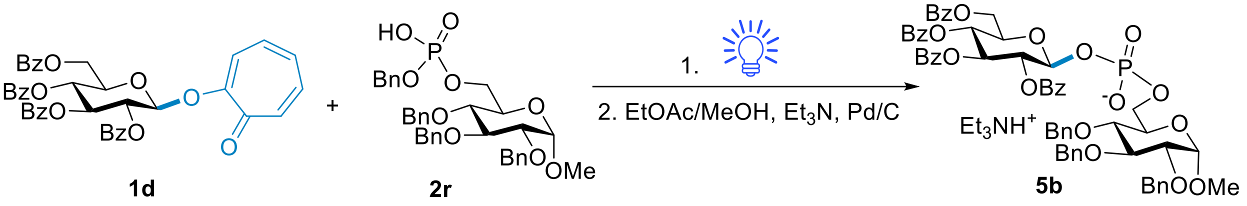

The mixture of the glycosyl donor **1d** (42.0 mg, 0.0600 mmol), acceptor **2r** (31.7 mg, 0.0500 mmol) and NaOTf (4.3 mg, 0.025 mmol) in dry $ClCH_2CH_2Cl$ (2.0 mL), was irradiated by blue LEDs at ambient temperature for 10 h. Upon completion, Et_3N (0.1 mL) was added to the reaction. The solvent was removed in vacuo, and the residue was loaded to silica gel column which was neutralized by using hexanes/Et_3N (100/1) and purified by eluting with solvents (petroleum ether/EtOAc = 8/1) to give an intermediate product benzyl phosphate (39.3 mg, 65%). To a solution of above product in EtOAc/MeOH (2.0 mL/2.0 mL) was added Et_3N (40.0 μL) and Pd/C (30.0 mg). Then, the mixture was stirred under an atmosphere of H_2 at room temperature for 0.5 h, until TLC showed the completion of hydrogenolysis. After filtration through glass funnel filter, the filtrate was concentrated to afford the residue. The residue was loaded to silica gel column which was neutralized by using hexanes/Et_3N (100/1) and purified by eluting with solvents (DCM/MeOH = 25/1) to afford **5b** as a white solid (quantitative yield). $[a]_D^{25}$: -6.67 (*c*: 0.075 $CHCl_3$). **^{1}H NMR** (400 MHz, $CDCl_3$) δ 7.99 - 7.94 (m, 5H), 7.89 - 7.87 (m, 2H), 7.81 - 7.79 (m, 2H), 7.55 - 7.28 (m, 24H), 7.24 - 7.21 (m, 2H), 5.86 (t, *J* = 9.6 Hz, 1H), 5.78 - 5.67 (m, 2H), 5.58 - 5.54 (m, 1H), 4.90 (d, *J* = 11.0 Hz, 1H), 4.81 - 4.55 (m, 6H), 4.47 - 4.35 (m, 2H), 4.24 - 4.19 (m, 1H), 4.08 - 4.02 (m, 2H), 3.85 (t, *J* = 9.2 Hz, 1H), 3.50 - 3.48 (m, 1H), 3.42 - 3.30 (m, 2H), 3.20 (s, 3H). **^{31}P NMR** (162 MHz, $CDCl_3$) δ -2.96. **^{13}C NMR** (101 MHz, $CDCl_3$) δ 166.0, 165.6, 165.3, 165.2, 139.1, 138.6, 138.3, 133.3, 133.06, 133.00, 130.0, 129.8, 129.7, 129.57, 129.54, 129.0, 128.9, 128.4, 128.3, 128.27, 128.25, 128.21, 128.1, 128.0, 127.9, 127.7, 127.4, 127.3, 97.6, 96.2 (d, *J* = 5 Hz), 81.9, 79.6, 77.7, 75.4, 74.8, 73.3, 73.0, 72.5 (d, *J* = 9 Hz), 72.2, 69.9 (d, *J* = 9 Hz), 69.5, 64.5 (d, *J* = 6 Hz), 63.0, 54.9. **HRMS** (m/z): $[M-Et_3NH^++Na^++H^+]^+$calculated for $C_{62}H_{59}O_{18}PNa^+$: 1145.3337 Found 1145.3344.

Triethylammonium (2,3,4,6-tetra-*O*-benzoyl-β-D-glucopyranos-1-yl)(methyl 2-*O*-benzyl-4,6-*O*-benzylidene-α-D-glucopyranosid-3-yl)phosphate (5c)

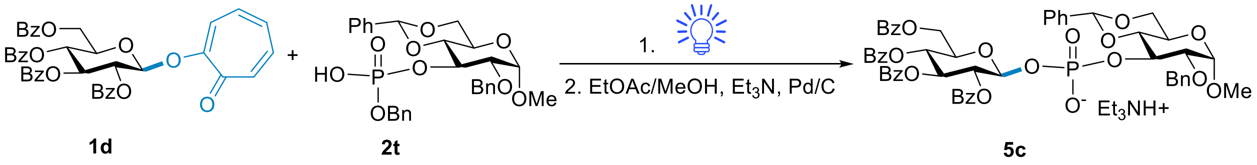

The mixture of the glycosyl donor **1d** (42.0 mg, 0.0600 mmol), acceptor **2t** (27.1 mg, 0.0500 mmol) and NaOTf (4.3 mg, 0.025 mmol) in dry $ClCH_2CH_2Cl$ (2.0 mL), was irradiated by blue LEDs at ambient temperature for 10 h. Upon completion, Et_3N (0.1 mL) was added to the reaction. The solvent was removed in vacuo, and the residue was loaded to silica gel column which was neutralized by using hexanes/Et_3N (100/1) and purified by eluting with solvents (petroleum ether/EtOAc = 6/1) to give an intermediate product benzyl phosphate (33.6 mg, 60%). To a

solution of above product in EtOAc/MeOH (2.0 mL/2.0 mL) was added Et_3N (40.0 µL) and Pd/C (30.0 mg). Then, the mixture was stirred under an atmosphere of H_2 at room temperature for 0.5 h, until TLC showed the completion of hydrogenolysis. After filtration through glass funnel filter, the filtrate was concentrated to afford the residue. The residue was loaded to silica gel column which was neutralized by using hexanes/Et_3N (100/1) and purified by eluting with solvents (DCM/MeOH = 25/1) to afford **5c** as a white solid (quantitative yield). $[\alpha]_D^{25}$: 19.5 (*c*: 0.21 $CHCl_3$). **^{1}H NMR** (400 MHz, $CDCl_3$) δ 8.09 (d, *J* = 7.8 Hz, 2H), 8.03 (d, *J* = 7.7 Hz, 2H), 7.89 (d, *J* = 7.7 Hz, 2H), 7.81 (d, *J* = 7.7 Hz, 2H), 7.67 (d, *J* = 7.6 Hz, 2H), 7.56 - 7.30 (m, 19H), 7.26 - 7.24 (m, 1H), 5.89 (t, *J* = 7.6 Hz, 1H), 5.70 (t, *J* = 9.6 Hz, 1H), 5.61 (t, *J* = 9.7 Hz, 1H), 5.50 - 5.47 (m, 2H), 4.94 (d, *J* = 12.4 Hz, 1H), 4.65 - 4.60 (m, 2H), 4.52 (d, *J* = 12.3 Hz, 1H), 4.38 - 4.35 (m, 2H), 4.14 - 4.11 (m, 1H), 4.04 - 4.02 (m, 1H), 3.63 - 3.57 (m, 1H), 3.51 - 3.46 (m, 1H), 3.25 (s, 3H), 3.12 (t, *J* = 9.4 Hz, 1H), 3.03 - 3.01 (m, 1H). **^{31}P NMR** (162 MHz, $CDCl_3$) δ -4.44. **^{13}C NMR** (101 MHz, $CDCl_3$) δ 166.1, 165.5, 165.3, 165.2, 139.0, 137.8, 133.3, 133.0, 132.95, 132.92, 130.4, 129.87, 129.82, 129.7, 129.6, 129.1, 128.9, 128.7, 128.4, 128.33, 128.31, 128.24, 128.21, 128.1, 128.0, 127.4, 126.6, 100.9, 99.5, 96.5 (d, *J* = 4.0 Hz), 80.3, 78.7 (d, *J* = 3.0 Hz), 75.2 (d, *J* = 6.0 Hz), 73.9, 73.3, 72.7 (d, *J* = 7.0 Hz), 72.1, 69.5, 68.8, 62.9, 62.2, 55.1. **HRMS** (m/z): $[M-Et_3NH^++Na^++H^+]^+$calculated for $C_{55}H_{51}O_{18}PNa^+$: 1053.2711 Found: 1053.2733.

Triethylammonium (2,3,4,6-tetra-*O*-benzoyl-β-D-glucopyranos-1-yl)(methyl *N*-(*tert*-butoxycarbonyl)-L-serin-3-yl)phosphate (5d)

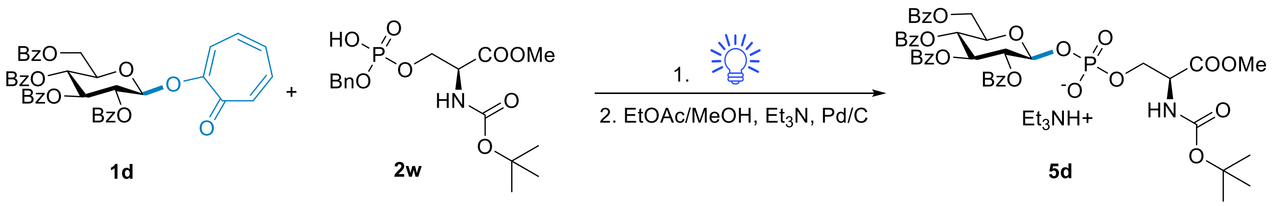

The mixture of the glycosyl donor **1d** (42.0 mg, 0.0600 mmol), acceptor **2w** (19.5 mg, 0.0500 mmol) and NaOTf (4.3 mg, 0.025 mmol) in dry $ClCH_2CH_2Cl$ (2.0 mL), was irradiated by blue LEDs at ambient temperature for 8 h. Upon completion, Et_3N (0.1 mL) was added to the reaction. The solvent was removed in vacuo, and the residue was loaded to silica gel column which was neutralized by using hexanes/Et_3N (100/1) and purified by eluting with solvents (petroleum ether/EtOAc = 6/1) to give an intermediate product benzyl phosphate (29.4 mg, 61%). To a solution of above product in EtOAc/MeOH (2.0 mL/2.0 mL) was added Et_3N (40.0 µL) and Pd/C (30.0 mg). Then, the mixture was stirred under an atmosphere of H_2 at room temperature for 0.5 h, until TLC showed the completion of hydrogenolysis. After filtration through glass funnel filter, the filtrate was concentrated to afford the residue. The residue was loaded to silica gel column which was neutralized by using hexanes/Et_3N (100/1) and purified by eluting with solvents (DCM/MeOH = 25/1) to afford **5d** as a colorless oil (quantitative yield). $[\alpha]_D^{25}$: 6.36 (*c*: 0.22 $CHCl_3$). **^{1}H NMR** (400 MHz, $CDCl_3$) δ 8.08 - 7.77 (m, 8H), 7.57 - 7.32 (m, 10H), 7.27 - 7.25 (m, 2H), 6.53 (d, *J* = 7.2 Hz, 1H), 5.88 (t, *J* = 9.6 Hz, 1H), 5.78 - 5.64 (m, 2H), 5.64 - 5.52 (m, 1H), 4.67 (dd, *J* = 12.3, 3.0 Hz, 1H),

4.51 (dd, J = 12.2, 4.7 Hz, 1H), 4.30 - 4.25 (m, 1H), 4.17 - 3.98 (m, 3H), 3.59 (s, 3H), 1.46 (s, 9H). ^{31}**P NMR** (162 MHz, $CDCl_3$) δ -2.50. ^{13}**C NMR** (101 MHz, $CDCl_3$) δ 170.6, 166.0, 165.6, 165.19, 165.17, 155.7, 133.3, 133.19, 133.11, 129.89, 129.80, 129.7, 129.6, 129.5, 129.3, 128.8, 128.7, 128.38, 128.36, 128.32, 128.2, 96.2 (d, J = 5.0 Hz), 79.4, 73.1, 72.4, 72.3, 69.4, 65.3 (d, J =5.0 Hz), 62.9, 54.8 (d, J =5.0 Hz), 52.0, 28.4. **HRMS** (m/z): $[M-Et_3NH^++Na^++H^+]^+$calculated for $C_{43}H_{44}NO_{17}PNa^+$: 900.2245 Found 900.2272.

Triethylammonium (2,3,4,6-tetra-*O*-benzoyl-β-D-glucopyranos-1-yl) (2',3'-*O*-isopropylideneuridin-5'-yl) phosphate (5e)

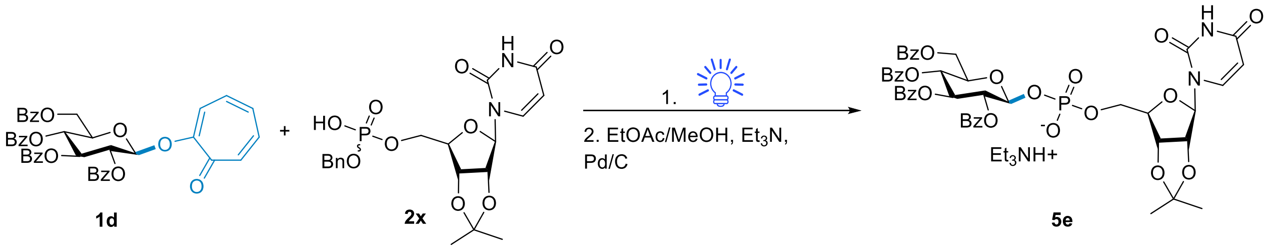

The mixture of the glycosyl donor **1d** (42.0 mg, 0.0600 mmol), acceptor **2x** (27.1 mg, 0.0500 mmol) and NaOTf (4.3 mg, 0.025 mmol) in dry $ClCH_2CH_2Cl$ (2.0 mL), was irradiated by blue LEDs at ambient temperature for 8 h. Upon completion, Et_3N (0.1 mL) was added to the reaction. The solvent was removed in vacuo, and the residue was loaded to silica gel column which was neutralized by using hexanes/Et_3N (100/1) and purified by eluting with solvents (petroleum ether/EtOAc= 1/2) to give an intermediate product benzyl phosphate (25.8 mg, 50%). To a solution of above product in EtOAc/MeOH (2.0 mL/2.0 mL) was added Et_3N (40.0 μL) and Pd/C (30.0 mg). Then, the mixture was stirred under an atmosphere of H_2 at room temperature for 0.5 h, until TLC showed the completion of hydrogenolysis. After filtration through glass funnel filter, the filtrate was concentrated to afford the residue. The residue was loaded to silica gel column which was neutralized by using hexanes/Et_3N (100/1) and purified by eluting with solvents (DCM/MeOH = 15/1) to afford **5e** as a white solid (quantitative yield). $[\alpha]_D^{25}$: -3.07 (c: 0.13 $CHCl_3$). ^{1}**H NMR** (400 MHz, $CDCl_3$) δ 8.08 - 8.00 (m, 2H), 8.00 - 7.94 (m, 2H), 7.94 - 7.87 (m, 2H), 7.85 - 7.79 (m, 2H), 7.77 (d, J = 8.1 Hz, 1H), 7.60 - 7.46 (m, 3H), 7.46 - 7.30 (m, 7H), 7.29 - 7.27 (m, 1H), 7.26 - 7.23 (m, 1H), 5.90 (t, J = 9.6 Hz, 1H), 5.85 (d, J = 3.4 Hz, 1H), 5.79 - 5.67 (m, 3H), 5.56 (dd, J = 9.8, 7.9 Hz, 1H), 4.76 - 4.64 (m, 2H), 4.58 (dd, J = 6.1, 3.3 Hz, 1H), 4.41 (dd, J = 12.3, 4.1 Hz, 1H), 4.30 - 4.26 (m, 1H), 4.15 - 4.02 (m, 2H), 3.97 - 3.92 (m, 1H), 1.51 (s, 3H), 1.28 (s, 3H). ^{31}**P NMR** (162 MHz, $CDCl_3$) δ -3.54. ^{13}**C NMR** (101 MHz, $CDCl_3$) δ 166.0, 165.6, 165.15, 165.13, 163.0, 150.0, 141.1, 133.4, 133.3, 133.2, 133.1, 129.9, 129.8, 129.75, 129.72, 129.5, 129.1, 128.8, 128.7, 128.46, 128.42, 128.40, 128.2, 113.7, 102.0, 96.3 (d, J = 5.0 Hz), 91.7, 84.9, 84.8, 80.6, 73.0, 72.5 (d, J = 9.0 Hz), 72.4, 69.2, 65.4 (d, J = 5.0 Hz), 62.7, 27.2, 25.3. **HRMS** (m/z): $[M-Et_3NH^++Na^++H^+]^+$calculated for $C_{46}H_{43}N_2O_{18}PNa^+$: 965.2146 Found 965.2173.

2,3,4,6-Tetra-*O*-benzyl-α-D-glucopyranosyl(diphenyl)phosphate (5f)

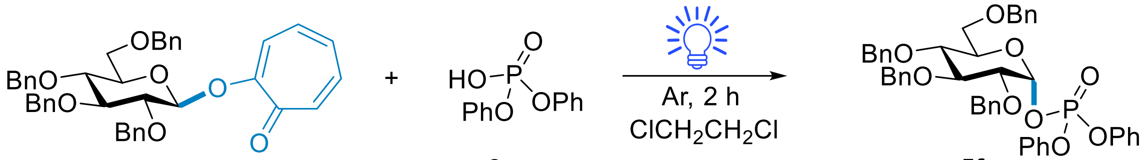

The glycosyl donor **1b** (38.7 mg, 0.0600 mmol) and acceptor **2p** (12.5 mg, 0.0500 mmol) were dissolved in dry $ClCH_2CH_2Cl$ (2.0 mL), and the mixture was irradiated by blue LEDs at ambient temperature for 2 h. Upon completion, Et_3N (0.1 mL) was added to the reaction. The solvent was removed in vacuo and the residue was loaded to silica gel column which was neutralized by using hexanes/Et_3N (100/1) and purified by eluting with solvents (petroleum ether/EtOAc = 8/1) to give the product **5f** (35.0 mg, 91%, α/β > 20/1) as a yellow solid. For the α anomer, **^{1}H NMR** (400 MHz, $CDCl_3$) δ 7.32 - 7.26 (m, 17H), 7.26 - 7.08 (m, 13H), 6.06 (dd, *J* = 6.7, 3.1 Hz, 1H), 4.89 (d, *J* = 10.9 Hz, 1H), 4.83 - 4.70 (m, 3H), 4.63 (d, *J* = 11.5 Hz, 1H), 4.54 (d, *J* = 12.1 Hz, 1H), 4.48 - 4.36 (m, 2H), 3.93 - 3.85 (m, 1H), 3.80 - 3.68 (m, 2H), 3.65 - 3.60 (m, 2H), 3.35 (d, *J* = 10.9 Hz, 1H). **^{31}P NMR** (162 MHz, $CDCl_3$) δ -13.30. The ^{1} H NMR data coincide with the reported data[21].

2,3,4,6-Tetra-*O*-benzyl-α-D-glucopyranosyl(dibenzyl)phosphate (5g)

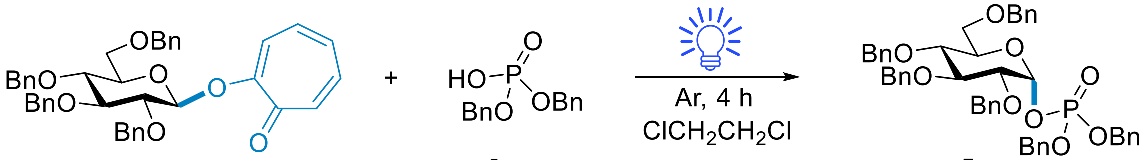

The glycosyl donor **1b** (38.7 mg, 0.0600 mmol) and acceptor **2q** (13.9 mg, 0.0500 mmol) were dissolved in dry $ClCH_2CH_2Cl$ (2.0 mL), and the mixture was irradiated by blue LEDs at ambient temperature for 4 h. Upon completion, Et_3N (0.1 mL) was added to the reaction. The solvent was removed in vacuo, and the residue was loaded to silica gel column which was neutralized by using hexanes/Et_3N (100/1) and purified by eluting with solvents (petroleum ether/EtOAc = 8/1) to give the product **5g** (31.2 mg, 69%, α/β > 20:1) as a yellow oil. For the α anomer, **^{1}H NMR** (400 MHz, $CDCl_3$) δ 7.36 - 7.26 (m, 25H), 7.24 - 7.23 (m, 3H), 7.14 - 7.11 (m, 2H), 5.96 (dd, *J* = 6.8, 3.3 Hz, 1H), 5.10 - 5.00 (m, 4H), 4.91 (d, *J* = 11.0 Hz, 1H), 4.82 (d, *J* = 10.8 Hz, 1H), 4.78 (d, *J* = 11.1 Hz, 2H), 4.67 (d, *J* = 11.3 Hz, 1H), 4.54 (d, *J* = 12.1 Hz, 1H), 4.48 (d, *J* = 10.7 Hz, 1H), 4.41 (d, *J* = 12.1 Hz, 1H), 3.93 - 3.89 (m, 2H), 3.71 (t, *J* = 9.6 Hz, 1H), 3.67 - 3.61 (m, 2H), 3.47 (dd, *J* = 10.9, 1.9 Hz, 1H). **^{31}P NMR** (162 MHz, $CDCl_3$) δ -2.28. The ^{1}H NMR data coincide with the reported data[9].

2,3,4,6-Tetra-*O*-benzyl-α-D-galactopyranosyl(diphenyl)phosphate (5h)

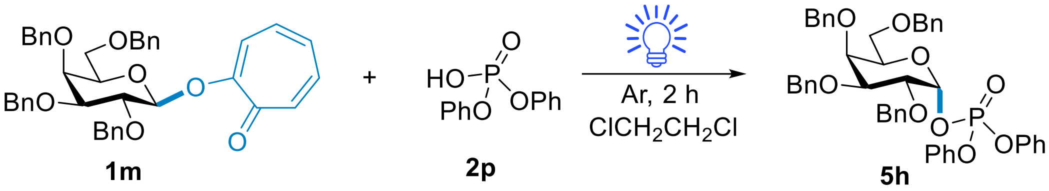

The glycosyl donor **1m** (38.7 mg, 0.0600 mmol) and acceptor **2p** (12.5 mg, 0.0500 mmol) were dissolved in dry $ClCH_2CH_2Cl$ (2.0 mL), and the mixture was irradiated by blue LEDs at ambient temperature for 2 h. Upon completion, Et_3N (0.1 mL) was added to the reaction. The solvent was removed in vacuo, and the residue was loaded to silica gel column which was neutralized by using hexanes/Et_3N (100/1) and purified by eluting with solvents (petroleum ether/EtOAc = 8/1) to give the product **5h** (32.6 mg, 85%, α/β = 13/1) as a yellow oil. For the α anomer, **^{1}H NMR** (400 MHz, $CDCl_3$) δ 7.38 - 7.26 (m, 19H), 7.26 - 7.07 (m, 11H), 6.06 (dd, J = 6.2, 3.3 Hz, 1H), 4.92 (d, J = 11.3 Hz, 1H), 4.80 - 4.64 (m, 4H), 4.55 (d, J = 11.3 Hz, 1H), 4.36 (s, 2H), 4.14 - 4.10 (m, 1H), 3.98 - 3.91 (m, 2H), 3.83 (dd, J = 10.0, 2.7 Hz, 1H), 3.52 (t, J = 8.6 Hz, 1H), 3.28 (dd, J = 8.9, 5.3 Hz, 1H). **^{31}P NMR** (162 MHz, $CDCl_3$) δ -13.23. The ^{1}H NMR data coincide with the reported data[9].

2,3,4,6-Tetra-*O*-benzyl-α-D-mannopyranosyl(diphenyl)phosphate (5i)

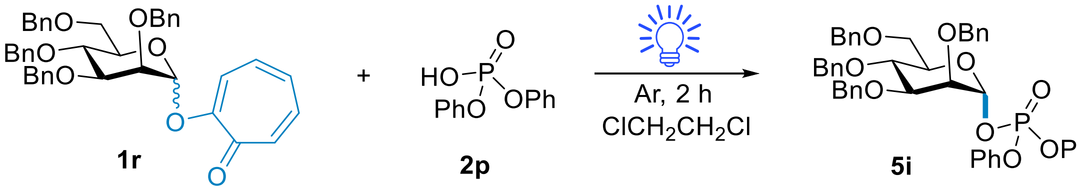

The glycosyl donor **1r** (38.7 mg, 0.0600 mmol) and acceptor **2p** (12.5 mg, 0.0500 mmol) were dissolved in dry $ClCH_2CH_2Cl$ (2.0 mL), and the mixture was irradiated by blue LEDs at ambient temperature for 2 h. Upon completion, Et_3N (0.1 mL) was added to the reaction. The solvent was removed in vacuo, and the residue was loaded to silica gel column which was neutralized by using hexanes/Et_3N (100/1) and purified by eluting with solvents (petroleum ether/EtOAc = 8/1) to give the product **5i** (30.1 mg, 78%, α/β > 20/1) as a colorless oil. For the α anomer, **^{1}H NMR** (400 MHz, $CDCl_3$) δ 7.37 - 7.26 (m, 22H), 7.22 - 7.09 (m, 8H), 5.99 (dd, J = 6.3, 2.0 Hz, 1H), 4.85 (d, J = 10.8 Hz, 1H), 4.69 (s, 2H), 4.63 (d, J = 12.0 Hz, 1H), 4.54 - 4.40 (m, 4H), 4.10 (t, J = 9.7 Hz, 1H), 3.89 - 3.78 (m, 2H), 3.78 - 3.70 (m, 2H), 3.53 (dd, J = 11.2, 1.8 Hz, 1H). **^{31}P NMR** (162 MHz, $CDCl_3$) δ -14.00. The ^{1}H NMR data coincide with the reported data[9].

2,3,4-Tri-*O*-benzyl-α-D-xylopyranosyl(dibenzyl)phosphate (5j)

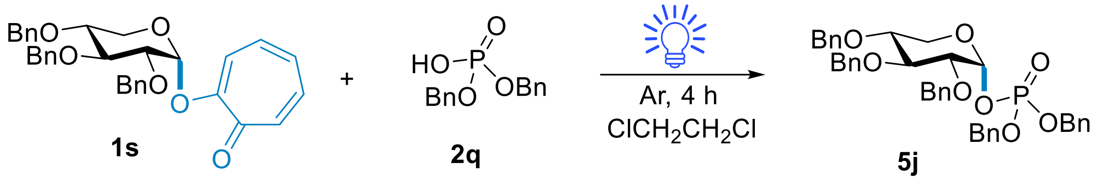

The glycosyl donor **1s** (31.5 mg, 0.0600 mmol) and acceptor **2q** (13.9 mg, 0.0500 mmol) were dissolved in dry $ClCH_2CH_2Cl$ (2.0 mL), and the mixture was irradiated by blue LEDs at ambient temperature for 4 h. Upon completion, Et_3N (0.1 mL) was added to the reaction. The solvent was removed in vacuo, and the residue was loaded to silica gel column which was neutralized by using hexanes/Et_3N (100/1) and purified by eluting with solvents (petroleum ether/EtOAc = 8/1) to give the product **5j** (20.3 mg, 60%, α/β = 9/1) as a colorless oil. For the α anomer, **^{1}H NMR** (400 MHz, $CDCl_3$) δ 7.39 - 7.26 (m, 19H), 7.25 - 7.24 (m, 6H), 5.85 (dd, J = 6.7, 3.2 Hz, 1H), 5.08 - 5.00 (m, 4H), 4.90 - 4.80 (m, 2H), 4.77 - 4.66 (m, 3H), 4.60 (d, J = 11.8 Hz, 1H), 3.83 (t, J = 8.9 Hz, 1H), 3.72 - 3.57 (m, 3H), 3.54 - 3.50 (m, 1H). **^{31}P NMR** (162 MHz, $CDCl_3$) δ -2.25. The ^{1}H NMR data coincide with the reported data[9].

2-Azido-3,4,6-tri-*O*-benzyl-2-deoxy-α-D-glucopyranosyl(diphenyl)phosphate (5k)

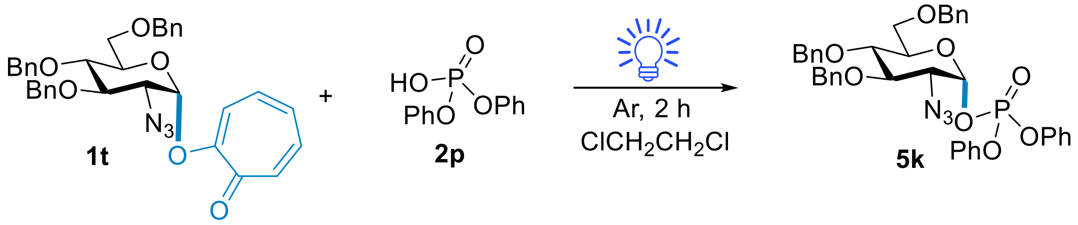

The glycosyl donor **1t** (34.8 mg, 0.0600 mmol) and acceptor **2p** (12.5 mg, 0.0500 mmol) were dissolved in dry $ClCH_2CH_2Cl$ (2.0 mL), and the mixture was irradiated by blue LEDs at ambient temperature for 2 h. Upon completion, Et_3N (0.1 mL) was added to the reaction. The solvent was removed in vacuo, and the residue was loaded to silica gel column which was neutralized by using hexanes/Et_3N (100/1) and purified by eluting with solvents (petroleum ether/EtOAc = 8/1) to give the product **5k** (20.1 mg, 57%, α/β = 6/1) as a pale-yellow oil. For the α anomer, **^{1}H NMR** (400 MHz, $CDCl_3$) δ 7.40 - 7.26 (m, 20H), 7.25 - 7.14 (m, 5H), 5.98 (dd, J = 6.2, 3.3 Hz, 1H), 4.94 - 4.74 (m, 3H), 4.60 - 4.48 (m, 2H), 4.43 (d, J = 12.1 Hz, 1H), 3.94 - 3.79 (m, 3H), 3.72 - 3.56 (m, 2H), 3.35 (d, J = 11.1 Hz, 1H). **^{31}P NMR** (162 MHz, $CDCl_3$) δ -10.36. The ^{1}H NMR data coincide with the reported data[22].

Triethylammonium bis(2,3,4,6-tetra-*O*-benzyl-α-D-glucopyranos-1-yl)phosphate (5l)

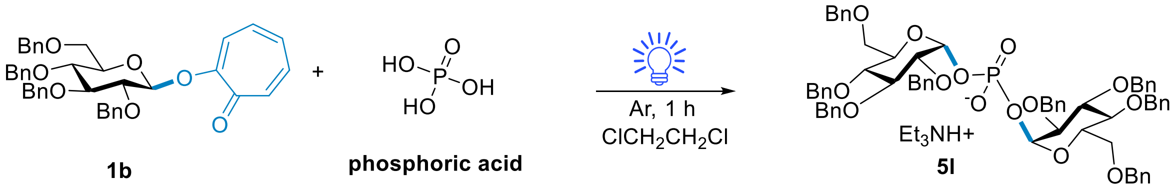

The glycosyl donor **1b** (77.2 mg, 0.120 mmol) and phosphoric acid (2.90 uL, 0.0500 mmol) were dissolved in dry $ClCH_2CH_2Cl$ (2.0 mL), and the mixture was irradiated by blue LEDs at ambient temperature for 0.5 h. Upon completion, Et_3N (0.1 mL) was added to the reaction. The solvent was removed in vacuo, and the residue was loaded to silica gel column which was neutralized by using hexanes/Et_3N (100/1) and purified by eluting with solvents (DCM/MeOH = 20/1) to afford **5l** (37.3 mg, 60 %, α/β = 8/1) as a colorless oil. For the α anomer, $[\alpha]_D^{25}$: 43.81 (*c*: 0.63 $CHCl_3$). **^{1}H NMR** (600 MHz, $CDCl_3$) δ 7.43 - 7.39 (m, 2H), 7.29 - 7.19 (m, 16H), 7.17 - 7.14 (m, 2H), 5.97 (dd, *J* = 7.5, 3.2 Hz, 1H), 4.93 - 4.88 (m, 2H), 4.83 (d, *J* = 11.1 Hz, 1H), 4.73 (d, *J* = 10.9 Hz, 1H), 4.59 (d, *J* = 11.7 Hz, 1H), 4.49 (t, *J* = 11.8 Hz, 2H), 4.29 (d, *J* = 12.1 Hz, 1H), 4.13 (dt, *J* = 10.1, 2.5 Hz, 1H), 4.03 (t, *J* = 9.3 Hz, 1H), 3.77 - 3.73 (m, 1H), 3.73 - 3.68 (m, 2H), 3.60 (dt, *J* = 9.6, 3.0 Hz, 1H). **^{31}P NMR** (162 MHz, $CDCl_3$) δ -2.54. **^{13}C NMR** (151 MHz, $CDCl_3$) δ 139.0, 138.8, 138.7, 138.1, 128.26, 128.21, 128.1, 127.94, 127.89, 127.86, 127.5, 127.45, 127.40, 127.3, 127.2, 92.9 (d, *J* = 4 Hz), 81.6, 80.0 (d, *J* = 6 Hz), 77.4, 75.4, 74.6, 73.2, 71.7, 71.1, 68.4. **HRMS** (m/z): $[M-Et_3NH^+]^-$ calculated for $C_{68}H_{70}O_{14}P^-$: 1141.4509 Found :1141.4524.

Triethylammonium (2,3,4,6-tetra-*O*-benzyl-α-D-glucopyranos-1-yl)(methyl 2,3,4-tri-*O*-benzyl-α-D-glucopyranosid-6-yl)phosphate (5m)

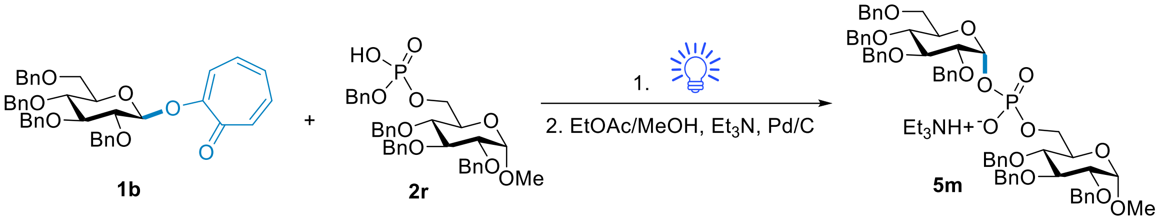

The mixture of the glycosyl donor **1b** (38.7 mg, 0.0600 mmol), acceptor **2r** (31.7 mg, 0.0500 mmol) and $NaSO_4Me$ (2.0 mg, 0.015 mmol) in dry $ClCH_2CH_2Cl$ (2.0 mL), was irradiated by blue LEDs at ambient temperature for 8 h. Upon completion, Et_3N (0.1 mL) was added to the reaction. The solvent was removed in vacuo, and the residue was loaded to silica gel column which was neutralized by using hexanes/Et_3N (100/1) and purified by eluting with solvents (petroleum ether/EtOAc = 8/1) to give an intermediate product benzyl phosphate (31.7 mg, 55%, α/β = 9/1). To a solution of above product in EtOAc/MeOH (2.0 mL/2.0 mL) was added Et_3N (40.0 μL) and Pd/C (30.0 mg). Then, the mixture was stirred under an atmosphere of H_2 at room temperature for 0.5 h, until TLC showed the completion of hydrogenolysis. After filtration through glass funnel filter, the filtrate was concentrated to afford the oil. The oil was loaded to silica gel column which was neutralized by using hexanes/Et_3N (100/1) and purified

by eluting with solvents (DCM/MeOH = 30/1) to afford **5m** as a pale-yellow solid (quantitative yield). For the α anomer, $[\alpha]_D^{25}$: 13.67 (*c*: 0.30 $CHCl_3$). **^{1}H NMR** (400 MHz, $CDCl_3$) δ 7.43 - 7.26 (m, 20H), 7.26 - 7.12 (m, 15H), 5.92 (dd, *J* = 8.0, 3.3 Hz, 1H), 4.97 - 4.69 (m, 9H), 4.61 (d, *J* = 11.8 Hz, 2H), 4.52 - 4.47 (m, 3H), 4.35 (d, *J* = 12.2 Hz, 1H), 4.20 - 4.18 (m, 2H), 4.07 - 3.92 (m, 3H), 3.73 - 3.71 (m, 2H), 3.68 - 3.55 (m, 4H), 3.45 (dd, *J* = 9.8, 3.5 Hz, 1H), 3.29 (s, 3H). **^{13}C NMR** (101 MHz, $CDCl_3$) δ 139.0, 138.9, 138.7, 138.6, 138.29, 138.20, 128.37, 128.33, 128.27, 128.25, 128.20, 128.1, 128.0, 127.9, 127.86, 127.81, 127.7, 127.53, 127.51, 127.4, 127.3, 97.8, 92.9 (d, *J* = 6.0 Hz), 82.0, 81.5, 80.1, 79.7, 77.7, 77.4, 75.5, 75.3, 74.8, 74.6, 73.3, 73.2, 72.0, 71.2, 70.1 (d, *J* = 10.0 Hz), 68.5, 64.3, 55.0. **^{31}P NMR** (162 MHz, $CDCl_3$) δ -1.12. **HRMS** (m/z): $[M-Et_3NH^++Na^++H^+]^+$calculated for $C_{62}H_{67}O_{14}PNa^+$: 1089.4166 Found 1089.4161.

Triethylammonium (2,3,4,6-tetra-*O*-benzyl-α-D-glucopyranos-1-yl)(1,2:3,4-di-*O*-isopropylidene-α-D-galactopyranosid-6-yl)phosphate (5n)

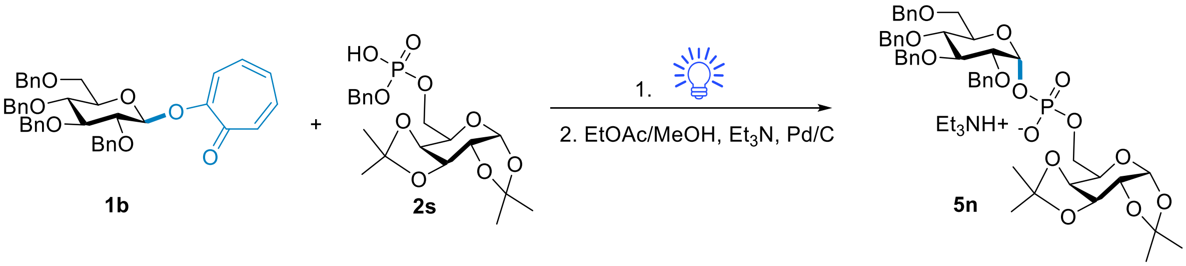

1b **2s** **5n**

The mixture of the glycosyl donor **1b** (38.7 mg, 0.0600 mmol), acceptor **2s** (21.5 mg, 0.0500 mmol) and $NaSO_4Me$ (2.0 mg, 0.015 mmol) in dry $ClCH_2CH_2Cl$ (2.0 mL), was irradiated by blue LEDs at ambient temperature for 1.5 h. Upon completion, Et_3N (0.1 mL) was added to the reaction. The solvent was removed in vacuo, and the residue was loaded to silica gel column which was neutralized by using hexanes/Et_3N (100/1) and purified by eluting with solvents (petroleum ether/EtOAc = 6/1) to give an intermediate product benzyl phosphate (29.8 mg, 63%, α/β = 7/1). To a solution of above product in EtOAc/MeOH (2.0 mL/2.0 mL) was added Et_3N (40.0 μL) and Pd/C (30.0 mg). Then, the mixture was stirred under an atmosphere of H_2 at room temperature for 0.5 h, until TLC showed the completion of hydrogenolysis. After filtration through glass funnel filter, the filtrate was concentrated to afford the oil. The oil was loaded to silica gel column which was neutralized by using hexanes/Et_3N (100/1) and purified by eluting with solvents (DCM/MeOH = 30/1) to afford **5n** as a pale-yellow solid (quantitative yield). For the α anomer, **^{1}H NMR** (400 MHz, $CDCl_3$) δ 7.43 - 7.41 (m, 2H), 7.34 - 7.25 (m, 16H), 7.20 - 7.15 (m, 2H), 5.92 (dd, *J* = 8.0, 3.3 Hz, 1H), 5.50 (d, *J* = 5.0 Hz, 1H), 4.97 - 4.90 (m, 2H), 4.86 (d, *J* = 11.2 Hz, 1H), 4.79 (d, *J* = 10.9 Hz, 1H), 4.68 - 4.60 (m, 2H), 4.56 - 4.50 (m, 2H), 4.47 (d, *J* = 12.2 Hz, 1H), 4.28 - 4.23 (m, 2H), 4.16 - 4.00 (m, 5H), 3.81 - 3.72 (m, 2H), 3.71 - 3.67 (m, 1H), 3.65 - 3.59 (m, 1H), 1.51 (s, 3H), 1.39 (s, 3H), 1.32 (s, 3H), 1.25 (s, 3H). **^{31}P NMR** (162 MHz, $CDCl_3$) δ -1.17. The ^{1}H NMR data coincide with the reported data[9].

Triethylammonium (2,3,4,6-tetra-*O*-benzyl-α-D-glucopyranos-1-yl)(methyl 3,4,6-tri-*O*-

benzyl-α-D-glucopyranosid-2-yl)phosphate (5o)

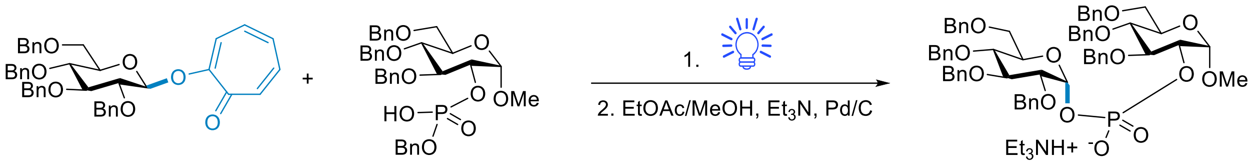

The glycosyl donor **1b** (38.7 mg, 0.0600 mmol) and acceptor **2u** (31.7 mg, 0.0500 mmol) were dissolved in dry $ClCH_2CH_2Cl$ (2.0 mL), and the mixture was irradiated by blue LEDs at 100°C for 14 h. Upon completion, Et_3N (0.1 mL) was added to the reaction. The solvent was removed in vacuo, and the residue was loaded to silica gel column which was neutralized by using hexanes/Et_3N (100/1) and purified by eluting with solvents (petroleum ether/EtOAc = 6/1) to give an intermediate product benzyl phosphate (41.0mg, 71%, α/β = 10/1). To a solution of above product in EtOAc/MeOH (2.0 mL/2.0 mL) was added Et_3N (40.0 μL) and Pd/C (30.0 mg). Then, the mixture was stirred under an atmosphere of H_2 at room temperature for 0.5 h, until TLC showed the completion of hydrogenolysis. After filtration through glass funnel filter, the filtrate was concentrated to afford the residue. The residue was loaded to silica gel column which was neutralized by using hexanes/Et_3N (100/1) and purified by eluting with solvents (DCM/MeOH = 30/1) to afford **5o** as a white solid (quantitative yield). For the α anomer, $[\alpha]_D^{25}$: 40.00 (*c*: 0.23 $CHCl_3$). **^{1}H NMR** (400 MHz, $CDCl_3$) δ 7.42 - 7.27 (m, 12H), 7.26 - 7.06 (m, 23H), 5.98 (dd, *J* = 7.7, 3.3 Hz, 1H), 5.29 (d, *J* = 3.6 Hz, 1H), 5.09 (d, *J* = 11.2 Hz, 1H), 4.90 (d, *J* = 12.0 Hz, 2H), 4.81 (d, *J* = 11.2 Hz, 1H), 4.74 (dd, *J* = 10.9, 4.0 Hz, 2H), 4.69 - 4.56 (m, 3H), 4.55 - 4.46 (m, 3H), 4.42 - 4.38 (m, 2H), 4.35 - 4.30 (m, 1H), 4.16 - 4.12 (m, 1H), 4.00 - 3.92 (m, 2H), 3.81 - 3.69 (m, 2H), 3.69 - 3.56 (m, 5H), 3.46 (t, *J* = 9.5 Hz, 1H), 3.37 (s, 3H). **^{31}P NMR** (162 MHz, $CDCl_3$) δ -1.93. **^{13}C NMR** (101 MHz, $CDCl_3$) δ 139.3, 139.0, 138.9, 138.5, 138.48, 138.40, 138.2, 128.3, 128.29, 128.27, 128.24, 128.15, 128.14, 128.11, 128.0, 127.9, 127.8, 127.7, 127.54, 127.52, 127.49, 127.43, 127.3, 127.2, 127.1, 98.7, 93.3 (d, *J* = 6 Hz), 81.7, 81.6, 79.9 (d, *J* = 8 Hz), 77.8, 77.5, 75.4, 75.37, 75.30, 74.9, 74.6, 73.4, 73.3, 72.0, 70.9, 70.1, 69.1, 68.5, 55.0. **HRMS** (m/z): $[M-Et_3NH^++Na^++H^+]^+$calculated for $C_{62}H_{67}O_{14}PNa^+$:1089.4166 Found:1089.4175.

Triethylammonium (2,3,4,6-tetra-*O*-benzyl-α-D-glucopyranos-1-yl)(methyl 2,3,6-tri-*O*-benzyl-α-D-glucopyranosid-4-yl)phosphate (5p)

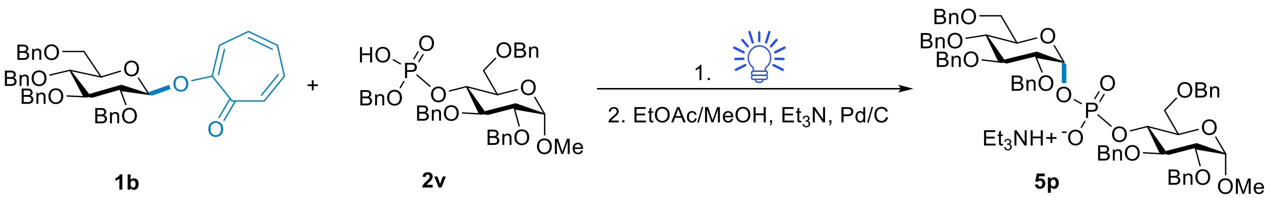

The mixture of the glycosyl donor **1b** (38.7 mg, 0.0600 mmol), acceptor **2v** (31.7 mg, 0.0500 mmol) and $NaSO_4Me$ (2.0 mg, 0.015 mmol) in dry $ClCH_2CH_2Cl$ (2.0 mL), was irradiated by blue LEDs at ambient temperature for 2 h. Upon completion, Et_3N (0.1 mL) was added to the reaction. The solvent was removed in vacuo, and the residue was loaded to silica gel column which was neutralized by using hexanes/Et_3N (100/1) and purified by eluting with solvents (petroleum ether/EtOAc = 6/1) to give an intermediate product benzyl phosphate (23.0 mg, 40%, α/β > 20/1). To a solution of above product in EtOAc/MeOH (2.0 mL/2.0 mL) was added Et_3N (40.0 µL) and Pd/C (30.0 mg). Then, the mixture was stirred under an atmosphere of H_2 at room temperature for 0.5 h, until TLC showed the completion of hydrogenolysis. After filtration through glass funnel filter, the filtrate was concentrated to afford the oil. The oil was loaded to silica gel column which was neutralized by using hexanes/Et_3N (100/1) and purified by eluting with solvents (DCM/MeOH = 30/1) to afford **5p** as a white solid (quantitative yield). For the α anomer, **^{1}H NMR** (400 MHz, $CDCl_3$) δ 7.49 (d, *J* = 7.5 Hz, 2H), 7.37 (d, *J* = 7.3 Hz, 2H), 7.30 - 7.12 (m, 31H), 5.93 (dd, *J* = 7.9, 3.2 Hz, 1H), 5.19 (d, *J* = 11.3 Hz, 1H), 4.89 - 4.76 (m, 4H), 4.72 - 4.63 (m, 2H), 4.61 - 4.46 (m, 7H), 4.44 - 4.31 (m, 2H), 4.15 - 4.07 (m, 2H), 3.99 - 3.90 (m, 2H), 3.89 - 3.77 (m, 2H), 3.75 - 3.55 (m, 4H), 3.45 (dd, *J* = 9.6, 3.7 Hz, 1H), 3.34 (s, 3H). **^{31}P NMR** (162 MHz, $CDCl_3$) δ -2.46. The ^{1}H NMR data coincide with the reported data[9].

Triethylammonium (2,3,4,6-tetra-*O*-benzyl-α-D-glucopyranos-1-yl)(methyl *N*-(*tert*-butoxycarbonyl)-L-serin-3-yl)phosphate (5q)

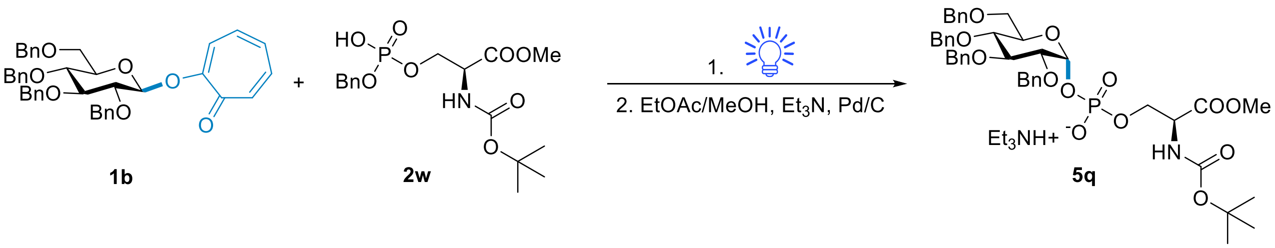

The glycosyl donor **1b** (38.7 mg, 0.0600 mmol) and acceptor **2w** (19.5 mg, 0.0500 mmol) were dissolved in dry $ClCH_2CH_2Cl$ (2.0 mL), and the mixture was irradiated by blue LEDs at 100°C for 10 h. Upon completion, Et_3N (0.1 mL) was added to the reaction. The solvent was removed in vacuo, and the residue was loaded to silica gel column which was neutralized by using hexanes/Et_3N (100/1) and purified by eluting with solvents (petroleum ether/EtOAc = 6/1) to give an intermediate product benzyl phosphate (26.4 mg, 58%, α/β = 15/1). To a solution of above product in EtOAc/MeOH (2.0 mL/2.0 mL) was added Et_3N (40.0 µL) and Pd/C (30.0 mg). Then, the mixture was stirred under an atmosphere of H_2 at room temperature for 0.5 h, until TLC showed the completion of hydrogenolysis. After filtration through glass funnel filter, the filtrate was concentrated to afford the residue. The residue was loaded to silica gel column which was neutralized by using hexanes/Et_3N (100/1) and purified by eluting with solvents (DCM/MeOH = 40/1) to afford **5q** as a pale-yellow solid (quantitative yield). For the α anomer, $[\alpha]_D^{25}$: 13.48 (*c*: 0.23 $CHCl_3$). **^{1}H NMR** (400 MHz, $CDCl_3$) δ 7.59 - 7.27 (m, 15H), 7.26 - 7.13 (m, 5H),

6.79 (d, J = 5.3 Hz, 1H), 5.84 (dd, J = 8.2, 3.2 Hz, 1H), 4.95 (d, J = 10.9 Hz, 1H), 4.90 - 4.76 (m, 3H), 4.69 - 4.55 (m, 2H), 4.52 - 4.45 (m, 2H), 4.31 - 4.14 (m, 3H), 4.11 - 3.98 (m, 2H), 3.72 (dd, J = 10.8, 3.6 Hz, 1H), 3.68 (s, 3H), 3.67 - 3.49 (m, 3H), 1.41 (s, 9H). **^{31}P NMR** (162 MHz, $CDCl_3$) δ -0.83. **^{13}C NMR** (101 MHz, $CDCl_3$) δ 170.9, 155.9, 138.9, 138.8, 138.6, 138.5, 138.48, 138.46, 138.45, 138.16, 138.10, 128.3, 128.27, 128.24, 128.22, 128.1, 127.94, 127.92, 127.8, 127.57, 127.55, 127.54, 127.49, 127.46, 127.44, 127.40, 93.1 (d, J = 6 Hz), 81.4, 79.8, 79.2, 77.5, 75.4, 74.7, 73.4, 72.1, 71.4 (d, J = 3 Hz), 68.7 (d, J = 9 Hz), 65.1 (d, J = 3 Hz), 55.1, 52.1 (d, J = 6 Hz), 28.4. **HRMS** (m/z): $[M\text{-}Et_3NH^+ + Na^+ + H^+]^+$calculated for $C_{43}H_{52}NO_{13}PNa^+$: 844.3074 Found 844.3082.

Triethylammonium (2,3,4,6-tetra-*O*-benzyl-α-D-glucopyranos-1-yl)(2',3'-*O*-isopropylideneuridin-5'-yl)phosphate (5r)

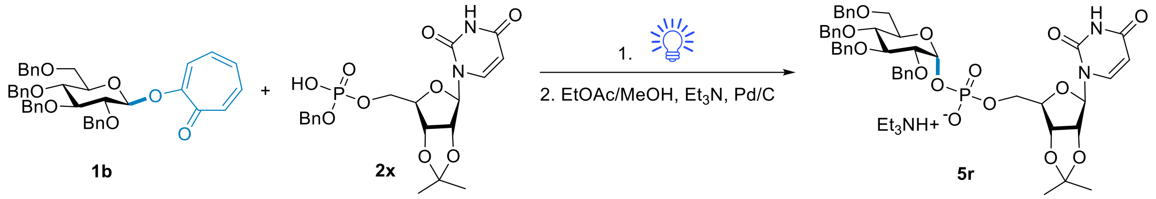

The glycosyl donor **1b** (38.7 mg, 0.0600 mmol) and acceptor **2x** (22.7 mg, 0.0500 mmol) were dissolved in dry $ClCH_2CH_2Cl$ (2.0 mL), and the mixture was irradiated by blue LEDs at 100°C for 12 h. Upon completion, Et_3N (0.1 mL) was added to the reaction. The solvent was removed in vacuo, and the residue was loaded to silica gel column which was neutralized by using hexanes/Et_3N (100/1) and purified by eluting with solvents (DCM/MeOH = 60/1) to give an intermediate product benzyl phosphate (24.3 mg, 50%, α/β = 10/1). To a solution of above product in EtOAc/MeOH (2.0 mL/2.0 mL) was added Et_3N (40.0 μL) and Pd/C (30.0 mg). Then, the mixture was stirred under an atmosphere of H_2 at room temperature for 0.5 h, until TLC showed the completion of hydrogenolysis. After filtration through glass funnel filter, the filtrate was concentrated to afford the residue. The residue was loaded to silica gel column which was neutralized by using hexanes/Et_3N (100/1) and purified by eluting with solvents (DCM/MeOH = 20/1) to afford **5r** as a pale-yellow solid (quantitative yield). For the α anomer, $[\alpha]_D^{25}$: 12.50 (*c*: 0.04 MeOH). **^{1}H NMR** (400 MHz, CD_3OD) δ 7.87 (d, J = 8.0 Hz, 1H), 7.41 - 7.38 (m, 2H), 7.35 - 7.11 (m, 18H), 5.91 (d, J = 3.3 Hz, 1H), 5.80 (dd, J = 8.0, 3.3 Hz, 1H), 5.74 (d, J = 8.1 Hz, 1H), 4.90 (d, J = 11.2 Hz, 2H), 4.81 - 4.71 (m, 4H), 4.64 (d, J = 11.5 Hz, 1H), 4.55 - 4.48 (m, 2H), 4.43 (d, J = 12.0 Hz, 1H), 4.27 - 4.24 (m, 1H), 4.16 - 4.04 (m, 2H), 4.03 - 3.90 (m, 2H), 3.69 - 3.52 (m, 4H), 1.51 (s, 3H), 1.29 (s, 3H). **^{31}P NMR** (162 MHz, CD_3OD) δ -2.08. **^{13}C NMR** (101 MHz, CD_3OD) δ 164.7, 150.7, 141.8, 138.7, 138.4, 138.3, 138.0, 127.98, 127.95, 127.88, 127.79, 127.76, 127.5, 127.4, 127.3, 127.2, 127.18, 127.14, 113.5, 101.6, 93.2 (d, J = 6 Hz), 91.9, 85.1 (d, J = 9 Hz), 84.4, 81.2, 80.9, 79.7 (d, J = 7 Hz), 77.3, 75.0, 74.4, 73.0, 72.0, 71.6, 68.4, 65.2 (d, J = 5 Hz), 26.2, 24.2. **HRMS** (m/z): $[M\text{-}Et_3NH^+ + Na^+ + H^+]^+$calculated for $C_{46}H_{51}N_2O_{14}PNa^+$:909.2976 Found 909.2976.

1-*N*-(2,3,4,6-Tetra-*O*-benzoyl-β-D-galactopyranosyl)-uracil (6a)

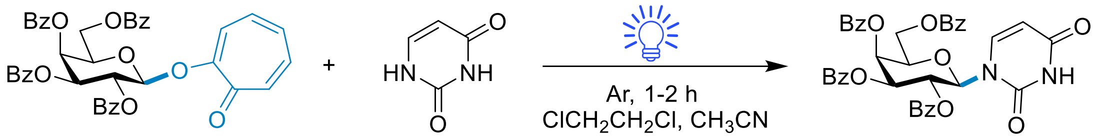

1c **uracil** **6a**

To a solution of uracil (5.6 mg, 0.050 mmol) in anhydrous CH_3CN (1.0 mL) was added BSTFA (53.1 uL, 0.200 mmol) under Ar atmosphere. The suspension was stirred at room temperature until it becomes a clear solution. Then the **1c** (42.0 mg, 0.0600 mmol) dissolved in $ClCH_2CH_2Cl$ (1.0 mL) and TMSOTf (0.90 uL, 0.0050 mmol) were added to the above solution through a syringe, the mixture was irradiated by blue LEDs at ambient temperature. The progress of the reaction was monitored by TLC (1-2 h). Upon completion, the reaction mixture was concentrated in vacuo. The residue was purified by flash column chromatography on silica gel (petroleum ether/EtOAc = 2/1) to obtain **6a** (32.8 mg, 95%) as a white solid. **^{1}H NMR** (400 MHz, $CDCl_3$) δ 8.78 (s, 1H), 8.06 - 7.98(m, 4H), 7.89 - 7.76 (m, 4H), 7.71 - 7.65 (m, 1H), 7.60 - 7.32 (m, 10H), 7.27 -7.23 (m, 2H), 6.26 (d, J = 9.1 Hz, 1H), 6.11 (d, J = 3.2 Hz, 1H), 6.00 - 5.80 (m, 3H), 4.71 - 4.55 (m, 2H), 4.50 - 4.38 (m, 1H). The ^{1}H NMR data coincide with the reported data[23].

5-Ethyl-1-*N*-(2,3,4,6-tetra-*O*-benzoyl-β-D-glucopyranosyl)-uracil (6b)

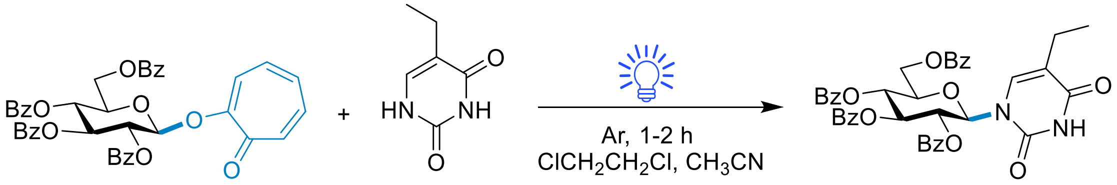

1d **5-ethyluracil** **6b**

To a solution of 5-ethyluracil (7.0 mg, 0.050 mmol) in anhydrous CH_3CN (1.0 mL) was added BSTFA (53.1 uL, 0.200 mmol) under Ar atmosphere. The suspension was stirred at room temperature until it becomes a clear solution. Then the **1d** (42.0 mg, 0.0600 mmol) dissolved in $ClCH_2CH_2Cl$ (1.0 mL) and TMSOTf (0.90 uL, 0.0050 mmol) were added to the above solution through a syringe, the mixture was irradiated by blue LEDs at ambient temperature. The progress of the reaction was monitored by TLC (1-2 h). Upon completion, the reaction mixture was concentrated in vacuo. The residue was purified by flash column chromatography on silica gel (petroleum ether/EtOAc = 2/1) to obtain **6b** (34.2 mg, 96%) as a white solid. $[\alpha]_D^{25}$: -0.65 (c: 0.31 $CHCl_3$). **^{1}H NMR** (400 MHz, $CDCl_3$) δ 8.60 (s, 1H), 8.09 - 7.99 (m, 2H), 7.97 - 7.89 (m, 2H), 7.89 - 7.77 (m, 4H), 7.61 - 7.26 (m, 12H), 7.23 - 7.22 (m, 1H), 6.28 (d, J = 9.5 Hz, 1H), 6.10 (t, J = 9.6 Hz, 1H), 5.79 (t, J = 9.8 Hz, 1H), 5.71 (t, J = 9.6 Hz, 1H), 4.68 (dd, J = 12.4, 2.7 Hz, 1H), 4.48 (dd, J = 12.4, 4.9 Hz, 1H), 4.42 - 4.37 (m, 1H), 2.44 - 2.27 (m, 2H), 1.14 (t, J = 7.4 Hz, 3H). **^{13}C NMR** (101 MHz, $CDCl_3$) δ 165.9, 165.4, 165.1, 162.5, 150.2, 133.8, 133.7, 133.6, 133.4,

133.2, 129.95, 129.91, 129.77, 129.72, 129.4, 128.55, 128.51, 128.4, 128.3, 128.0, 117.8, 80.6, 75.33, 73.0, 69.9, 68.9, 62.5, 20.1, 12.5. **HRMS** (ESI) Calculated for $C_{40}H_{38}N_3O_{11}$ $[M+NH_4]^+$: 736.2506 Found: 736.2512.

1-*N*-(2,3,5-Tri-*O*-benzoyl-α-D-arabinofuranosyl)-uracil (6c)

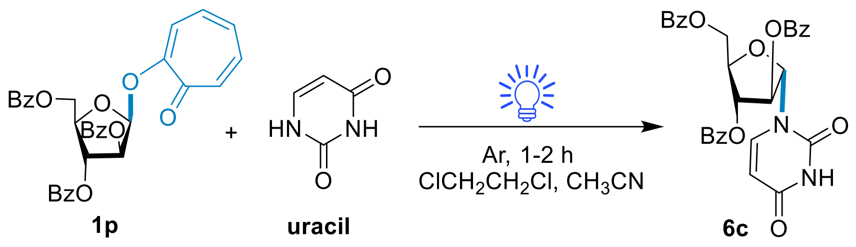

To a solution of uracil (5.6 mg, 0.050 mmol) in anhydrous CH_3CN (1.0 mL) was added BSTFA (53.1 uL, 0.200 mmol) under Ar atmosphere. The suspension was stirred at room temperature until it becomes a clear solution. Then the **1p** (34.0 mg, 0.0600 mmol) dissolved in $ClCH_2CH_2Cl$ (1.0 mL) and TMSOTf (0.90 uL, 0.0050 mmol) were added to the above solution through a syringe, the mixture was irradiated by blue LEDs at ambient temperature. The progress of the reaction was monitored by TLC (1-2 h). Upon completion, the reaction mixture was concentrated in vacuo. The residue was purified by flash column chromatography on silica gel (petroleum ether/EtOAc = 2/1) to obtain **6c** (26.1 mg, 94%) as a white solid. $[\alpha]_D^{25}$: -9.13 (*c*: 0.23 $CHCl_3$). **^{1}H NMR** (400 MHz, $CDCl_3$) δ 8.97 - 8.88 (m, 1H), 8.14 - 8.07 (m, 2H), 8.05 - 7.99 (m, 4H), 7.64 - 7.51 (m, 3H), 7.51 - 7.35 (m, 7H), 6.21 (d, *J* = 3.1 Hz, 1H), 5.96 (t, *J* = 3.0 Hz, 1H), 5.89 - 5.71 (m, 2H), 4.99 - 4.95 (m, 1H), 4.75 (dd, *J* = 11.9, 5.8 Hz, 1H), 4.68 (dd, *J* = 12.0, 4.5 Hz, 1H). **^{13}C NMR** (101 MHz, $CDCl_3$) δ 166.0, 165.3, 165.2, 162.8, 149.9, 140.2, 133.98, 133.93, 133.3, 130.0, 129.88, 129.83, 129.4, 128.7, 128.6, 128.5, 128.4, 128.3, 102.7, 91.7, 83.7, 80.5, 77.4, 63.9. **HRMS** (ESI) Calculated for $C_{30}H_{25}N_2O_9$ $[M+H]^+$: 557.1560 Found: 557.1558.

5-Fluoro-1-*N*-(2,3,5-tri-*O*-benzoyl-β-D-ribofuranosyl)-uracil (6d)

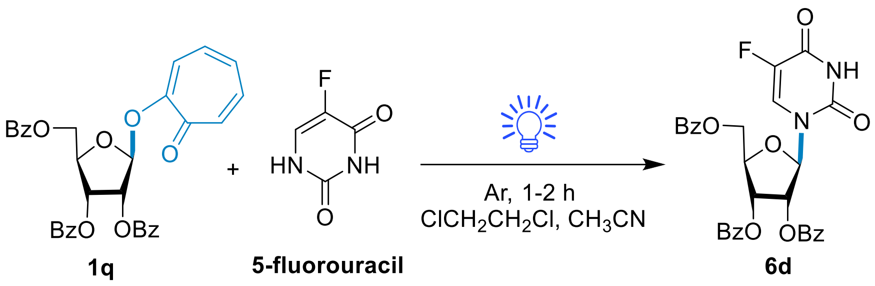

To a solution of 5-fluorouracil (6.5 mg, 0.050 mmol) in anhydrous CH_3CN (1.0 mL) was added BSTFA (53.1 uL, 0.200 mmol) under Ar atmosphere. The suspension was stirred at room temperature until it becomes a clear solution. Then the **1q** (34.0 mg, 0.0600 mmol) dissolved in $ClCH_2CH_2Cl$ (1.0 mL) and TMSOTf (0.90 uL, 0.0050 mmol) were added to the above solution through a syringe, the mixture was irradiated by blue LEDs at ambient

temperature. The progress of the reaction was monitored by TLC (1-2 h). Upon completion, the reaction mixture was concentrated in vacuo. The residue was purified by flash column chromatography on silica gel (petroleum ether/EtOAc = 2/1) to obtain **6d** (28.1 mg, 98%) as a white solid. **^{1}H NMR** (400 MHz, $CDCl_3$) δ 8.55 (s, 1H), 8.11 (d, *J* = 7.7 Hz, 2H), 7.99 (d, *J* = 7.8 Hz, 2H), 7.93 (d, *J* = 7.8 Hz, 2H), 7.64 - 7.48 (m, 6H), 7.43 - 7.34 (m, 4H), 6.35 (d, *J* = 5.9 Hz, 1H), 5.91 - 5.81 (m, 1H), 5.69 (t, *J* = 6.0 Hz, 1H), 4.82 (dd, *J* = 13.1, 3.8 Hz, 1H), 4.75 - 4.71 (m, 2H). **^{19}F NMR** (376 MHz, $CDCl_3$) δ -162.79. The ^{1}H NMR data coincide with the reported data[24].

2-Fluoro-6-chloro-9-*N*-(2,3,4,6-tetra-*O*-benzoyl-β-D-galactopyranosyl)-purine (6e)

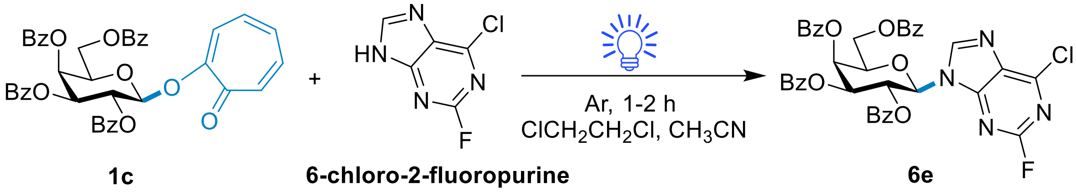

To a solution of 6-chloro-2-fluoropurine (8.6 mg, 0.050 mmol) in anhydrous CH_3CN (1.0 mL) was added BSTFA (53.1 uL, 0.200 mmol) under Ar atmosphere. The suspension was stirred at room temperature until it becomes a clear solution. Then the **1c** (42.0 mg, 0.0600 mmol) dissolved in $ClCH_2CH_2Cl$ (1.0 mL) and TMSOTf (0.90 uL, 0.0050 mmol) were added to the above solution through a syringe, the mixture was irradiated by blue LEDs at ambient temperature. The progress of the reaction was monitored by TLC (1-2 h). Upon completion, the reaction mixture was concentrated in vacuo. The residue was purified by flash column chromatography on silica gel (petroleum ether/EtOAc = 3/1) to obtain **6e** (33.8 mg, 90%) as a white solid. $[a]_D^{25}$: 44.76 (*c*: 0.21 $CHCl_3$). **^{1}H NMR** (400 MHz, $CDCl_3$) δ 8.41 (s, 1H), 8.27 - 8.21 (m, 2H), 8.03 - 7.96 (m, 2H), 7.82 - 7.67 (m, 5H), 7.62 - 7.52 (m, 3H), 7.50 - 7.38 (m, 4H), 7.33 - 7.26 (m, 3H), 7.26 - 7.24 (m, 1H), 6.50 (t, *J* = 9.7 Hz, 1H), 6.18 (d, *J* = 3.2 Hz, 1H), 6.10 (d, *J* = 9.3 Hz, 1H), 5.89 (dd, *J* = 10.1, 3.3 Hz, 1H), 4.74 - 4.59 (m, 2H), 4.50 (dd, *J* = 10.9, 5.1 Hz, 1H). **^{13}C NMR** (101 MHz, $CDCl_3$) δ 165.9, 165.3, 164.91, 157.4 (d, *J* = 220 Hz), 153.6 (d, *J* = 17 Hz), 153.4 (d, *J* = 17 Hz), 143.8 (d, *J* = 3 Hz), 134.0, 133.9, 133.5, 133.4, 130.5 (d, *J* = 5 Hz), 130.1, 129.8, 129.7, 129.1, 128.9, 128.7, 128.5, 128.48, 128.40, 127.6, 82.5, 74.7, 71.6, 68.4, 67.8, 62.0. **^{19}F NMR** (376 MHz, $CDCl_3$) δ -48.65. **HRMS** (ESI) Calculated for $C_{39}H_{28}ClFN_4NaO_9$ $[M+Na]^+$: 773.1427 Found: 773.1419.

2,6-Dichloro-9-*N*-(2,3,4-tri-*O*-benzoyl-β-D-xylopyranosyl)-purine (6f)

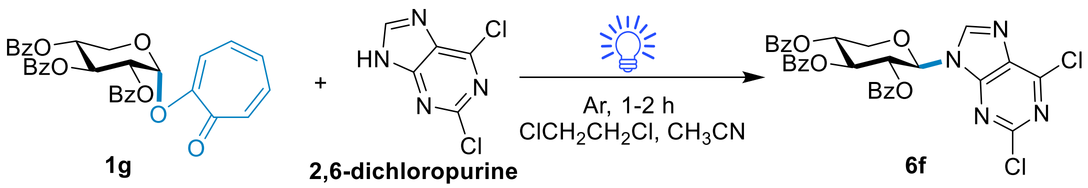

To a solution of 2,6-dichloropurine (9.4 mg, 0.050 mmol) in anhydrous CH_3CN (1.0 mL) was added BSTFA (53.1 uL, 0.200 mmol) under Ar atmosphere. The suspension was stirred at room temperature until it becomes a clear solution. Then the **1g** (34.0 mg, 0.0600 mmol) dissolved in $ClCH_2CH_2Cl$ (1.0 mL) and TMSOTf (0.90 uL, 0.0050 mmol) were added to the above solution through a syringe, the mixture was irradiated by Blue LEDs at ambient temperature. And the progress of the reaction was monitored by TLC (1-2 h). Upon completion, the reaction mixture was concentrated in vacuo. The residue was purified by flash column chromatography on silica gel (petroleum ether/EtOAc = 3/1) to obtain **6f** (33.8 mg, 91%) as a white solid. $[\alpha]_D^{25}$: 34.84 (*c*: 0.31 $CHCl_3$). **^{1}H NMR** (400 MHz, $CDCl_3$) δ 8.48 (s, 1H), 8.04 - 7.97 (m, 2H), 7.96 -7.87 (m, 2H), 7.81 - 7.72 (m, 2H), 7.63 - 7.55 (m, 1H), 7.52 - 7.41 (m, 4H), 7.33 - 7.28 (m, 4H), 6.17 - 6.12 (m, 2H), 5.99 (t, *J* = 9.5 Hz, 1H), 5.65 - 5.59 (m, 1H), 4.66 (dd, *J* = 11.6, 5.6 Hz, 1H), 4.05 - 3.84 (m, 1H). **^{13}C NMR** (101 MHz, $CDCl_3$) δ 165.46, 165.43, 164.8, 153.5, 152.9, 152.1, 143.3, 133.9, 133.7, 133.5, 130.5, 129.88, 129.81, 129.7, 128.5, 128.49, 128.44, 127.3, 81.8, 72.2, 71.1, 69.3, 66.2. **HRMS** (ESI) Calculated for $C_{31}H_{22}Cl_2N_4NaO_7$ $[M+Na]^+$: 655.0763 Found: 655.0750.

6-Chloro-9-*N*-(2,3,5-tri-*O*-benzoyl-β-D-ribofuranosyl)-purine (6g)

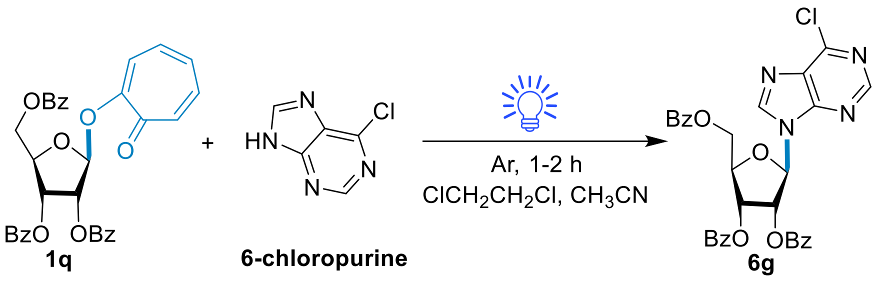

To a solution of 6-chloropurine (7.7 mg, 0.050 mmol) in anhydrous CH_3CN (1.0 mL) was added BSTFA (53.1 uL, 0.200 mmol) under Ar atmosphere. The suspension was stirred at room temperature until it becomes a clear solution. Then the **1q** (34.0 mg, 0.0600 mmol) dissolved in $ClCH_2CH_2Cl$ (1.0 mL) and TMSOTf (0.90 uL, 0.0050 mmol) were added to the above solution through a syringe, the mixture was irradiated by blue LEDs at ambient temperature. The progress of the reaction was monitored by TLC (1-2 h). Upon completion, the reaction mixture was concentrated in vacuo. The residue was purified by flash column chromatography on silica gel (petroleum ether/EtOAc = 3/1) to obtain product **6g** (26.9 mg, 94%) as a white solid. $[\alpha]_D^{25}$: -57.94 (*c*: 0.34 $CHCl_3$). **^{1}H NMR** (400 MHz, $CDCl_3$) δ 8.60 (s, 1H), 8.28 (s, 1H), 8.08 - 8.06 (m, 2H), 8.03 - 8.00 (m, 2H), 7.93 - 7.90 (m, 2H), 7.63 - 7.53 (m, 3H), 7.49 - 7.34 (m, 6H), 6.46 (d, *J* = 5.1 Hz, 1H), 6.42 (t, *J* = 5.3 Hz, 1H), 6.26 (t, *J* = 5.3 Hz, 1H), 4.94 (dd, *J* = 12.2, 3.2 Hz, 1H), 4.87 - 4.84 (m, 1H), 4.70 (dd, *J* = 12.2, 4.1 Hz, 1H). **^{13}C NMR** (101 MHz, $CDCl_3$) δ 166.0, 165.2, 165.1, 152.3, 151.6, 151.2, 143.9, 133.9, 133.8, 133.5, 132.4, 129.83, 129.82, 129.7, 129.2, 128.62, 128.60, 128.5, 128.2, 87.5, 81.0, 73.8, 71.3, 63.2. **HRMS** (ESI) Calculated for $C_{31}H_{23}ClN_4NaO_7$ $[M+Na]^+$: 621.1153 Found: 621.1155.

2-Fluoro-6-chloro-9-*N*-(2,3,5-tri-*O*-benzoyl-β-D-ribofuranosyl)-purine (6h)

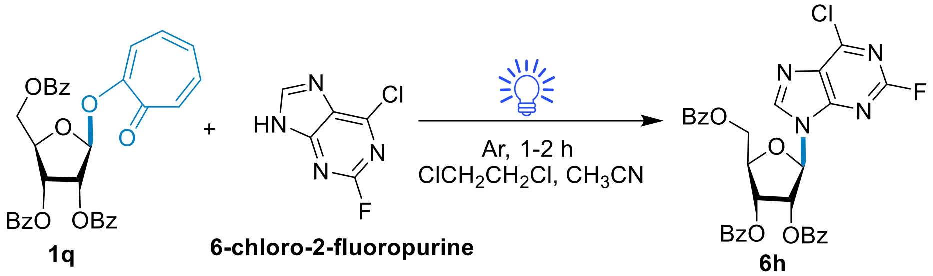

1q
6h

To a solution of 6-chloro-2-fluoropurine (8.6 mg, 0.050 mmol) in anhydrous CH_3CN (1.0 mL) was added BSTFA (53.1 uL, 0.200 mmol) under Ar atmosphere. The suspension was stirred at room temperature until it becomes a clear solution. Then the **1q** (34.0 mg, 0.0600 mmol) dissolved in $ClCH_2CH_2Cl$ (1.0 mL) and TMSOTf (0.90 uL, 0.0050 mmol) were added to the above solution through a syringe, the mixture was irradiated by blue LEDs at ambient temperature. The progress of the reaction was monitored by TLC (1-2 h). Upon completion, the reaction mixture was concentrated in vacuo. The residue was purified by flash column chromatography on silica gel (petroleum ether/EtOAc = 3/1) to obtain **6h** (29.2 mg, 95%) as a white solid. $[\alpha]_D^{25}$: -65.88 (*c*: 0.34 $CHCl_3$). **^{1}H NMR** (400 MHz, $CDCl_3$) δ 8.27 (s, 1H), 8.12 - 8.06 (m, 2H), 8.05 - 8.00 (m, 2H), 7.96 - 7.88 (m, 2H), 7.66 - 7.51 (m, 3H), 7.46 - 7.41 (m, 4H), 7.38 - 7.34 (m, 2H), 6.44 (d, *J* = 5.4 Hz, 1H), 6.22 (t, *J* = 5.6 Hz, 1H), 6.14 (dd, *J* = 5.8, 4.5 Hz, 1H), 4.97 - 4.82 (m, 2H), 4.73 (dd, *J* = 12.2, 4.0 Hz, 1H). **^{13}C NMR** (101 MHz, $CDCl_3$) δ 166.0, 165.2, 165.1, 157.2 (d, *J* = 220 Hz), 153.3 (d, *J* = 17 Hz), 153.1 (d, *J* = 17 Hz), 144.02 (d, *J* = 3 Hz), 134.0, 133.8, 133.6, 130.9(d, *J* = 5 Hz), 129.86, 129.84, 129.6, 129.0, 128.7, 128.6, 128.59, 128.54, 128.0, 87.0, 81.2, 74.0, 71.3, 63.3. **^{19}F NMR** (376 MHz, $CDCl_3$) δ -48.13. **HRMS** (ESI) Calculated for $C_{31}H_{22}ClFN_4NaO_7$ $[M+Na]^+$: 639.1059 Found: 639.1054.

3β-*O*-(2,3,4,6-Tetra-*O*-benzoyl-β-D-galactopyranosyl)-epiandrosterone (7a)

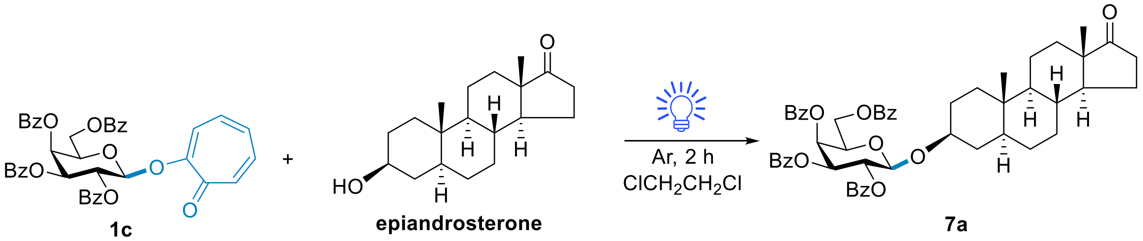

1c
7a

The glycosyl donor **1c** (1.56 g, 2.25 mmol), epiandrosterone (435.7 mg, 1.500 mmol) and TMSOTf (27.1 uL, 0.150 mmol) were dissolved in dry $ClCH_2CH_2Cl$ (50.0 mL), and the mixture was irradiated by blue LEDs at ambient temperature for 2 h. After the reaction was completed, the solvent was concentrated under reduced pressure. The resulting residue was eluted by flash column chromatography (petroleum ether/EtOAc = 8/1) to afford the glycosylated product **7a** as a pale-yellow solid (1.20 g, 93%). **^{1}H NMR** (400 MHz, $CDCl_3$) δ 8.15 - 8.08 (m, 2H), 8.07 - 8.01 (m, 2H), 7.99 - 7.93 (m, 2H), 7.82 - 7.75 (m, 2H), 7.64 - 7.51 (m, 3H), 7.50 - 7.36 (m, 7H), 7.25 - 7.22 (m, 2H), 5.98 (dd, *J* = 3.5, 1.1 Hz, 1H), 5.76 (dd, *J* = 10.4, 7.9 Hz, 1H), 5.59 (dd, *J* = 10.4, 3.5 Hz, 1H), 4.91 (d, *J*

= 8.0 Hz, 1H), 4.68 (dd, J = 11.2, 6.9 Hz, 1H), 4.42 (dd, J = 11.2, 6.5 Hz, 1H), 4.36 - 4.28 (m, 1H), 3.66 - 3.58 (m, 1H), 2.43 (dd, J = 19.2, 8.8 Hz, 1H), 2.09 - 2.00 (m, 1H), 1.95 - 1.86 (m, 2H), 1.82 - 1.69 (m, 2H), 1.63 - 1.43 (m, 6H), 1.30 - 1.13 (m, 6H), 1.05 - 0.86 (m, 3H), 0.83 (s, 3H), 0.72 (s, 3H), 0.65 - 0.59 (m, 1H). The ^{1}H NMR data coincide with the reported data[25].

17-*O*-(2,3,4,6-Tetra-*O*-benzoyl-β-D-galactopyranosyl)-estradiol benzoate (7b)

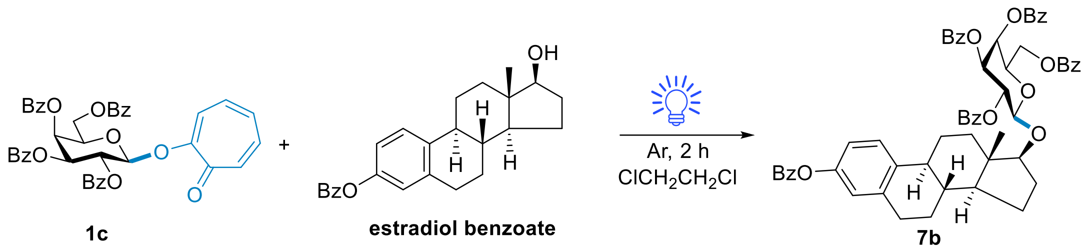

1c **estradiol benzoate** **7b**

The glycosyl donor **1c** (1.56 g, 2.25 mmol), estradiol benzoate (564.8 mg, 1.500 mmol) and TMSOTf (27.1 uL, 0.150 mmol) were dissolved in dry $ClCH_2CH_2Cl$ (50.0 mL), and the mixture was irradiated by blue LEDs at ambient temperature for 2 h. After the reaction was completed, the solvent was concentrated under reduced pressure. The resulting residue was eluted by flash column chromatography (petroleum ether/EtOAc = 8/1) to afford the glycosylated product **7b** as a pale-yellow solid (1.36 g, 95%). $[\alpha]_D^{25}$: 93.10 (c: 0.15 $CHCl_3$). **^{1}H NMR** (400 MHz, $CDCl_3$) δ 8.22 - 8.16 (m, 2H), 8.15 - 8.08 (m, 2H), 8.08 - 8.03 (m, 2H), 8.02 - 7.96 (m, 2H), 7.84 - 7.76 (m, 2H), 7.65 - 7.37 (m, 13H), 7.26 - 7.25 (m, 1H), 7.25 - 7.22 (m, 2H), 6.94 (dd, J = 8.5, 2.6 Hz, 1H), 6.90 (d, J = 2.5 Hz, 1H), 5.99 (dd, J = 3.5, 1.1 Hz, 1H), 5.81 (dd, J = 10.4, 7.9 Hz, 1H), 5.62 (dd, J = 10.4, 3.5 Hz, 1H), 4.88 (d, J = 7.9 Hz, 1H), 4.69 (dd, J = 11.3, 6.9 Hz, 1H), 4.44 (dd, J = 11.3, 6.5 Hz, 1H), 4.37 - 4.29 (m, 1H), 3.73 (t, J = 8.5 Hz, 1H), 2.86 - 2.83 (m, 2H), 2.22 - 1.99 (m, 3H), 1.91 - 1.70 (m, 3H), 1.67 - 1.59 (m, 1H), 1.44 - 1.27 (m, 4H), 1.25 - 1.17 (m, 1H), 1.16 - 1.04 (m, 1H), 0.65 (s, 3H). **^{13}C NMR** (101 MHz, $CDCl_3$) δ 166.0, 165.7, 165.6, 165.4, 165.2, 148.6, 138.2, 137.8, 133.5, 133.4, 133.28, 133.25, 133.20, 130.13, 130.11, 129.79, 129.71, 129.5, 129.0, 128.8, 128.59, 128.51, 128.4, 128.3, 128.2, 126.4, 121.5, 118.6, 102.4, 90.3, 71.7, 71.3, 70.0, 68.2, 62.1, 49.7, 44.0, 43.2, 38.1, 37.3, 29.5, 28.8, 26.9, 25.9, 23.0, 11.4. **HRMS** (ESI) Calculated for $C_{59}H_{58}NO_{12}$ $[M+NH_4]^+$: 972.3959 Found: 972.3959.

3-*O*-(2,3,4,6-Tetra-*O*-benzoyl-β-D-galactopyranosyl)-17-*O*-(2,3,4,6-tetra-*O*-benzoyl-β-D-galactopyranosyl)estradiol (7c)

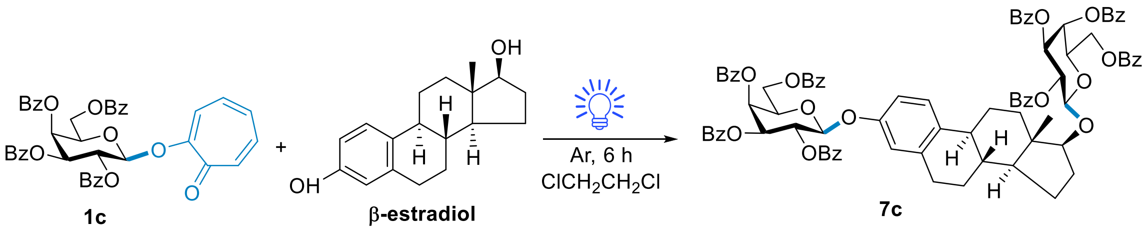

1c **β-estradiol** **7c**

The glycosyl donor **1c** (3.12 g, 4.50 mmol), β-estradiol (408.6 mg, 1.500 mmol) and TMSOTf (54.2 uL, 0.300 mmol) was dissolved in dry $ClCH_2CH_2Cl$ (50.0 mL), and the mixture was irradiated by blue LEDs at ambient temperature for 6 h. After the reaction was completed, the solvent was concentrated under reduced pressure. The resulting residue was eluted by flash column chromatography (petroleum ether/EtOAc = 6/1) to afford the glycosylated product **7c** as a pale-yellow solid (1.70 g, 80%). $[\alpha]_D^{25}$: 116.11 (*c*: 0.18 $CHCl_3$). **^{1}H NMR** (400 MHz, $CDCl_3$) δ 8.13 - 8.10 (m, 4H), 8.06 - 8.03 (m, 4H), 7.99 - 7.93 (m, 4H), 7.82 - 7.77 (m, 4H), 7.66 - 7.33 (m, 20H), 7.28 - 7.27 (m, 2H), 7.24 - 7.22 (m, 2H), 6.97 (d, *J* = 8.7 Hz, 1H), 6.84 - 6.64 (m, 2H), 6.12 - 5.93 (m, 3H), 5.80 (dd, *J* = 10.4, 7.9 Hz, 1H), 5.67 - 5.59 (m, 2H), 5.32 (d, *J* = 7.9 Hz, 1H), 4.87 (d, *J* = 7.9 Hz, 1H), 4.71 - 4.63 (m, 2H), 4.57 - 4.39 (m, 3H), 4.30 (t, *J* = 6.7 Hz, 1H), 3.71 (t, *J* = 8.5 Hz, 1H), 2.75 - 2.51 (m, 2H), 2.09 - 1.98 (m, 3H), 1.75 - 1.69 (m, 4H), 1.36 - 1.28 (m, 2H), 1.23 -1.17 (m, 3H), 1.11 - 1.04 (m, 1H), 0.61 (s, 3H). **^{13}C NMR** (101 MHz, $CDCl_3$) δ 166.08, 166.04, 165.7, 165.6, 165.5, 165.25, 165.23, 154.9, 138.1, 135.1, 133.7, 133.5, 133.37, 133.30, 133.1, 130.1, 129.86, 129.83, 129.80, 129.78, 129.72, 129.58, 129.55, 129.4, 129.2, 129.0, 128.9, 128.8, 128.7, 128.68, 128.61, 128.4, 128.39, 128.34, 128.30, 126.3, 117.0, 114.4, 102.4, 100.1, 90.3, 71.79, 71.76, 71.73, 71.3, 70.1, 69.6, 68.2, 68.1, 62.4, 62.1, 49.7, 43.8, 43.2, 38.2, 37.3, 29.7, 29.5, 28.8, 27.0, 26.0, 23.0, 11.4. **HRMS** (ESI) Calculated for $C_{86}H_{80}NO_{20}$ $[M+NH_4]^+$: 1446.5274 Found: 1446.5276.

Cholesteryl 2,3,4,6-tetra-*O*-benzoyl-β-D-galactopyranoside(7d)

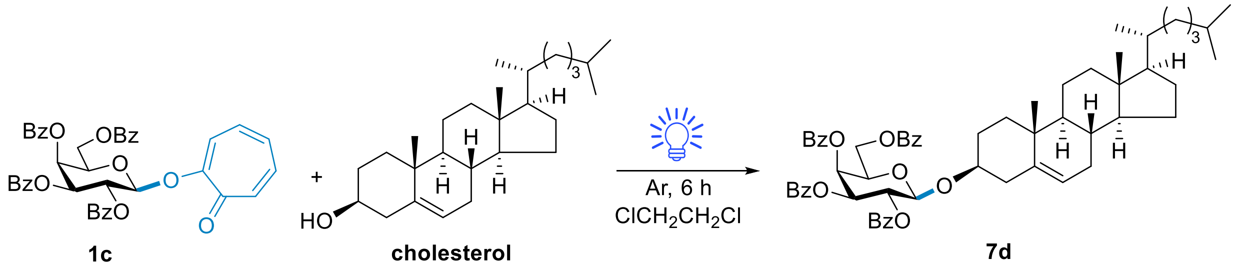

The glycosyl donor **1c** (2.10 g, 3.00 mmol), cholesterol (773.3 mg, 2.000 mmol) and TMSOTf (36.2 uL, 0.200 mmol) were dissolved in dry $ClCH_2CH_2Cl$ (70.0 mL), and the mixture was irradiated by blue LEDs at ambient temperature for 6 h. After the reaction was completed, the solvent was concentrated under reduced pressure. The resulting residue was eluted by flash column chromatography (petroleum ether/EtOAc = 8/1) to afford the glycosylated product **7d** as a white solid (1.64 g, 85%). **^{1}H NMR** (400 MHz, $CDCl_3$) δ 8.14 - 8.06 (m, 2H), 8.05 - 8.00 (m, 2H), 7.99 - 7.93 (m, 2H), 7.84 - 7.76 (m, 2H), 7.64 - 7.36 (m, 10H), 7.26 - 7.20 (m, 2H), 5.97 (dd, *J* = 3.5, 1.1 Hz, 1H), 5.77 (dd, *J* = 10.4, 7.9 Hz, 1H), 5.59 (dd, *J* = 10.4, 3.5 Hz, 1H), 5.26 - 5.20 (m, 1H), 4.91 (d, *J* = 8.0 Hz, 1H), 4.67 (dd, *J* = 11.3, 6.9 Hz, 1H), 4.42 (dd, *J* = 11.3, 6.4 Hz, 1H), 4.36 - 4.28 (m, 1H), 3.58 - 3.52 (m, 1H), 2.19 - 2.17 (m, 2H), 2.05 - 1.57 (m, 7H), 1.53 - 0.95 (m, 19H), 0.92 (d, *J* = 6.1 Hz, 6H), 0.87 (d, *J* = 1.6 Hz, 3H), 0.85 (d, *J* = 2.0 Hz, 3H), 0.66 (s, 3H). The ^{1}H NMR data coincide with the reported data[13].

Simvastatinyl 2,3,4-tri-*O*-benzoyl-α-L-rhamnopyranoside (7e)

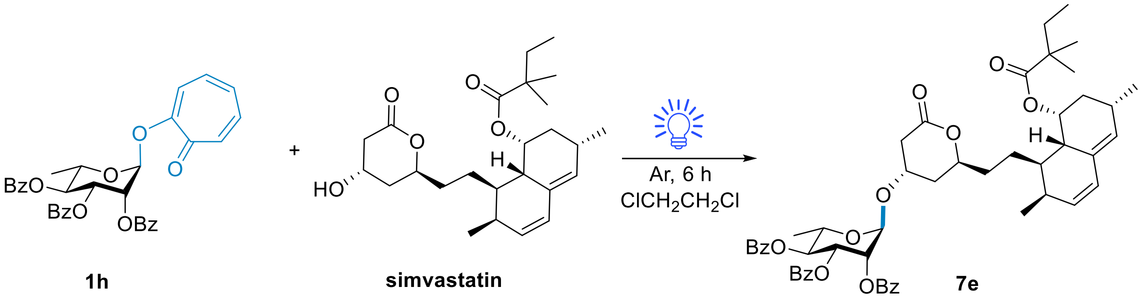

1h **simvastatin** **7e**

The glycosyl donor **1h** (1.31 g, 2.25 mmol), simvastatin (627.9 mg, 1.500 mmol) and TMSOTf (27.1 uL, 0.150 mmol) were dissolved in dry $ClCH_2CH_2Cl$ (70.0 mL), and the mixture was irradiated by blue LEDs at ambient temperature for 6 h. After the reaction was completed, the solvent was concentrated under reduced pressure. The resulting residue was eluted by flash column chromatography (petroleum ether/EtOAc = 8/1) to afford the glycosylated product **7e** as white solid (1.23 g, 94%). $[\alpha]_D^{25}$: 170.63 (*c*: 0.16 $CHCl_3$). **^{1}H NMR** (400 MHz, $CDCl_3$) δ 8.16 - 8.05 (m, 2H), 8.04 - 7.97 (m, 2H), 7.88 - 7.78 (m, 2H), 7.67 - 7.58 (m, 1H), 7.55 - 7.47 (m, 3H), 7.43 - 7.36 (m, 3H), 7.28 - 7.24 (m, 2H), 6.00 (d, *J* = 9.6 Hz, 1H), 5.80 (dd, *J* = 9.6, 6.2 Hz, 1H), 5.77 - 5.65 (m, 2H), 5.61 (dd, *J* = 3.1, 1.7 Hz, 1H), 5.52 (t, *J* = 3.2 Hz, 1H), 5.42 - 5.39 (m, 1H), 5.16 (d, *J* = 1.8 Hz, 1H), 4.75 - 4.62 (m, 1H), 4.37 - 4.33 (m, 1H), 4.20 - 4.13 (m, 1H), 2.99 - 2.90 (m, 1H), 2.81 (dd, *J* = 17.4, 5.2 Hz, 1H), 2.44 - 2.36 (m, 2H), 2.33 - 2.17 (m, 2H), 2.00 - 1.89 (m, 3H), 1.77 - 1.68 (m, 2H), 1.63 - 1.52 (m, 3H), 1.45 - 1.38 (m, 2H), 1.37 (d, *J* = 6.2 Hz, 3H), 1.14 (s, 6H), 1.08 (d, *J* = 7.4 Hz, 3H), 0.93 (d, *J* = 6.9 Hz, 3H), 0.84 (t, *J* = 7.4 Hz, 3H). **^{13}C NMR** (101 MHz, $CDCl_3$) δ 177.6, 169.4, 165.7, 165.6, 165.3, 133.5, 133.3, 133.1, 132.8, 131.5, 129.9, 129.8, 129.7, 129.6, 129.2, 129.1, 128.6, 128.46, 128.41, 128.2, 97.0, 76.5, 71.4, 70.9, 70.2, 69.7, 67.9, 67.5, 43.0, 37.5, 36.8, 36.6, 33.6, 33.0, 32.9, 32.8, 30.6, 27.3, 24.8, 24.0, 23.0, 17.5, 13.9, 9.3. **HRMS** (ESI) Calculated for $C_{52}H_{64}NO_{12}$ $[M+NH_4]^+$: 894.4429 Found: 894.4431.

1-*N*-(2,3,5-Tri-*O*-benzoyl-β-D-ribofuranosyl)-thymine (7f)

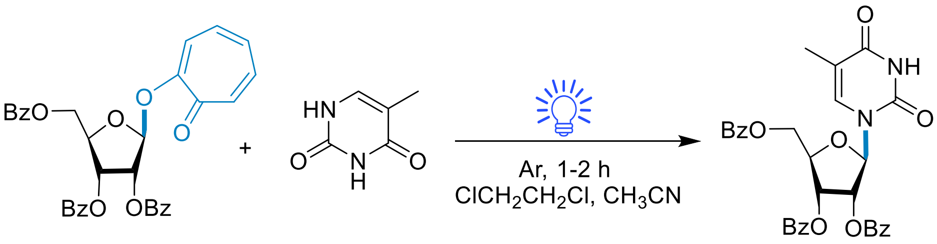

1q **thymine** **7f**

To a solution of thymine (252.0 mg, 2.000 mmol) in anhydrous CH_3CN (30.0 mL) was added BSTFA (2.10 mL, 8.00 mmol) under Ar atmosphere. The suspension was stirred at room temperature until it becomes a clear solution. Then the **1q** (1.36 g, 2.40 mmol) dissolved in $ClCH_2CH_2Cl$ (30.0 mL) and TMSOTf (36.2 uL, 0.200 mmol) were added to the above solution through a syringe, the mixture was irradiated by blue LEDs at ambient temperature. And the progress of the reaction was monitored by TLC (2 h). Upon completion, the reaction mixture was

concentrated in vacuo. The residue was purified by flash column chromatography on silica gel (petroleum ether/EtOAc = 3/1) to obtain product **7f** (0.95 g, 83%) as a white solid. **^{1}H NMR** (400 MHz, $CDCl_3$) δ 8.14 (d, *J* = 7.7 Hz, 2H), 7.99 - 7.93 (m, 4H), 7.68 - 7.34 (m, 9H), 7.16 (s, 1H), 6.44 (d, *J* = 6.1 Hz, 1H), 5.93 - 5.91 (m, 1H), 5.77 - 5.74 (m, 1H), 4.88 (d, *J* = 12.1 Hz, 1H), 4.71 - 4.63 (m, 2H), 1.59 (s, 3H). The ^{1}H NMR data coincide with the reported data[23].

Methyl 2,3,4-tri-*O*-benzyl-6-*O*-(2,3-di-*O*-benzoyl-4-*O*-benzyl-6-*O*-tert-butyldiphenylsilyl-β-D-glucopyranosyl)-α-D-glucopyranoside (8a)

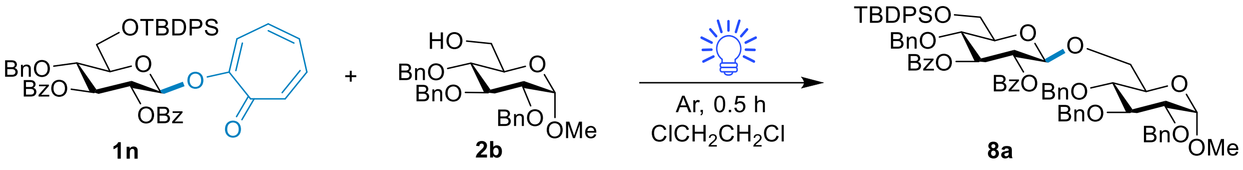

The glycosyl donor **1n** (152.6 mg, 0.1860 mmol), acceptor **2b** (58.0 mg, 0.125 mmol) and TMSOTf (2.30 uL, 0.0125 mmol) were dissolved in dry $ClCH_2CH_2Cl$ (2.0 mL), and the mixture was irradiated by blue LEDs at ambient temperature for 0.5 h. After the reaction was completed, the solvent was concentrated under reduced pressure. The resulting residue was eluted by flash column chromatography (petroleum ether/EtOAc = 10/1) to afford the glycosylated product **8a** as white solid (138.7 mg, 97%). $[a]_D^{25}$: 30.00 (*c*: 0.19 $CHCl_3$). **^{1}H NMR** (400 MHz, $CDCl_3$) δ 7.97 - 7.91 (m, 2H), 7.90 - 7.84 (m, 2H), 7.78 - 7.75 (m, 2H), 7.75 - 7.68 (m, 2H), 7.49 - 7.45 (m, 1H), 7.42 - 7.23 (m, 19H), 7.22 - 7.15 (m, 5H), 7.14 - 7.10 (m, 3H), 7.06 - 7.01 (m, 4H), 5.74 - 5.69 (m, 1H), 5.46 (dd, *J* = 9.9, 7.9 Hz, 1H), 4.88 (d, *J* = 11.0 Hz, 1H), 4.73 (d, *J* = 12.1 Hz, 1H), 4.66 (dd, *J* = 11.6, 9.4 Hz, 2H), 4.62 - 4.56 (m, 3H), 4.53 (d, *J* = 3.5 Hz, 1H), 4.46 (d, *J* = 11.2 Hz, 1H), 4.27 (d, *J* = 11.2 Hz, 1H), 4.19 (dd, *J* = 10.5, 1.6 Hz, 1H), 4.06 - 3.94 (m, 3H), 3.89 (t, *J* = 9.3 Hz, 1H), 3.77 - 3.63 (m, 2H), 3.55 (dt, *J* = 9.9, 2.9 Hz, 1H), 3.47 - 3.33 (m, 2H), 3.22 (s, 3H), 1.09 (s, 9H). **^{13}C NMR** (101 MHz, $CDCl_3$) δ 165.7, 165.1, 138.7, 138.2, 138.1, 137.3, 135.8, 135.5, 133.4, 133.0, 132.8, 129.69, 129.67, 129.5, 129.4, 128.39, 128.33, 128.25, 128.20, 128.0, 127.88, 127.83, 127.75, 127.73, 127.63, 127.60, 127.46, 127.40, 127.3, 101.2, 97.9, 81.8, 79.7, 76.1, 76.0, 75.4, 75.2, 74.9, 74.5, 73.3, 72.2, 69.6, 67.8, 62.5, 54.9, 26.8, 19.2. **HRMS** (ESI) Calculated for $C_{71}H_{78}NO_{13}Si$ $[M+NH_4]^+$: 1180.5242 Found: 1180.5248.

Methyl 2,3,4-tri-*O*-benzyl-6-*O*-(2,3-di-*O*-benzoyl-4-*O*-benzyl-β-D-glucopyranosyl)-α-D-glucopyranoside (8b)

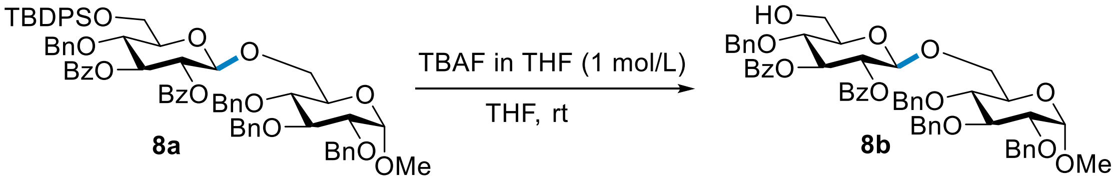

To a solution of **8a** (138.7 mg, 0.1200 mmol) in anhydrous THF (5.0 mL) was added TBAF/THF (1.0 mol/L, 0.18 mL, 0.18 mmol) under Air atmosphere. Then the mixture was stirred at room temperature and the progress of the reaction was monitored by TLC. Upon completion, the reaction mixture was concentrated in vacuo. The residue was purified by flash column chromatography on silica gel (petroleum ether/EtOAc = 4/1) to obtained **8b** as a white solid (102.9 mg, 92%). $[a]_D^{25}$: 26.15 (*c*: 0.13 $CHCl_3$). **^{1}H NMR** (400 MHz, $CDCl_3$) δ 7.91 - 7.84 (m, 4H), 7.49 (t, *J* = 7.6 Hz, 1H), 7.41 - 7.26 (m, 12H), 7.25 - 7.08 (m, 11H), 7.06 - 6.97 (m, 2H), 5.71 (t, *J* = 9.5 Hz, 1H), 5.40 (t, *J* = 8.7 Hz, 1H), 4.88 (d, *J* = 10.9 Hz, 1H), 4.82 - 4.64 (m, 3H), 4.60 (d, *J* = 12.4 Hz, 3H), 4.51 (d, *J* = 3.6 Hz, 1H), 4.44 (d, *J* = 11.1 Hz, 1H), 4.24 (d, *J* = 11.1 Hz, 1H), 4.07 (d, *J* = 10.2 Hz, 1H), 3.99 - 3.75 (m, 4H), 3.71 - 3.66 (m, 2H), 3.57 (d, *J* = 9.6 Hz, 1H), 3.47 - 3.33 (m, 2H), 3.23 (s, 3H), 2.05 (br, 1H). **^{13}C NMR** (101 MHz, $CDCl_3$) δ 165.7, 165.1, 138.7, 138.1, 137.1, 133.1, 133.0, 129.7, 129.3, 129.2, 128.4, 128.3, 128.28, 128.25, 128.17, 128.11, 127.9, 127.89, 127.86, 127.5, 127.4, 101.1, 98.0, 81.8, 79.7, 75.54, 75.50, 75.4, 74.9, 74.8, 74.6, 73.3, 71.9, 69.5, 68.3, 61.5, 55.0. **HRMS** (ESI) Calculated for $C_{55}H_{60}NO_{13}$ $[M+Na]^+$: 942.4065 Found: 942.4067.

Methyl (2,3-di-*O*-benzoyl-4-*O*-benzyl-6-*O*-tert-butyldiphenylsilyl-β-D-glucopyranosyl)-(1 → 6)-(2,3-di-*O*-benzoyl-4-*O*-benzyl-β-D-glucopyranosyl)-(1 → 6)-2,3,4-tri-*O*-benzyl-α-D-glucopyranoside (8c)

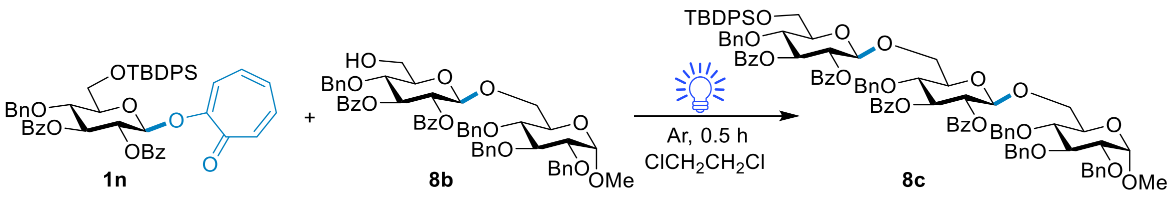

The glycosyl donor **1n** (137.0 mg, 0.1700 mmol), acceptor **8b** (102.6 mg, 0.1100 mmol) and TMSOTf (2.00 uL, 0.0110 mmol) were dissolved in dry $ClCH_2CH_2Cl$ (4.0 mL), and the mixture was irradiated by blue LEDs at ambient temperature for 0.5 h. After the reaction was completed, the solvent was concentrated under reduced pressure. The resulting residue was eluted by flash column chromatography (petroleum ether/EtOAc = 8/1) to afford the glycosylated product **8c** as a colorless syrup (169.3mg, 94%). $[a]_D^{25}$: 28.20 (*c*: 0.39 $CHCl_3$). **^{1}H NMR** (400 MHz, $CDCl_3$) δ 7.95 - 7.90 (m, 4H), 7.82 - 7.78 (m, 6H), 7.73 - 7.71 (m, 2H), 7.54 - 7.27 (m, 20H), 7.24 - 7.19 (m, 9H), 7.14 - 7.10 (m, 8H), 7.07 - 7.01 (m, 2H), 6.97 - 6.91 (m, 4H), 5.71 (t, *J* = 9.6 Hz, 1H), 5.60 (t, *J* = 8.9 Hz, 1H), 5.43 (dd, *J* = 9.9, 7.8 Hz, 1H), 5.34 (dd, *J* = 9.9, 7.8 Hz, 1H), 4.84 (dd, *J* = 11.9, 9.4 Hz, 2H), 4.75 - 4.50 (m, 6H), 4.47 (d, *J* = 7.9 Hz, 1H), 4.34 - 4.22 (m, 4H), 4.16 - 3.95 (m, 5H), 3.84 (t, *J* = 9.3 Hz, 1H), 3.75 (dd, *J* = 11.6, 5.1 Hz, 1H), 3.68 - 3.49 (m, 5H), 3.42 - 3.30 (m, 2H), 3.22 (s, 3H), 1.12 (s, 9H). **^{13}C NMR** (101 MHz, $CDCl_3$) δ 165.7, 165.5, 165.2, 165.0, 138.9, 138.3, 138.2, 137.4, 137.0, 135.9, 135.6, 133.5, 133.15, 133.13, 133.0, 132.95, 132.91, 129.74, 129.70, 129.68, 129.63, 129.61, 129.4, 129.32, 129.31, 128.41, 128.40, 128.32, 128.30, 128.27, 128.22, 128.1, 127.9, 127.84, 127.80, 127.71, 127.70, 127.4, 127.39, 127.31, 101.2, 100.8, 98.0, 81.9, 79.7,

77.1, 76.1, 76.0, 75.9, 75.4, 75.3, 75.2, 75.1, 75.0, 74.5, 73.3, 72.5, 71.9, 69.4, 67.6, 67.5, 62.5, 55.2, 26.9, 19.3. **HRMS** (ESI) Calculated for $C_{98}H_{102}NO_{20}Si$ $[M+NH_4]^+$: 1640.6764 Found: 1640.6749.

Methyl (2,3-di-*O*-benzoyl-4-*O*-benzyl-β-D-glucopyranosyl)-(1 → 6)-(2,3-di-*O*-benzoyl-4-*O*-benzyl-β-D-glucopyranosyl)-(1→6)-2,3,4-tri-*O*-benzyl-α-D-glucopyranoside (8d)

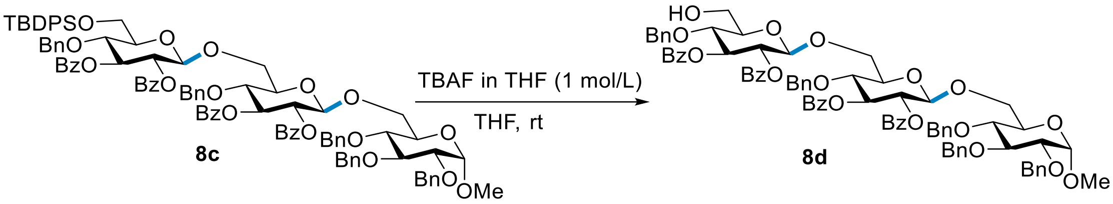

To a solution of **8c** (169.3 mg, 0.1040 mmol) in anhydrous THF (5.0 mL) was added TBAF/THF (1.0 mol/L, 0.16 mL, 0.16 mmol) under Air atmosphere. Then the mixture was stirred at room temperature and the progress of the reaction was monitored by TLC. Upon completion, the reaction mixture was concentrated in vacuo. The residue was purified by flash column chromatography on silica gel (petroleum ether/EtOAc = 3/1) to obtained **8d** as a colorless syrup (119.5 mg, 83%). $[\alpha]_D^{25}$: 13.33 (*c*: 0.06 $CHCl_3$). **^{1}H NMR** (400 MHz, $CDCl_3$) δ 8.01 - 7.69 (m, 8H), 7.56 - 7.26 (m, 17H), 7.25 - 7.07 (m, 16H), 7.03 - 6.90 (m, 4H), 5.71 (t, *J* = 9.7 Hz, 1H), 5.60 (t, *J* = 9.6 Hz, 1H), 5.42 - 5.35 (m, 2H), 5.00 - 4.25 (m, 12H), 4.19 (d, *J* = 11.1 Hz, 1H), 4.14 - 3.75 (m, 8H), 3.67 - 3.58 (m, 4H), 3.43 - 3.34 (m, 2H), 3.26 (s, 3H), 2.26 (br, 1H). **^{13}C NMR** (101 MHz, $CDCl_3$) δ 165.67, 165.61, 165.1, 165.0, 138.8, 138.2, 138.1, 137.2, 137.0, 133.2, 133.15, 133.12, 132.9, 129.7, 129.6, 129.37, 129.35, 129.2, 128.4, 128.37, 128.33, 128.2, 128.1, 128.0, 127.9, 127.85, 127.83, 127.80, 127.47, 127.40, 101.2, 100.8, 97.9, 81.8, 79.7, 77.2, 75.8, 75.6, 75.4, 75.05, 75.01, 74.8, 74.7, 74.6, 73.3, 72.2, 71.8, 69.5, 68.1, 67.8, 61.5, 55.2. **HRMS** (ESI) Calculated for $C_{82}H_{84}NO_{20}$ $[M+NH_4]^+$: 1402.5587 Found: 1402.5605.

Methyl (2,3-di-*O*-benzoyl-4-*O*-benzyl-6-*O*-tert-butyldiphenylsilyl-β-D-glucopyranosyl)-(1 → 6)-(2,3-di-*O*-benzoyl-4-*O*-benzyl-β-D-glucopyranosyl)-(1→6)-(2,3-di-*O*-benzoyl-4-*O*-benzyl-β-D-glucopyranosyl)-(1→6)-2,3,4-tri-*O*-benzyl-α-D-glucopyranoside (8e)

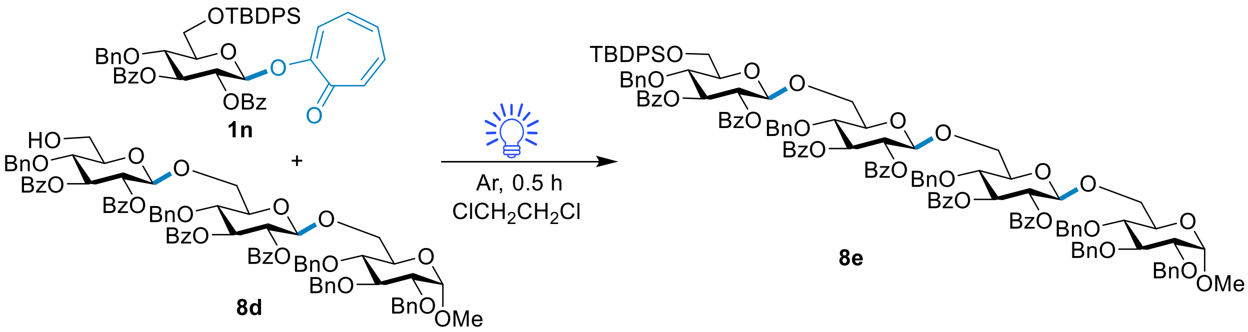

The glycosyl donor **1n** (105.8 mg, 0.1290 mmol), acceptor **8d** (119.1 mg, 0.08600 mmol) and TMSOTf (1.60 uL, 0.00860 mmol) were dissolved in dry $ClCH_2CH_2Cl$ (4.0 mL), and the mixture was irradiated by blue LEDs at ambient temperature for 0.5 h. After the reaction was completed, the solvent was concentrated under reduced pressure. The resulting residue was eluted by flash column chromatography (petroleum ether/EtOAc = 6/1) to afford the glycosylated product **8e** as colorless syrup (150.4 mg, 84%). $[\alpha]_D^{25}$: 10.00 (*c*: 0.08 $CHCl_3$). **^{1}H NMR** (400 MHz, $CDCl_3$) δ 7.96 - 7.92 (m, 4H), 7.90 - 7.85 (m, 2H), 7.85 - 7.75 (m, 8H), 7.75 - 7.70 (m, 2H), 7.54 - 7.26 (m, 27H), 7.25 - 7.18 (m, 9H), 7.14 - 7.07 (m, 10H), 7.03 - 7.01 (m, 2H), 6.95 - 6.91 (m, 4H), 6.88 - 6.86 (m, 2H), 5.75 (t, *J* = 9.6 Hz, 1H), 5.64 - 5.52 (m, 2H), 5.47 (dd, *J* = 9.9, 7.9 Hz, 1H), 5.33 - 5.28 (m, 2H), 4.85 (d, *J* = 11.1 Hz, 1H), 4.78 - 4.63 (m, 4H), 4.62 - 4.52 (m, 4H), 4.44 (d, *J* = 7.9 Hz, 1H), 4.32 - 4.19 (m, 5H), 4.17 - 4.04 (m, 5H), 3.98 - 3.97 (m, 2H), 3.84 (t, *J* = 9.3 Hz, 1H), 3.74 - 3.51 (m, 8H), 3.47 (dd, *J* = 10.0, 5.0 Hz, 1H), 3.43 - 3.33 (m, 2H), 3.21 (s, 3H), 1.12 (s, 9H). **^{13}C NMR** (101 MHz, $CDCl_3$) δ 165.8, 165.54, 165.50, 165.2, 165.0, 138.9, 138.3, 138.2, 137.3, 137.14, 137.11, 135.9, 135.5, 133.4, 133.16, 133.14, 133.06, 133.00, 132.9, 132.8, 129.7, 129.68, 129.66, 129.64, 129.5, 129.4, 129.34, 129.31, 129.2, 128.4, 128.39, 128.36, 128.31, 128.29, 128.26, 128.24, 128.22, 128.19, 128.10, 127.9, 127.8, 127.77, 127.72, 127.67, 127.64, 127.62, 127.38, 127.33, 101.3, 101.1, 100.9, 98.0, 81.8, 79.6, 77.1, 76.1, 76.0, 75.9, 75.4, 75.2, 75.18, 75.15, 75.14, 75.0, 74.8, 74.5, 73.3, 72.3, 72.2, 71.8, 69.4, 67.8, 67.6, 67.3, 62.5, 55.2, 26.9, 19.3. **HRMS** (ESI) Calculated for $C_{125}H_{126}NO_{27}Si$ $[M+NH_4]^+$: 2100.8286 Found: 2100.8259.

Methyl (2,3-di-*O*-benzoyl-4-*O*-benzyl-β-D-glucopyranosyl)-(1 → 6)-(2,3-di-*O*-benzoyl-4-*O*-benzyl-β-D-glucopyranosyl)-(1 → 6)-(2,3-di-*O*-benzoyl-4-*O*-benzyl-β-D-glucopyranosyl)-(1 → 6)-2,3,4-tri-*O*-benzyl-α-D-glucopyranoside (8f)

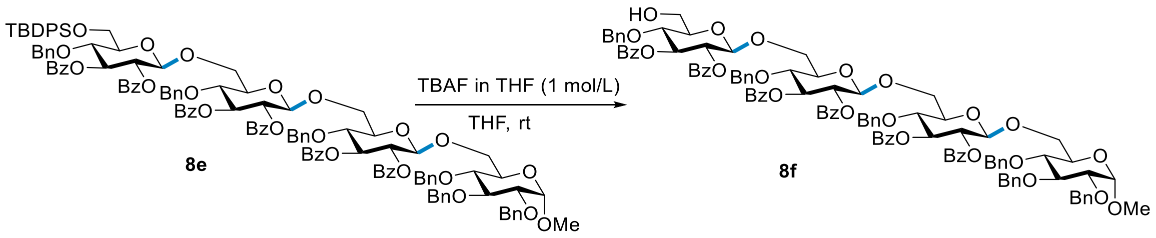

To a solution of **8e** (150.4 mg, 0.07200 mmol) in anhydrous THF (5.0 mL) was added TBAF/THF (1.0 mol/L, 0.11 mL, 0.11 mmol) under Air atmosphere. Then the mixture was stirred at room temperature and the progress of the reaction was monitored by TLC. Upon completion, the reaction mixture was concentrated in vacuo. The residue was purified by flash column chromatography on silica gel (petroleum ether/EtOAc = 3/1) to obtained product **8f** as a colorless syrup (108.9 mg, 82%). $[\alpha]_D^{25}$: 8.00 (*c*: 0.15 $CHCl_3$). **^{1}H NMR** (400 MHz, $CDCl_3$) δ 7.96 - 7.82 (m, 12H), 7.55 - 7.26 (m, 24H), 7.22 (d, *J* = 4.3 Hz, 5H), 7.14 (d, *J* = 7.9 Hz, 13H), 7.04 - 6.99 (m, 2H), 6.99 - 6.90 (m, 4H), 5.77 (t, *J* = 9.5 Hz, 1H), 5.63 - 5.58 (m, 2H), 5.49 (t, *J* = 8.7 Hz, 1H), 5.38 - 5.30 (m, 2H), 4.85 (d, *J* = 10.9 Hz, 1H), 4.76 - 4.71 (m, 2H), 4.68 - 4.51 (m, 7H), 4.40 - 4.15 (m, 7H), 4.08 - 3.75 (m, 10H), 3.66 - 3.54 (m, 5H), 3.42 - 3.32 (m, 2H), 3.24 (s, 3H), 2.52 (br, 1H). **^{13}C NMR** (101 MHz, $CDCl_3$) δ 165.7, 165.6, 165.5, 165.2,

165.19, 165.10, 138.8, 138.29, 138.21, 137.2, 133.19, 133.14, 133.11, 133.0, 132.9, 129.74, 129.72, 129.69, 129.61, 129.5, 129.43, 129.40, 129.35, 129.32, 129.2, 128.42, 128.40, 128.37, 128.36, 128.33, 128.26, 128.23, 128.20, 128.1, 128.0, 127.9, 127.89, 127.84, 127.79, 127.76, 127.74, 127.4, 127.3, 101.7, 101.5, 100.6, 97.9, 81.8, 79.7, 75.89, 75.85, 75.6, 75.4, 75.28, 75.21, 75.0, 74.9, 74.76, 74.70, 74.6, 73.1, 72.3, 72.2, 71.9, 69.4, 68.3, 67.7, 61.5, 55.2. **HRMS** (ESI) Calculated for $C_{109}H_{108}NO_{27}$ $[M+NH_4]^+$: 1862.7109 Found: 1862.7064.

Methyl (2,3-di-*O*-benzoyl-4-*O*-benzyl-6-*O*-tert-butyldiphenylsilyl-β-D-glucopyranosyl)-(1 → 6)- (2,3-di-*O*-benzoyl-4-*O*-benzyl-β-D-glucopyranosyl)-(1→6)-(2,3-di-*O*-benzoyl-4-*O*-benzyl-β-D-glucopyranosyl)-(1 → 6)-(2,3-di-*O*-benzoyl-4-*O*-benzyl-β-D-glucopyranosyl)-(1 → 6)-2,3,4-tri-*O*-benzyl-α-D-glucopyranoside (8g)

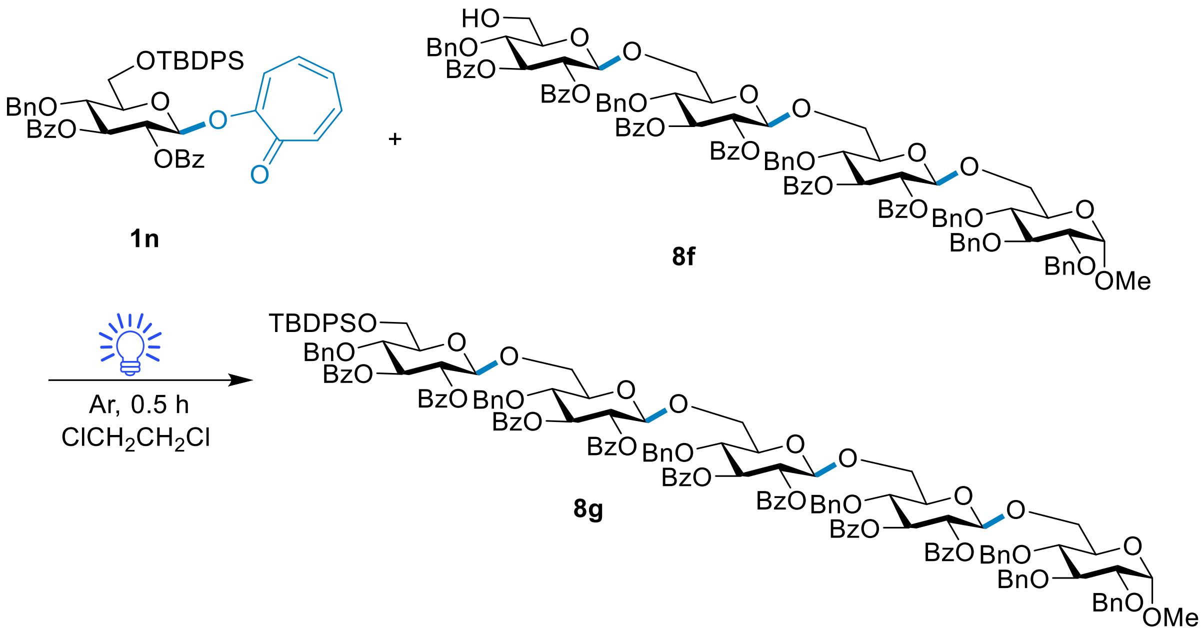

The glycosyl donor **1n** (72.6 mg, 0.0880 mmol), acceptor **8f** (108.8 mg, 0.05900 mmol) and TMSOTf (1.10 uL, 0.00590 mmol) were dissolved in dry $ClCH_2CH_2Cl$ (4.0 mL), and the mixture was irradiated by blue LEDs at ambient temperature for 0.5 h. After the reaction was completed, the solvent was concentrated under reduced pressure. The resulting residue was eluted by flash column chromatography (petroleum ether/EtOAc = 4/1) to afford the glycosylated product **8g** as colorless syrup (115.5 mg, 77%). $[\alpha]_D^{25}$: 18.00 (*c*: 0.10 $CHCl_3$). **^{1}H NMR** (400 MHz, $CDCl_3$) δ 8.03 - 7.93 (m, 4H), 7.93 - 7.78 (m, 12H), 7.75 - 7.70 (m, 4H), 7.52 - 7.26 (m, 26H), 7.25 - 7.14 (m, 13H), 7.14 - 6.90 (m, 22H), 6.90 - 6.79 (m, 4H), 5.76 (t, *J* = 9.6 Hz, 1H), 5.70 - 5.61 (m, 3H), 5.55 - 5.31 (m, 4H), 4.87 - 4.83 (m, 2H), 4.77 - 4.52 (m, 8H), 4.42 (d, *J* = 10.8 Hz, 1H), 4.37 - 4.02 (m, 13H), 3.98 - 3.71 (m, 9H), 3.67 - 3.57 (m, 6H), 3.46 - 3.37 (m, 2H), 3.25 (s, 3H), 1.13 (s, 9H). **^{13}C NMR** (151 MHz, $CDCl_3$) δ 165.8, 165.59, 165.55, 165.18, 165.15, 165.10, 164.8, 138.9, 138.4, 138.2, 137.4, 137.3, 137.1, 137.0, 135.8, 135.5, 133.5, 133.2, 133.1, 133.06, 133.04, 133.01, 132.89, 132.83, 132.7, 132.6, 129.8, 129.7, 129.68, 129.66, 129.56, 129.54,

129.48, 129.43, 129.39, 129.34, 128.4, 128.38, 128.32, 128.30, 128.24, 128.21, 128.18, 128.15, 128.14, 128.12, 128.10, 128.08, 128.00, 127.9, 127.8, 127.79, 127.78, 127.77, 127.73, 127.70, 127.67, 127.65, 127.62, 127.61, 127.3, 127.29, 127.24, 102.1, 101.6, 101.4, 100.7, 97.9, 81.9, 79.7, 77.4, 76.7, 76.1, 75.55, 75.51, 75.4, 75.3, 75.1, 74.9, 74.8, 74.7, 74.5, 74.4, 74.0, 73.2, 72.7, 72.4, 72.1, 72.0, 69.4, 69.0, 68.9, 68.6, 67.8, 62.8, 55.2, 26.9, 19.2. **HRMS** (ESI) Calculated for $C_{125}H_{126}NO_{27}Si$ $[M+NH_4]^+$: 2560.9809 Found: 2560.9792.

Methyl (2,3-di-*O*-benzoyl-4-*O*-benzyl-β-D-glucopyranosyl)-(1→6)- (2,3-di-*O*-benzoyl-4-*O*-benzyl-β-D-glucopyranosyl)-(1→6)-(2,3-di-*O*-benzoyl-4-*O*-benzyl-β-D-glucopyranosyl)-(1→6)-(2,3-di-*O*-benzoyl-4-*O*-benzyl-β-D-glucopyranosyl)-(1→6)-2,3,4-tri-*O*-benzyl-α-D-glucopyranoside (8h)

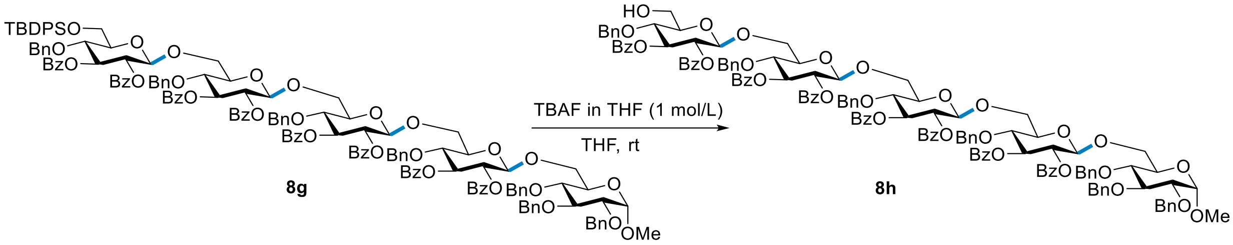

To a solution of **8g** (115.5 mg, 0.04500 mmol) in anhydrous THF (3.0 mL) was added TBAF/THF (1.0 mol/L, 0.068 mL, 0.068 mmol) under Air atmosphere. Then the mixture was stirred at room temperature and the progress of the reaction was monitored by TLC. Upon completion, the reaction mixture was concentrated in vacuo. The residue was purified by flash column chromatography on silica gel (petroleum ether/EtOAc = 3/1) to obtained **8h** as a colorless syrup (86.8 mg, 83%). $[a]_D^{25}$: 17.50 (*c*: 0.16 $CHCl_3$). **^{1}H NMR** (400 MHz, $CDCl_3$) δ 8.01 - 7.77 (m, 15H), 7.57 - 7.25 (m, 21H), 7.25 - 6.74 (m, 39H), 5.90 - 5.61 (m, 4H), 5.58 - 5.39 (m, 4H), 5.03 (d, *J* = 7.7 Hz, 1H), 4.85 (d, *J* = 10.9 Hz, 1H), 4.80 - 4.49 (m, 9H), 4.44 - 4.09 (m, 11H), 4.07 - 3.57 (m, 17H), 3.44 - 3.40 (m, 2H), 3.27 (s, 3H), 2.78 (br, 1H). **^{13}C NMR** (101 MHz, $CDCl_3$) δ 165.7, 165.65, 165.61, 165.4, 165.2, 164.9, 164.7, 138.9, 138.4, 138.2, 137.6, 137.2, 136.9, 136.8, 133.18, 133.13, 133.08, 133.02, 132.8, 132.7, 132.6, 129.9, 129.8, 129.79, 129.74, 129.6, 129.5, 129.49, 129.47, 129.41, 129.3, 128.4, 128.36, 128.32, 128.30, 128.29, 128.25, 128.17, 128.13, 128.10, 128.07, 128.00, 127.9, 127.8, 127.79, 127.75, 127.5, 127.3, 127.2, 103.0, 102.3, 101.3, 100.5, 97.9, 81.9, 79.7, 77.4, , 76.8, 76.5, 76.0, 75.7, 75.5, 75.4, 75.3, 75.05, 75.02, 74.9, 74.8, 74.7, 74.5, 74.1, 73.8, 73.2, 72.8, 72.5, 72.16, 72.11, 70.4, 70.2, 69.4, 69.3, 67.7, 61.5, 55.2. **HRMS** (ESI) Calculated for $C_{136}H_{132}NO_{34}$ $[M+NH_4]^+$: 2322.8631 Found: 2322.8608.

Methyl (2,3-di-*O*-benzoyl-4-*O*-benzyl-6-*O*-tert-butyldiphenylsilyl-β-D-glucopyranosyl)-(1 → 6)-(2,3-di-*O*-benzoyl-4-*O*-benzyl-β-D-glucopyranosyl)-(1 → 6)-(2,3-di-*O*-benzoyl-4-*O*-benzyl-β-D-glucopyranosyl)-(1 → 6)-(2,3-di-*O*-benzoyl-4-*O*-benzyl-β-D-glucopyranosyl)-(1 → 6)-(2,3-di-*O*-benzoyl-4-*O*-benzyl-β-D-glucopyranosyl)-(1→6)-2,3,4-tri-*O*-benzyl-α-D-glucopyranoside (8i)

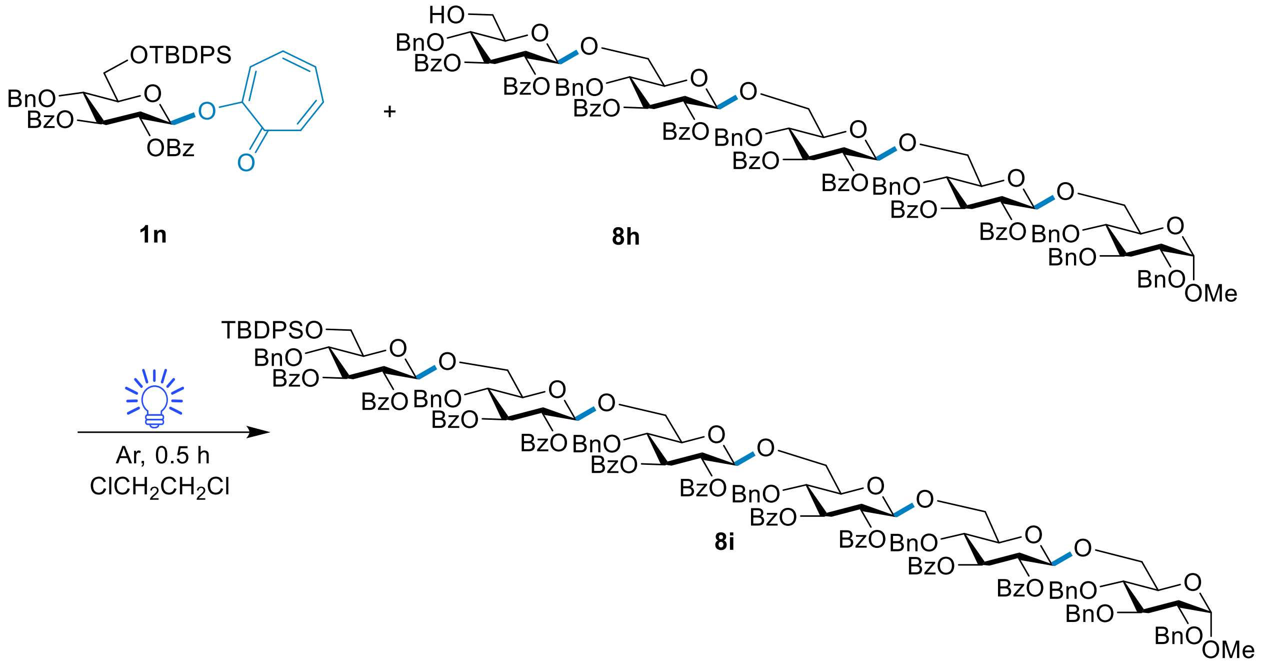

The glycosyl donor **1n** (45.5 mg, 0.0560 mmol), acceptor **8h** (85.2 mg, 0.0370 mmol) and TMSOTf (0.67 uL, 0.0037 mmol) were dissolved in dry $ClCH_2CH_2Cl$ (4.0 mL), and the mixture was irradiated by blue LEDs at ambient temperature for 0.5 h. After the reaction was completed, the solvent was concentrated under reduced pressure. The resulting residue was eluted by flash column chromatography (petroleum ether/EtOAc = 4/1) to afford the glycosylated product **8i** as a colorless syrup (84.4 mg, 76%). $[\alpha]_D^{25}$: 26.00 (*c*: 0.15 $CHCl_3$). **^{1}H NMR** (400 MHz, $CDCl_3$) δ 8.31 - 7.89 (m, 17H), 7.71 - 6.50 (m, 81H), 6.34 - 6.30 (m, 2H), 6.10 - 5.29 (m, 13H), 5.07 - 3.43 (m, 45H), 3.38 (s, 3H), 1.16 (s, 9H). **^{13}C NMR** (101 MHz, $CDCl_3$) δ 165.78, 165.74, 165.71, 165.6, 165.3, 164.6, 164.5, 139.0, 138.5, 138.3, 137.7, 137.3, 136.9, 136.6, 135.69, 135.60, 133.6, 133.1, 133.0, 132.99, 132.95, 132.77, 132.73, 132.6, 132.5, 132.3, 132.2, 131.6, 130.5, 130.2, 130.09, 130.02, 129.9, 129.86, 129.81, 129.7, 129.69, 129.67, 129.61, 129.55, 129.51, 129.4, 128.7, 128.5, 128.48, 128.46, 128.43, 128.38, 128.34, 128.25, 128.24, 128.1, 128.06, 128.02, 127.95, 127.93, 127.90, 127.8, 127.77, 127.74, 127.69, 127.67, 127.63, 127.5, 127.4, 127.3, 127.25, 127.20, 127.1, 126.8, 104.1, 103.8, 102.4, 101.8, 100.1, 97.7, 82.0, 79.8, 79.6, 79.0, 78.8, 78.6, 78.3, 78.0, 76.8, 76.5, 76.4, 75.9, 75.8, 75.4, 75.1, 75.0, 74.9, 74.8, 74.4, 74.3, 73.6, 73.5, 73.1, 73.0, 72.8, 72.7, 72.49, 72.40, 72.2, 71.8, 69.6, 67.5, 63.7, 55.2, 27.0, 19.1. **HRMS** (ESI) Calculated for $C_{179}H_{174}NO_{41}Si$ $[M+NH_4]^+$: 3021.1331 Found: 3021.1362.

6. 2-Glycosyloxytropone as the acceptor

6.1 Synthesis of compound S27

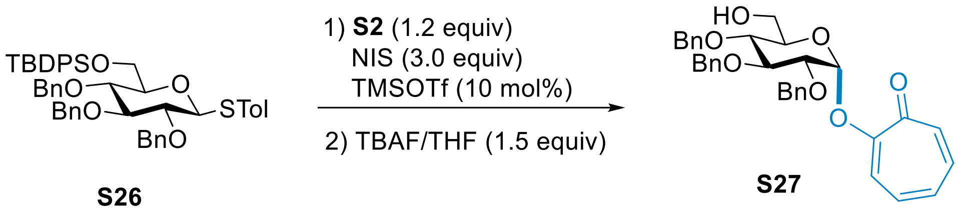

To a solution of **S26**[26] (1.59 g, 2.00 mmol), **S2** (292.8 mg, 2.400 mmol) and NIS (1.35 g, 6.00 mmol) in dry CH_2Cl_2 (15.0 mL), TMSOTf (36.2 uL, 0.200 mmol) was added slowly at 0°C under argon atmosphere. Then the mixture was stirred at room temperature and monitored by TLC. The reaction was quenched by saturated $Na_2S_2O_3$ solution, diluted with CH_2Cl_2, and washed with water (3×20 mL). The combined organic layers were dried over Na_2SO_4 and filtered. The solvent was removed in vacuo. The residue was dissolved in THF (10.0 mL), and TBAF/THF (1.0 mol/L, 1.0 mL) was added to the mixture. Then the mixture was stirred at room temperature and the progress of the reaction was monitored by TLC. Upon completion, the reaction mixture was concentrated in vacuo. The residue was purified by flash column chromatography on silica gel (petroleum ether/EtOAc = 2/1) to obtain product **S27** as a yellow syrup (554.2 mg, 50%). $[\alpha]_D^{25}$: 160.71 (*c*: 0.14 $CHCl_3$). **^{1}H NMR** (400 MHz, $CDCl_3$) δ 7.42 - 7.26 (m, 15H), 7.20 - 7.19 (m, 2H), 7.13 - 7.04 (m, 1H), 6.99 - 6.87 (m, 2H), 5.96 (d, *J* = 3.6 Hz, 1H), 5.07 (d, *J* = 10.9 Hz, 1H), 4.94 - 4.80 (m, 4H), 4.65 (d, *J* = 11.0 Hz, 1H), 4.28 (t, *J* = 9.3 Hz, 1H), 3.86 (dt, *J* = 10.1, 3.4 Hz, 1H), 3.70 - 3.57 (m, 4H). **^{13}C NMR** (151 MHz, $CDCl_3$) δ 181.0, 162.1, 139.2, 138.7, 138.0, 137.9, 136.1, 132.2, 130.9, 128.48, 128.42, 128.3, 128.2, 128.0, 127.99, 127.91, 127.8, 127.6, 123.2, 94.7, 81.2, 79.5, 75.7, 75.0, 73.0, 72.4, 61.5. **HRMS** (ESI) Calculated for $C_{34}H_{34}NaO_7$ $[M+Na]^+$: 577.2202 Found: 577.2201.

6.2 Glycosylation reaction between 2-glycosyloxytropone with thioglycoside

Method A:

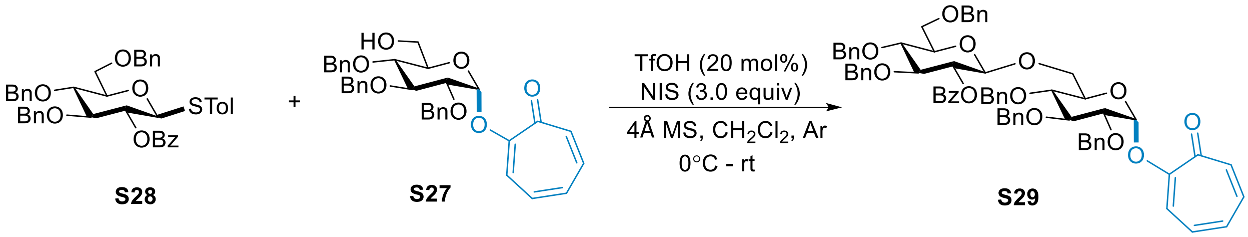

To a mixture of **S28**[27] (33.0 mg, 0.0500 mmol), **S27** (25.0 mg, 0.0450 mmol), NIS (33.8 mg, 0.150 mmol) and 4Å MS (400.0 mg) in dry CH_2Cl_2 (4.0 mL), TfOH (0.44 uL, 0.010 mmol) was added slowly at 0°C under argon atmosphere. Then the mixture was stirred at room temperature and monitored by TLC (~ 4 h). The reaction was quenched by saturated $Na_2S_2O_3$ solution, diluted with CH_2Cl_2, and washed with water (3×10 mL). The combined organic layers were dried over Na_2SO_4 and filtered, and the solvent was removed in vacuo. The residue was purified by flash column chromatography on silica gel (petroleum ether/EtOAc = 3/1) to obtain product **S29** as a colorless oil (26.0 mg, 53%). $[\alpha]_D^{25}$: 65.45 (*c*: 0.11 $CHCl_3$). **^{1}H NMR** (400 MHz, $CDCl_3$) δ 7.94 - 7.89 (m, 2H), 7.45 - 7.41

(m, 1H), 7.35 - 7.27 (m, 11H), 7.25 - 7.09 (m, 21H), 7.01 - 6.97 (m, 3H), 6.86 - 6.83 (m, 2H), 5.59 (d, J = 3.5 Hz, 1H), 5.31 (dd, J = 9.4, 8.0 Hz, 1H), 4.95 (d, J = 11.1 Hz, 1H), 4.82 - 4.69 (m, 5H), 4.65 (d, J = 11.1 Hz, 1H), 4.58 - 4.49 (m, 3H), 4.47 - 4.38 (m, 2H), 4.27 - 4.17 (m, 2H), 4.01 (dd, J = 10.8, 2.0 Hz, 1H), 3.86 (dt, J = 10.1, 2.6 Hz, 1H), 3.79 (t, J = 9.1 Hz, 1H), 3.75 - 3.63 (m, 4H), 3.59 (dd, J = 9.7, 3.5 Hz, 1H), 3.53 - 3.46 (m, 2H). **^{13}C NMR** (101 MHz, $CDCl_3$) δ 180.7, 165.0, 162.4, 138.9, 138.8, 138.3, 138.19, 138.13, 137.8, 137.7, 135.9, 133.0, 132.3, 130.1, 129.8, 129.7, 128.44, 128.42, 128.39, 128.37, 128.28, 128.22, 128.1, 128.0, 127.9, 127.87, 127.84, 127.73, 127.71, 127.69, 127.61, 127.4, 127.3, 120.8, 101.2, 95.3, 82.7, 81.1, 79.5, 78.0, 77.2, 75.49, 75.44, 75.1, 75.0, 74.5, 73.6, 73.4, 73.1, 71.0, 68.8, 67.5. **HRMS** (ESI) Calculated for $C_{68}H_{66}NaO_{13}$ $[M+Na]^+$: 1113.4401 Found: 1113.4436.

Method B:

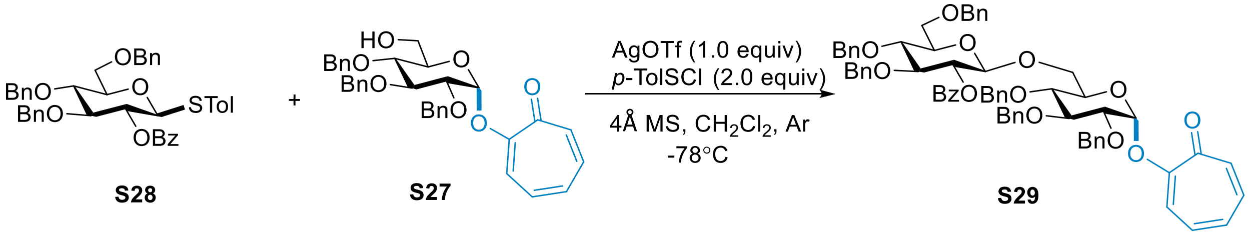

A mixture of **S28**[27] (33.0 mg, 0.0500 mmol) and activated 4Å MS (400.0 mg) in dry CH_2Cl_2 (2.0 mL) under argon atmosphere was cooled to -78°C. After 10 minutes, *p*-TolSCl (13.2 μL, 0.100 mmol) was added, followed by addition of AgOTf (12.8 mg, 0.0500 mmol, dissolved in anhydrous toluene/CH_2Cl_2 = 0.3 mL/0.1 mL). 10 minutes later, a solution of **S27** (25.0 mg, 0.0450 mmol) in anhydrous CH_2Cl_2 (2.0 mL) was slowly added. The resulting mixture was stirred at -78°C for 10 min, and then filtered through Celite. After removal of the solvent, the residue was purified by flash column chromatography on silica gel (petroleum ether/EtOAc = 3/1) to obtain product **S29** as a colorless oil (39.0 mg, 80%).

6.3 Glycosylation reaction between 2-glycosyloxytropone with glycosyl imidates

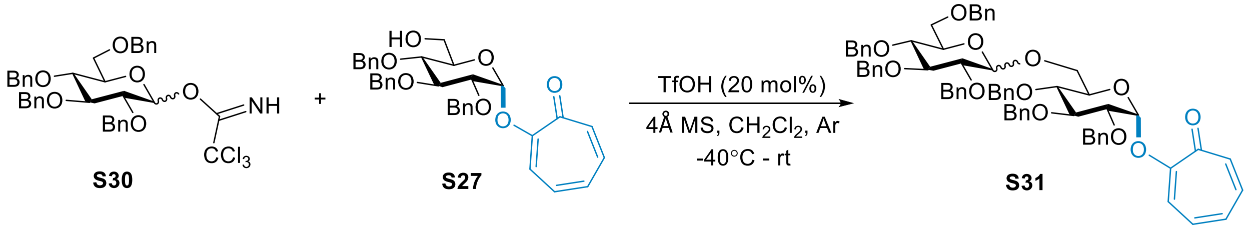

To a mixture of **S30**[28] (41.1 mg, 0.0600 mmol), **S27** (27.7 mg, 0.0500 mmol) and 4Å MS (400.0 mg) in dry CH_2Cl_2 (4.0 mL), TfOH (0.88 uL, 0.010 mmol) was added slowly at -40℃ under argon atmosphere. The mixture was stirred at room temperature and monitored by TLC. Then the solvent was removed in vacuo, the residue was

purified by flash column chromatography on silica gel (petroleum ether/EtOAc = 3/1) to obtain product **S31** as a colorless oil (α/β =1/1.1, 26.6 mg, 50%). **^{1}H NMR** (400 MHz, $CDCl_3$) δ 7.42 - 7.02 (m, 60.8H), 6.90 - 6.85 (m, 0.5H), 6.83 - 6.76 (m, 2H), 6.74 - 6.69 (m, 0.4H), 5.87 (d, *J* = 3.5 Hz, 1H), 5.77 (d, *J* = 3.5 Hz, 0.4H), 5.07 - 5.04 (m, 1.5H), 4.96 - 4.07 (m, 12.6H), 4.67 - 4.46 (m, 7.5H), 4.43 - 4.39 (m, 1H), 4.35 - 4.23 (m, 2.6H), 4.11 (dd, *J* = 10.8, 2.0 Hz, 1H), 4.09 - 3.98 (m, 1.6H), 3.84 - 3.46 (m, 12.6H), 3.43 - 3.35 (m, 2H). **^{13}C NMR** (101 MHz, $CDCl_3$) δ 180.98, 180.91, 162.3, 162.2, 139.1, 138.87, 138.84, 138.81, 138.4, 138.38, 138.34, 138.31, 138.29, 138.23, 138.1, 138.0, 137.9, 136.0, 135.8, 132.4, 132.1, 130.6, 130.5, 128.4, 128.38, 128.37, 128.36, 128.32, 128.29, 128.28, 128.1, 128.02, 128.00, 127.92, 127.90, 127.8, 127.75, 127.70, 127.67, 127.64, 127.60, 127.56, 127.54, 127.50, 122.9, 122.7, 103.8, 97.3, 94.8, 94.7, 84.7, 82.0, 81.7, 81.3, 81.2, 80.0, 79.7, 79.4, 77.8, 77.5, 77.5, 77.2, 75.7, 75.5, 75.0, 74.97, 74.91, 74.8, 73.43, 73.40, 72.9, 72.5, 71.9, 71.5, 70.2, 68.9, 68.6, 68.4, 66.3. **HRMS** (ESI) Calculated for $C_{68}H_{68}NaO_{12}$ $[M+Na]^+$: 1099.4608 Found: 1099.4626.

7. Mechanism experiments

7.1 The effect of 1,1-diphenylethylene

Supplementary Table 6. The Effect of 1,1-diphenylethylene on the reaction of compound 1a with 2a [a]

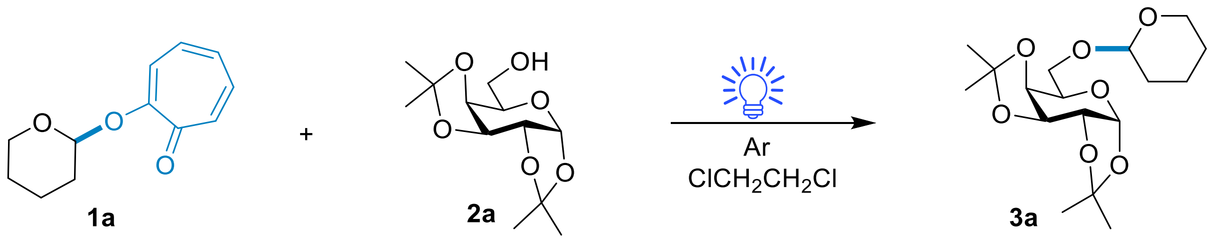

Entry	Additives	Time (h)	Yield (%)^b
1	1,1-diphenylethylene (0.1 equiv)	3 h	40
2	1,1-diphenylethylene (0.5 equiv)	3 h	24
3	1,1-diphenylethylene (1.0 equiv)	3 h	22
4	1,1-diphenylethylene (2.0 equiv)	3 h	16

[a] Reaction conditions: **1a** (0.075 mmol), **2a** (0.05 mmol), additives, $ClCH_2CH_2Cl$ (2.0 mL), blue LEDs (15 W), 3 h, Ar atmosphere. [b] Yields were determined by ^{1}H NMR.

7.2 Absorption and fluorescence spectra of compound 1a

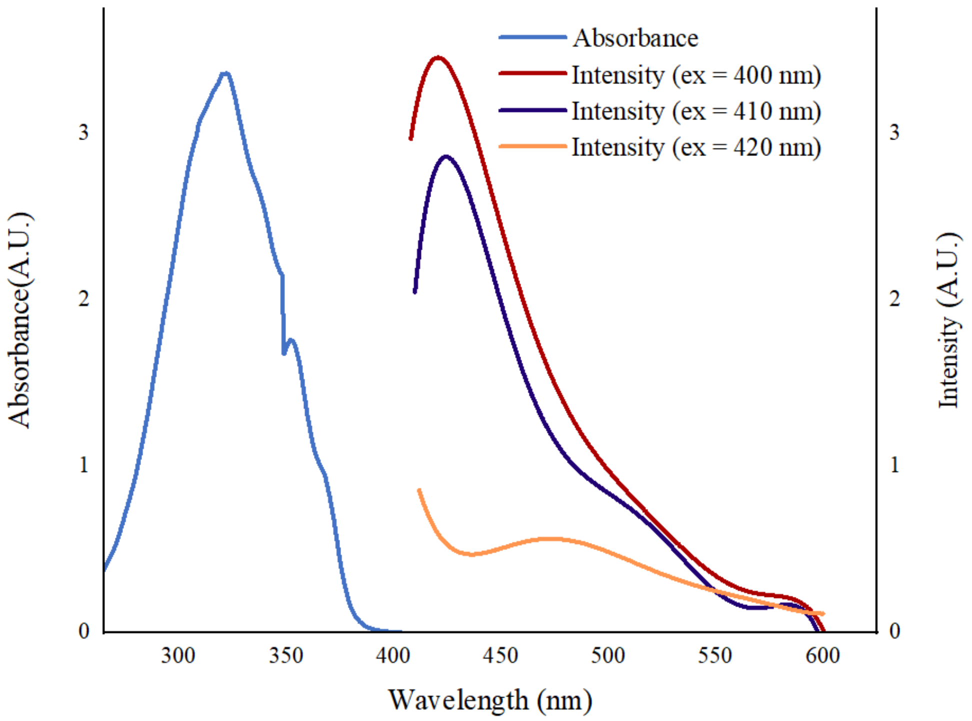

Supplementary Figure 1. UV−vis absorption and photoluminescence spectra of **1a** (0.1 mg/mL)

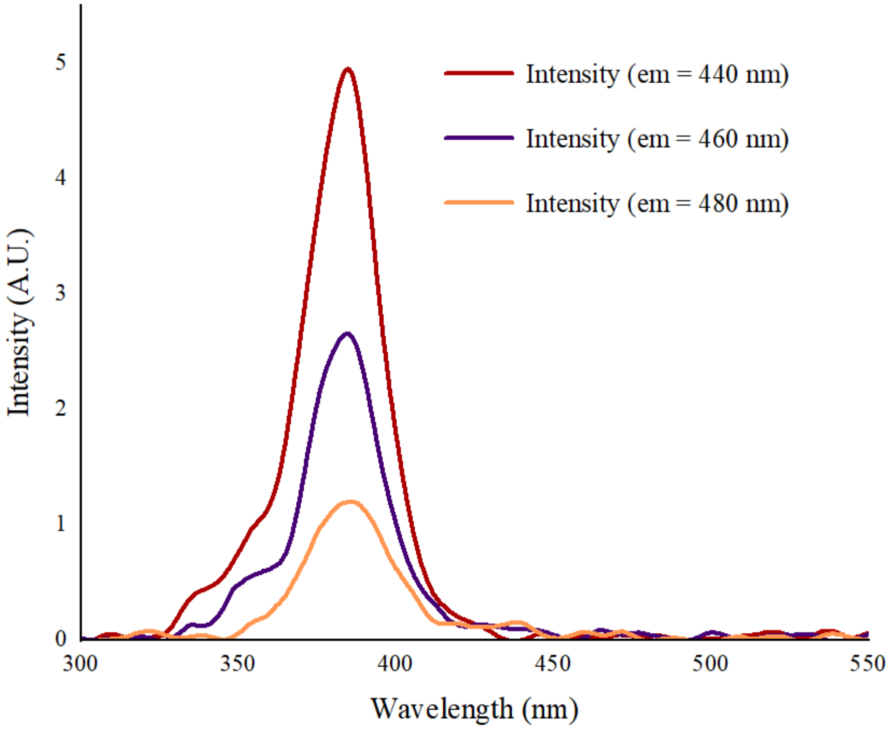

Supplementary Figure 2. Photoluminescence excitation spectra of **1a** (0.1 mg/mL)

7.3 Decomposition experiments of the glycosyl donor in the presence of light.

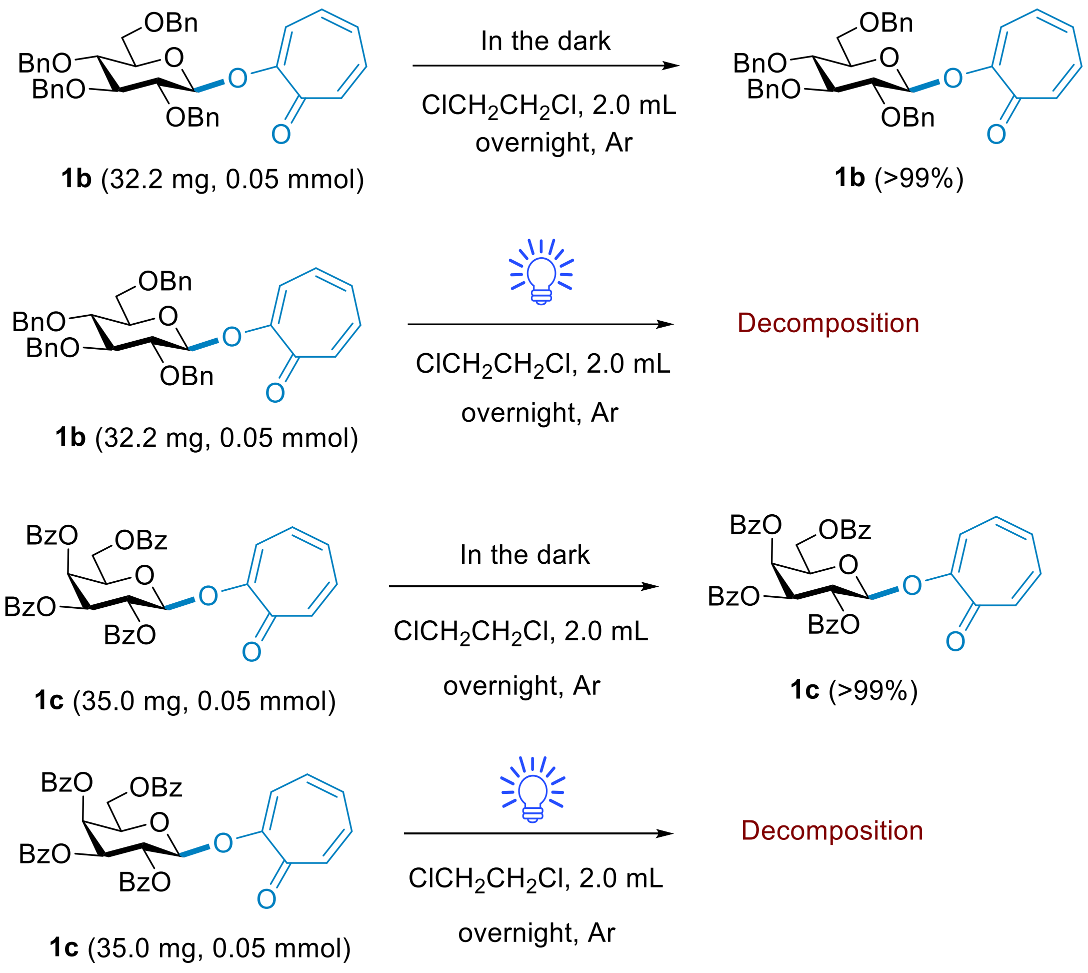

Supplementary Figure 3. Decomposition experiments of the donor **1b** and **1c** in the absence/presence of light.

7.4 Light on and Light off experiments

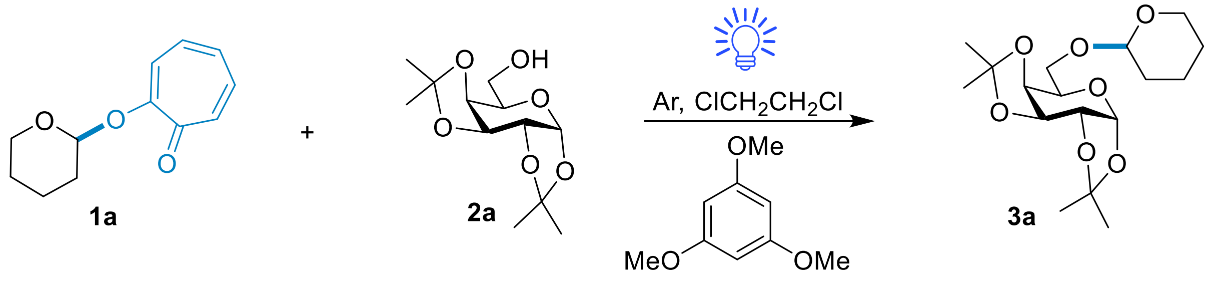

The donor **1a** (31.0 mg, 0.150 mmol) and the acceptor **2a** (26.0 mg, 0.100 mmol) were dissolved in dry $ClCH_2CH_2Cl$ (4.0 mL), and the mixture was irradiated by Blue LEDs for 0-20 min, 40-60 min,80-100 min, 120-140 min, 160-180 min, 200-220 min, 240-260 min, and 280-300 min (other time were in dark). The system was determined by ^{1}H NMR analysis every 20 min until 300 min. The 1,3,5-trimethoxybenzene was used as the internal standard substance for determining the yield of compound **3a** in this system. For example, at 220 min,

compound **3a** was obtained in 65% yield. More detailed data were shown in Supplementary Table 7, Figure 4 and Figure 5.

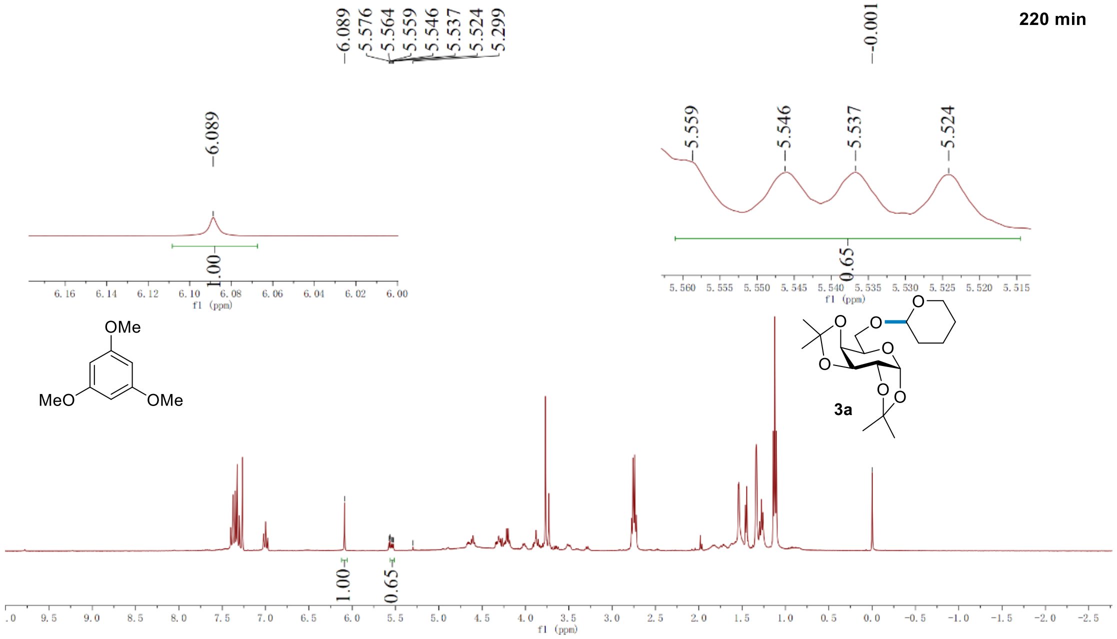

Supplementary Figure 4. ^{1}H NMR of the reaction of **1a** with **2a**

Supplementary Table 7. Light on and light off experiments using the reaction of 1a with 2a

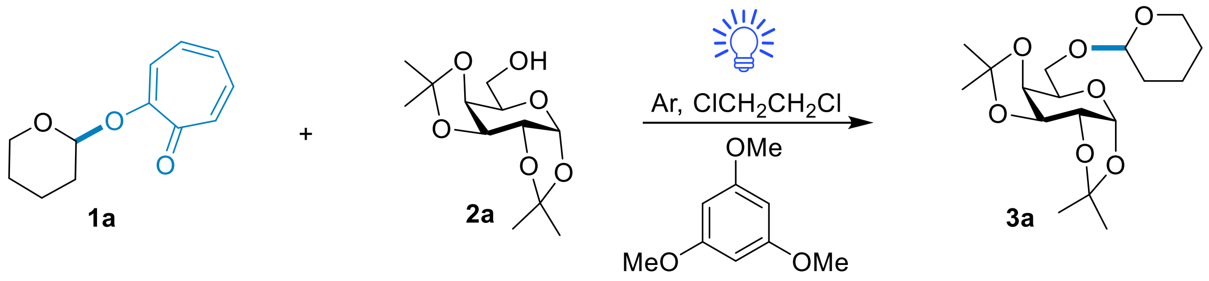

Entry	Time (min)	Yield of 3a (%)[a]
1	20	4
2	40	4
3	60	16
4	80	16

5	100	33
6	120	33
7	140	52
8	160	52
9	180	62
10	200	62
11	220	65
12	240	65
13	260	66
14	280	66
15	300	66

[a] Yields were determined by ^{1}H NMR

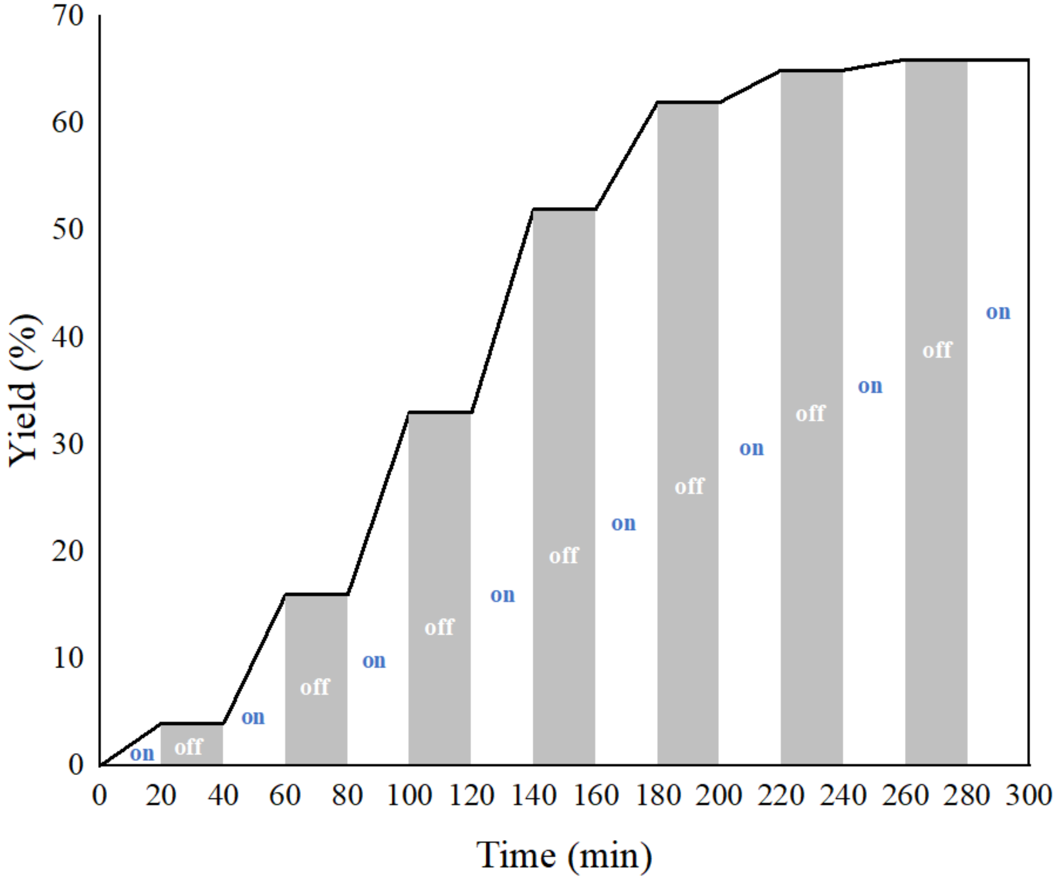

Supplementary Figure 5. Light on and light off experiments

Based on the light on and light off experiments mentioned above, we observed that the reaction rate was lower during the 0-20 minutes compared to the 40-60 minutes, 80-100 minutes and 120-140 minutes. This might be due to the low content of free tropolone in the mixture at the beginning of the reaction. To verify if tropolone has an impact on the reaction rate, we conducted the following experiments.

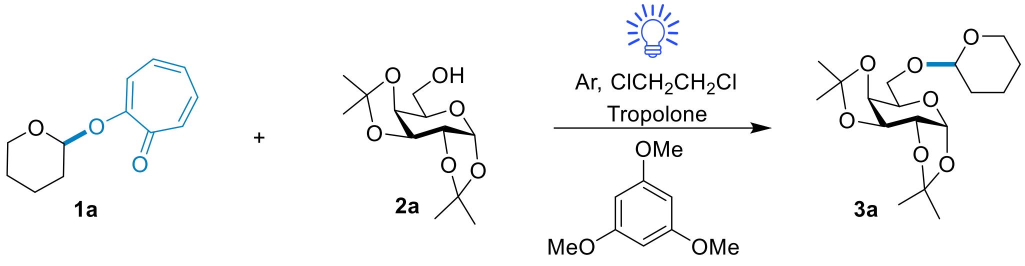

The donor **1a** (15.5 mg, 0.0750 mmol), acceptor **2a** (13.0 mg, 0.0500 mmol) and tropolone were dissolved in dry $ClCH_2CH_2Cl$ (4.0 mL), and the mixture was irradiated by Blue LEDs for 20 mins. The system was determined by ^1H NMR analysis. The 1,3,5-trimethoxybenzene was used as the

internal standard substance for determining the yield of compound **3a** in this system. According to the experimental results, we found that the addition of a catalytic amount of tropolone indeed improves the yield of the reaction. More detailed data were shown in Supplementary Table 8 and Figure 6.

Supplementary Table 8. The effect of tropolone on the reaction of **1a** with **2a**.[a]

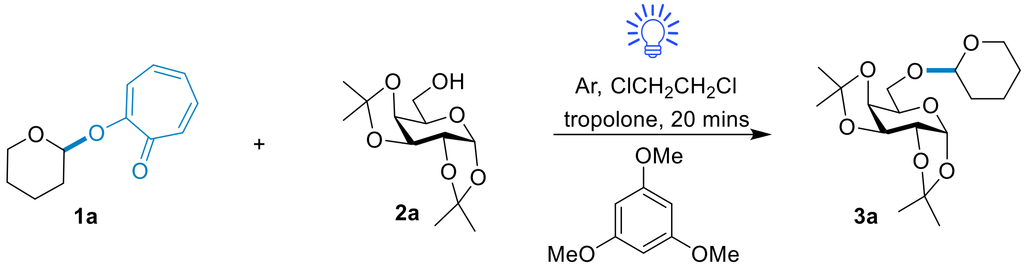

**Entry**	**Tropolone (equiv.)**	**Yield of 3a (%)**[b]
1	0	4
2	0.1	8
3	0.3	11
4	0.5	12

[a] Reaction conditions: **1a** (0.075 mmol), **2a** (0.05 mmol), irradiation wavelengths, $ClCH_2CH_2Cl$ (2.0 mL), argon atmosphere. [b] Isolated yields.

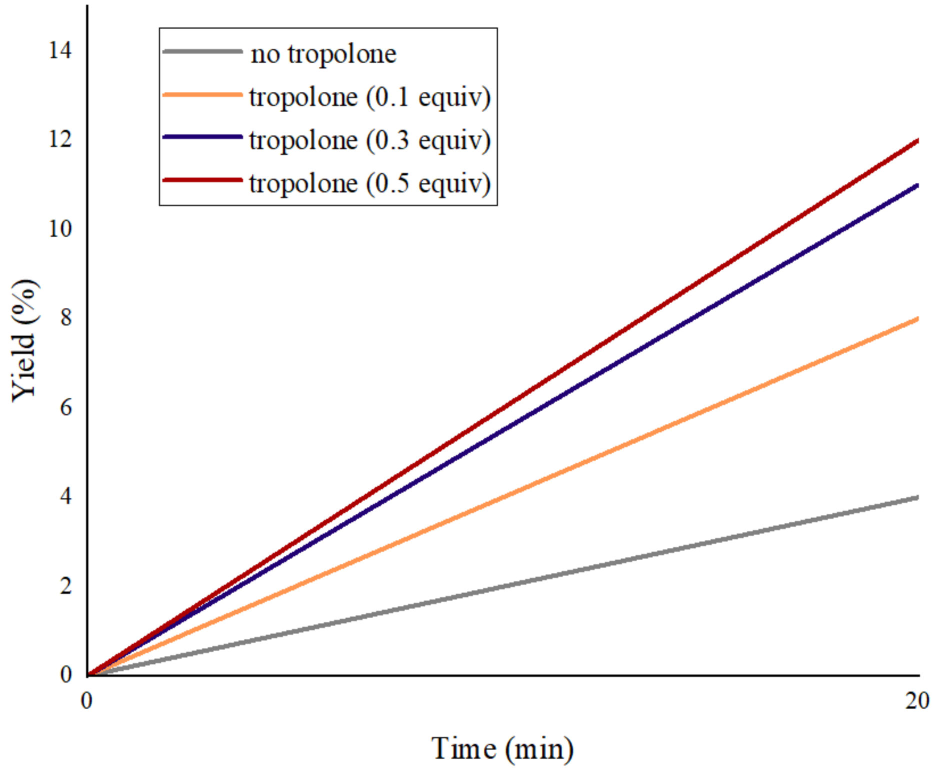

Supplementary Figure 6. The yield of reaction of **1a** with **2a** for 20 minutes

7.5 Proposed Mechanism

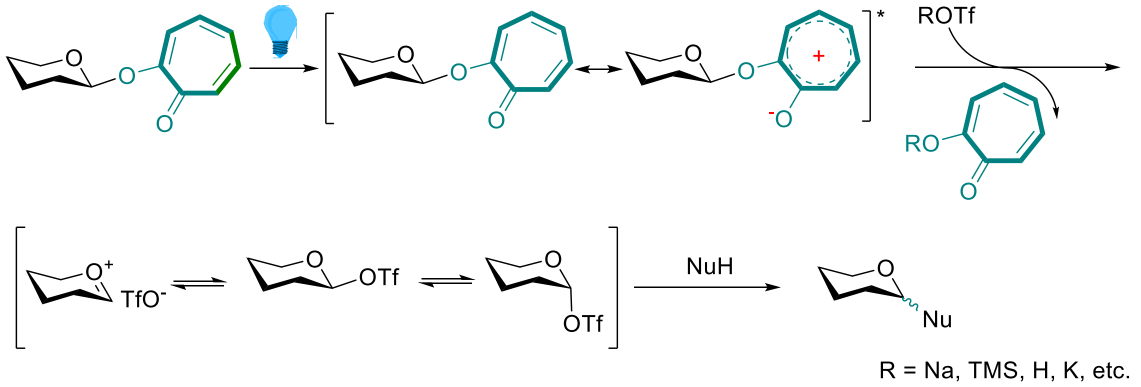

Supplementary Figure 7. Proposed Mechanism

8. References

[1] Lyons, D. J. M., Empel, C., Pace, D. P., Dinh, A. H., Mai, B. K., Koenigs, R. M. & Nguyen, T. V. Tropolonate salts as acyl-transfer catalysts under thermal and photochemical conditions: reaction scope and mechanistic insights. *ACS Catal.* **10**, 12596-12606 (2020).

[2] Lu, S.-R., Lai, Y.-H., Chen, J.-H., Liu, C.-Y. & Mong, K.-K. T. Dimethylformamide: an unusual glycosylation modulator. *Angew. Chem. Int. Ed.* **50**, 7315 -7320 (2011).

[3] Liang, X.-Y., Deng, L.-M., Liu, X. & Yang, J.-S. Efficient one-pot syntheses of α-D-arabinofuranosyl tri- and tetrasaccharides present in cell wall polysaccharide of Mycobacterium tuberculosis. *Tetrahedron* **66**, 87-93 (2010).

[4] Chang, C.-W., Lin, M-H., Chan, C.-K., Su, K.-Y., Wu, C.-H., Lo, W.-C., Lam, S., Cheng, Y.-T., Liao, P.-H., Wong, C.-H. & Wang, C.-C. Automated quantification of hydroxyl reactivities: prediction of glycosylation reactions. *Angew. Chem. Int. Ed.* **60**, 12413 -12423 (2021).

[5] Hu, J.-C., Feng, A.-F. W., Chang, B.-Y., Lin, C.-H. & Mong, K.-K. T. A flexible 1,2-cis α-glycosylation strategy based on in situ adduct transformation. *Org. Biomol. Chem.* **15**, 5345-5356 (2017).

[6] Koto, S., Takebe, Y. & Zen, S. The Synthesis of methyl 2,4,6-tri-*O*-benzyl-α-D-glucopyranoside. *B. Chem. Soc. Jap.* **45**, 291-293 (1972).

[7] Lecourt, T., Herault, A., Pearce, A. J., Sollogoub, M. & Sinaÿ, P. Triisobutylaluminium and diisobutylaluminium hydride as molecular scalpels: the regioselective stripping of perbenzylated sugars and cyclodextrins. *Chem. Eur. J.* **10**, 2960-2971 (2004).

[8] Balmond, E. I., Coe, D. M., Galan, M. C. & McGarrigle, E. M. a-Selective organocatalytic synthesis of 2-deoxygalactosides. *Angew. Chem. Int. Ed.* **51**, 9152 -9155 (2012).

[9] Zhang, X., Yang, Y., Ding, J., Zhao, Y., Zhang, H., & Zhu, Y. Stereoselective gold(I)-catalyzed approach to the synthesis of complex α-glycosyl phosphosaccharides. *Nat. Commun.* **13**, 421 (2022).

[10] Smajlagic, I., Durán, R., Pilkington, M. & Dudding, T. Cyclopropenium enhanced thiourea catalysis. *J. Org. Chem.* **83**, 13973-13980 (2018).

[11] Wang, H. Y., Simmons, C. J., Blaszczyk, S. A., Balzer, P. G., Luo, R., Duan, X. & Tang, W. Isoquinoline-1-carboxylate as a traceless leaving group for chelation-assisted glycosylation under mild and neutral reaction conditions. *Angew. Chem. Int. Ed.* **56**, 15698-15702 (2017).

[12] Shaikh, A. Y., Sureshkumar, G., Pati, D., Gupta, S. S. & Hotha, S. Facile synthesis of unusual glycosyl carbamates and amino acid glycosides from propargyl 1,2-orthoesters as glycosyl donors. *Org. Biomol.Chem.* **9**, 5951-5959 (2011).

[13] Liu, M., Li, B. H., Xiong, D. C. & Ye, X. S. *O*-Glycosylation Enabled by *N*-(Glycosyloxy)acetamides. *J. Org. Chem.* **83**, 8292-8303 (2018).

[14] Mao, R.-Z., Xiong, D.-C., Guo, F., Li, Q., Duan, J. & Ye, X.-S. Light-driven highly efficient glycosylation reactions. *Org. Chem. Front.* **3**, 737-743 (2016).

[15] Singh, Y. & Demchenko, A. V. Defining the scope of the acid-catalyzed glycosidation of glycosyl bromides. *Chem. Eur. J.* **26**, 1042-1051 (2020).

[16] Sui, J.-J., Xiong, D.-C. & Ye, X.-S. Copper-mediated O-arylation of lactols with aryl boronic acids. *Chin. Chem. Lett.* **30**, 1533-1537 (2019).

[17] Liu, D.-K., Xiong, D.-C., Wu, X., Li, Q. & Ye, X.-S. Rapid glycosylation of 2′-benzoylphenyl glycosides promoted by TfOH. *Org. Chem. Front.* **6**, 2756-2759 (2019).

[18] Kamkhachorn, T., Parameswar, A. R. & Demchenko, A. V. Comparison of the armed/disarmed building blocks of the D-gluco and D-glucosamino series in the context of chemoselective oligosaccharide synthesis. *Org. Lett.* **12**, 3078-3081 (2010).

[19] Mukhopadhyay, B., Maurer, S. V., Rudolph, N., Well, R. M., Russell, D. A. & Field, R. A. From solution phase to "on-column" chemistry: trichloroacetimidate-based glycosylation promoted by perchloric acid-silica. *J. Org. Chem.* **70**, 9059-9062 (2005).

[20] Garcia, B. A. & Gin, D. Y. Synthesis of glycosyl-1-phosphates via dehydrative glycosylation. *Org. Lett.* **2**, 2135-2138 (2000).

[21] Koshiba, M., Suzuki, N., Arihara, R., Tsuda, T., Nambu, H., Nakamura, S. & Hashimoto, S. Catalytic stereoselective glycosidation with glycosyl diphenyl phosphates: rapid construction of 1,2-cis-α-glycosidic linkages. *Chem. Asian J.* **3**, 1664-1677 (2008).

[22] Tsuda, T., Nakamura, S. & Hashimoto, S. A highly stereoselective construction of 1,2-trans-β-glycosidic linkages capitalizing on 2-azido-2-deoxy-d-glycosyl diphenyl phosphates as glycosyl donors. *Tetrahedron* **60**, 10711-10737 (2004).

[23] Liu, G.-J., Zhang, X.-T. & Xing, G.-W. A general method for *N*-glycosylation of nucleobases promoted by $(p\text{-Tol})_2SO/Tf_2O$ with thioglycoside as donor. *Chem. Commun.* **51**, 12803-12806 (2015).

[24] Sniady, A., Bedore, M. W. & Jamison, T. F. One-flow, multistep synthesis of nucleosides by brønsted acid-catalyzed glycosylation. *Angew. Chem. Int. Ed.* **50**, 2155-2158 (2011).

[25] Liang, H., Ma, L., Li, C., Peng, Q., Wang, Z., Zhang, Z.-X., Yu, L., Liu, H., An, F. & Xue, W. Efficient glycosylation with glycosyl *ortho*-allylbenzoates as donors. *Tetrahedron Lett.* **60**, 84-87 (2019).

[26] Huang, X., Huang, L., Wang, H. & Ye, X.-S. Iterative one-pot synthesis of oligosaccharides. *Angew. Chem. Int. Ed.* **43**, 5221-5224 (2004).

[27] Premathilake, H. D., Mydock, L. K. & Demchenko, A.V. Superarming common glycosyl donors by simple 2-*O*-benzoyl-3,4,6-tri-*O*-benzyl protection. *J. Org. Chem.* **75**, 1095-1100 (2010).

[28] Shaw, M. & Kumar, A. Visible-light-mediated β-C(sp^3)-H amination of glycosylimidates: En Route to oxazoline-fused/spiro nonclassical bicyclic sugars. *Org. Lett.* **21**, 3108-3113 (2019).

8. NMR spectra

^{1}H NMR Spectrum of **1a** (400 MHz, $CDCl_3$)

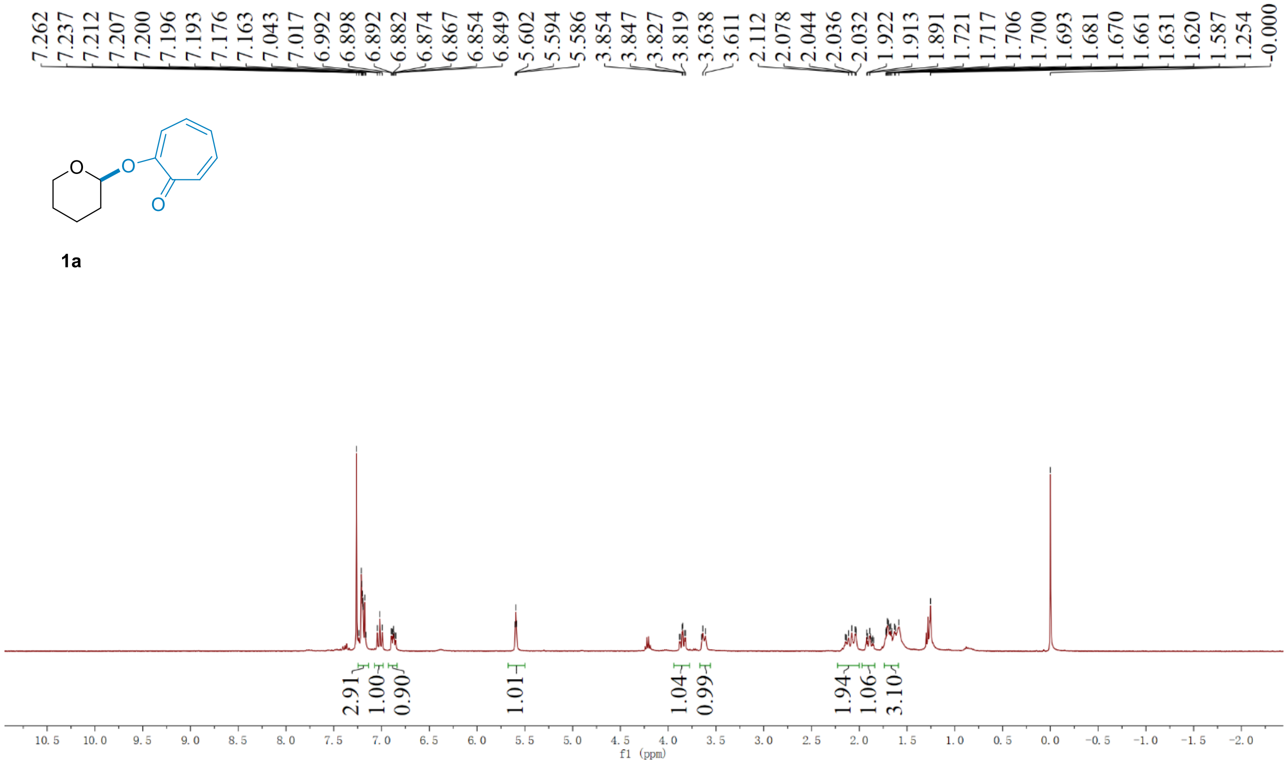

^{13}C NMR Spectrum of **1a** (101 MHz, $CDCl_3$)

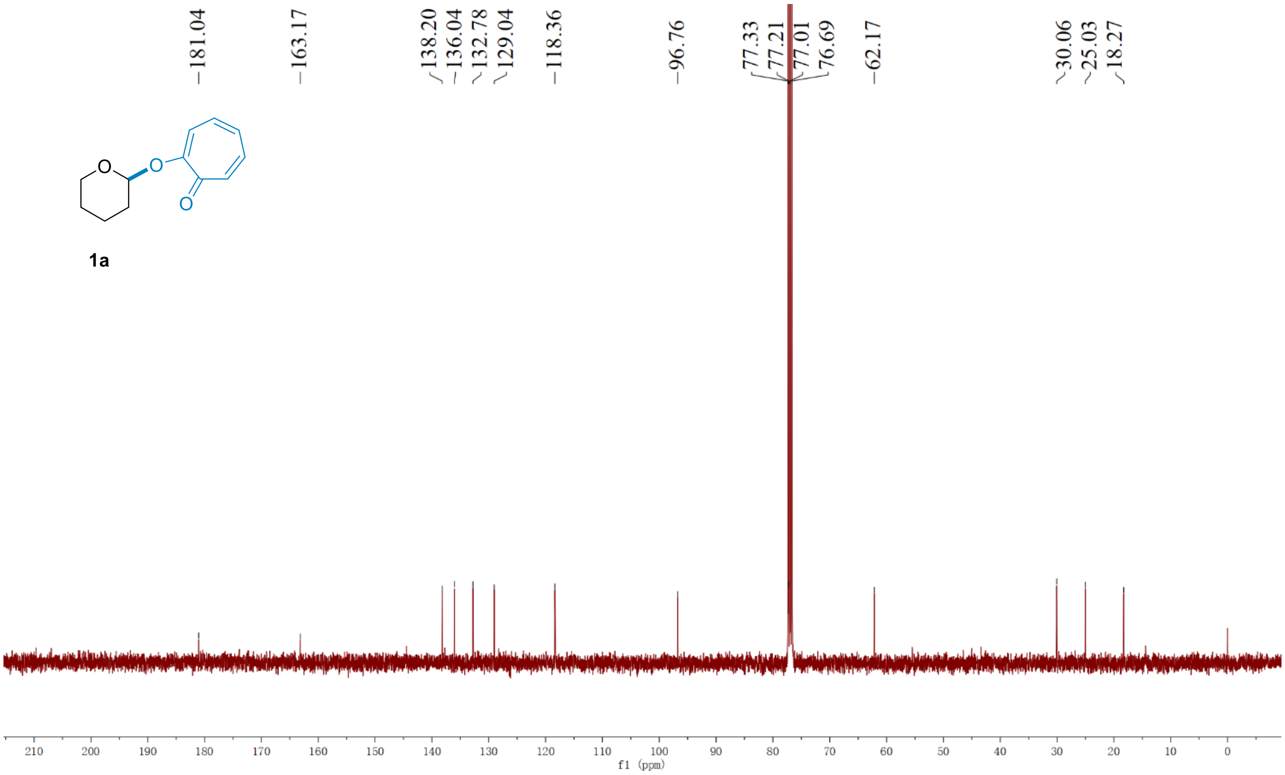

^{1}H NMR Spectrum of **1b** (400 MHz, $CDCl_3$)

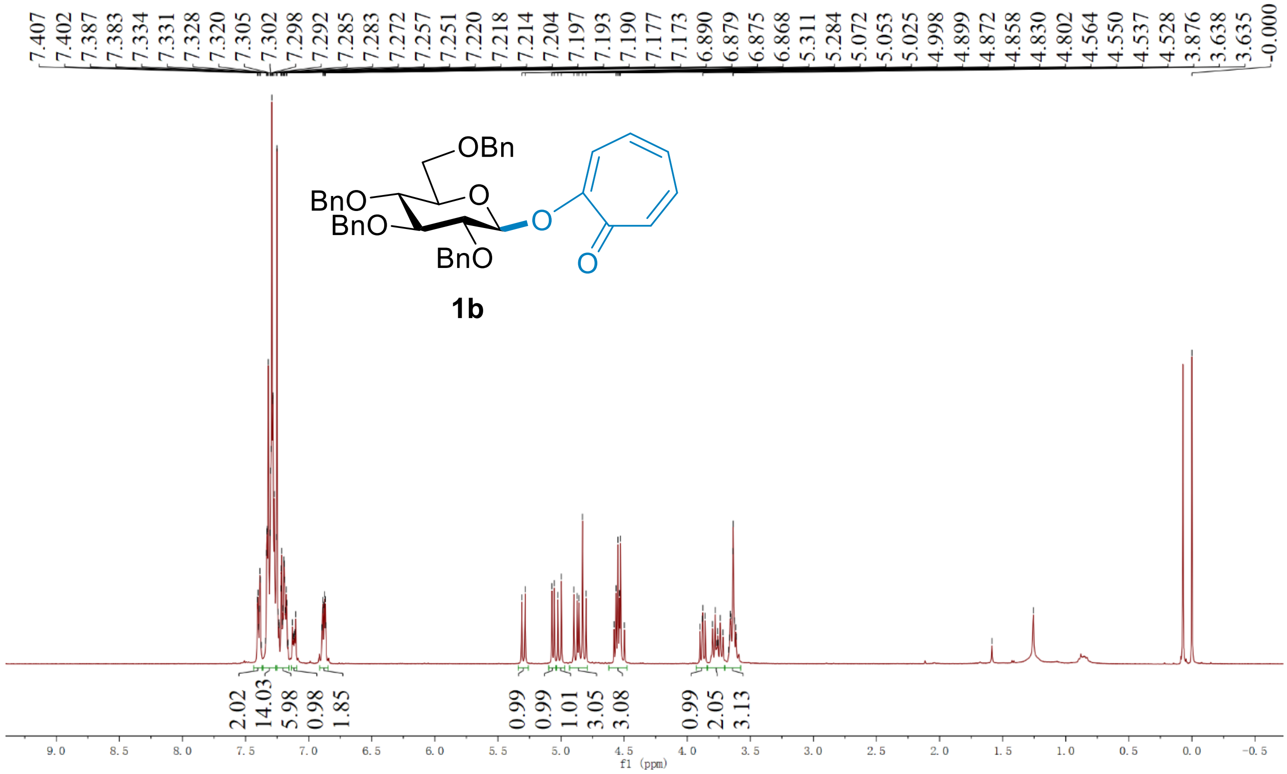

^{13}C NMR Spectrum of **1b** (101 MHz, $CDCl_3$)

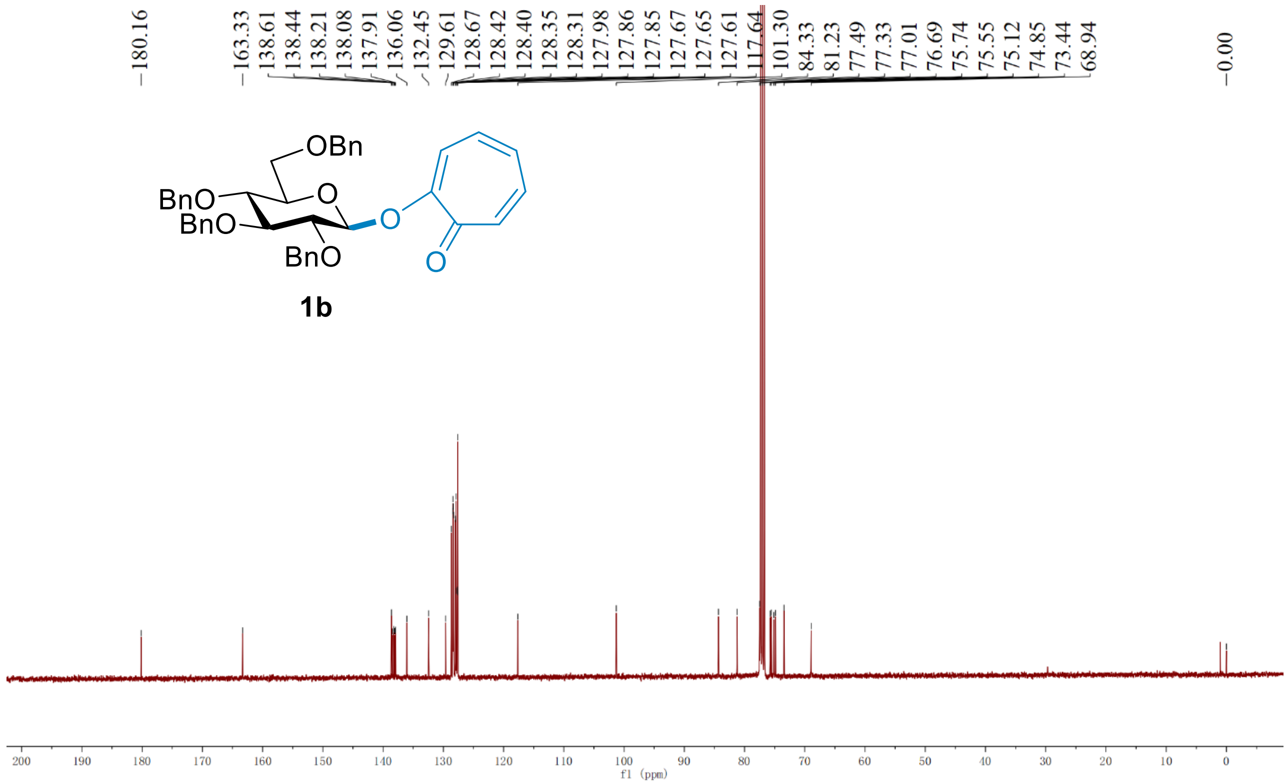

^{1}H NMR Spectrum of **1c** (400 MHz, $CDCl_3$)

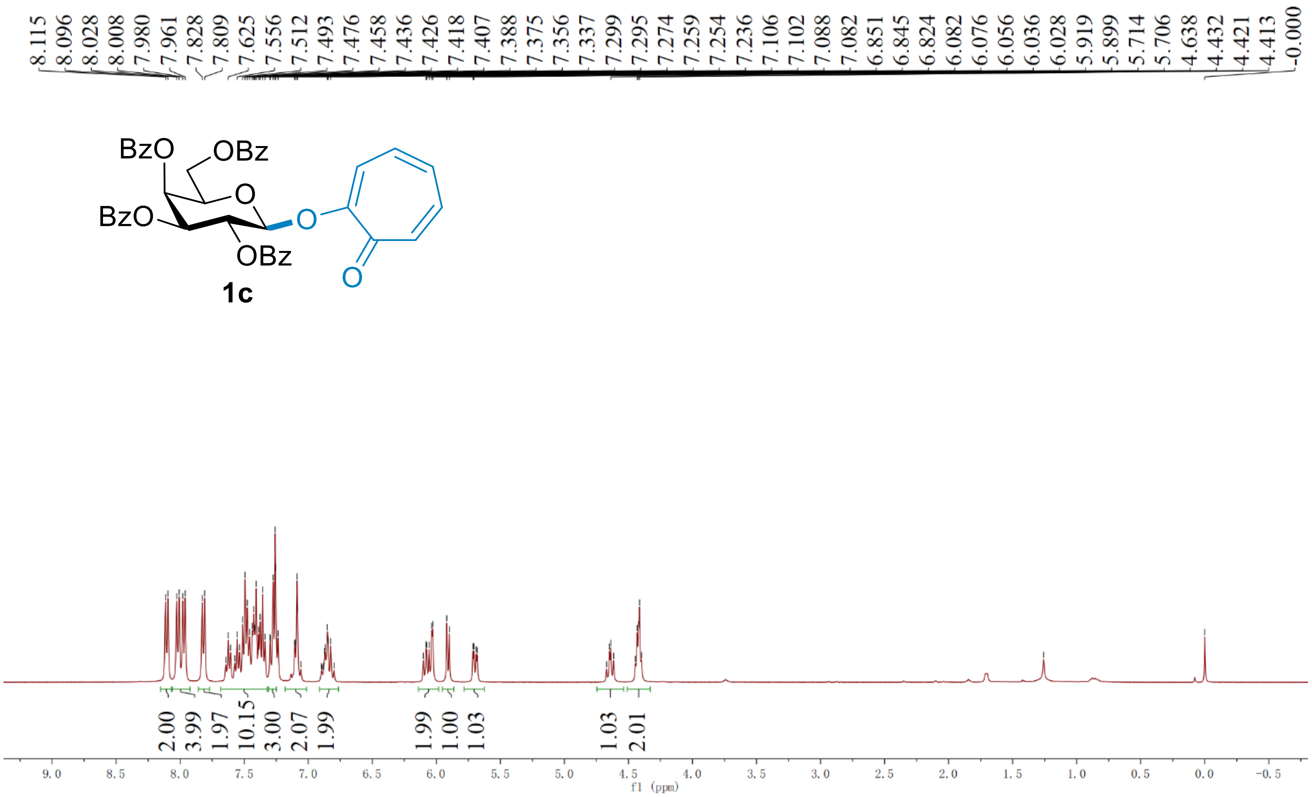

^{13}C NMR Spectrum of **1c** (101 MHz, $CDCl_3$)

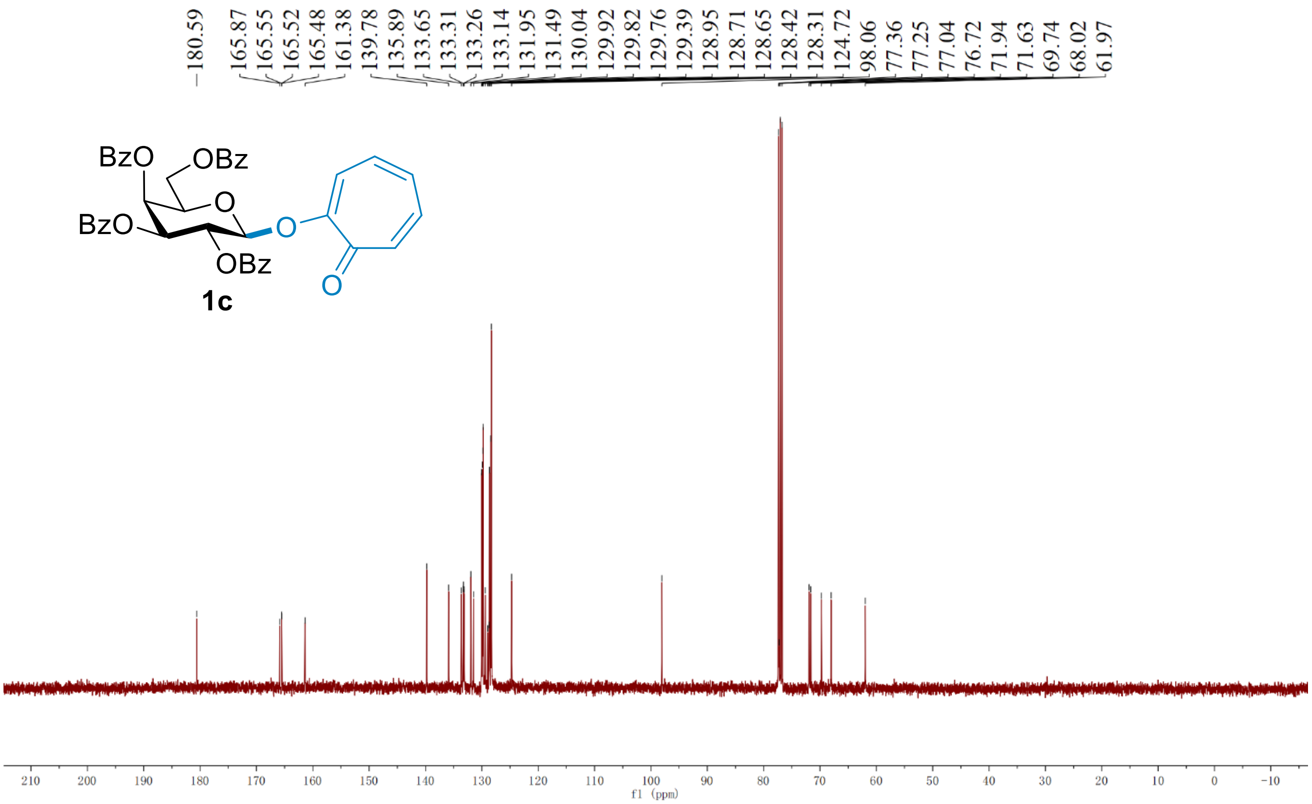

^{1}H NMR Spectrum of **1d** (400 MHz, $CDCl_3$)

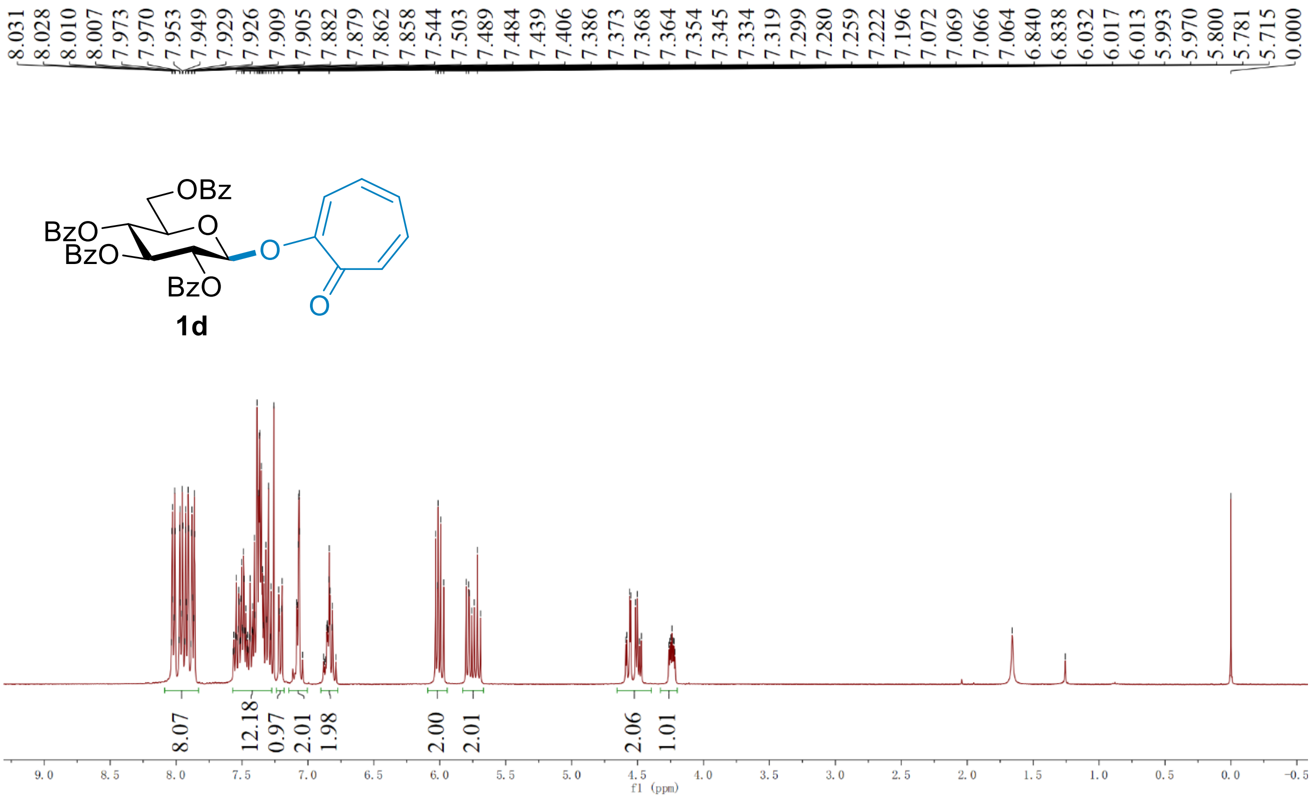

^{13}C NMR Spectrum of **1d** (101 MHz, $CDCl_3$)

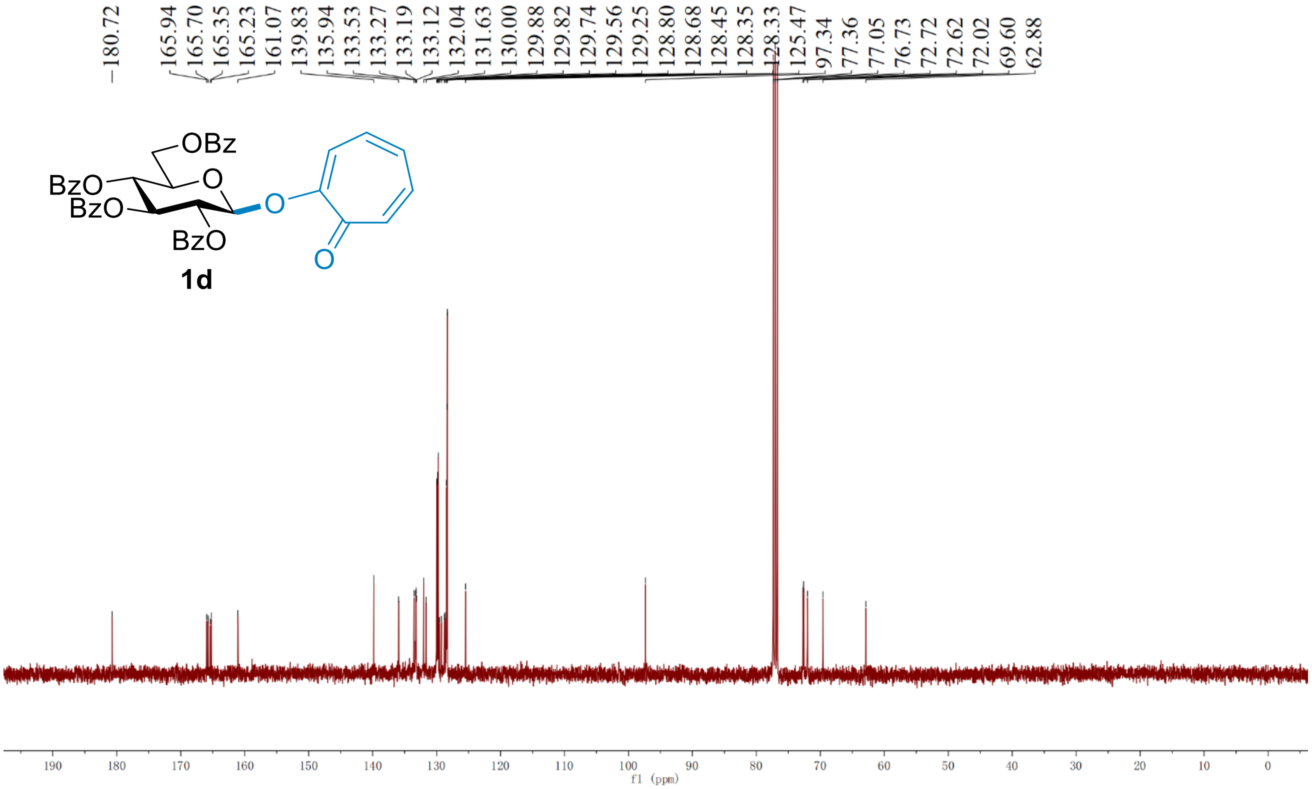

^{1}H NMR Spectrum of **1e** (400 MHz, $CDCl_3$)

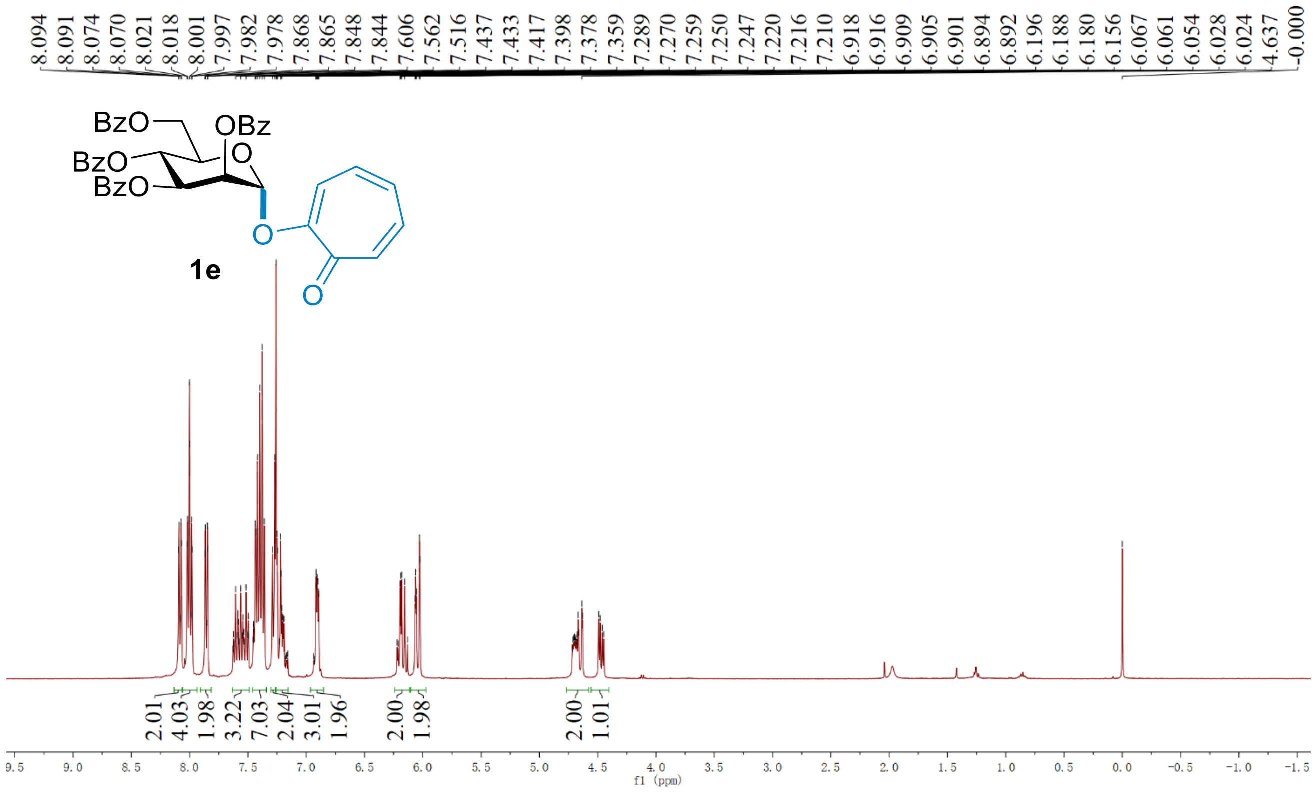

^{13}C NMR Spectrum of **1e** (101 MHz, $CDCl_3$)

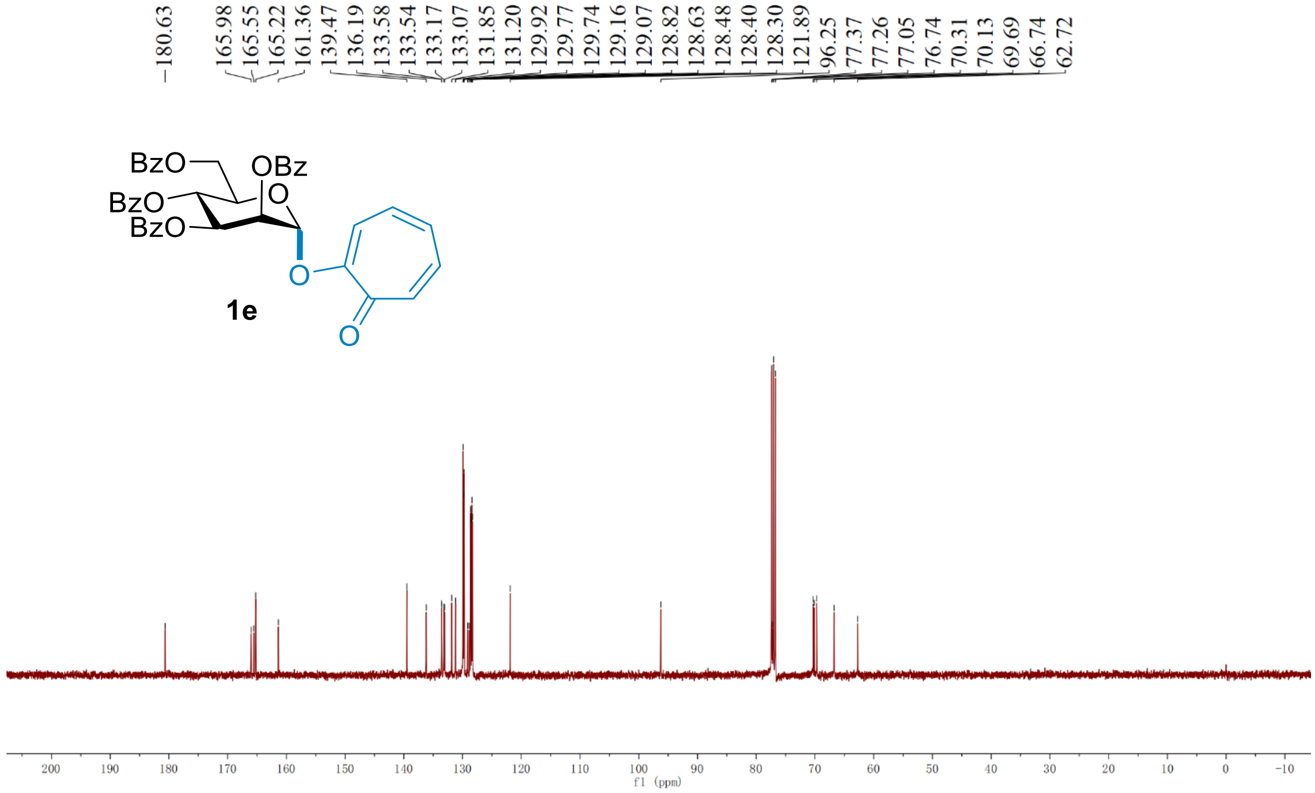

^{1}H NMR Spectrum of **1f** (400 MHz, $CDCl_3$)

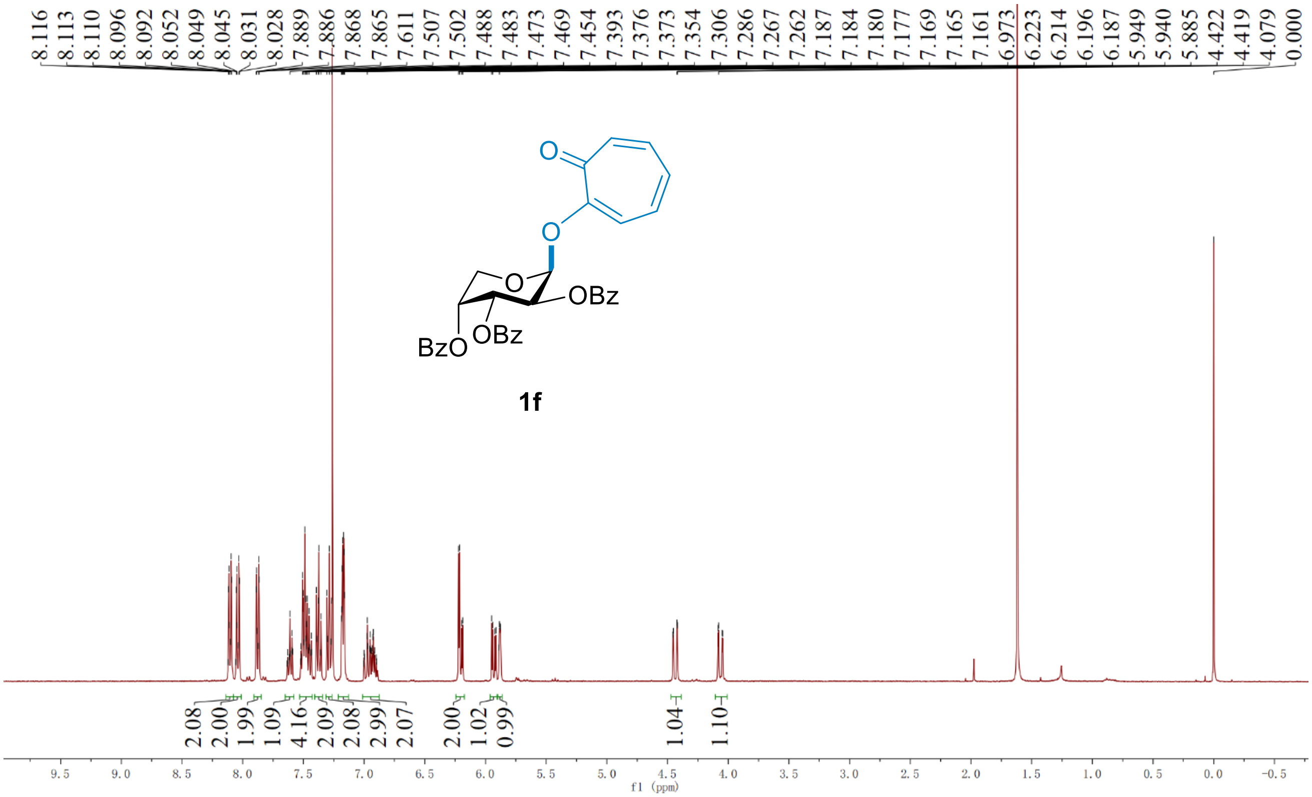

^{13}C NMR Spectrum of **1f** (101 MHz, $CDCl_3$)

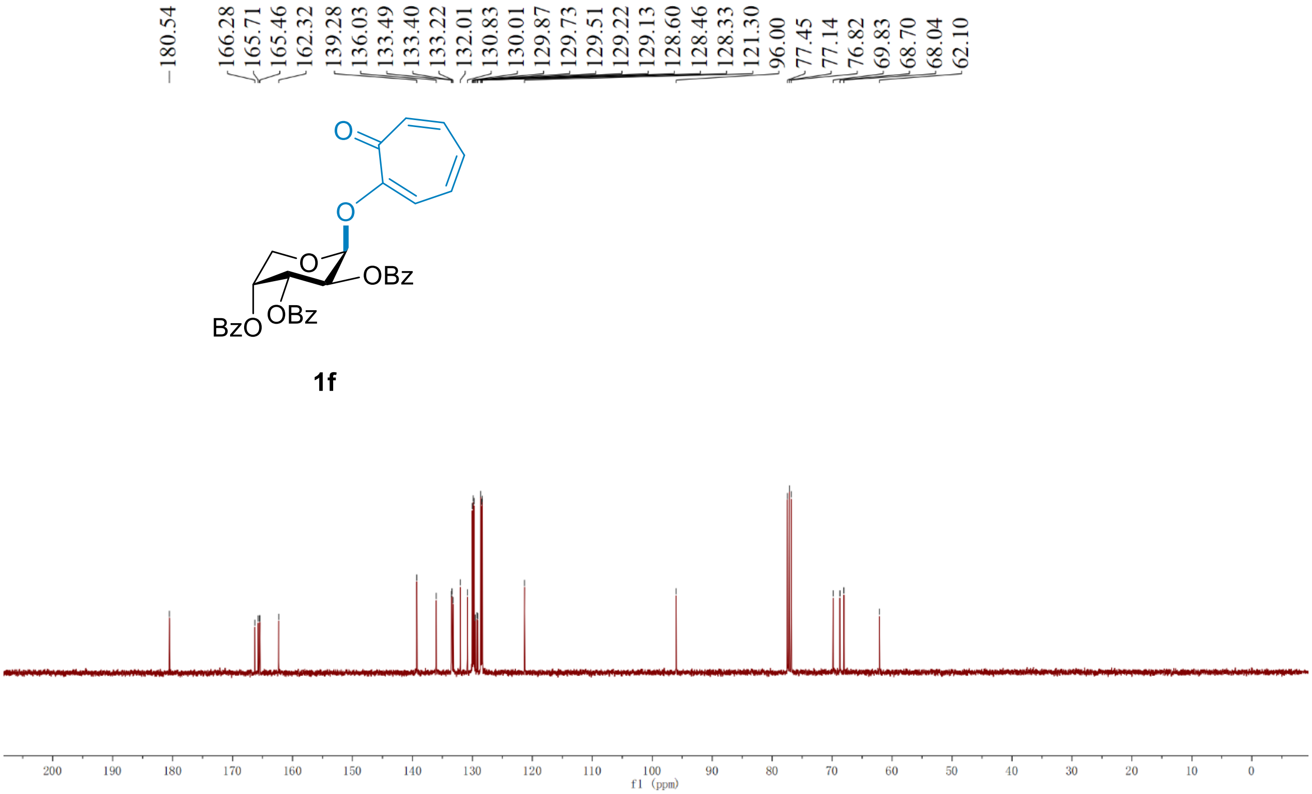

^{1}H NMR Spectrum of **1g** (400 MHz, $CDCl_3$)

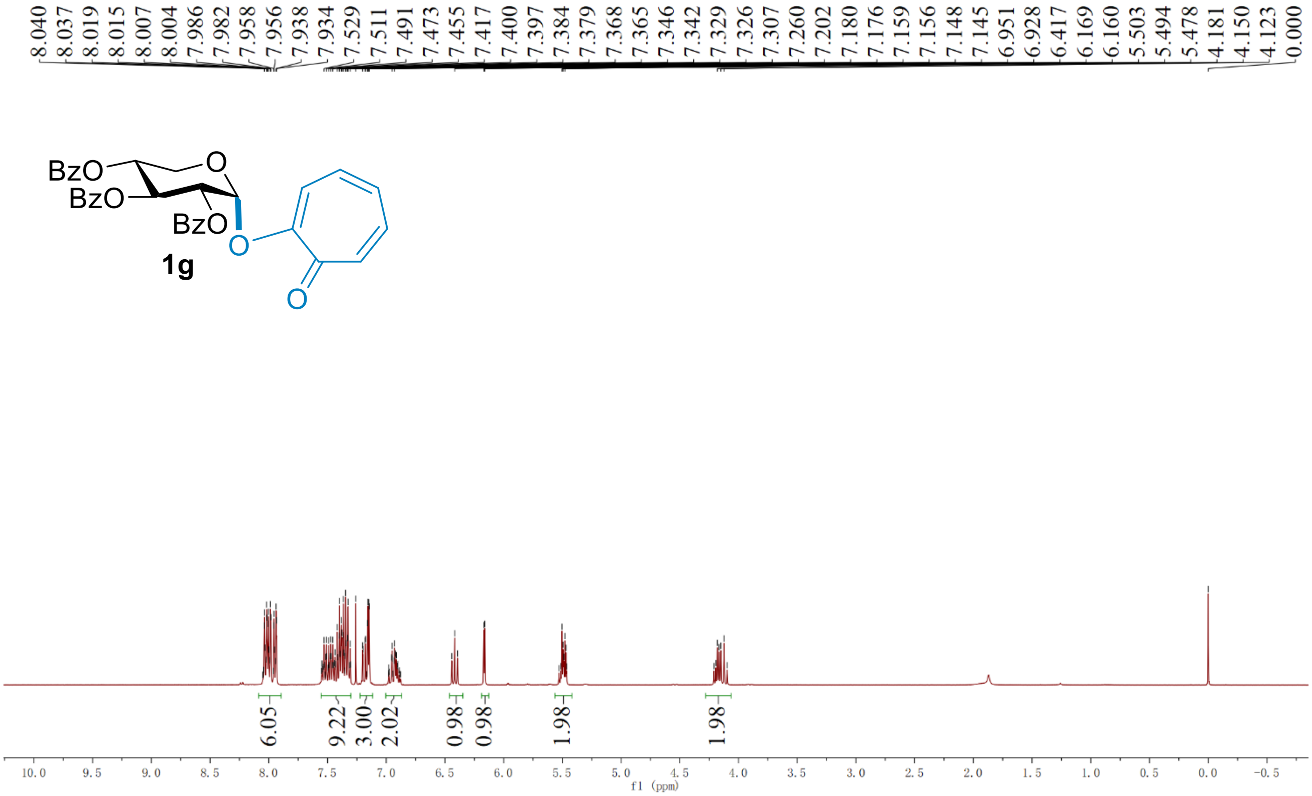

^{13}C NMR Spectrum of **1g** (101 MHz, $CDCl_3$)

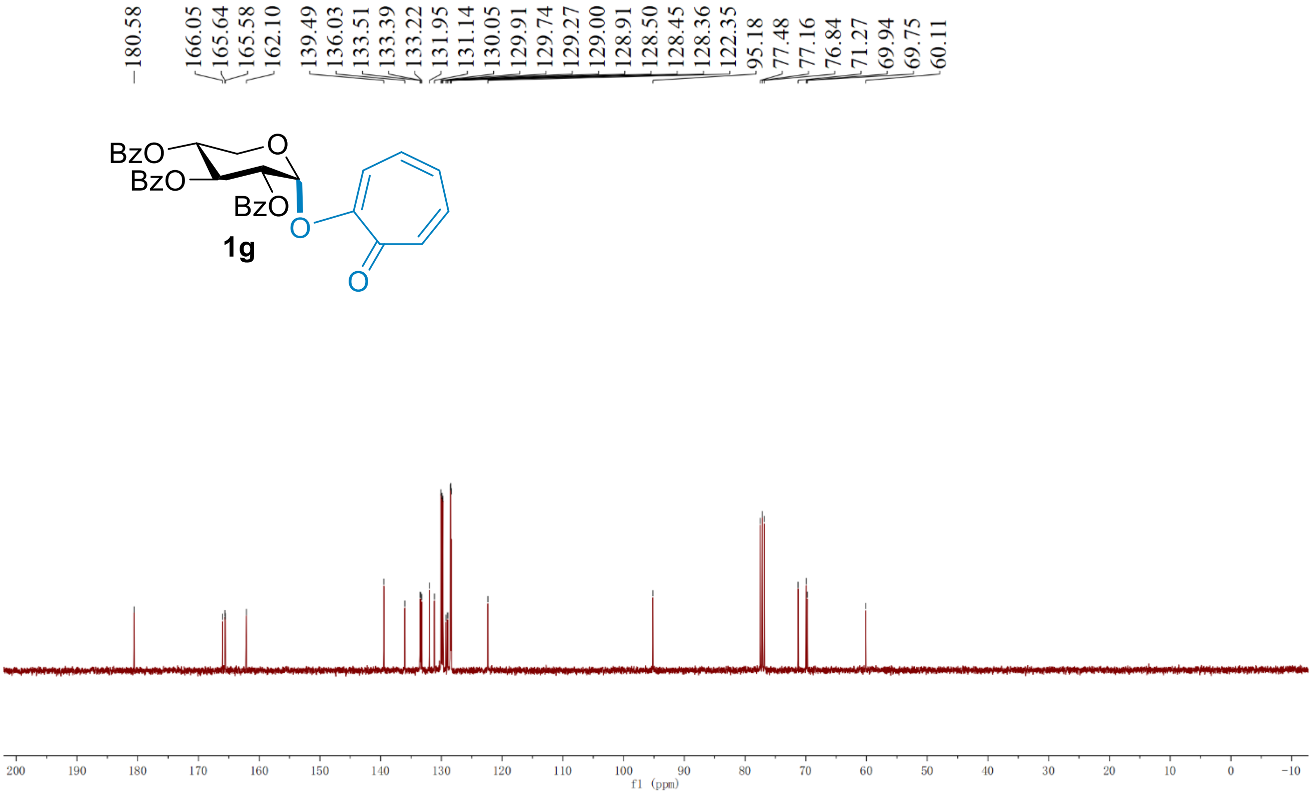

^{1}H NMR Spectrum of **1h** (400 MHz, $CDCl_3$)

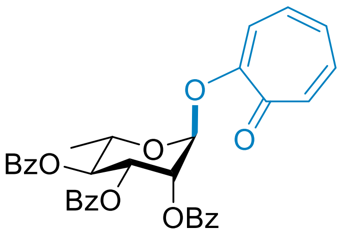

1h

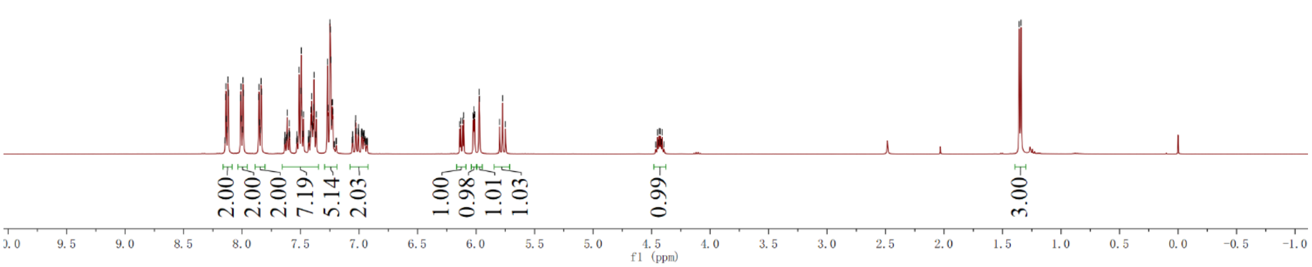

^{13}C NMR Spectrum of **1h** (101 MHz, $CDCl_3$)

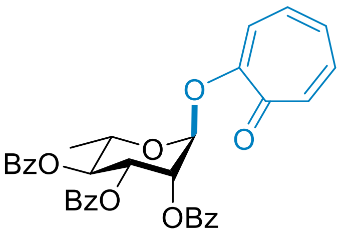

1h

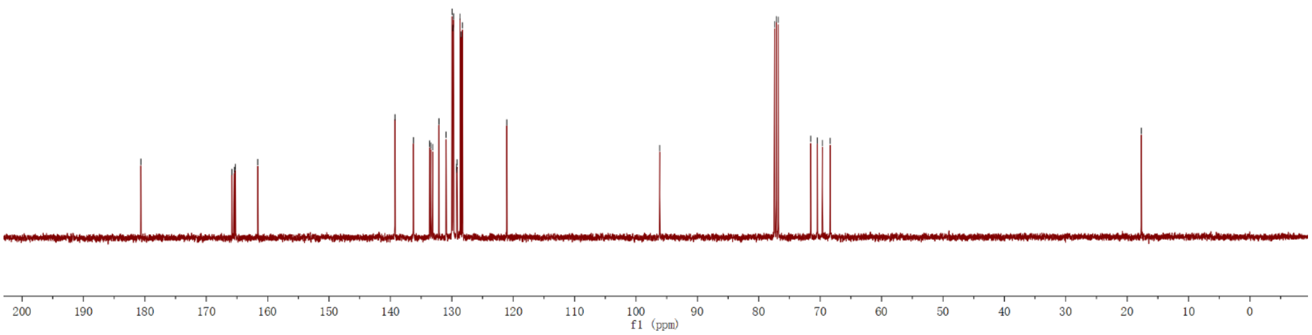

^{1}H NMR Spectrum of **1i** (400 MHz, $CDCl_3$)

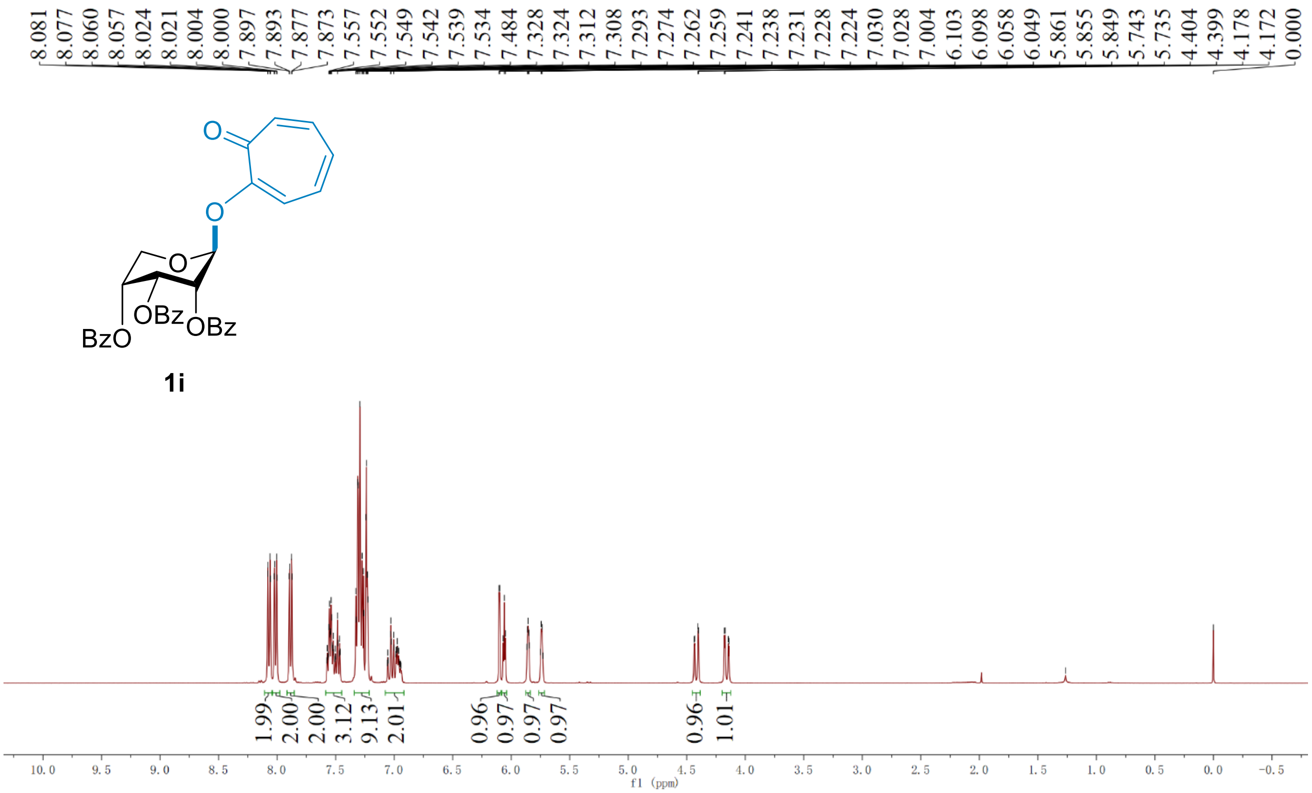

^{13}C NMR Spectrum of **1i** (101 MHz, $CDCl_3$)

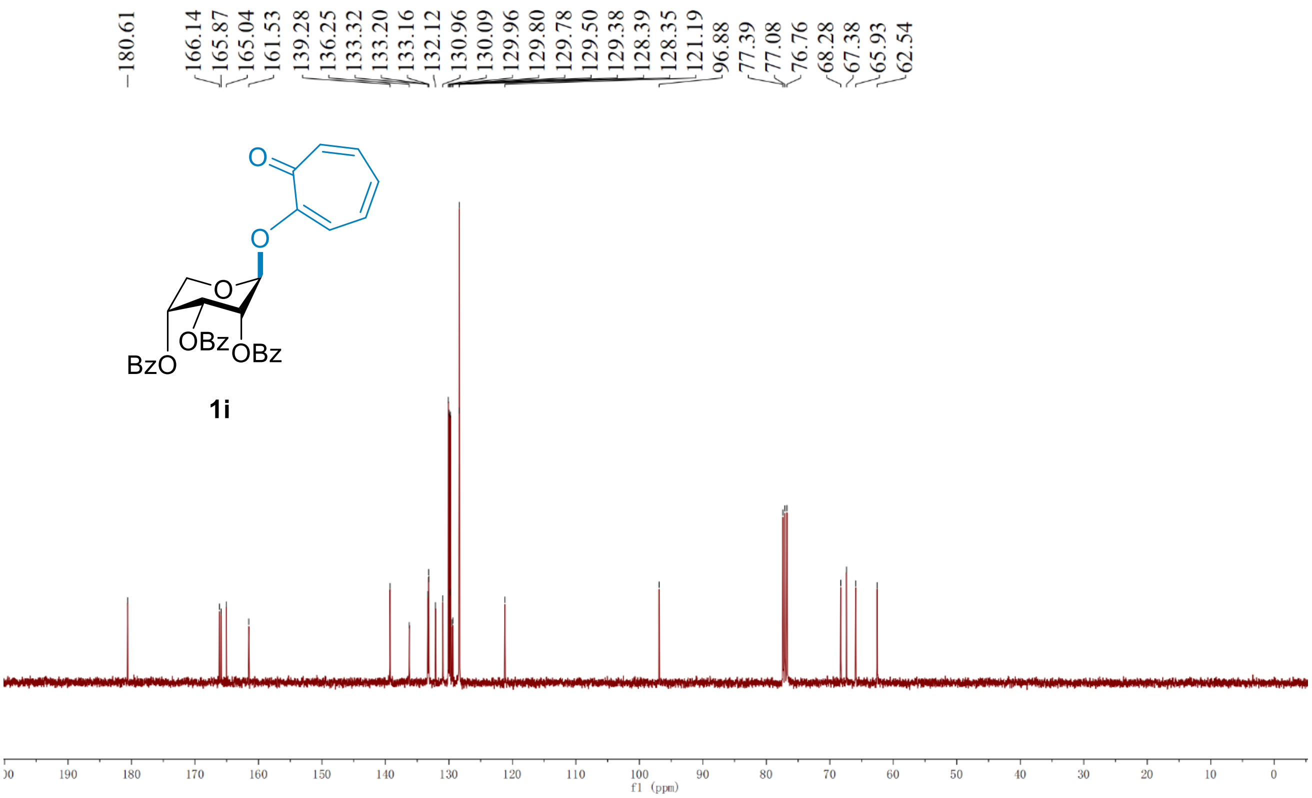

^{1}H NMR Spectrum of **1j** (400 MHz, $CDCl_3$)

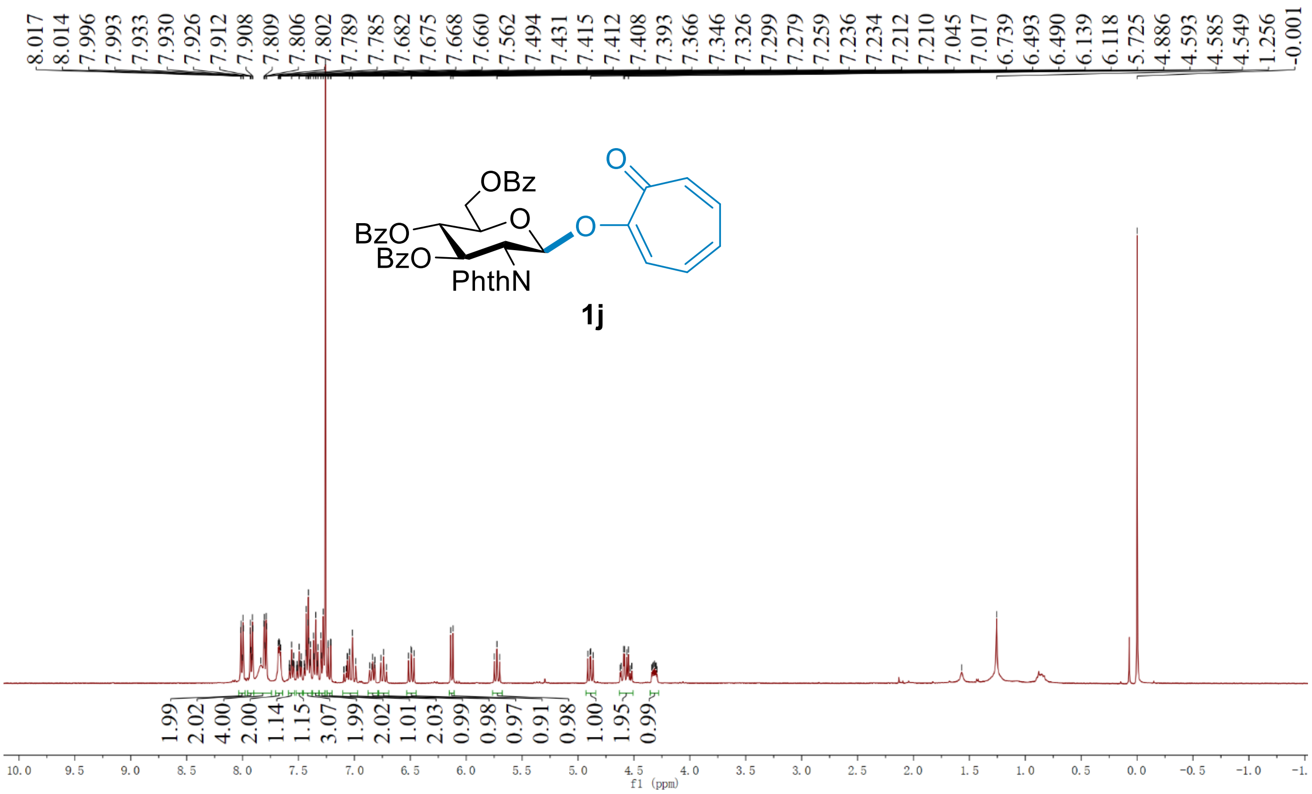

^{13}C NMR Spectrum of **1j** (101 MHz, $CDCl_3$)

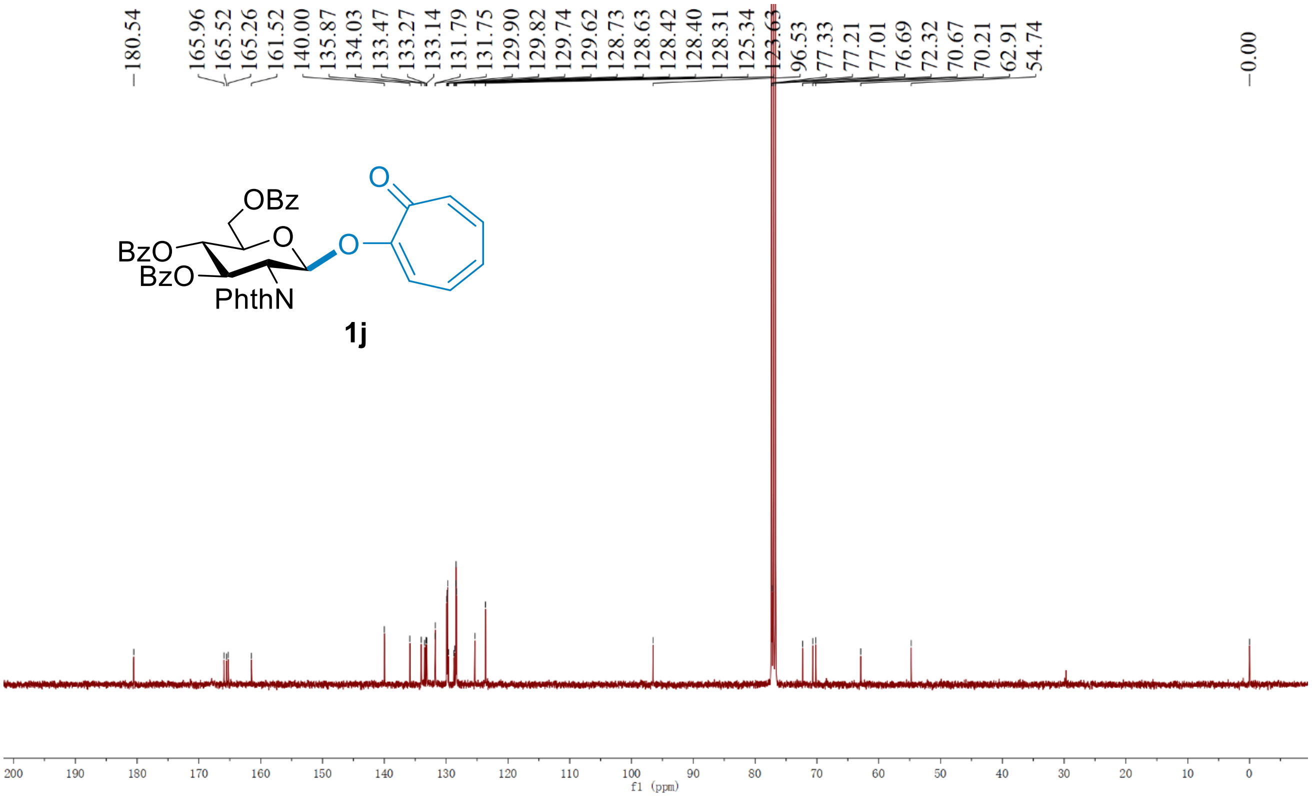

^{1}H NMR Spectrum of **1k** (400 MHz, $CDCl_3$)

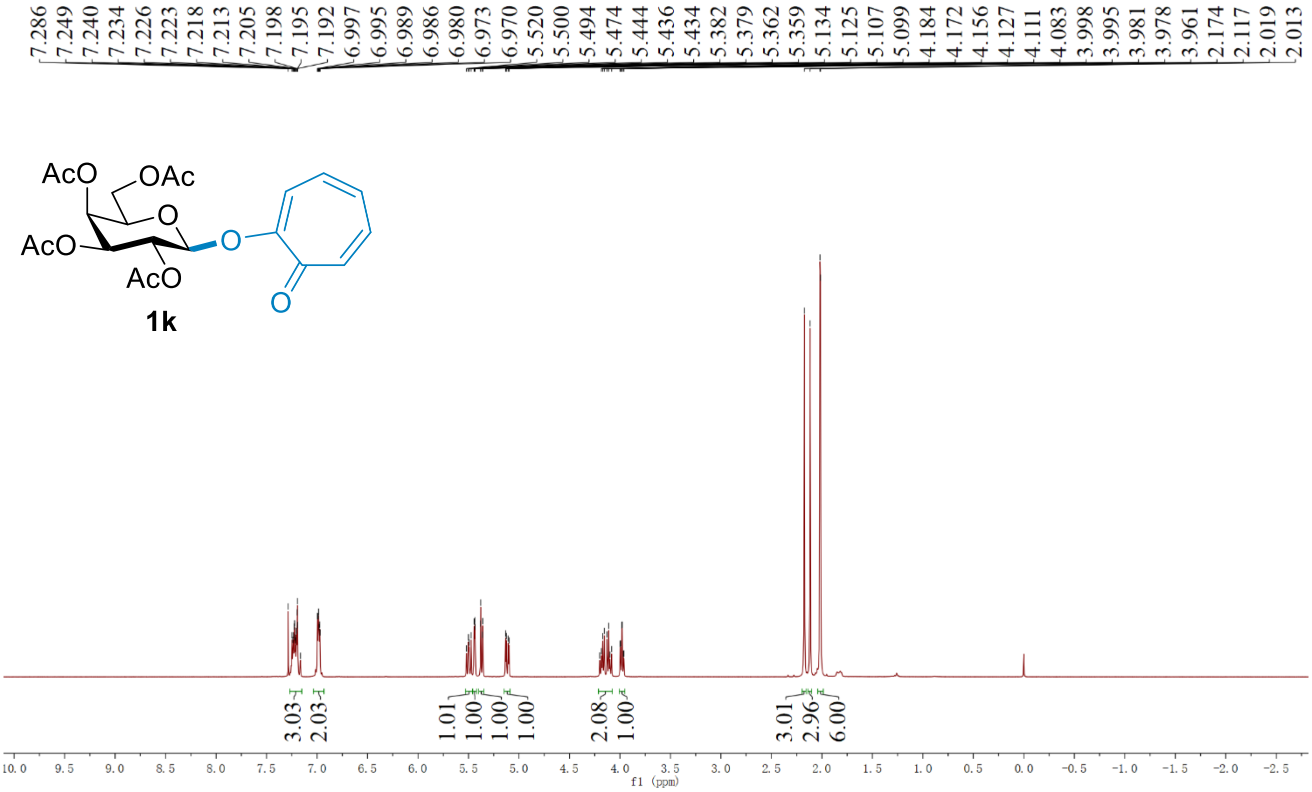

^{13}C NMR Spectrum of **1k** (101 MHz, $CDCl_3$)

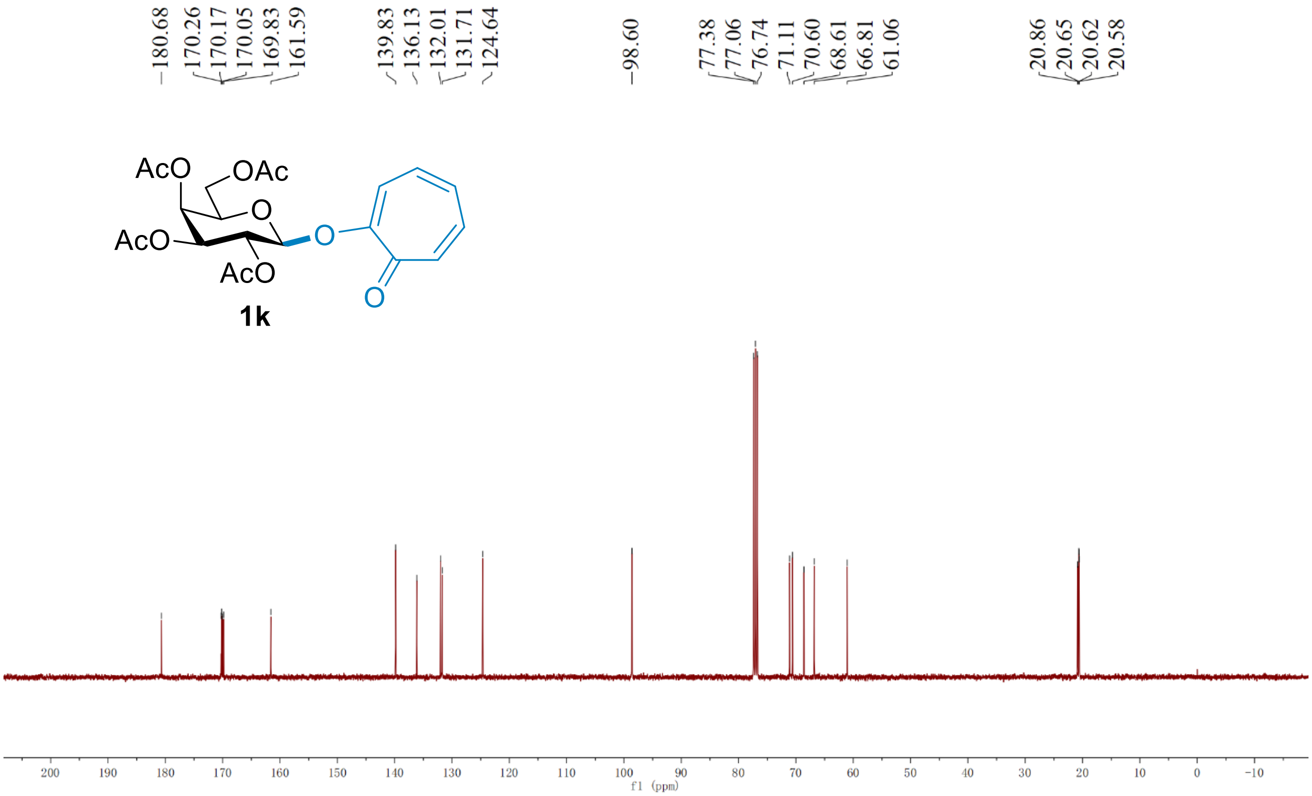

^{1}H NMR Spectrum of **1l** (400 MHz, $CDCl_3$)

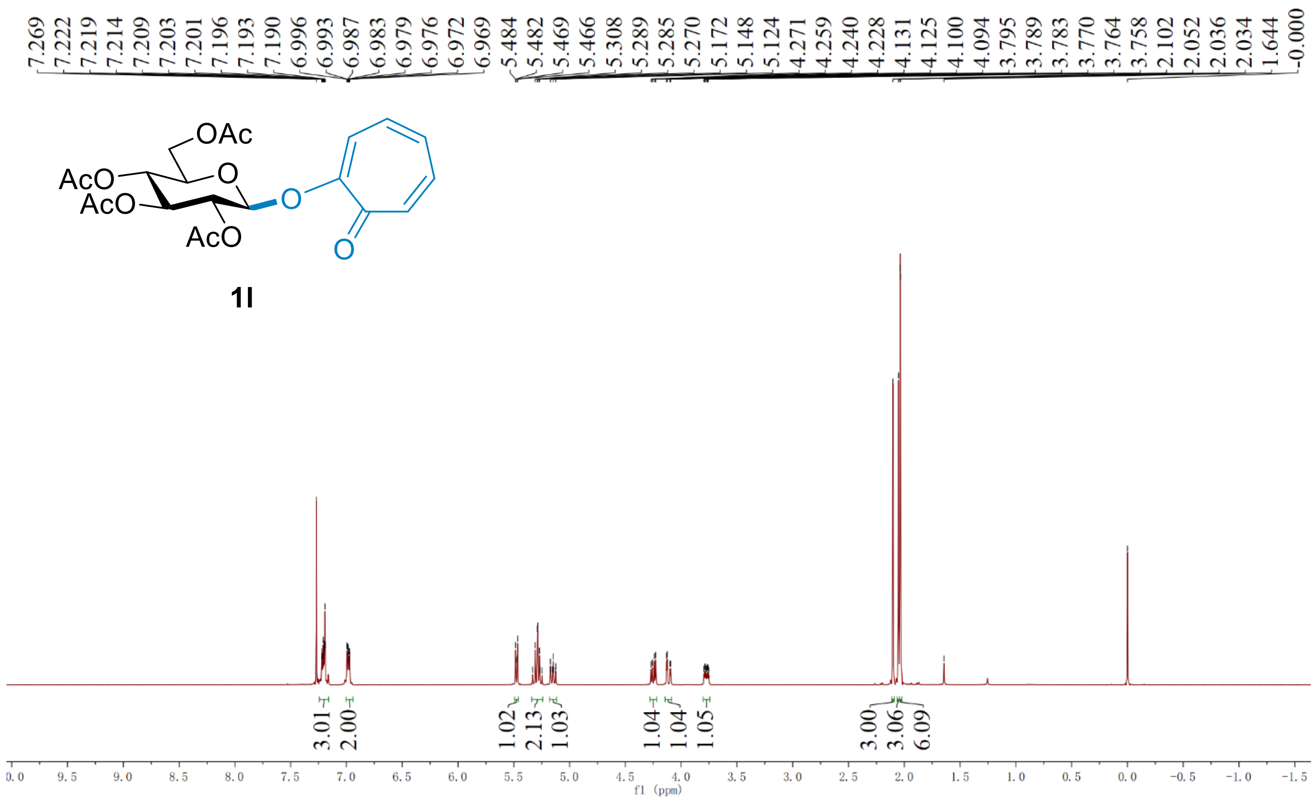

^{13}C NMR Spectrum of **1l** (101 MHz, $CDCl_3$)

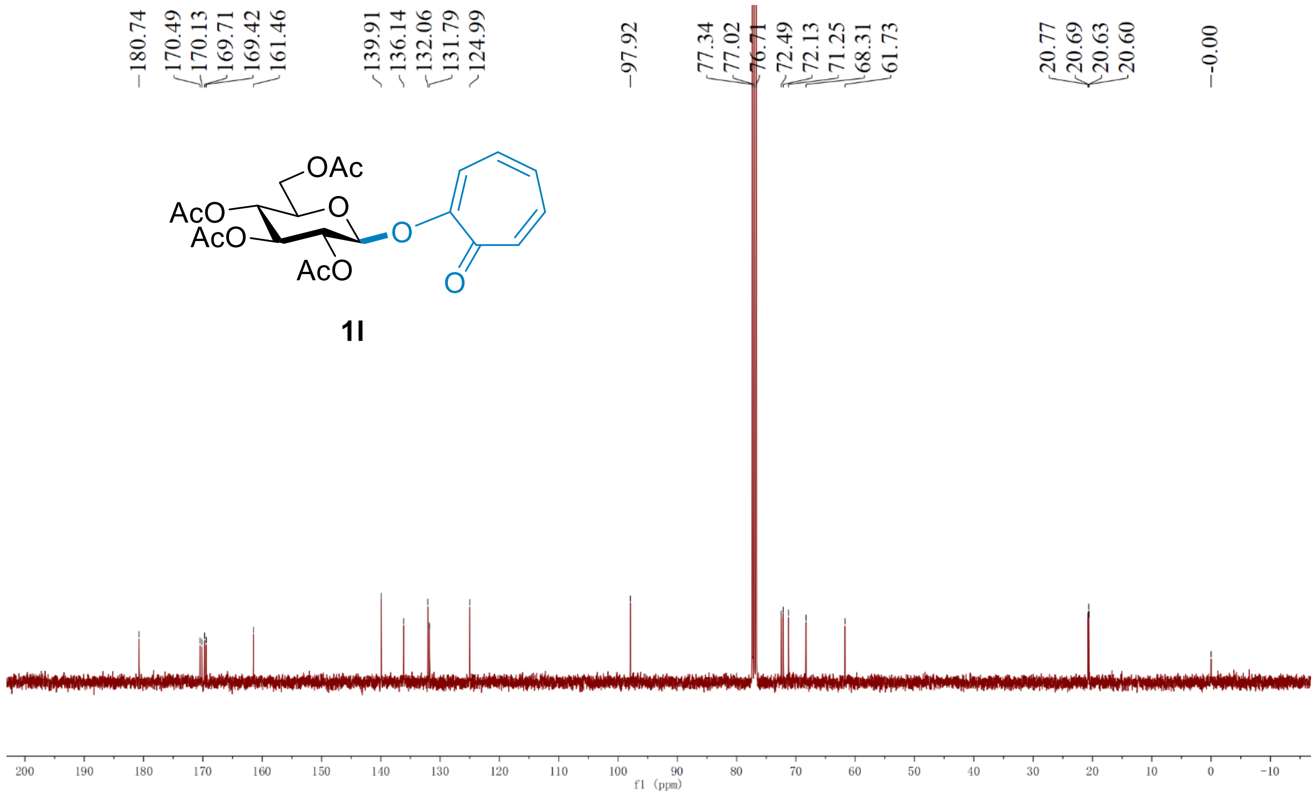

^{1}H NMR Spectrum of **1m** (400 MHz, $CDCl_3$)

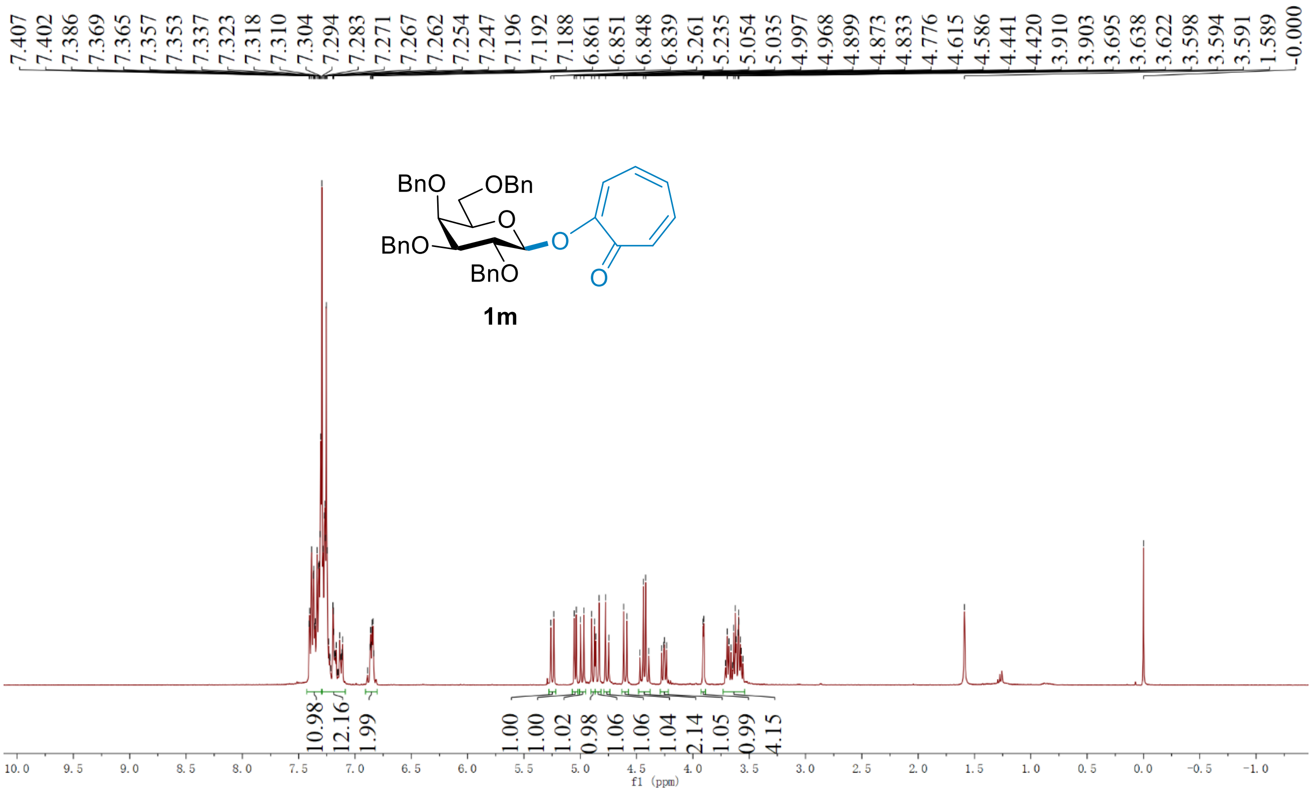

^{13}C NMR Spectrum of **1m** (101 MHz, $CDCl_3$)

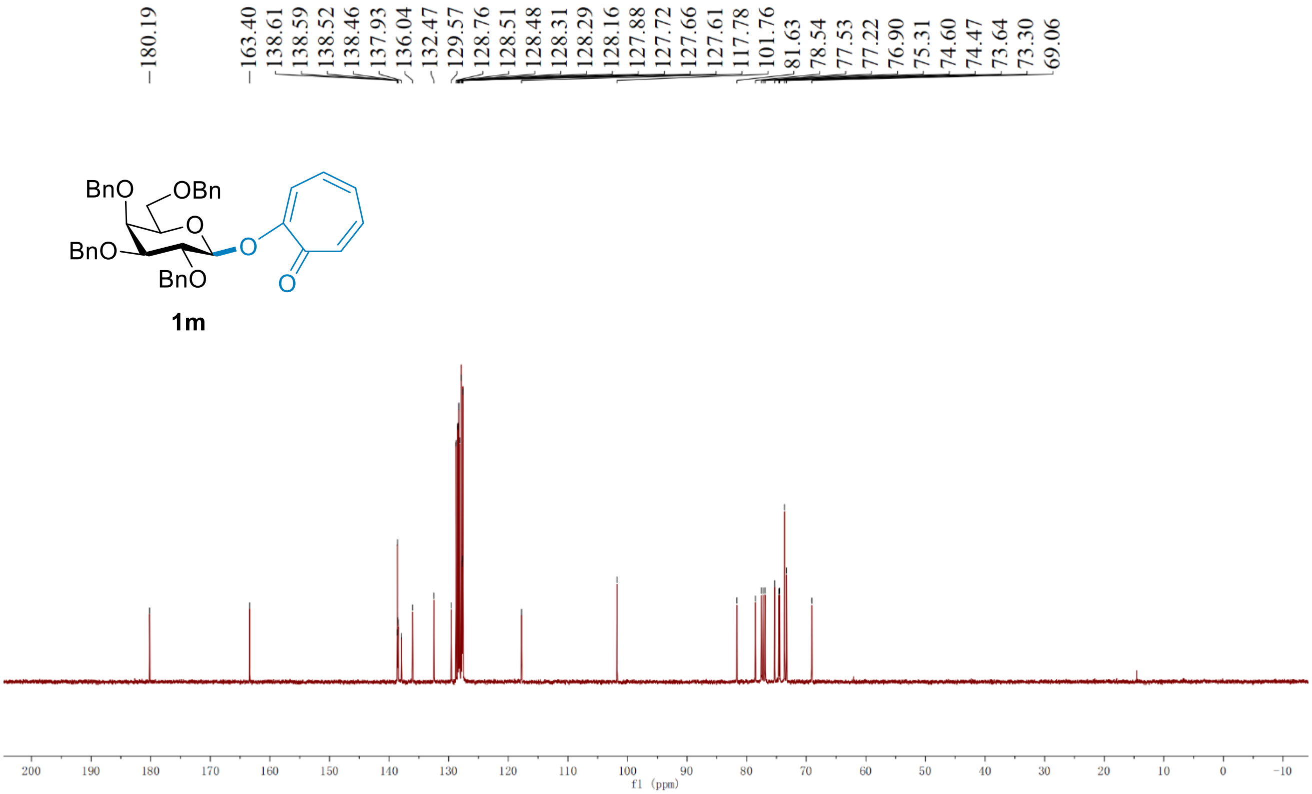

^{1}H NMR Spectrum of **1n** (400 MHz, $CDCl_3$)

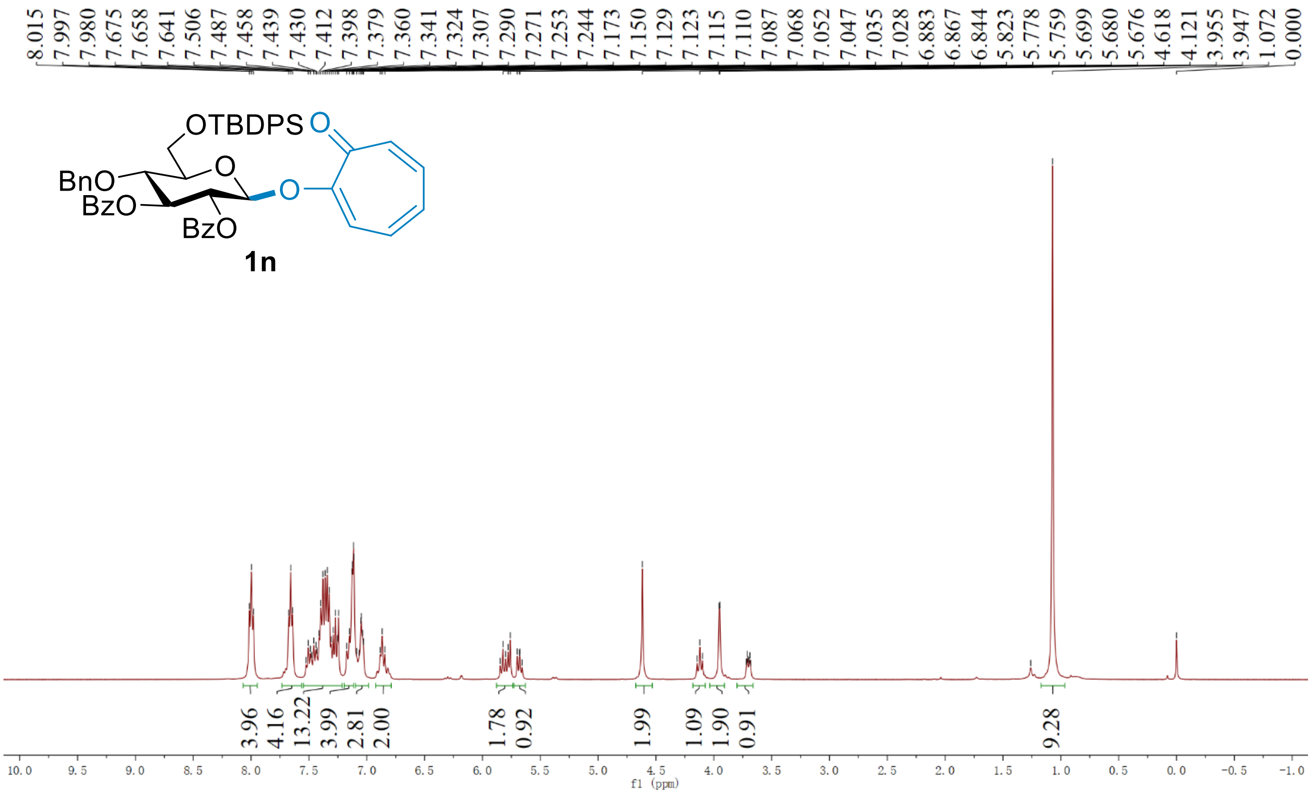

^{13}C NMR Spectrum of **1n** (101 MHz, $CDCl_3$)

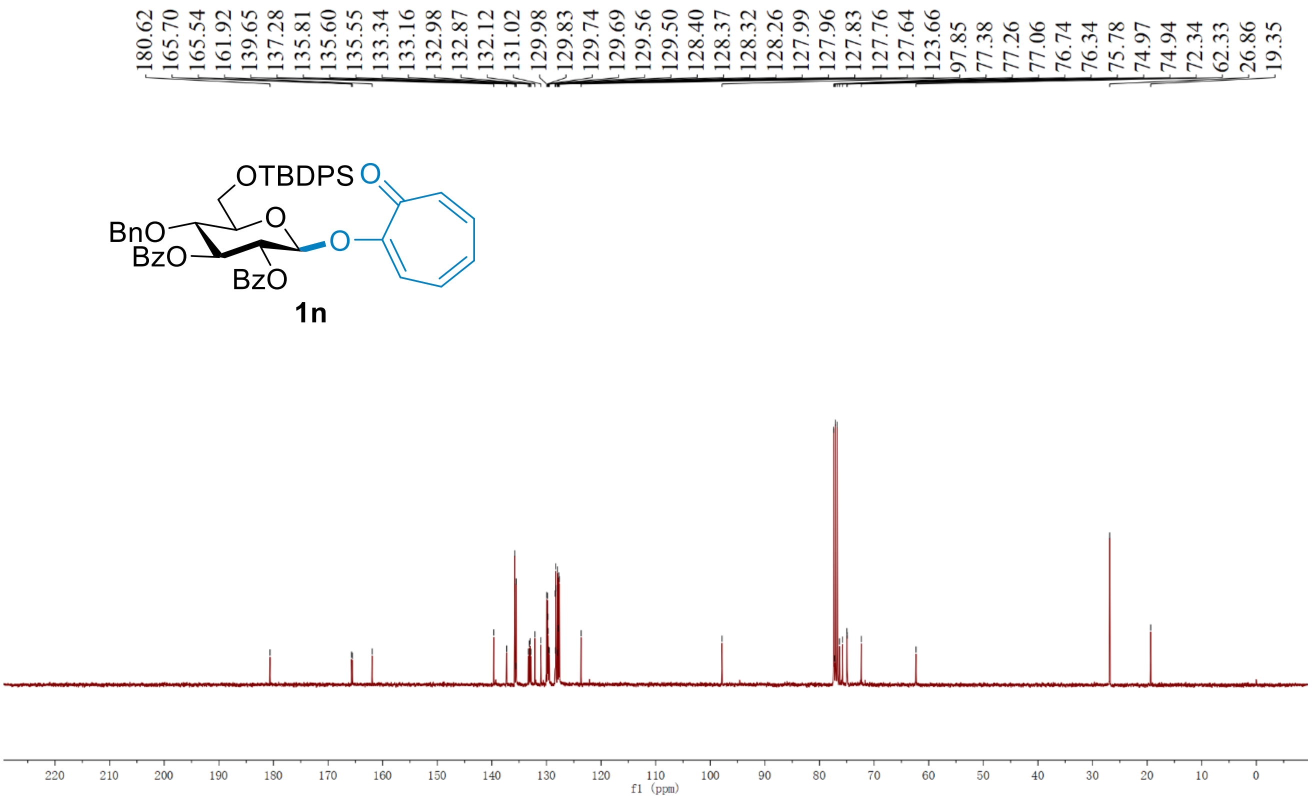

^{1}H NMR Spectrum of **1o** (400 MHz, $CDCl_3$)

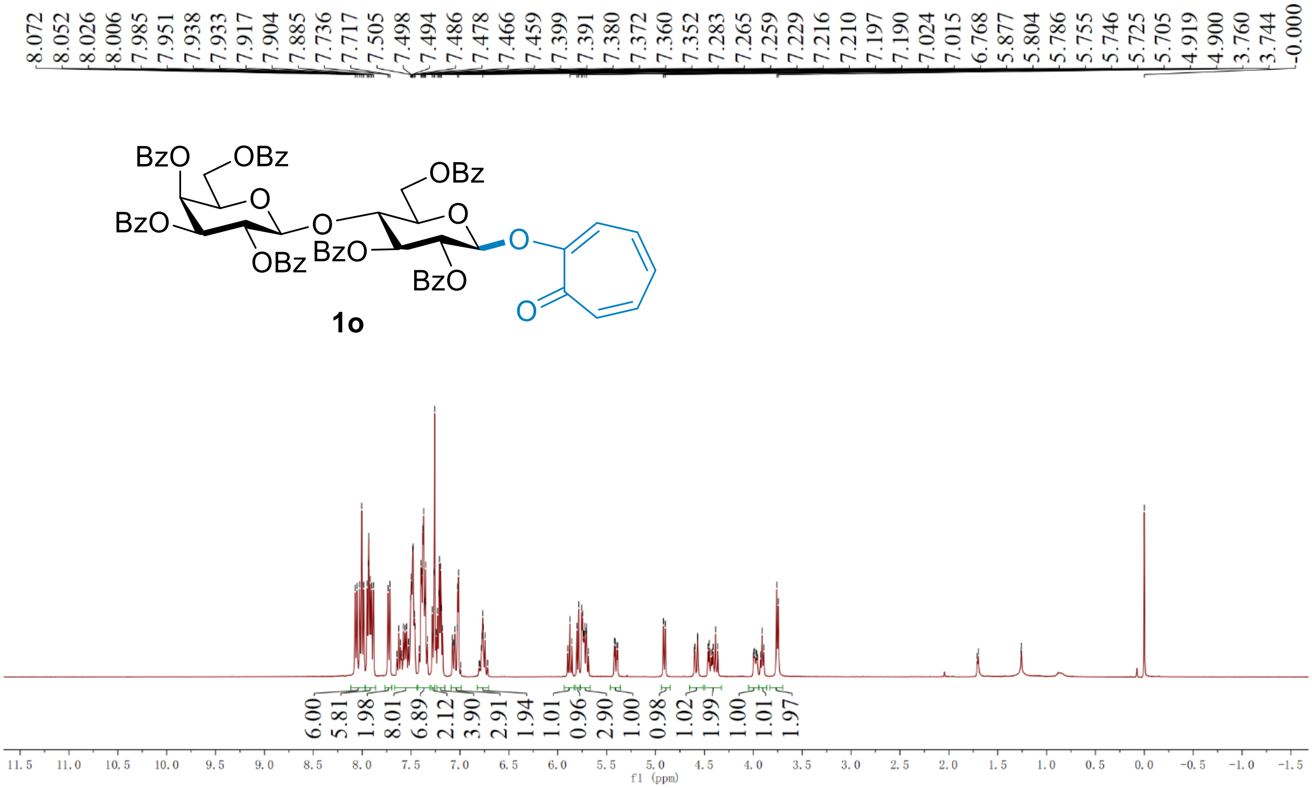

^{13}C NMR Spectrum of **1o** (101 MHz, $CDCl_3$)

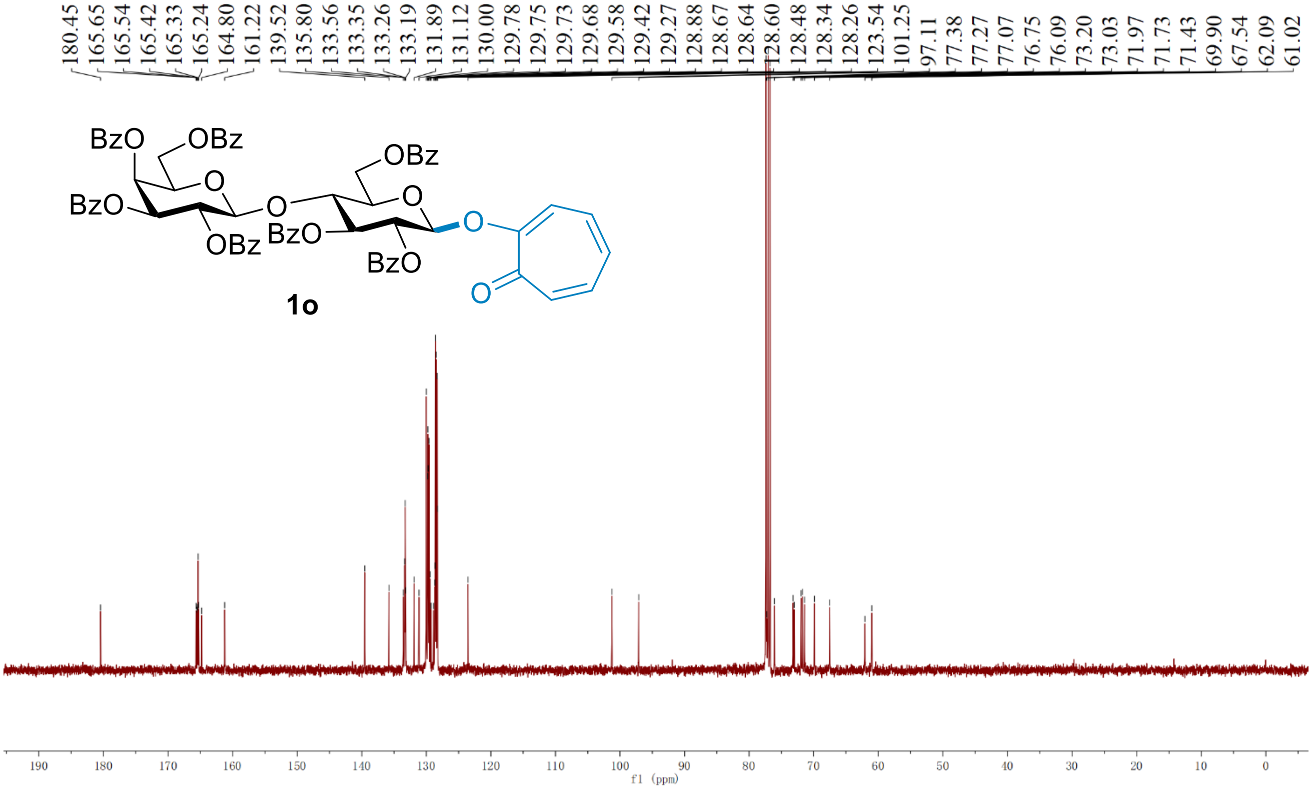

^{1}H NMR Spectrum of **1p** (400 MHz, $CDCl_3$)

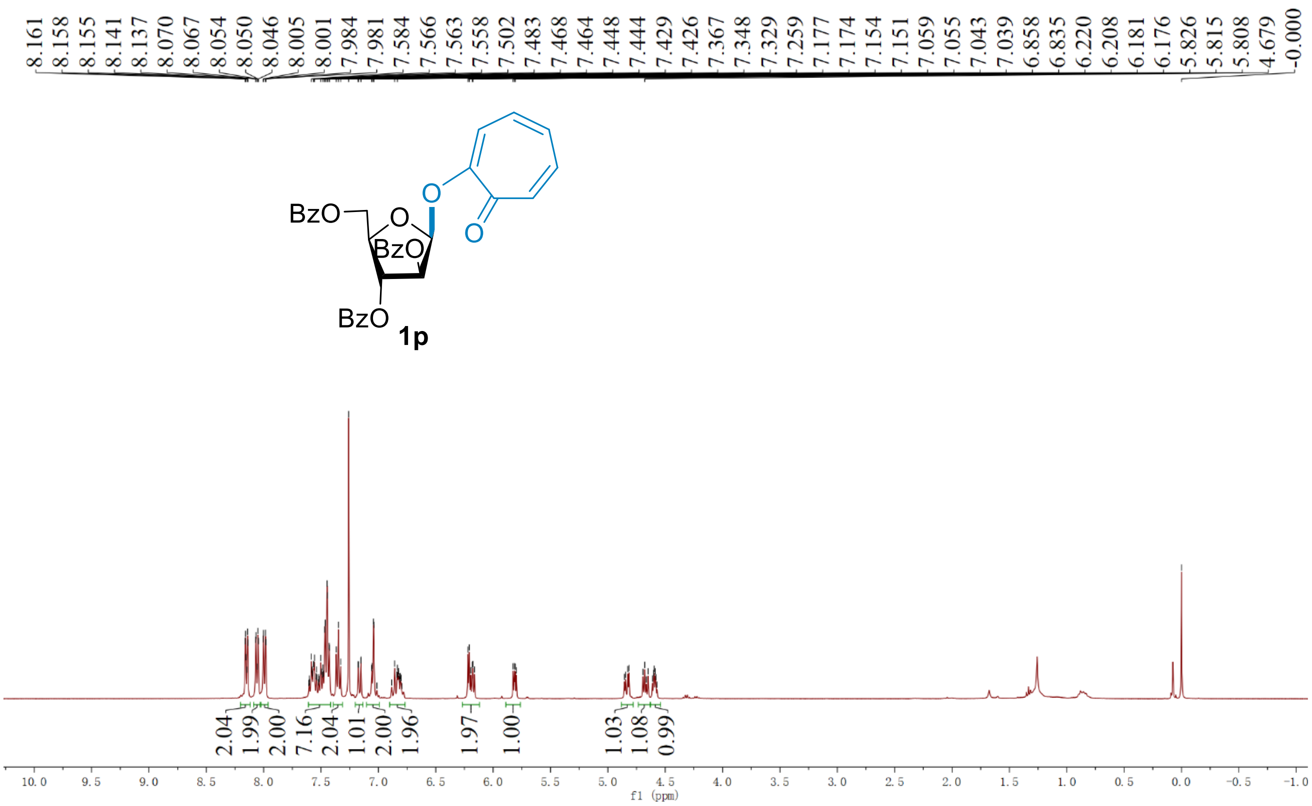

^{13}C NMR Spectrum of **1p** (101 MHz, $CDCl_3$)

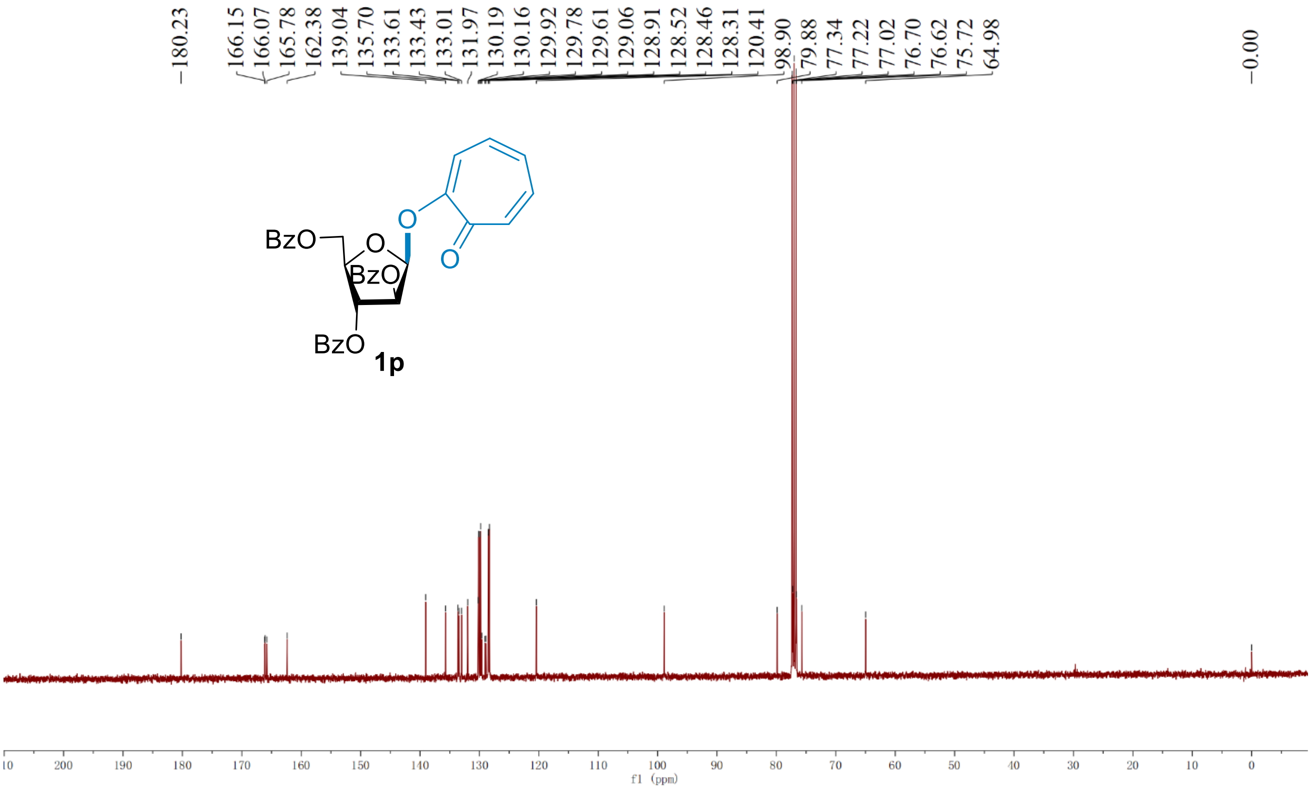

^{1}H NMR Spectrum of **1q** (400 MHz, $CDCl_3$)

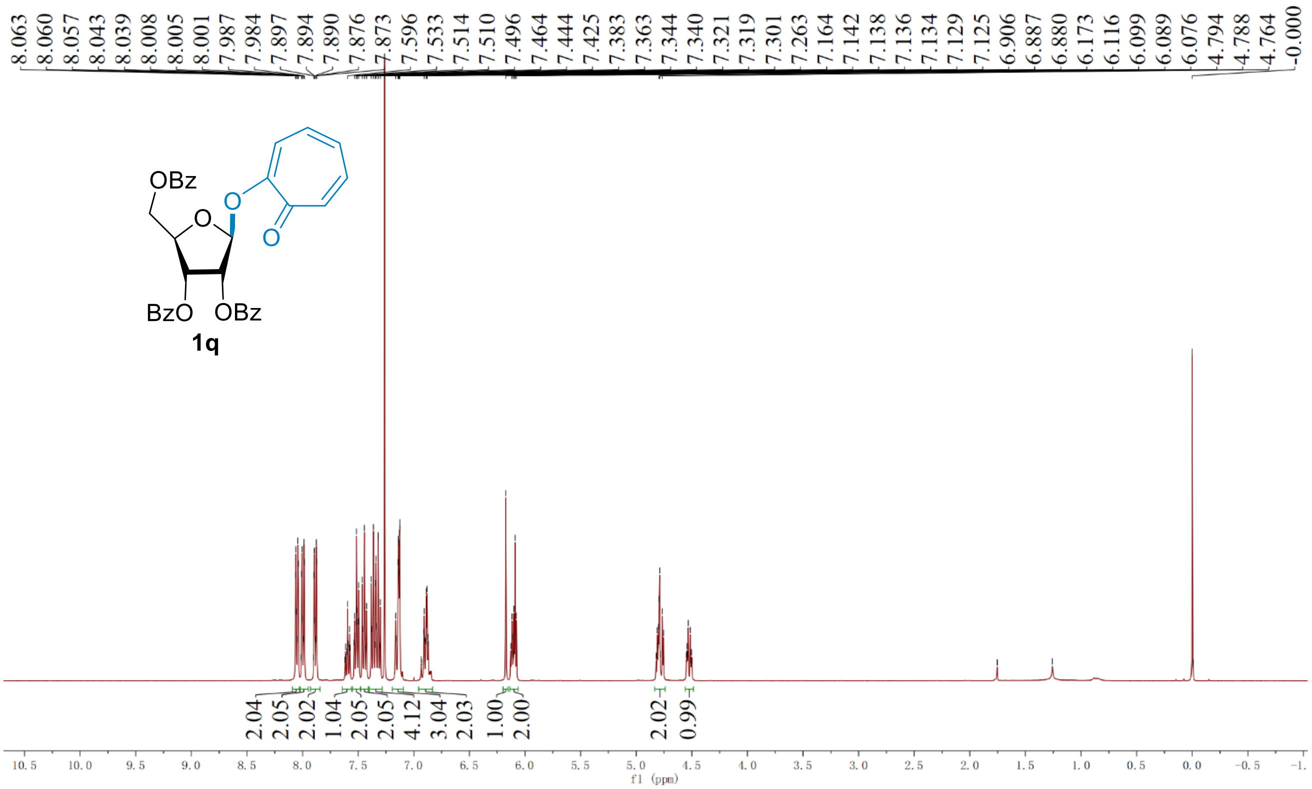

^{13}C NMR Spectrum of **1q** (101 MHz, $CDCl_3$)

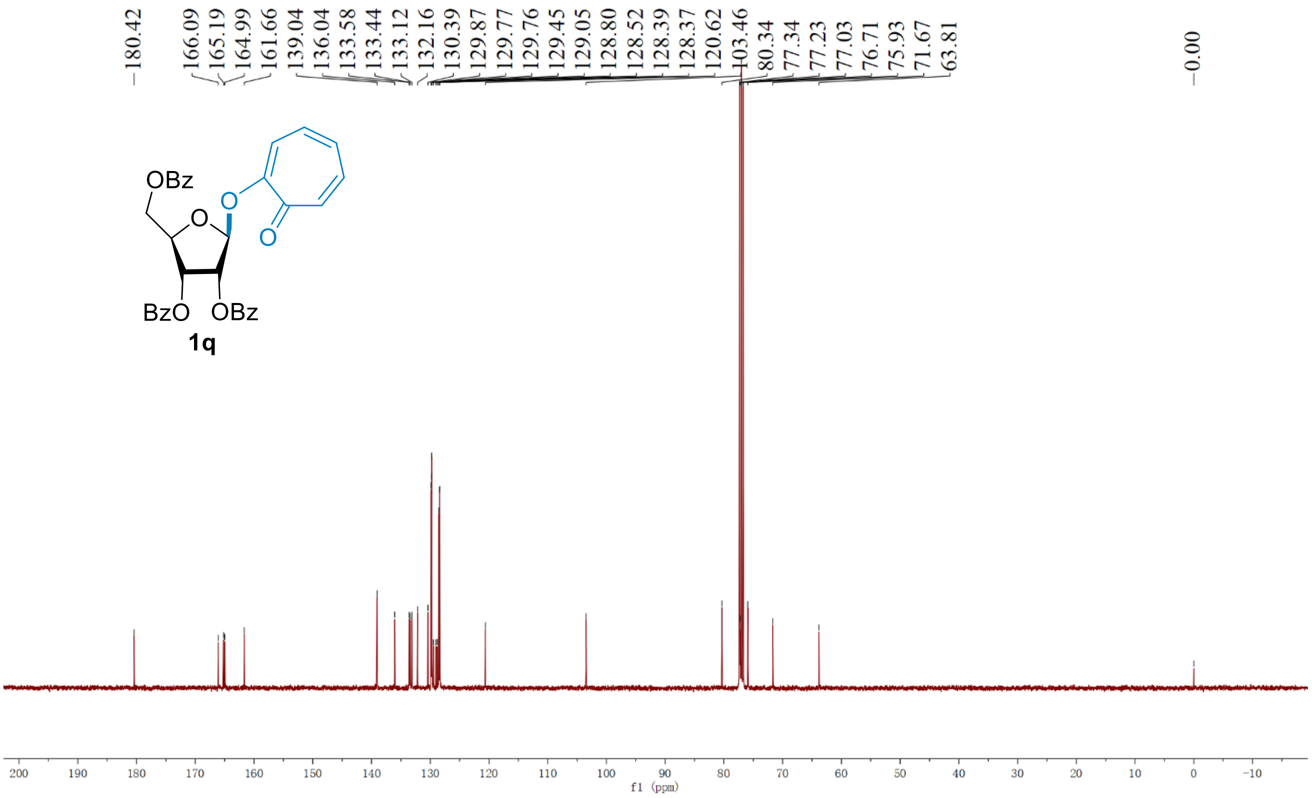

^{1}H NMR Spectrum of **1r** (400 MHz, $CDCl_3$)

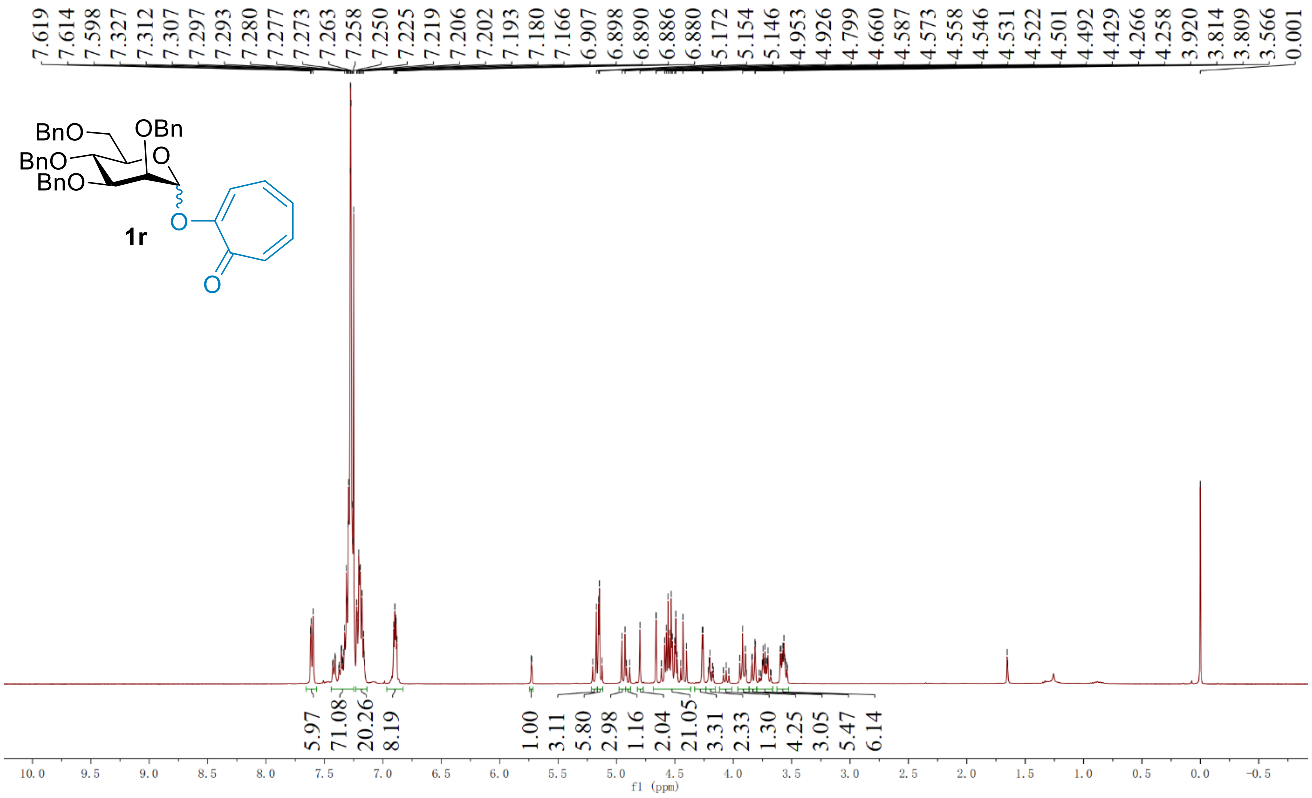

^{13}C NMR Spectrum of **1r** (101 MHz, $CDCl_3$)

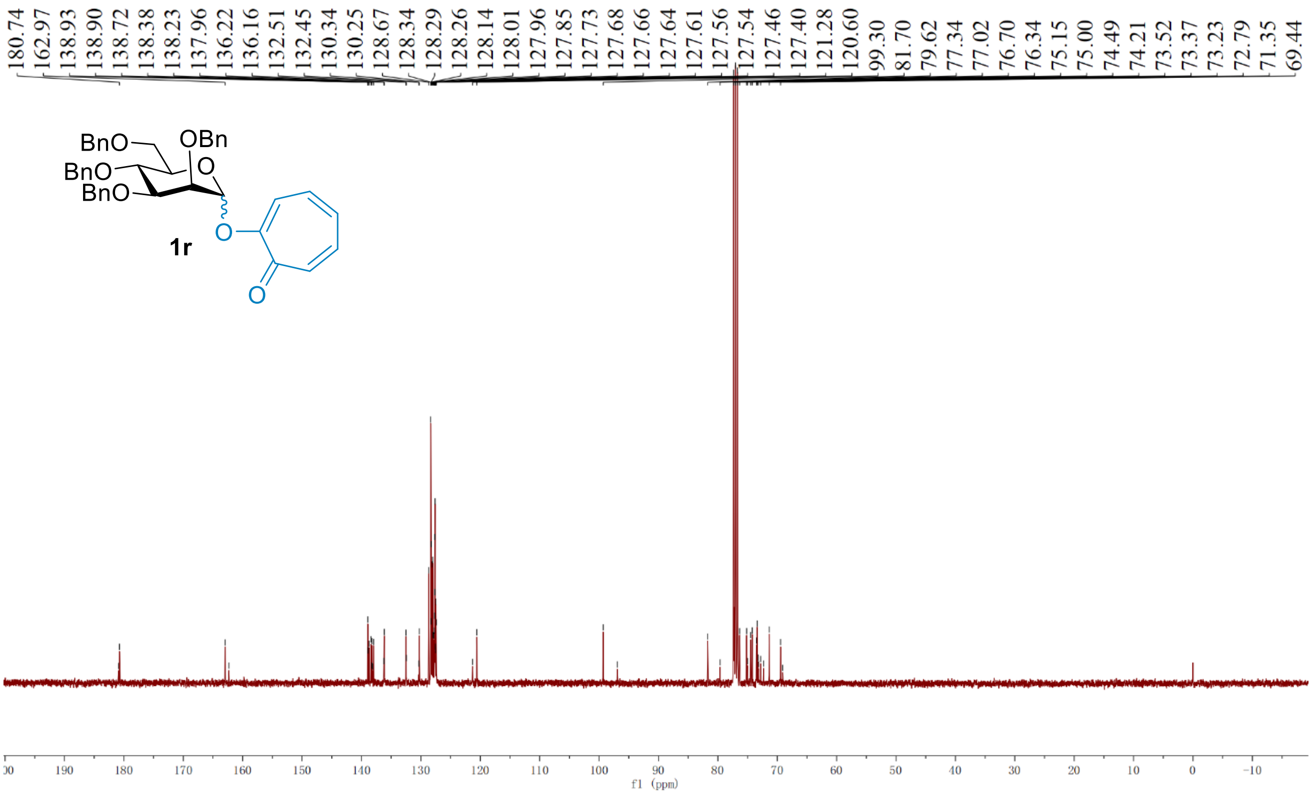

^{1}H NMR Spectrum of **1s** (400 MHz, $CDCl_3$)

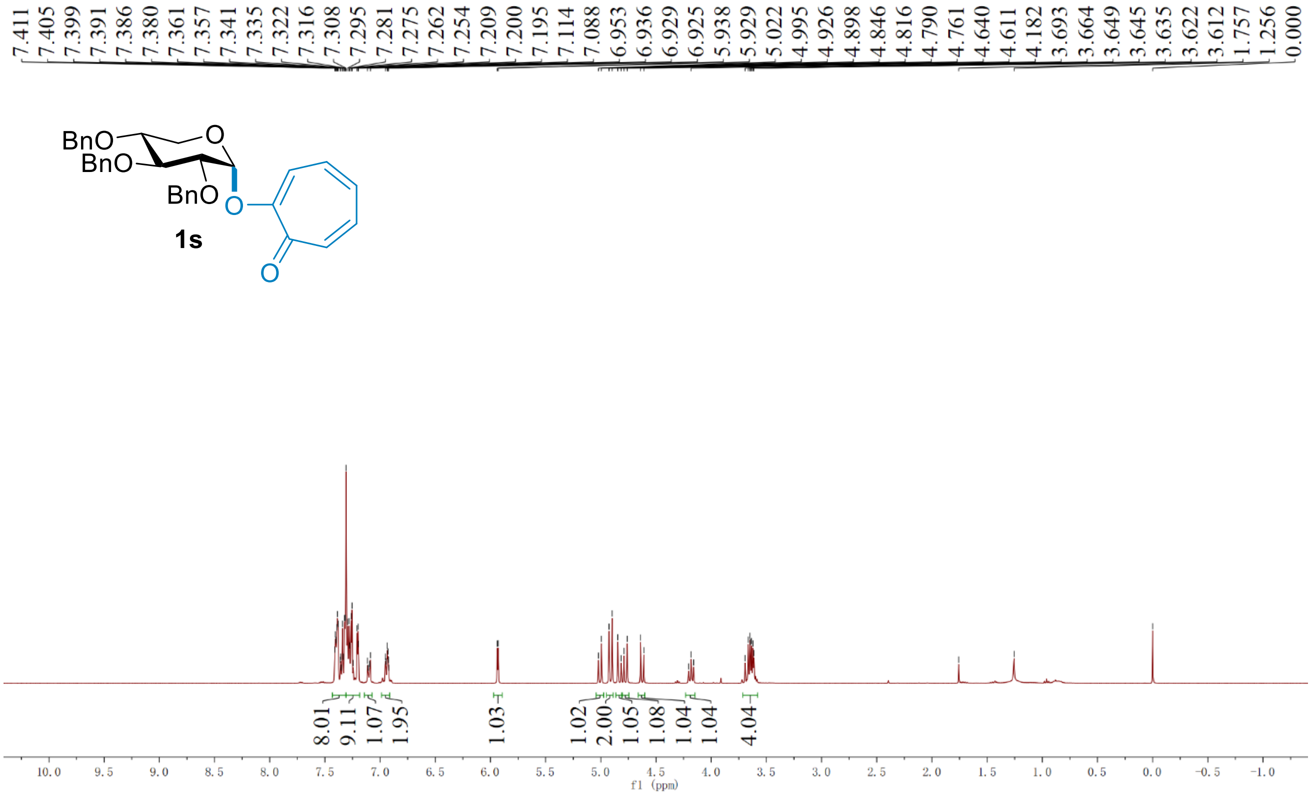

^{13}C NMR Spectrum of **1s** (101 MHz, $CDCl_3$)

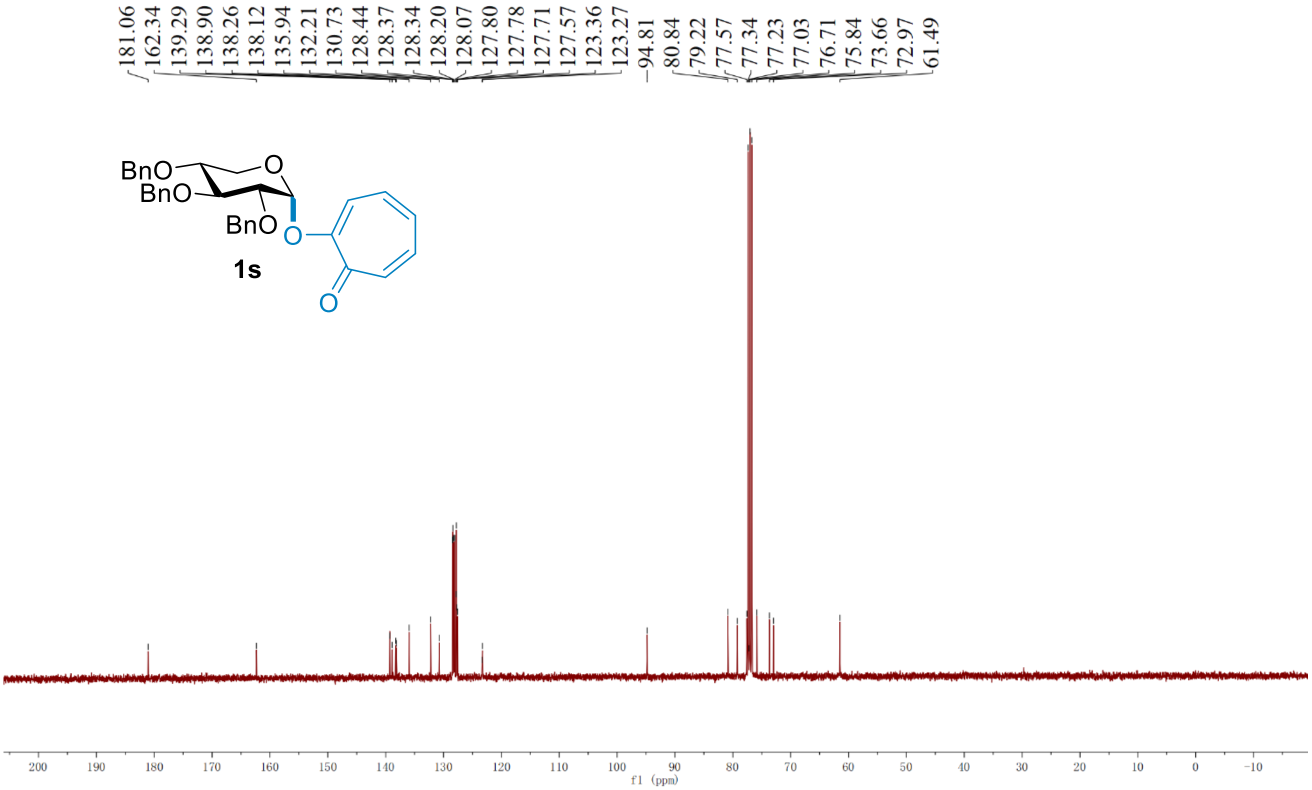

^{1}H NMR Spectrum of **1t** (400 MHz, $CDCl_3$)

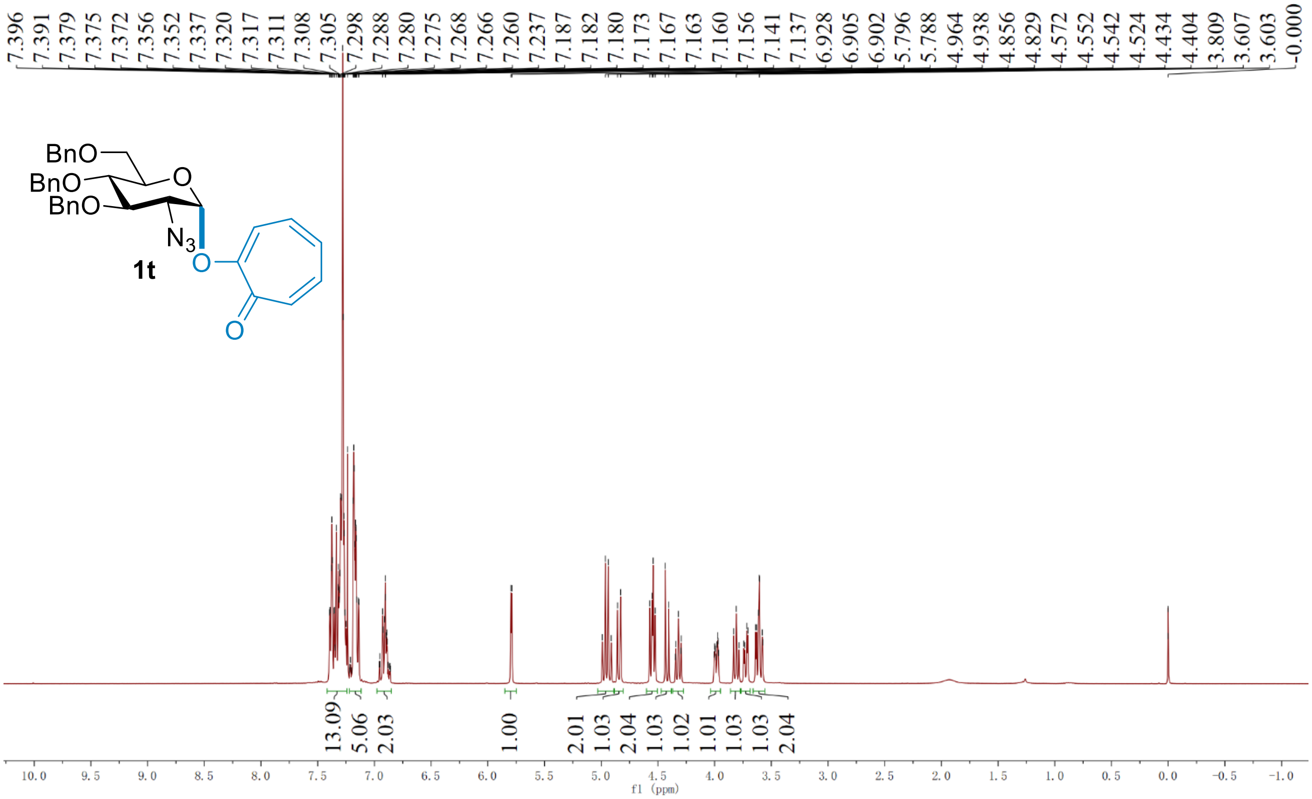

^{13}C NMR Spectrum of **1t** (101 MHz, $CDCl_3$)

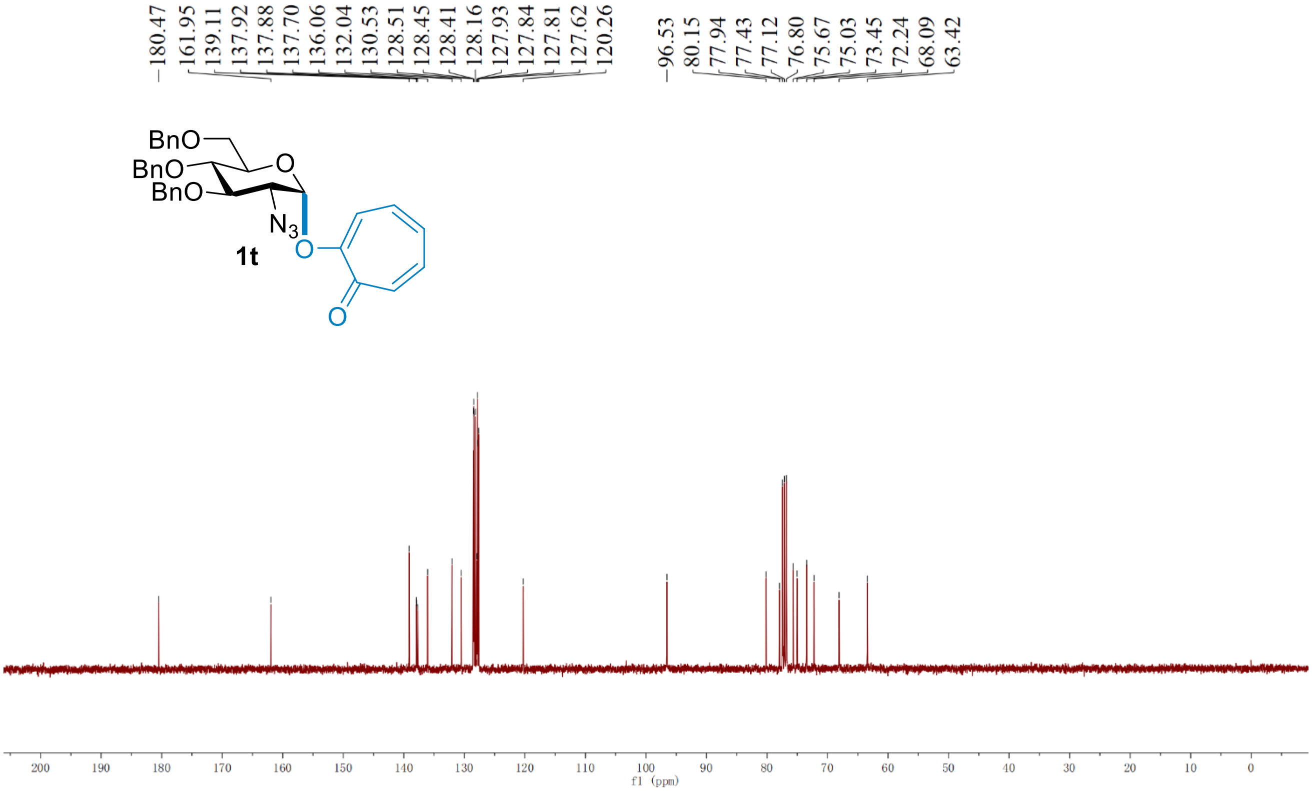

^{1}H NMR Spectrum of **2r** (400 MHz, $CDCl_3$)

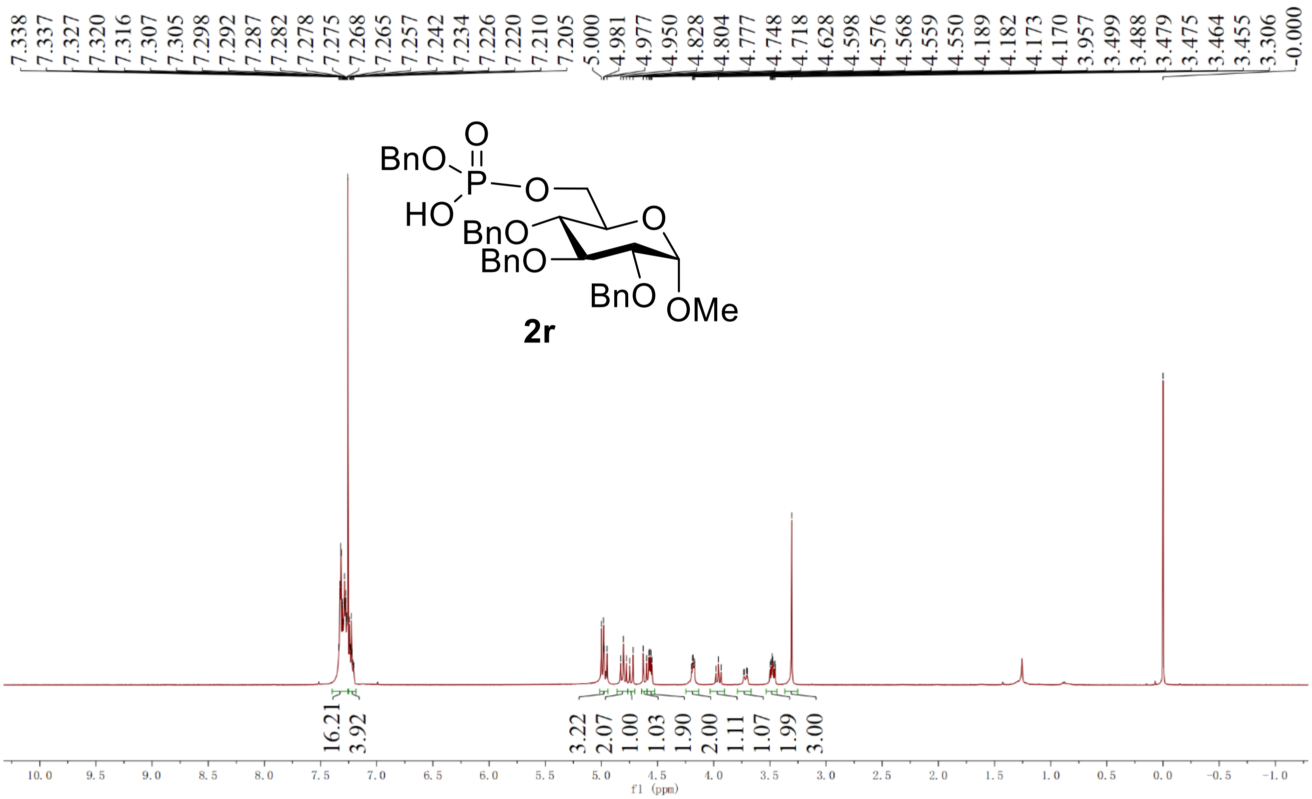

^{31}P NMR Spectrum of **2r** (162 MHz, $CDCl_3$)

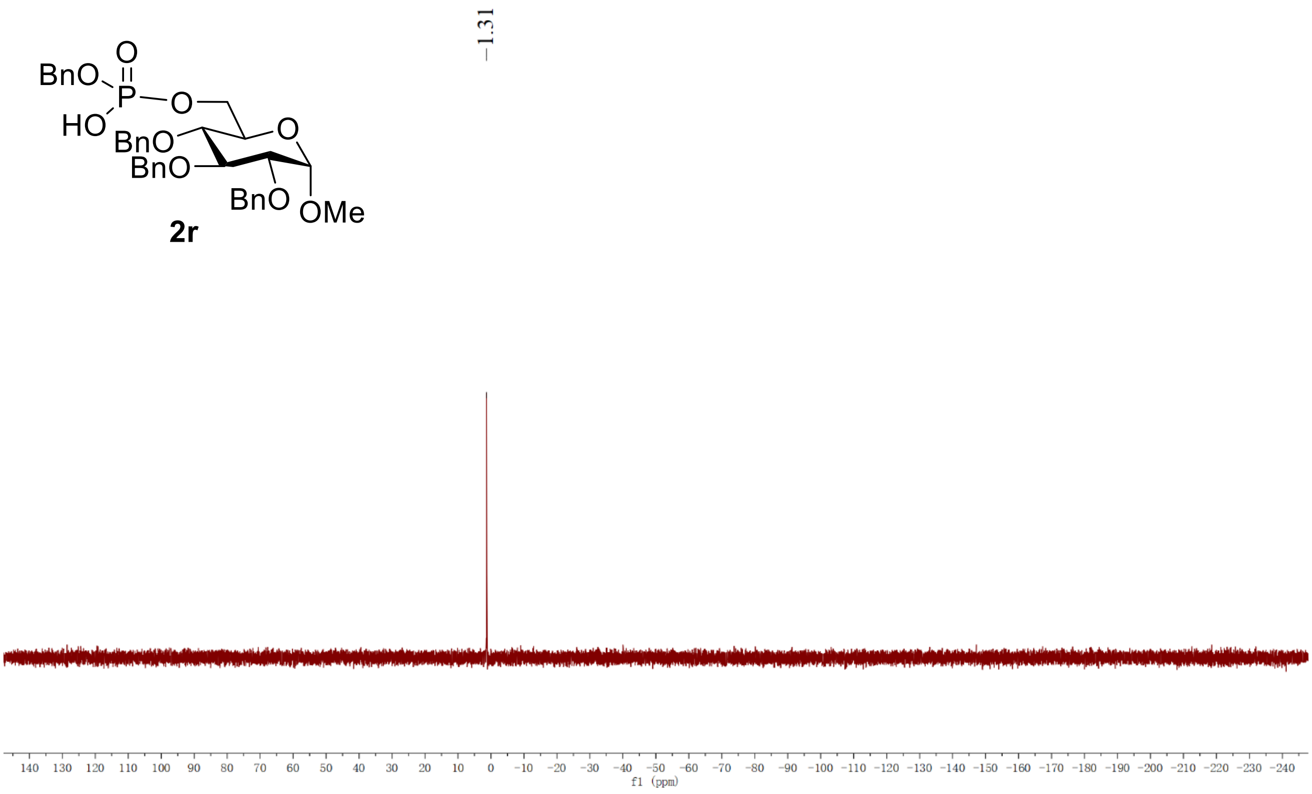

^{13}C NMR Spectrum of **2r** (101 MHz, $CDCl_3$)

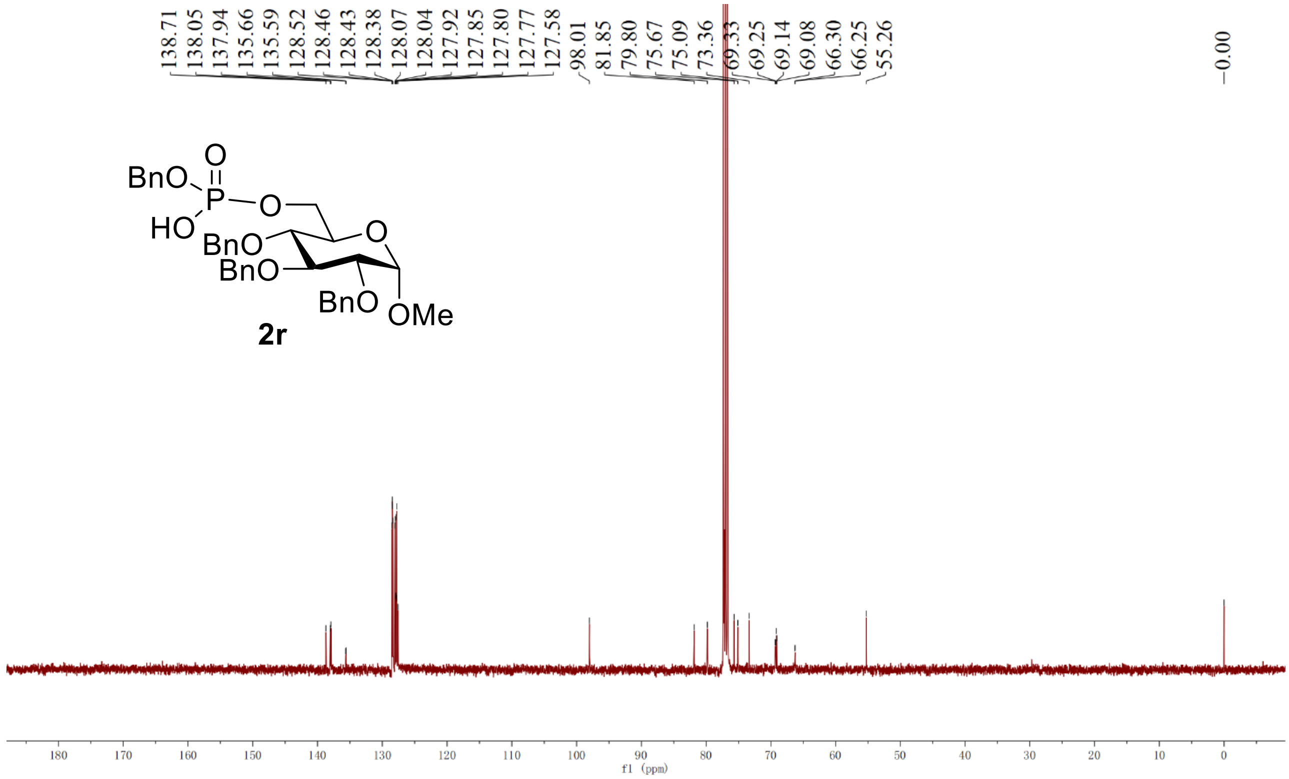

^1H NMR Spectrum of **2s** (400 MHz, $CDCl_3$)

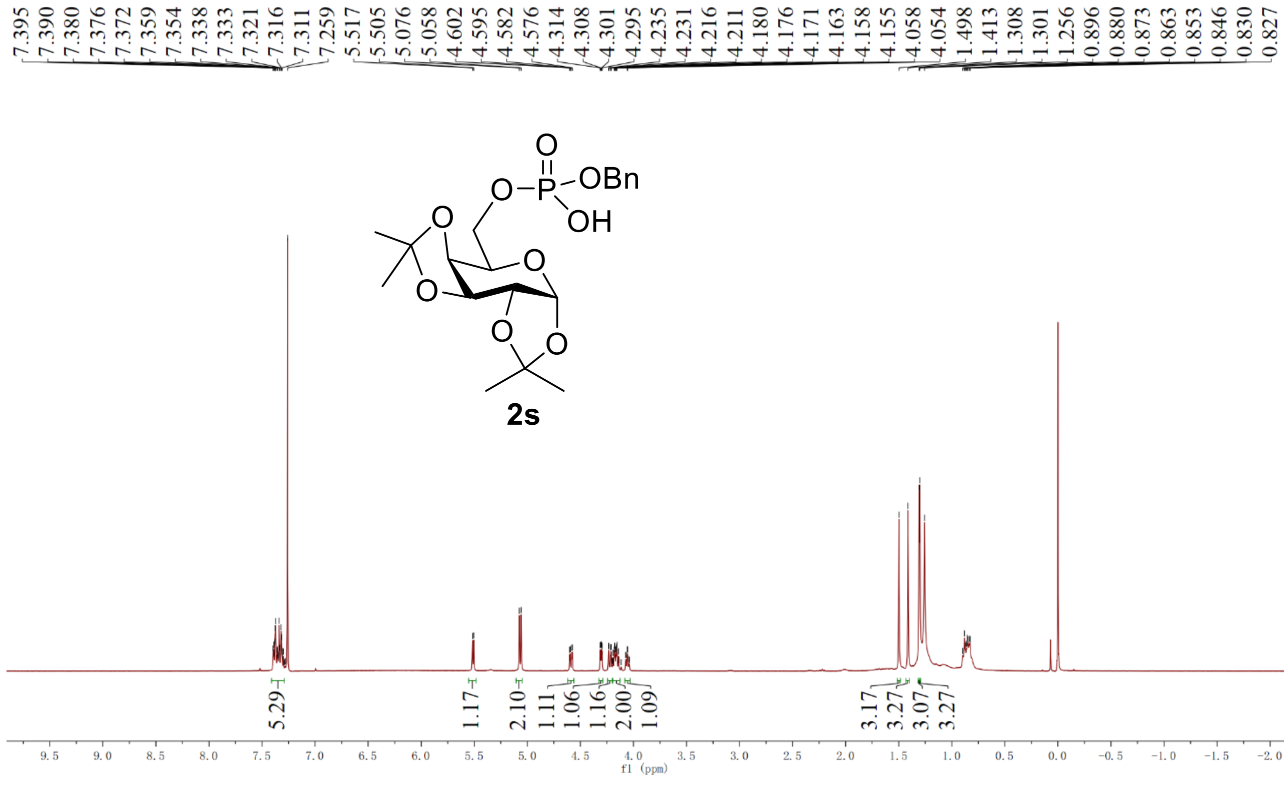

^{31}P NMR Spectrum of **2s** (162 MHz, $CDCl_3$)

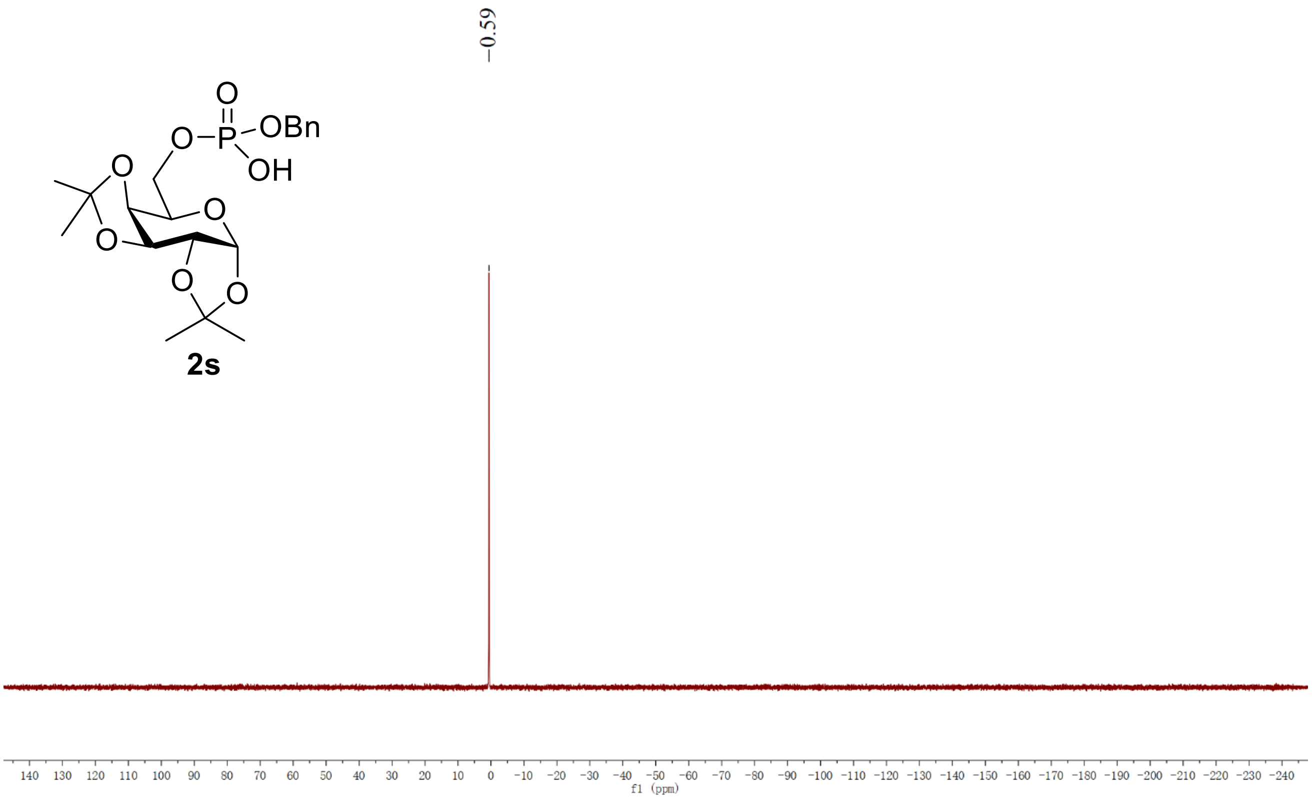

^{1}H NMR Spectrum of **2t** (400 MHz, $CDCl_3$)

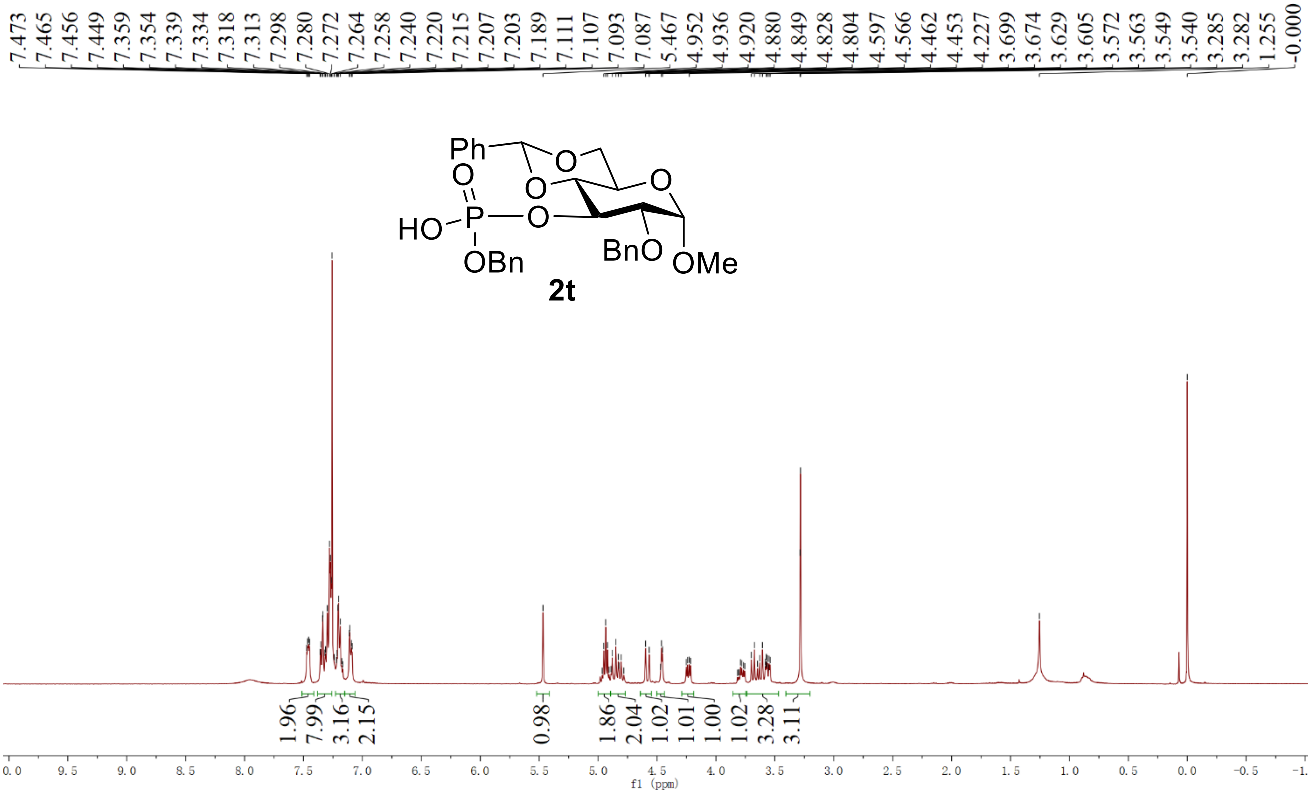

^{31}P NMR Spectrum of **2t** (162 MHz, $CDCl_3$)

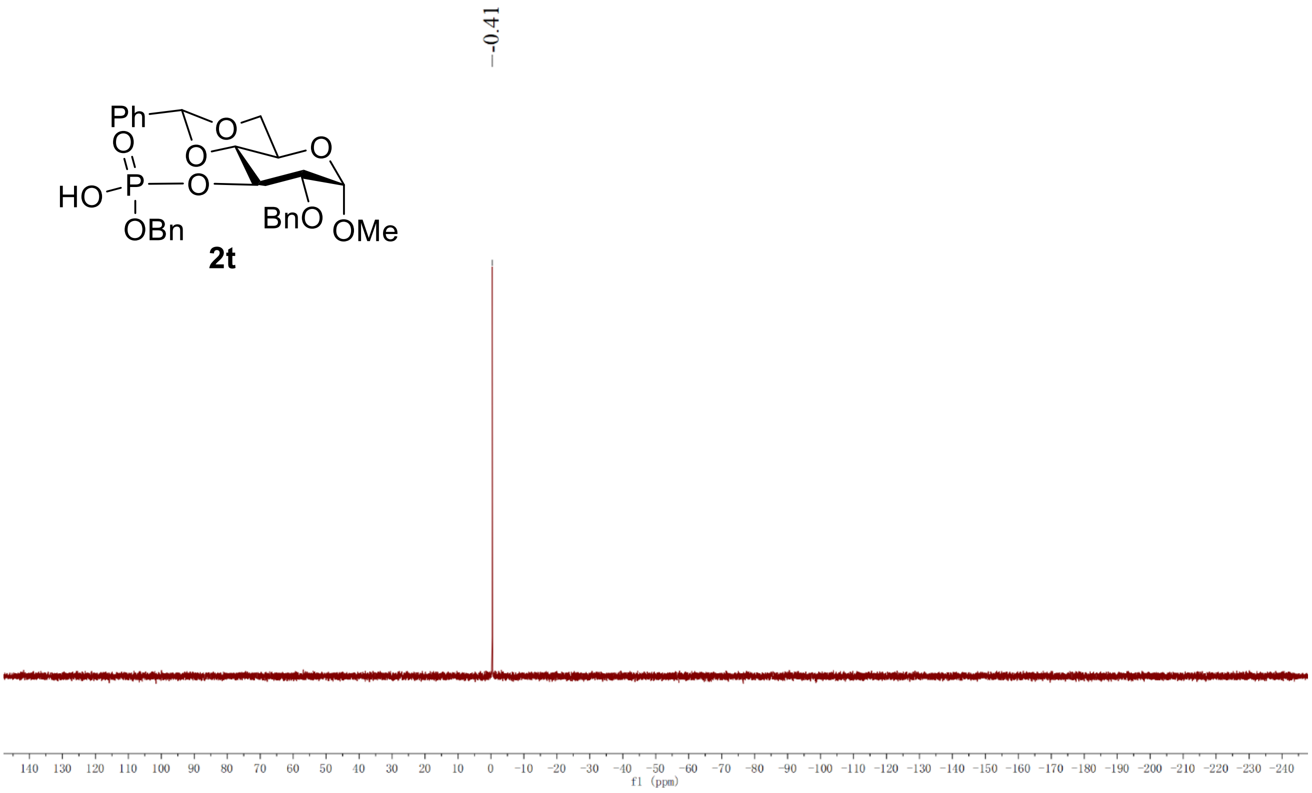

^{13}C NMR Spectrum of **2t** (101 MHz, $CDCl_3$)

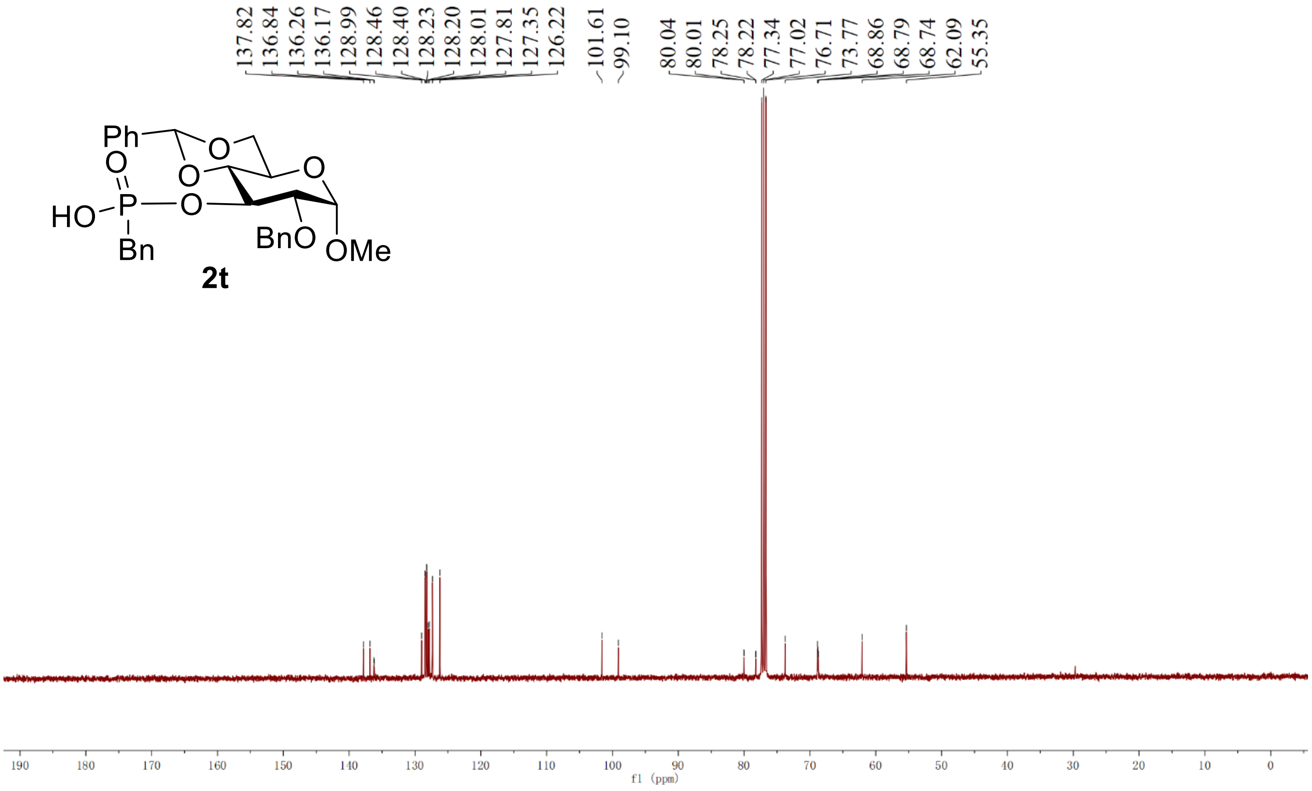

^{1}H NMR Spectrum of **2u** (400 MHz, $CDCl_3$)

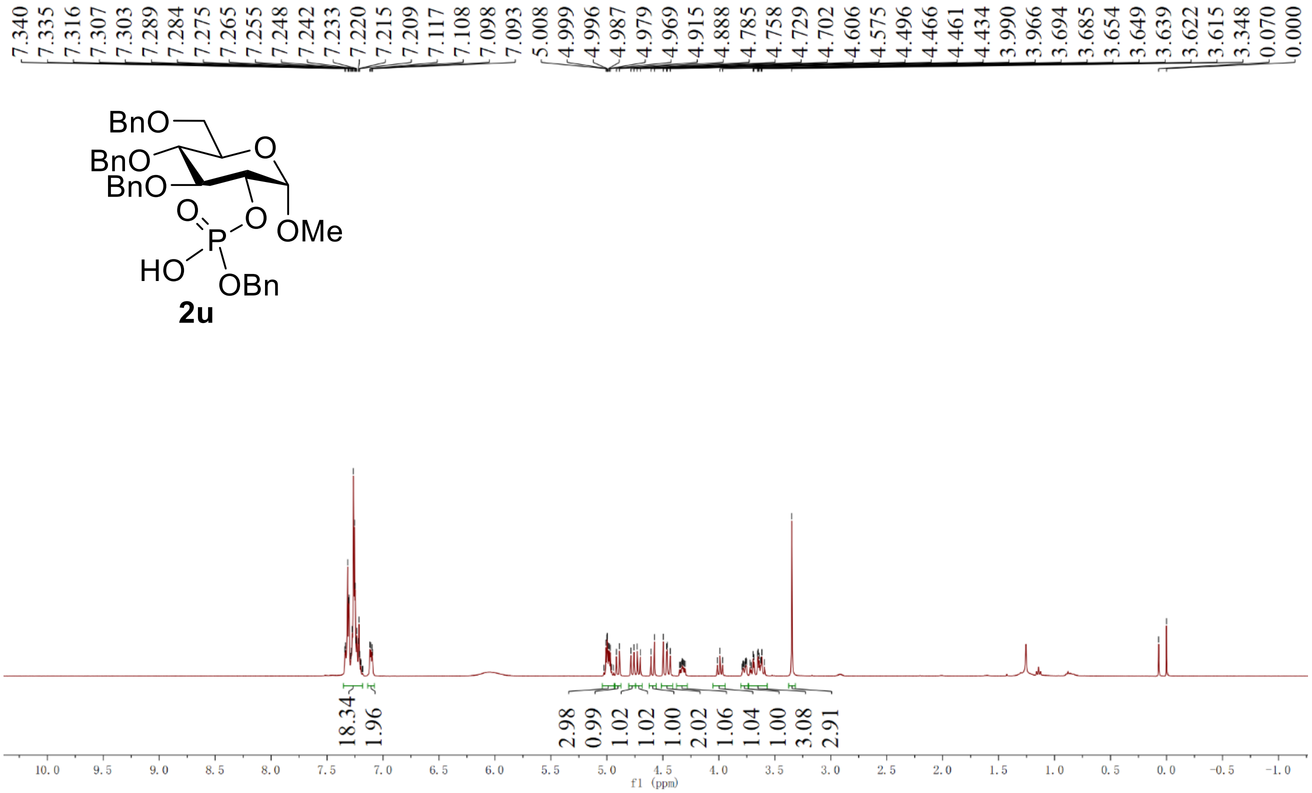

^{31}P NMR Spectrum of **2u** (162 MHz, $CDCl_3$)

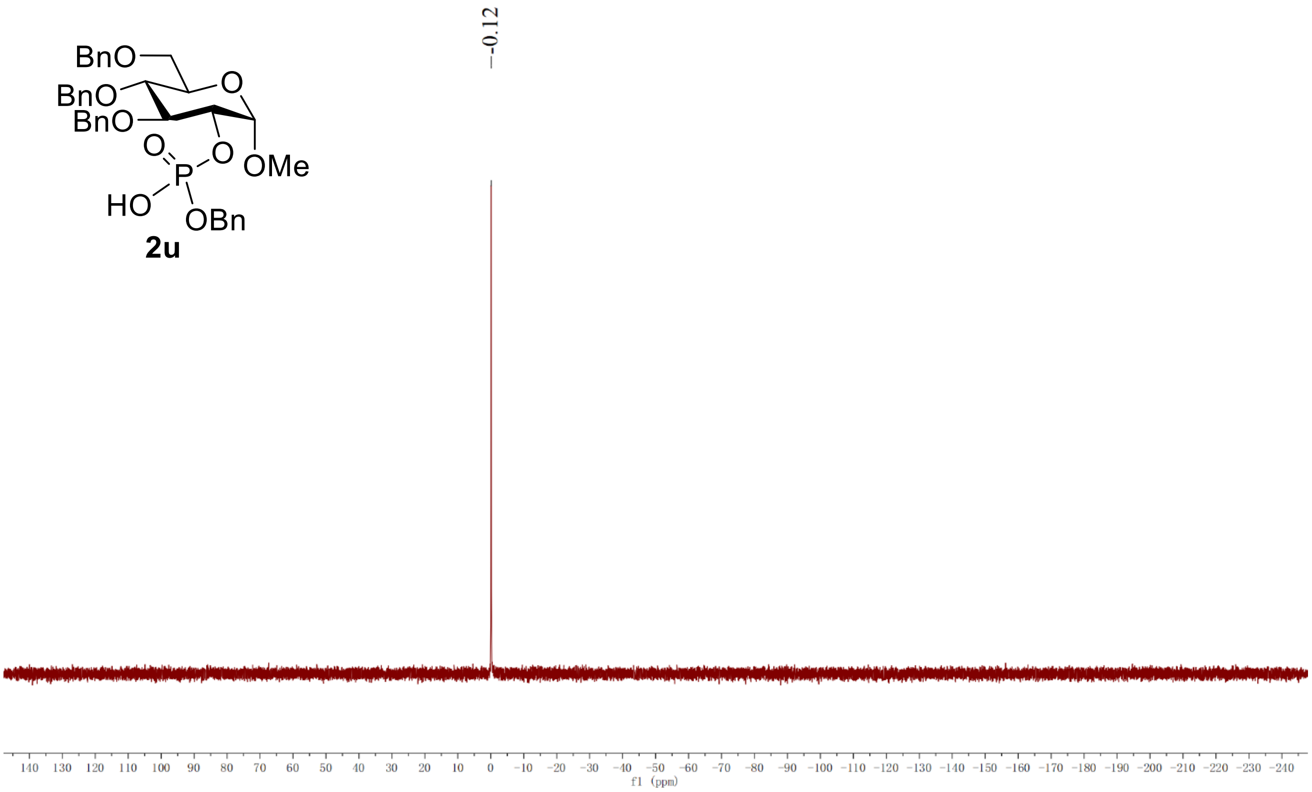

^{13}CNMR Spectrum of **2u** (101 MHz, $CDCl_3$)

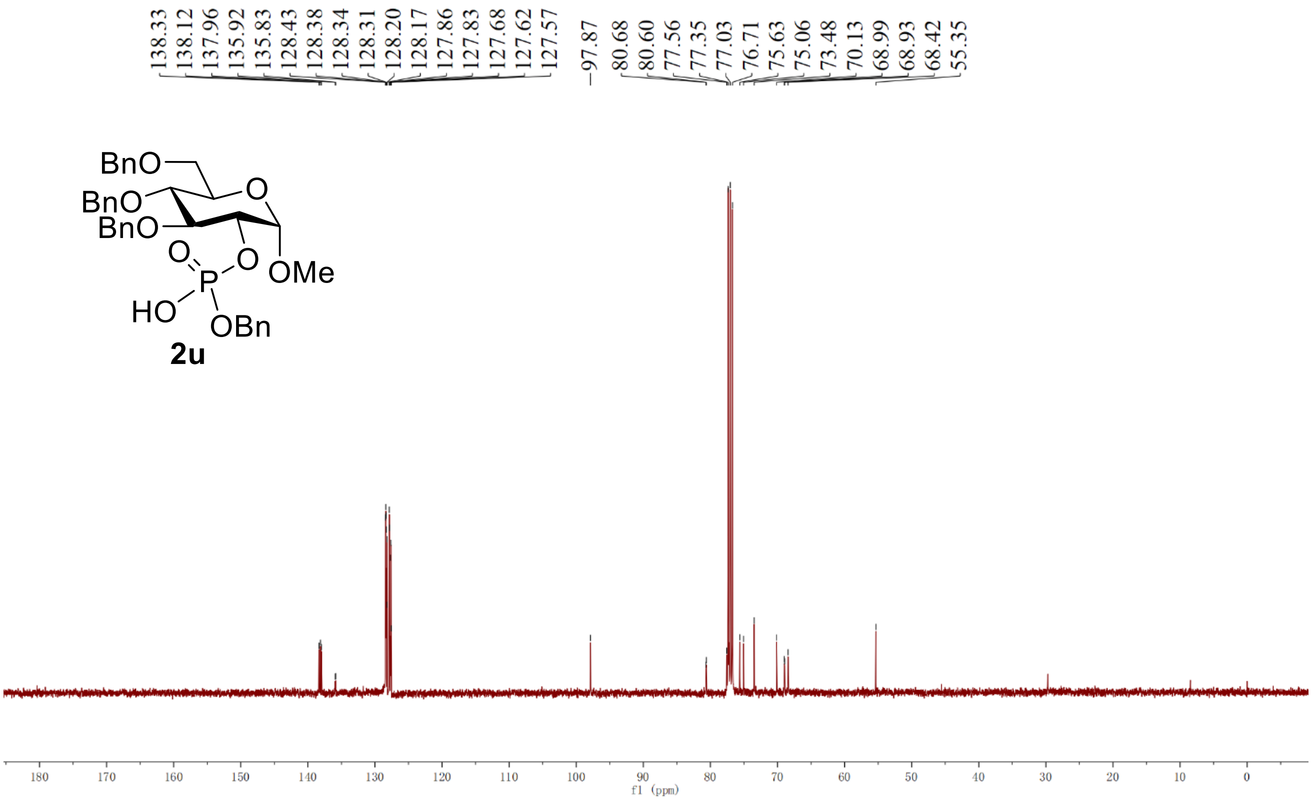

^{1}H NMR Spectrum of **2v** (400 MHz, $CDCl_3$)

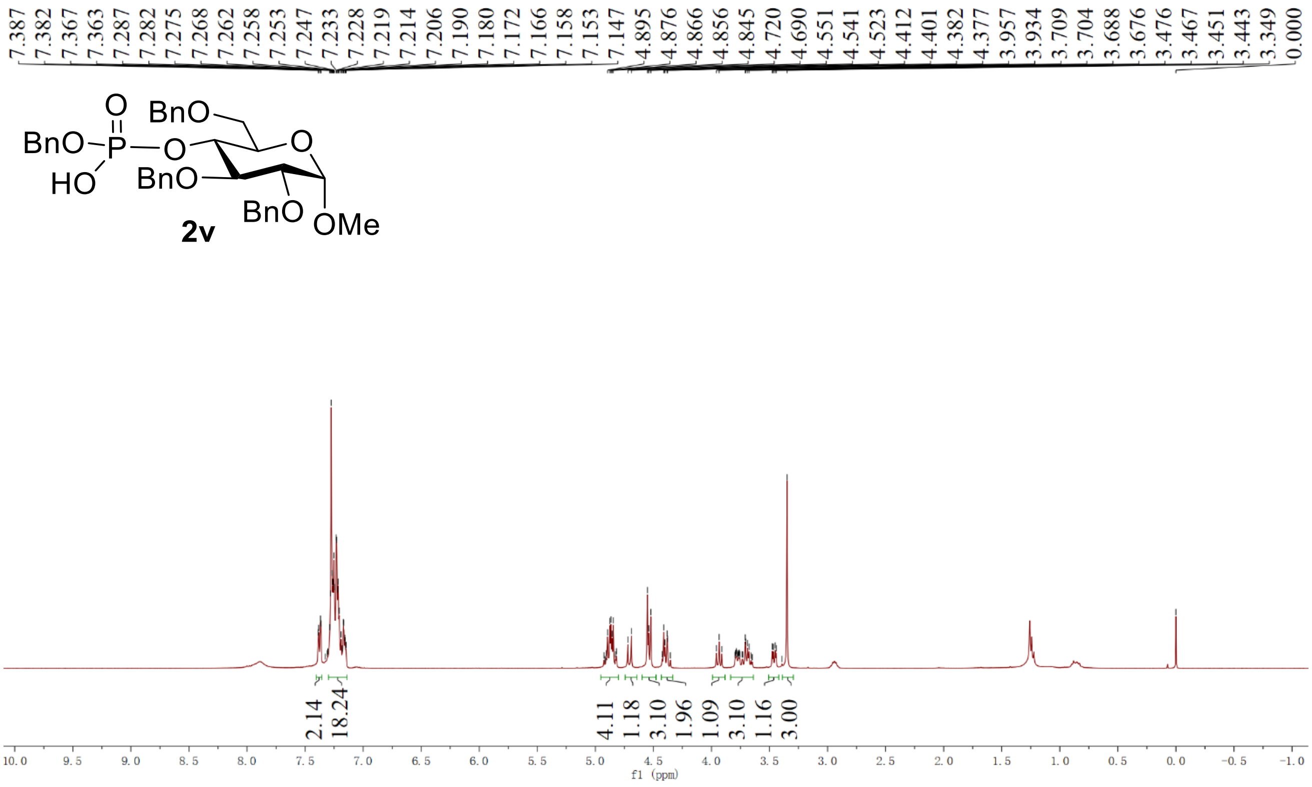

^{31}P NMR Spectrum of **2v** (162 MHz, $CDCl_3$)

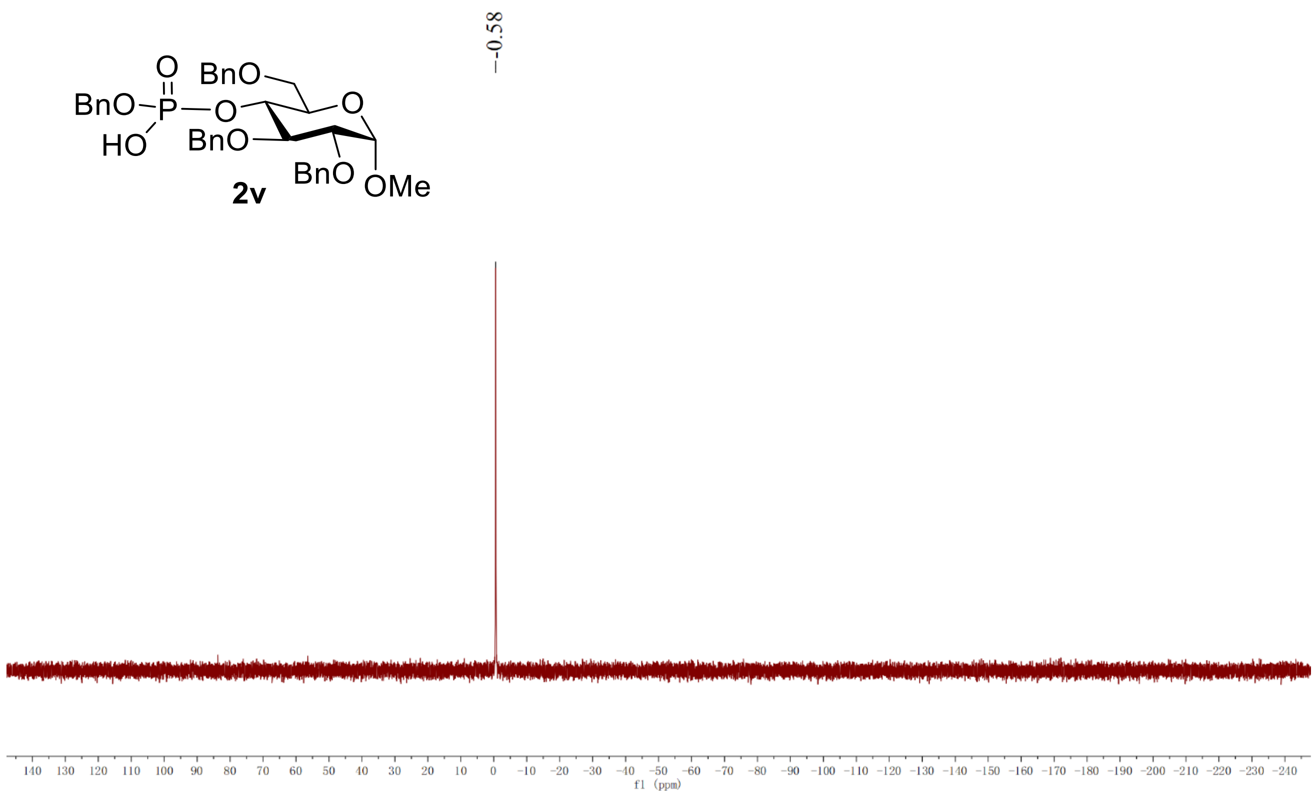

^{1}H NMR Spectrum of **2w** (400 MHz, $CDCl_3$)

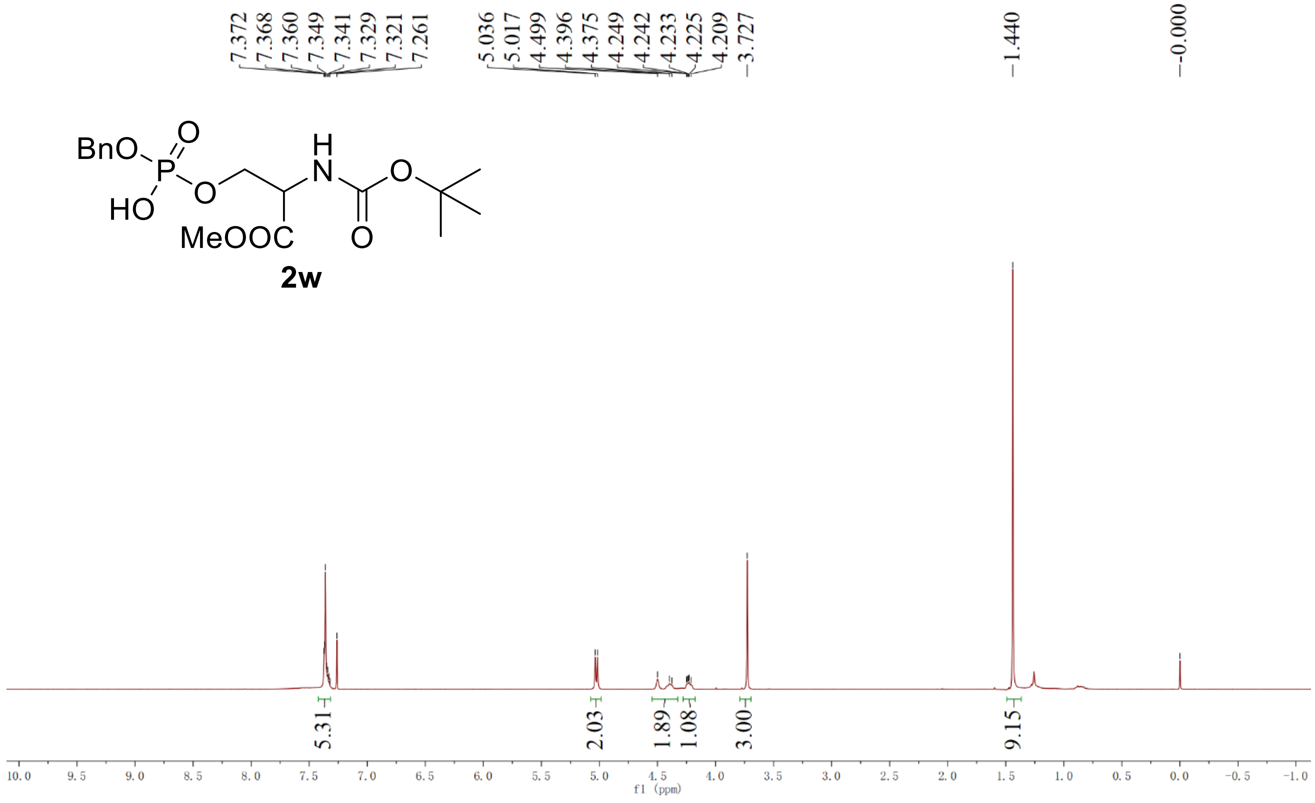

^{31}P NMR Spectrum of **2w** (162 MHz, $CDCl_3$)

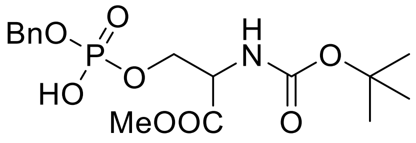

2w

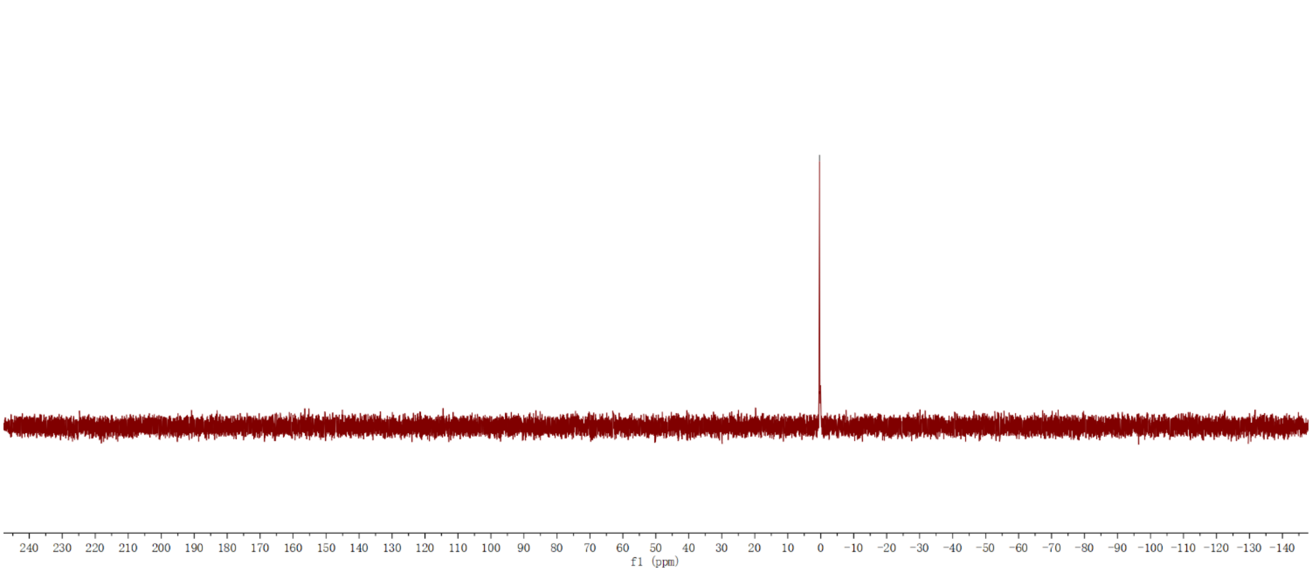

^{13}C NMR Spectrum of **2w** (101 MHz, $CDCl_3$)

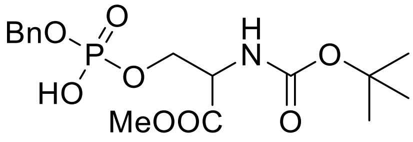

2w

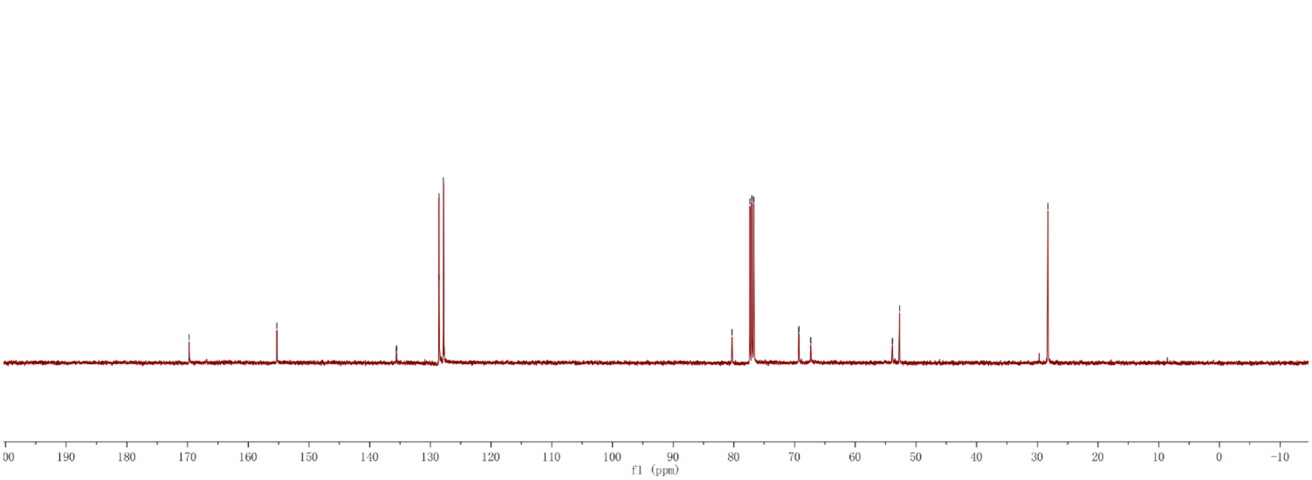

^{1}H NMR Spectrum of **2x** (400 MHz, CD_3OD)

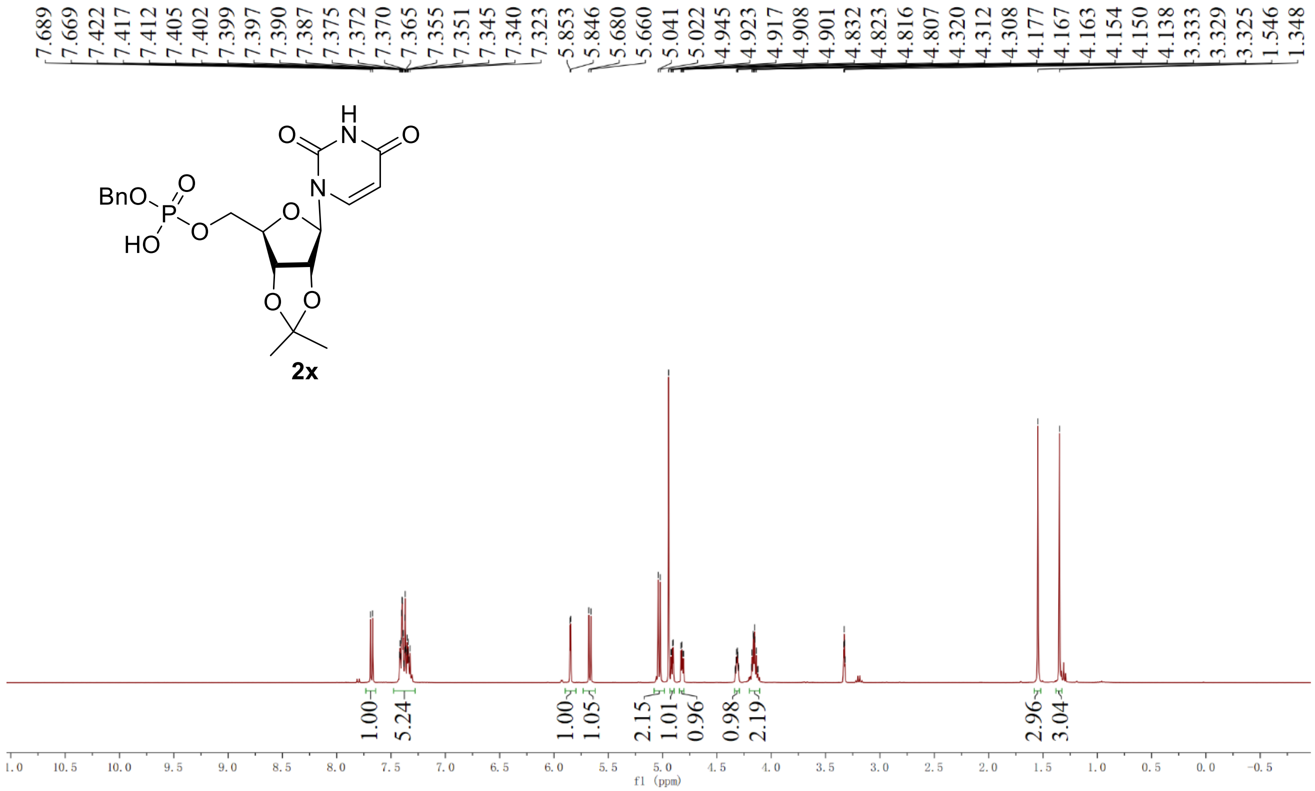

^{31}P NMR Spectrum of **2x** (162 MHz, CD_3OD)

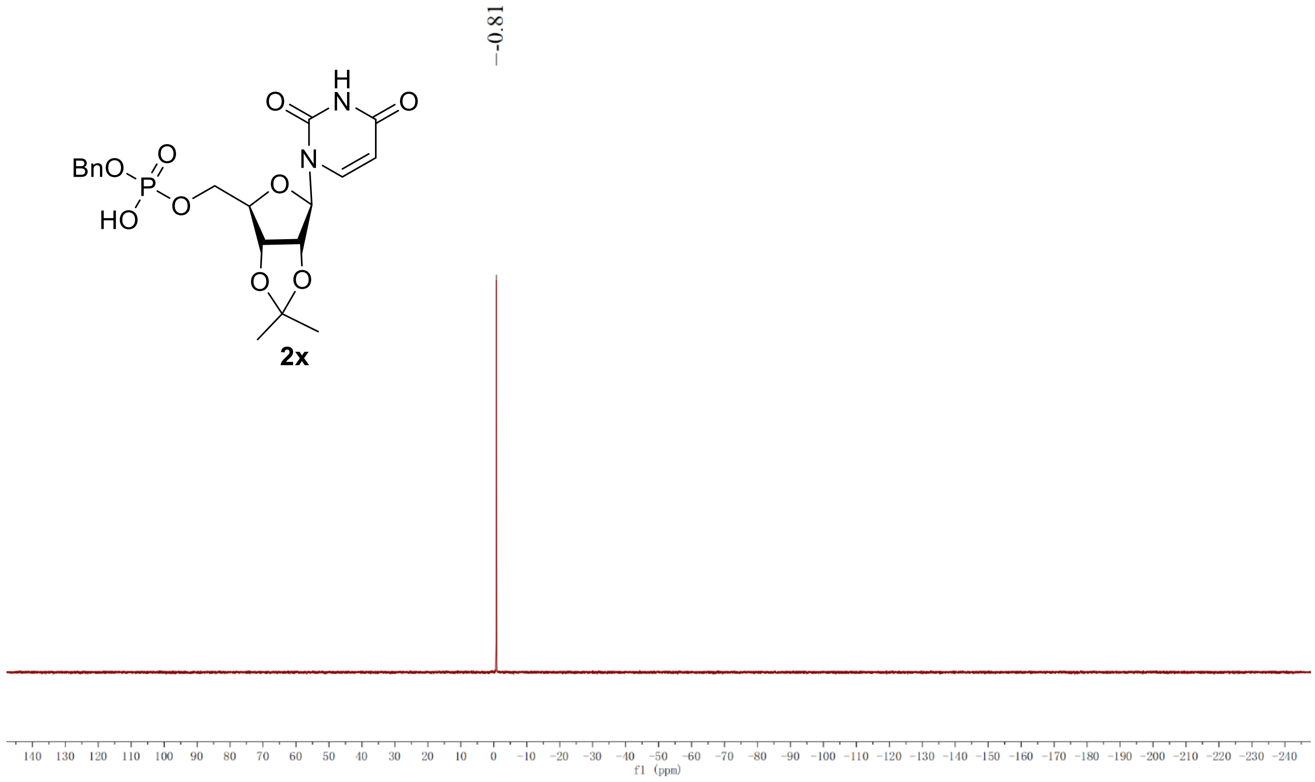

^{13}C NMR Spectrum of **2x** (101 MHz, CD_3OD)

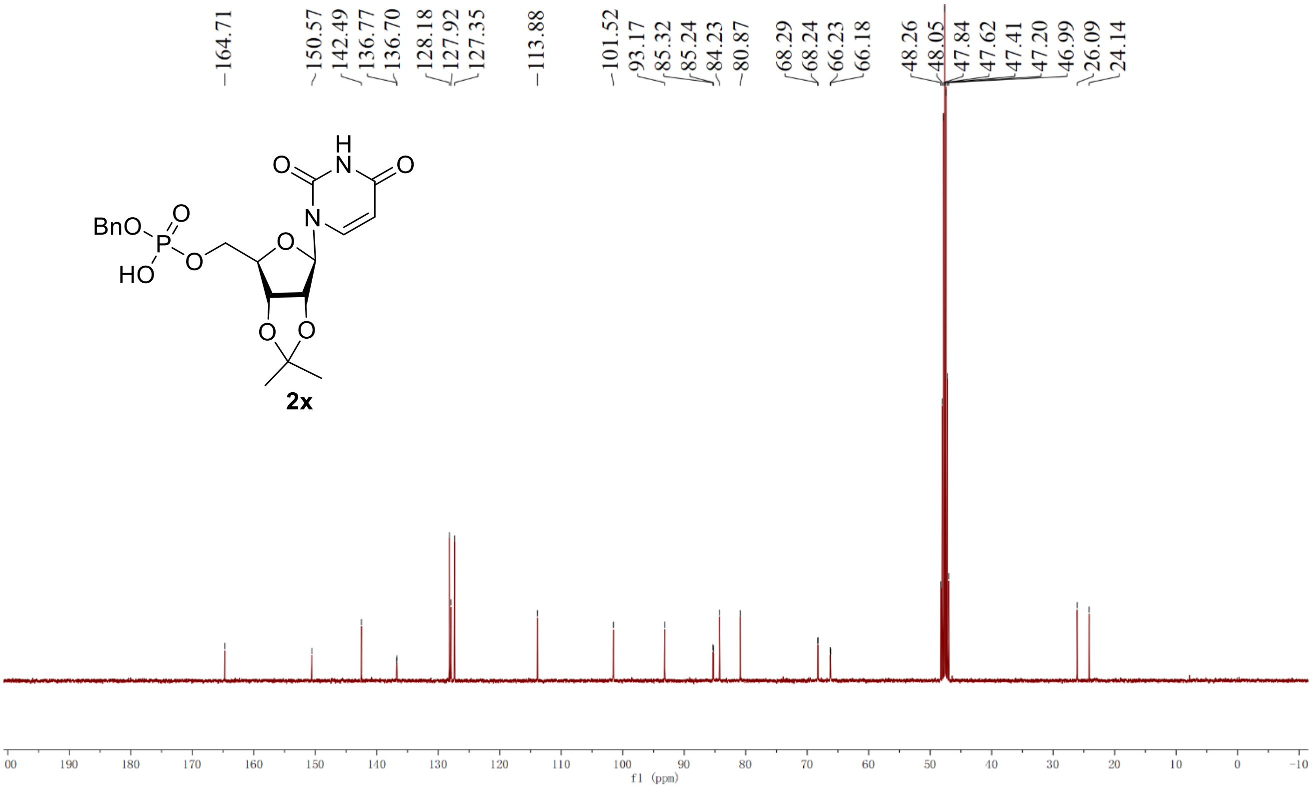

^{1}H NMR Spectrum of **3a** (400 MHz, $CDCl_3$)

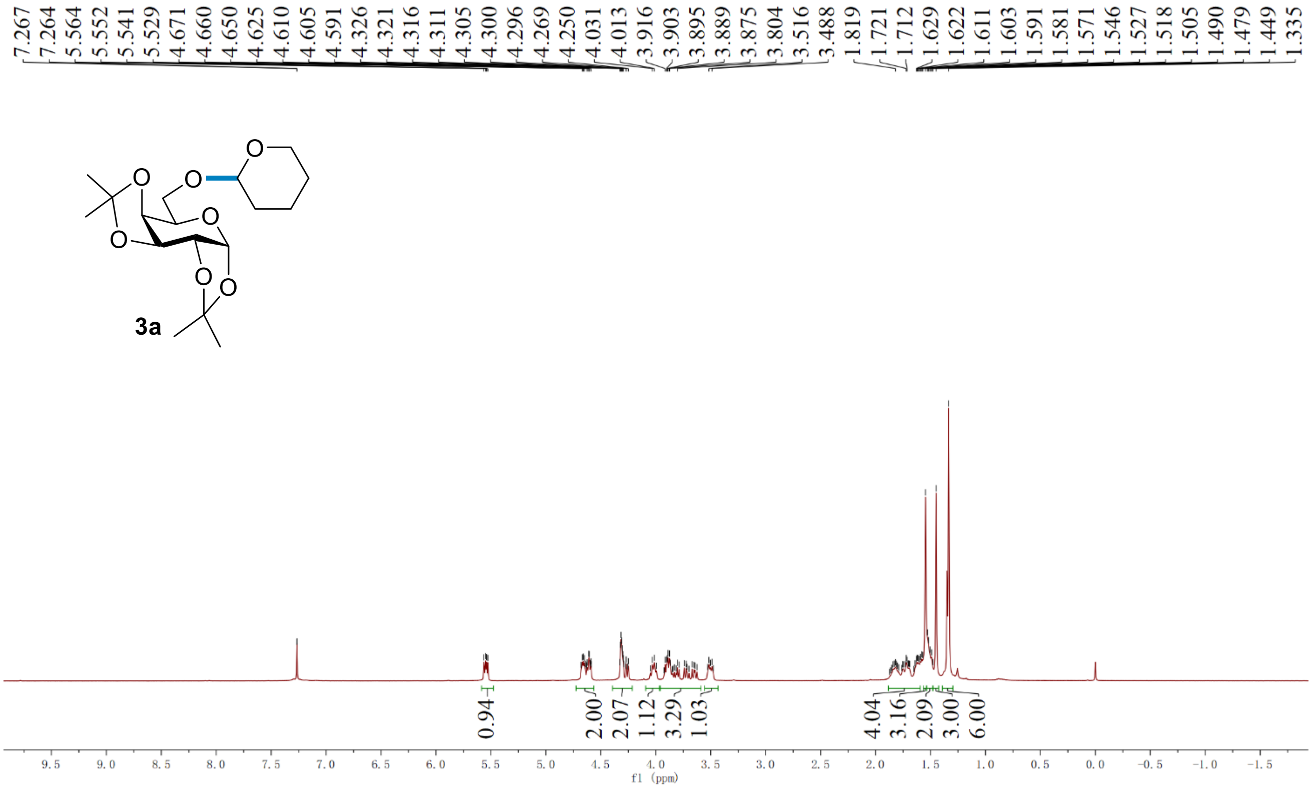

^{13}C NMR Spectrum of **3a** (101 MHz, $CDCl_3$)

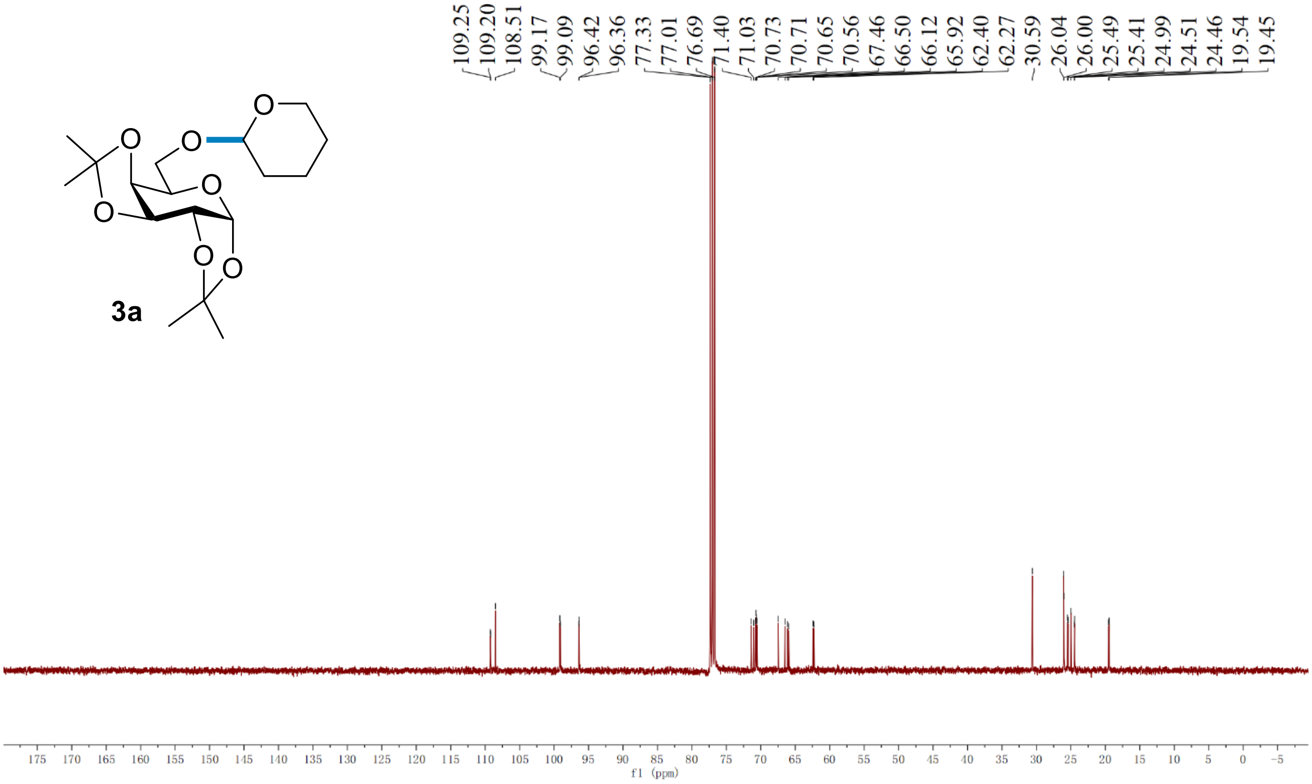

^{1}H NMR Spectrum of **3b** (400 MHz, $CDCl_3$)

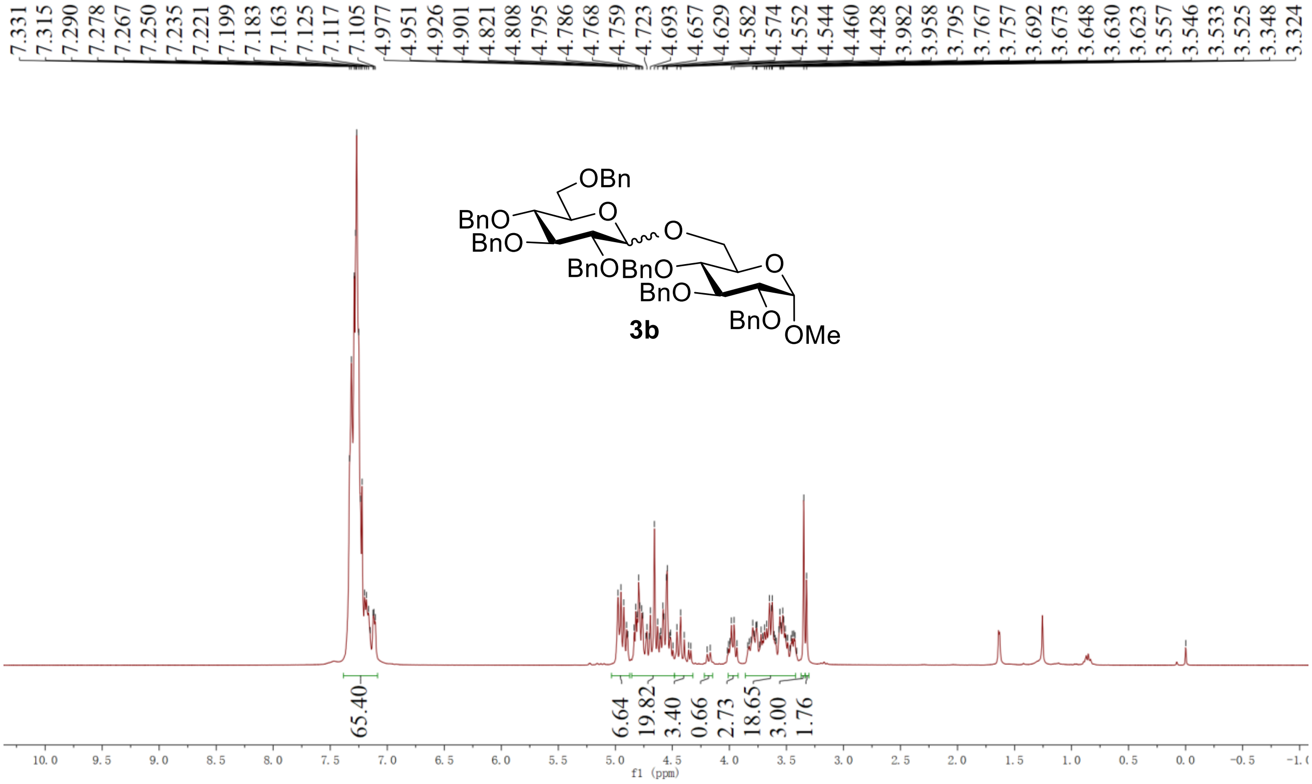

^{1}H NMR Spectrum of **3c** (400 MHz, $CDCl_3$)

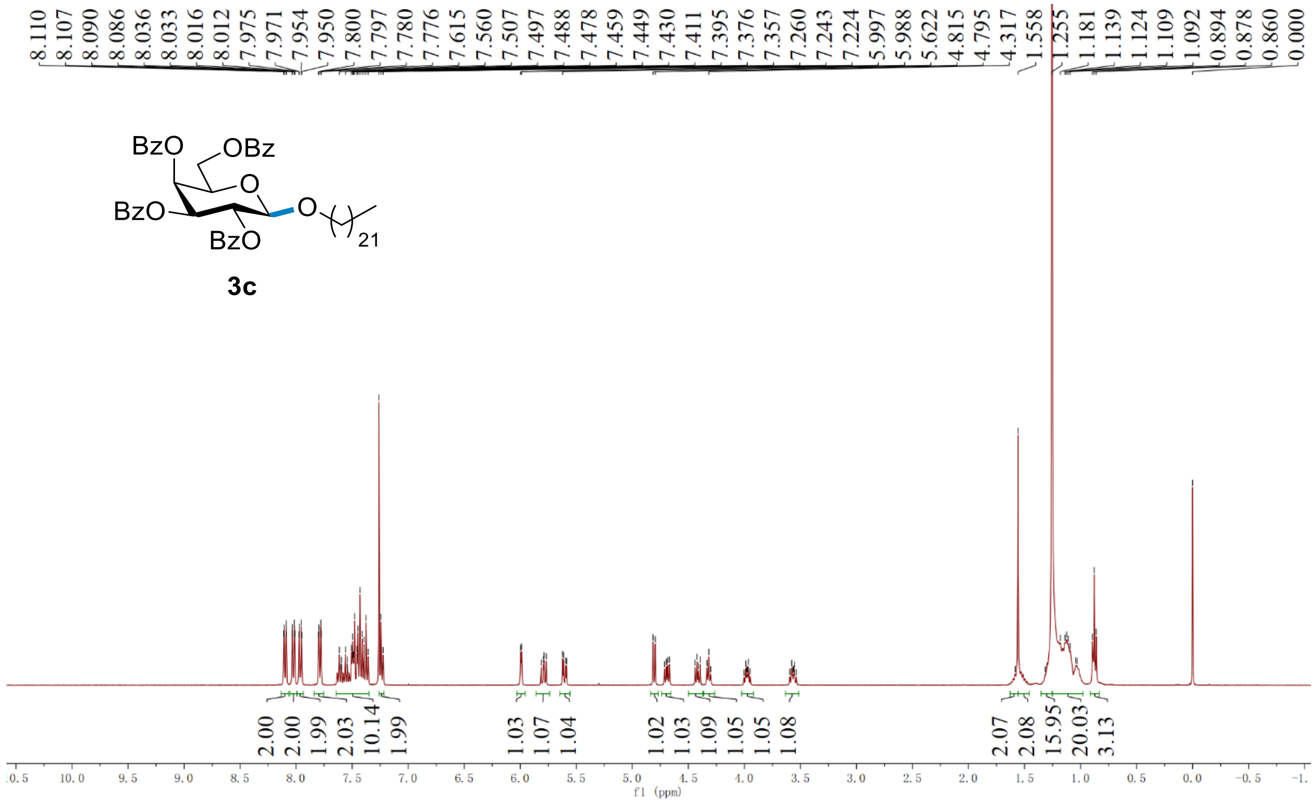

^{13}C NMR Spectrum of **3c** (101 MHz, $CDCl_3$)

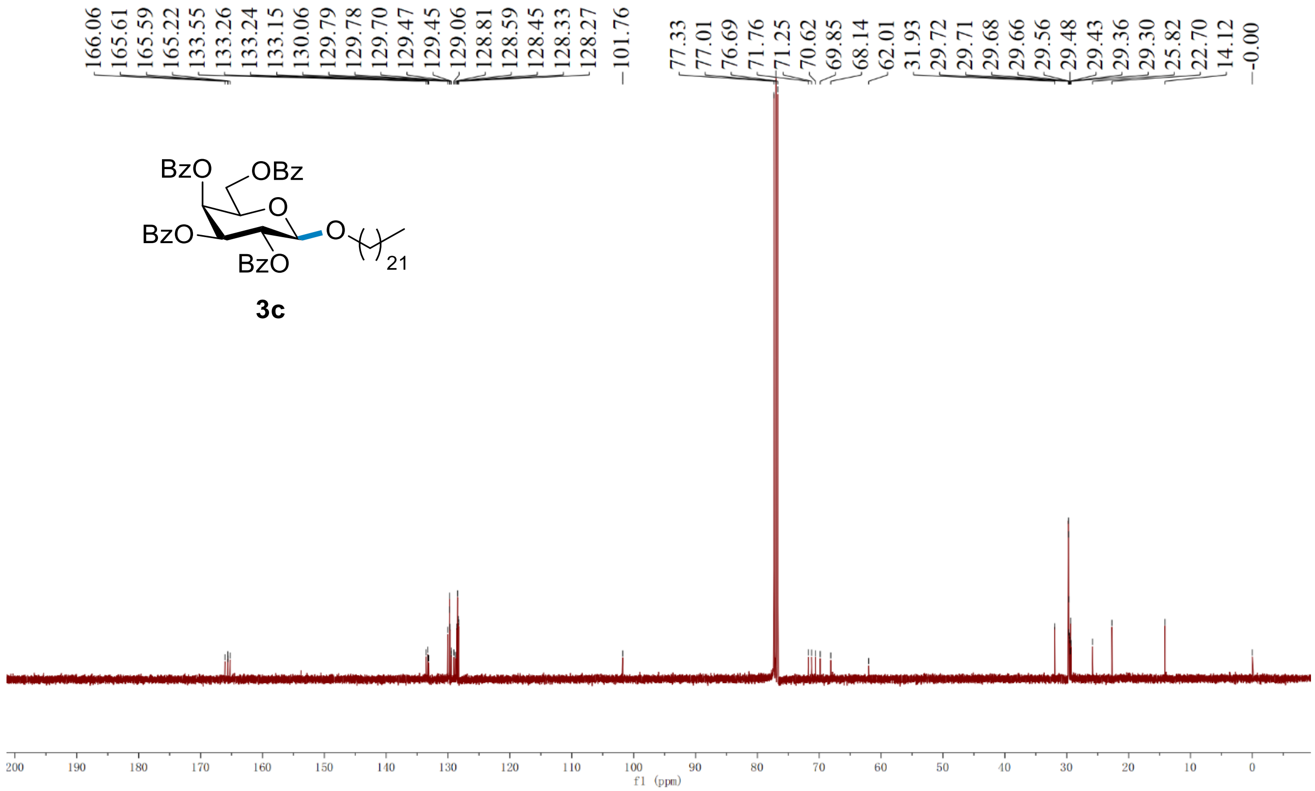

^{1}H NMR Spectrum of **3d** (400 MHz, $CDCl_3$)

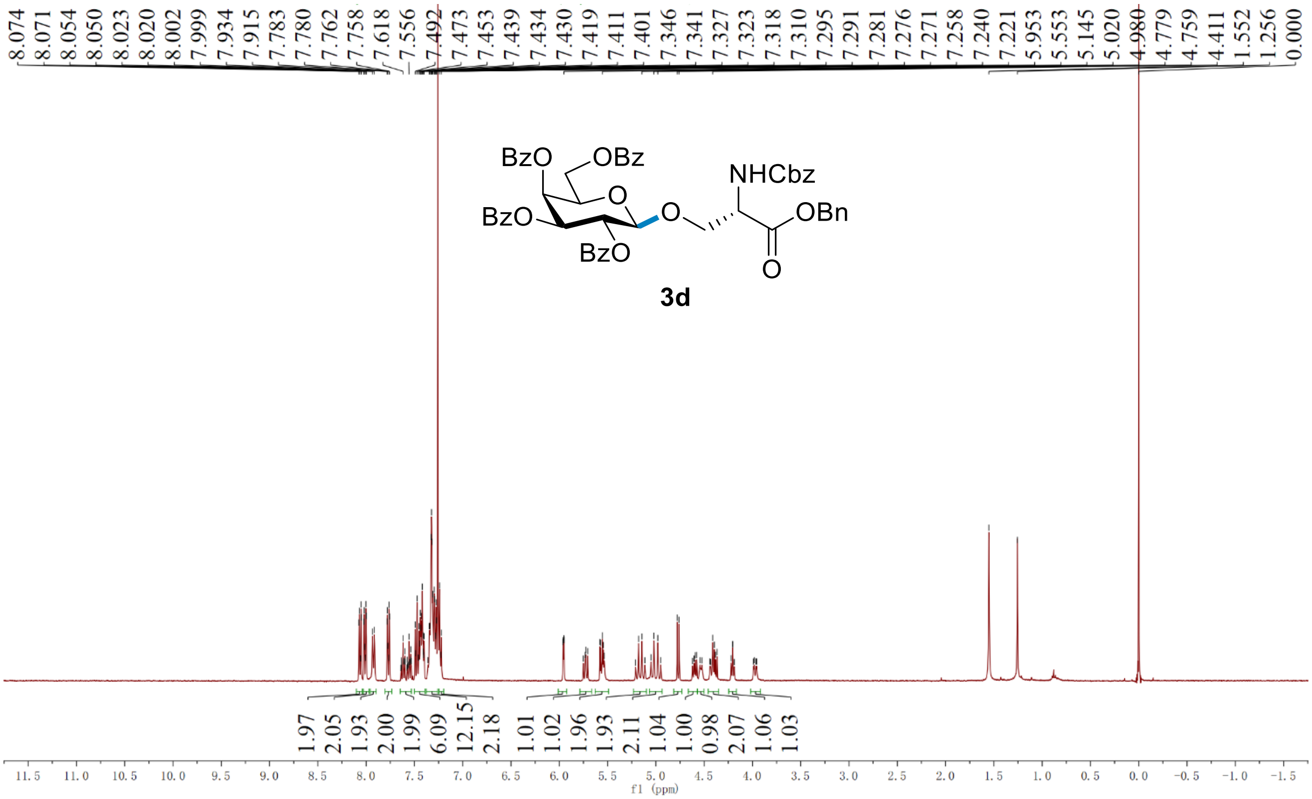

^{1}H NMR Spectrum of **3e** (400 MHz, $CDCl_3$)

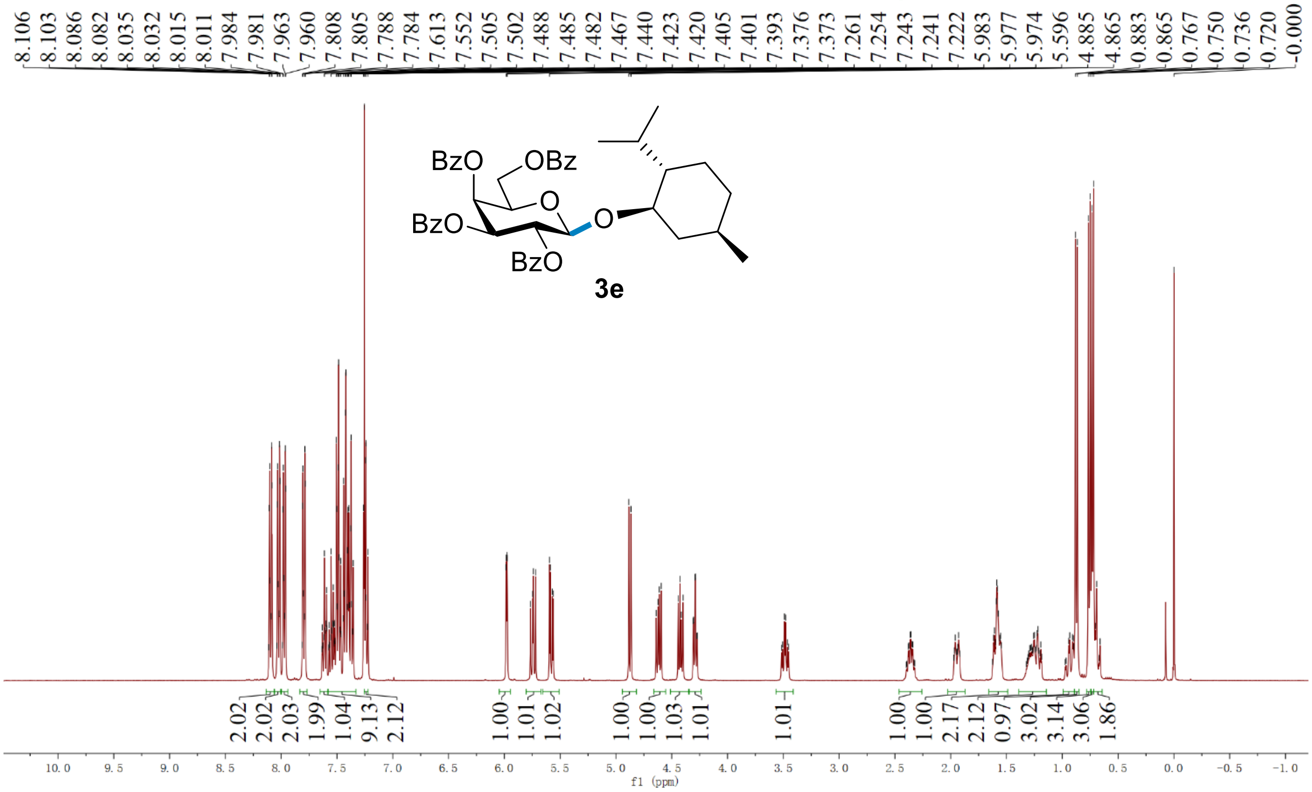

^{1}H NMR Spectrum of **3f** (400 MHz, $CDCl_3$)

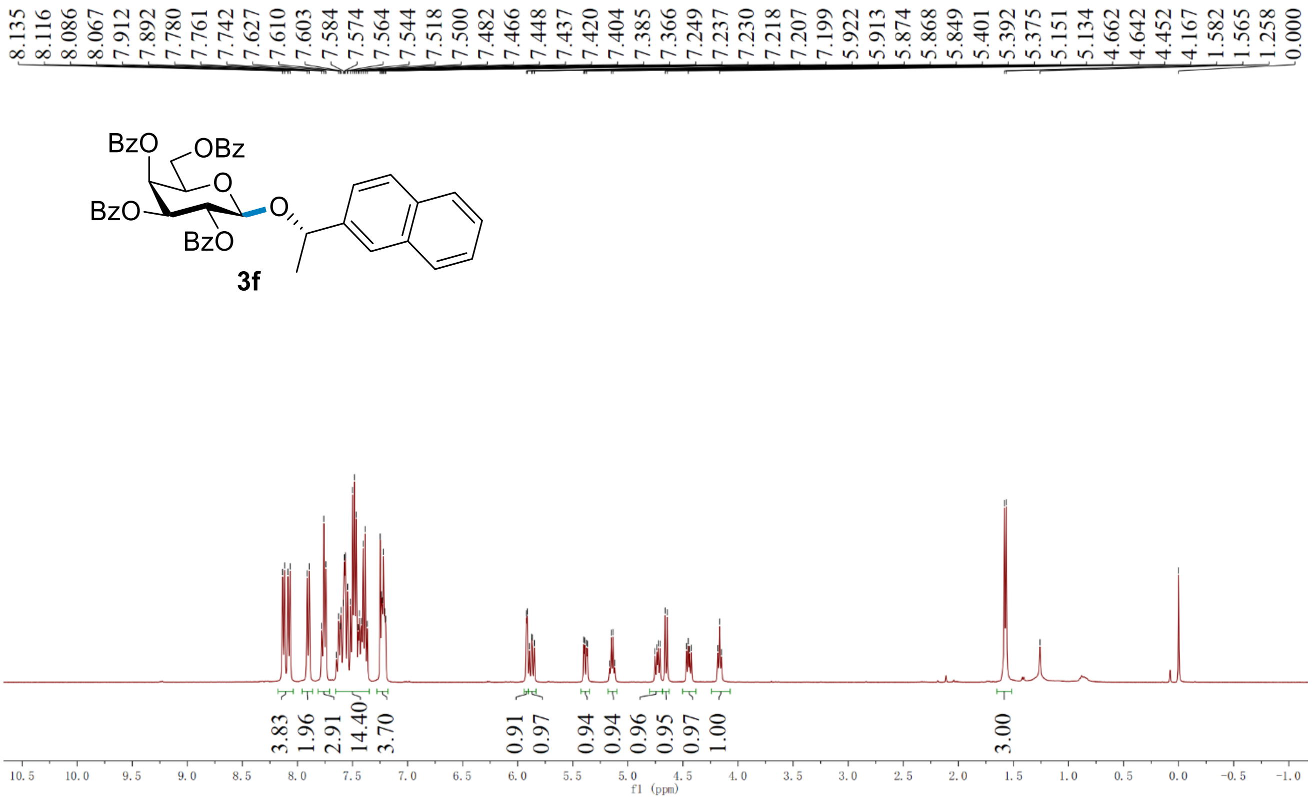

^{13}C NMR Spectrum of **3f** (101 MHz, $CDCl_3$)

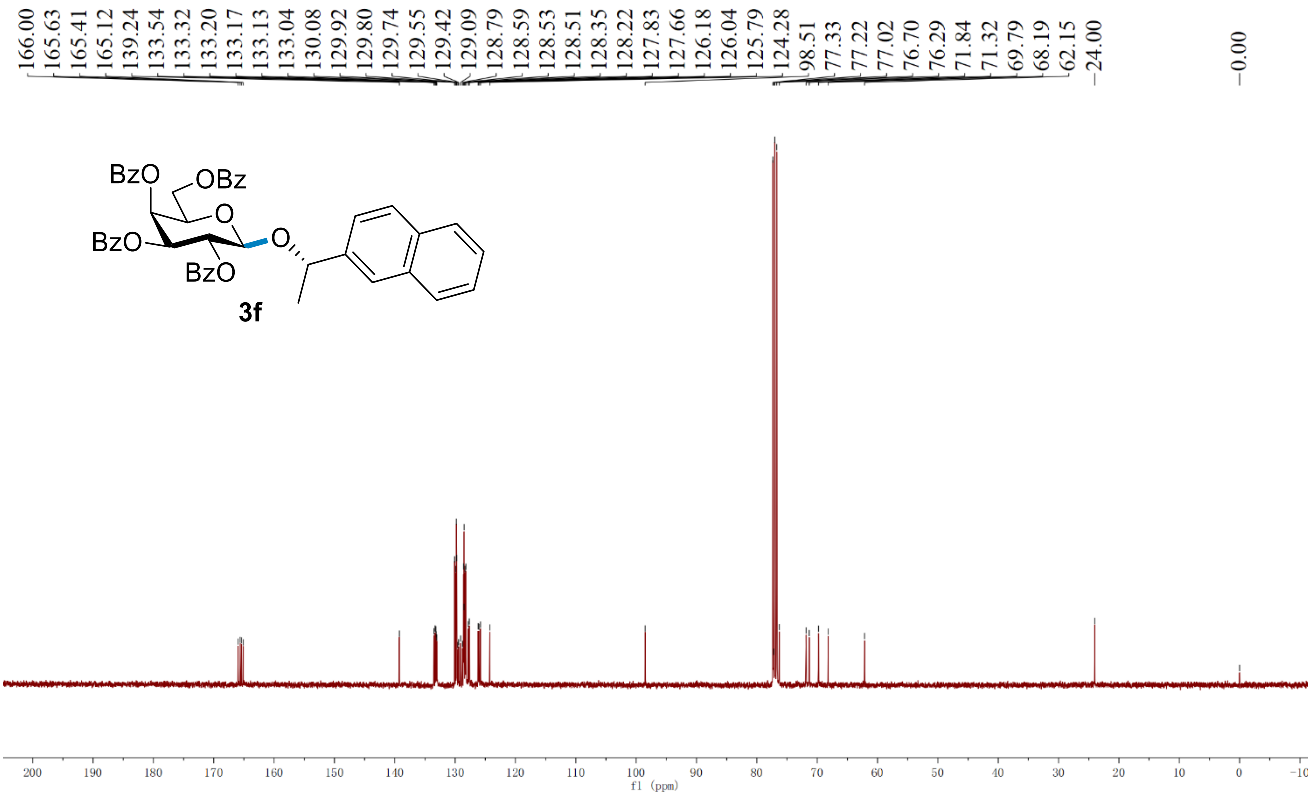

^{1}H NMR Spectrum of **3g** (400 MHz, $CDCl_3$)

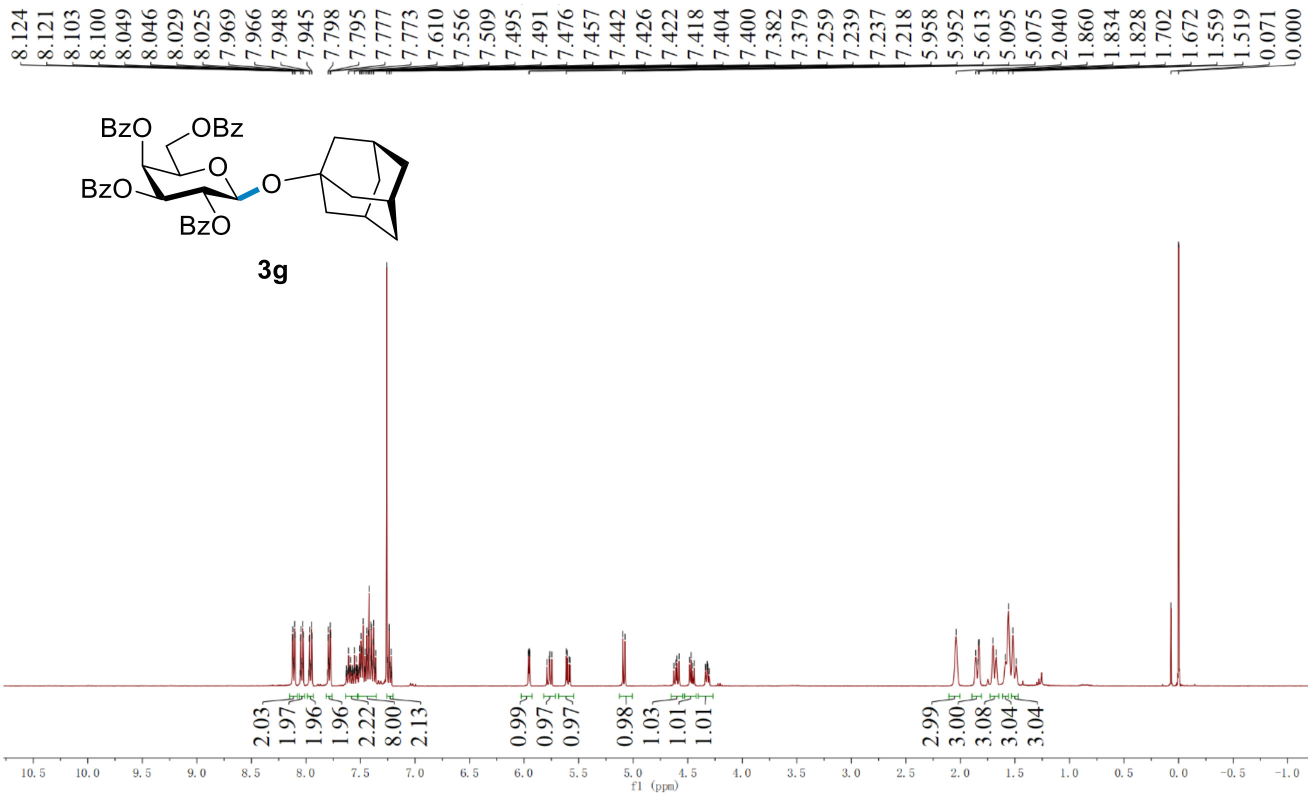

^{1}H NMR Spectrum of **3h** (400 MHz, $CDCl_3$)

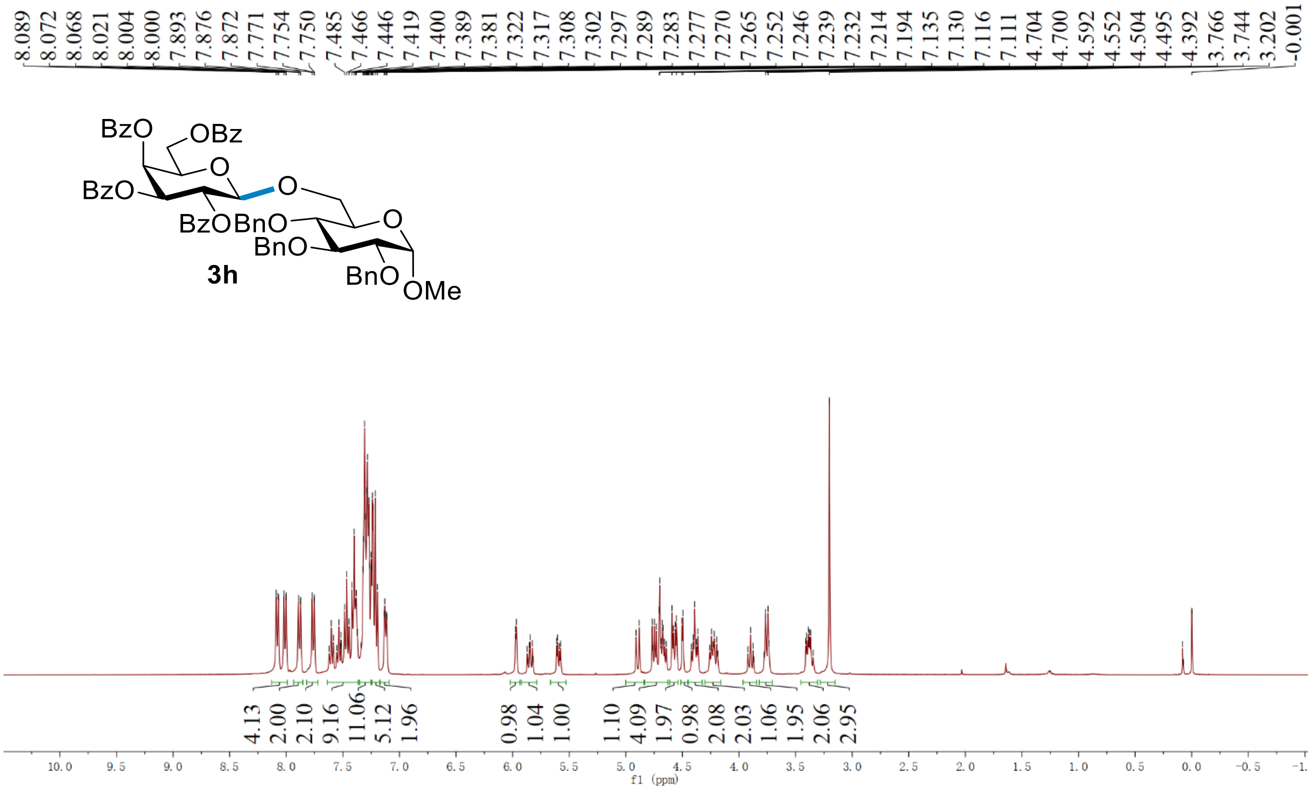

^{1}H NMR Spectrum of **3i** (400 MHz, $CDCl_3$)

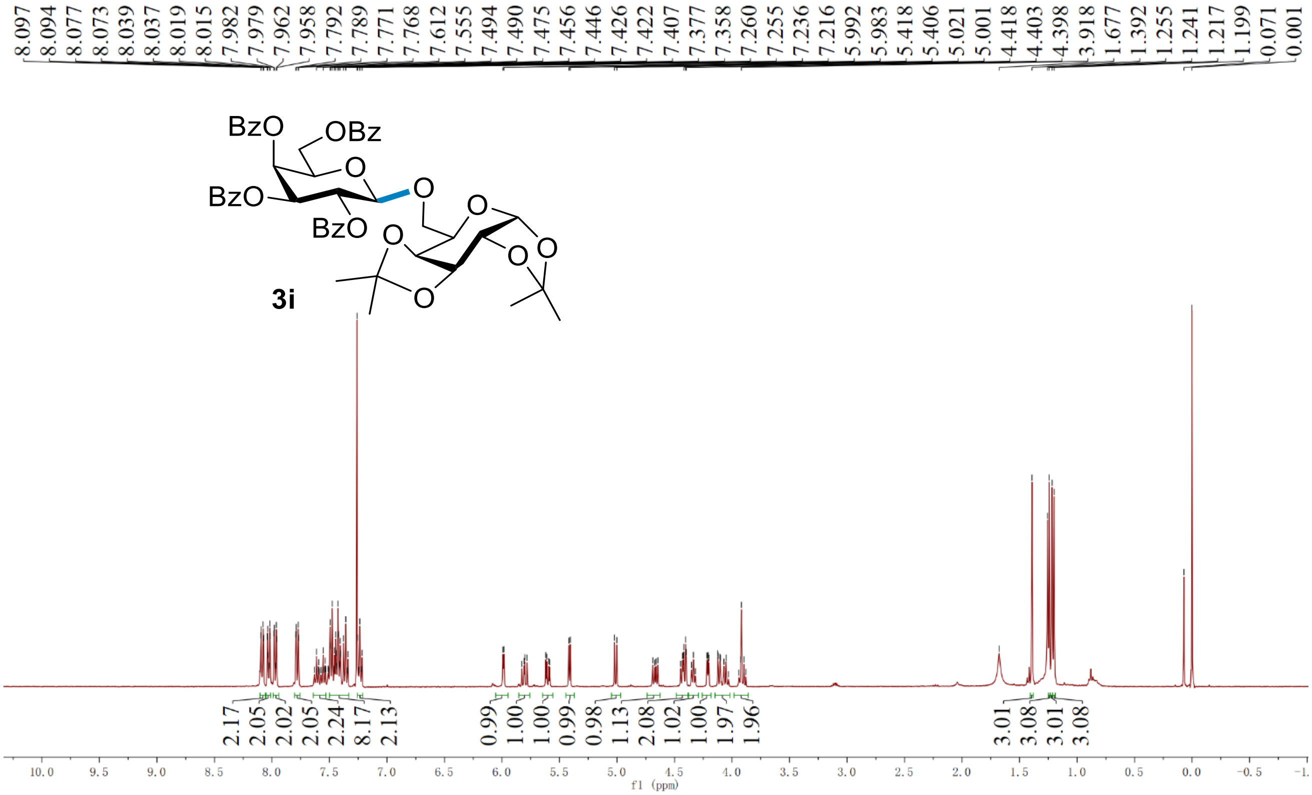

^{1}H NMR Spectrum of **3j** (400 MHz, $CDCl_3$)

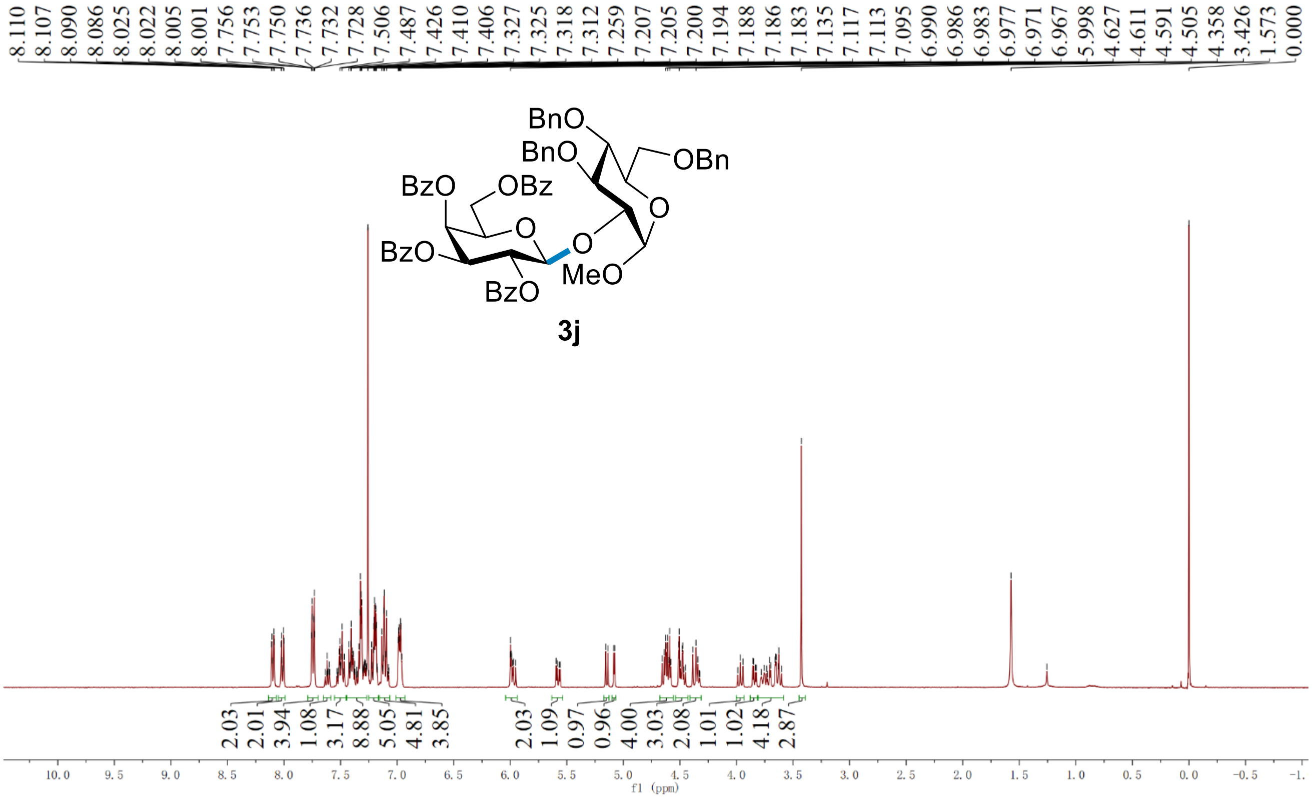

^{1}H NMR Spectrum of **3k** (400 MHz, $CDCl_3$)

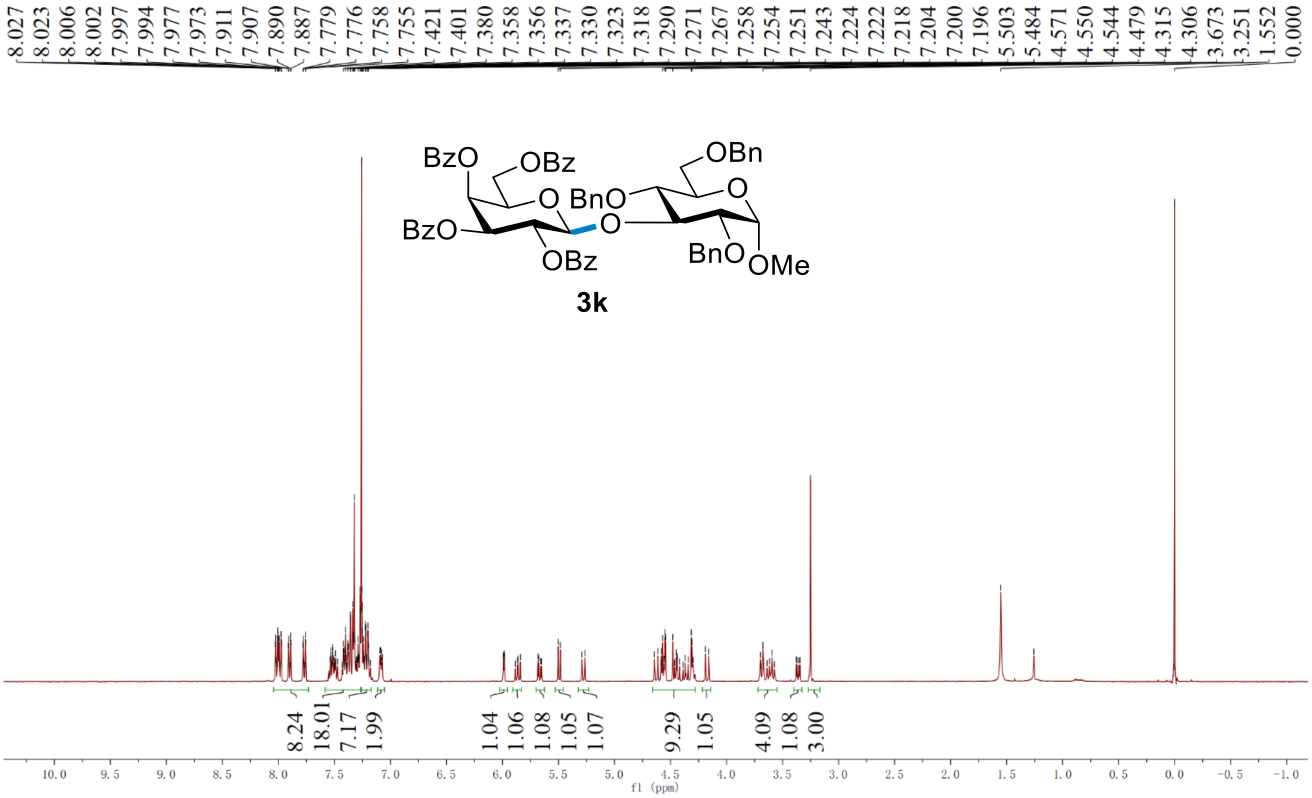

^{1}H NMR Spectrum of **3l** (400 MHz, $CDCl_3$)

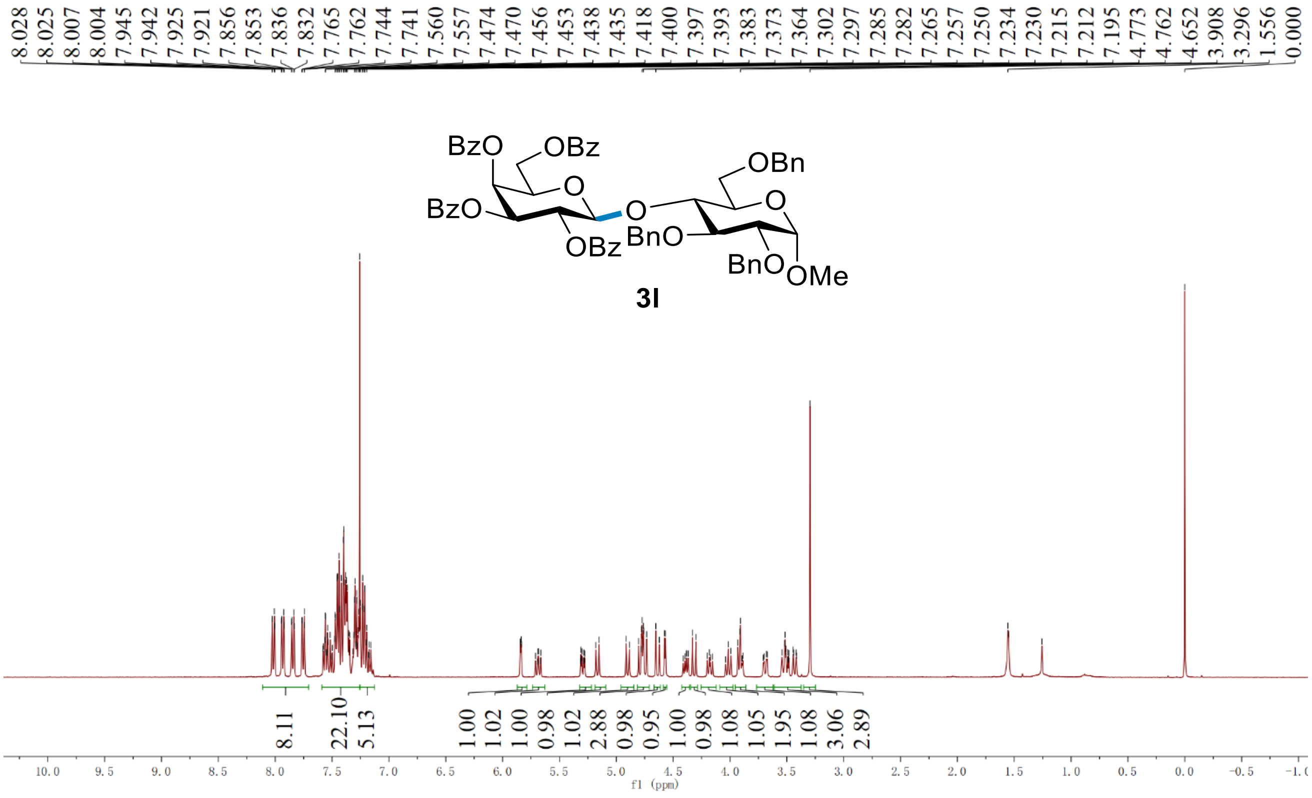

^{1}H NMR Spectrum of **3m** (400 MHz, $CDCl_3$)

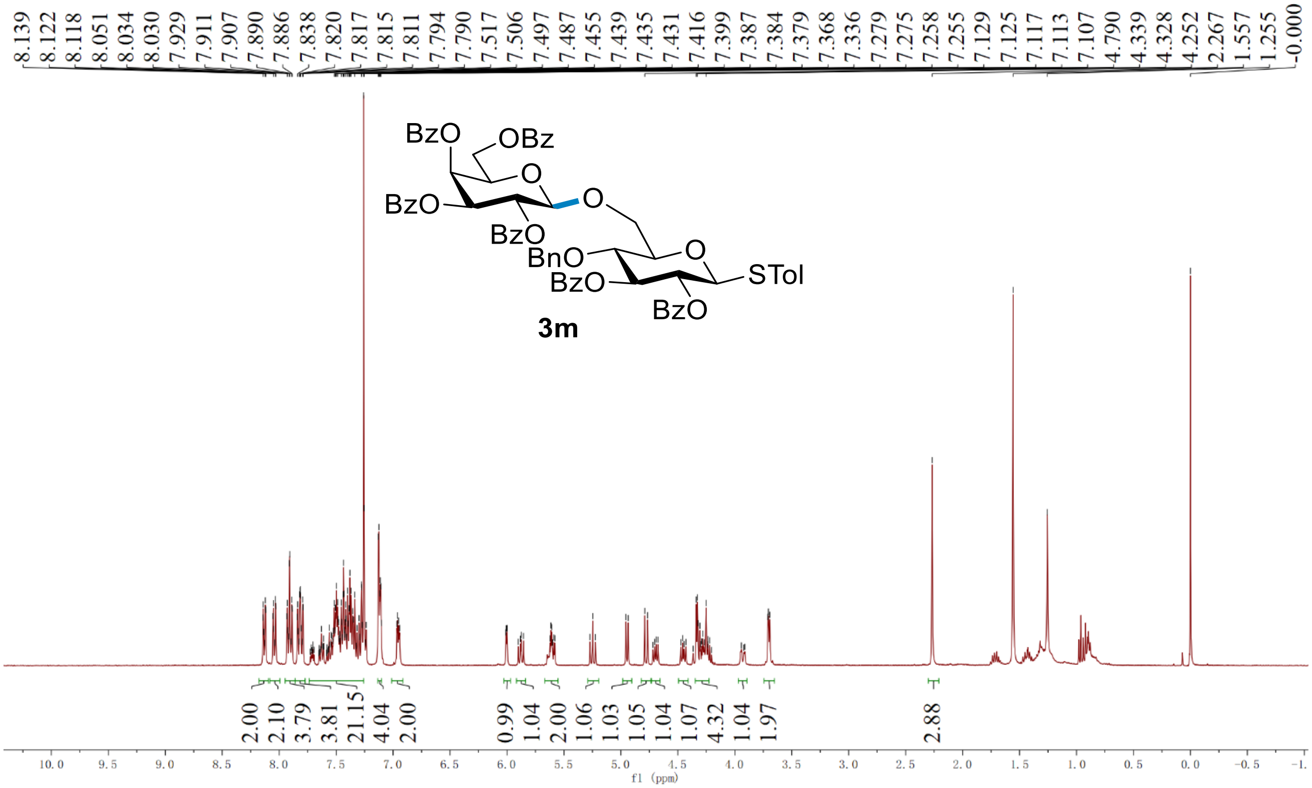

^{13}C NMR Spectrum of **3m** (101 MHz, $CDCl_3$)

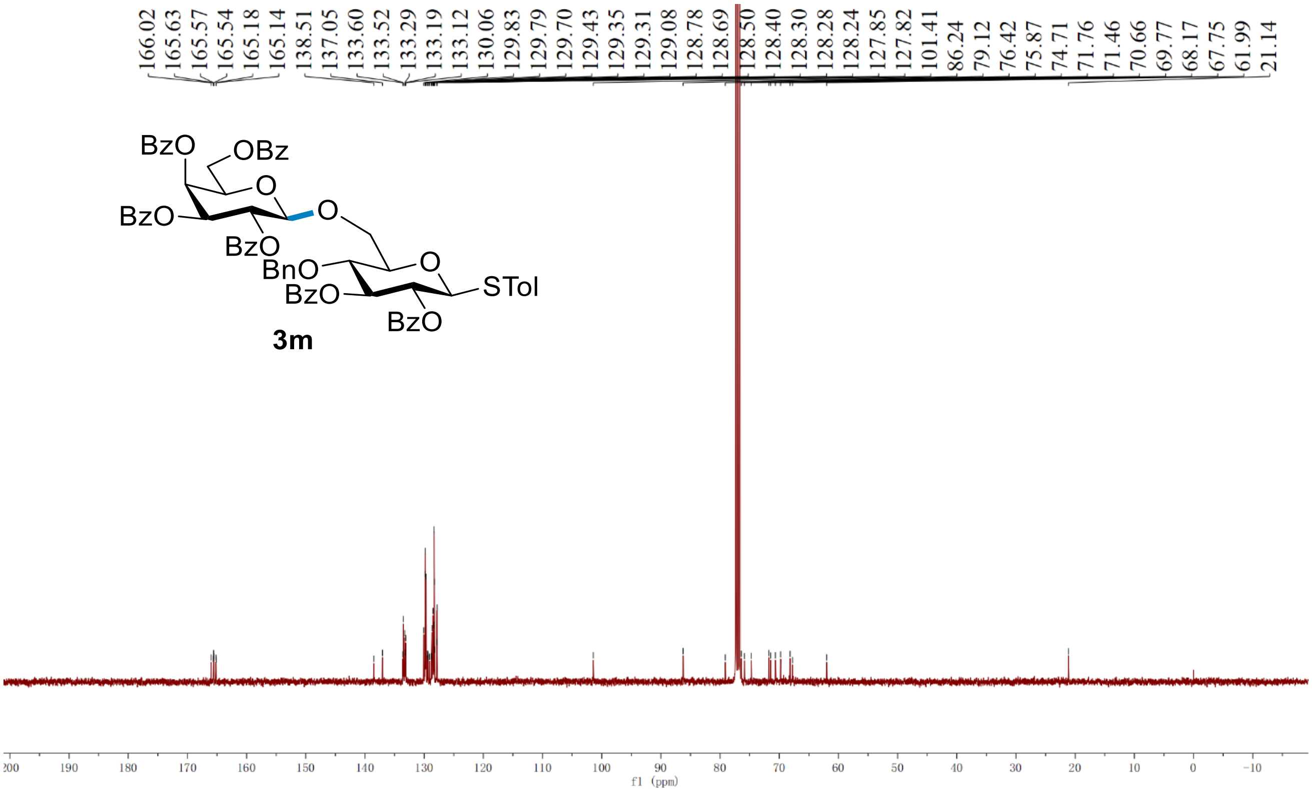

^{1}H NMR Spectrum of **3n** (400 MHz, $CDCl_3$)

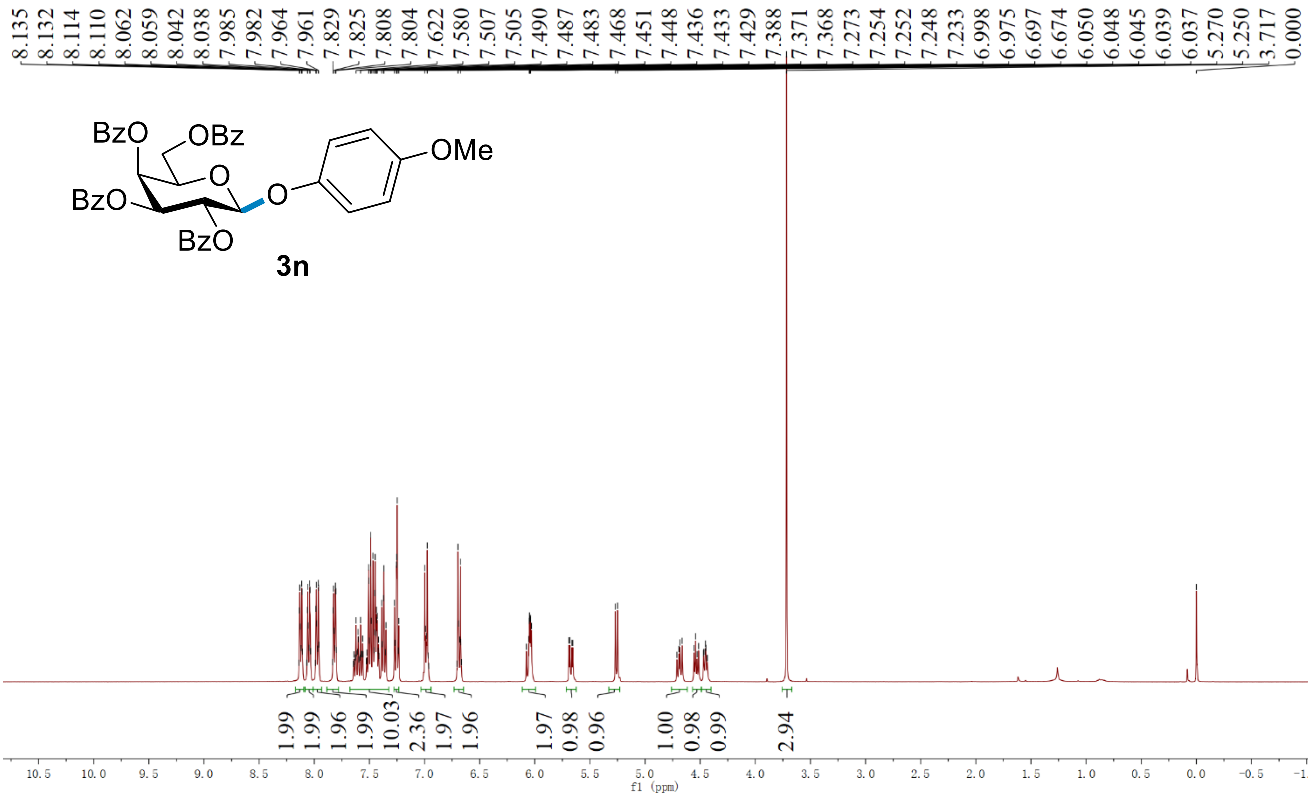

^{1}H NMR Spectrum of **3o** (400 MHz, $CDCl_3$)

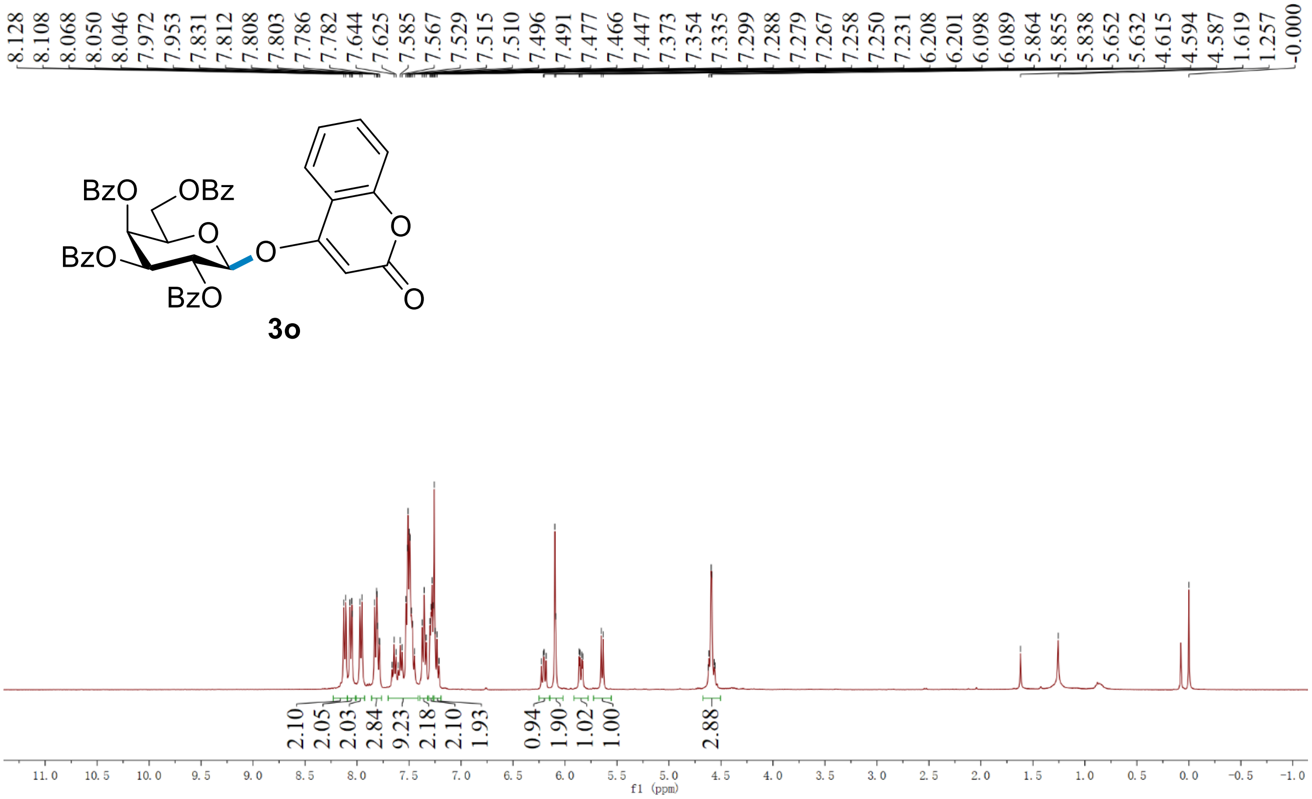

^{13}C NMR Spectrum of **3o** (101 MHz, $CDCl_3$)

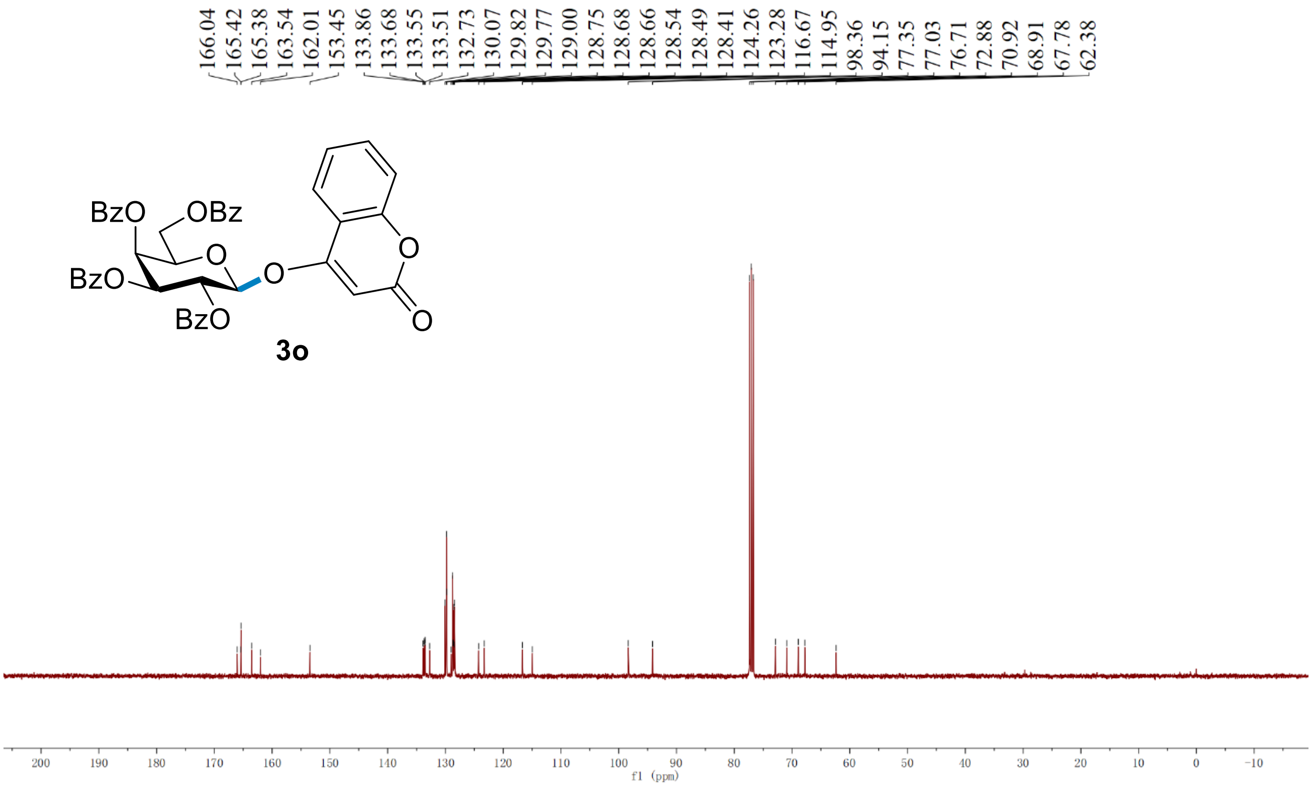

^{1}H NMR Spectrum of **3p** (400 MHz, $CDCl_3$)

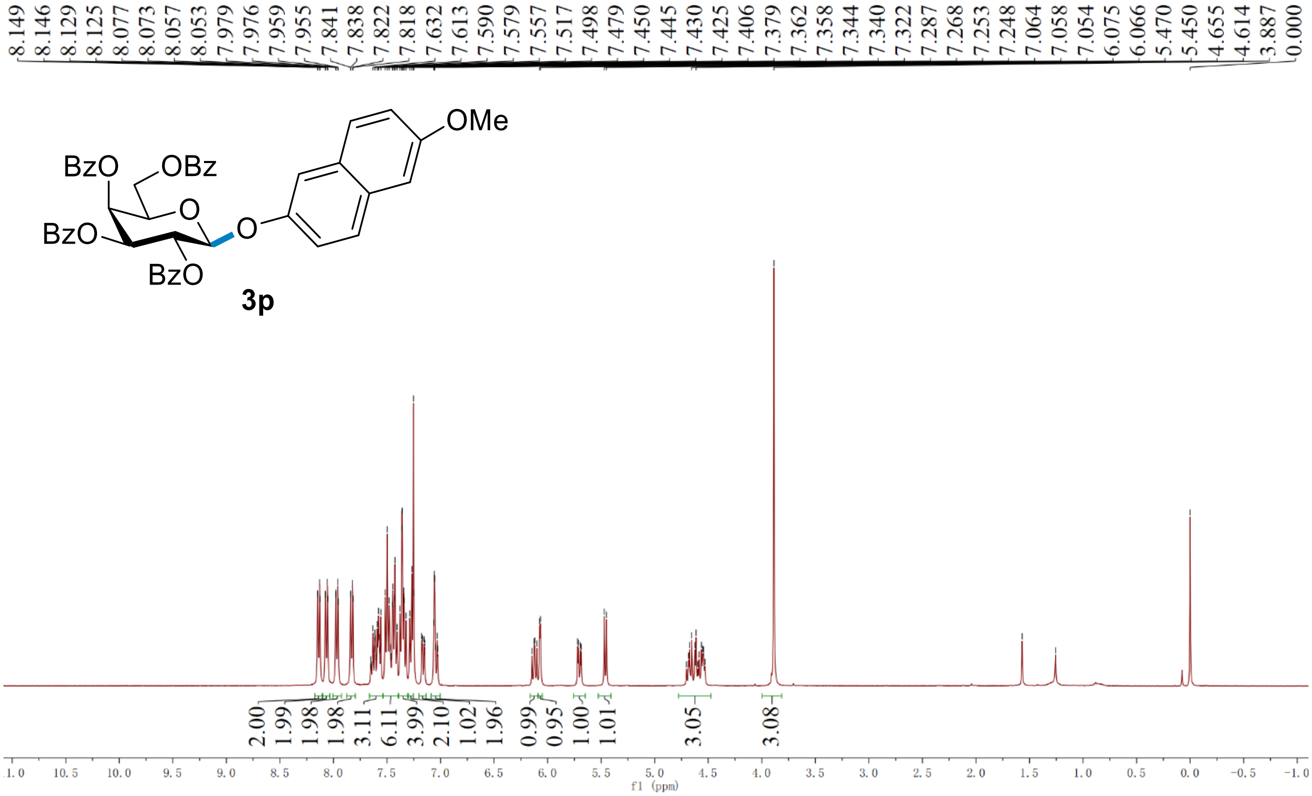

^{13}C NMR Spectrum of **3p** (101 MHz, $CDCl_3$)

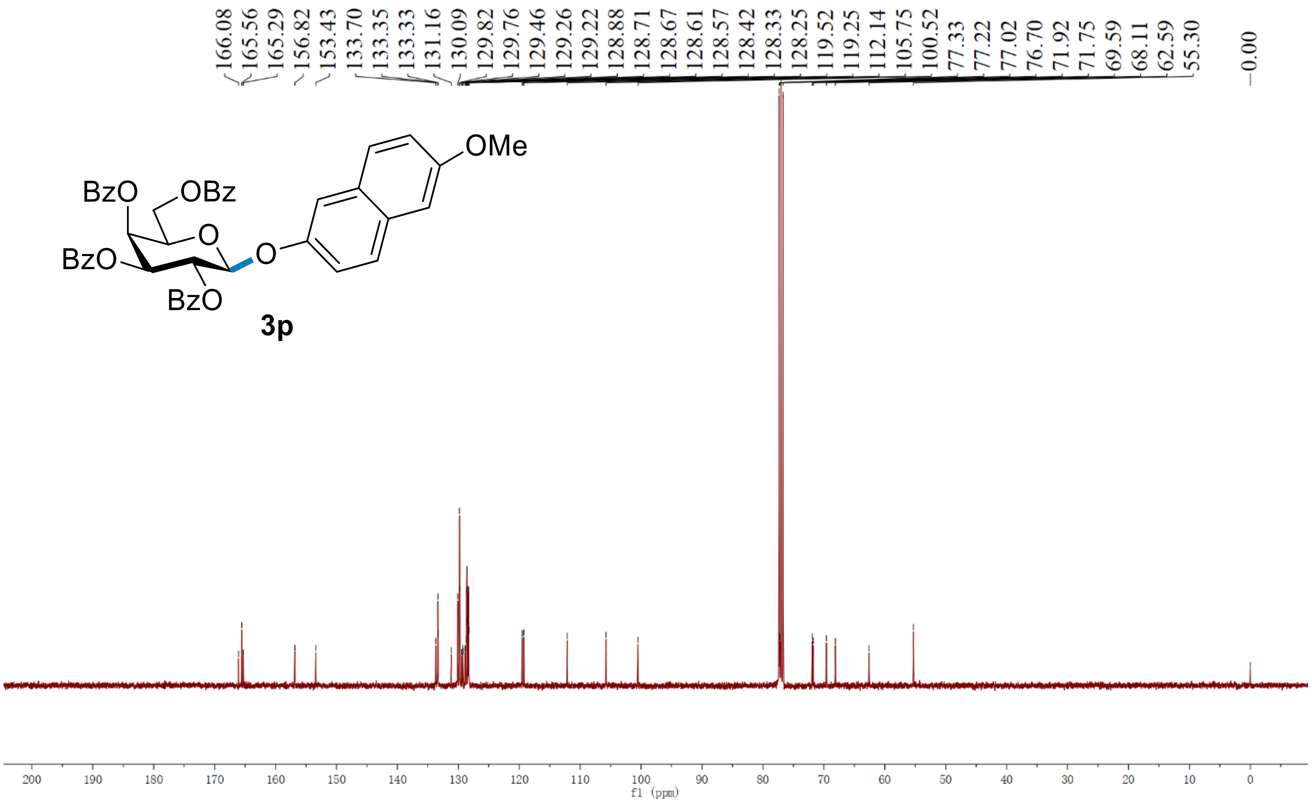

^{1}H NMR Spectrum of **3q** (400 MHz, $CDCl_3$)

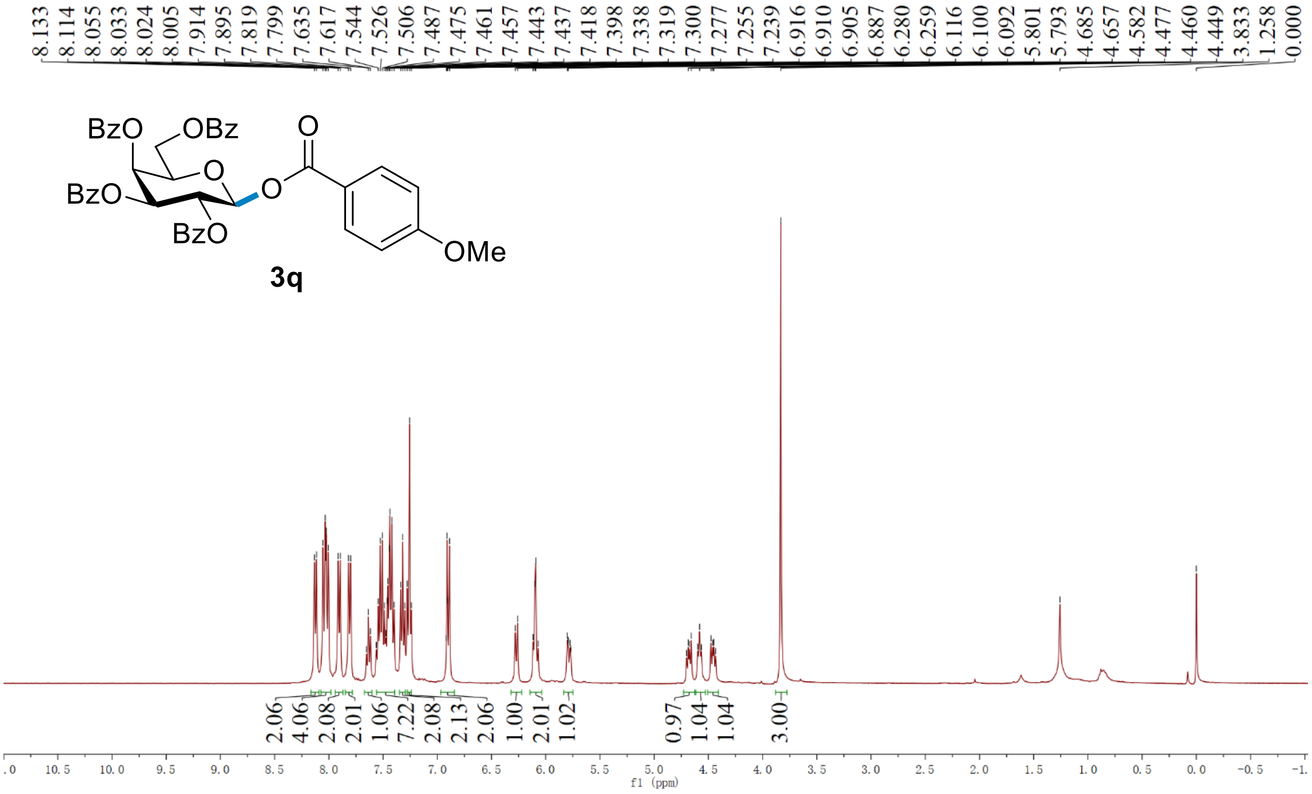

^{13}C NMR Spectrum of **3q** (101 MHz, $CDCl_3$)

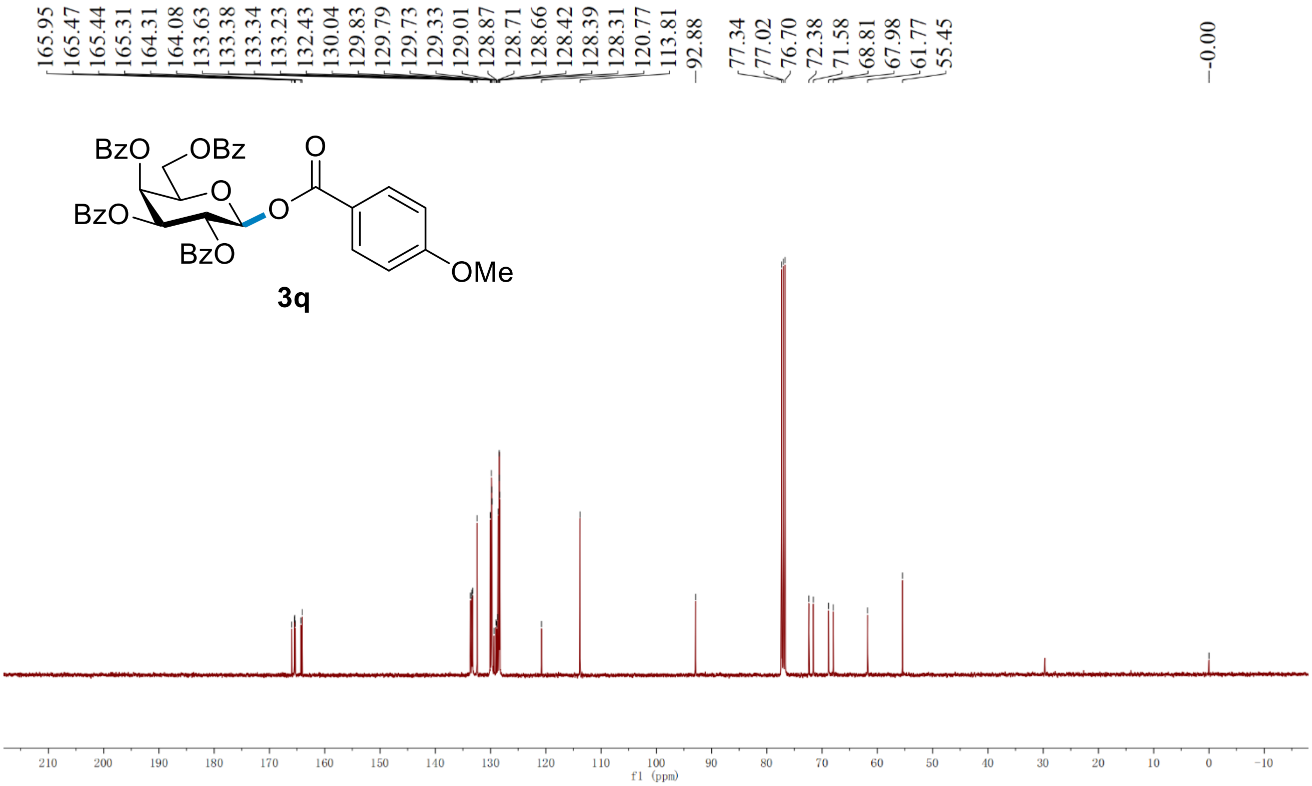

^{1}H NMR Spectrum of **3r** (400 MHz, $CDCl_3$)

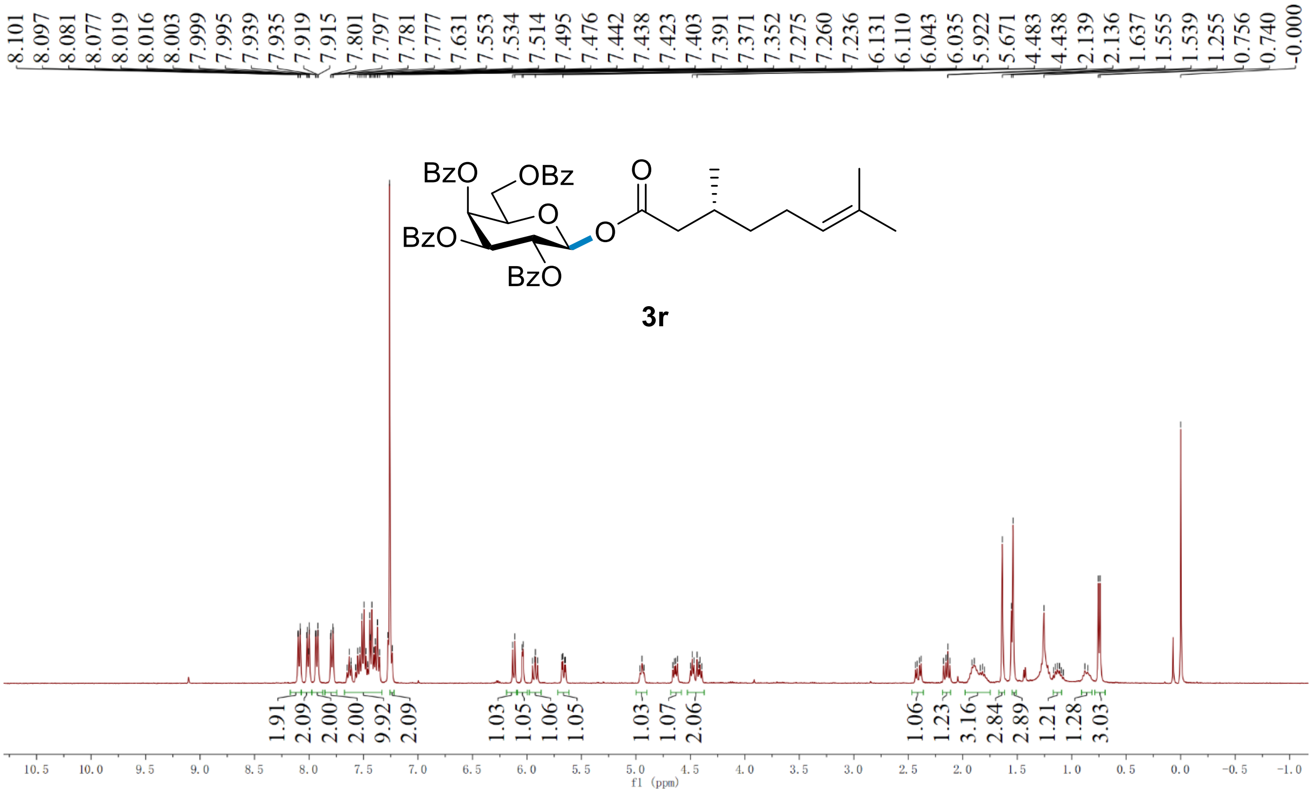

^{13}C NMR Spectrum of **3r** (101 MHz, $CDCl_3$)

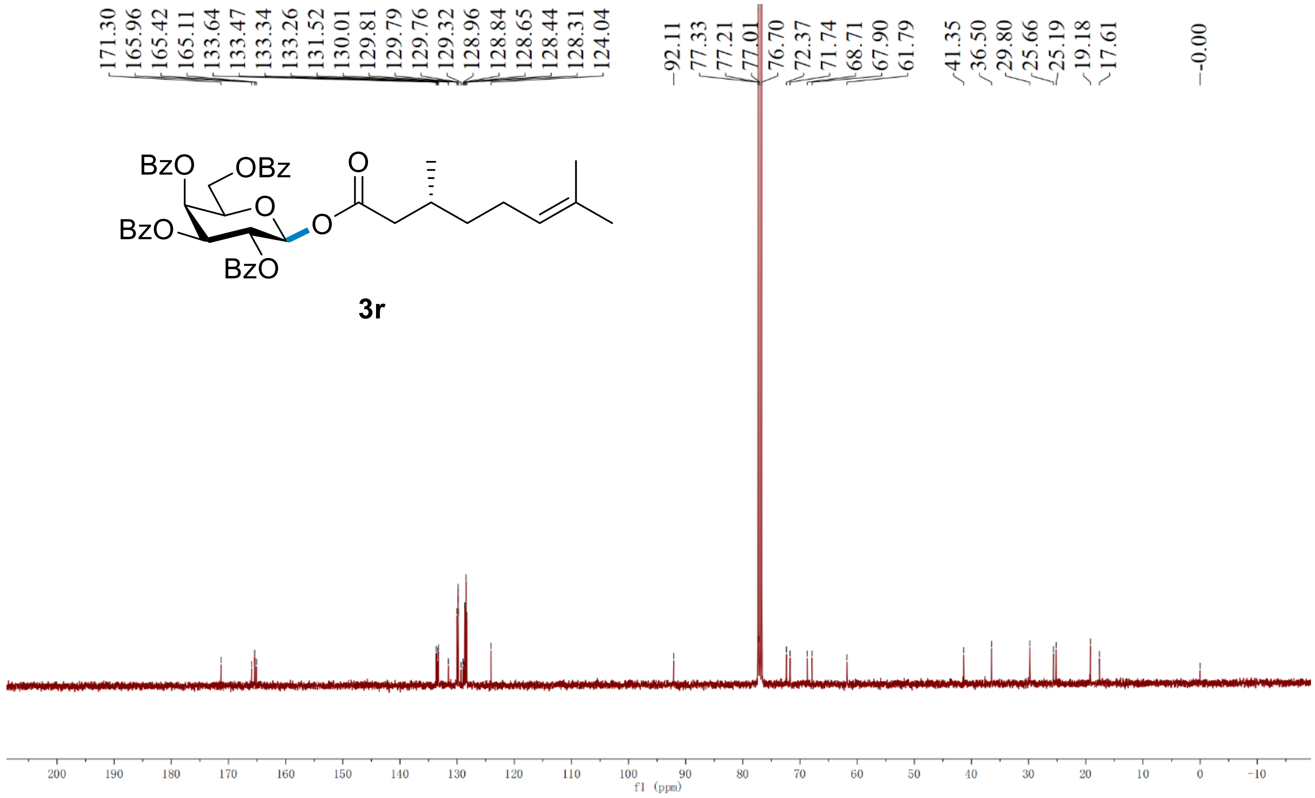

^{1}H NMR Spectrum of **4a** (400 MHz, $CDCl_3$)

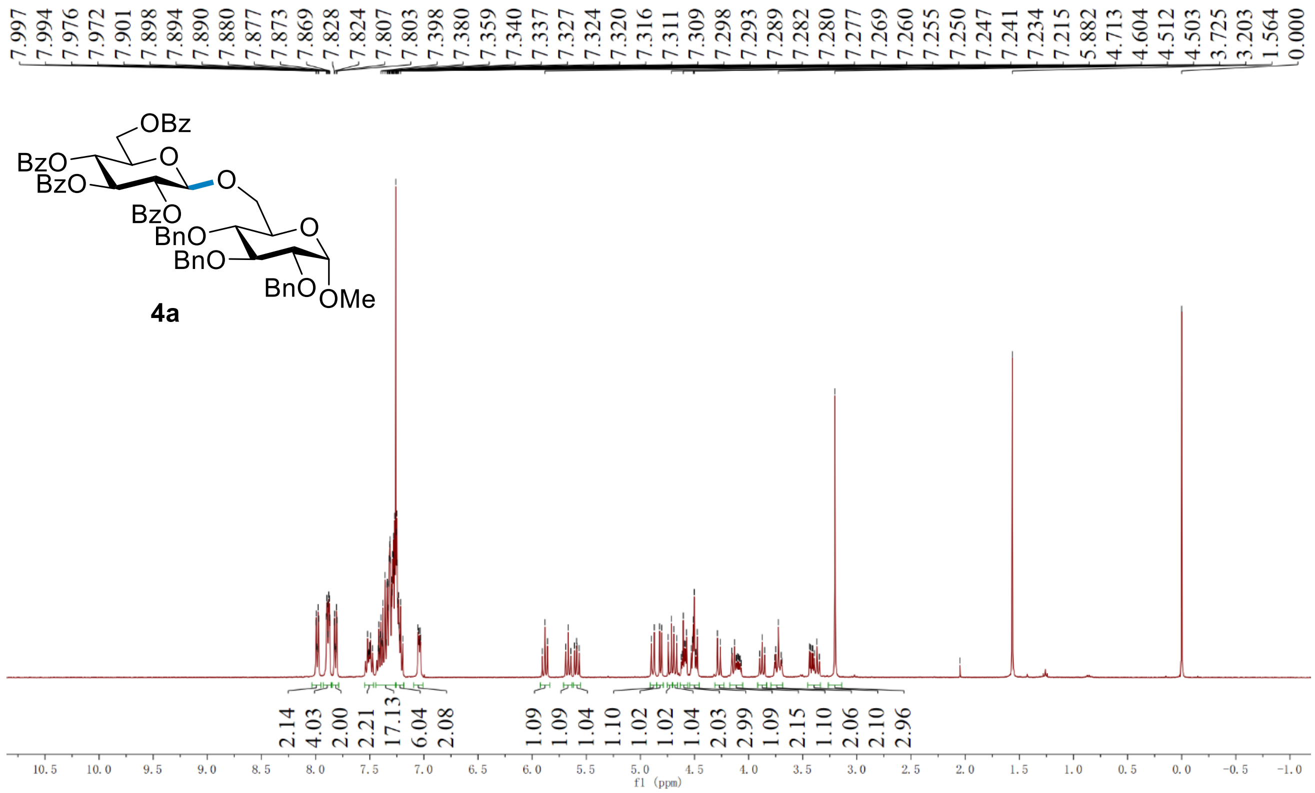

^{1}H NMR Spectrum of **4b** (400 MHz, $CDCl_3$)

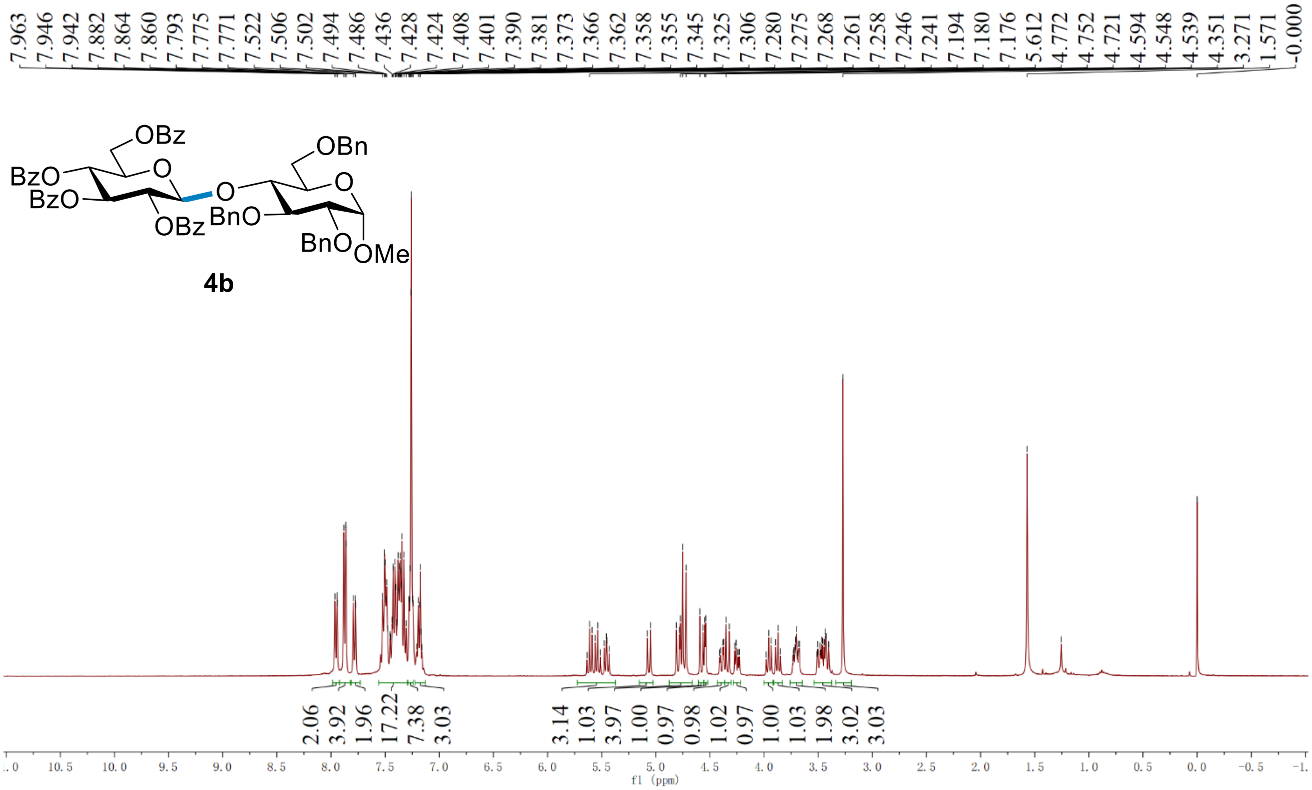

^{1}H NMR Spectrum of **4c** (400 MHz, $CDCl_3$)

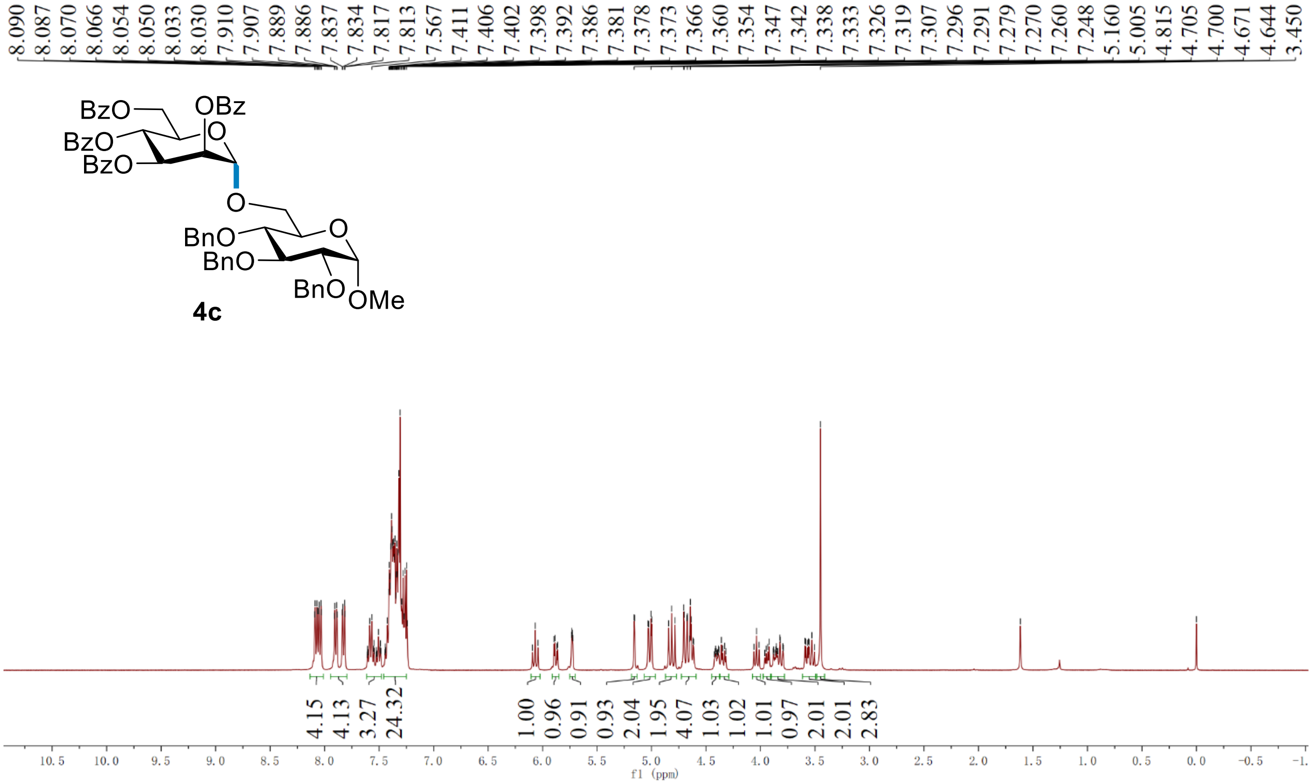

^{1}H NMR Spectrum of **4d** (400 MHz, $CDCl_3$)

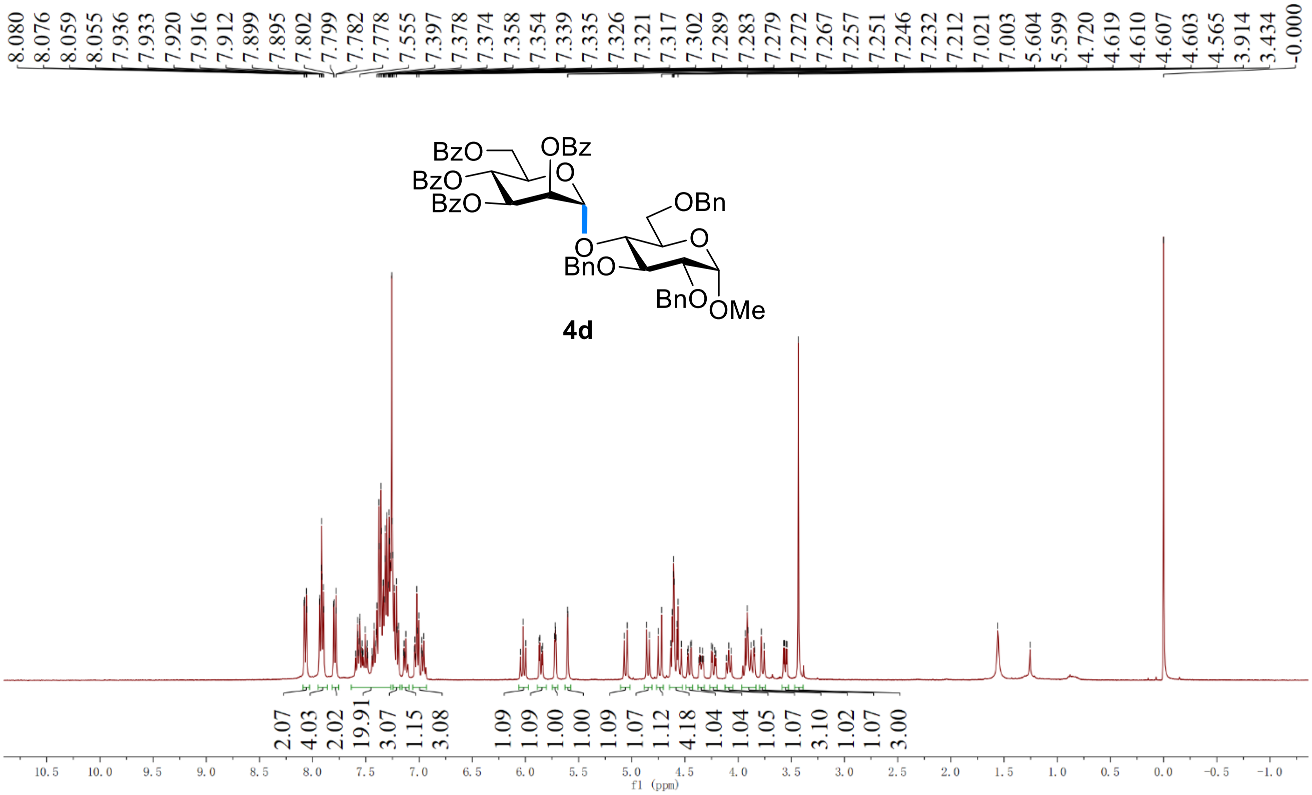

^{1}H NMR Spectrum of **4e** (400 MHz, $CDCl_3$)

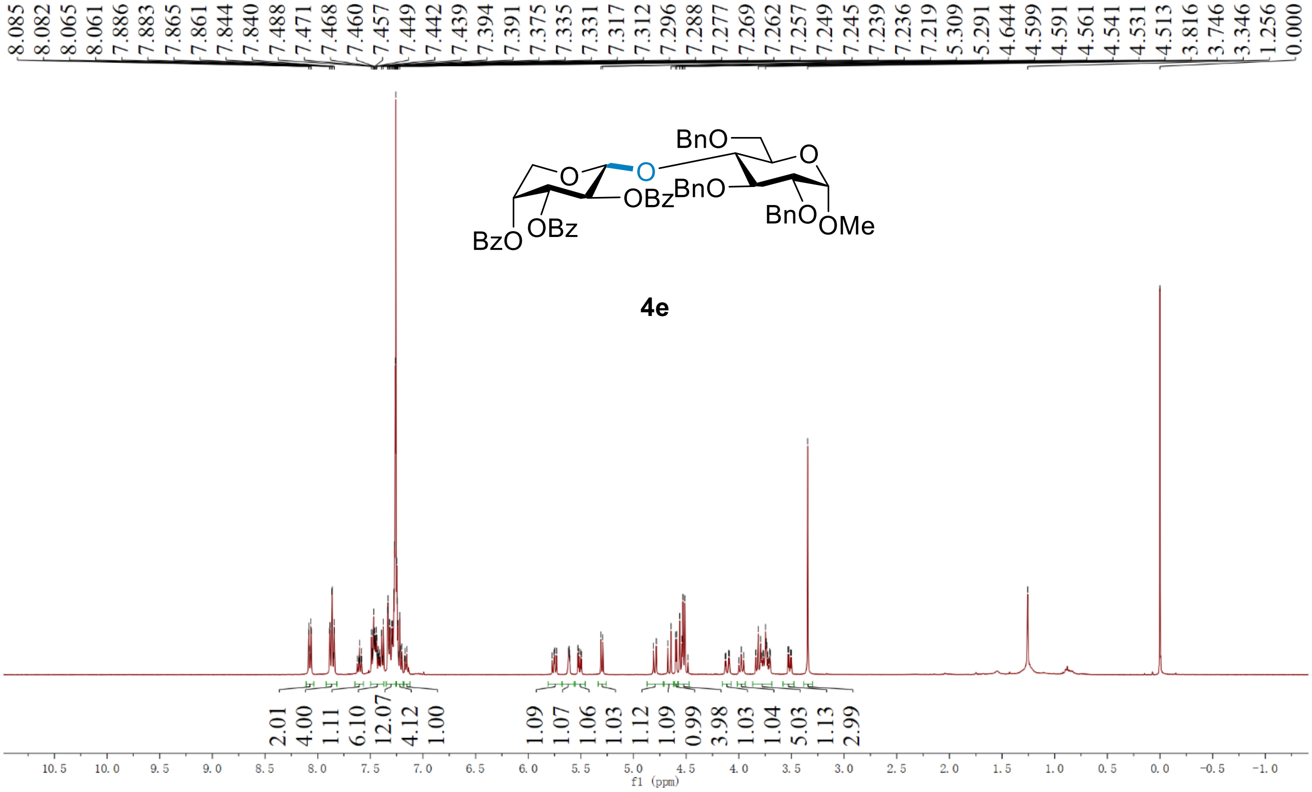

^{13}C NMR Spectrum of **4e** (101 MHz, $CDCl_3$)

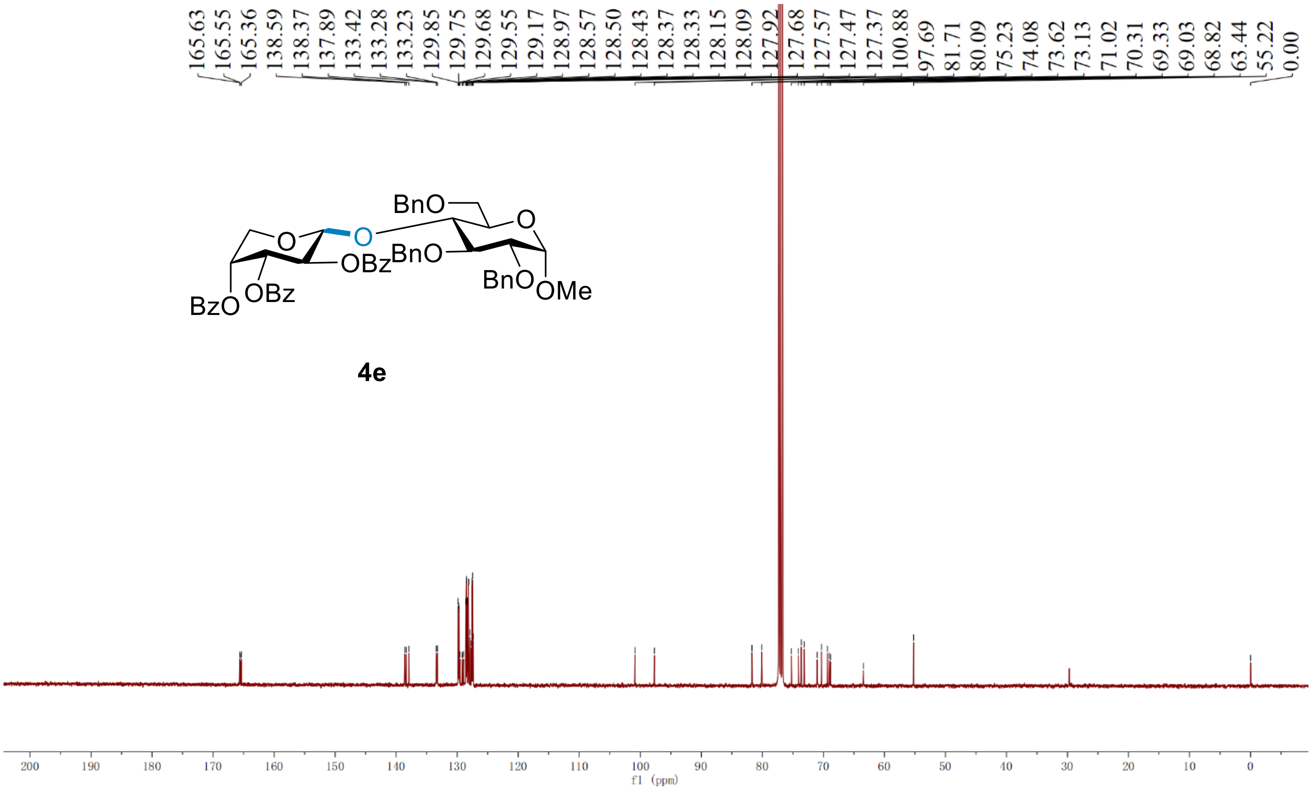

^{1}H NMR Spectrum of **4f** (400 MHz, $CDCl_3$)

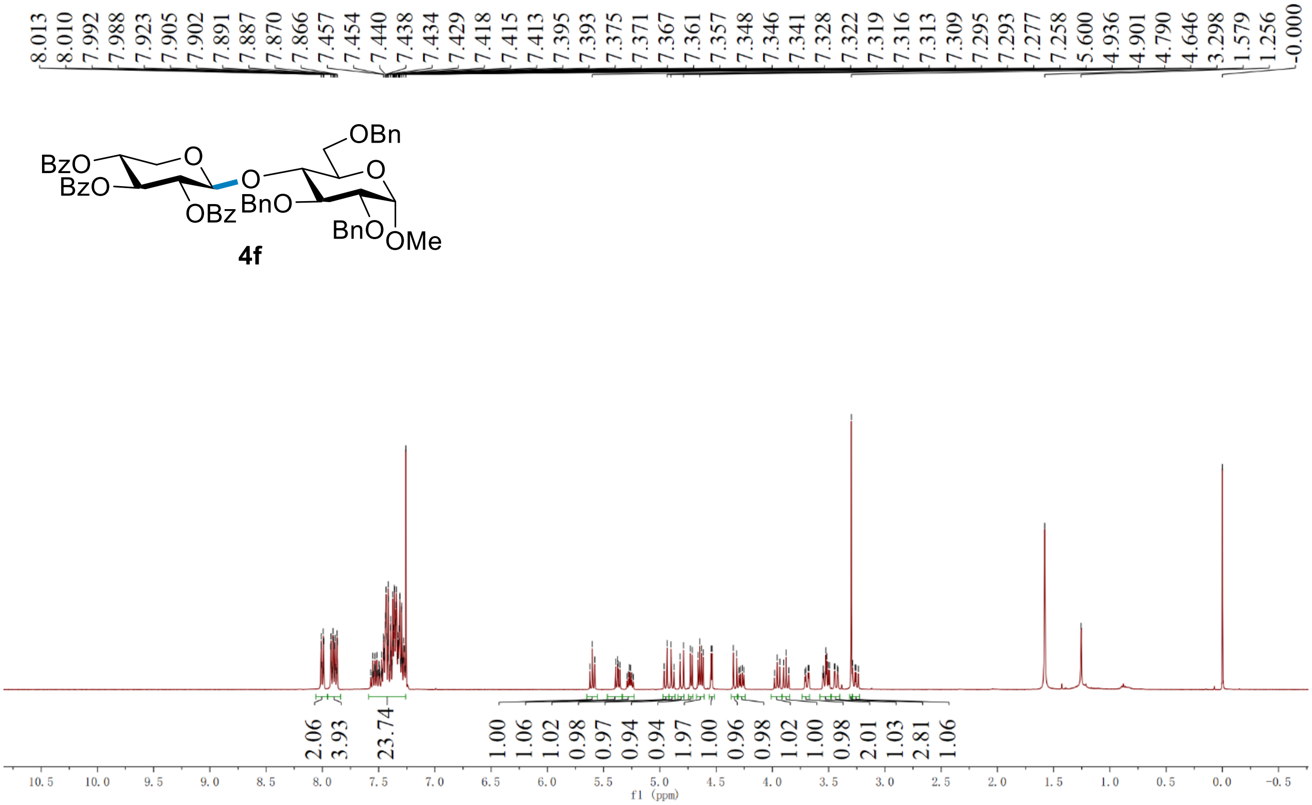

^{13}C NMR Spectrum of **4f** (101 MHz, $CDCl_3$)

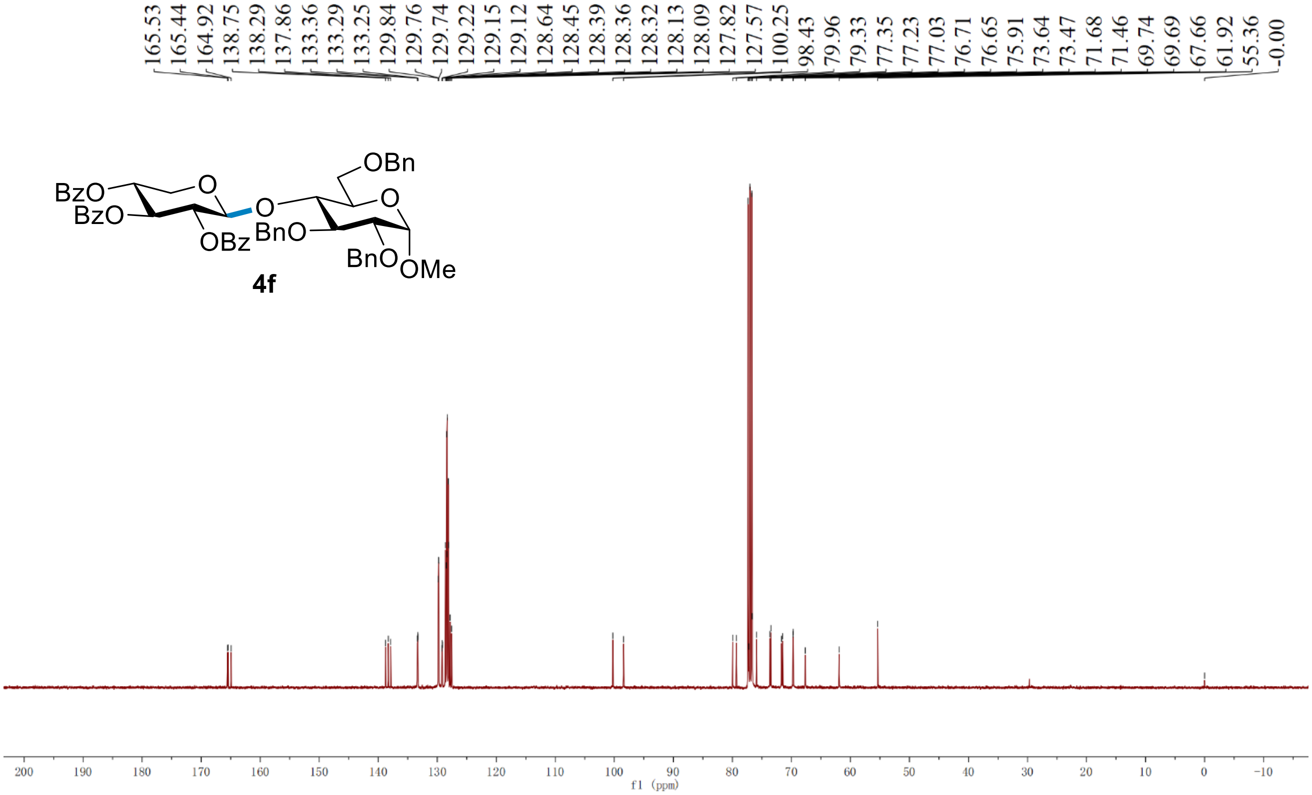

^{1}H NMR Spectrum of **4g** (400 MHz, $CDCl_3$)

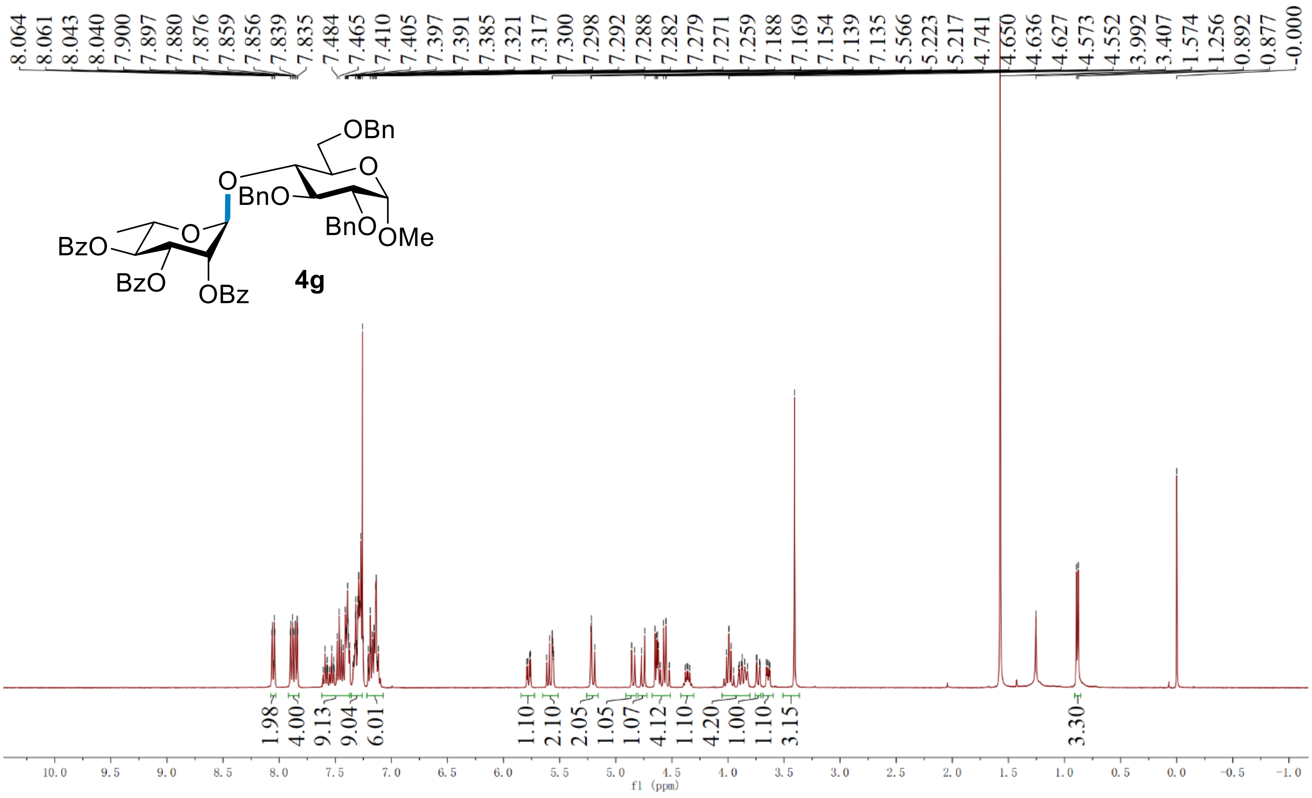

^{1}H NMR Spectrum of **4h** (400 MHz, $CDCl_3$)

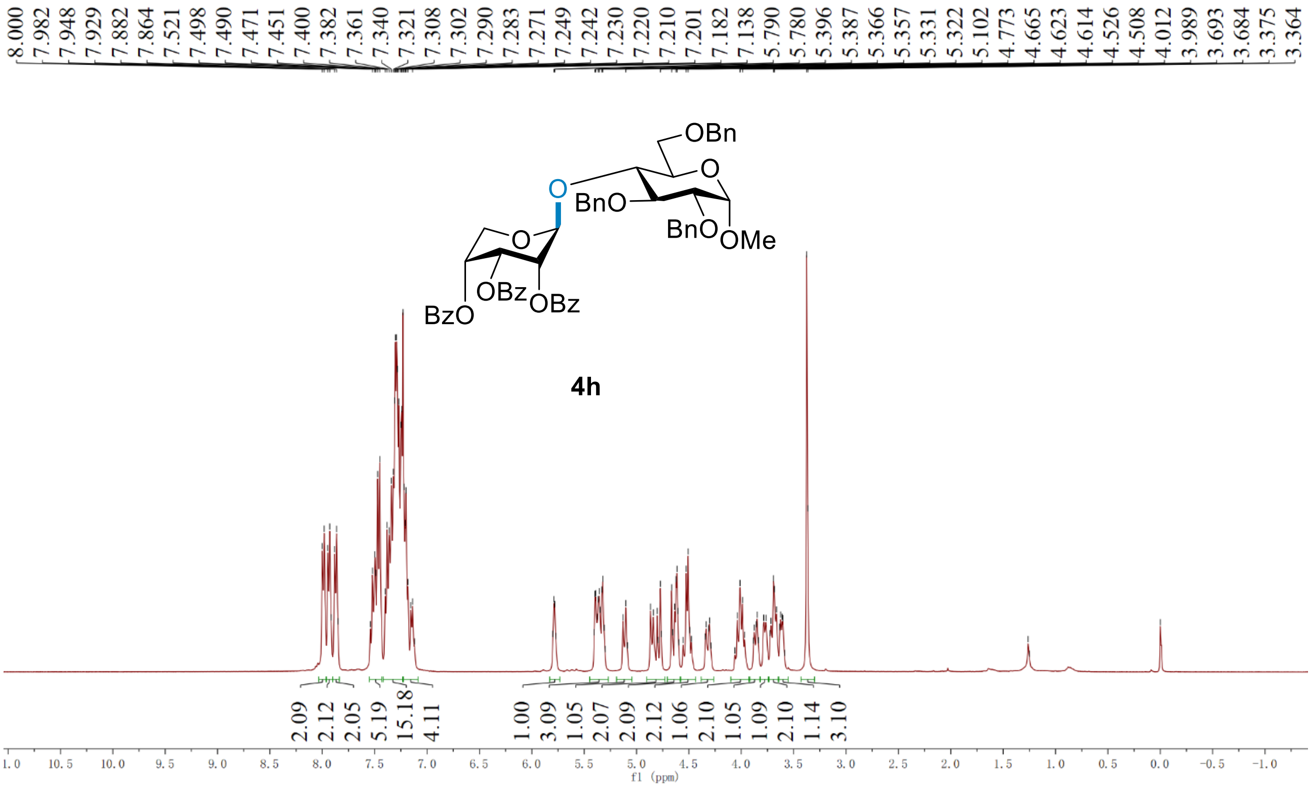

^{13}C NMR Spectrum of **4h** (101 MHz, $CDCl_3$)

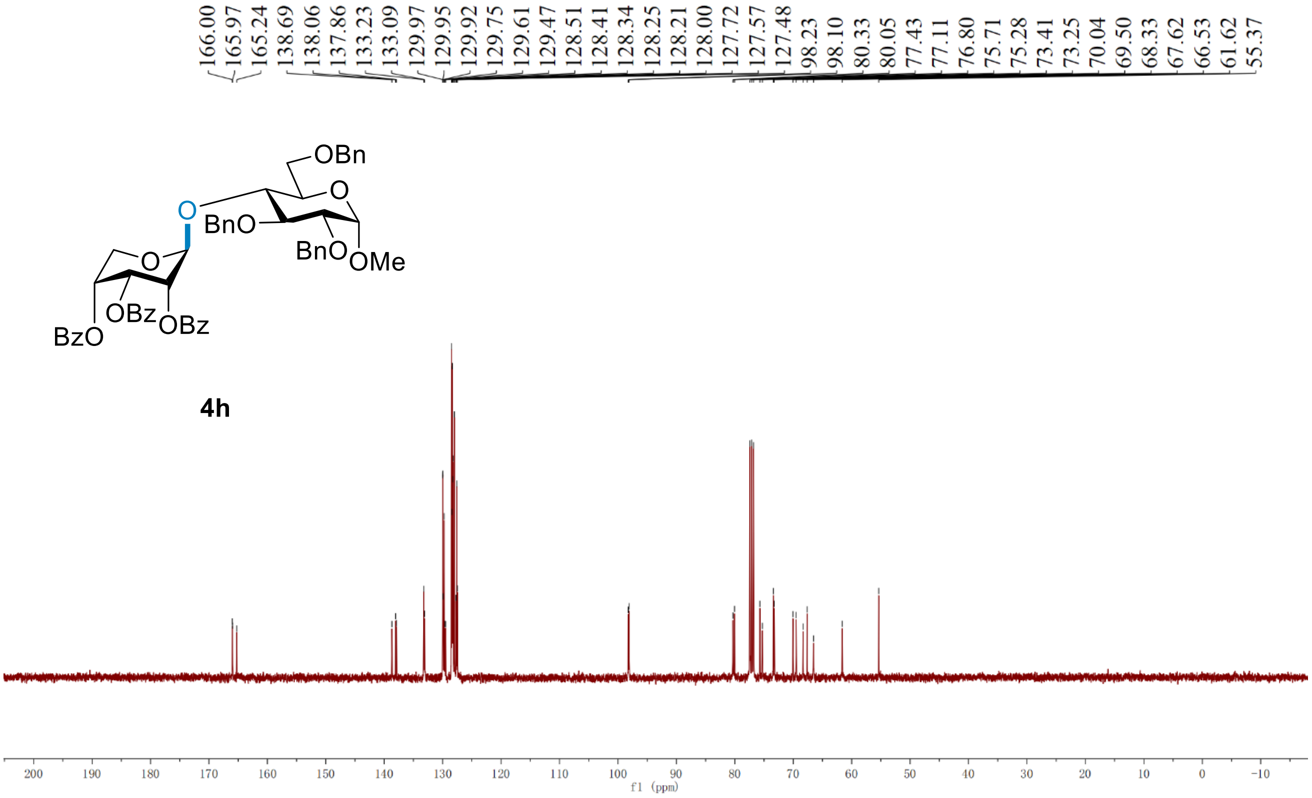

^{1}H NMR Spectrum of **4i** (400 MHz, $CDCl_3$)

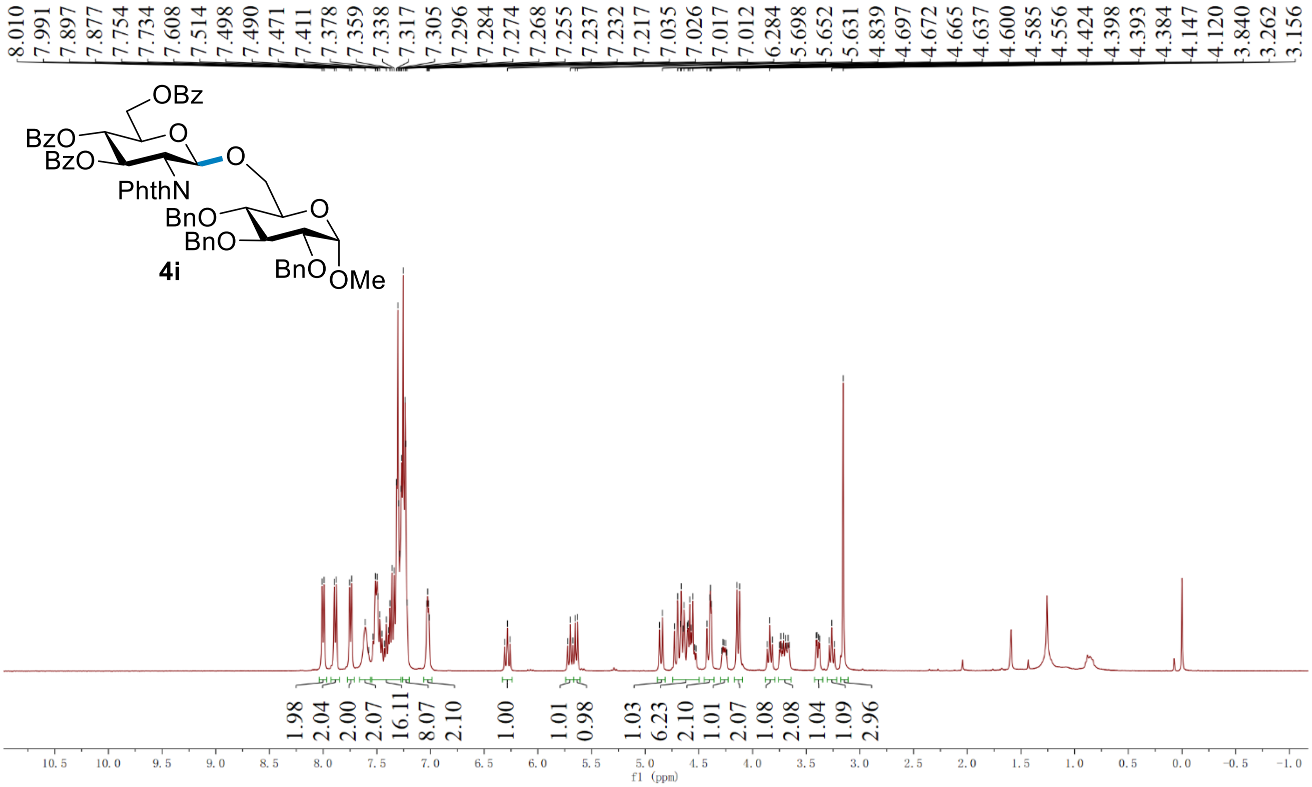

^{1}H NMR Spectrum of **4j** (400 MHz, $CDCl_3$)

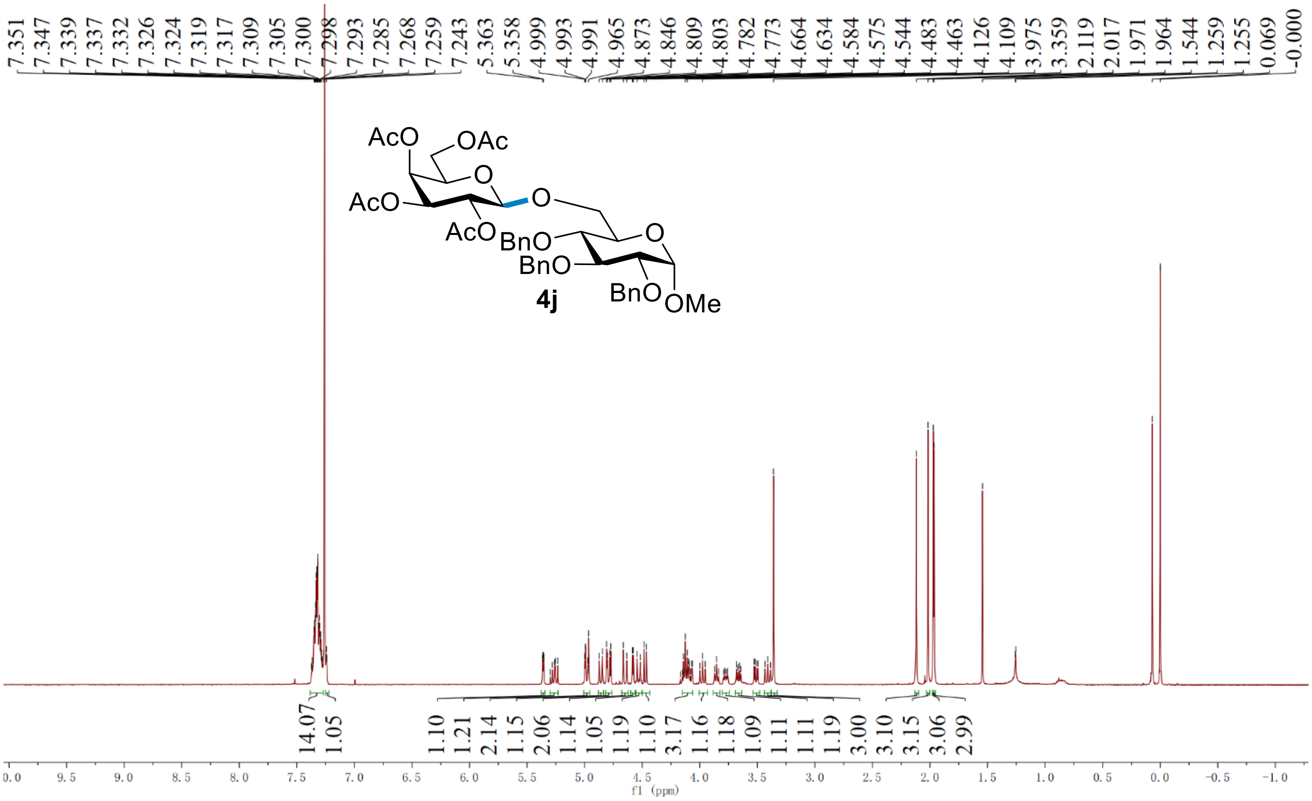

^{1}H NMR Spectrum of **4k** (400 MHz, $CDCl_3$)

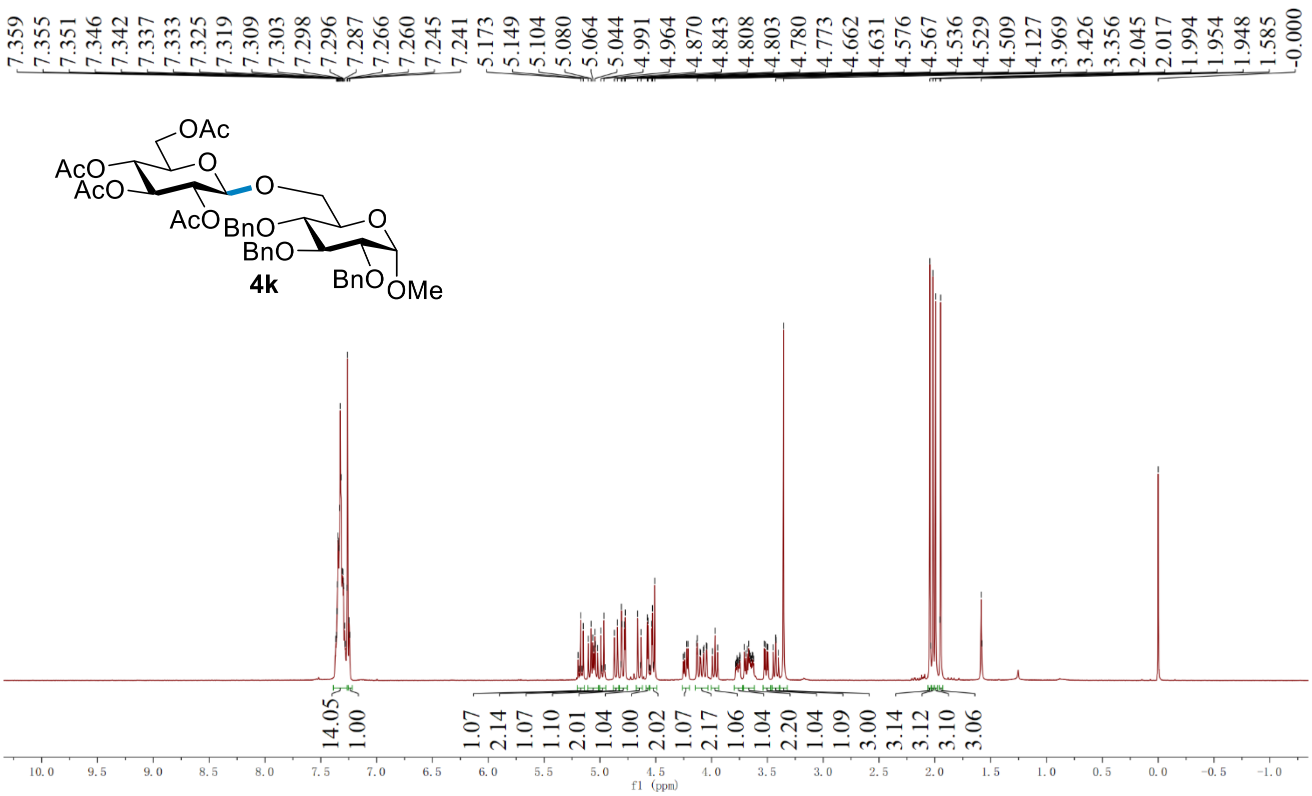

^{1}H NMR Spectrum of **4l** (400 MHz, $CDCl_3$)

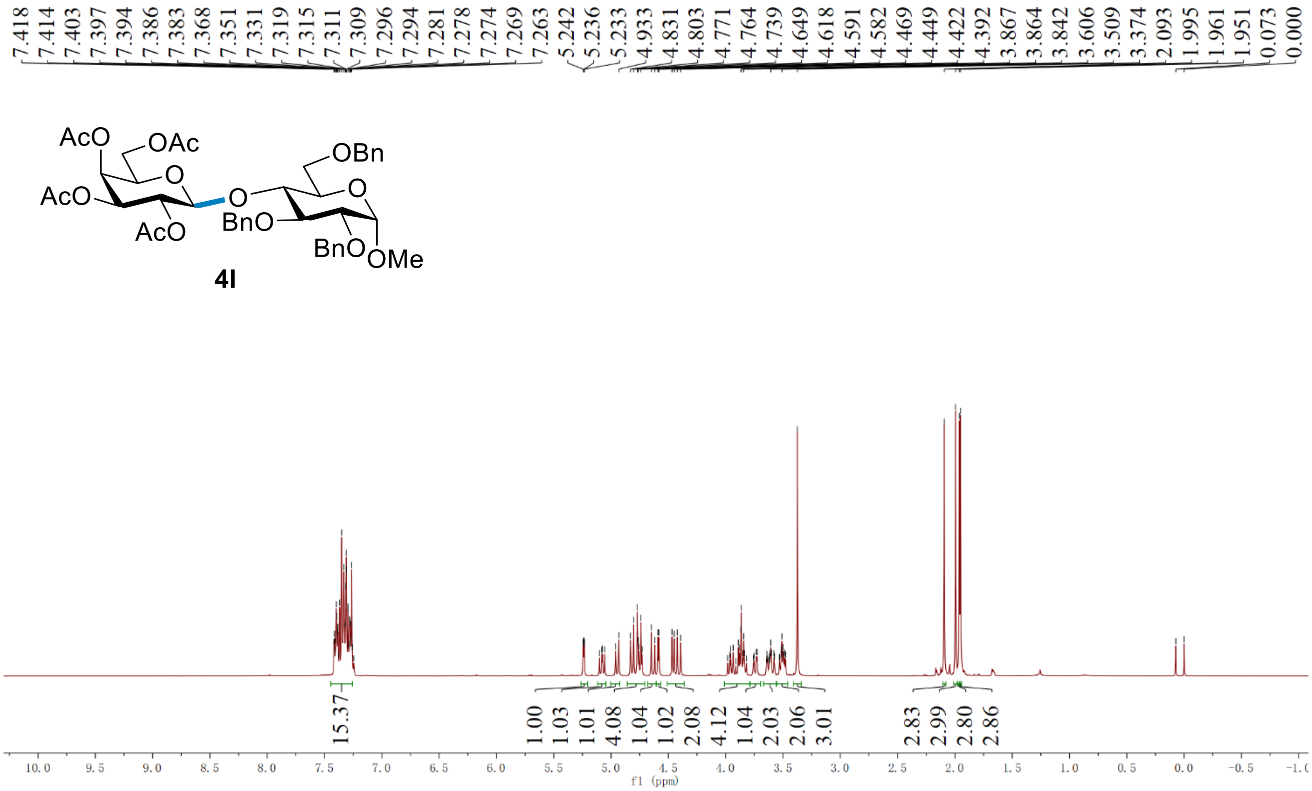

^{1}H NMR Spectrum of **4m** (400 MHz, $CDCl_3$)

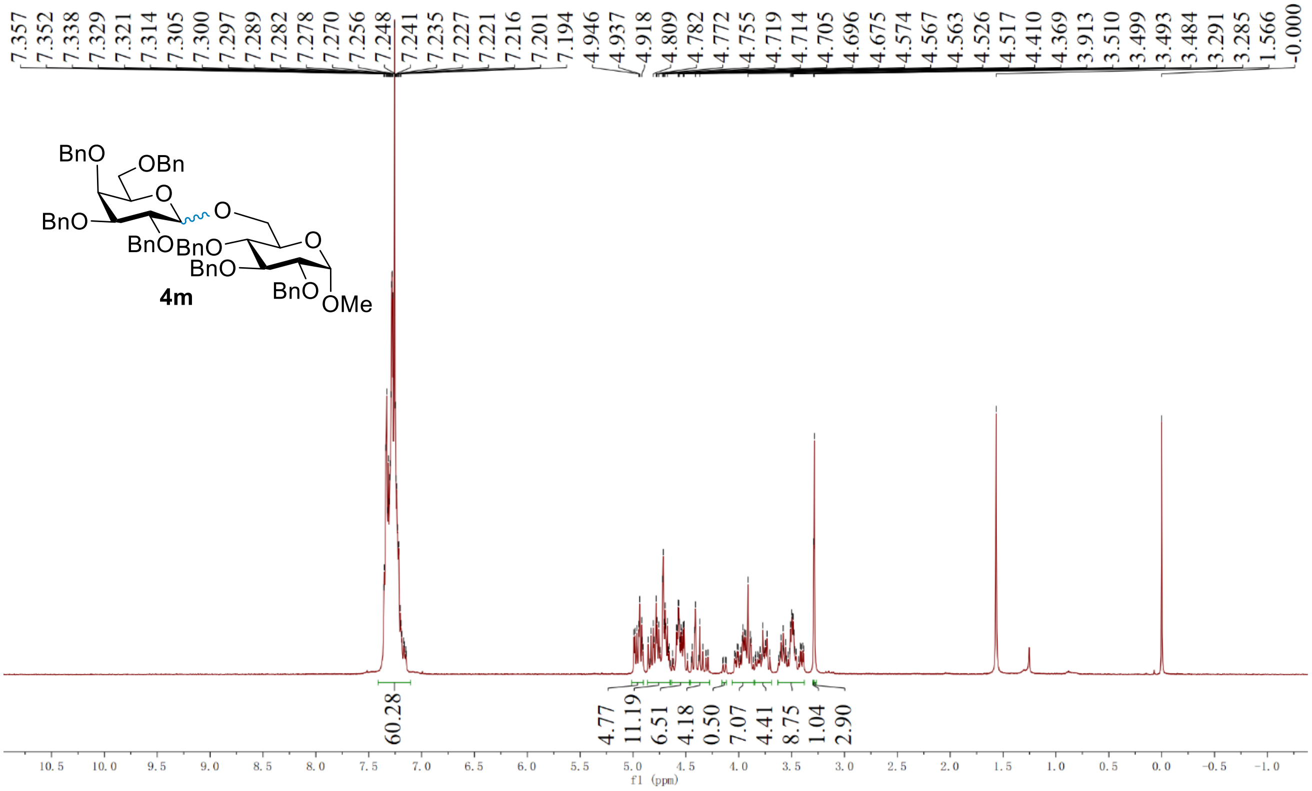

^{1}H NMR Spectrum of **4n** (400 MHz, $CDCl_3$)

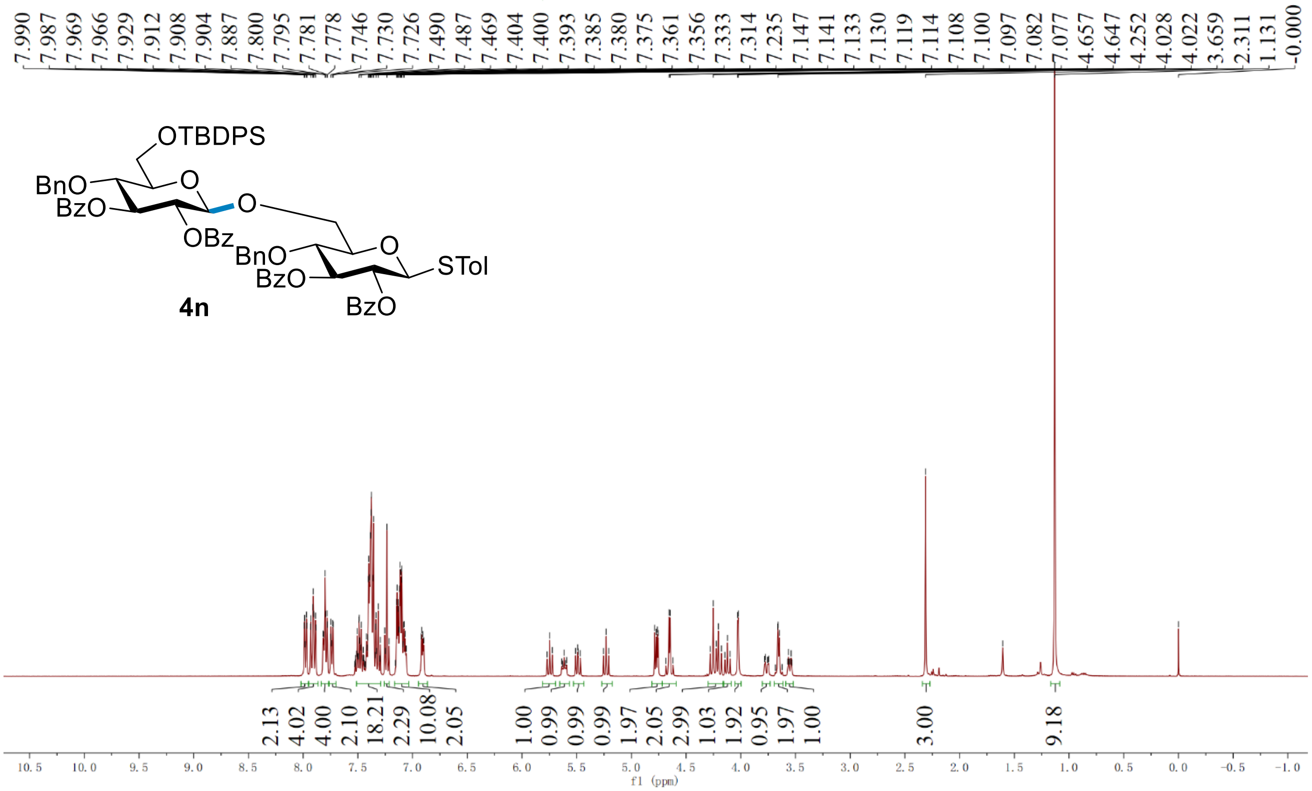

^{13}C NMR Spectrum of **4n** (101 MHz, $CDCl_3$)

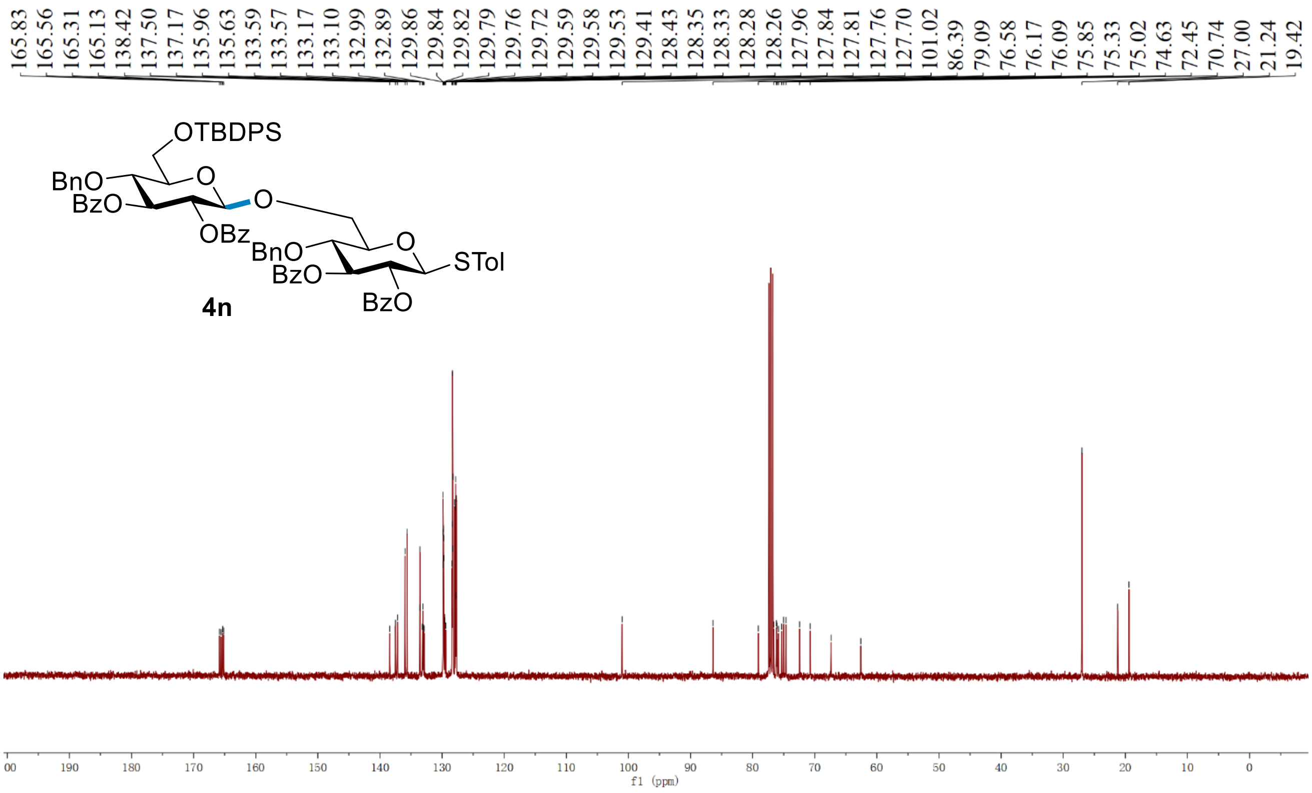

^{1}H NMR Spectrum of **4o** (400 MHz, $CDCl_3$)

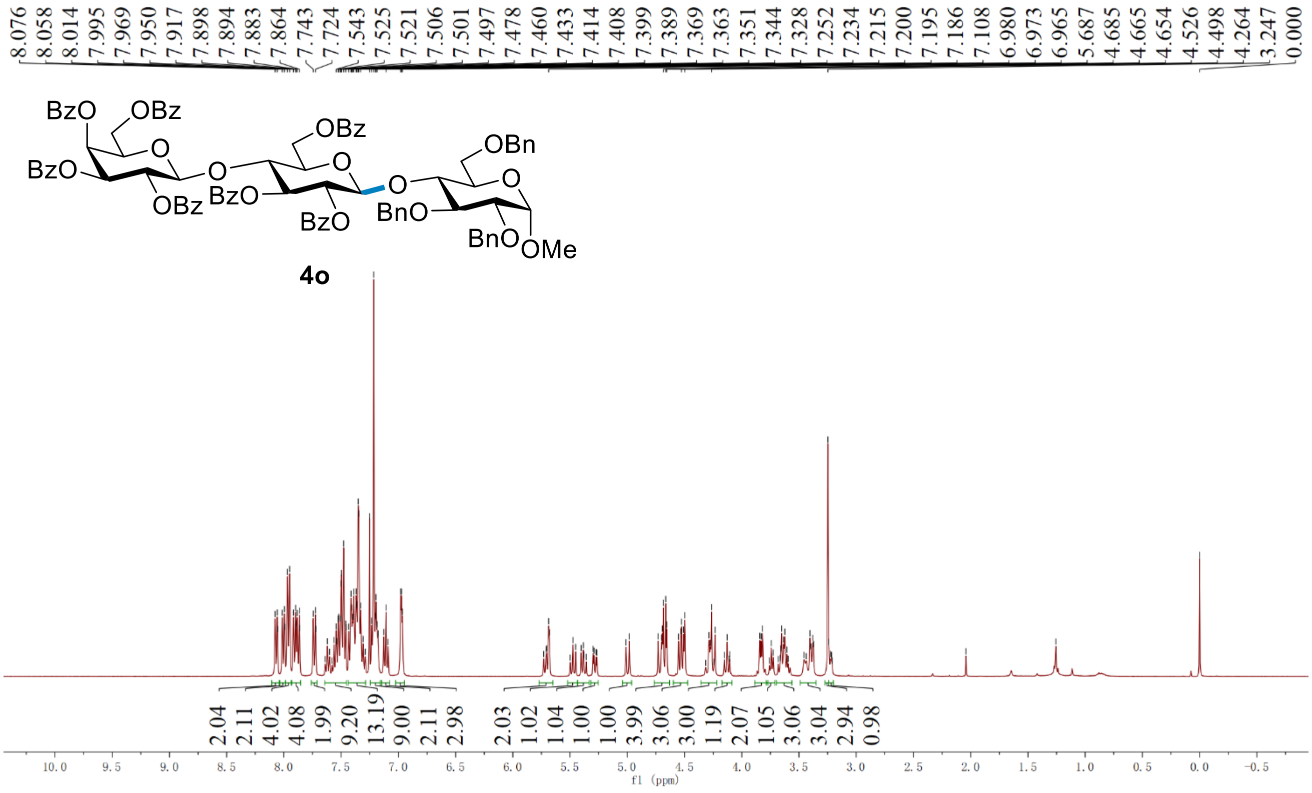

^{13}C NMR Spectrum of **4o** (101 MHz, $CDCl_3$)

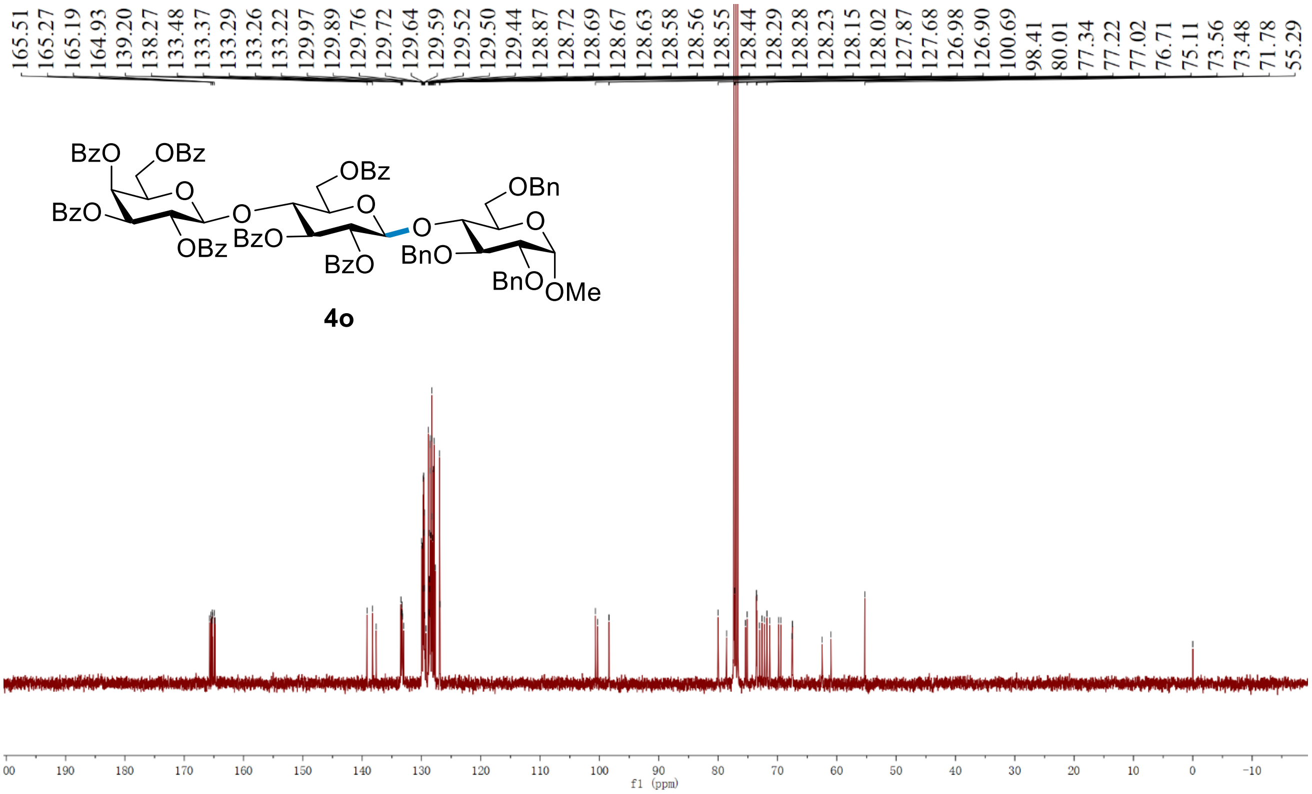

^{1}H NMR Spectrum of **5a** (400 MHz, $CDCl_3$)

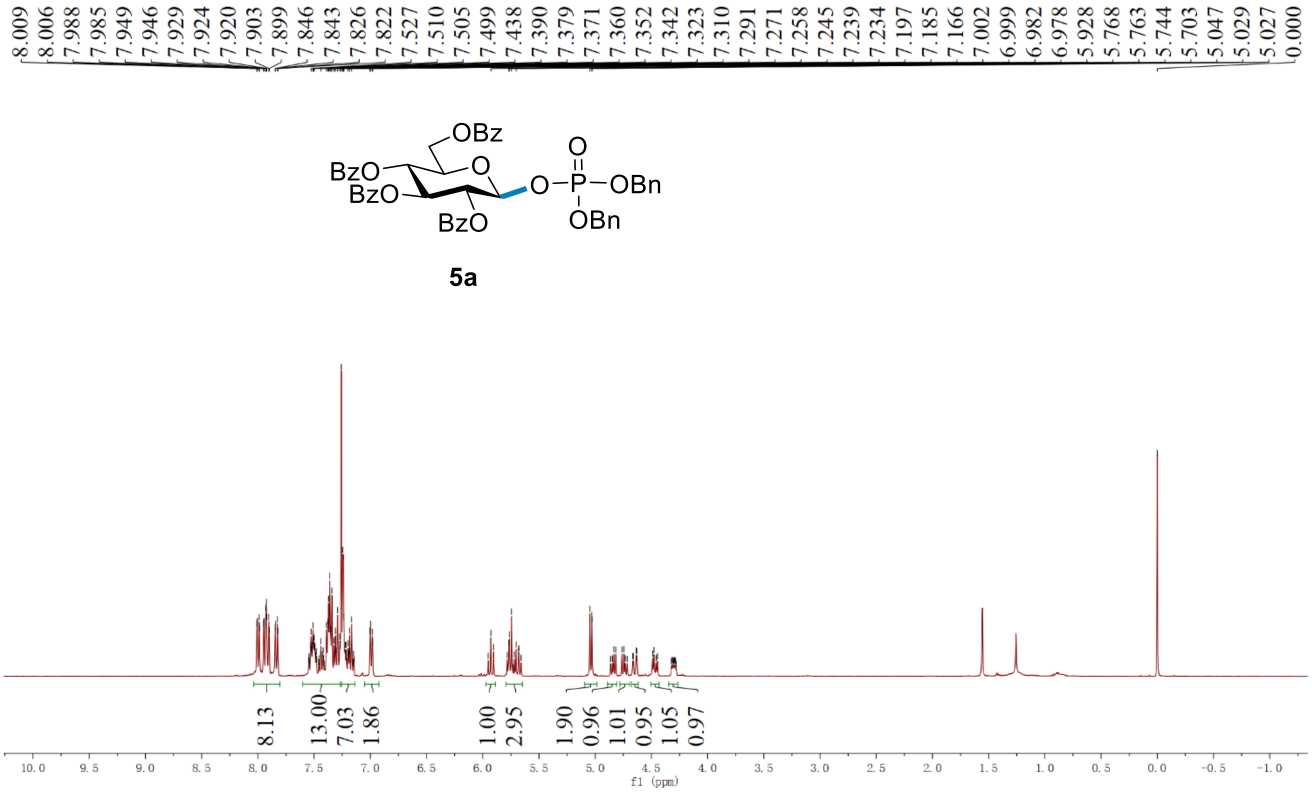

^{31}P NMR Spectrum of **5a** (162 MHz, $CDCl_3$)

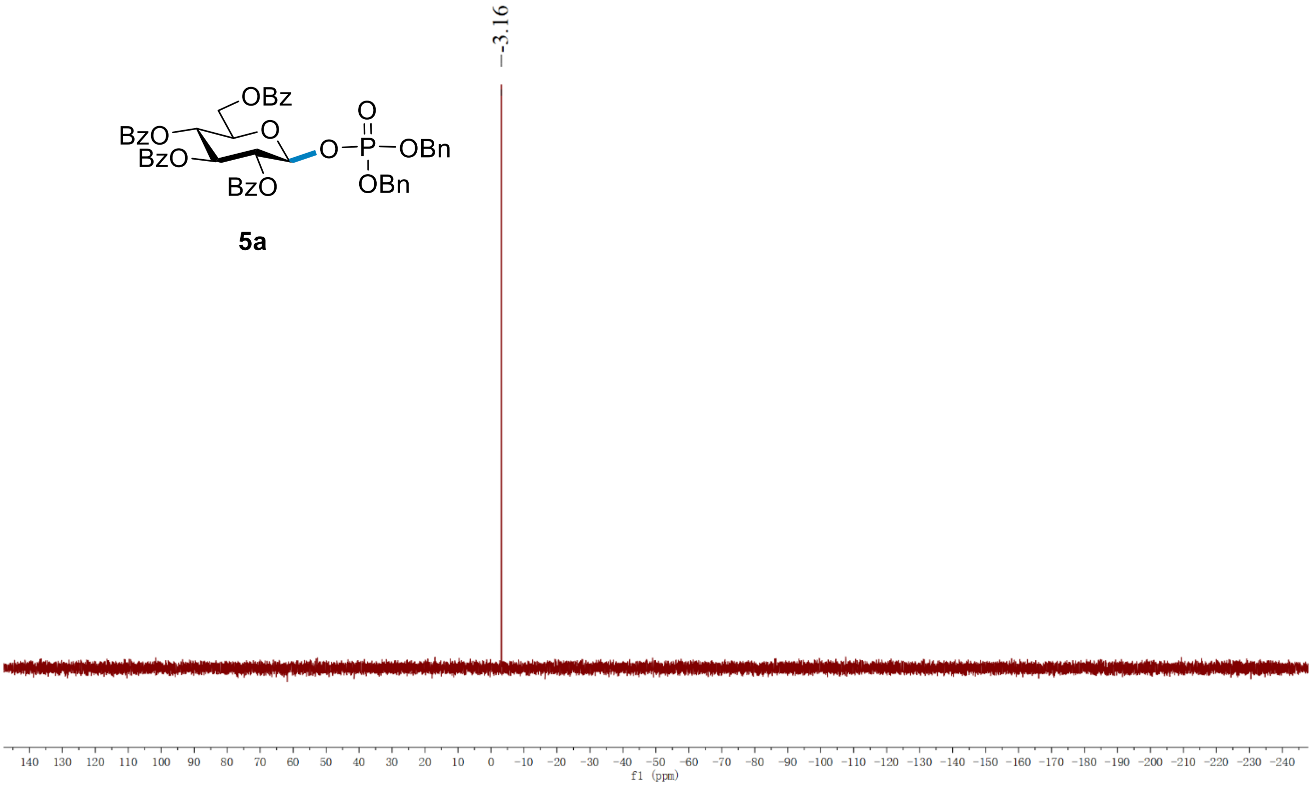

^{1}H NMR Spectrum of **5b** (400 MHz, $CDCl_3$)

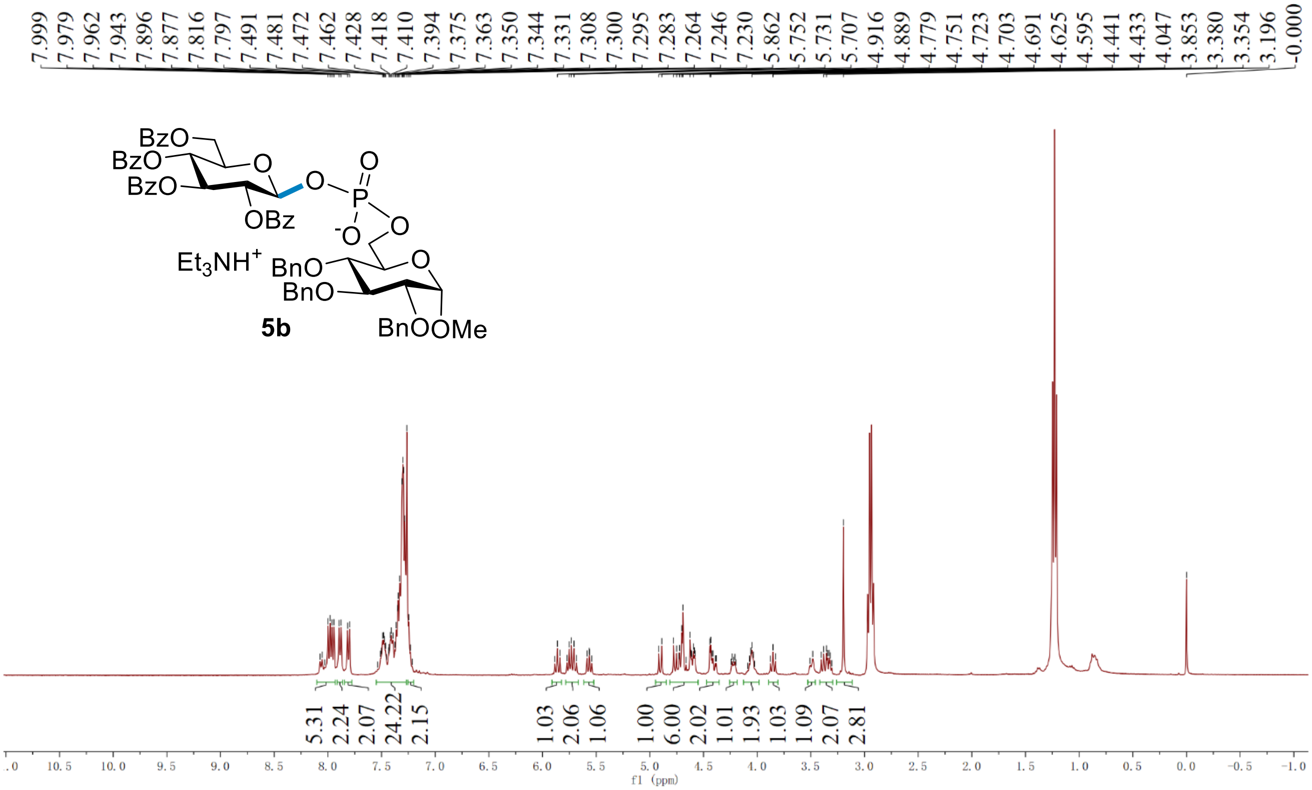

^{31}P NMR Spectrum of **5b** (162 MHz, $CDCl_3$)

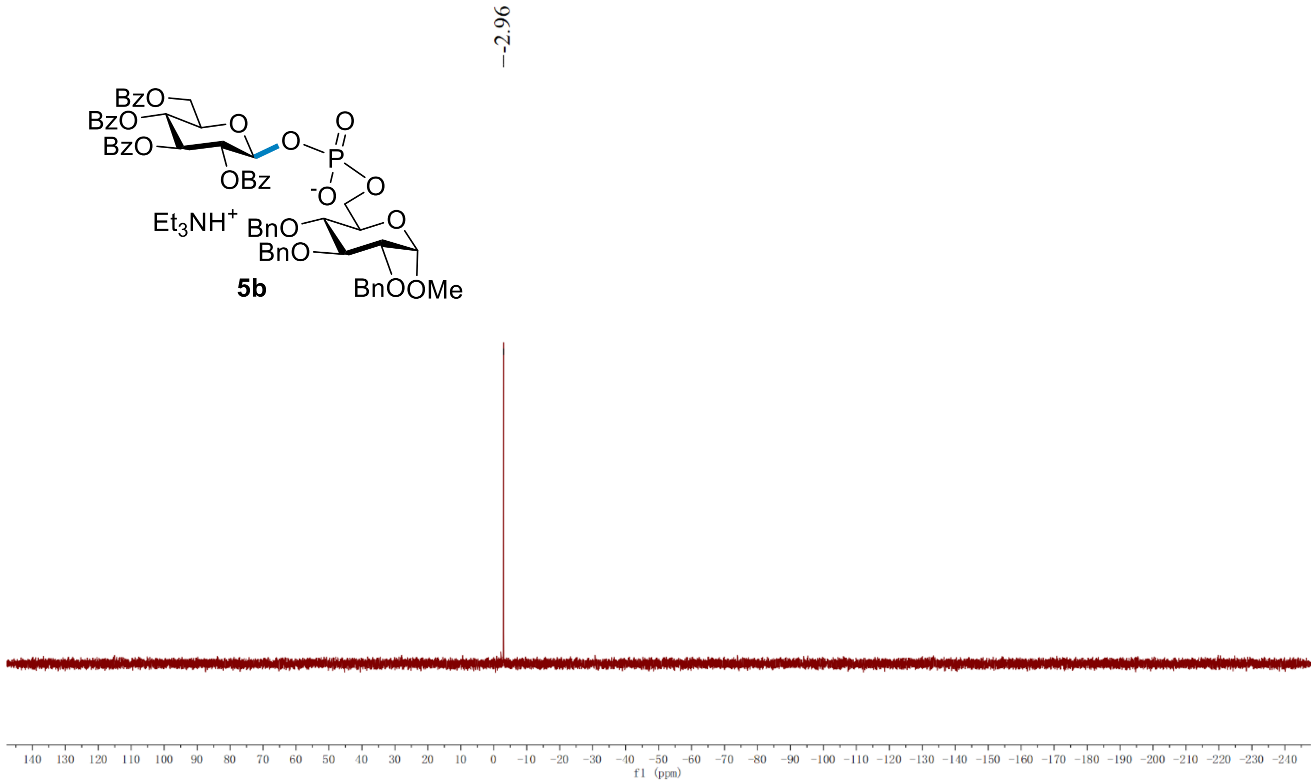

^{13}C NMR Spectrum of **5b** (101 MHz, $CDCl_3$)

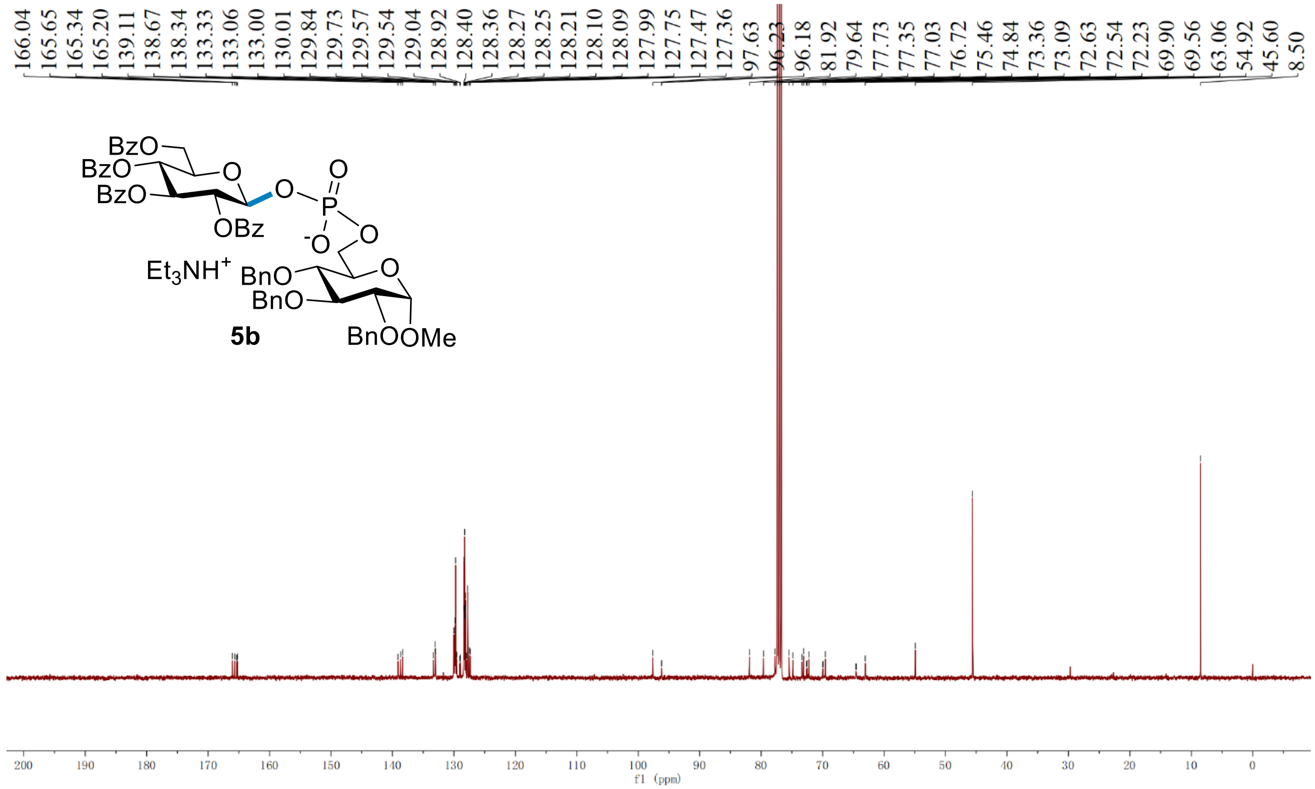

^{1}H NMR Spectrum of **5c** (400 MHz, $CDCl_3$)

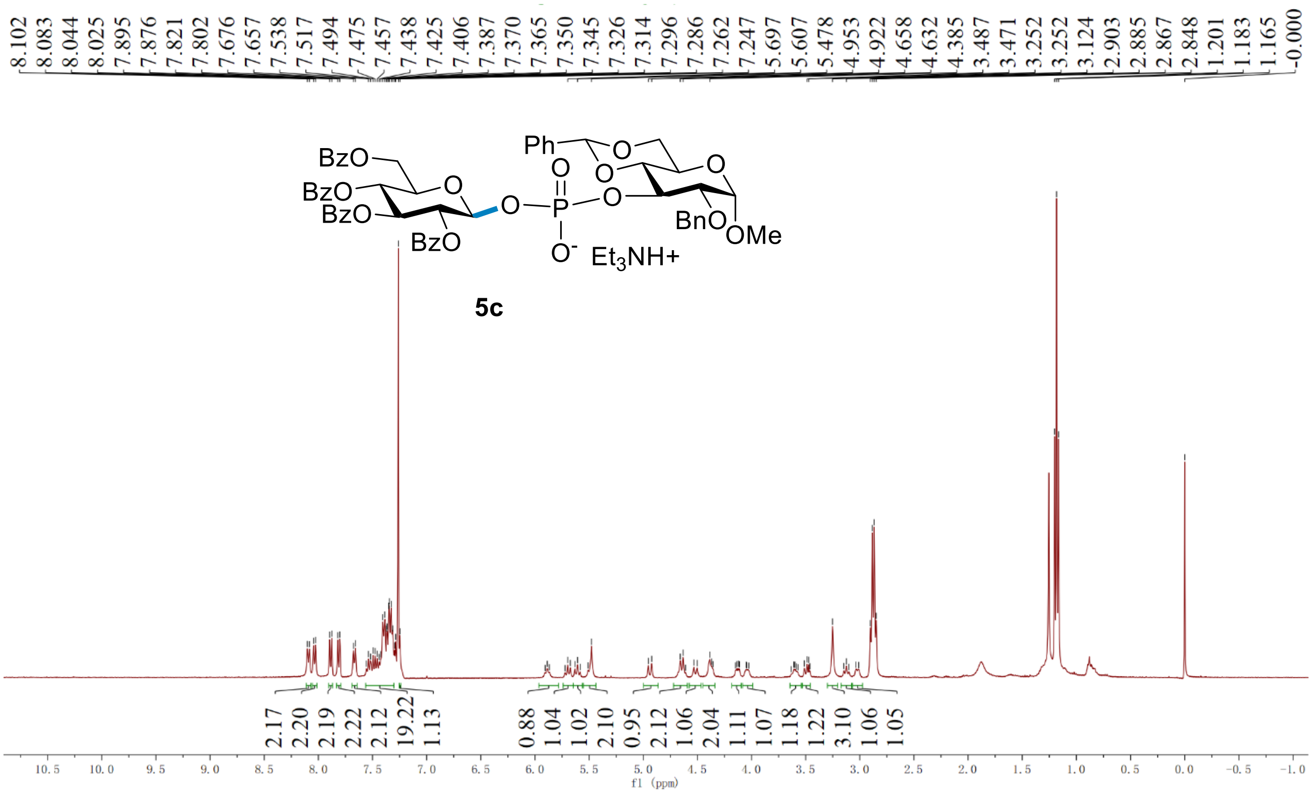

^{31}P NMR Spectrum of **5c** (162 MHz, $CDCl_3$)

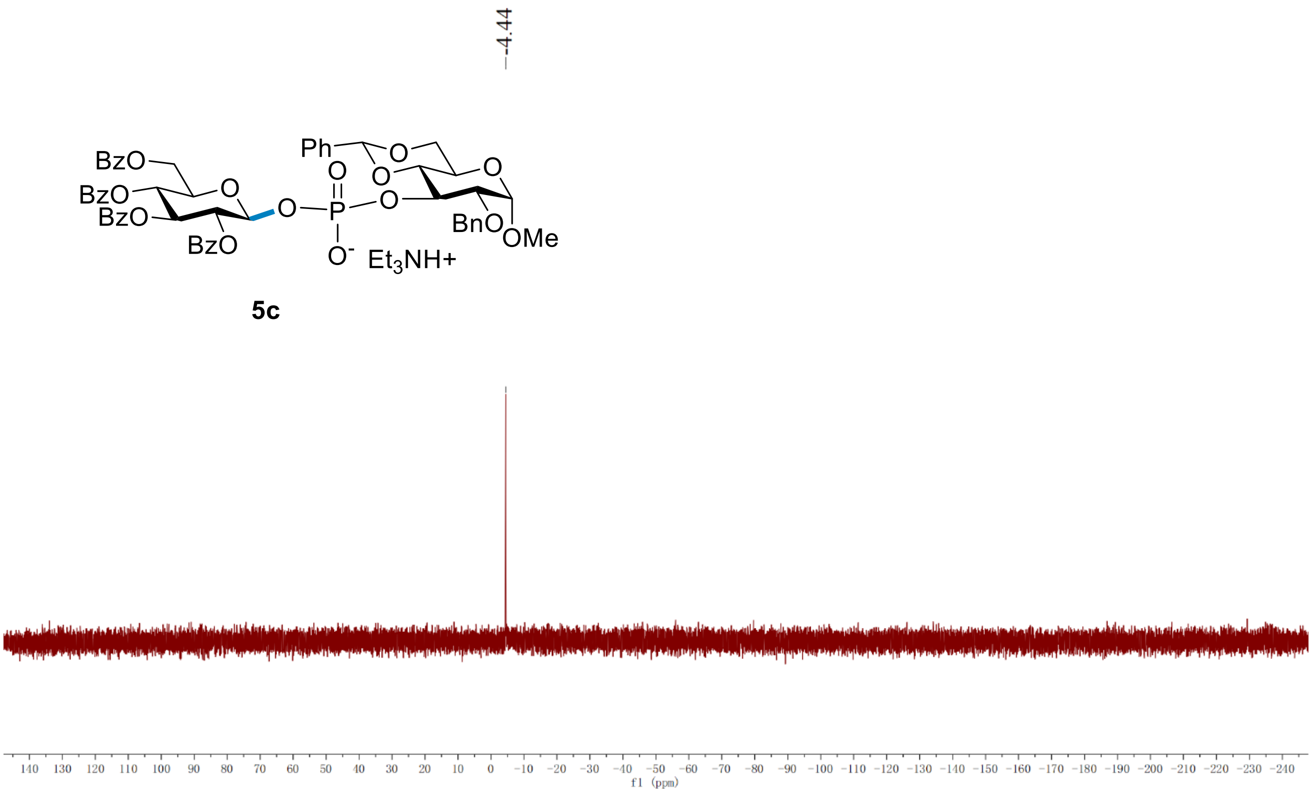

^{13}C NMR Spectrum of **5c** (101 MHz, $CDCl_3$)

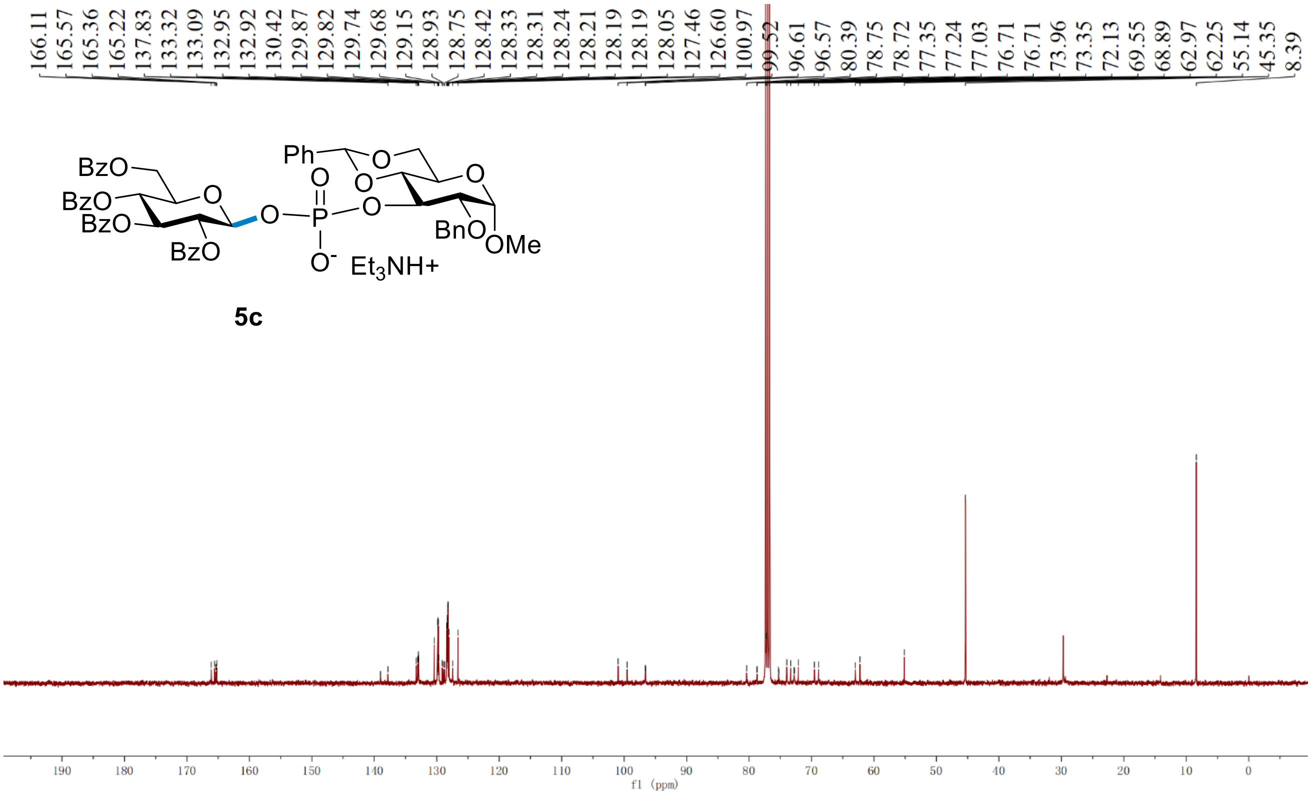

^{1}H NMR Spectrum of **5d** (400 MHz, $CDCl_3$)

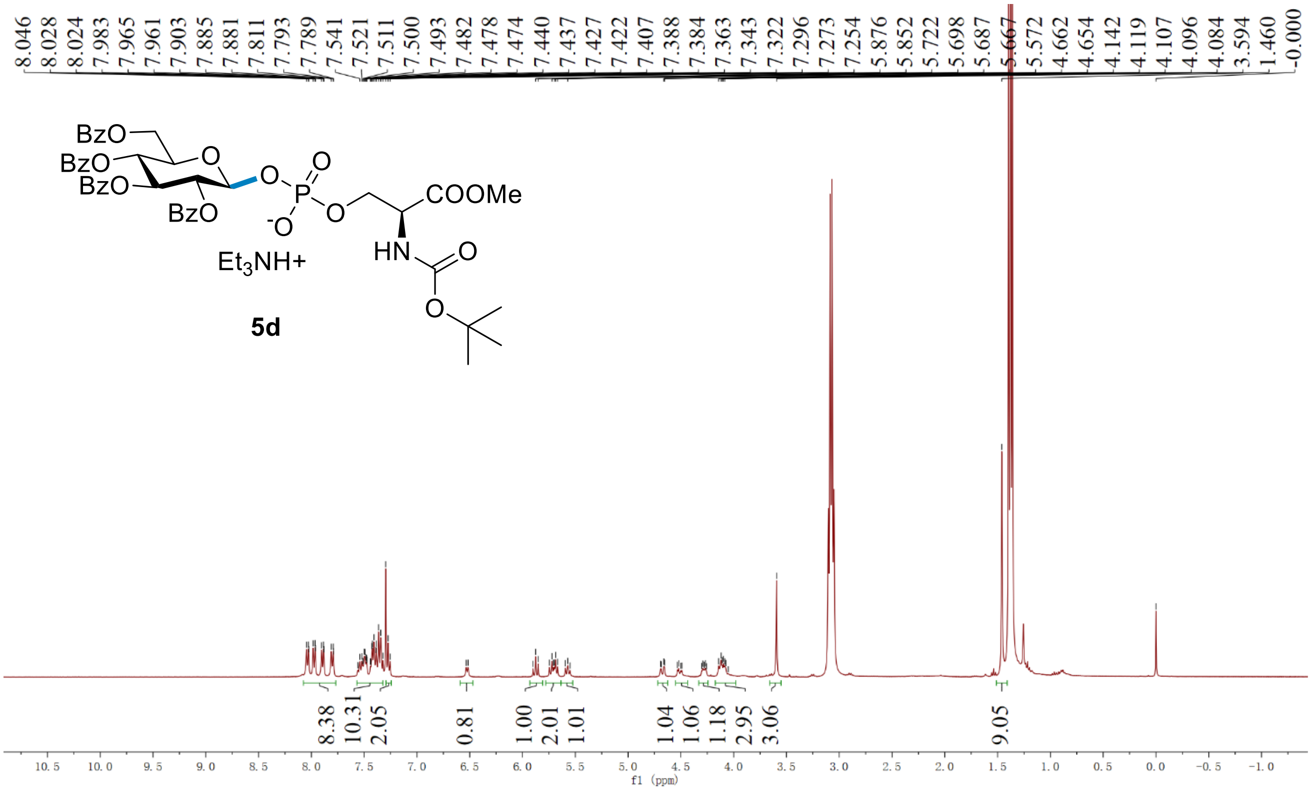

^{31}P NMR Spectrum of **5d** (162 MHz, $CDCl_3$)

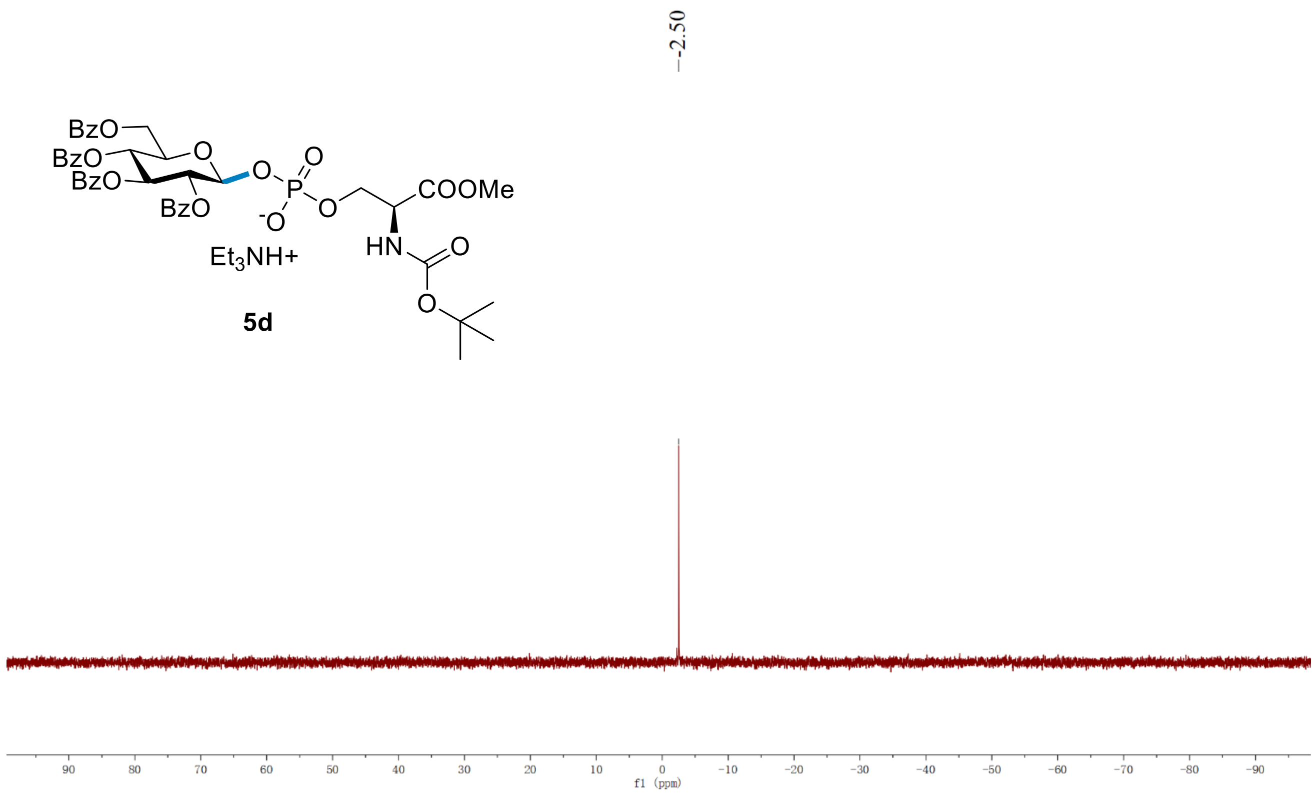

^{13}C NMR Spectrum of **5d** (101 MHz, $CDCl_3$)

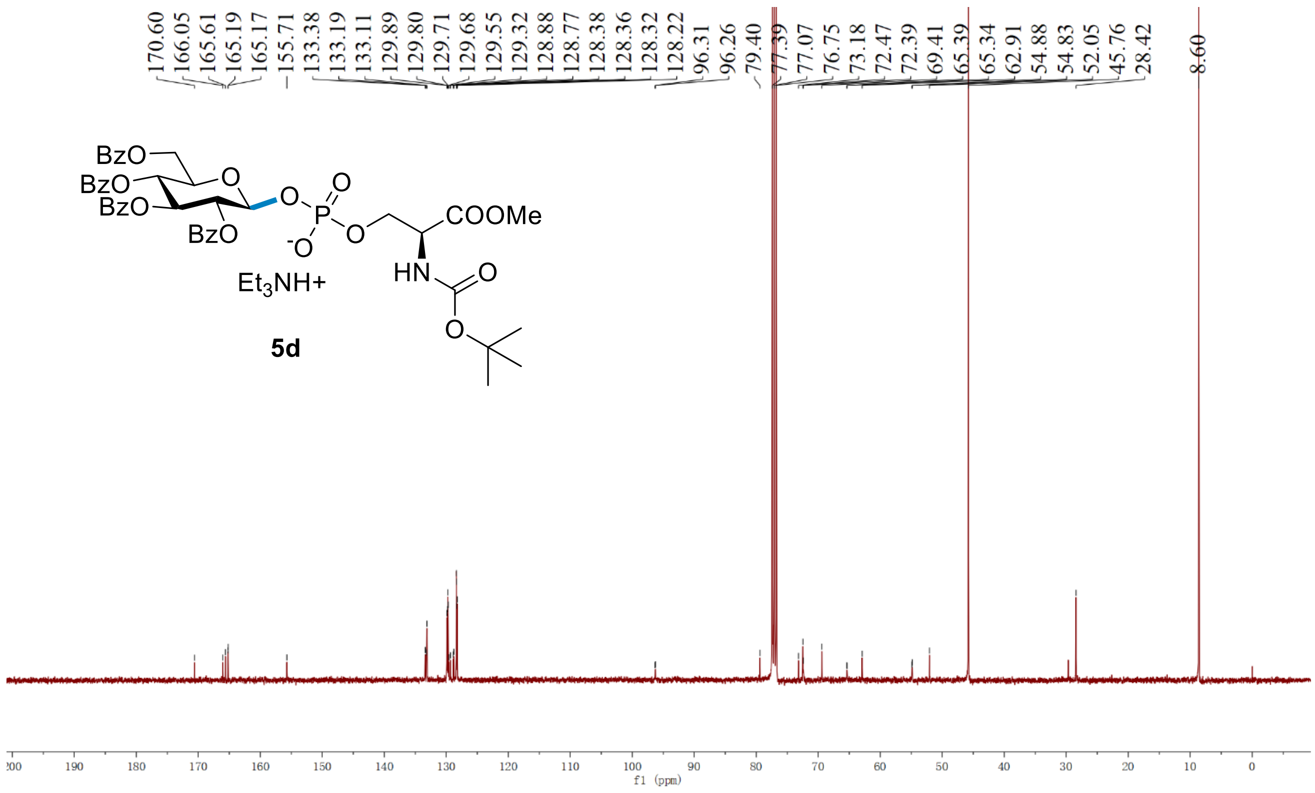

^{1}H NMR Spectrum of **5e** (400 MHz, $CDCl_3$)

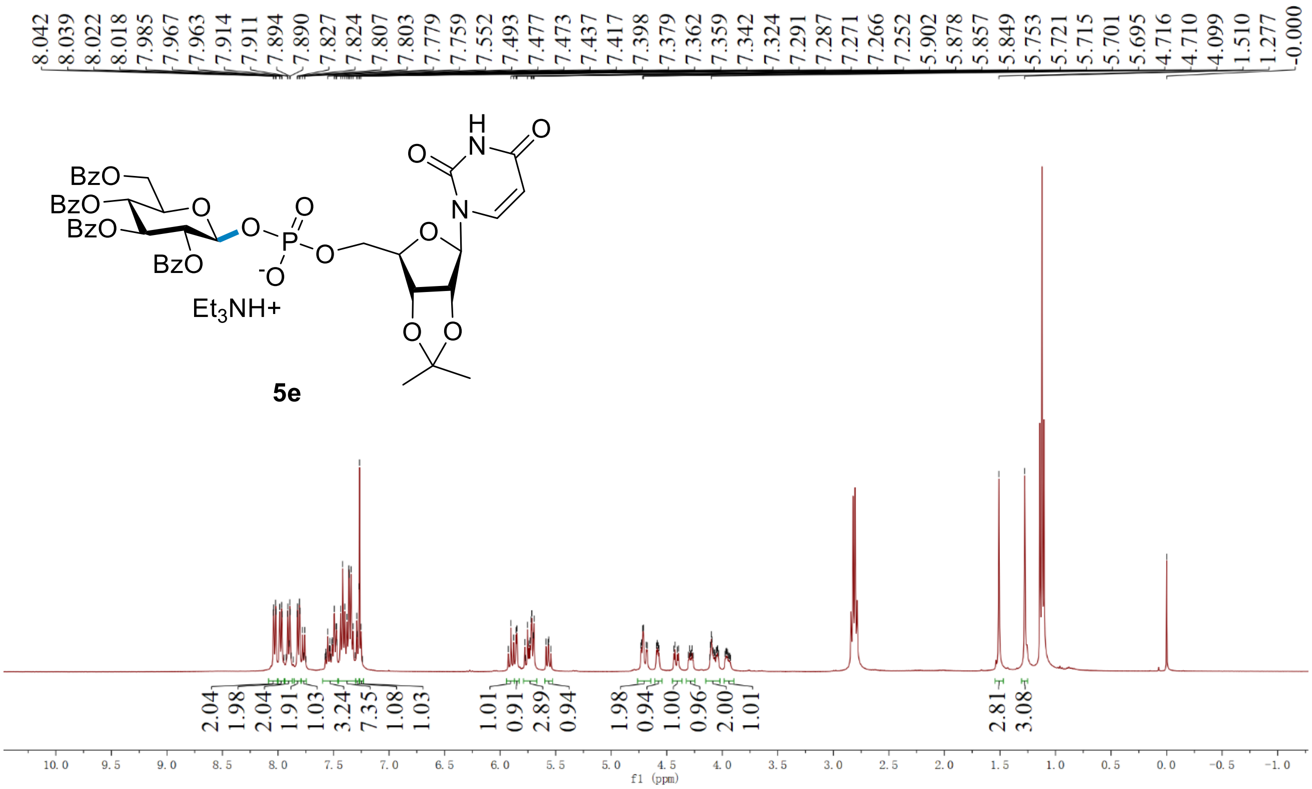

^{31}P NMR Spectrum of **5e** (162 MHz, $CDCl_3$)

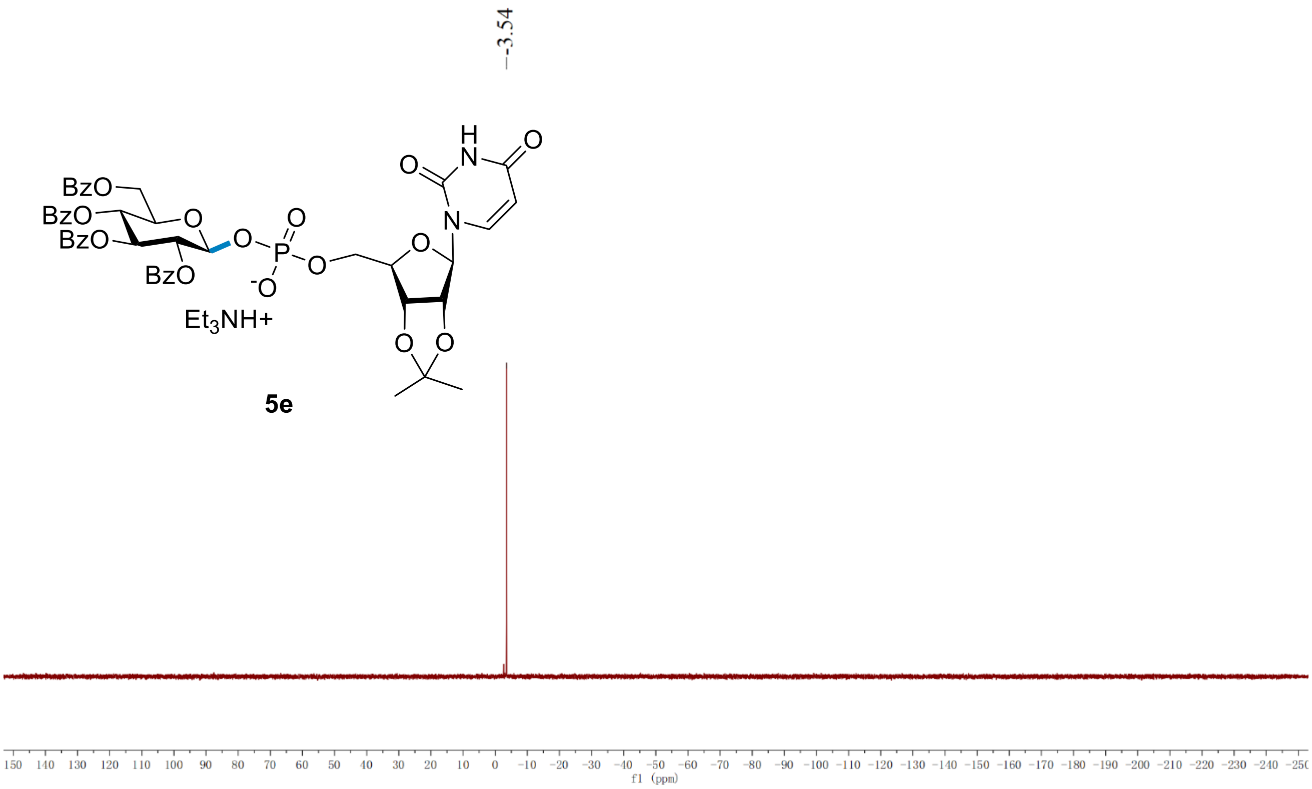

^{13}C NMR Spectrum of **5e** (101 MHz, $CDCl_3$)

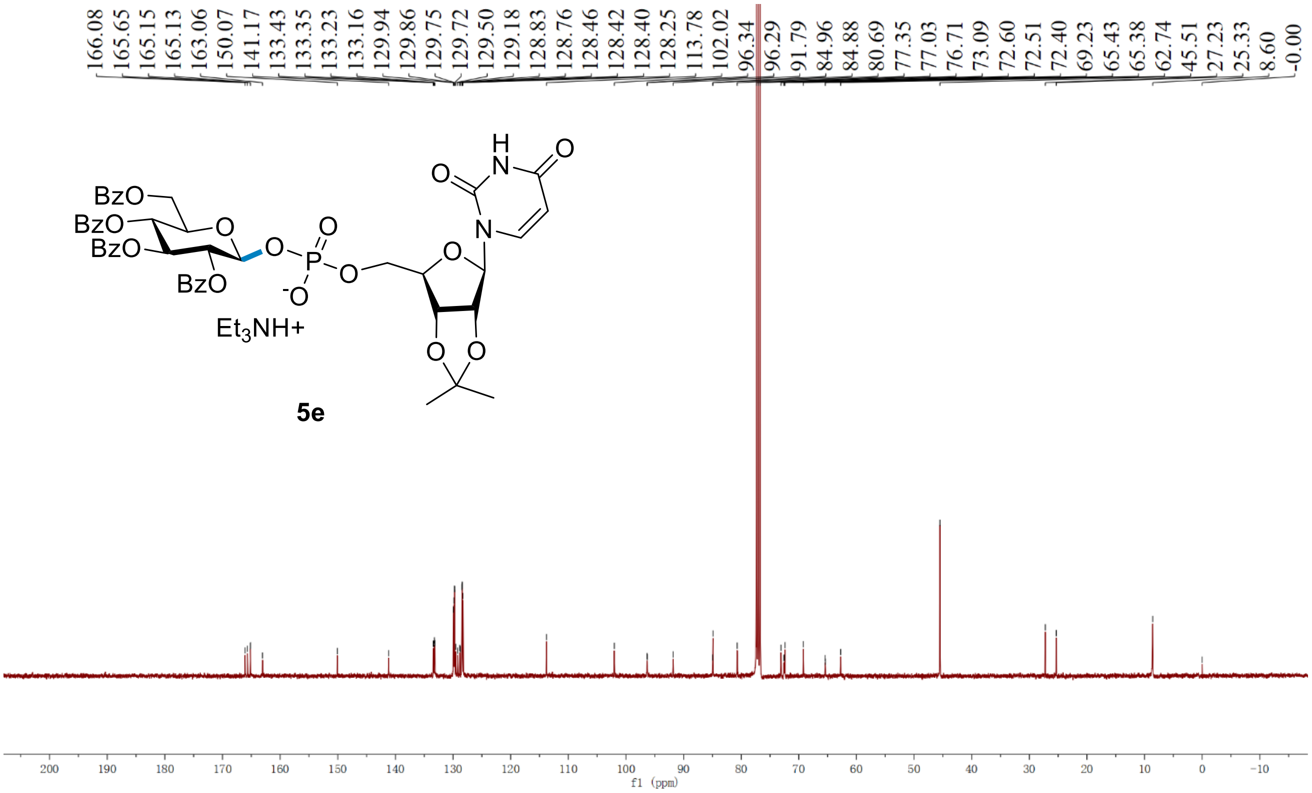

^{1}H NMR Spectrum of **5f** (400 MHz, $CDCl_3$)

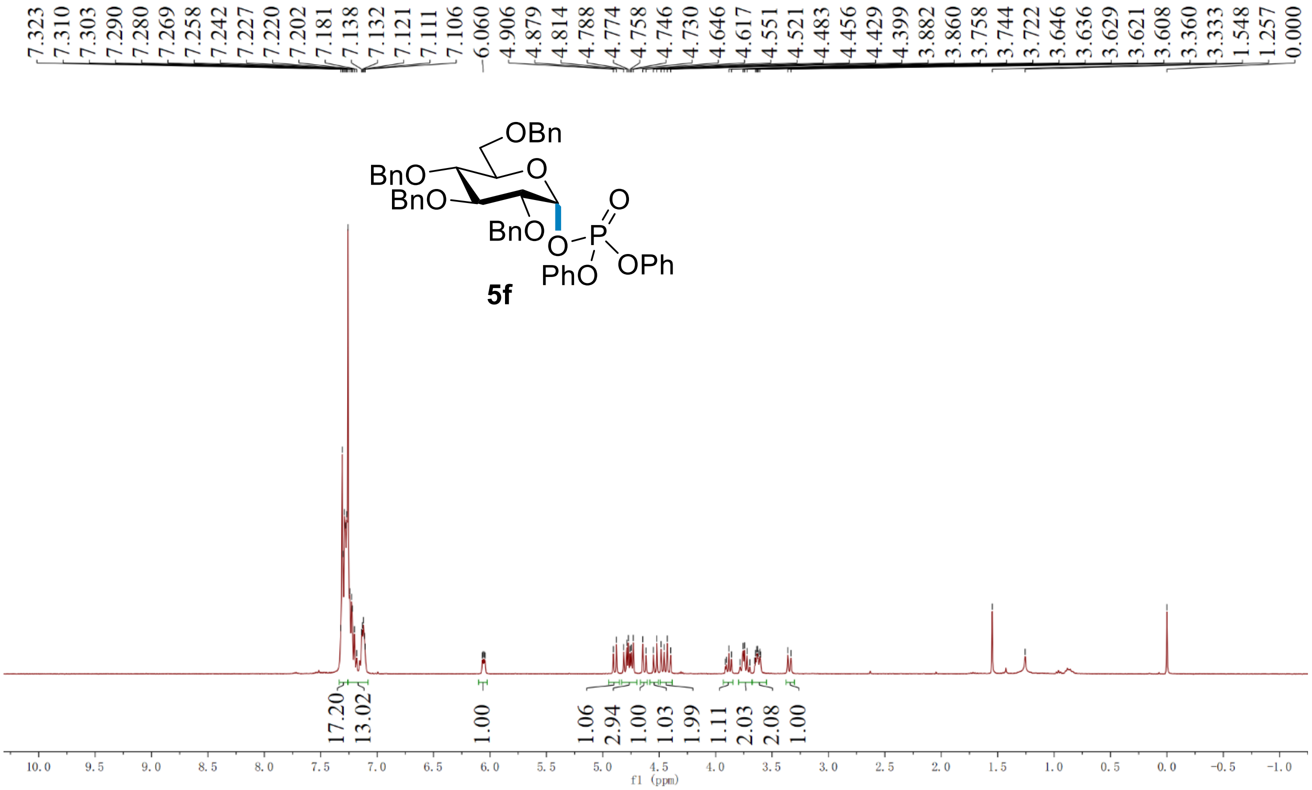

^{31}P NMR Spectrum of **5f** (162 MHz, $CDCl_3$)

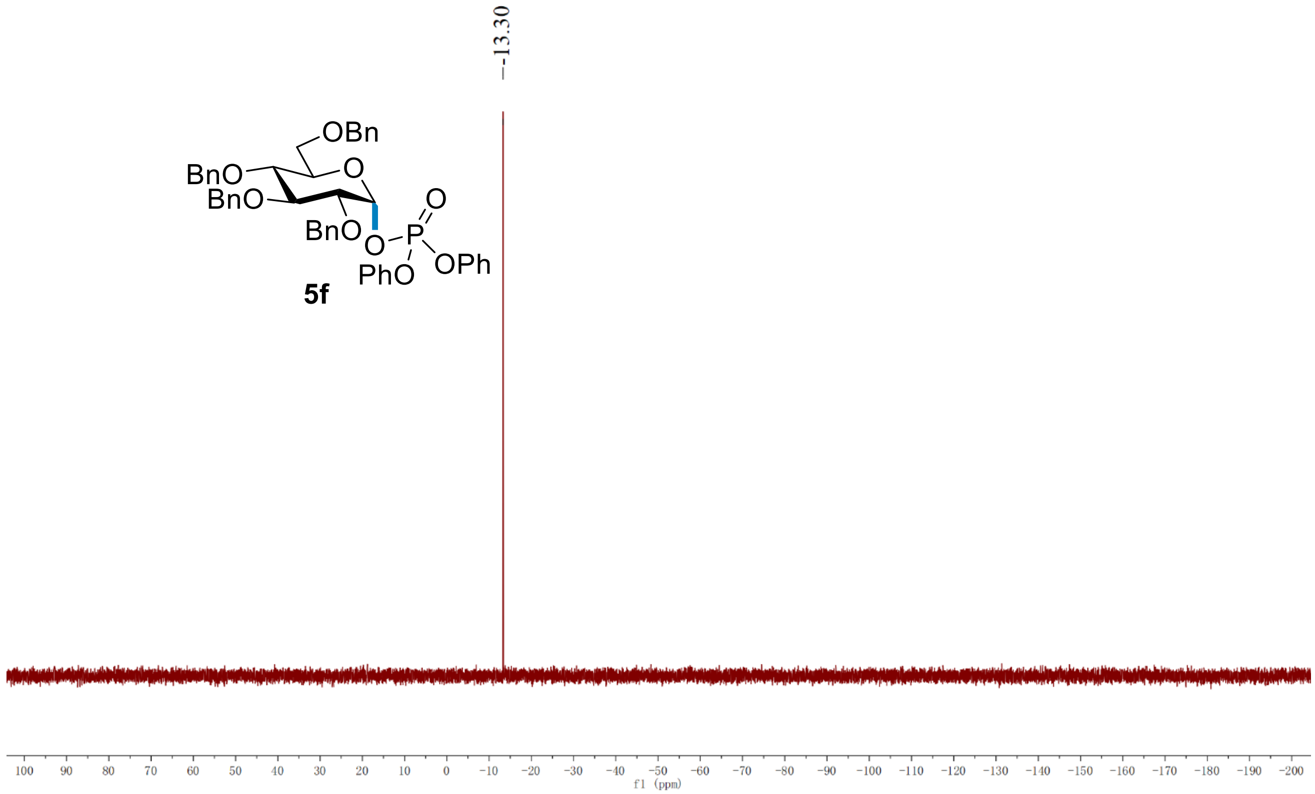

^{1}H NMR Spectrum of **5g** (400 MHz, $CDCl_3$)

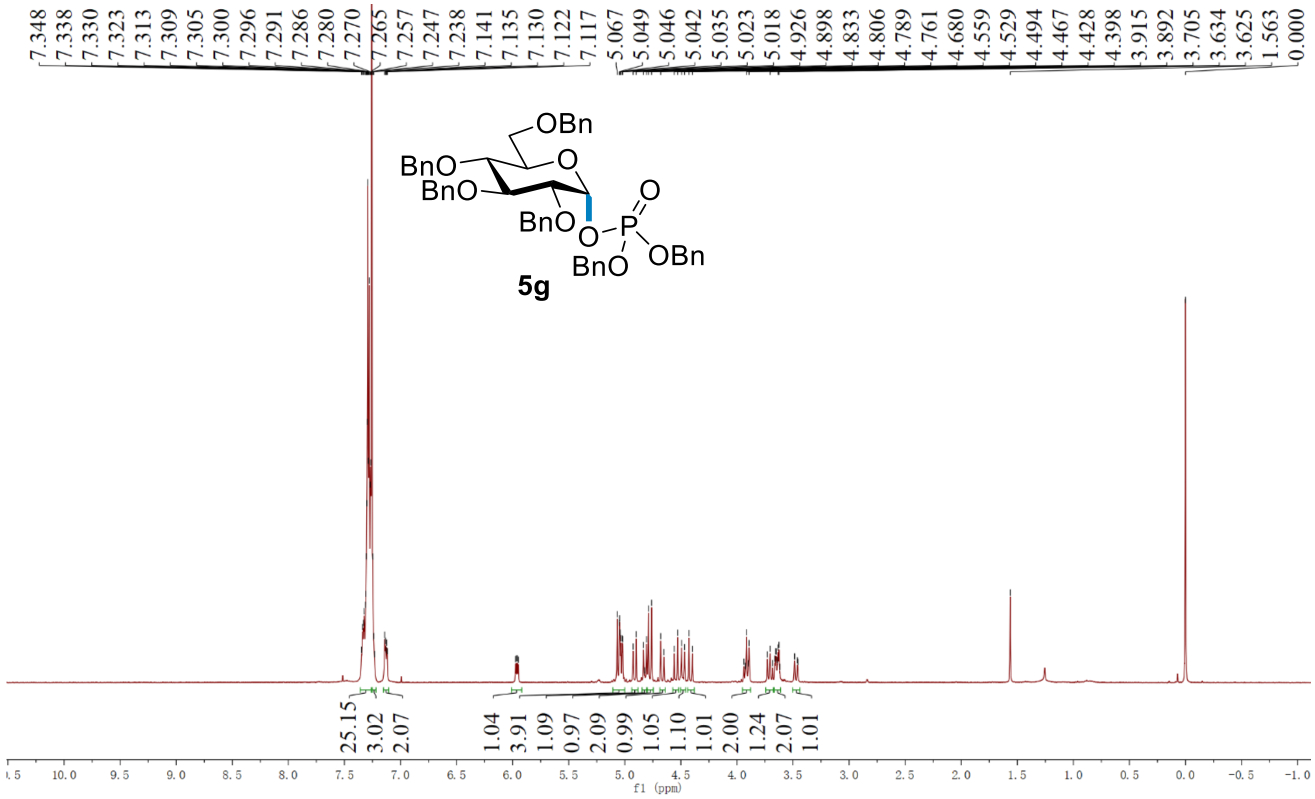

^{31}P NMR Spectrum of **5g** (162 MHz, $CDCl_3$)

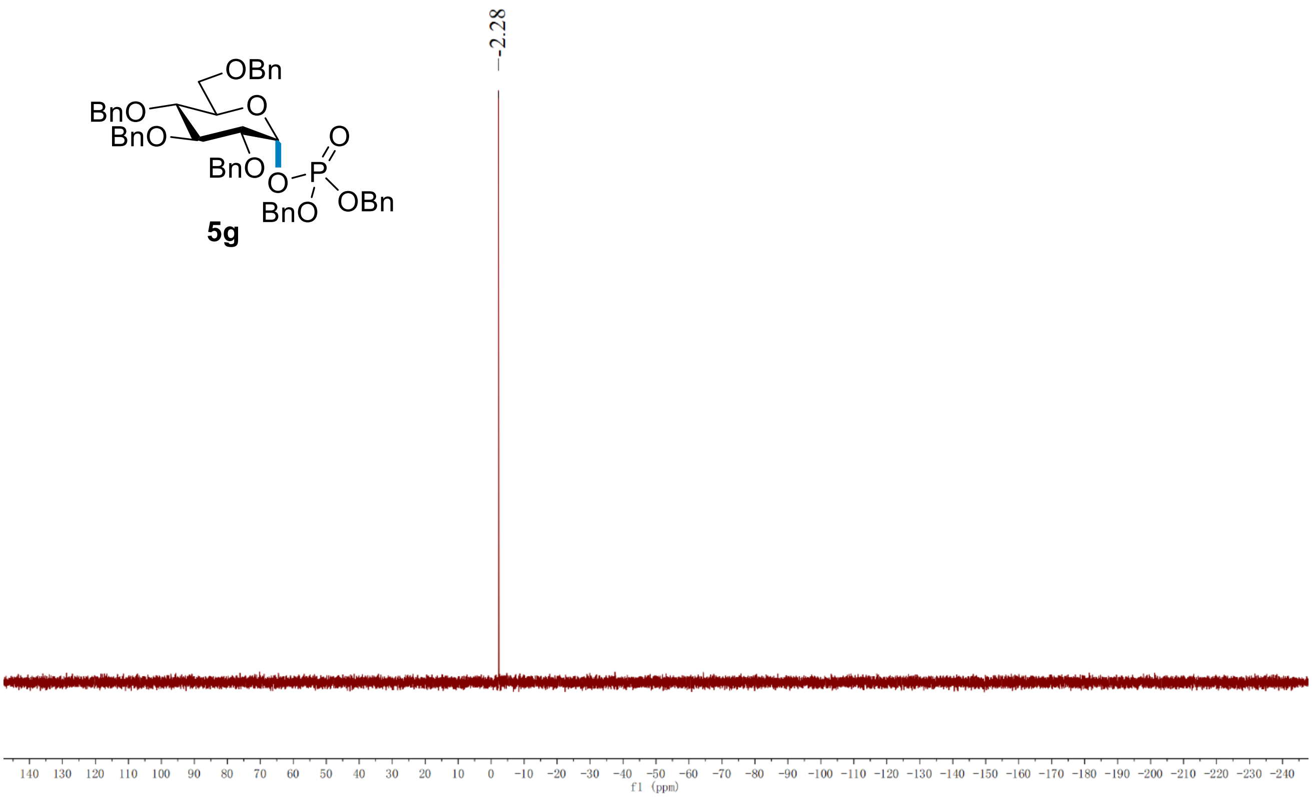

^{1}H NMR Spectrum of **5h** (400 MHz, $CDCl_3$)

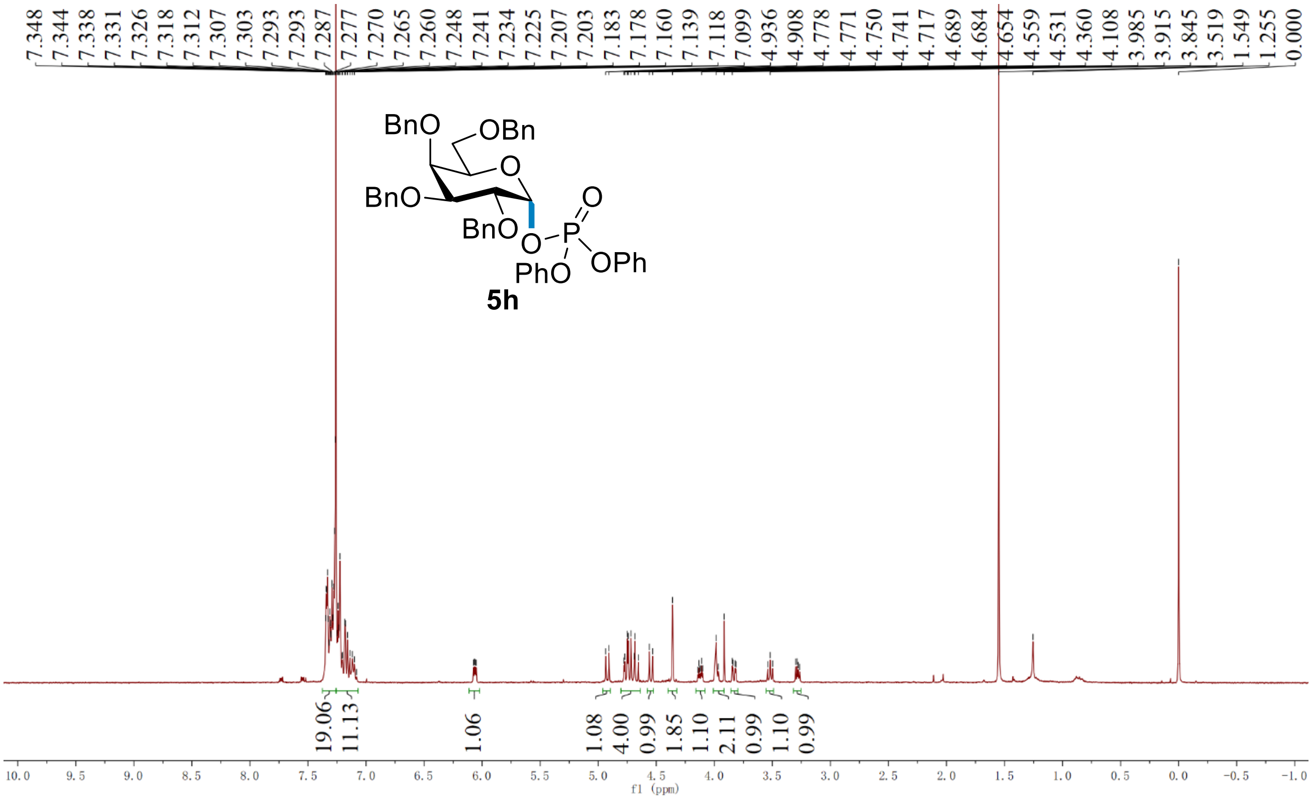

^{31}P NMR Spectrum of **5h** (162 MHz, $CDCl_3$)

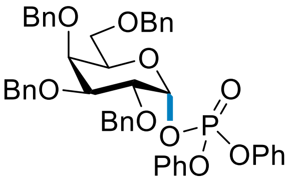

5h

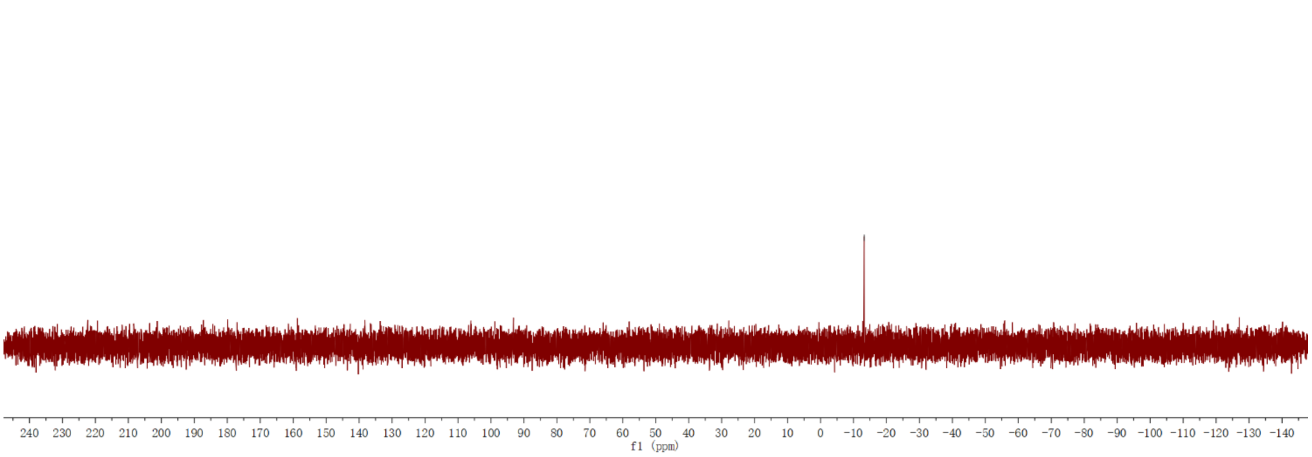

^{1}H NMR Spectrum of **5i** (400 MHz, $CDCl_3$)

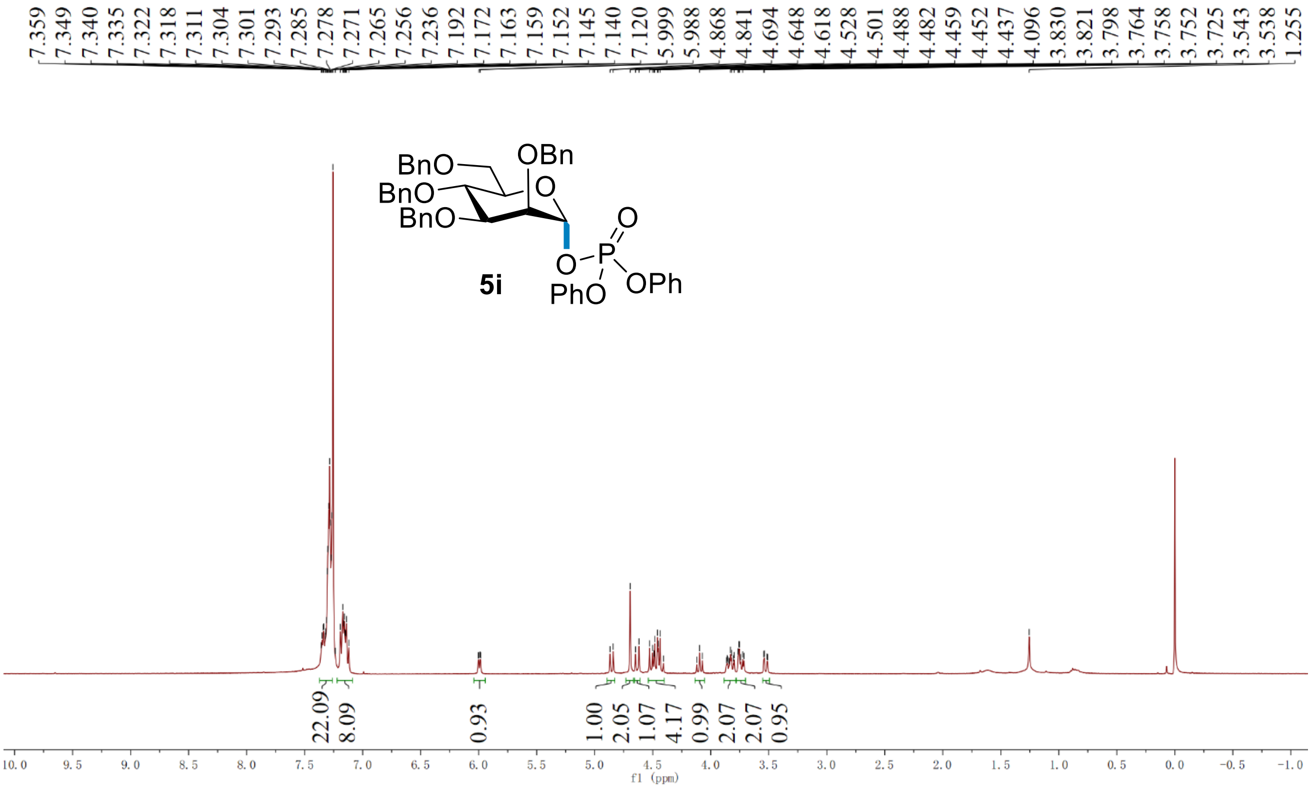

^{31}P NMR Spectrum of **5i** (162 MHz, $CDCl_3$)

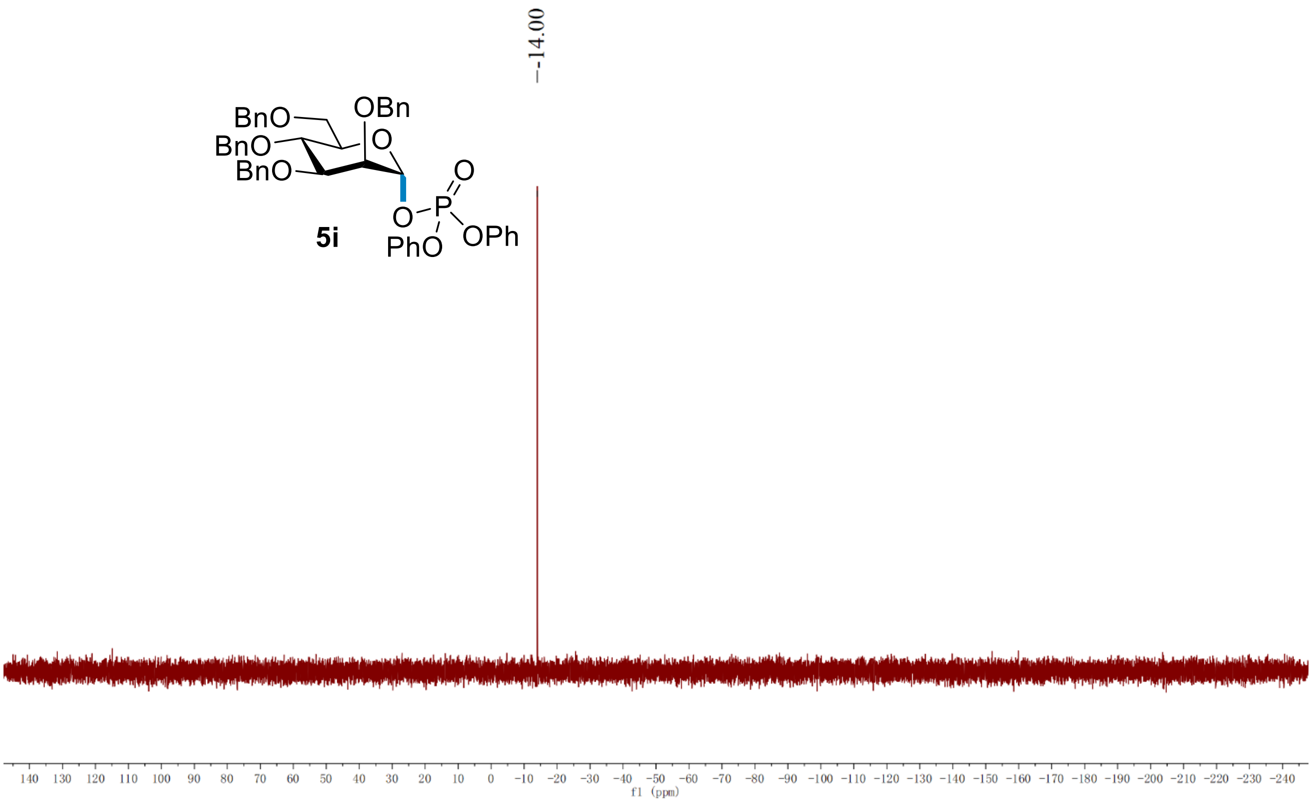

^{1}H NMR Spectrum of **5j** (400 MHz, $CDCl_3$)

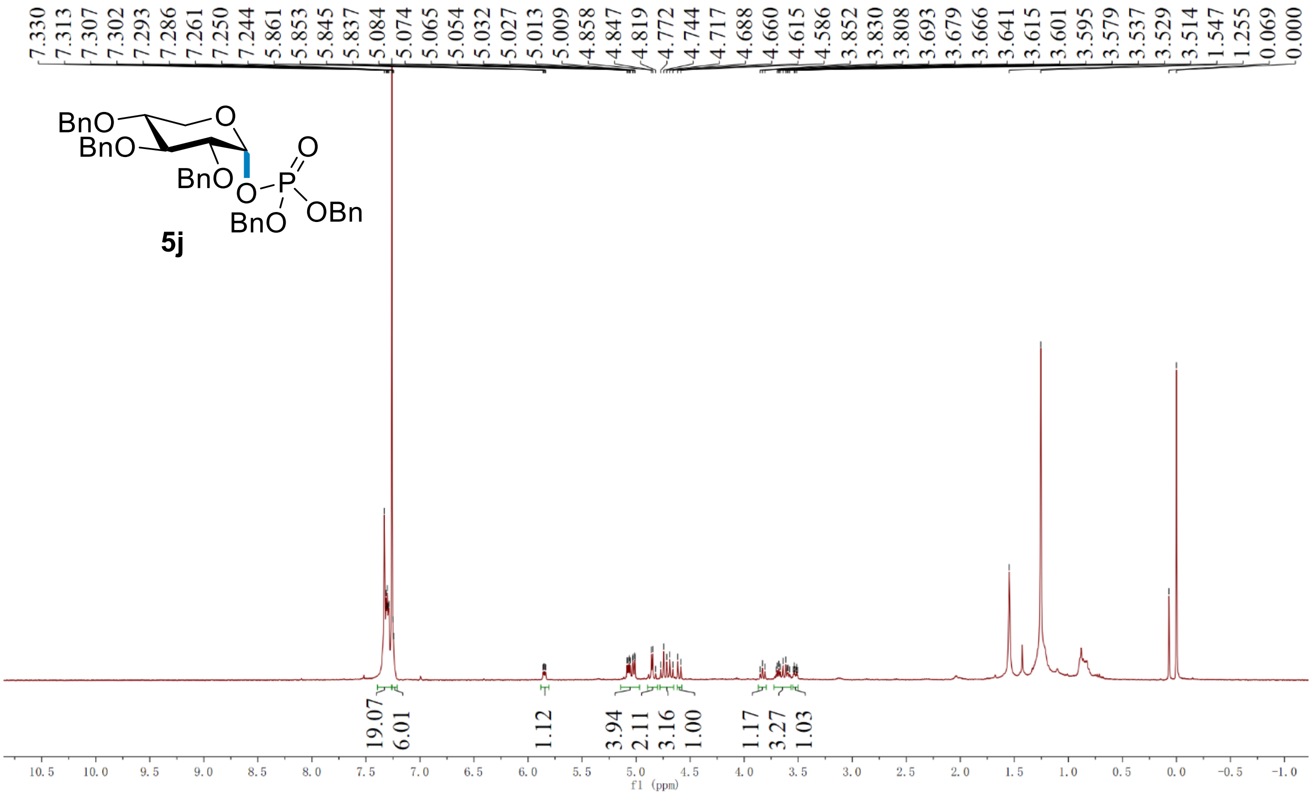

^{31}P NMR Spectrum of **5j** (162 MHz, $CDCl_3$)

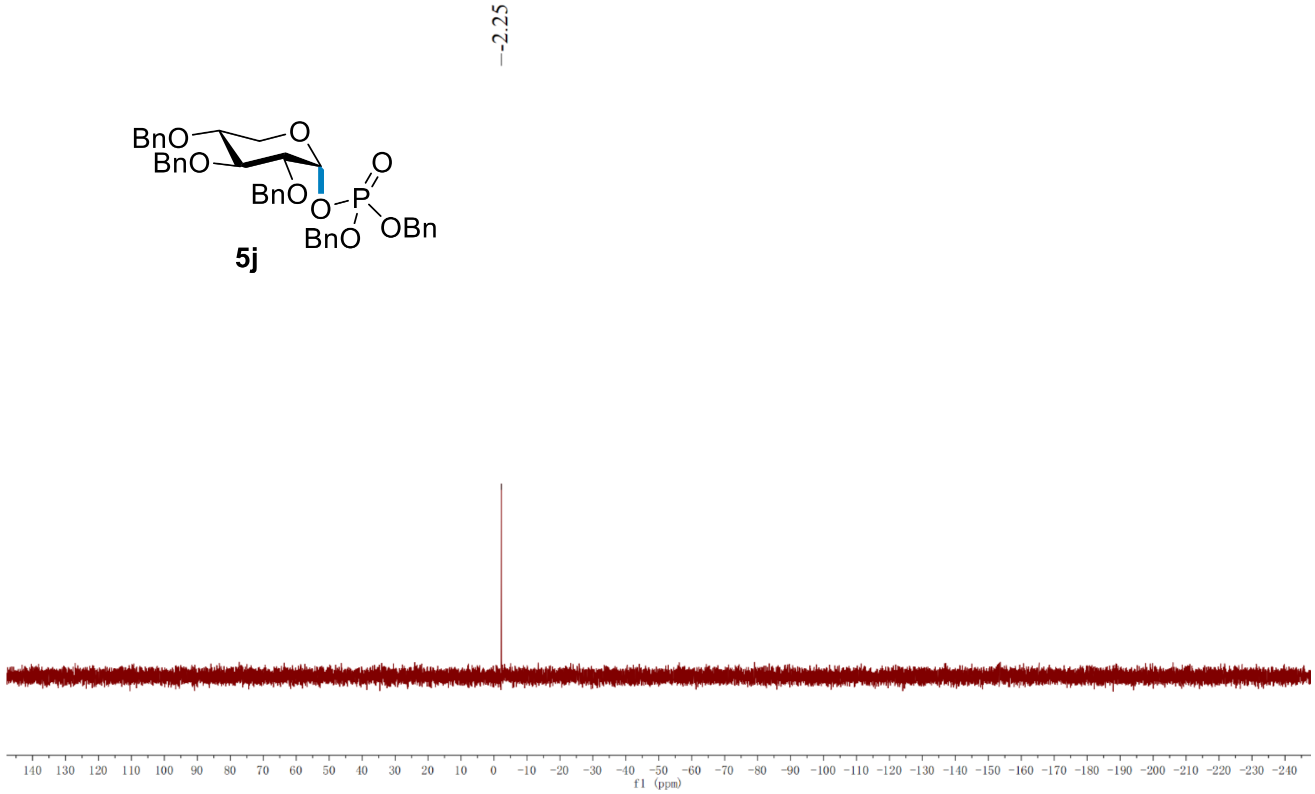

^{1}H NMR Spectrum of **5k** (400 MHz, $CDCl_3$)

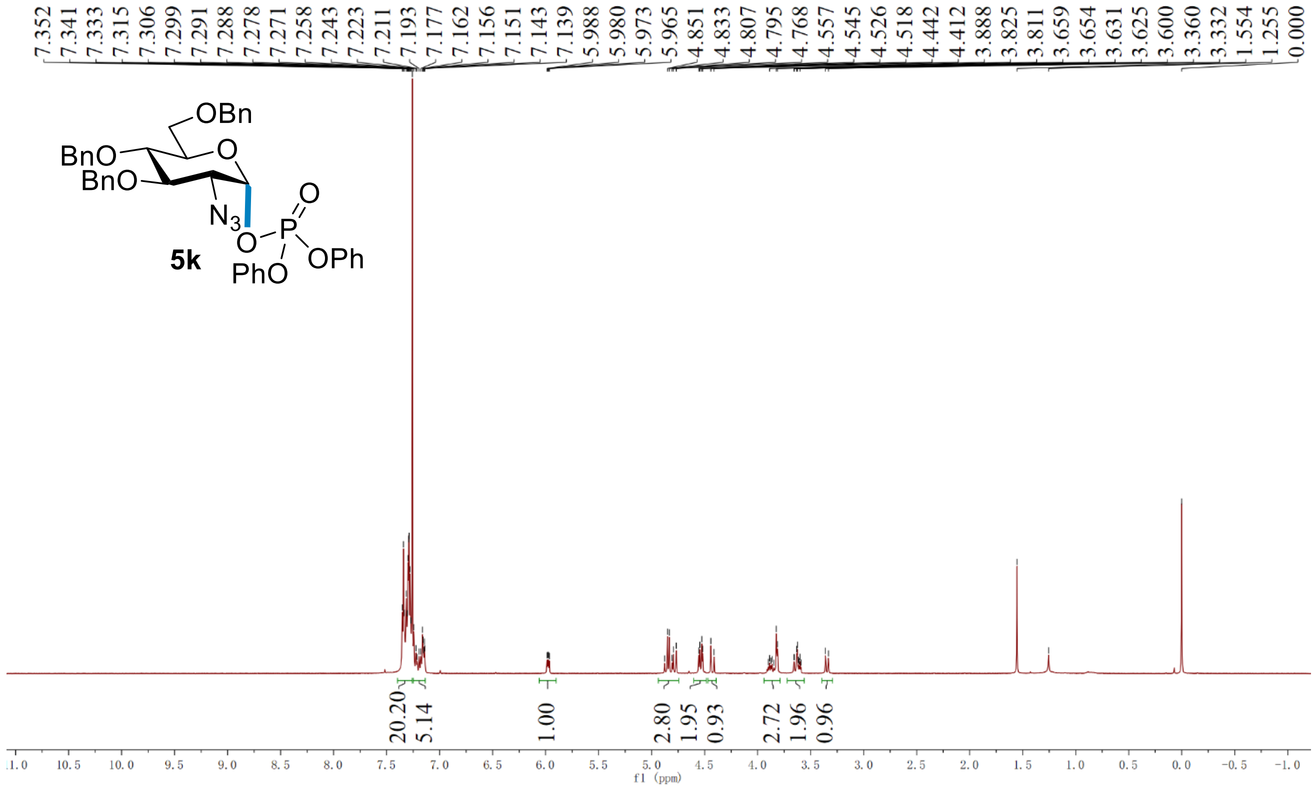

^{31}P NMR Spectrum of **5k** (162 MHz, $CDCl_3$)

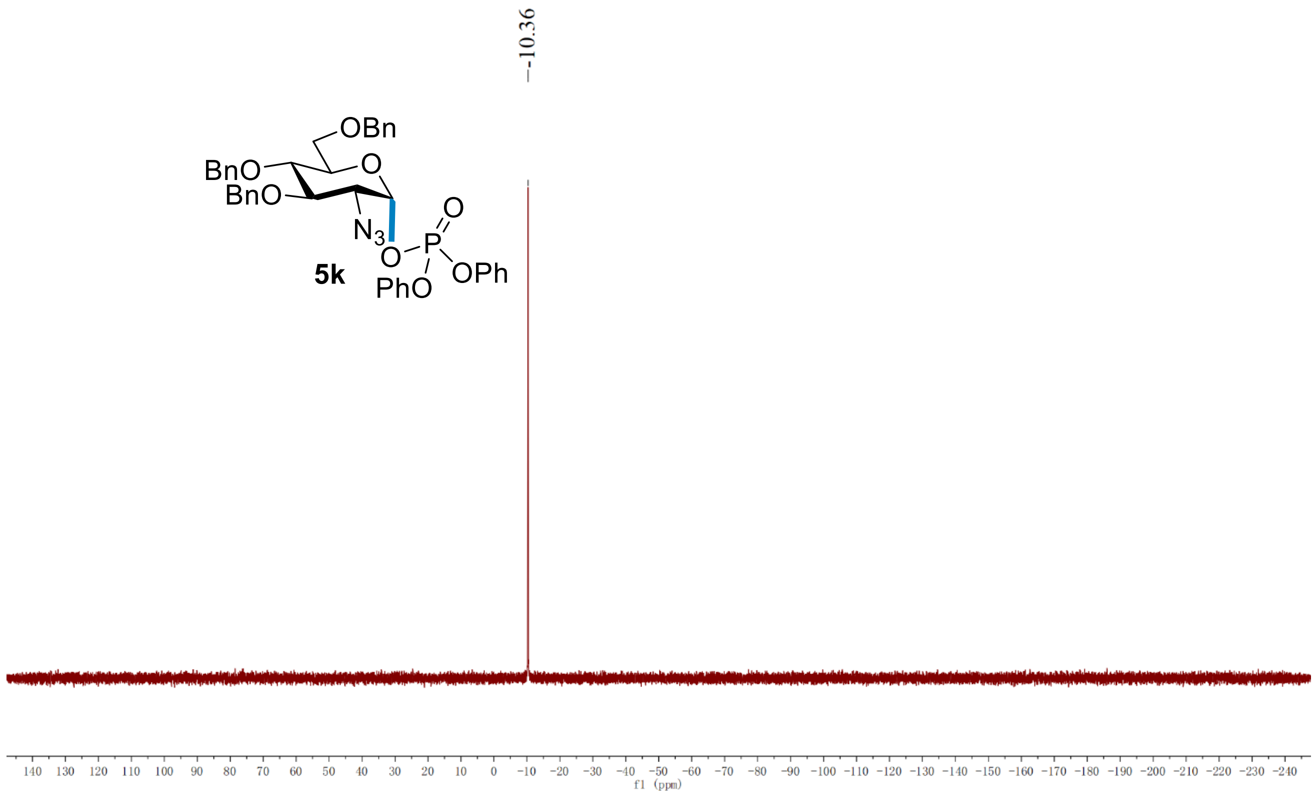

^{1}H NMR Spectrum of **5l** (600 MHz, $CDCl_3$)

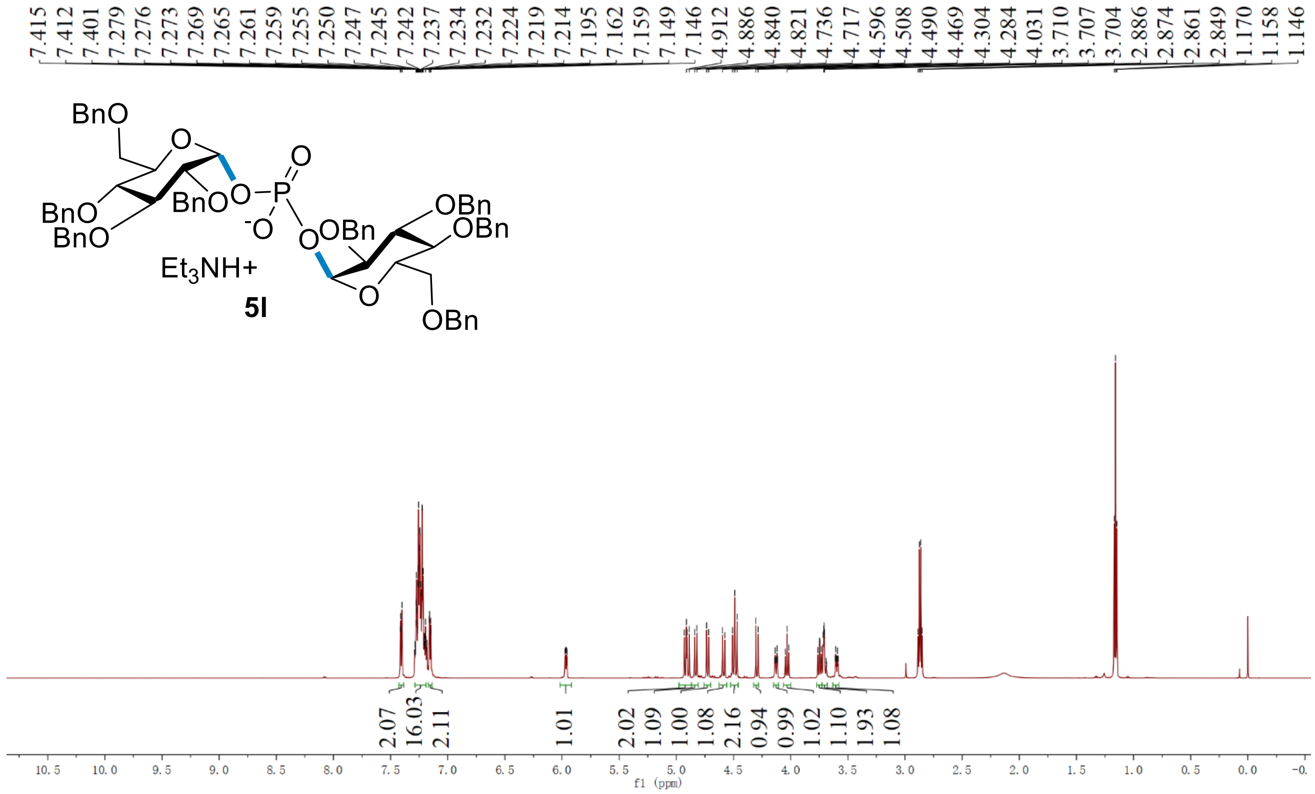

^{31}P NMR Spectrum of **5l** (162 MHz, $CDCl_3$)

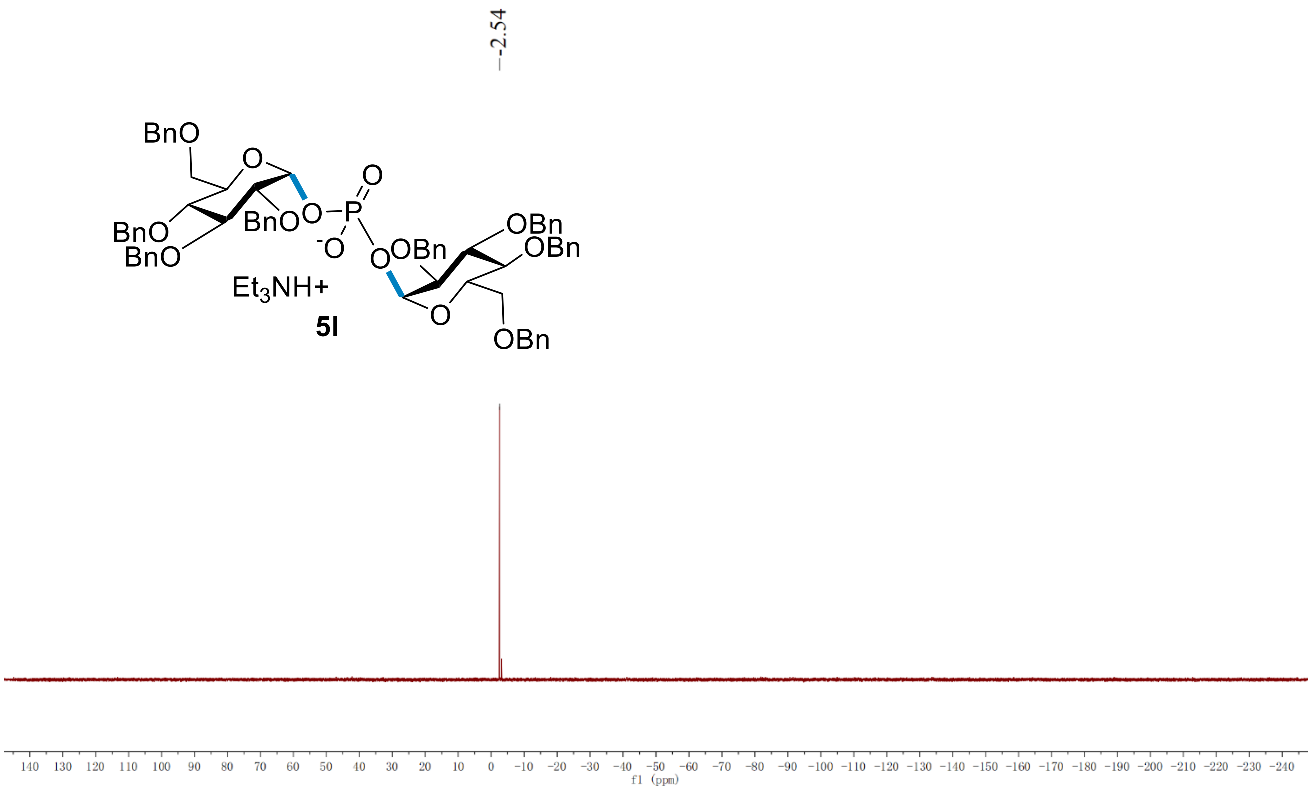

^{13}C NMR Spectrum of **5l** (151 MHz, $CDCl_3$)

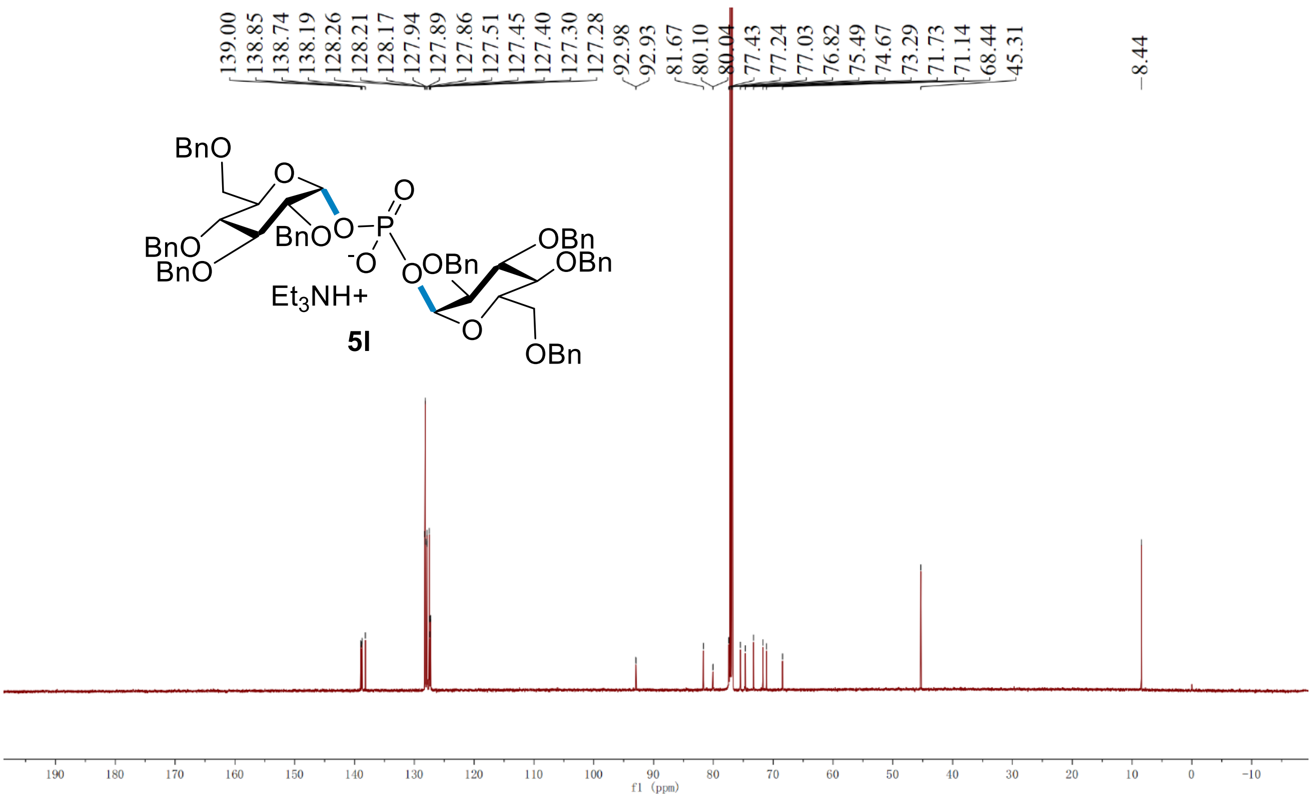

^{1}H NMR Spectrum of **5m** (400 MHz, $CDCl_3$)

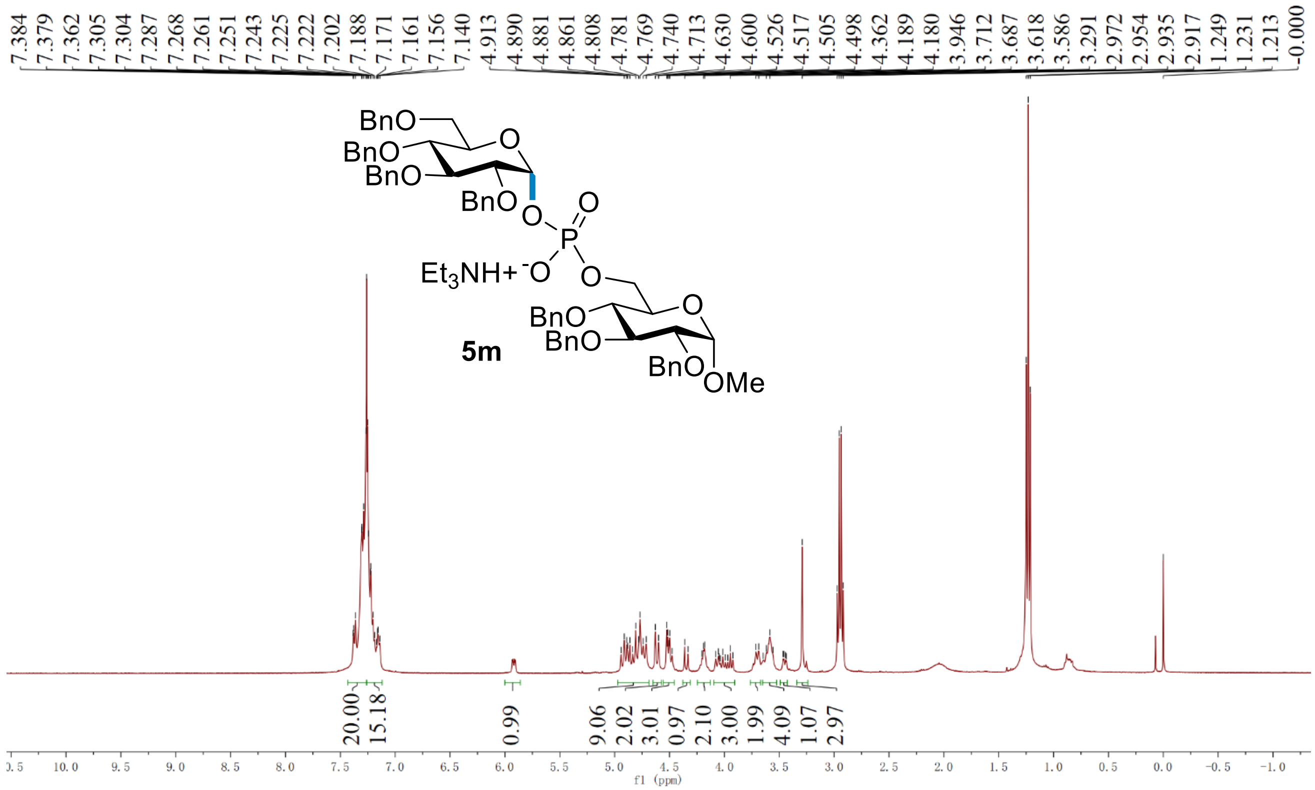

^{31}P NMR Spectrum of **5m** (162 MHz, $CDCl_3$)

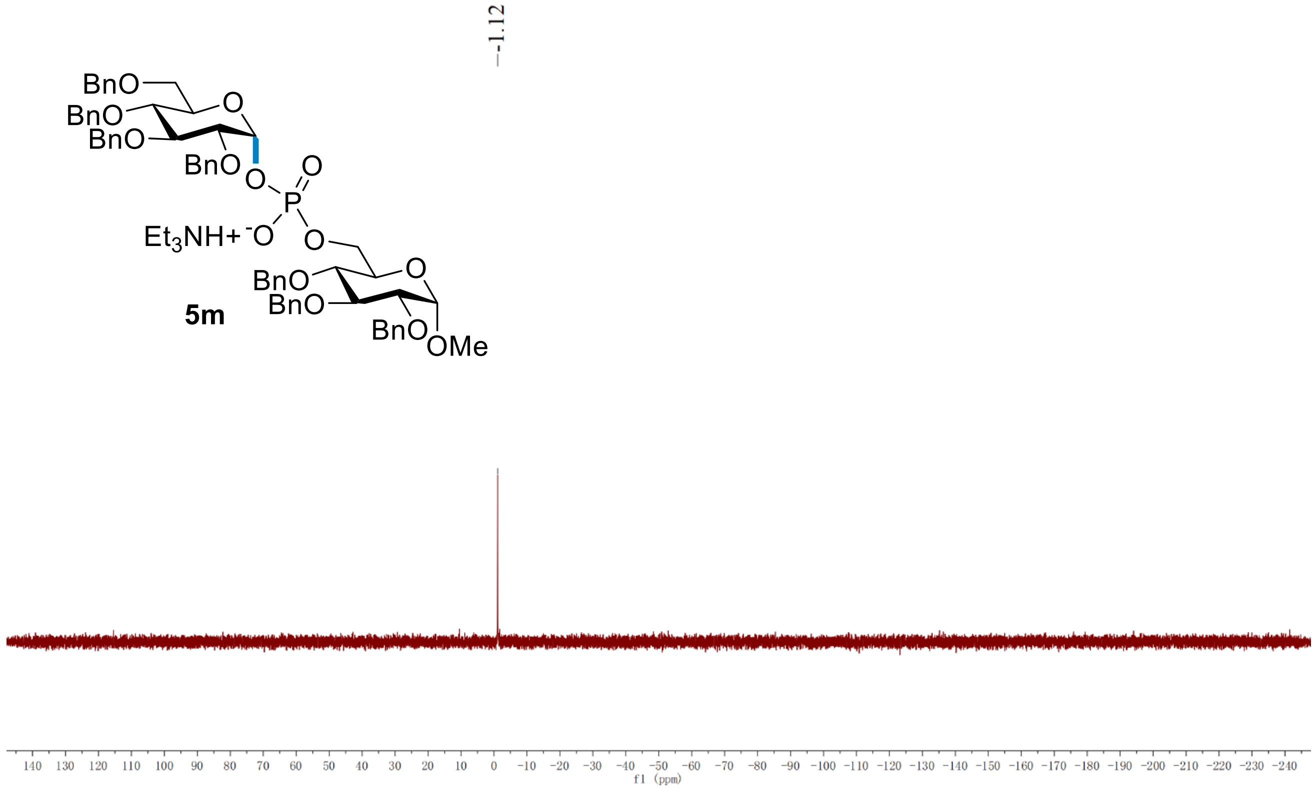

^{13}C NMR Spectrum of **5m** (101 MHz, $CDCl_3$)

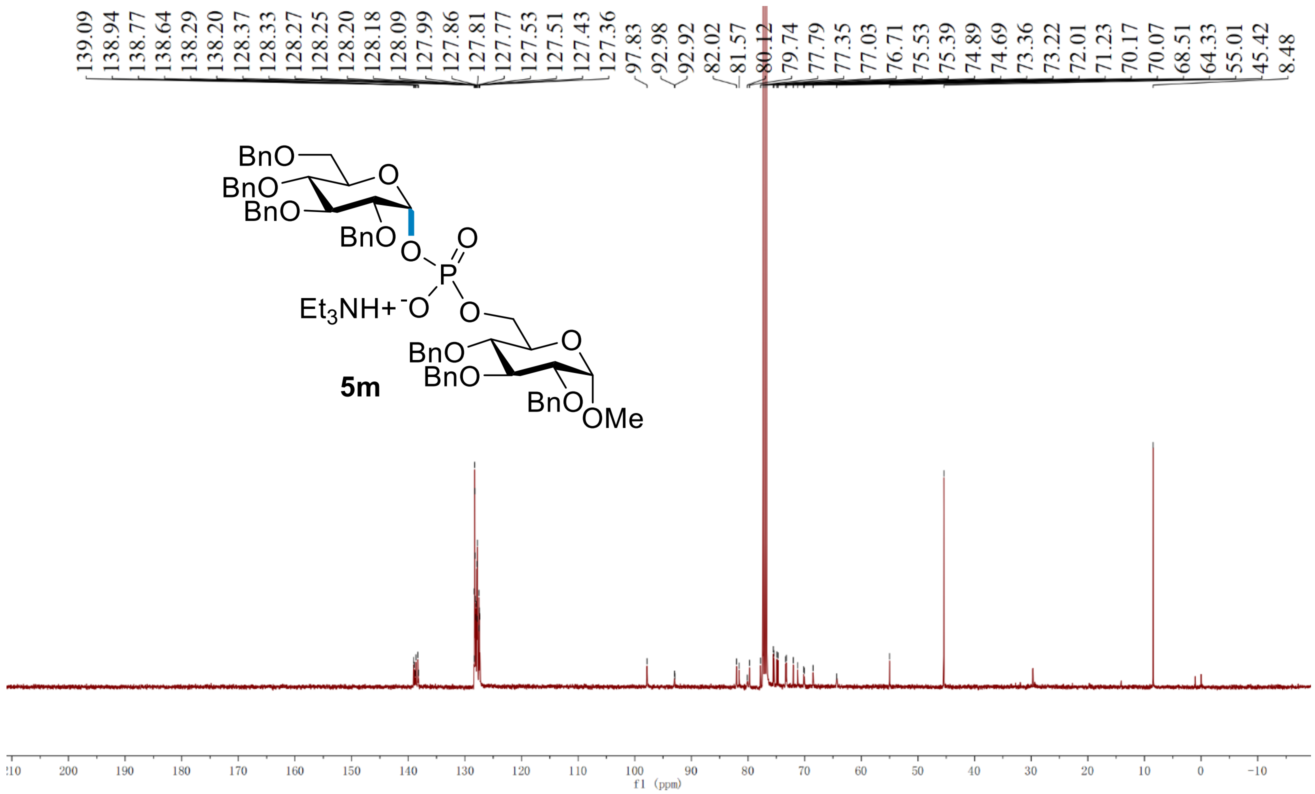

^{1}H NMR Spectrum of **5n** (400 MHz, $CDCl_3$)

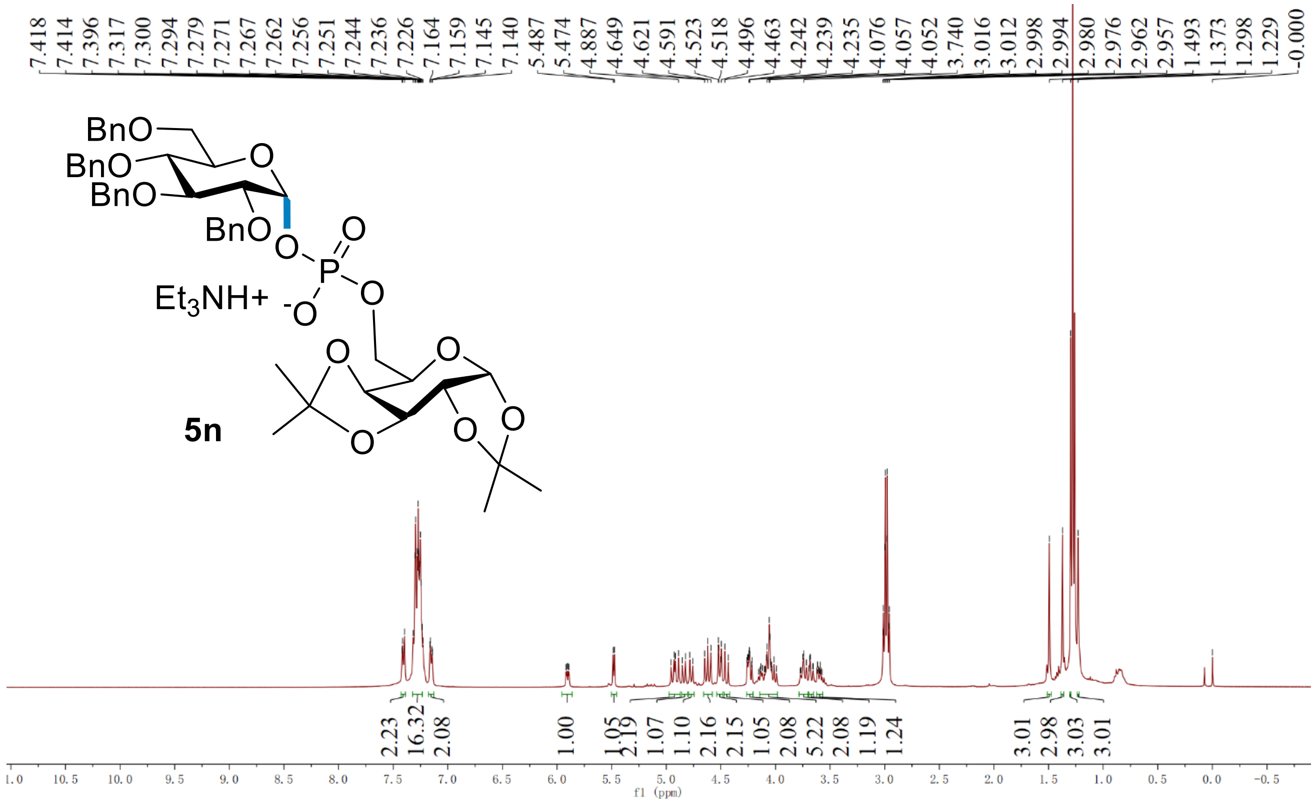

^{31}P NMR Spectrum of **5n** (162 MHz, $CDCl_3$)

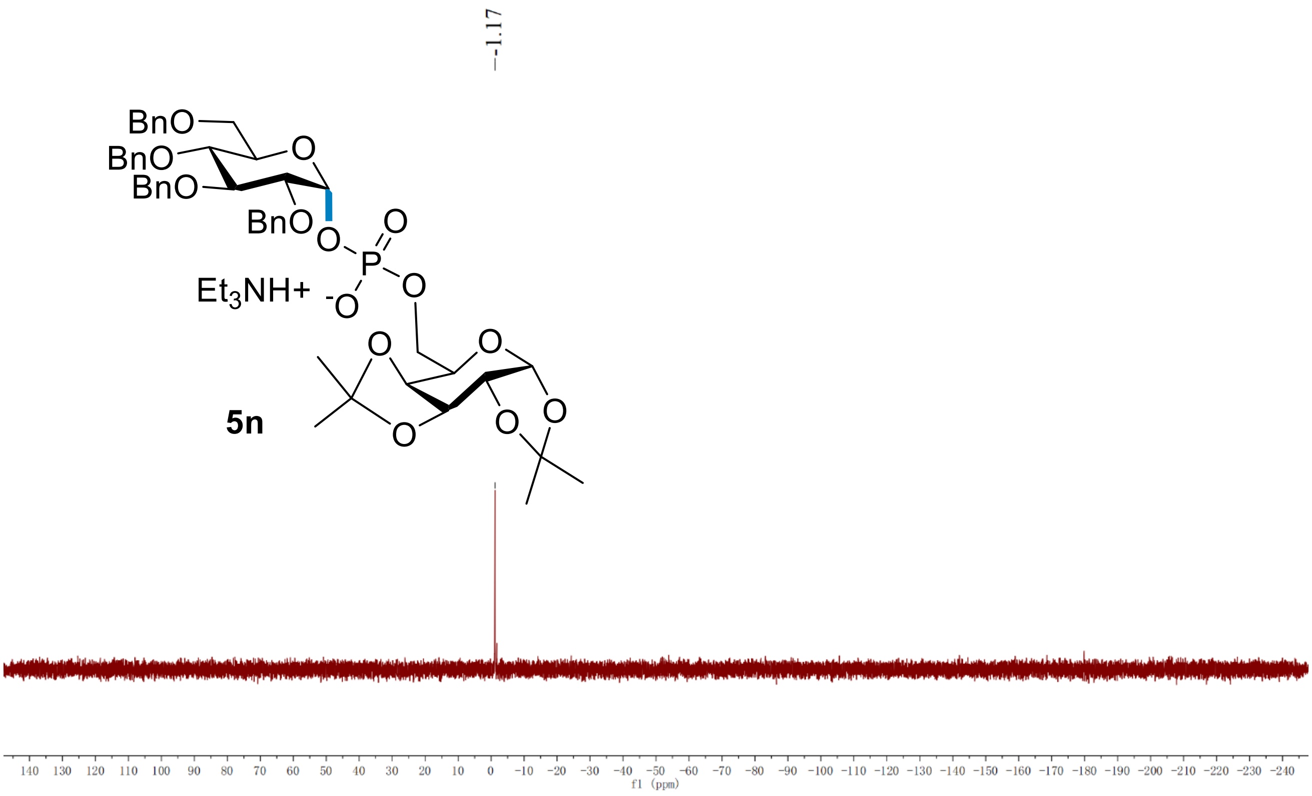

^{1}HNMR Spectrum of **5o** (400 MHz, $CDCl_3$)

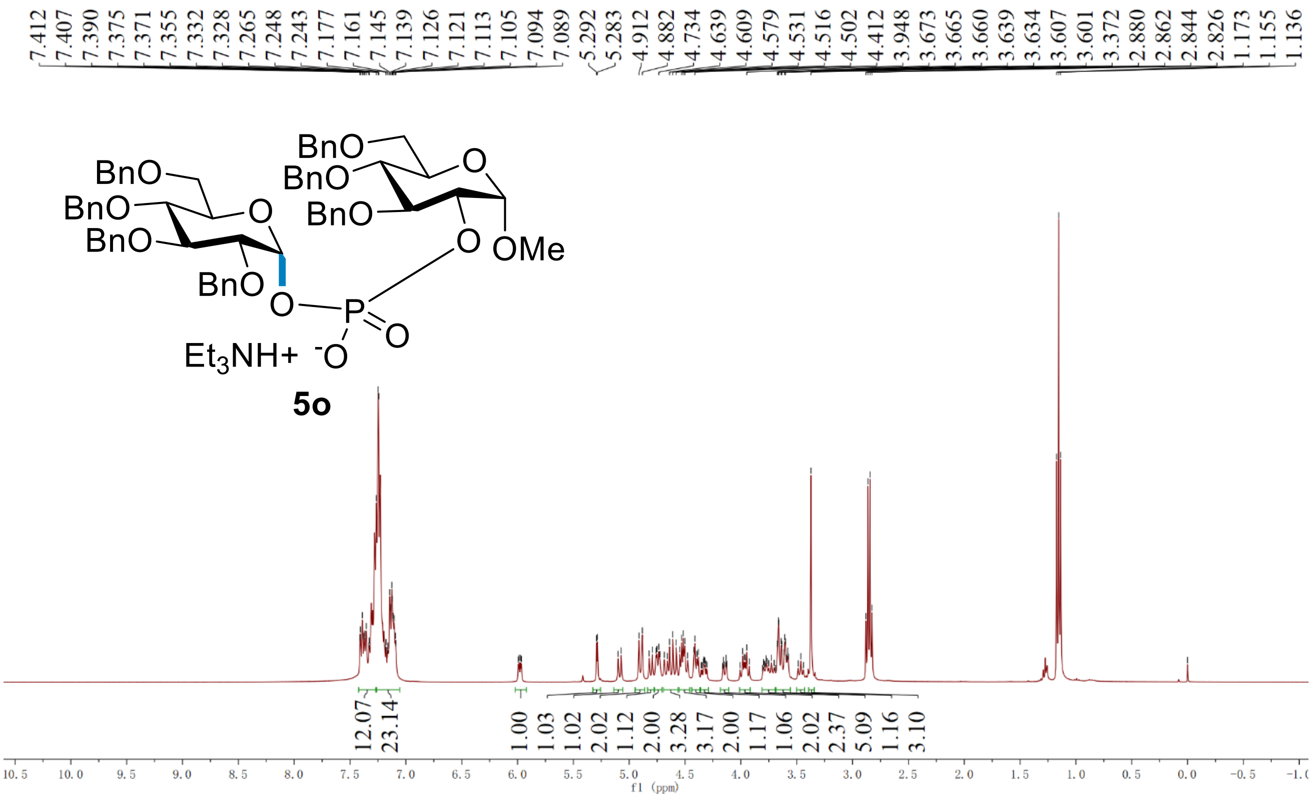

^{31}P NMR Spectrum of **5o** (162 MHz, $CDCl_3$)

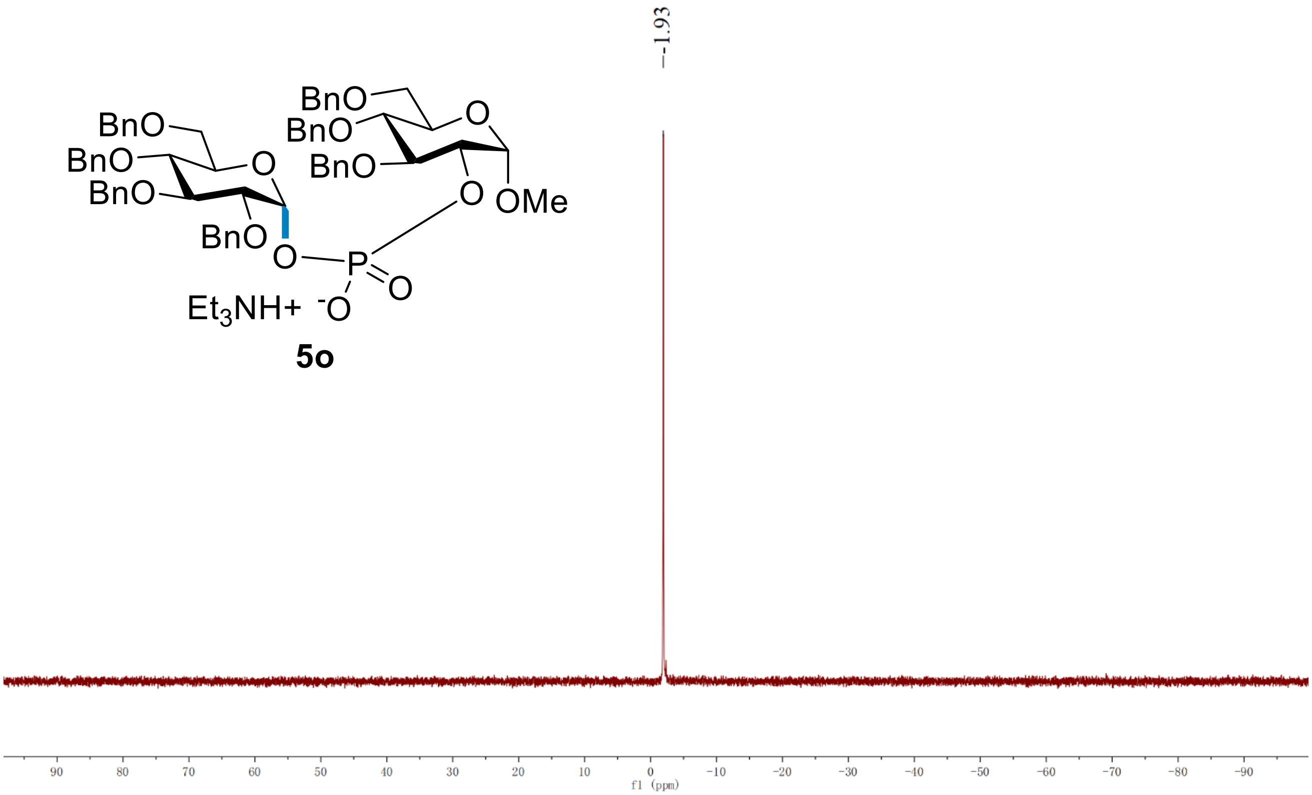

^{13}C NMR Spectrum of **5o** (101 MHz, $CDCl_3$)

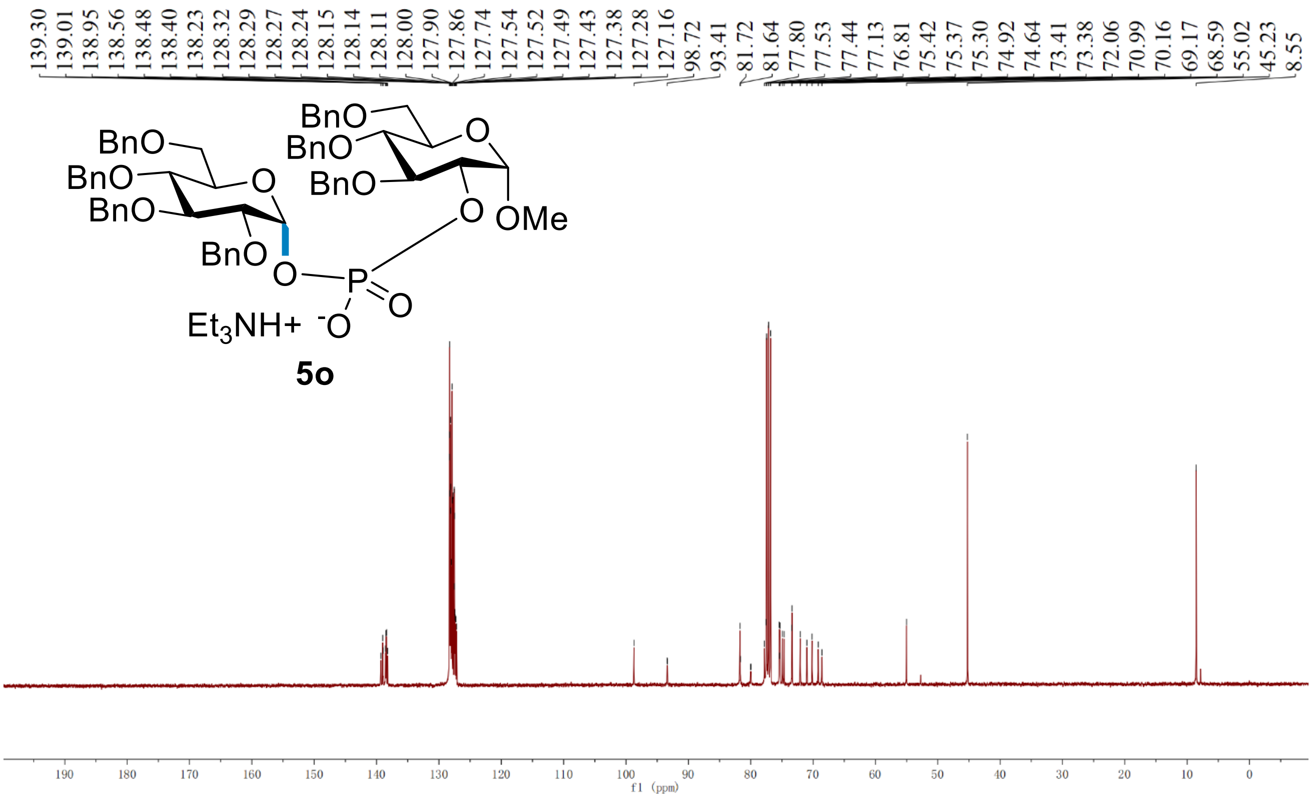

^{1}H NMR Spectrum of **5p** (400 MHz, $CDCl_3$)

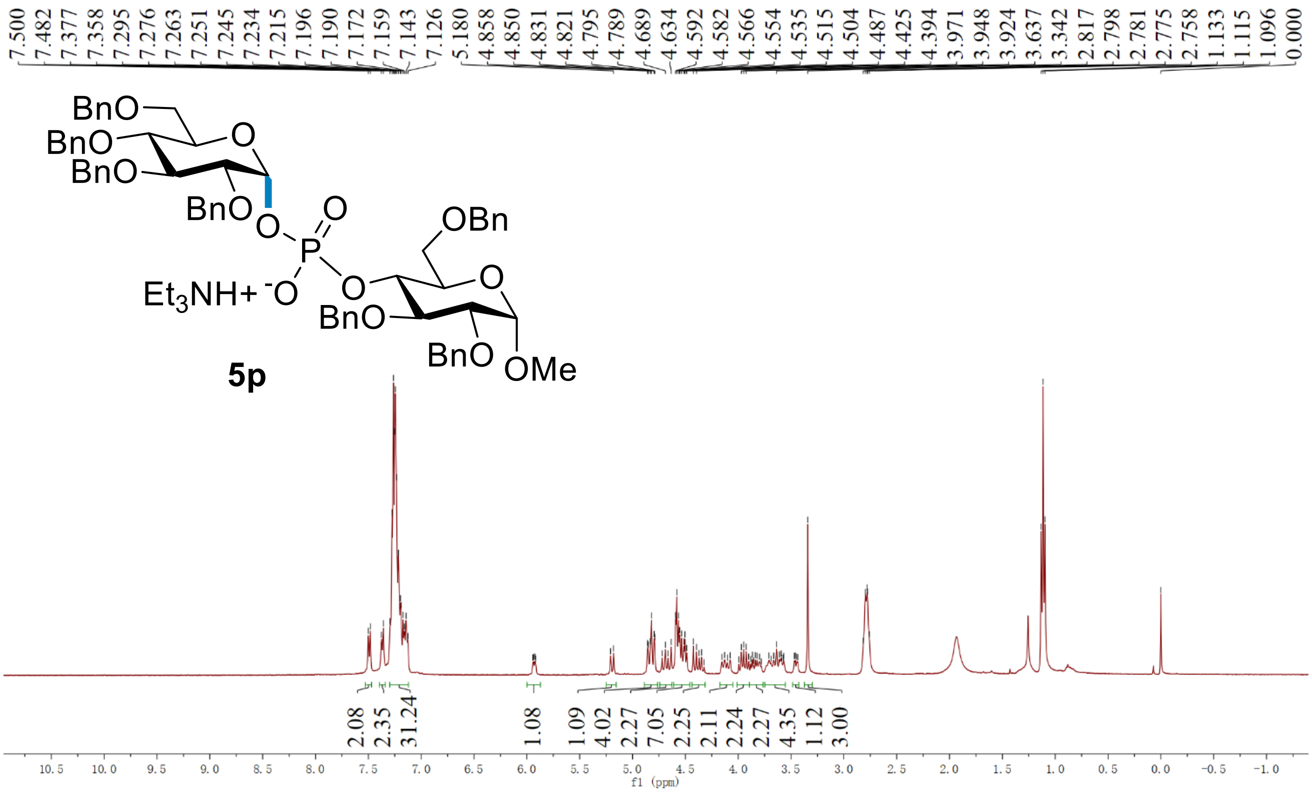

^{31}P NMR Spectrum of **5p** (162 MHz, $CDCl_3$)

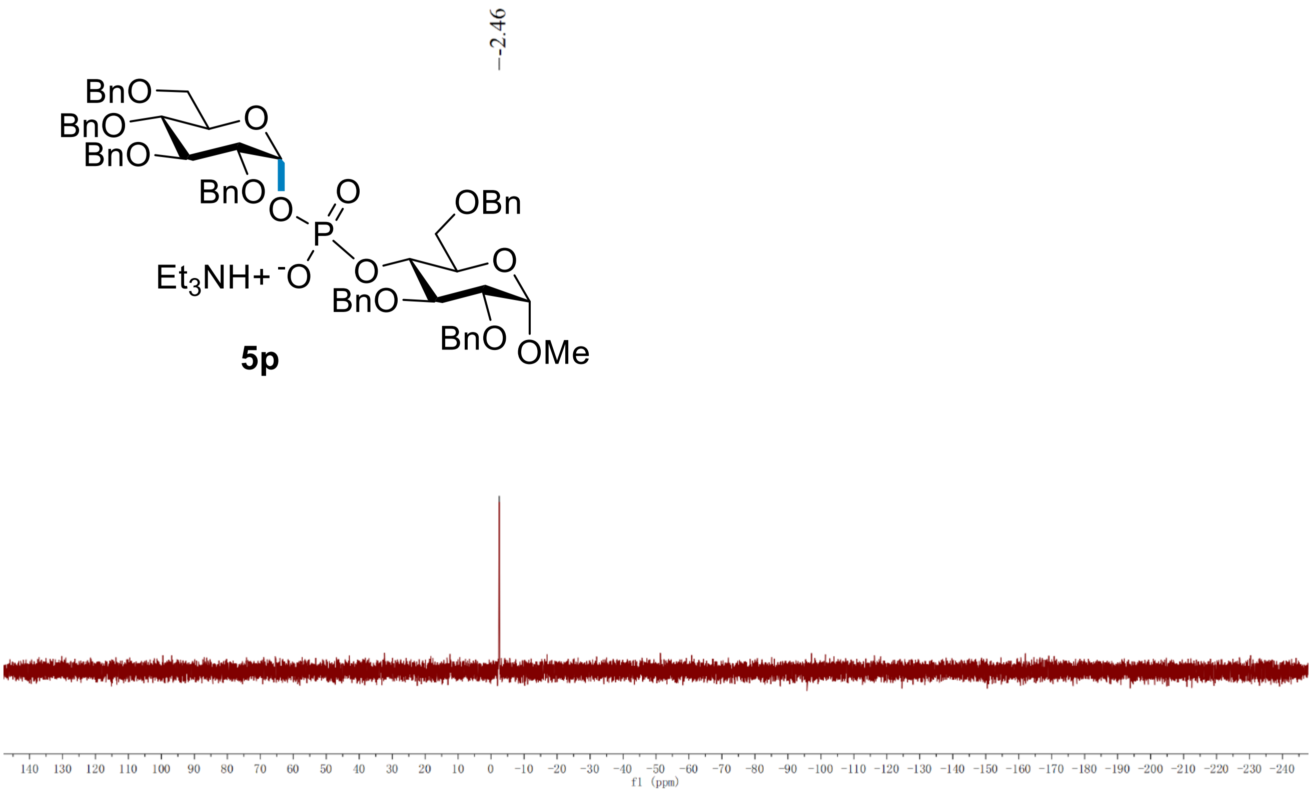

^{1}H NMR Spectrum of **5q** (400 MHz, $CDCl_3$)

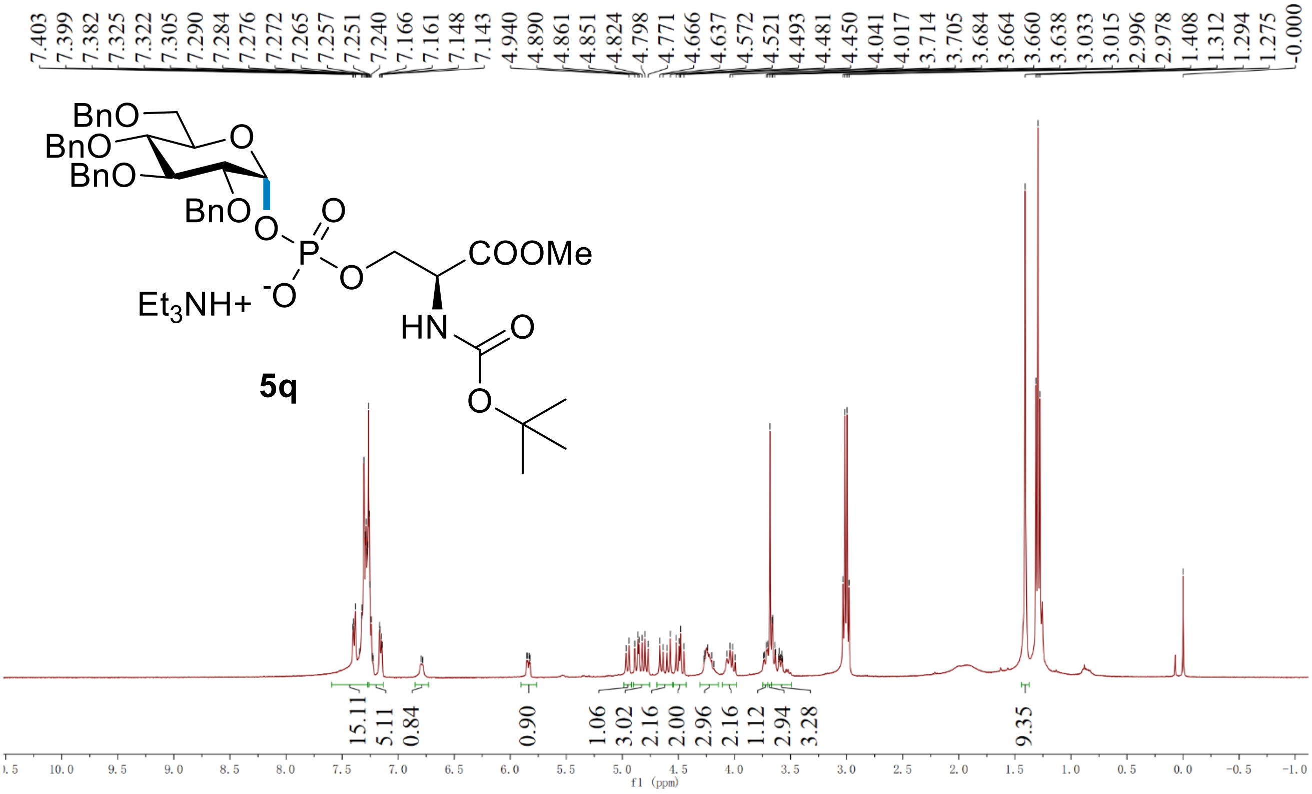

^{31}P NMR Spectrum of **5q** (162 MHz, $CDCl_3$)

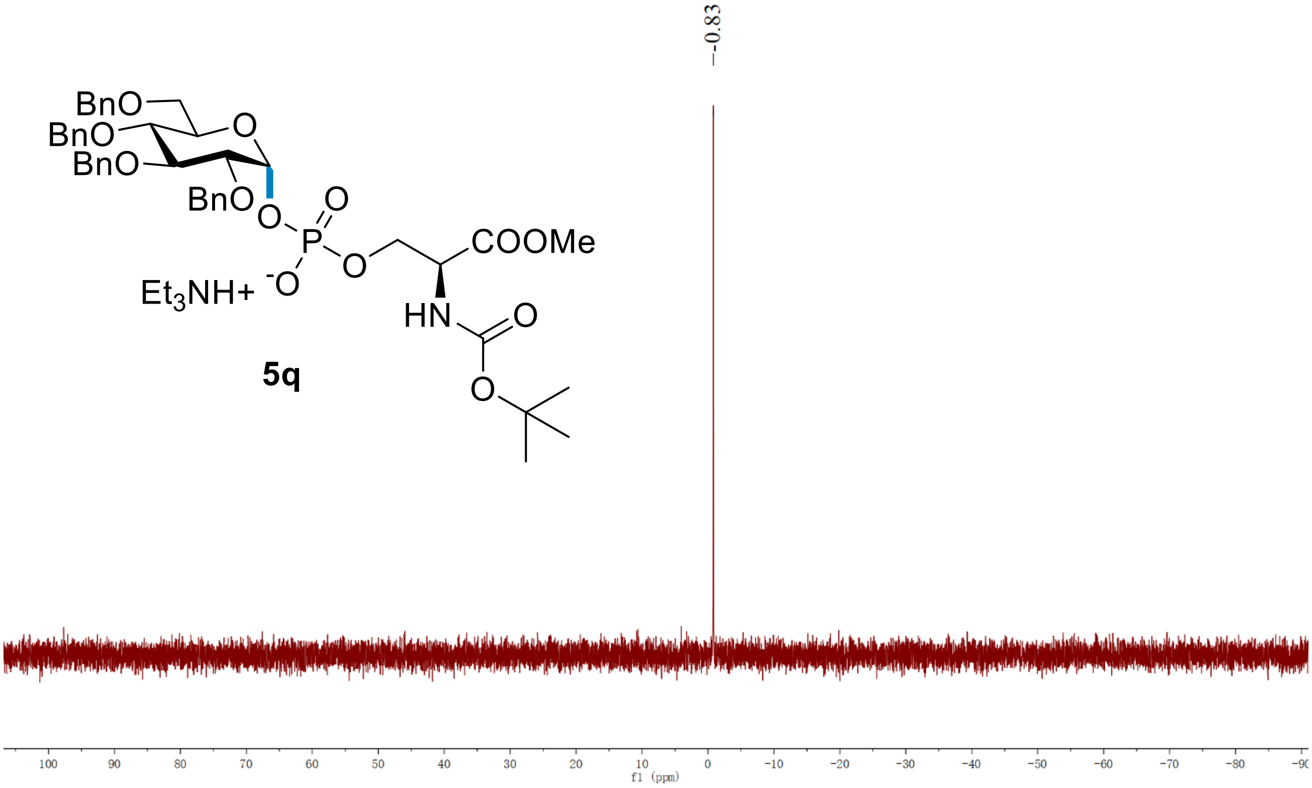

^{13}C NMR Spectrum of **5q** (101 MHz, $CDCl_3$)

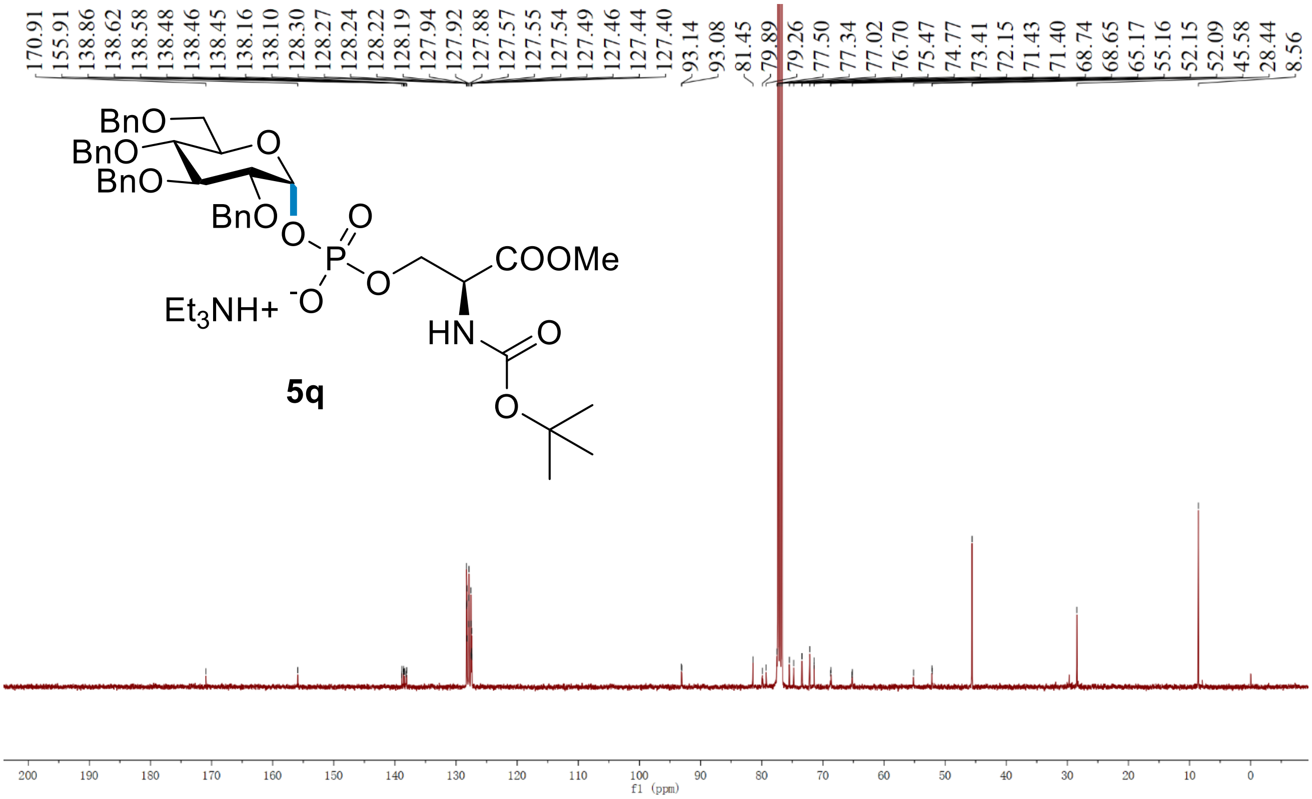

^{1}H NMR Spectrum of **5r** (400 MHz, CD_3OD)

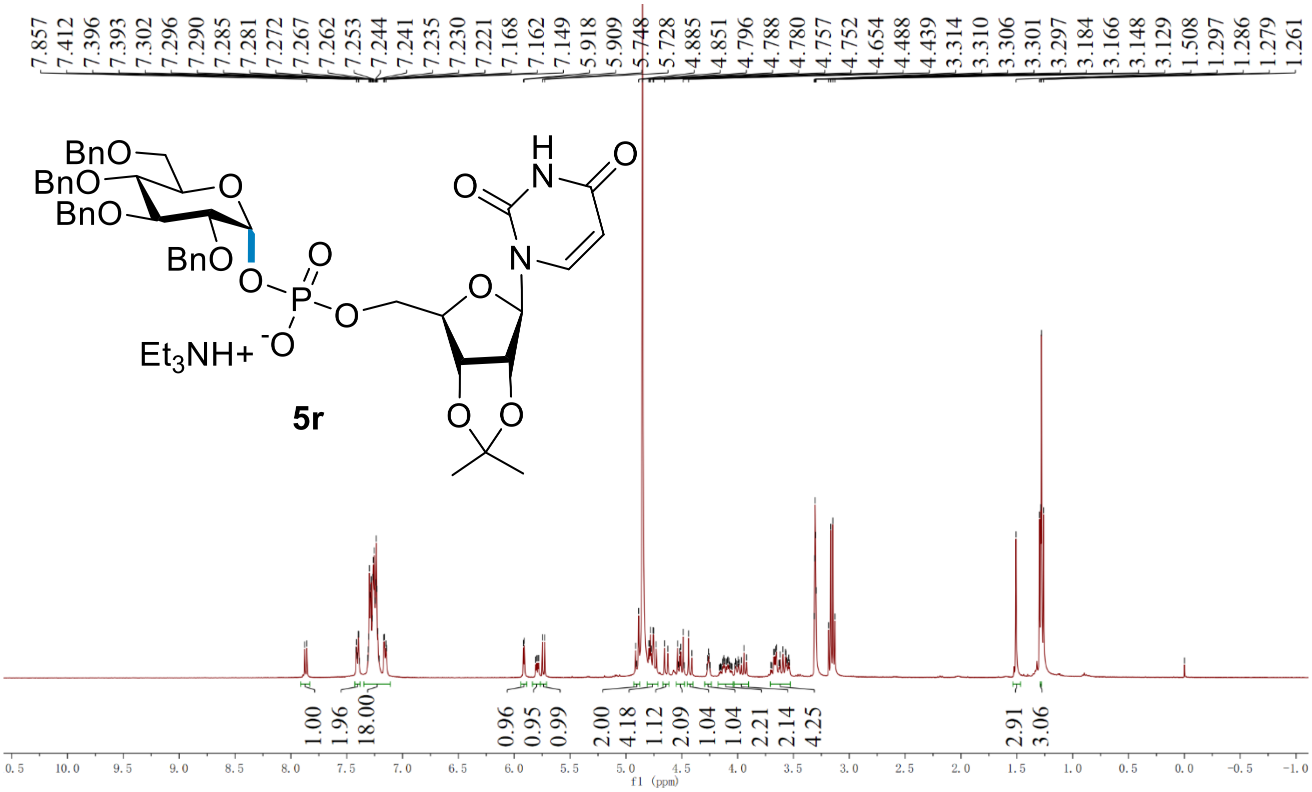

^{31}P NMR Spectrum of **5r** (162 MHz, CD_3OD)

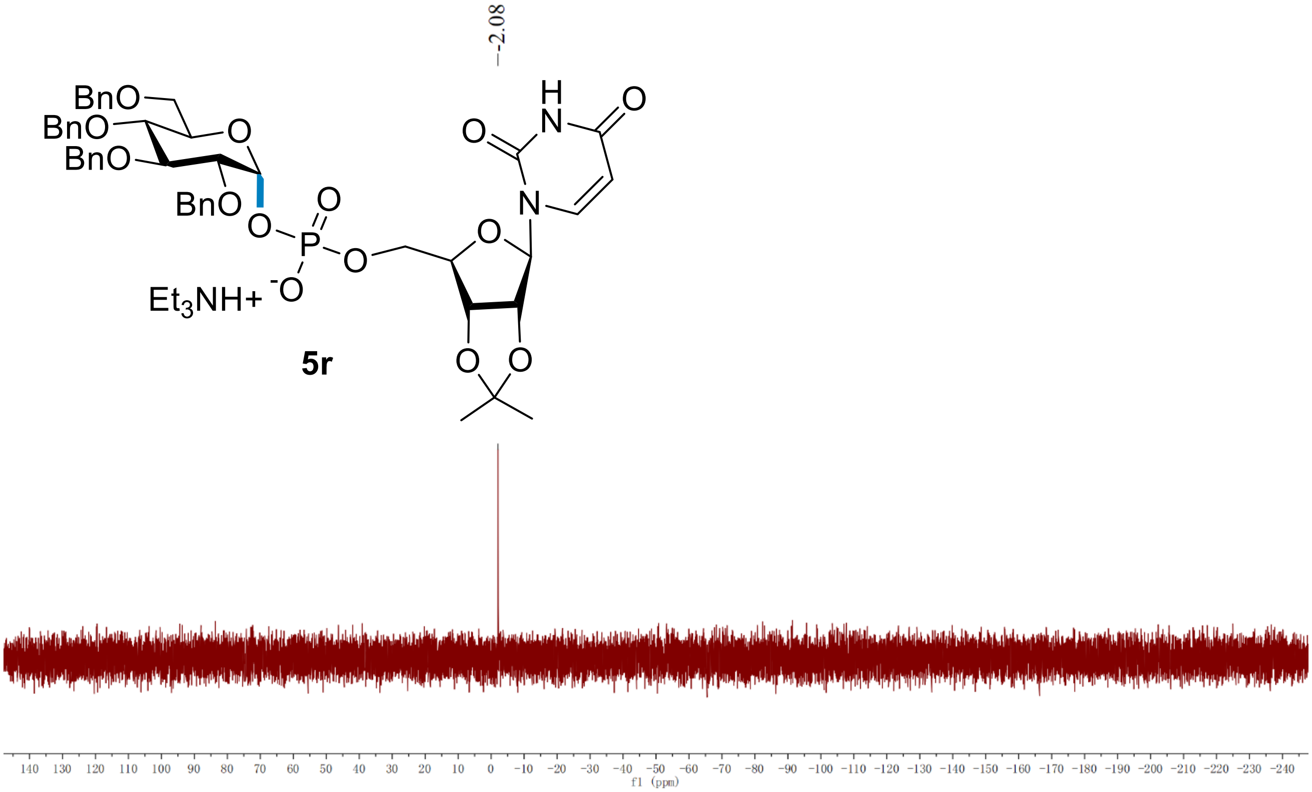

^{13}C NMR Spectrum of **5r** (101 MHz, CD_3OD)

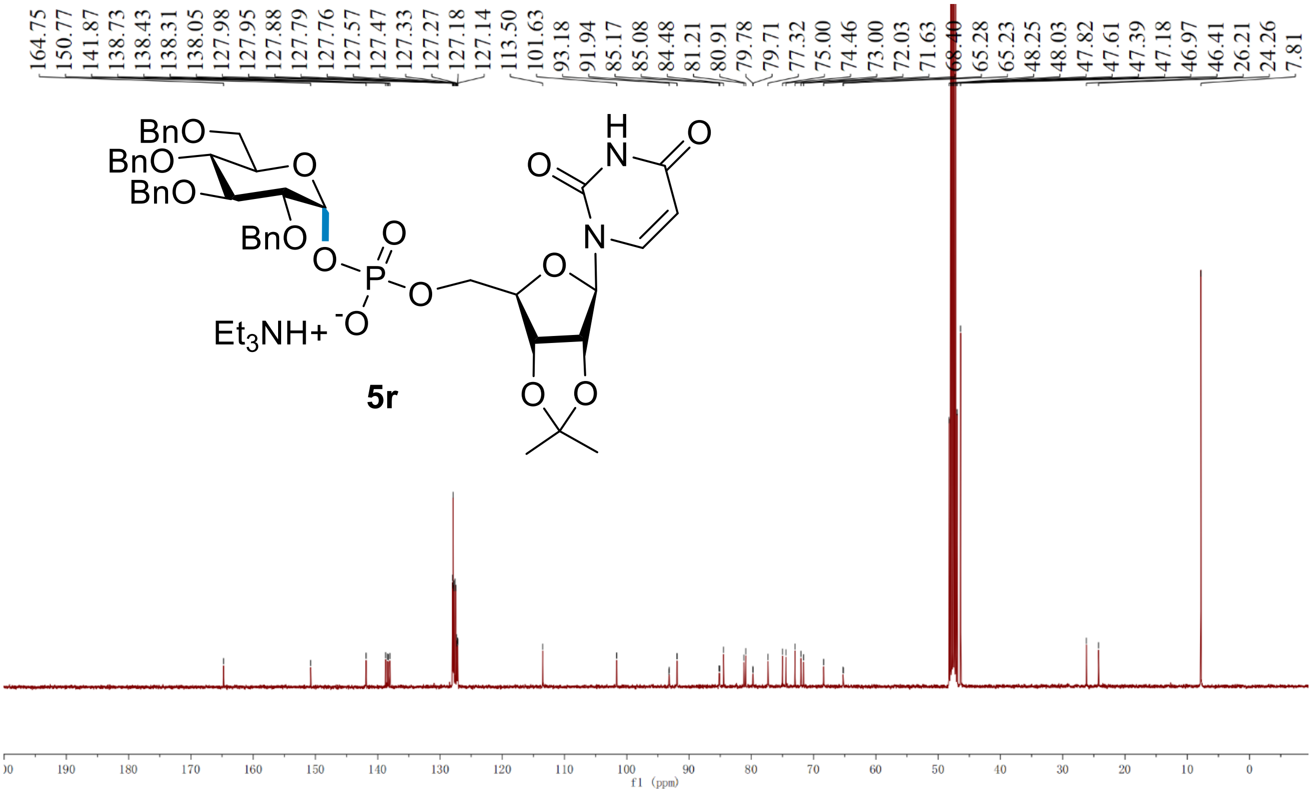

^{1}H NMR Spectrum of **6a** (400 MHz, $CDCl_3$)

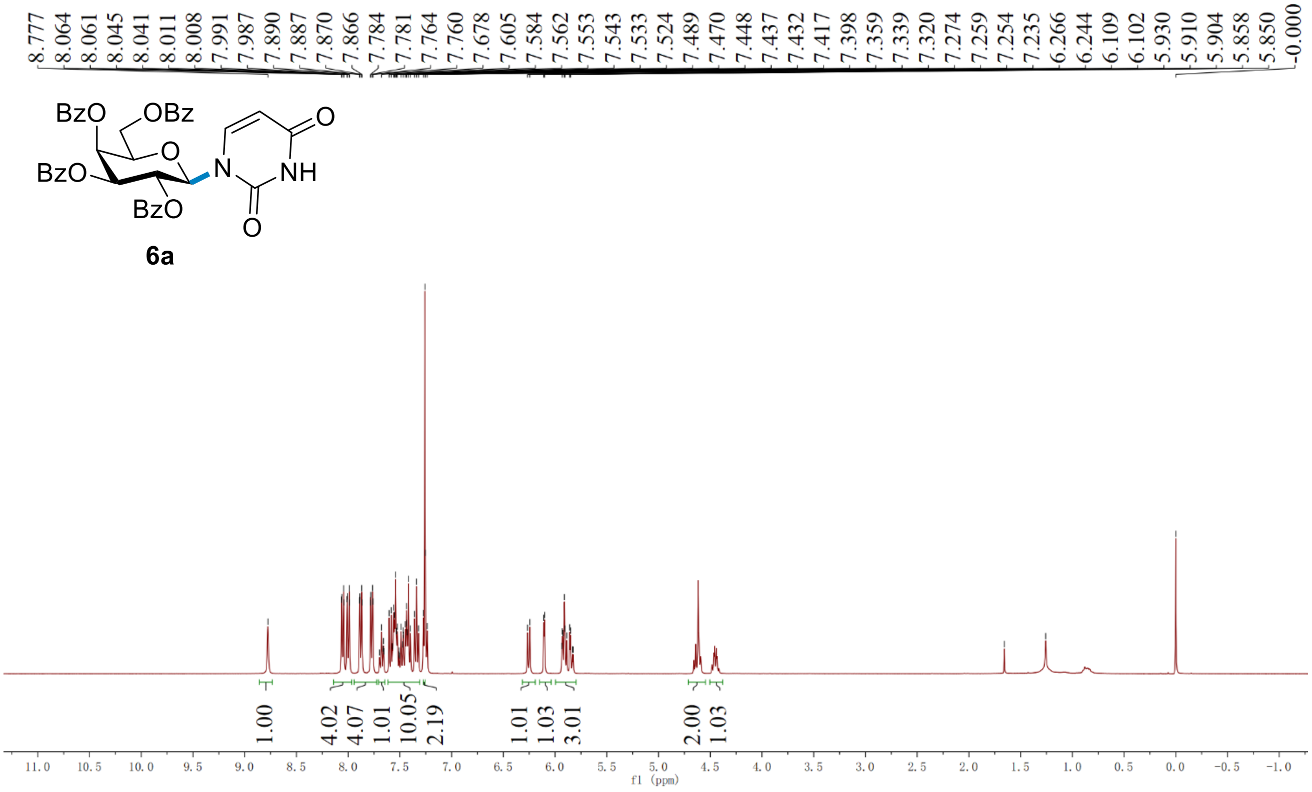

^{1}H NMR Spectrum of **6b** (400 MHz, $CDCl_3$)

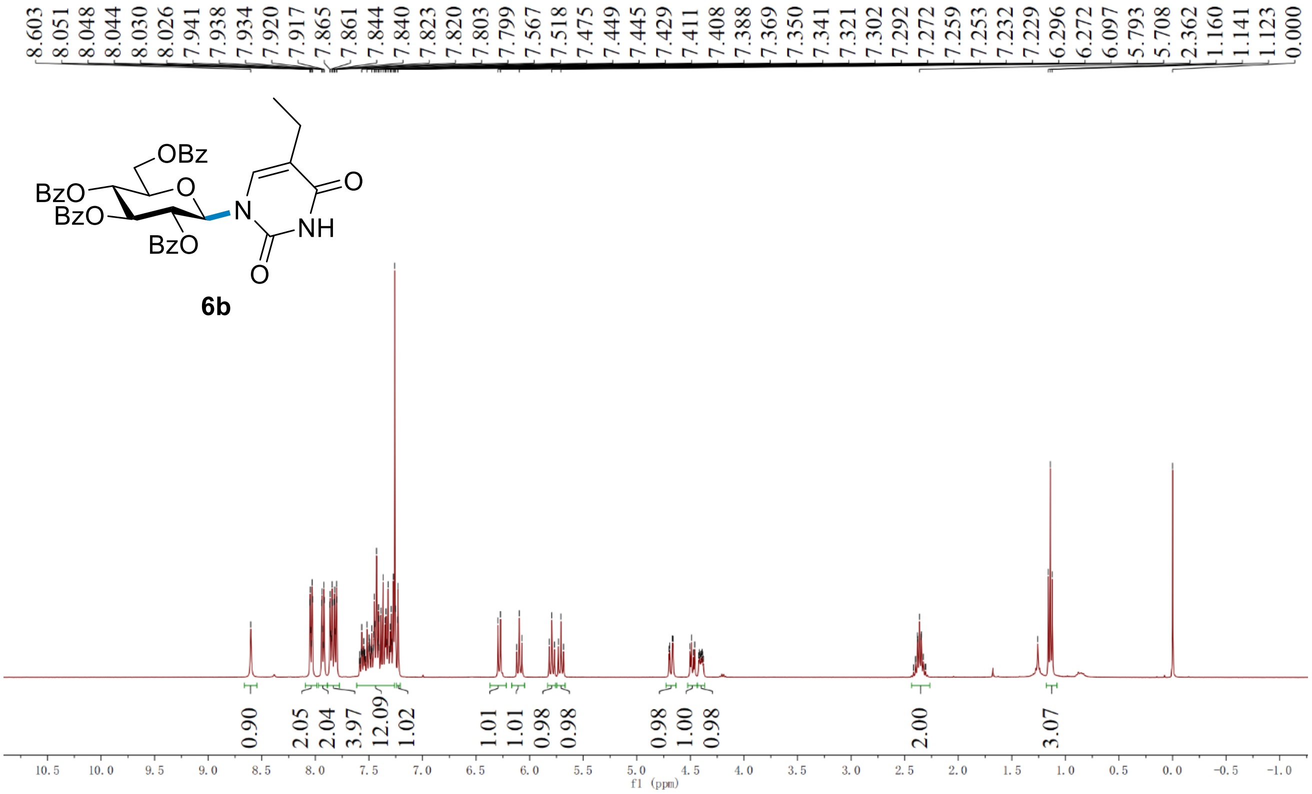

^{13}C NMR Spectrum of **6b** (101 MHz, $CDCl_3$)

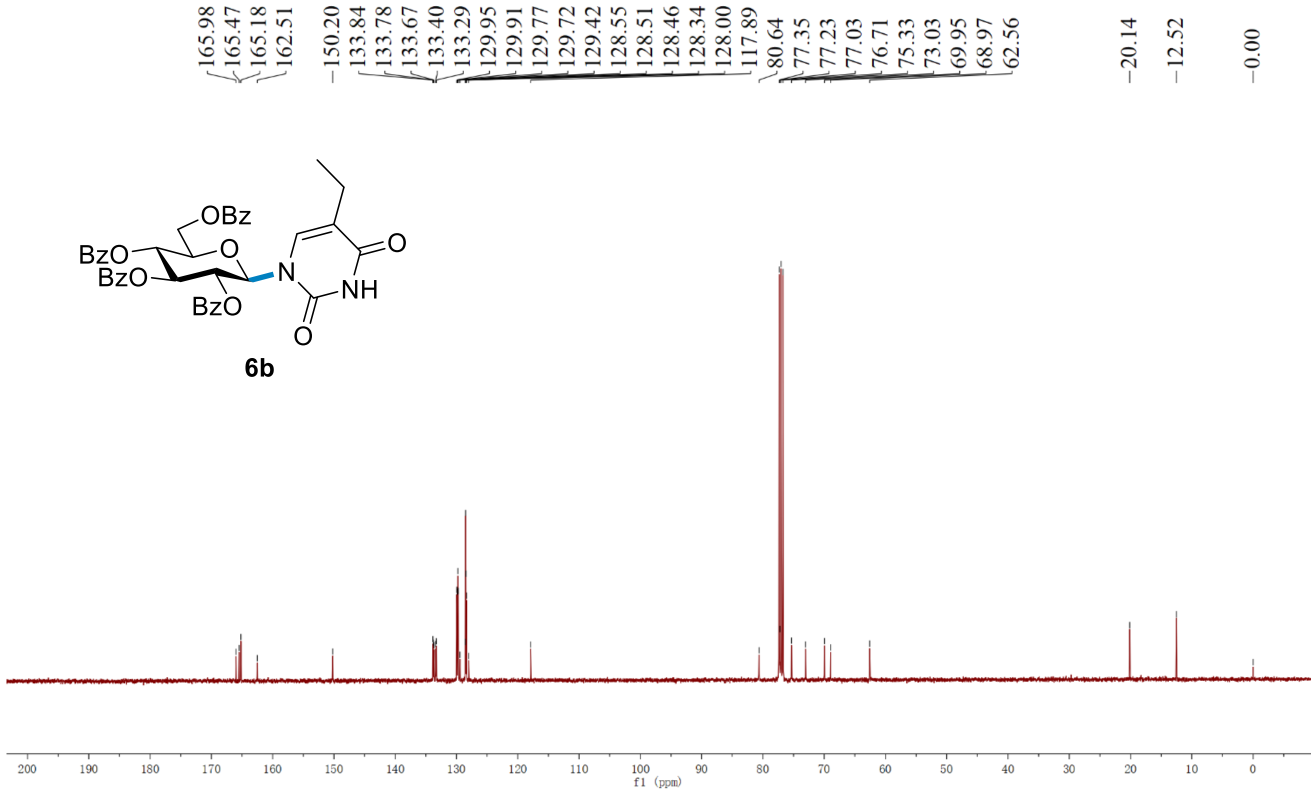

^{1}H NMR Spectrum of **6c** (400 MHz, $CDCl_3$)

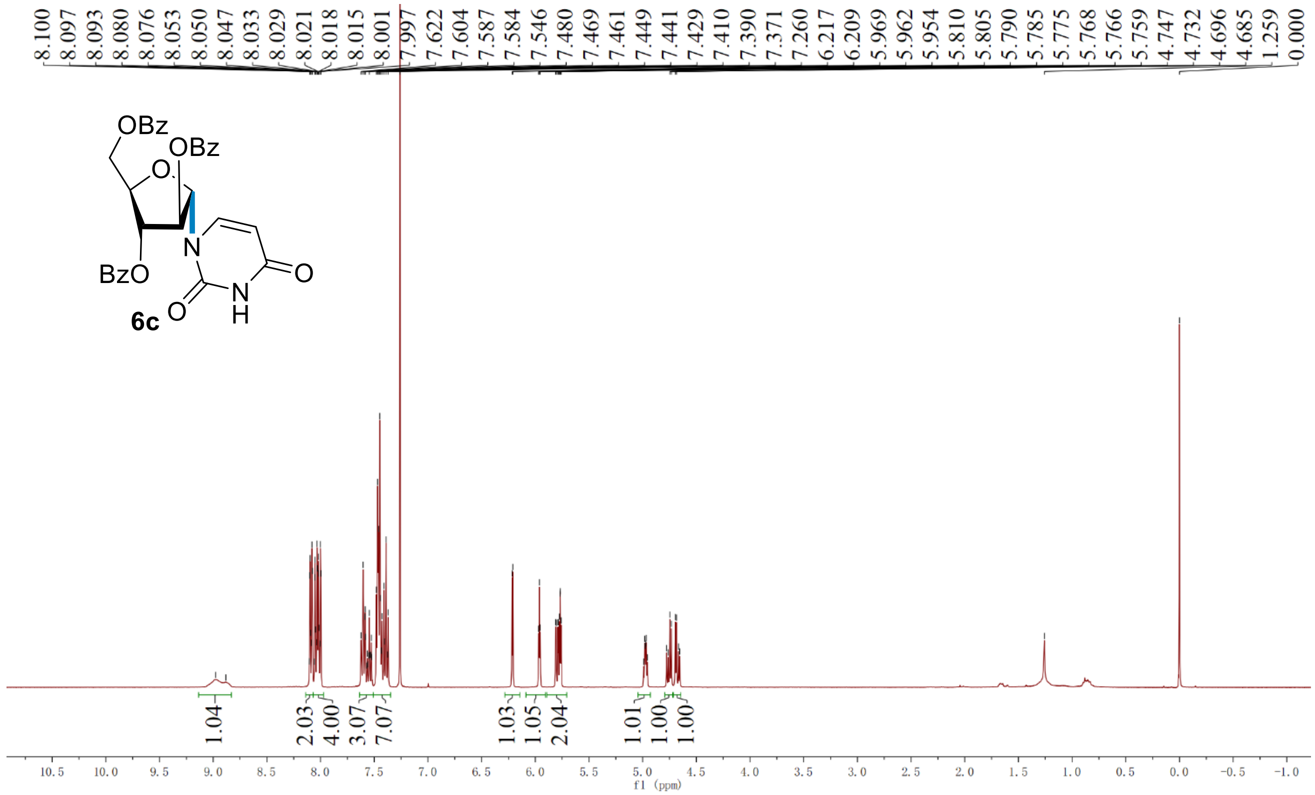

^{13}C NMR Spectrum of **6c** (101 MHz, $CDCl_3$)

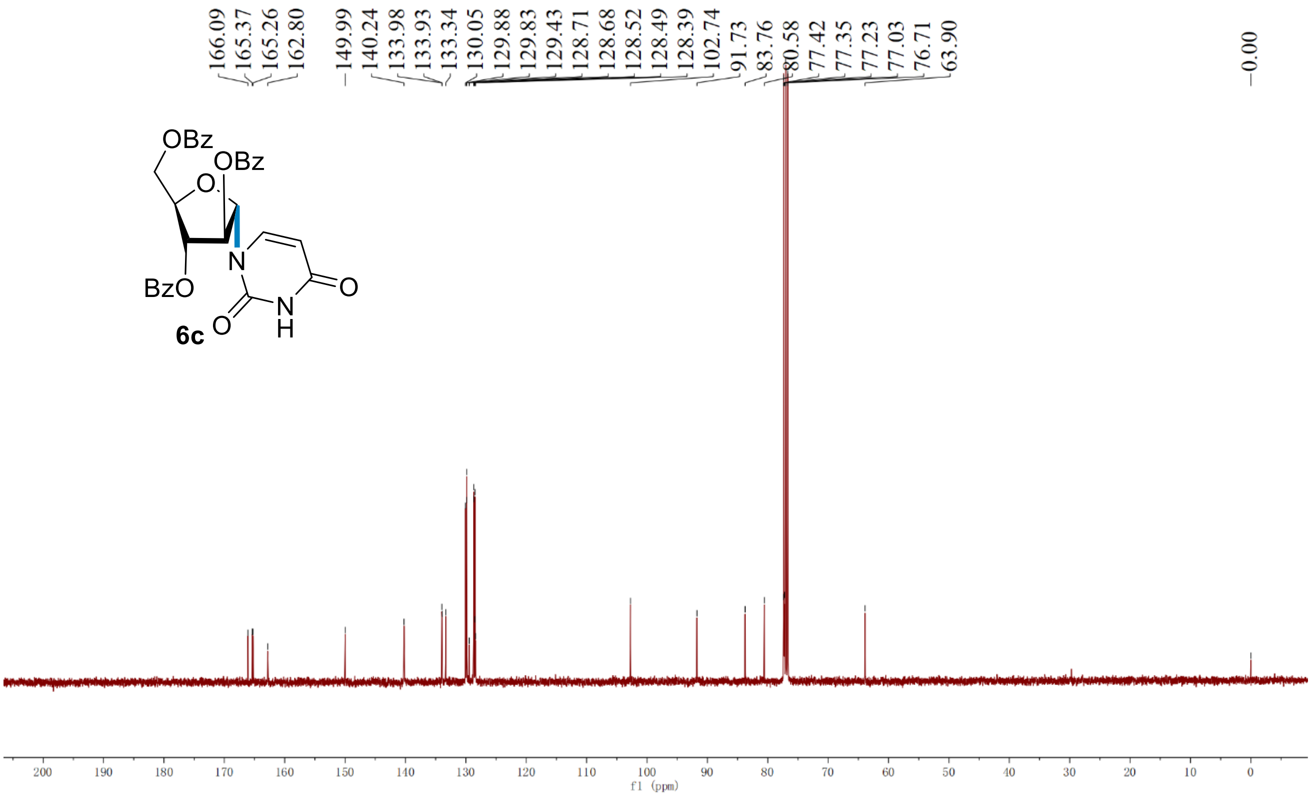

^{1}H NMR Spectrum of **6d** (400 MHz, $CDCl_3$)

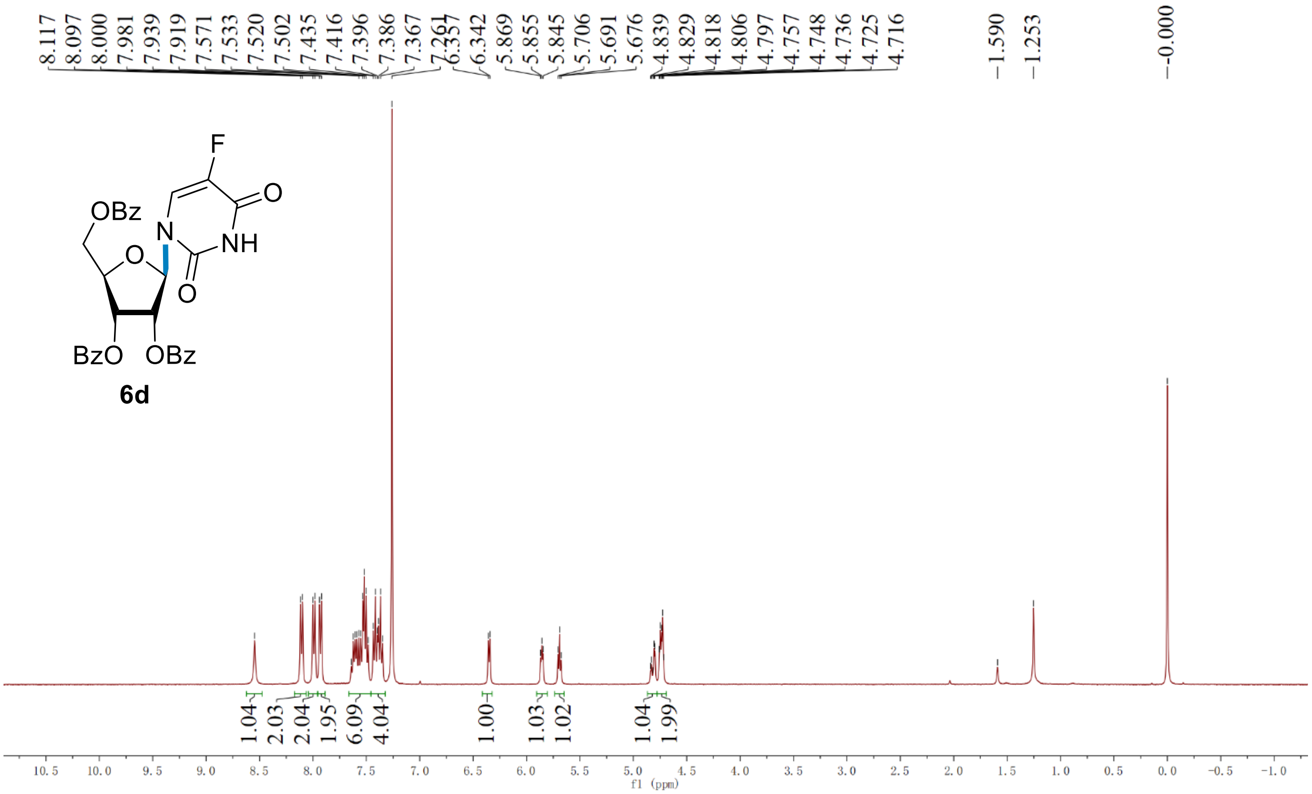

^{19}F NMR Spectrum of **6d** (376 MHz, $CDCl_3$)

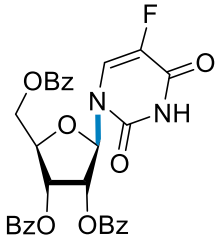

6d

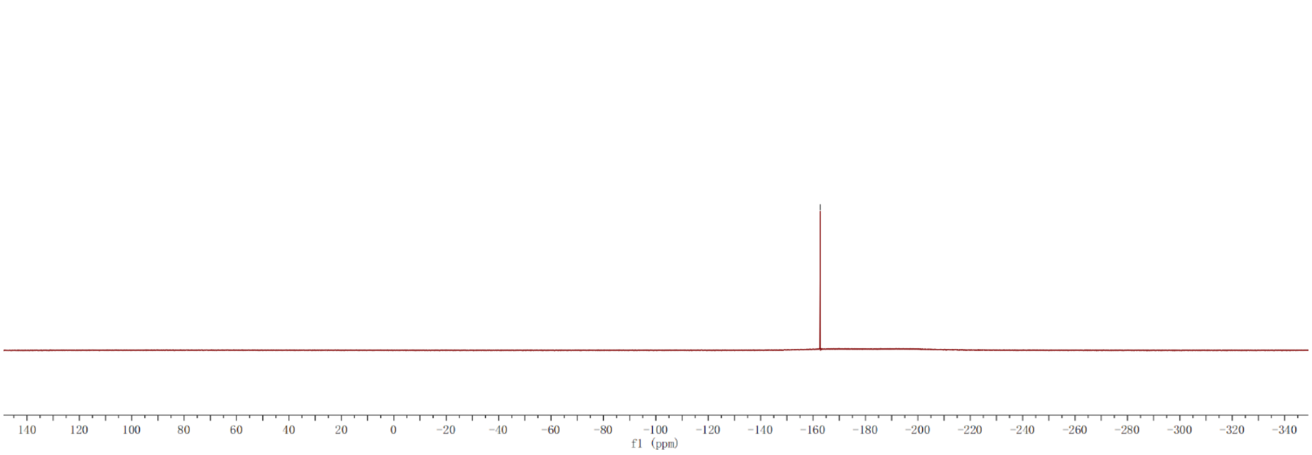

^{1}H NMR Spectrum of **6e** (400 MHz, $CDCl_3$)

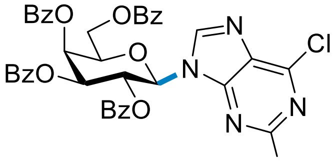

6e

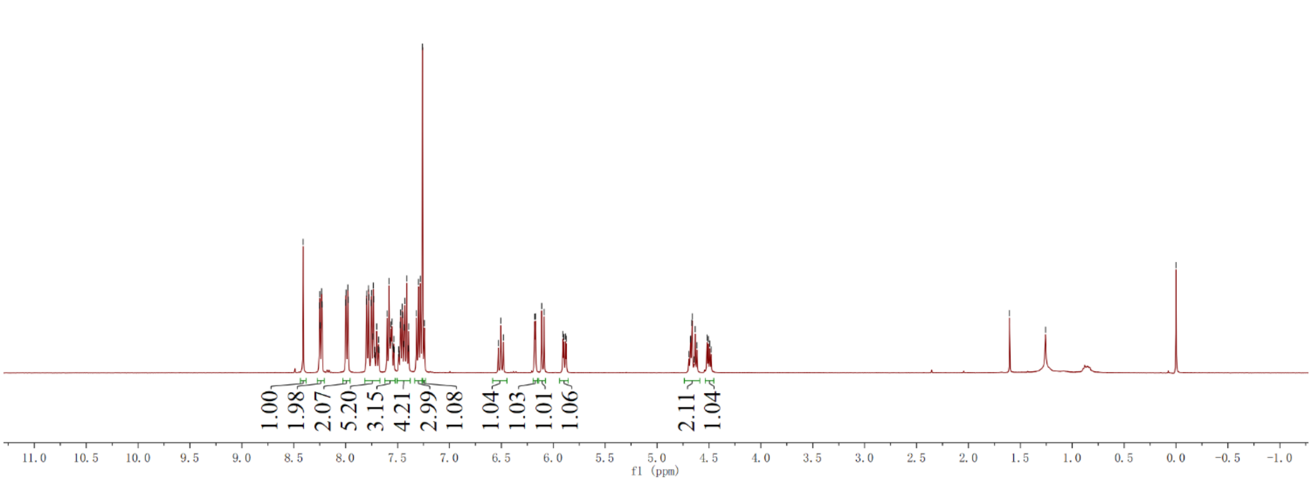

^{13}C NMR Spectrum of **6e** (101 MHz, $CDCl_3$)

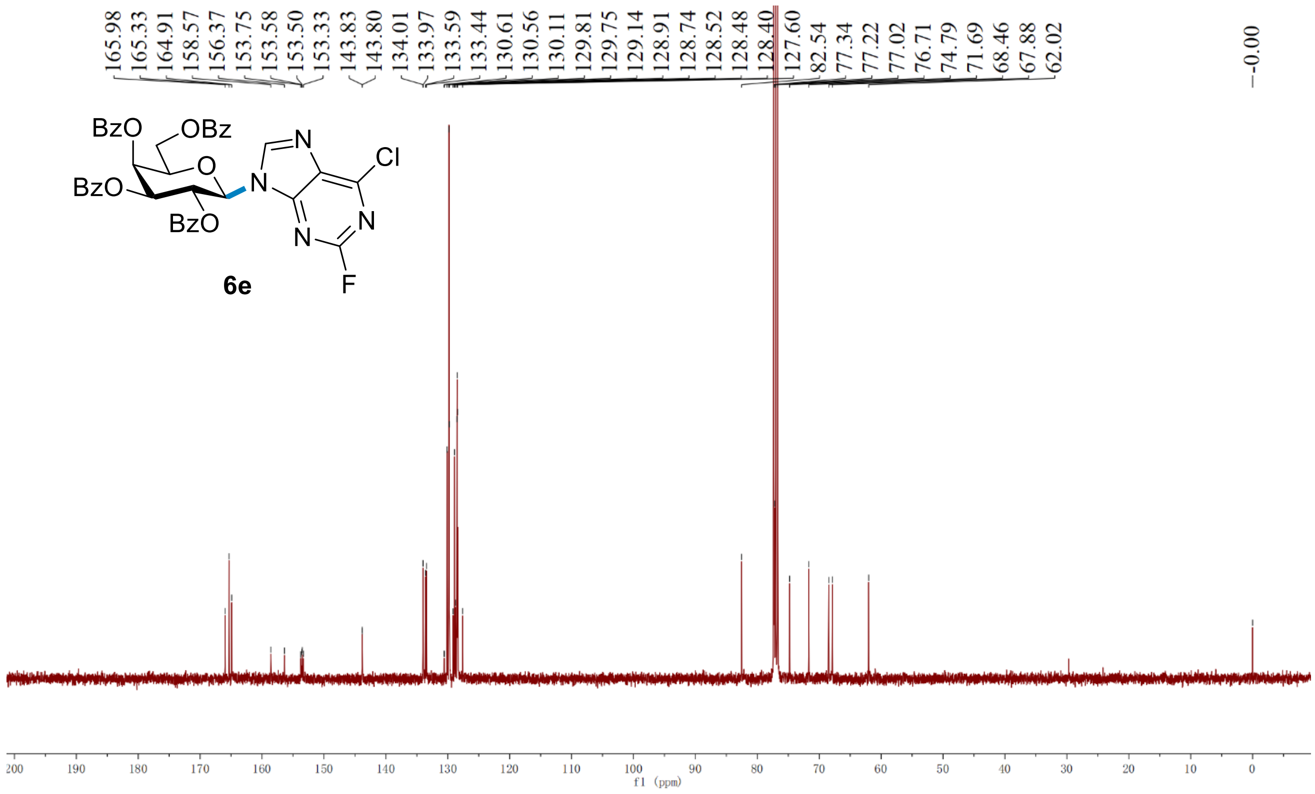

^{19}F NMR Spectrum of **6e** (376 MHz, $CDCl_3$)

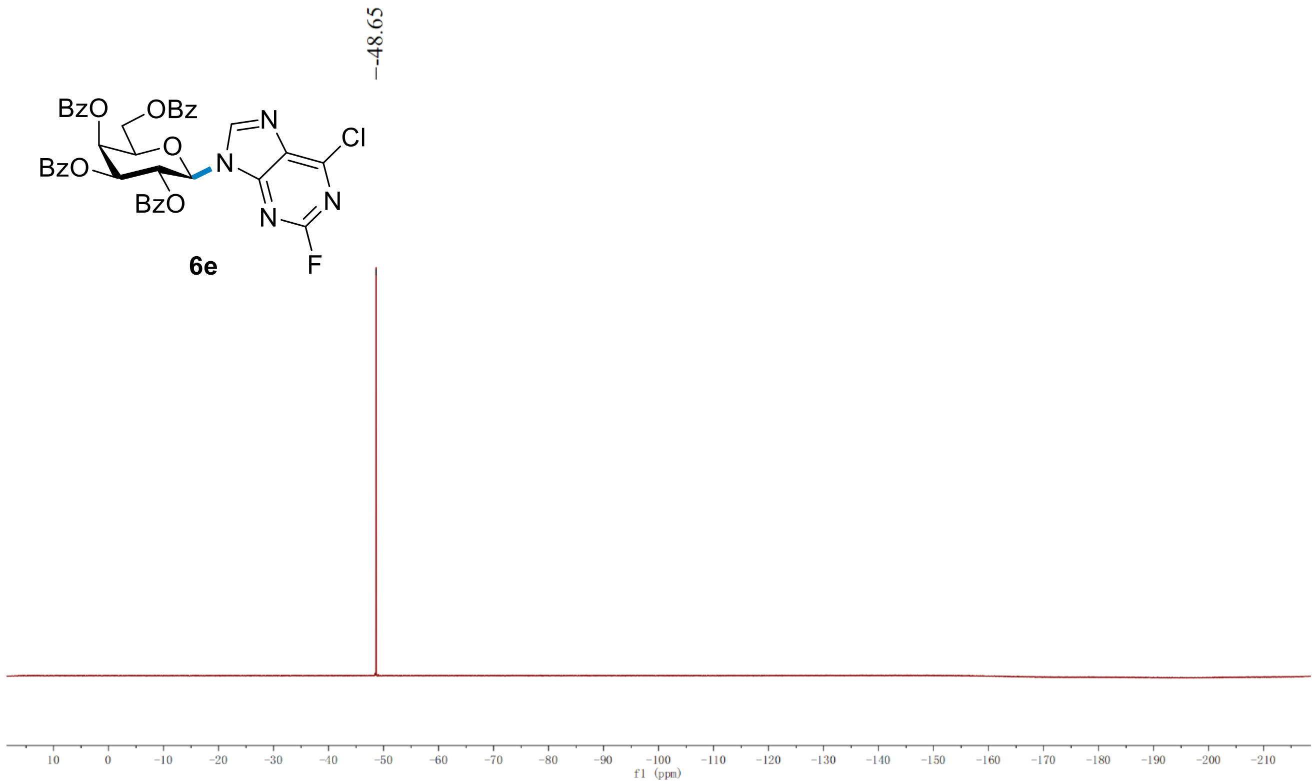

^{1}H NMR Spectrum of **6f** (400 MHz, $CDCl_3$)

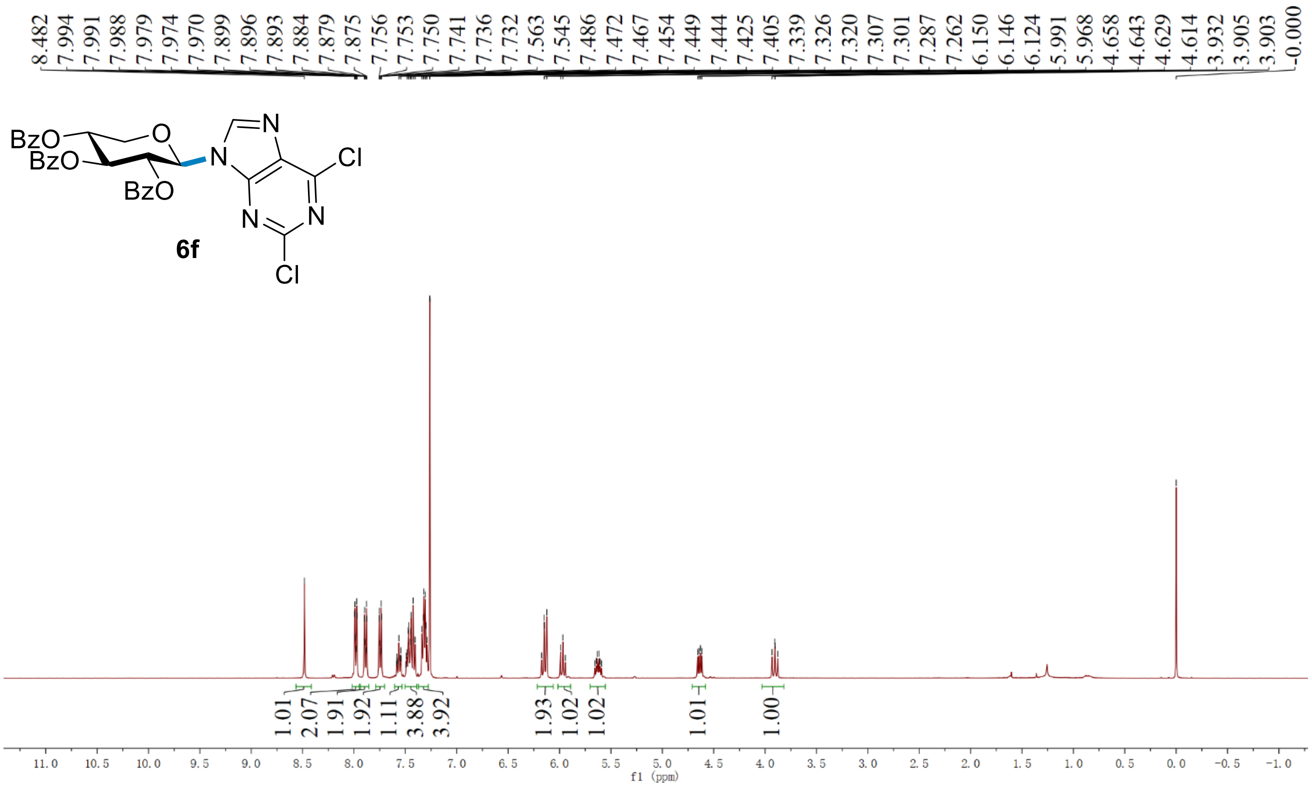

^{13}C NMR Spectrum of **6f** (101 MHz, $CDCl_3$)

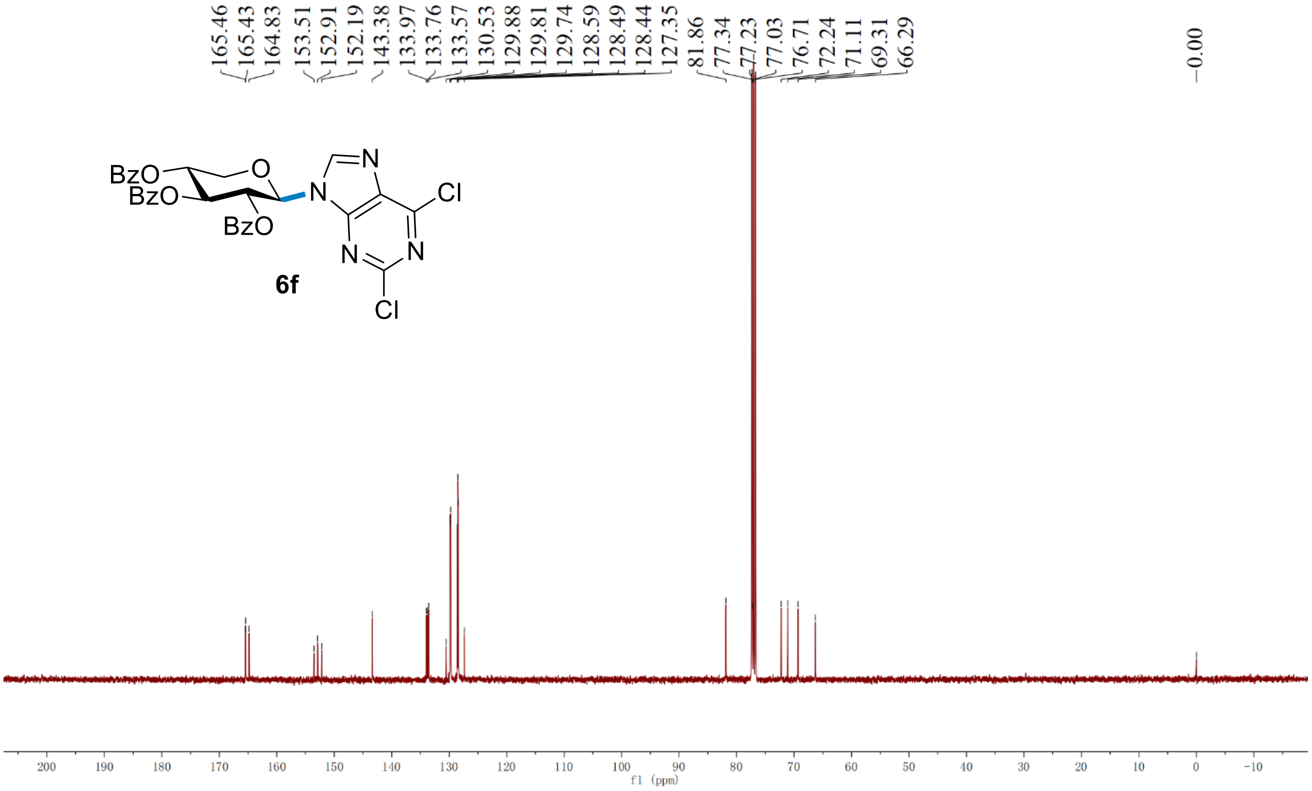

^{1}H NMR Spectrum of **6g** (400 MHz, $CDCl_3$)

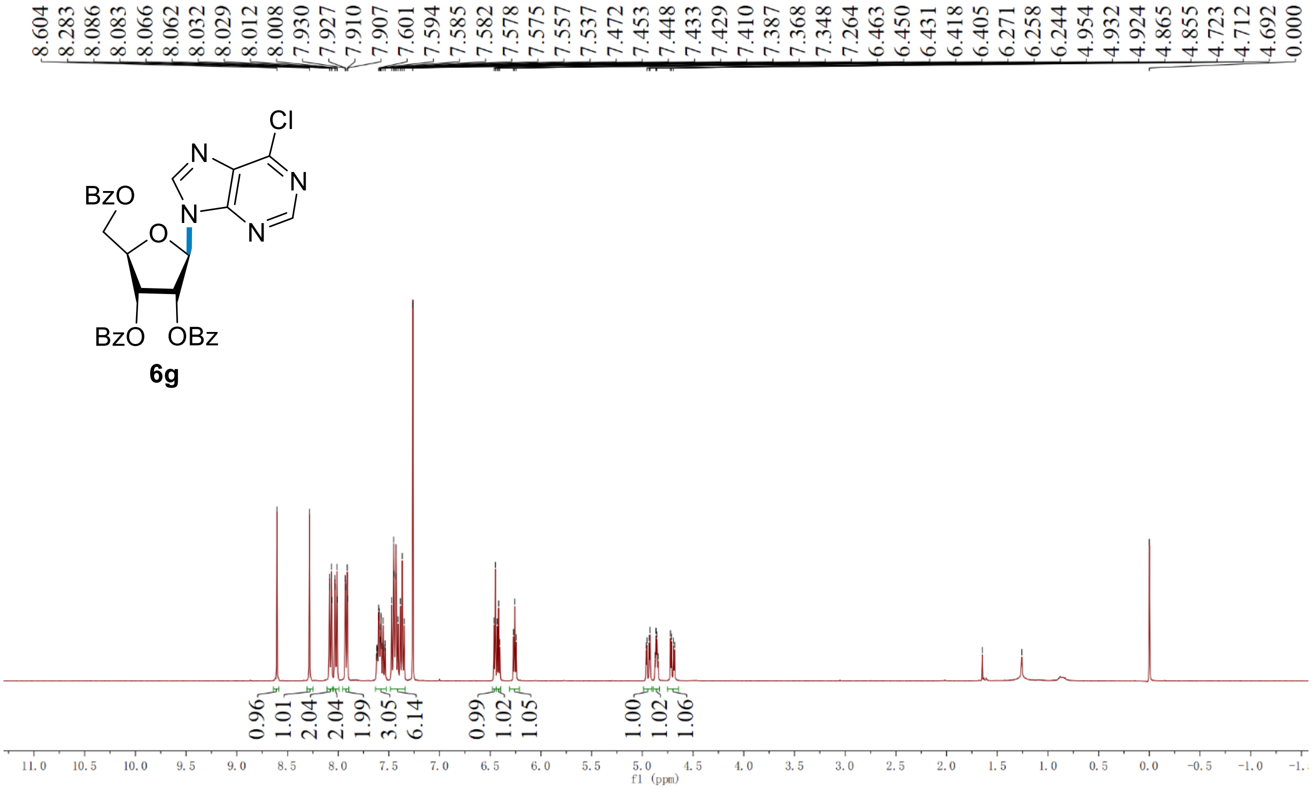

^{13}C NMR Spectrum of **6g** (101 MHz, $CDCl_3$)

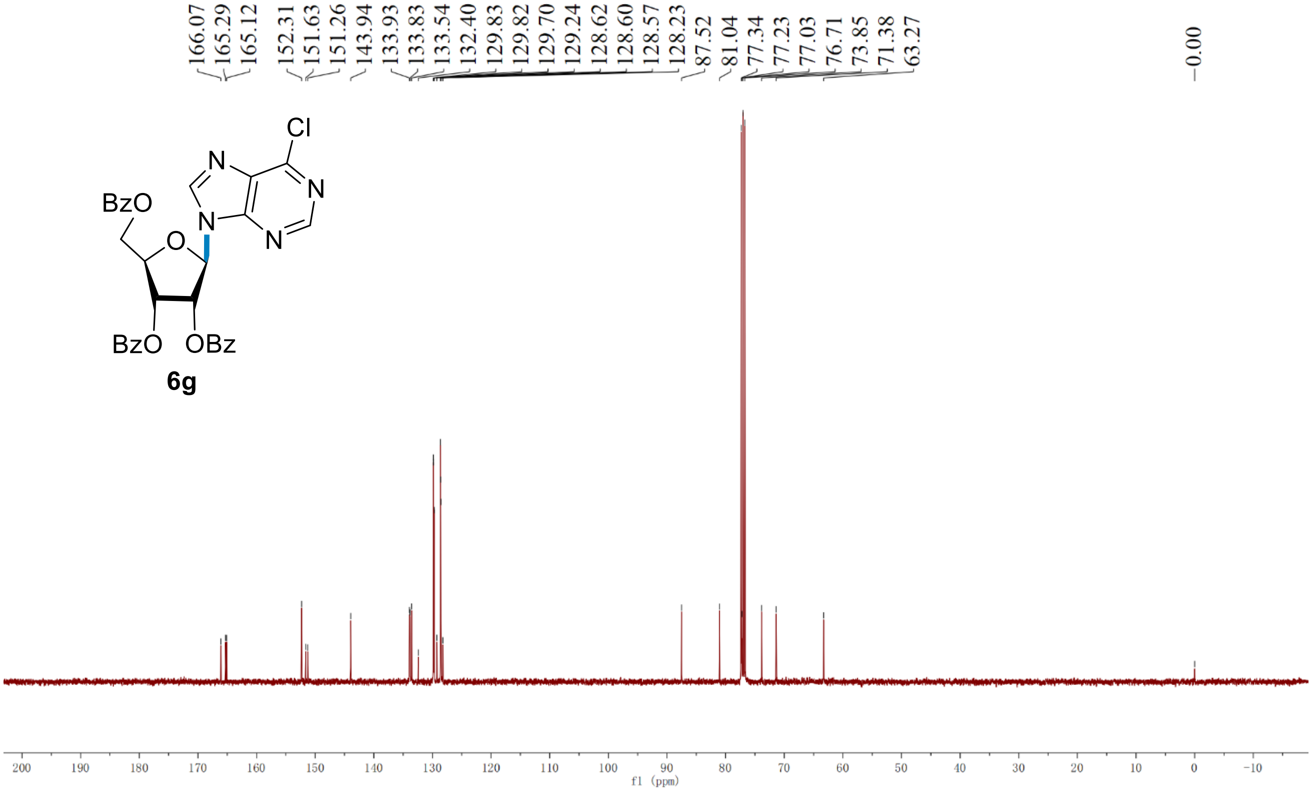

^{1}H NMR Spectrum of **6h** (400 MHz, $CDCl_3$)

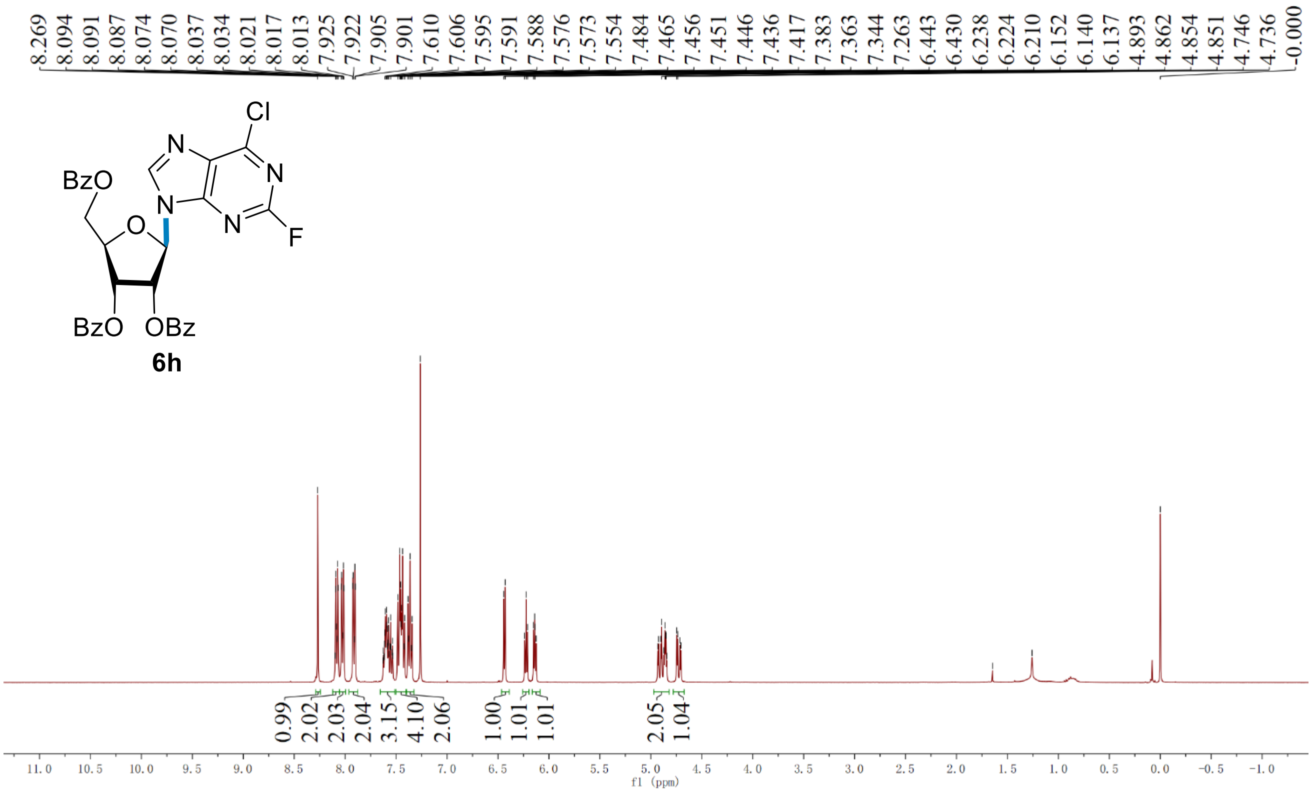

^{13}C NMR Spectrum of **6h** (101 MHz, $CDCl_3$)

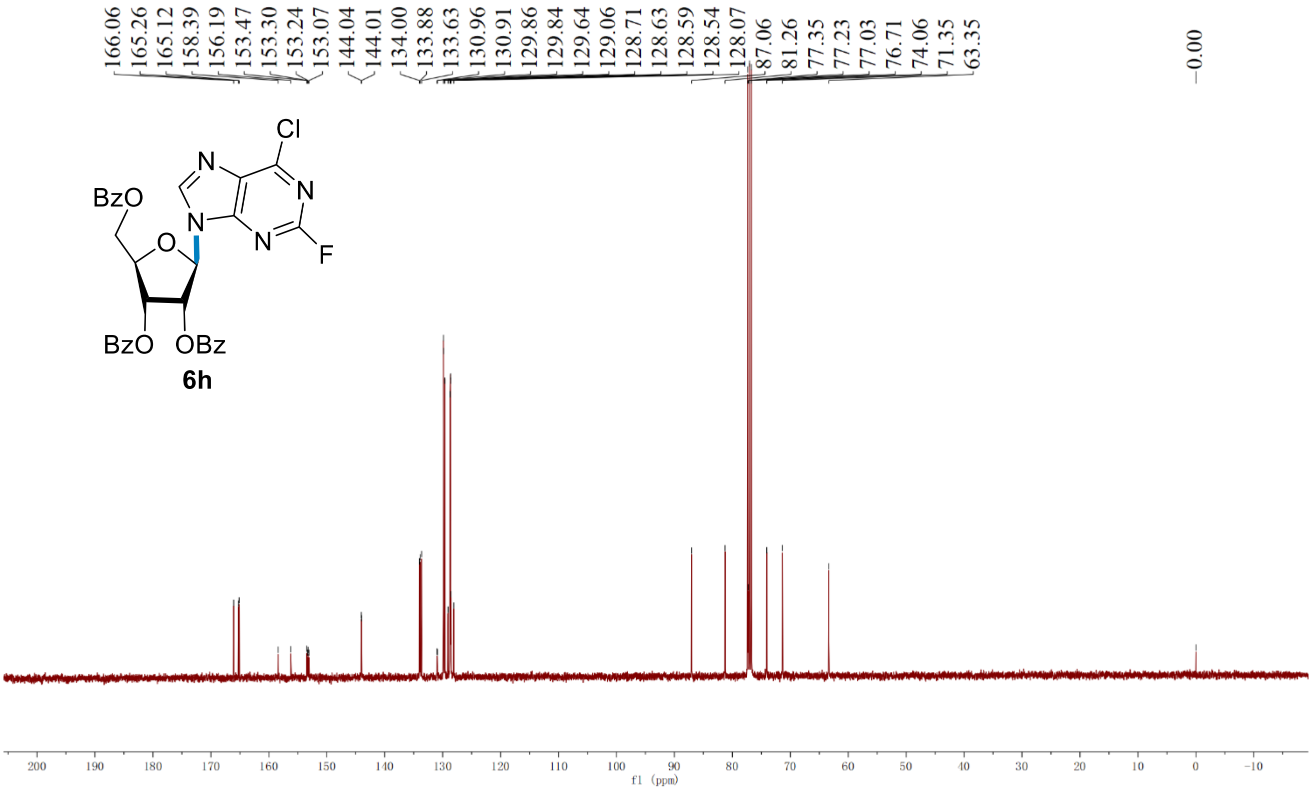

^{19}F NMR Spectrum of **6h** (376 MHz, $CDCl_3$)

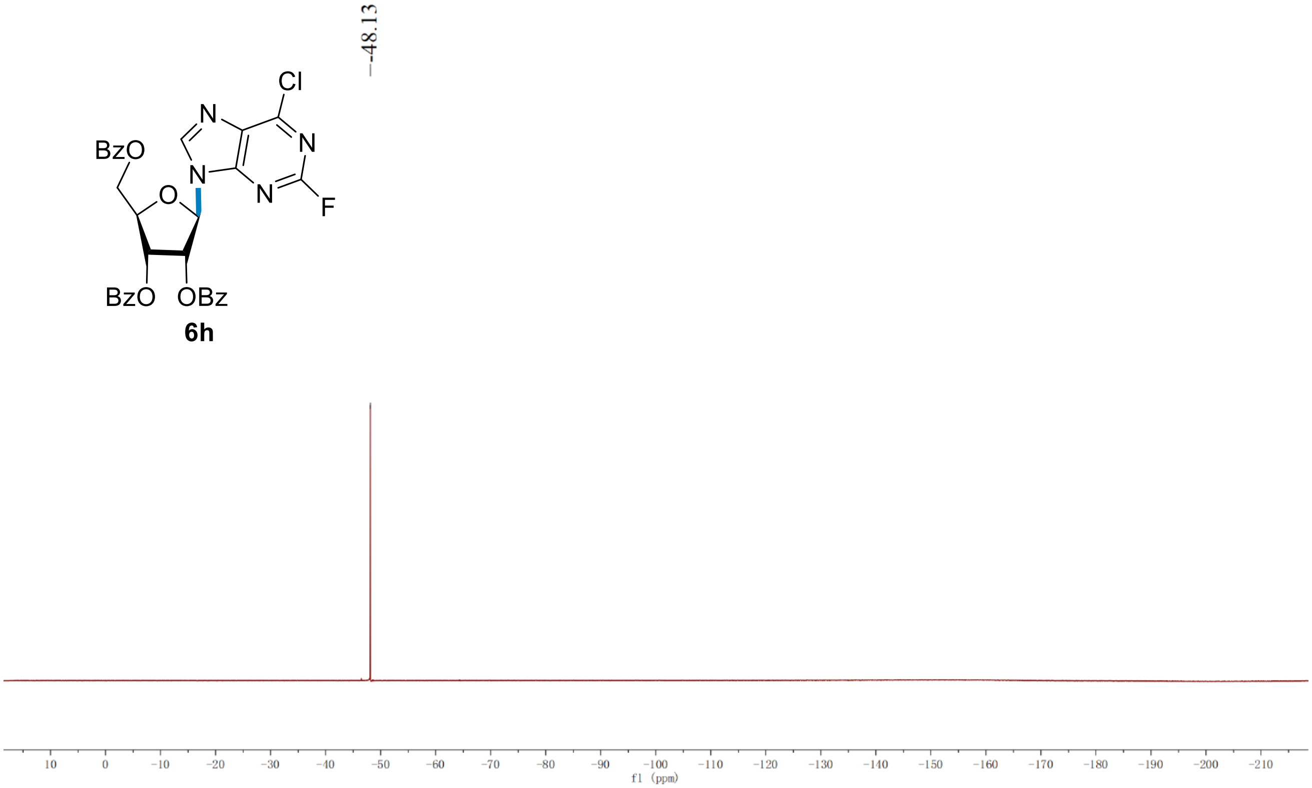

^{1}H NMR Spectrum of **7a** (400 MHz, $CDCl_3$)

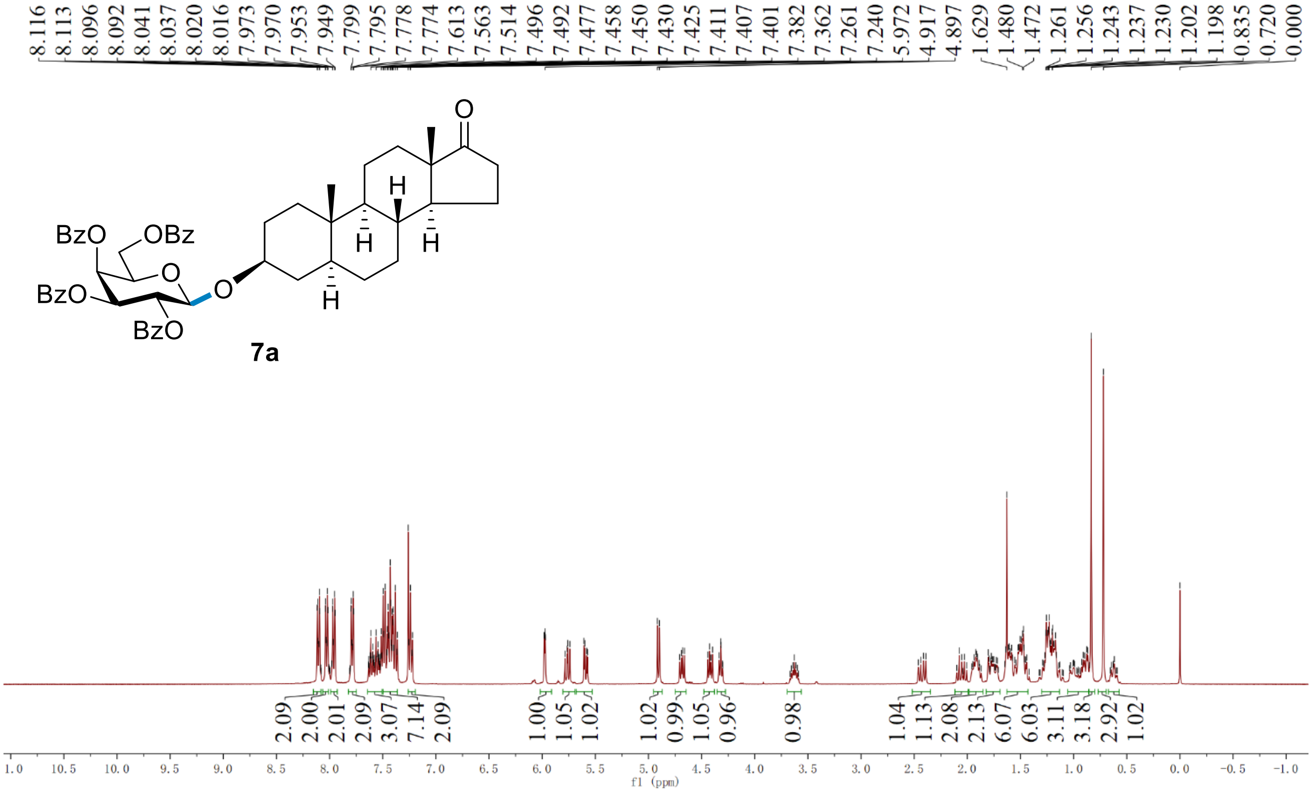

^{1}H NMR Spectrum of **7b** (400 MHz, $CDCl_3$)

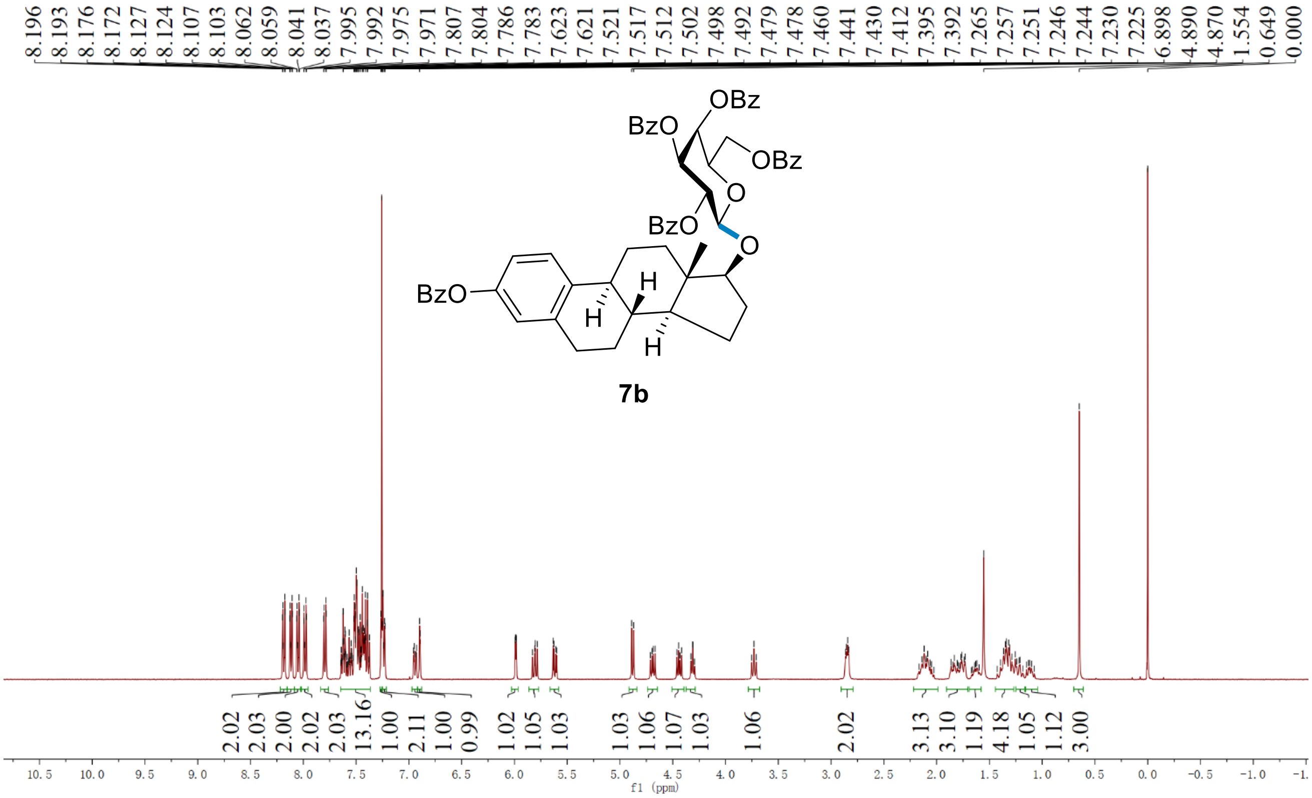

^{13}C NMR Spectrum of **7b** (101 MHz, $CDCl_3$)

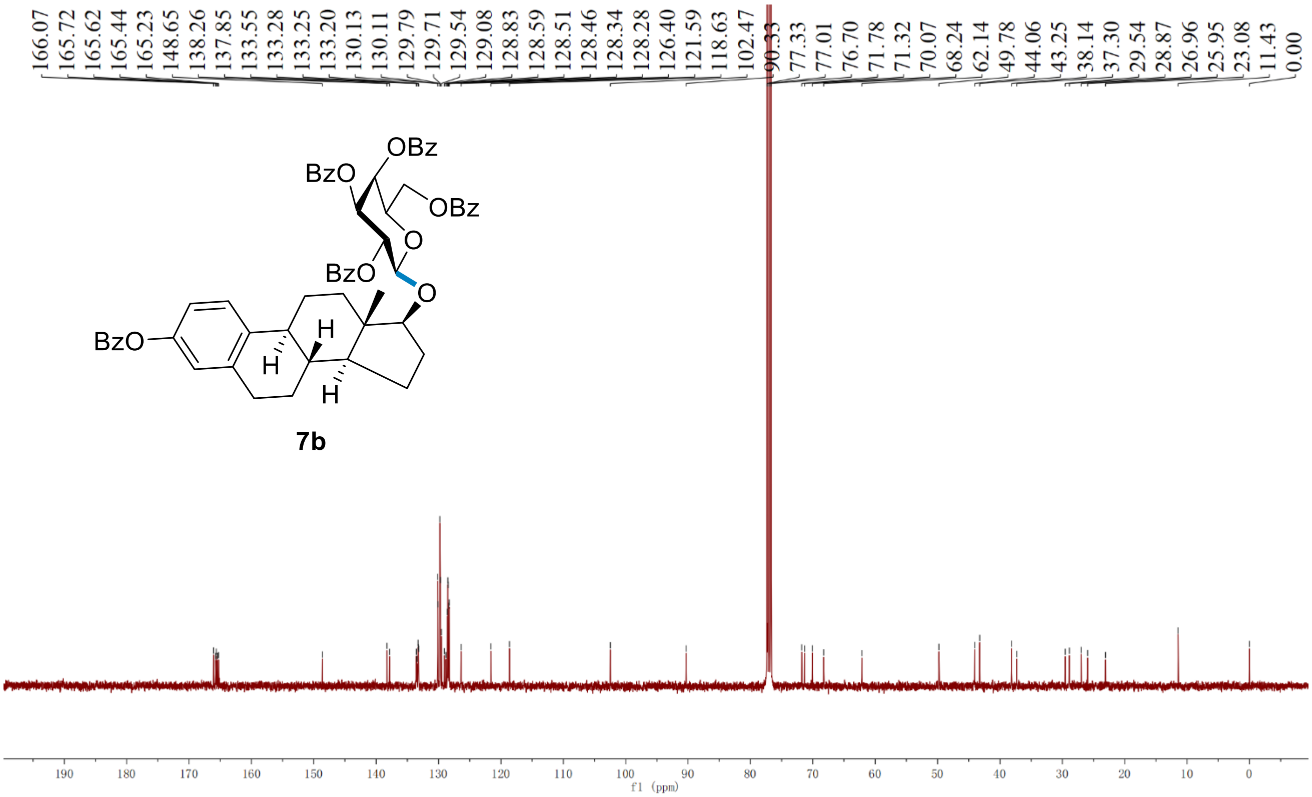

^{1}H NMR Spectrum of **7c** (400 MHz, $CDCl_3$)

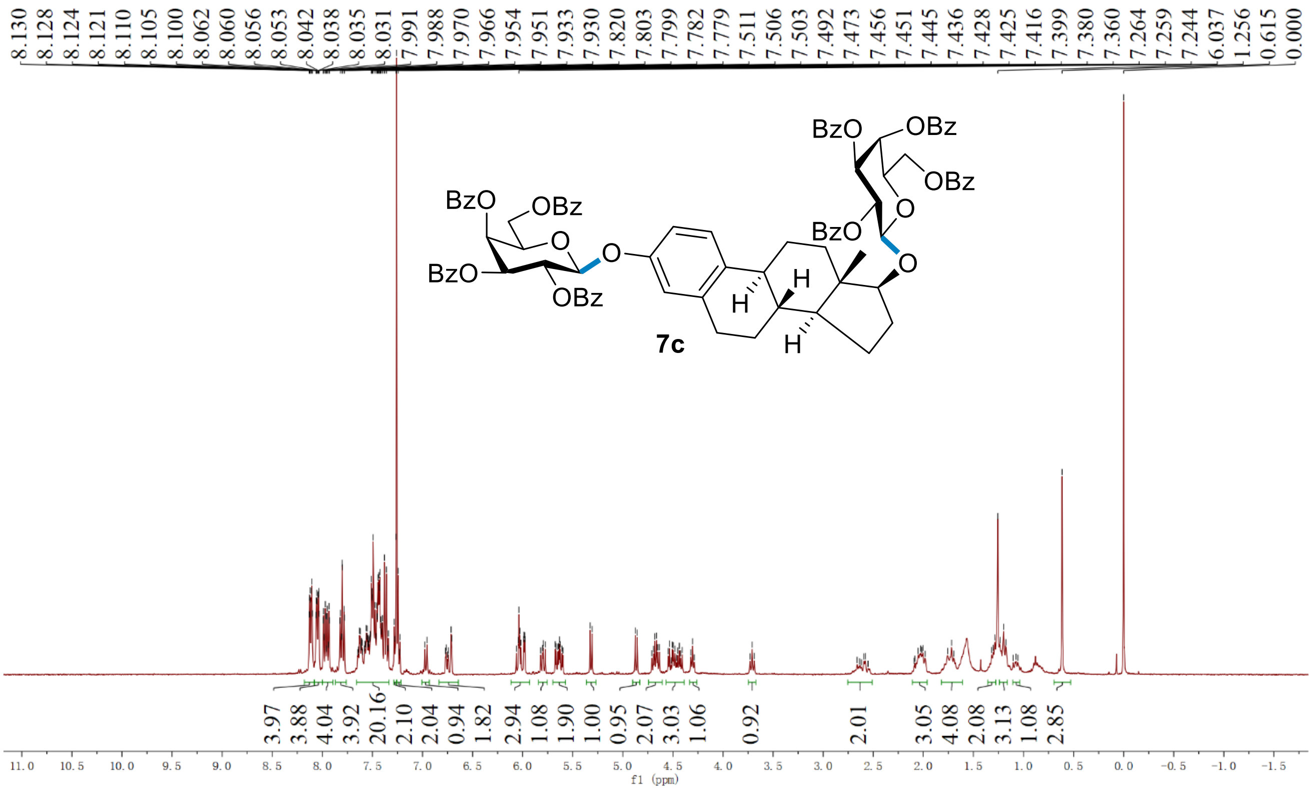

^{13}C NMR Spectrum of **7c** (101 MHz, $CDCl_3$)

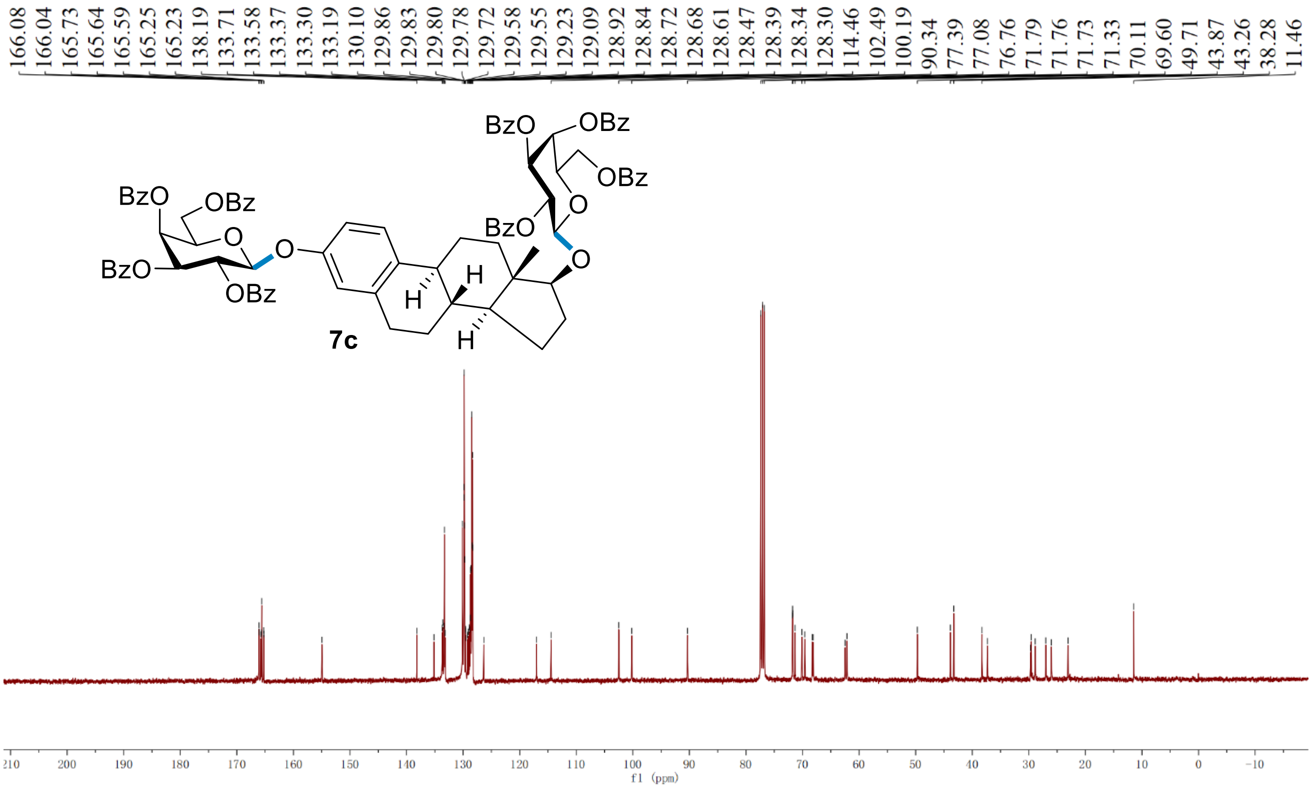

^{1}H NMR Spectrum of **7d** (400 MHz, $CDCl_3$)

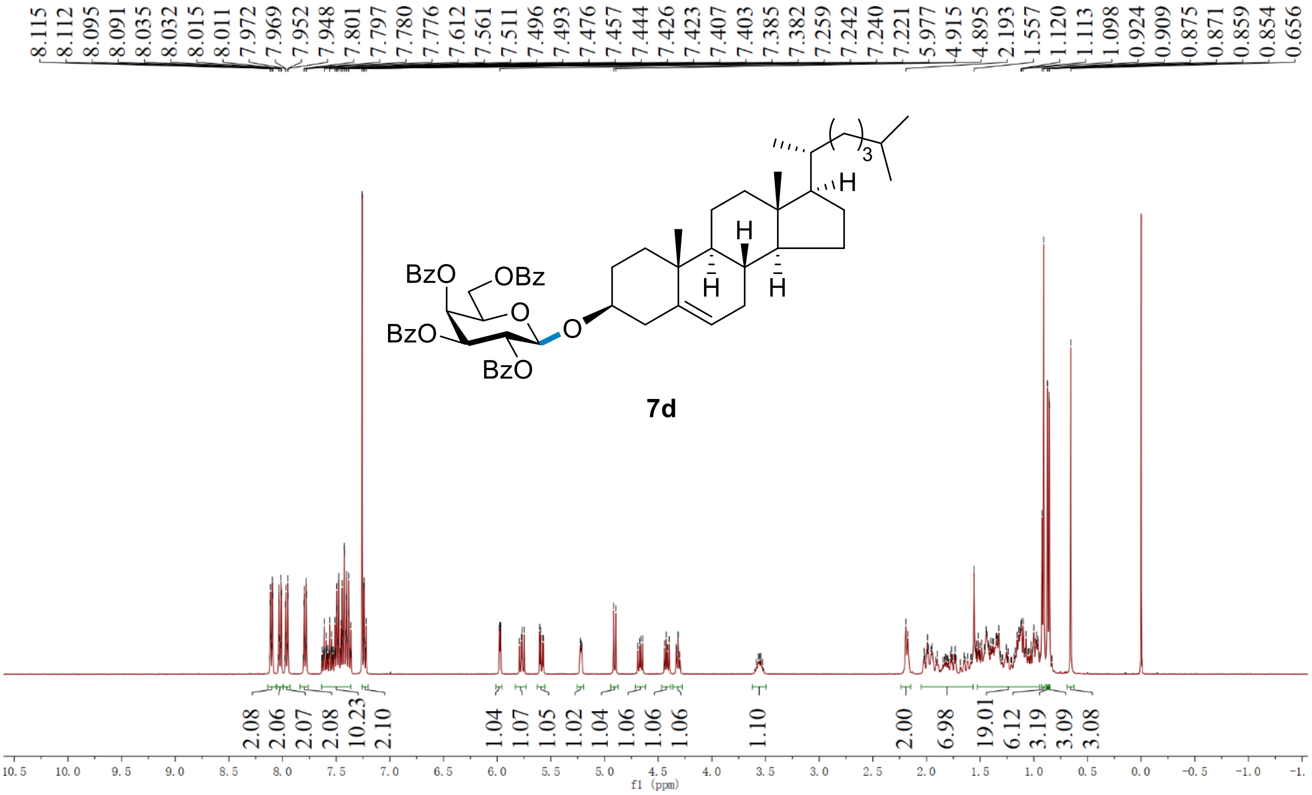

^{1}H NMR Spectrum of **7e** (400 MHz, $CDCl_3$)

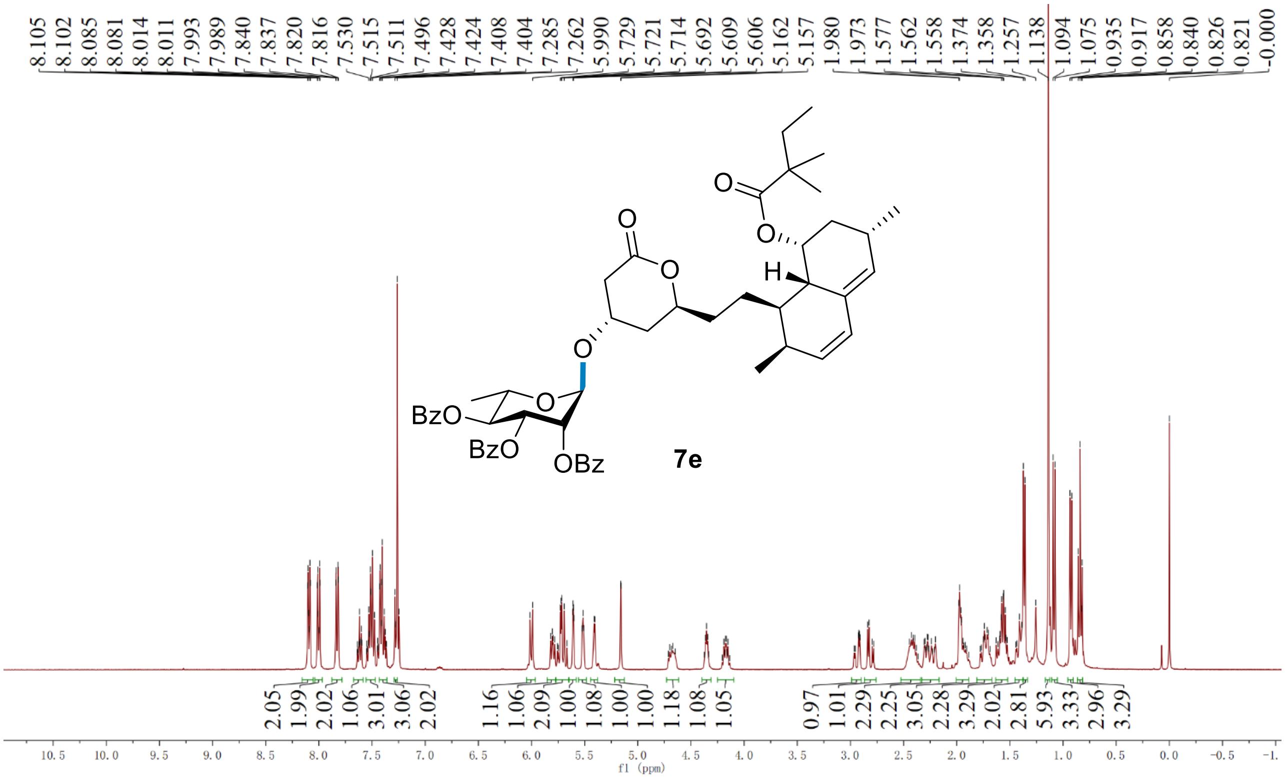

^{13}C NMR Spectrum of **7e** (101 MHz, $CDCl_3$)

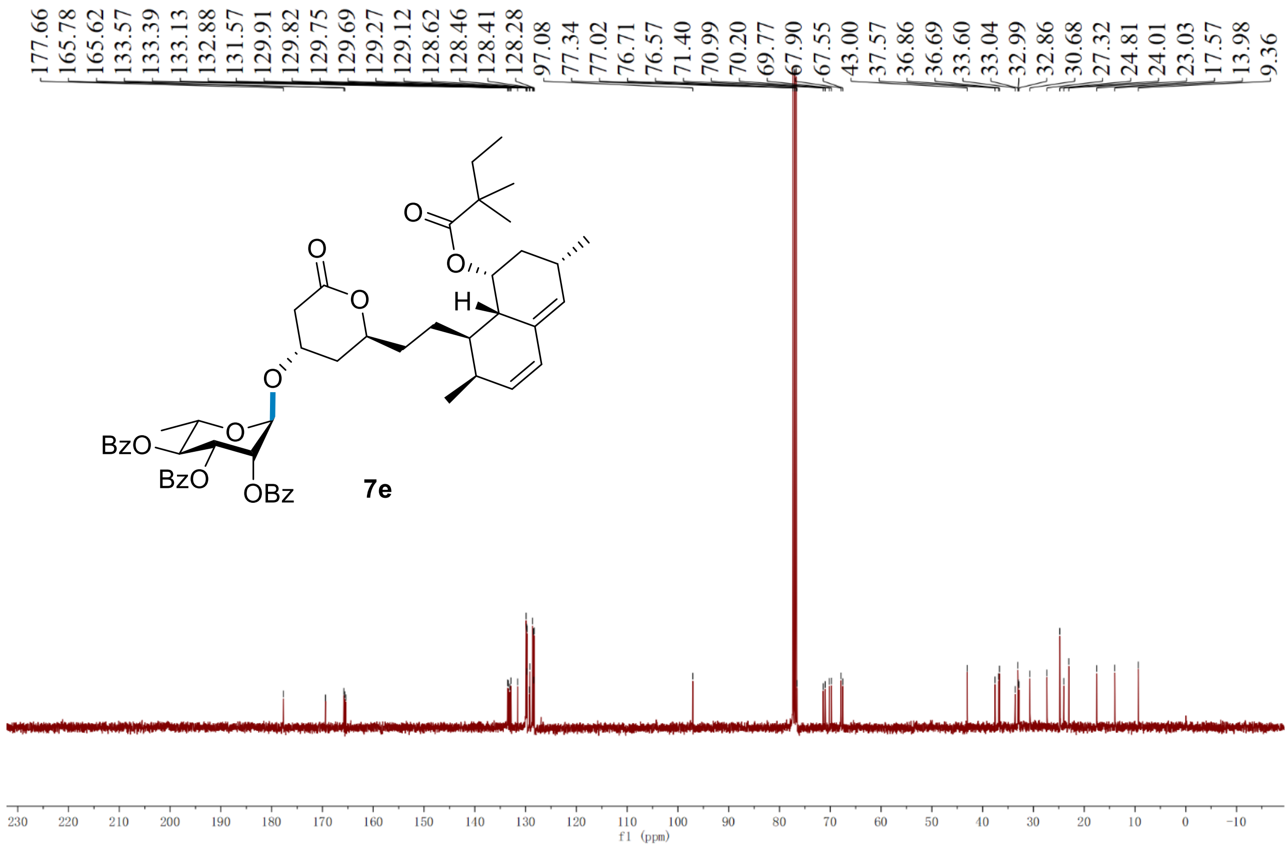

^{1}H NMR Spectrum of **7f** (400 MHz, $CDCl_3$)

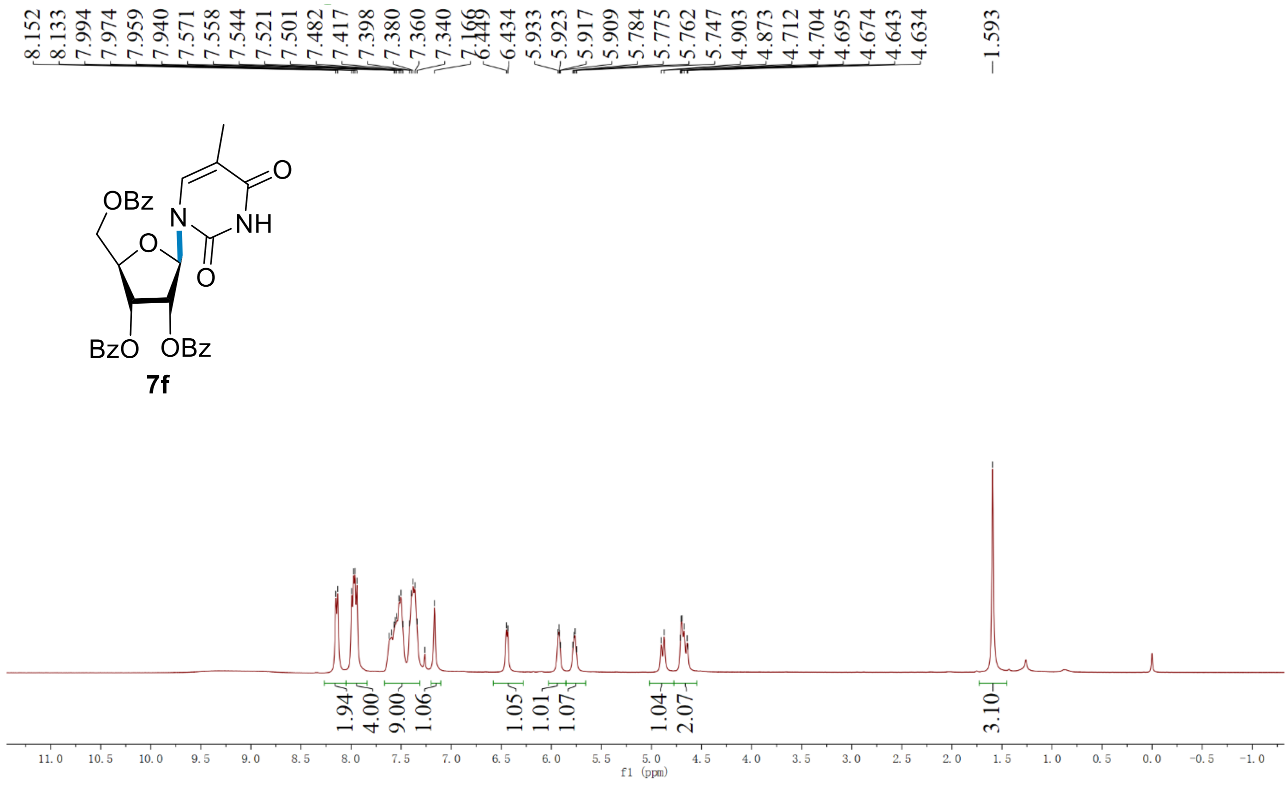

^{1}H NMR Spectrum of **8a** (400 MHz, $CDCl_3$)

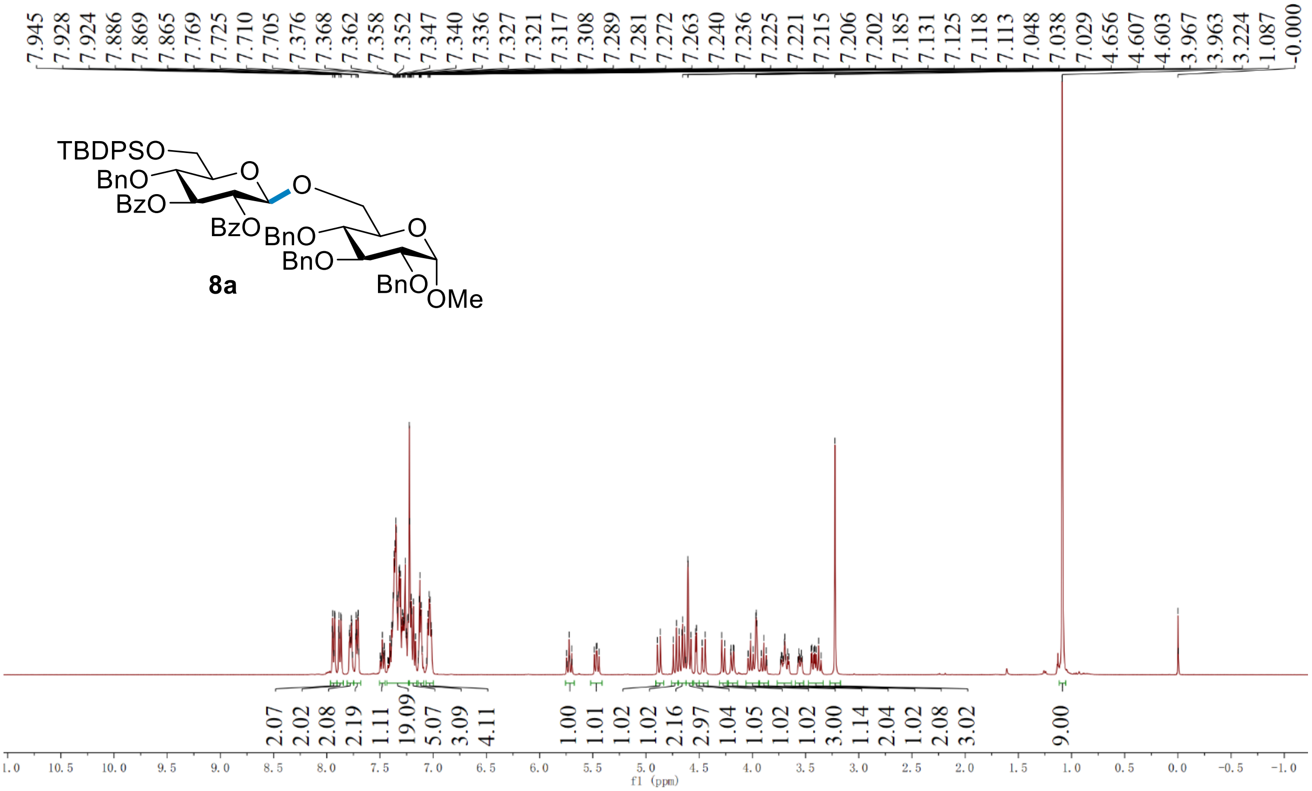

^{13}C NMR Spectrum of **8a** (101 MHz, $CDCl_3$)

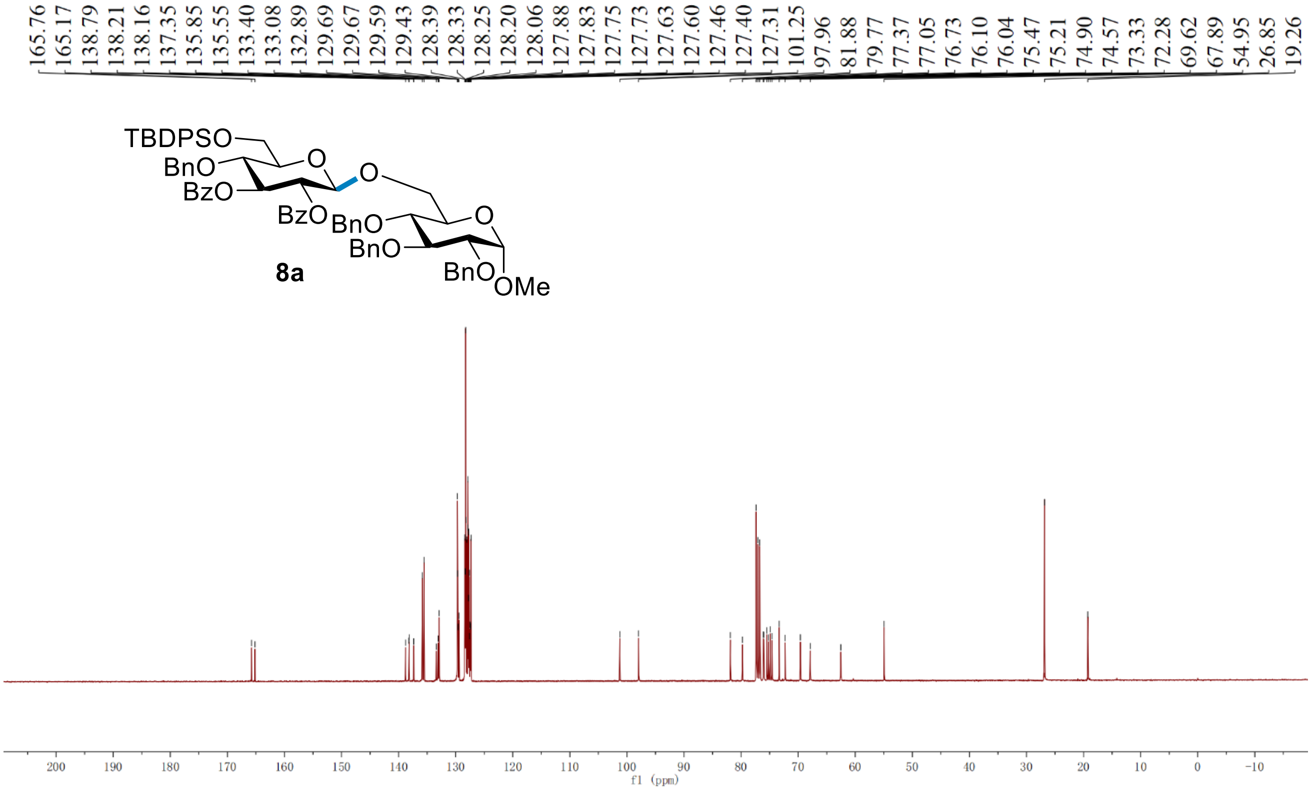

^{1}H NMR Spectrum of **8b** (400 MHz, $CDCl_3$)

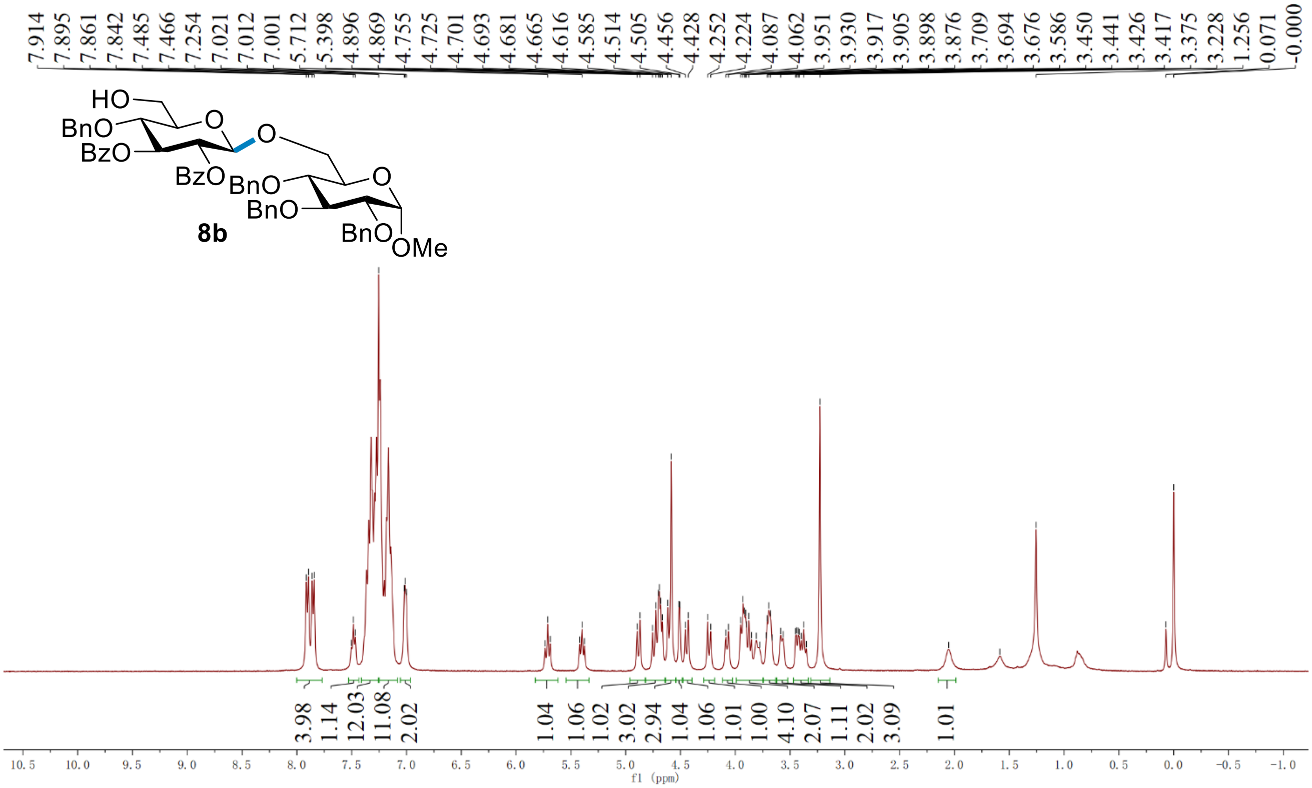

^{13}C NMR Spectrum of **8b** (101 MHz, $CDCl_3$)

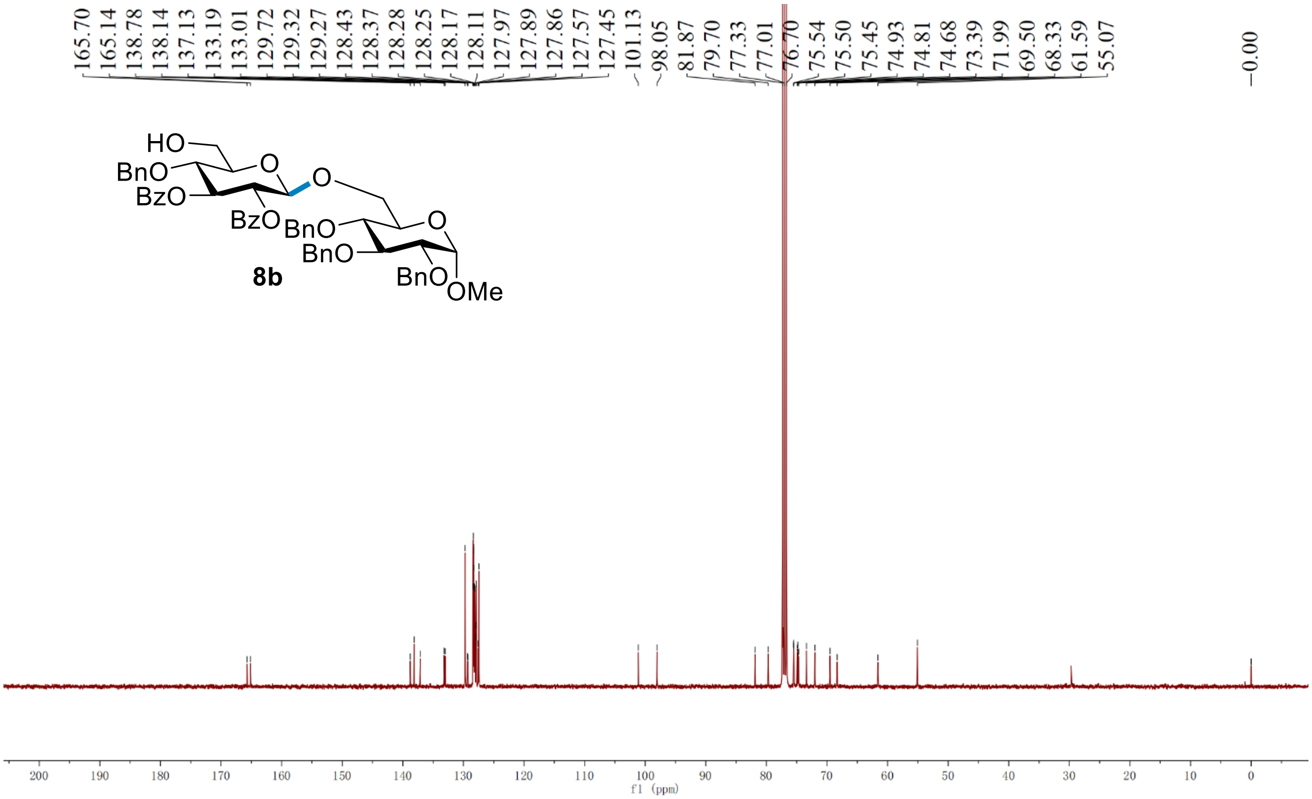

^{1}H NMR Spectrum of **8c** (400 MHz, $CDCl_3$)

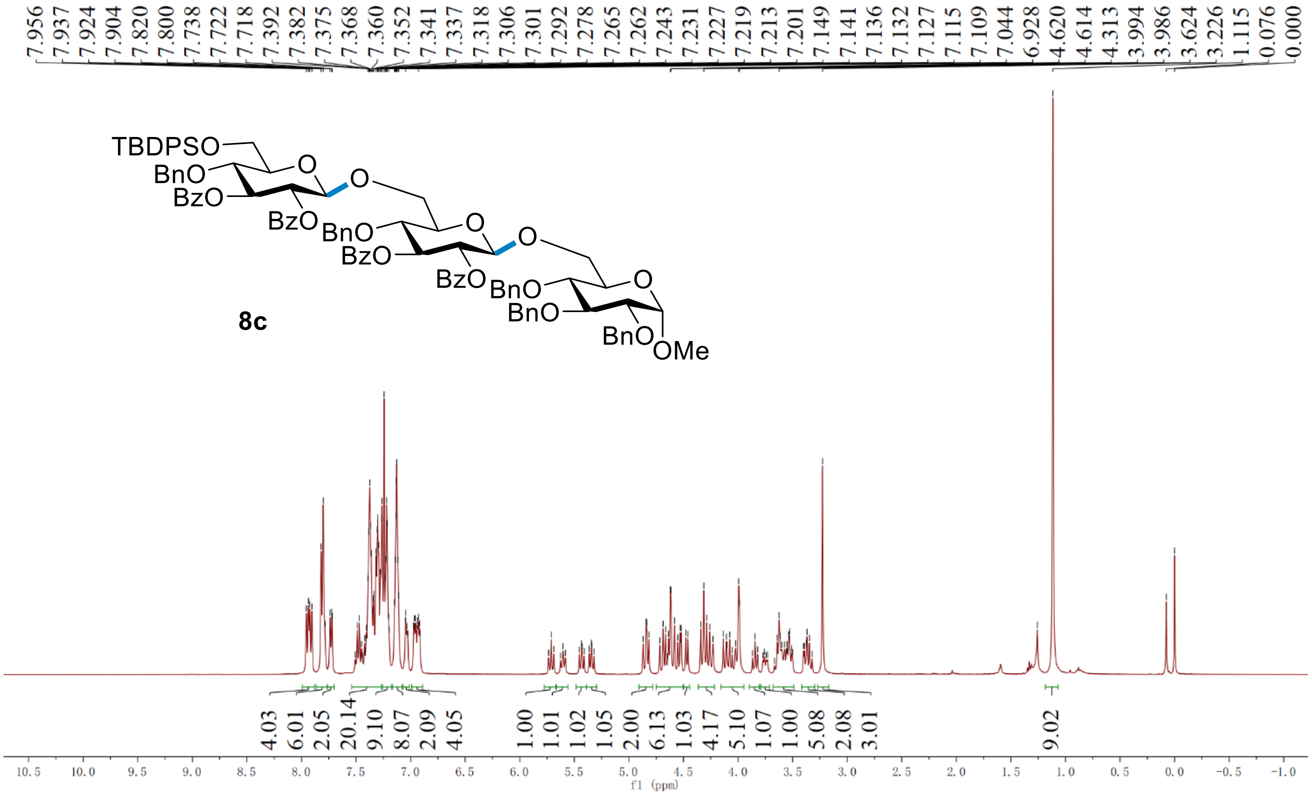

^{13}C NMR Spectrum of **8c** (101 MHz, $CDCl_3$)

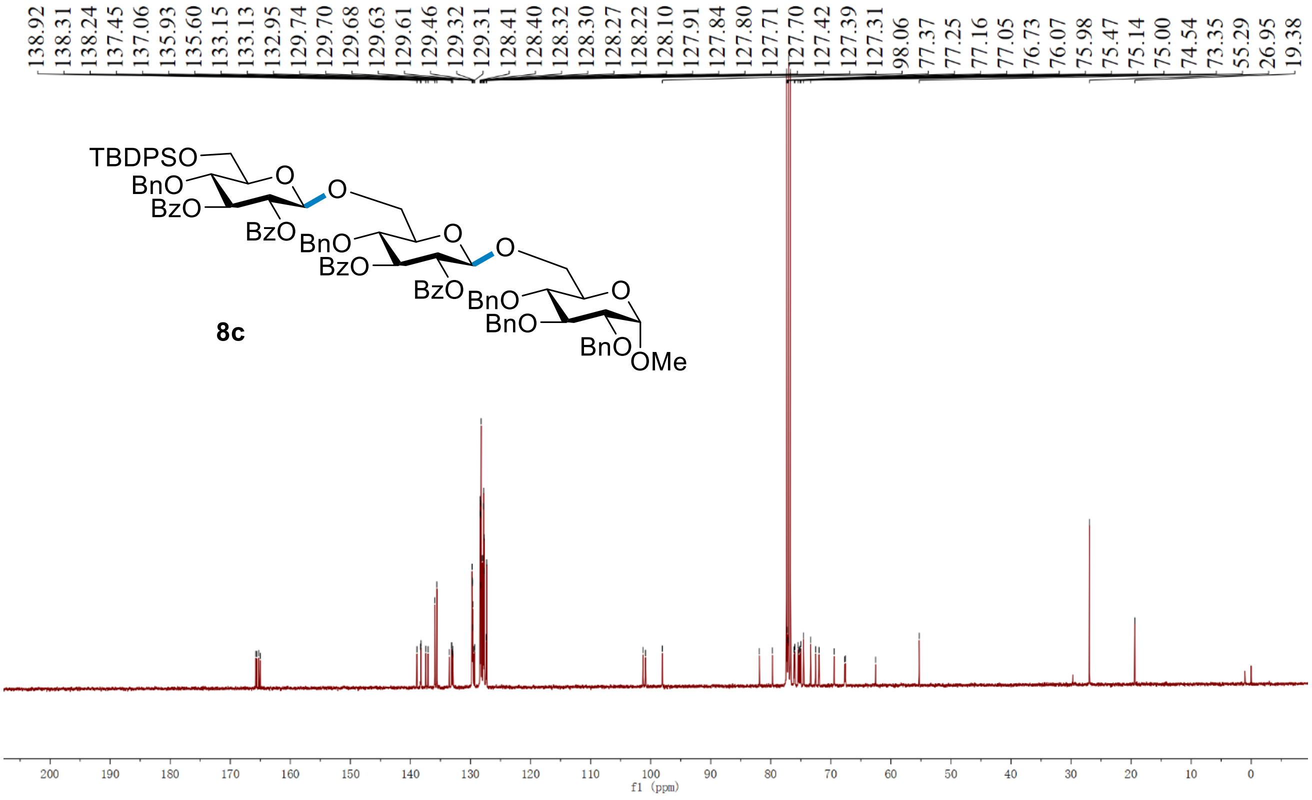

^{1}H NMR Spectrum of **8d** (400 MHz, $CDCl_3$)

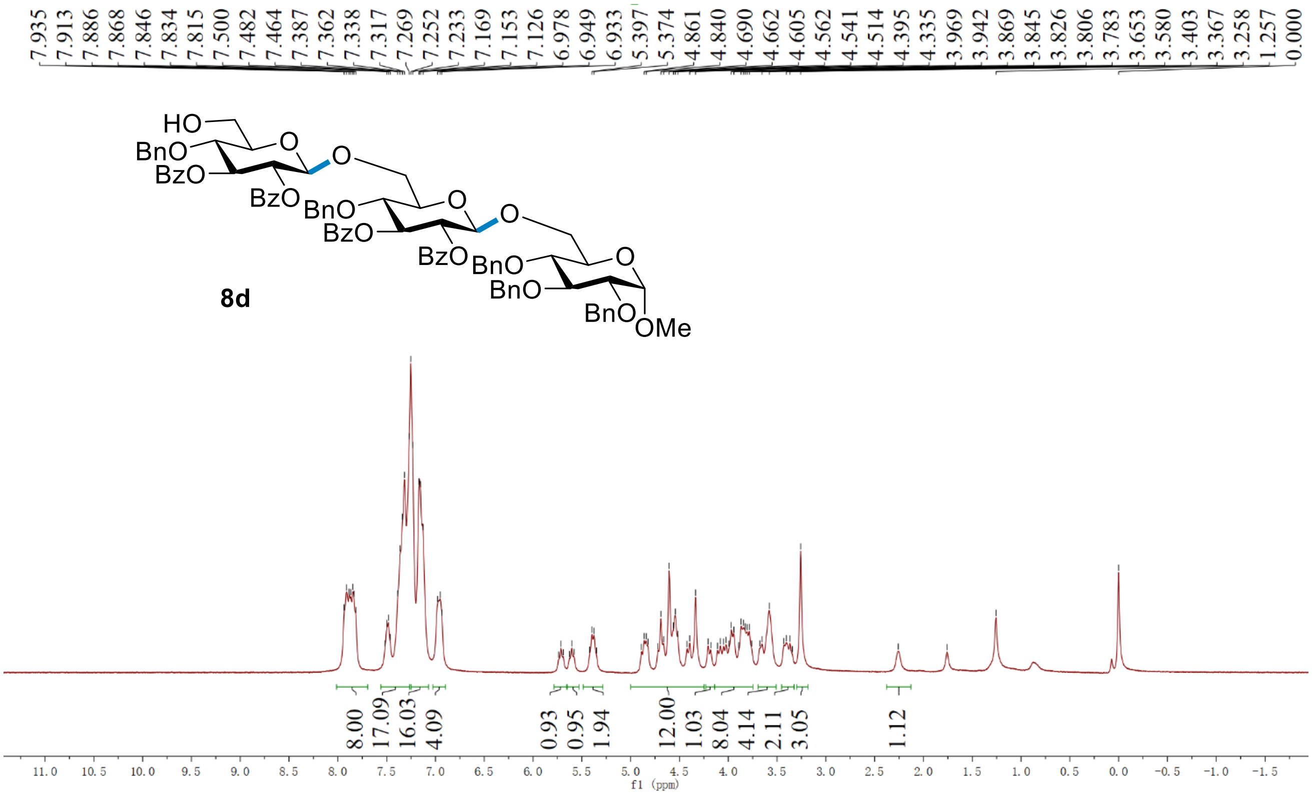

^{13}C NMR Spectrum of **8d** (101 MHz, $CDCl_3$)

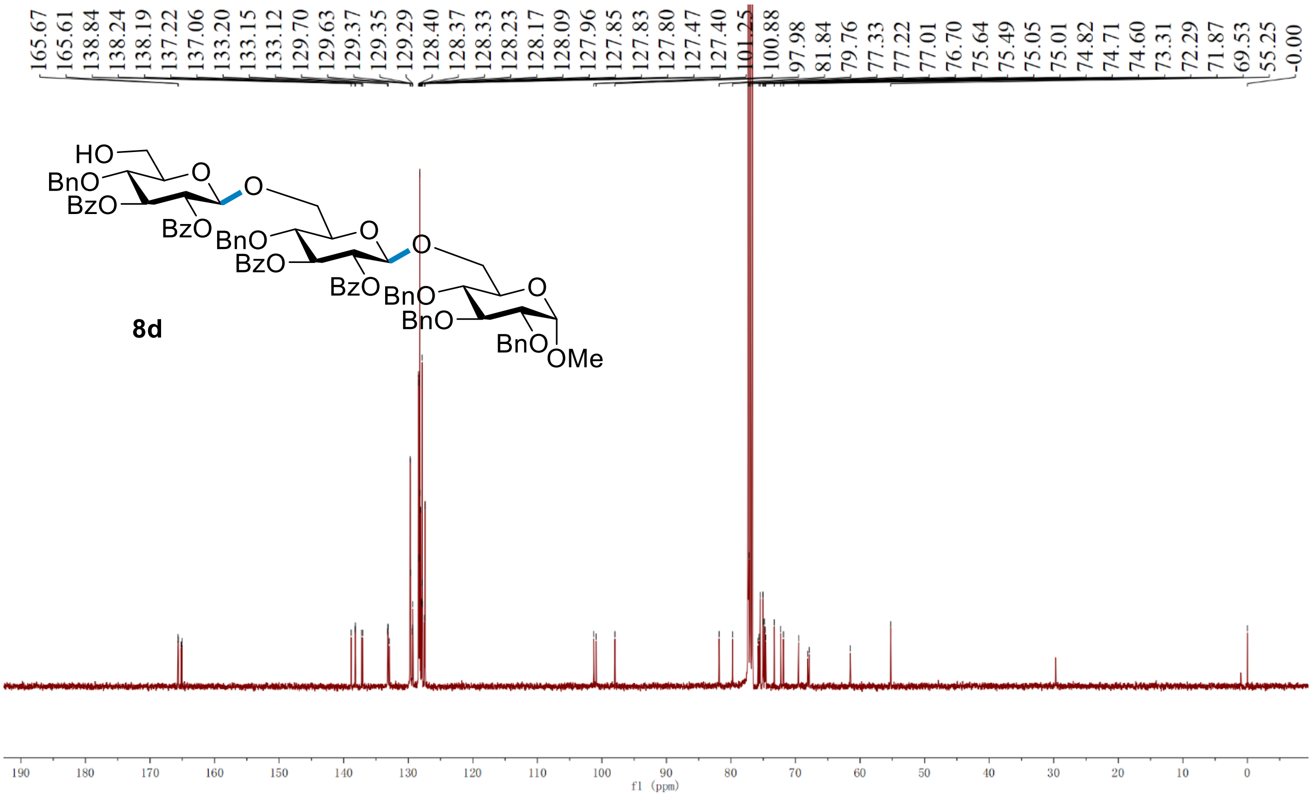

^{1}H NMR Spectrum of **8e** (400 MHz, $CDCl_3$)

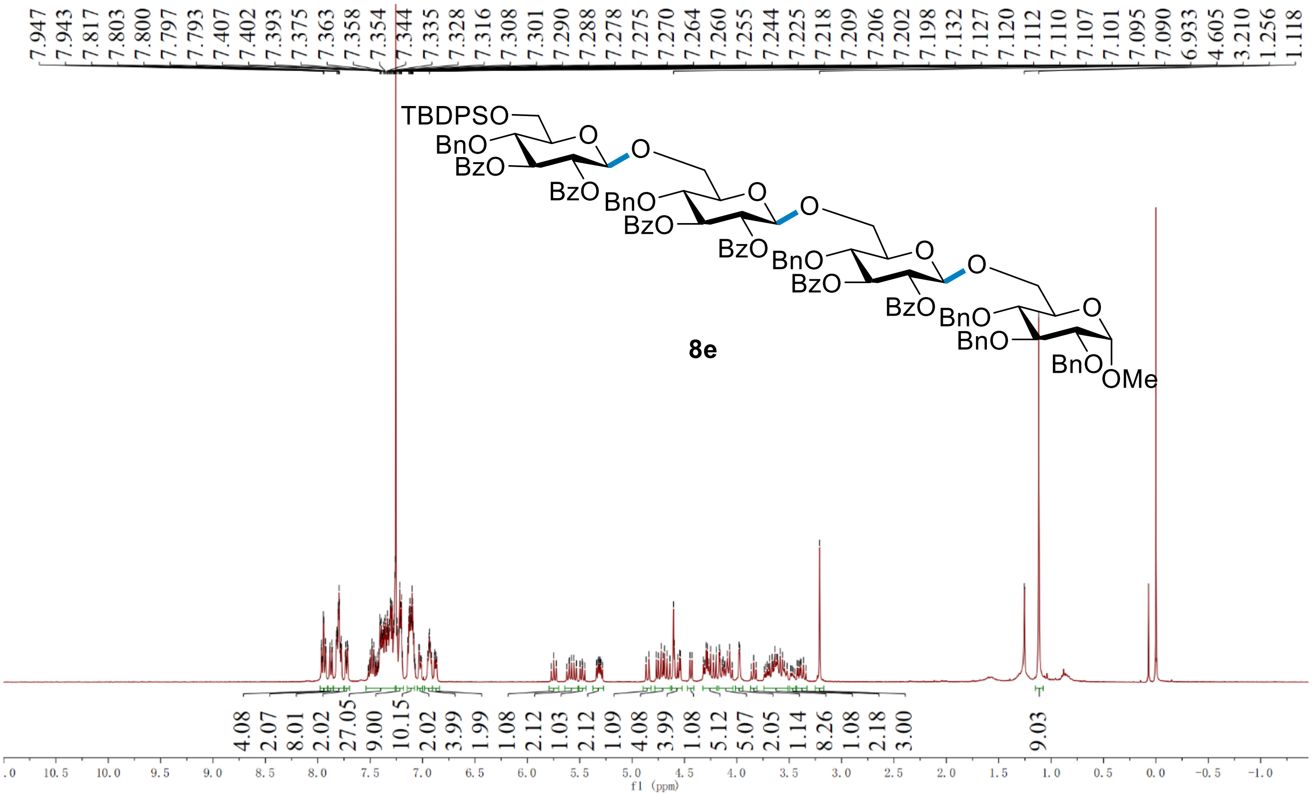

^{13}C NMR Spectrum of **8e** (101 MHz, $CDCl_3$)

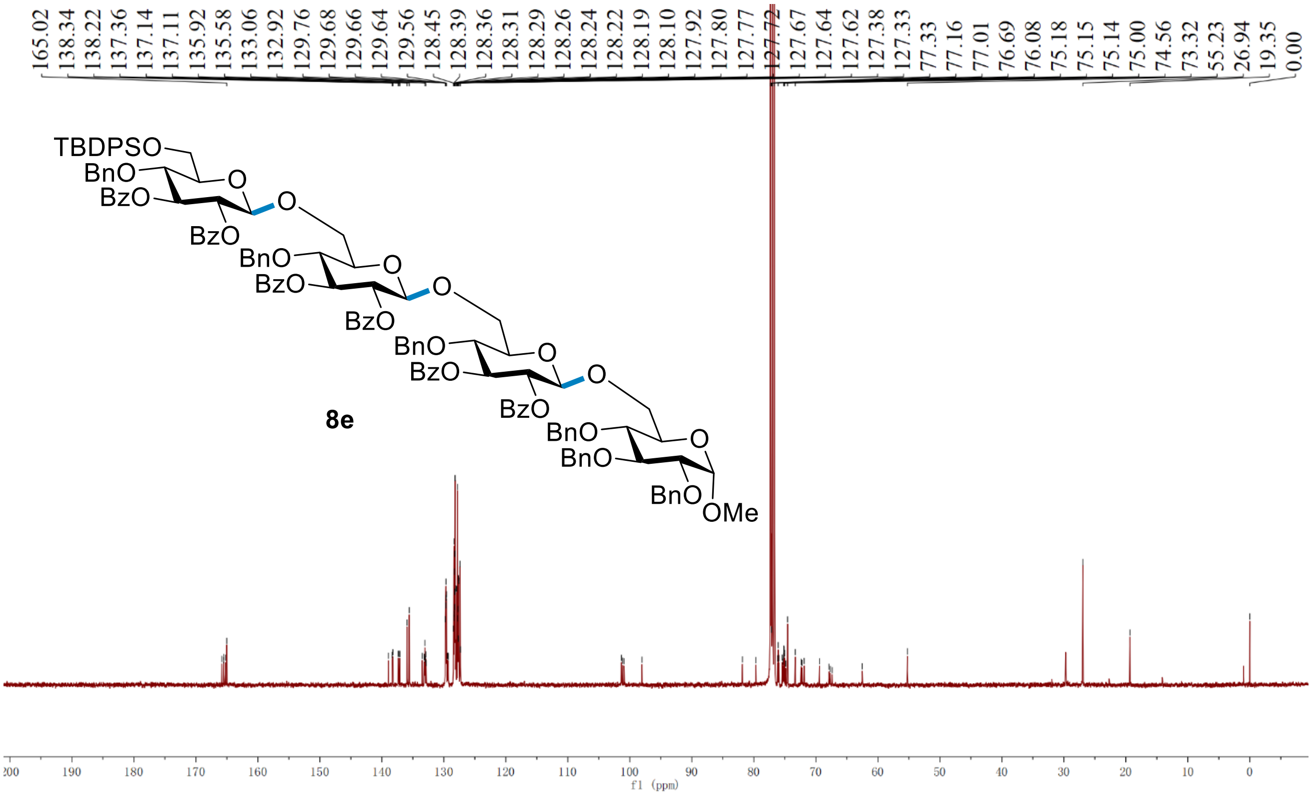

^{1}H NMR Spectrum of **8f** (400 MHz, $CDCl_3$)

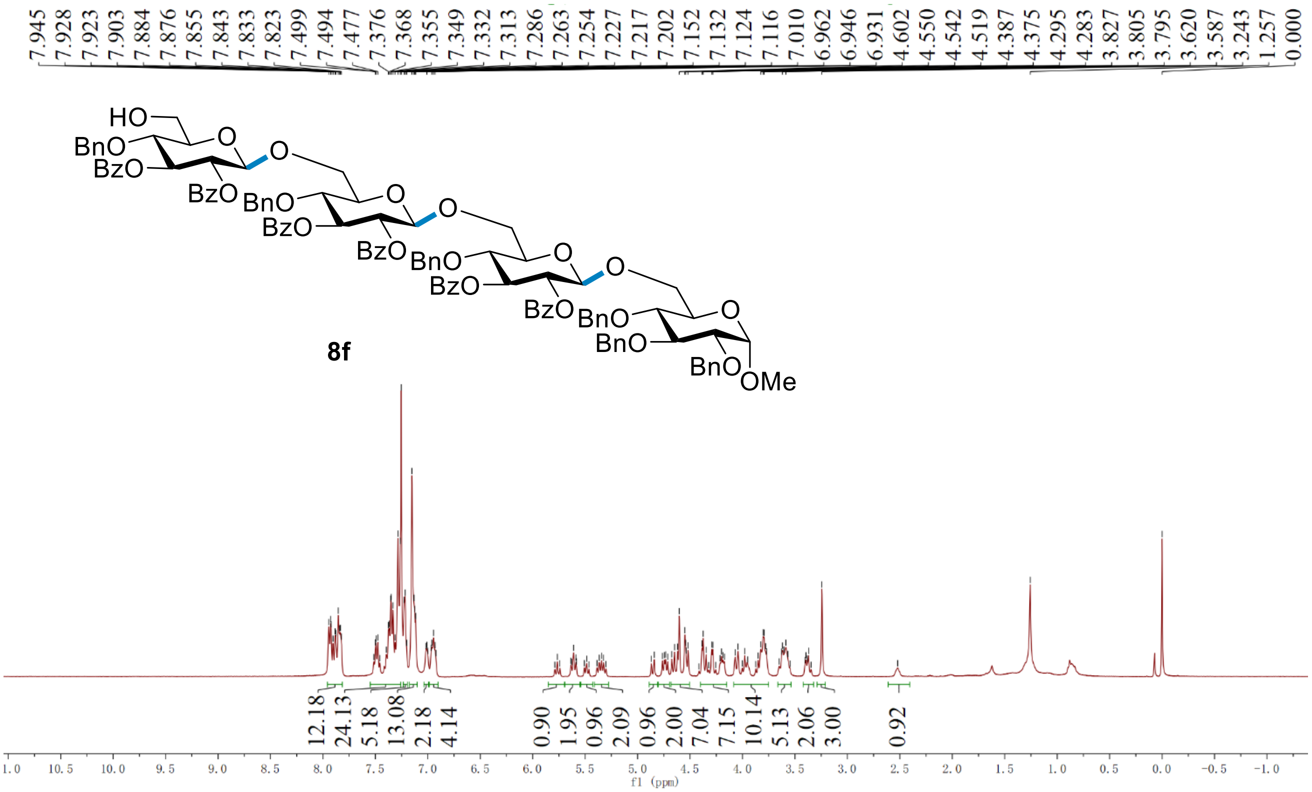

^{13}C NMR Spectrum of **8f** (101 MHz, $CDCl_3$)

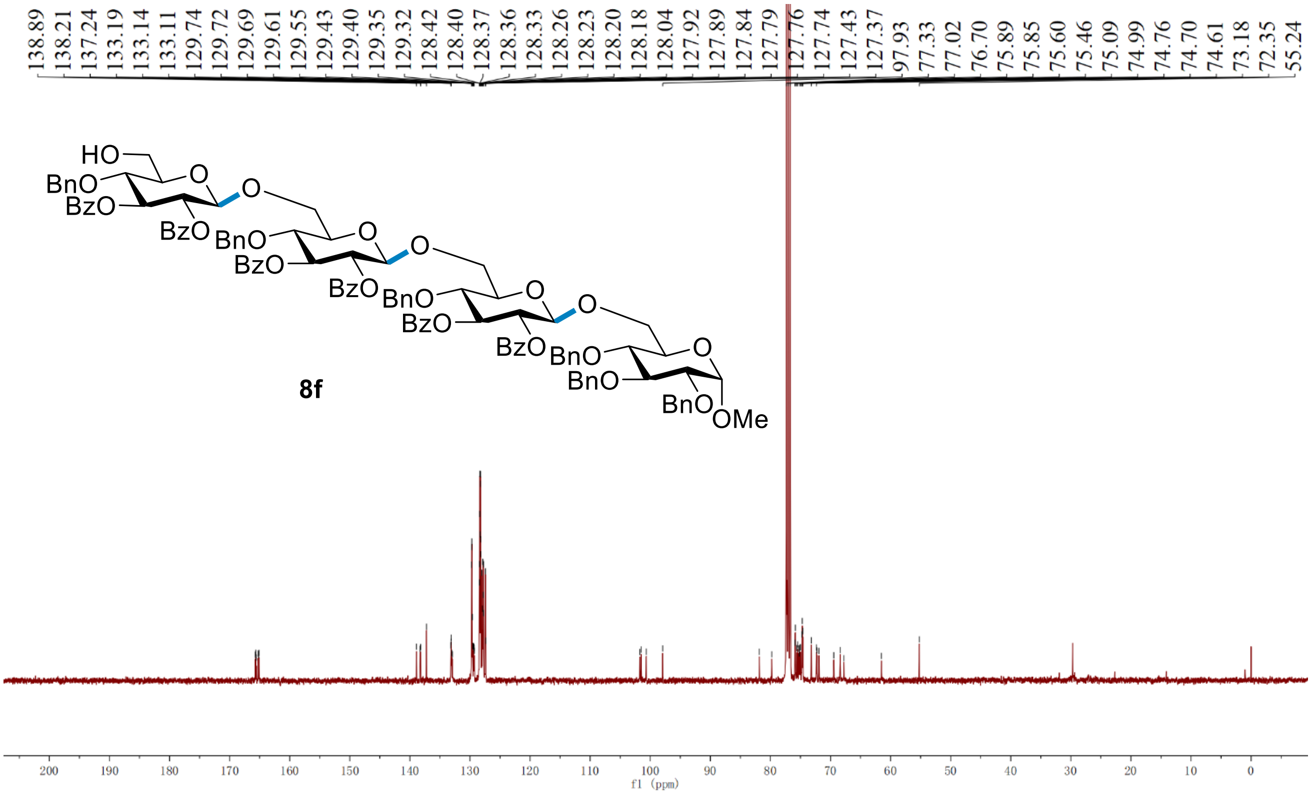

^{1}H NMR Spectrum of **8g** (400 MHz, $CDCl_3$)

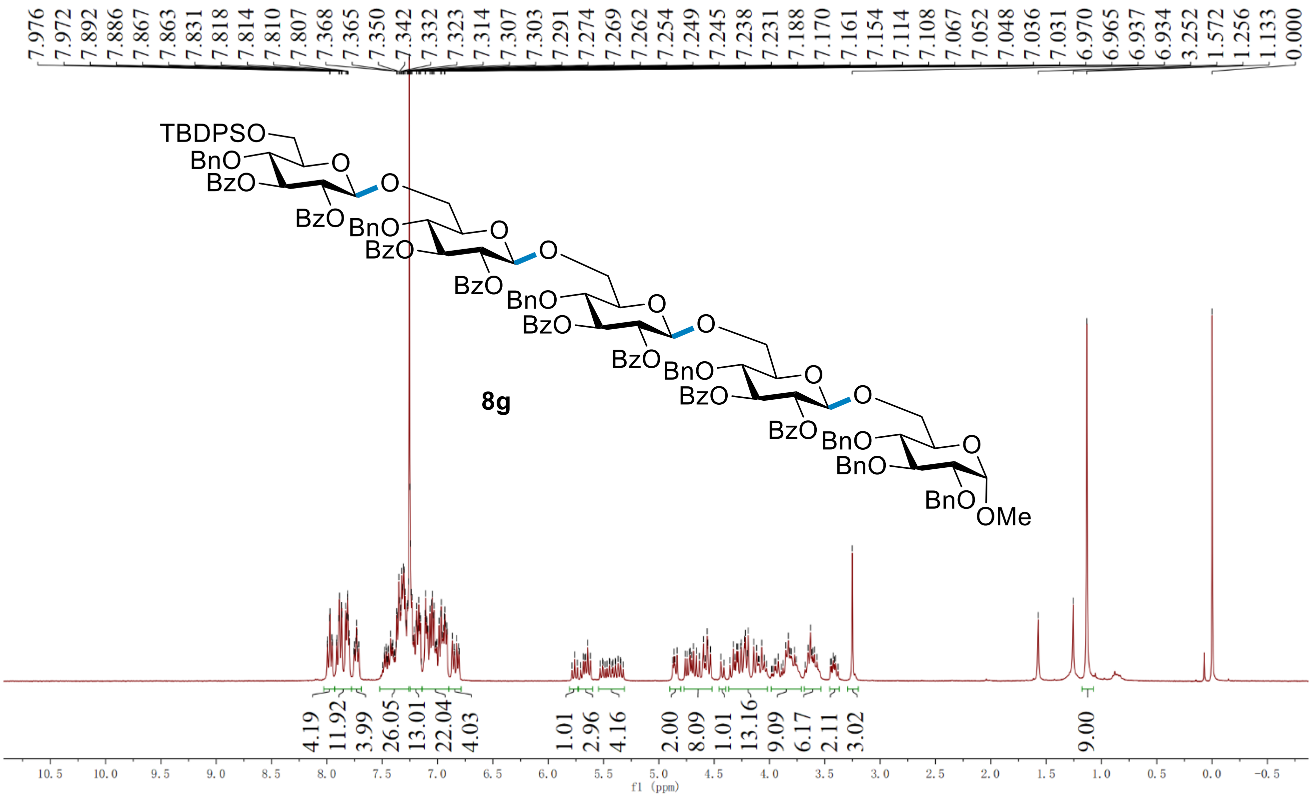

^{13}C NMR Spectrum of **8g** (151 MHz, $CDCl_3$)

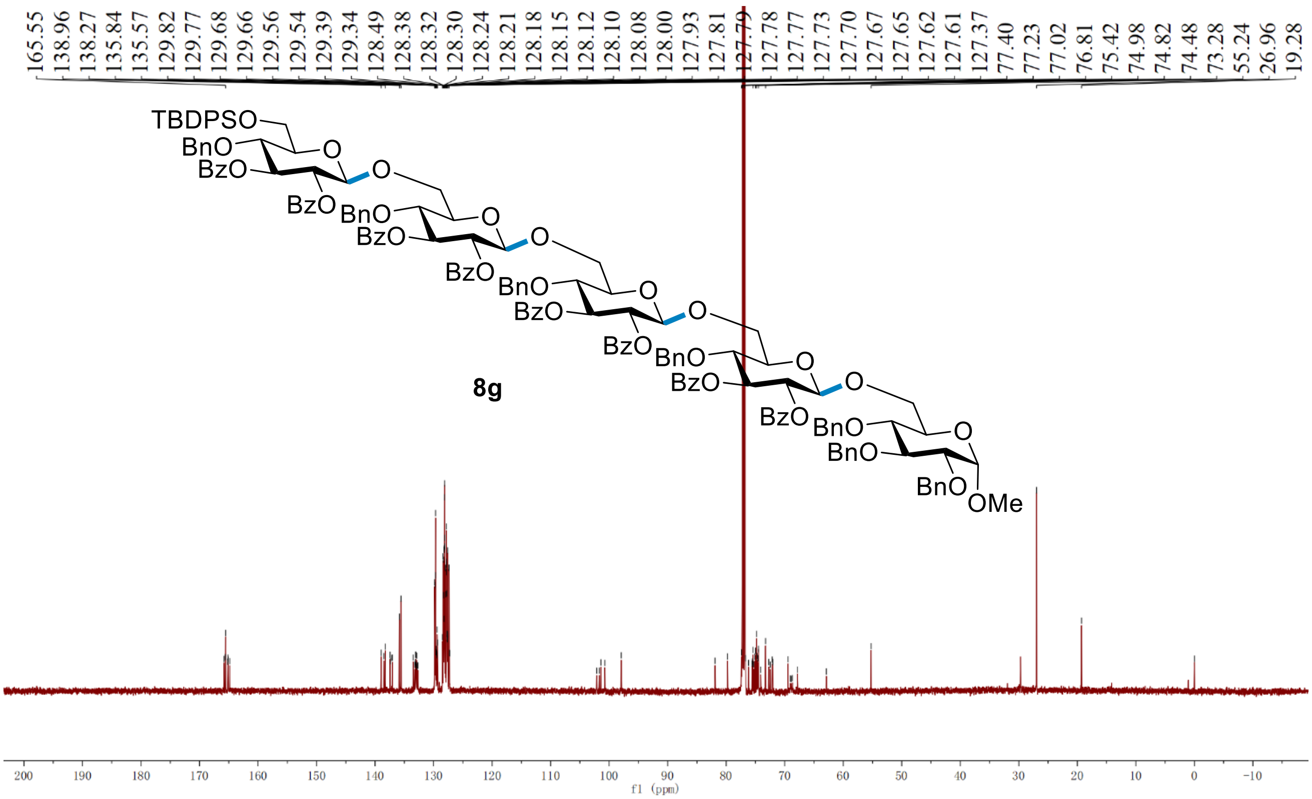

^{1}H NMR Spectrum of **8h** (400 MHz, $CDCl_3$)

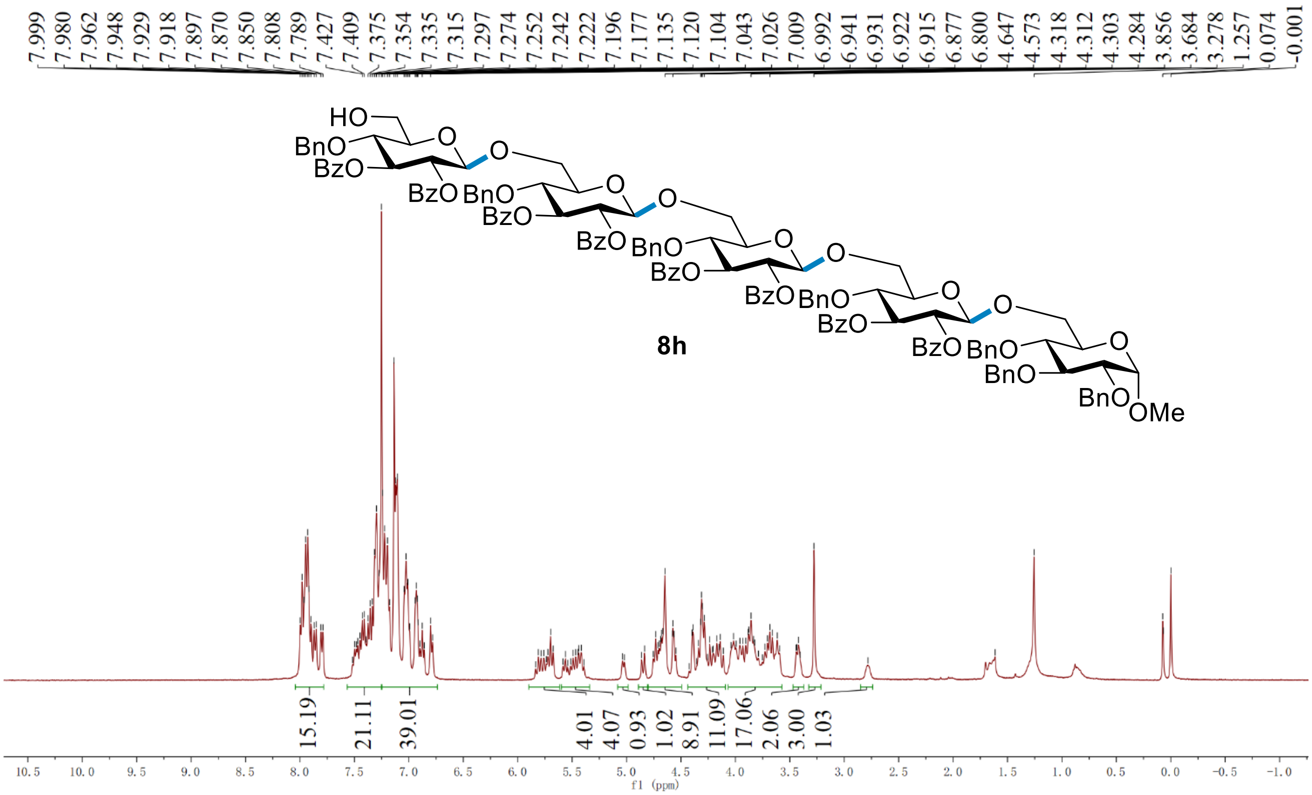

^{13}C NMR Spectrum of **8h** (101 MHz, $CDCl_3$)

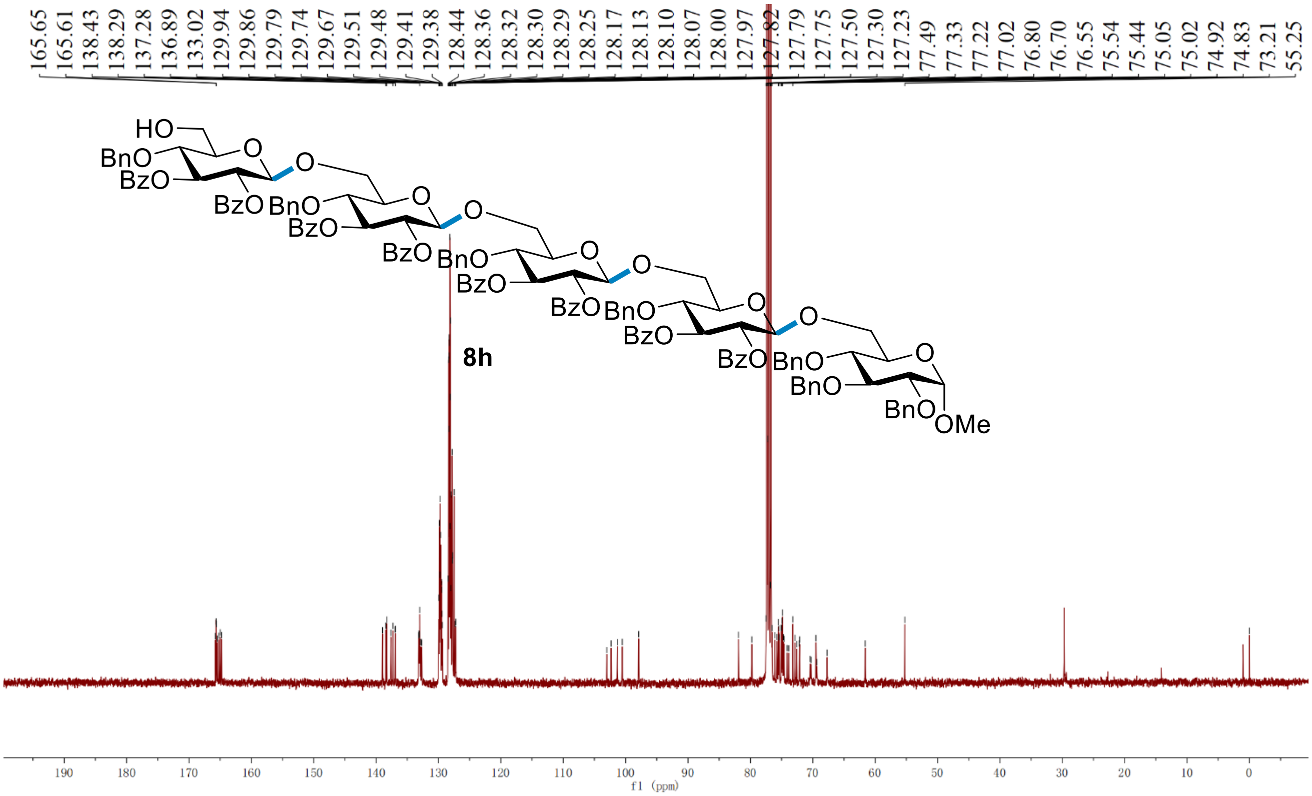

^{1}H NMR Spectrum of **8i** (400 MHz, $CDCl_3$)

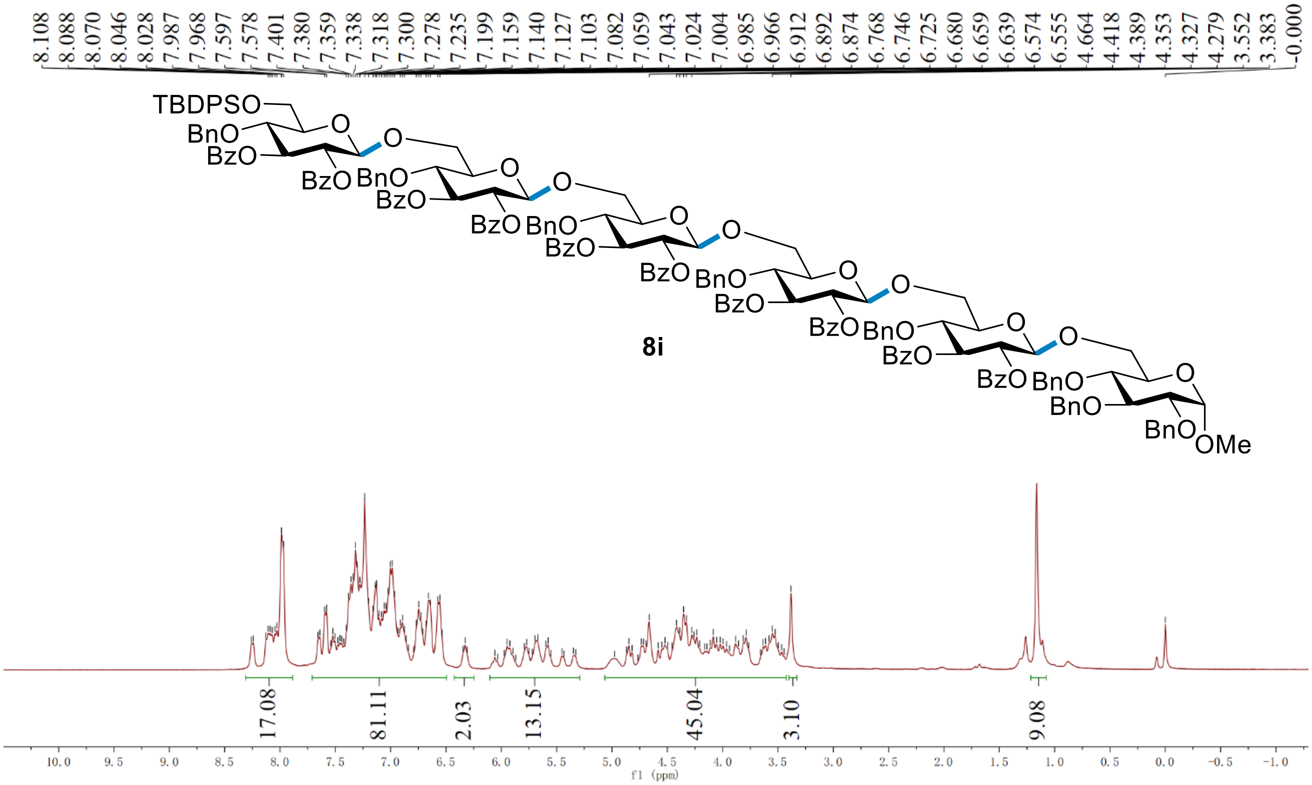

^{13}C NMR Spectrum of **8i** (101 MHz, $CDCl_3$)

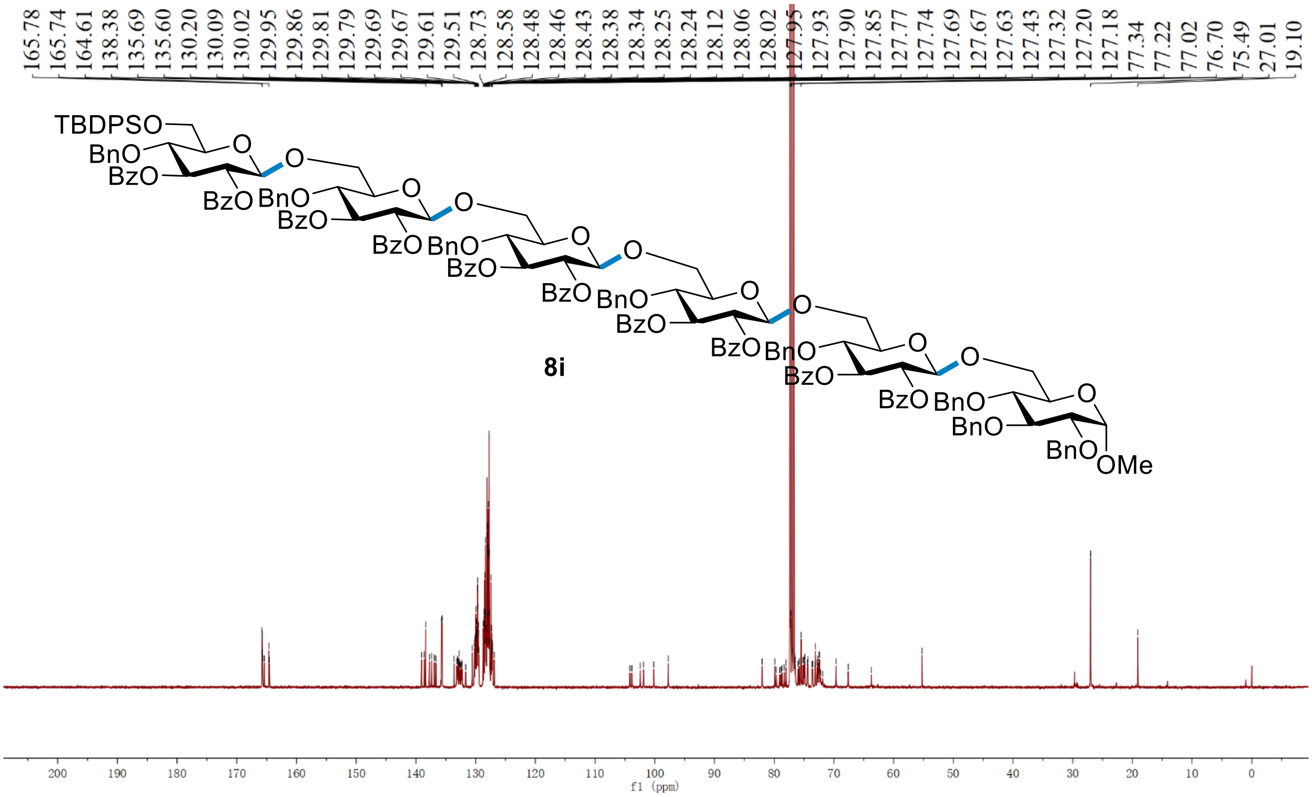

^{1}H NMR Spectrum of **S27** (400 MHz, $CDCl_3$)

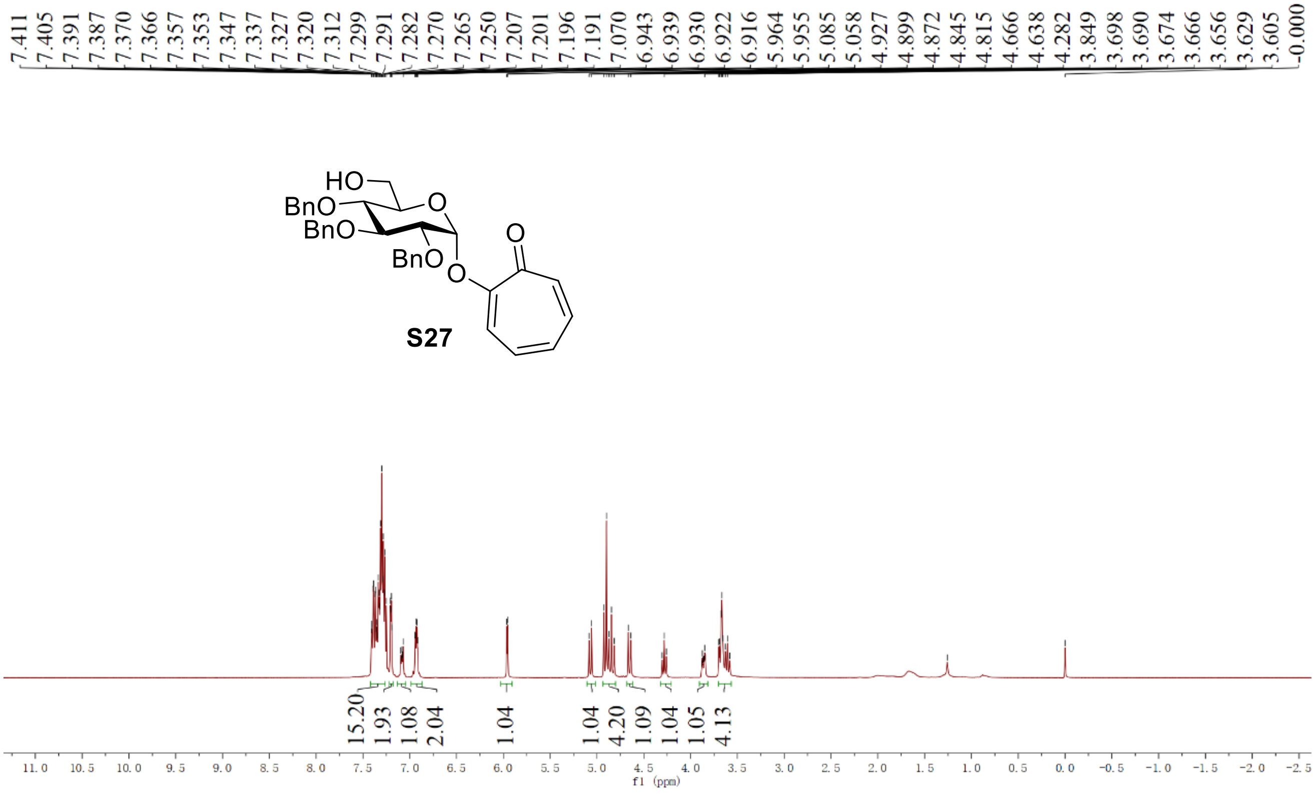

^{13}C NMR Spectrum of **S27** (151 MHz, $CDCl_3$)

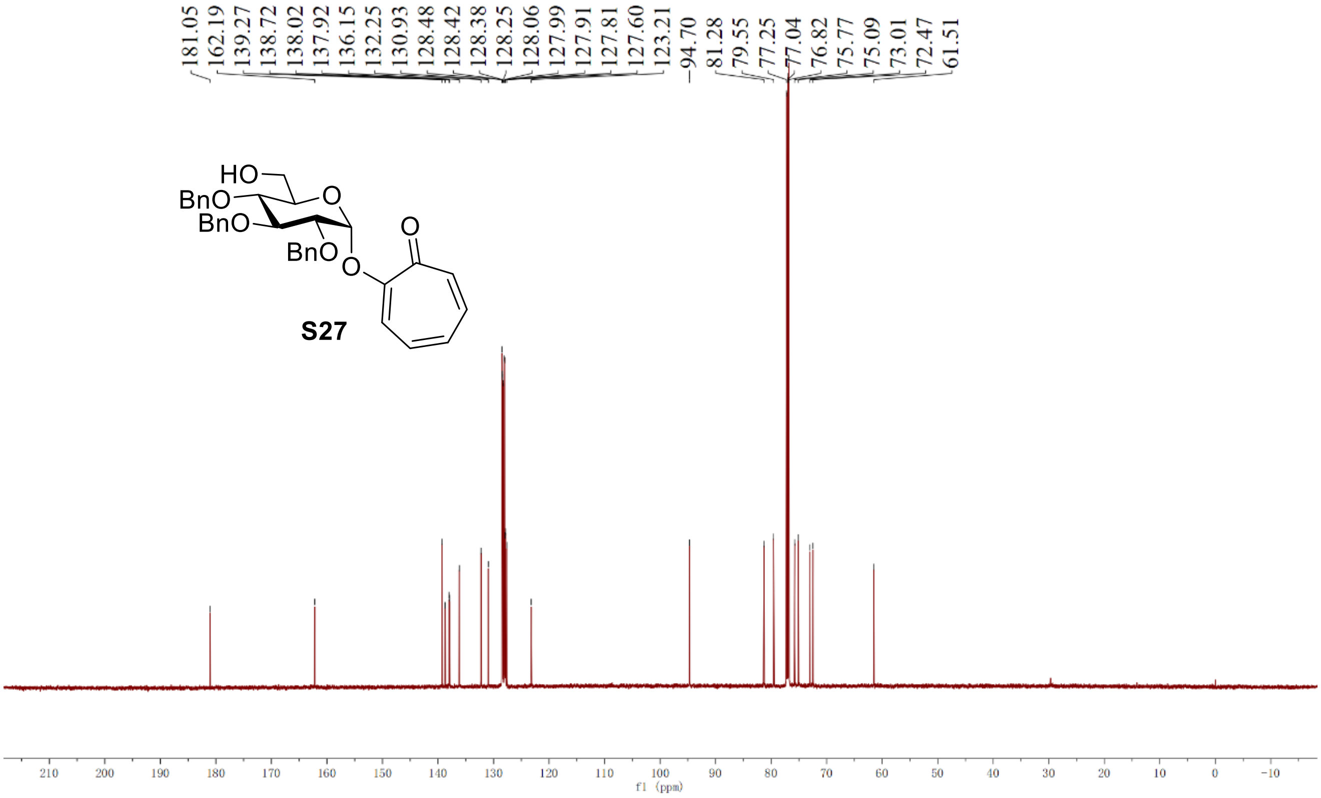

^{1}H NMR Spectrum of **S29** (400 MHz, $CDCl_3$)

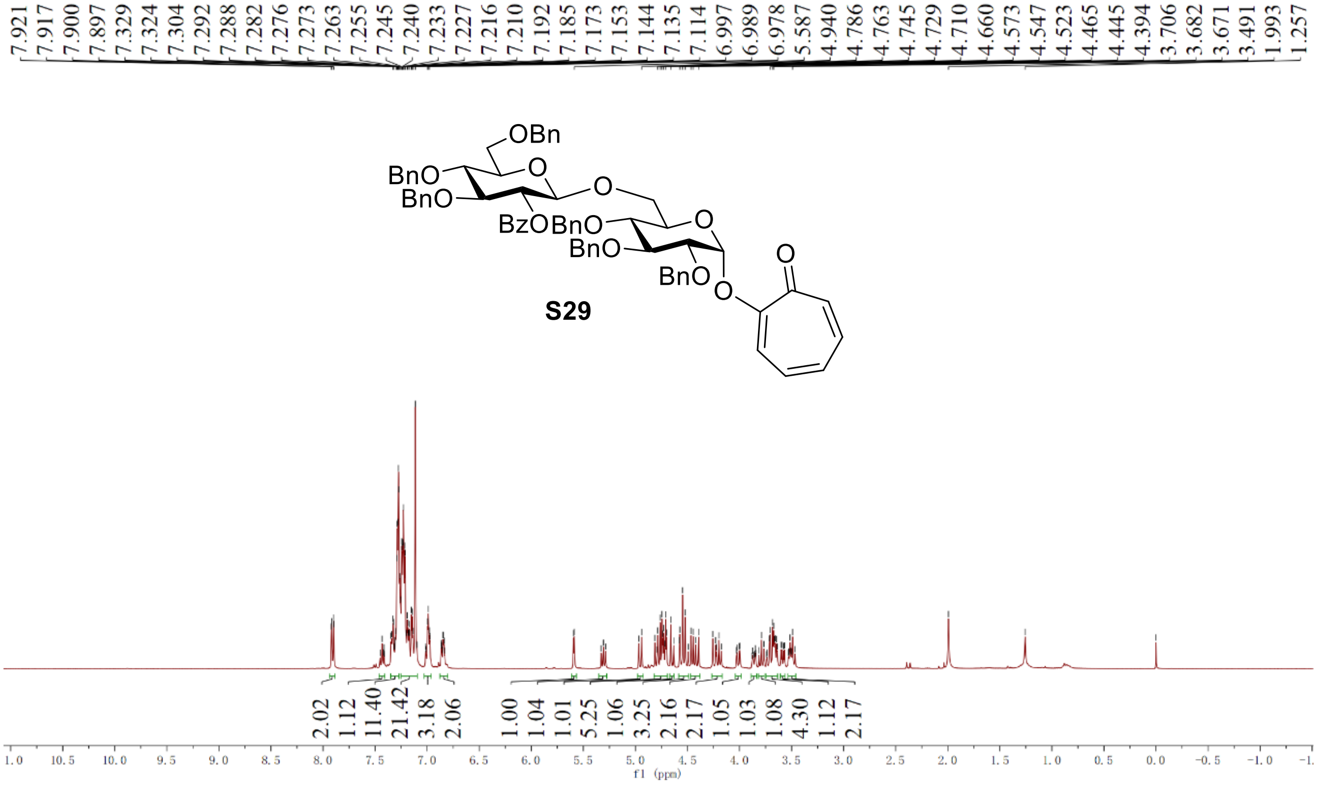

^{13}C NMR Spectrum of **S29** (100 MHz, $CDCl_3$)

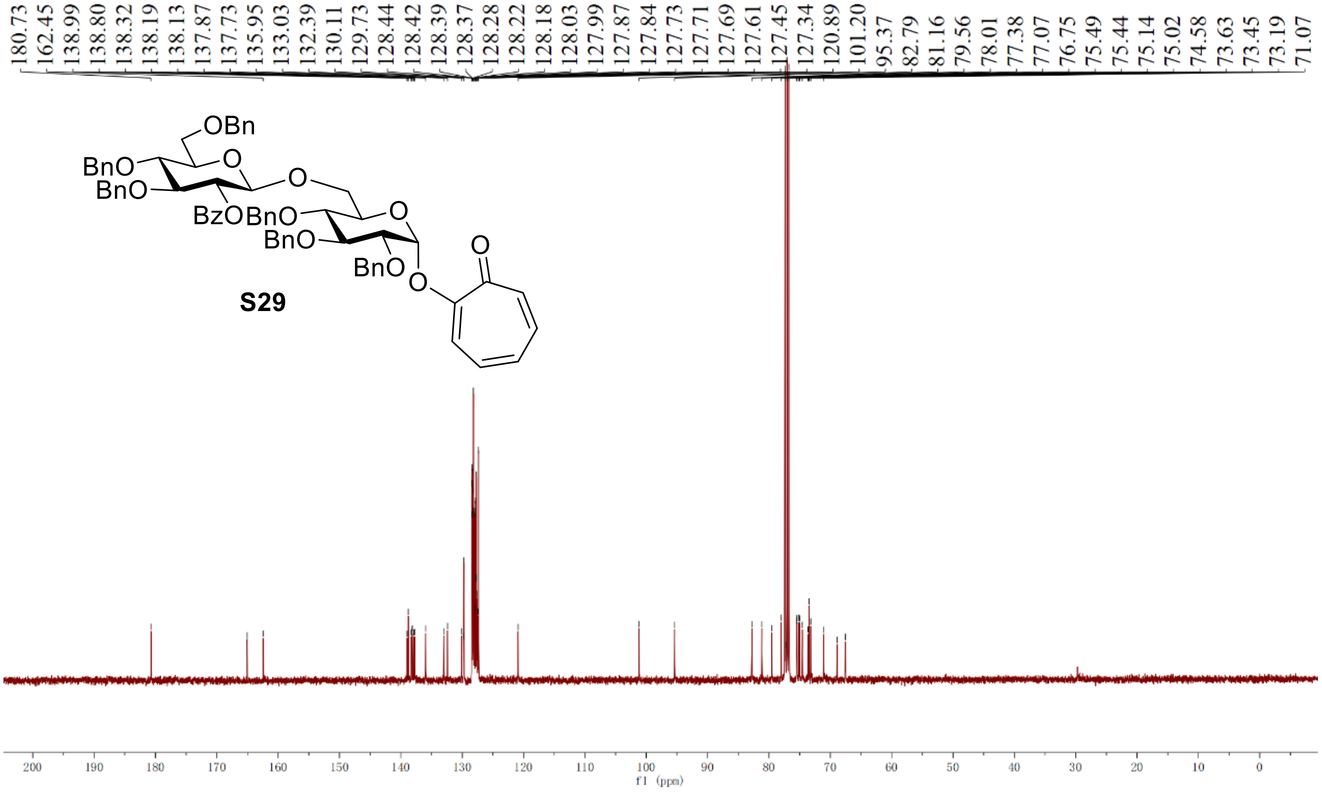

^{1}H NMR Spectrum of **S31** (400 MHz, $CDCl_3$)

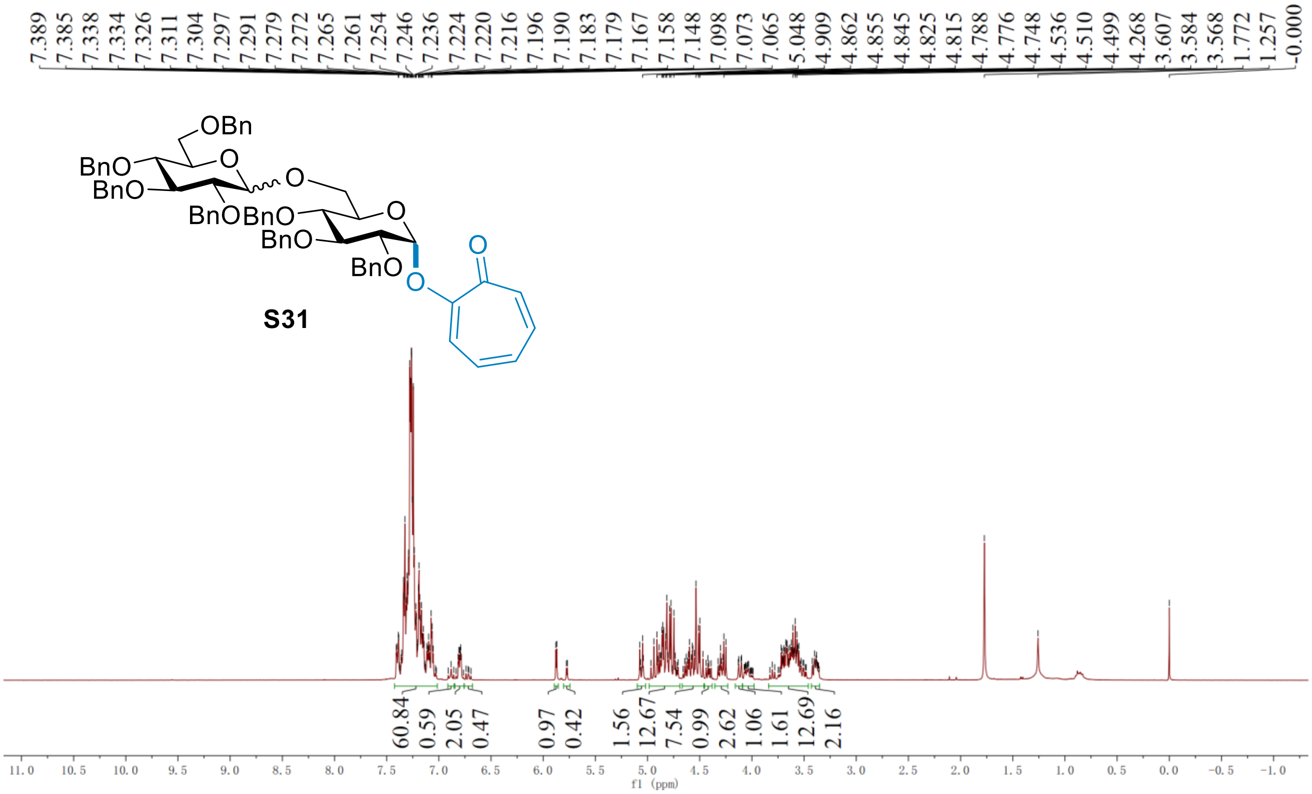

^{13}C NMR Spectrum of **S31** (100 MHz, $CDCl_3$)

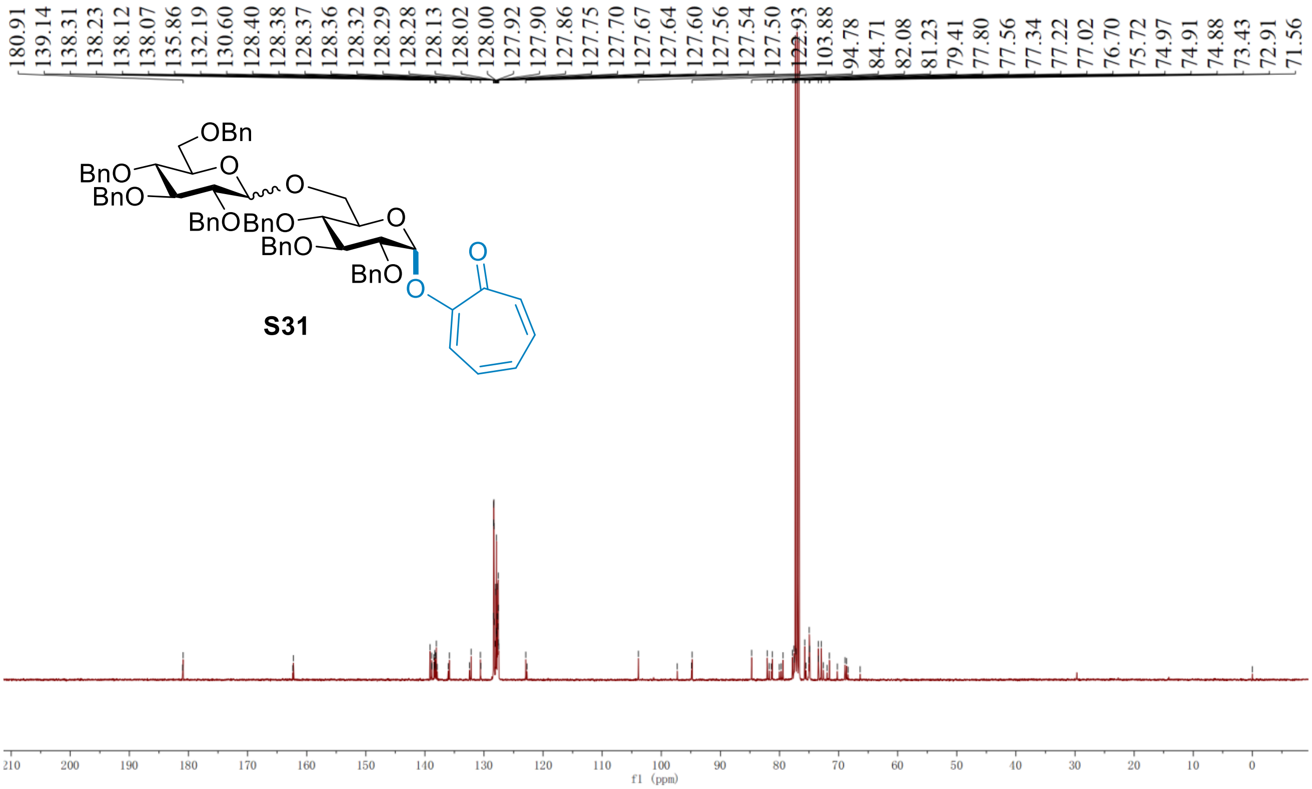

9. References

[1] Lyons, D. J. M., Empel, C., Pace, D. P., Dinh, A. H., Mai, B. K., Koenigs, R. M. & Nguyen, T. V. Tropolonate salts as acyl-transfer catalysts under thermal and photochemical conditions: reaction scope and mechanistic insights. *ACS Catal.* **10**, 12596-12606 (2020).

[2] Lu, S.-R., Lai, Y.-H., Chen, J.-H., Liu, C.-Y. & Mong, K.-K. T. Dimethylformamide: an unusual glycosylation modulator. *Angew. Chem. Int. Ed.* **50**, 7315 -7320 (2011).

[3] Liang, X.-Y., Deng, L.-M., Liu, X. & Yang, J.-S. Efficient one-pot syntheses of α-D-arabinofuranosyl tri- and tetrasaccharides present in cell wall polysaccharide of Mycobacterium tuberculosis. *Tetrahedron* **66**, 87-93 (2010).

[4] Chang, C.-W., Lin, M-H., Chan, C.-K., Su, K.-Y., Wu, C.-H., Lo, W.-C., Lam, S., Cheng, Y.-T., Liao, P.-H., Wong, C.-H. & Wang, C.-C. Automated quantification of hydroxyl reactivities: prediction of glycosylation reactions. *Angew. Chem. Int. Ed.* **60**, 12413 -12423 (2021).

[5] Hu, J.-C., Feng, A.-F. W., Chang, B.-Y., Lin, C.-H. & Mong, K.-K. T. A flexible 1,2-cis α-glycosylation strategy based on in situ adduct transformation. *Org. Biomol. Chem.* **15**, 5345-5356 (2017).

[6] Koto, S., Takebe, Y. & Zen, S. The Synthesis of methyl 2,4,6-tri-*O*-benzyl-α-D-glucopyranoside. *B. Chem. Soc. Jap.* **45**, 291-293 (1972).

[7] Lecourt, T., Herault, A., Pearce, A. J., Sollogoub, M. & Sinaÿ, P. Triisobutylaluminium and diisobutylaluminium hydride as molecular scalpels: the regioselective stripping of perbenzylated sugars and cyclodextrins. *Chem. Eur. J.* **10**, 2960-2971 (2004).

[8] Balmond, E. I., Coe, D. M., Galan, M. C. & McGarrigle, E. M. a-Selective organocatalytic synthesis of 2-deoxygalactosides. *Angew. Chem. Int. Ed.* **51**, 9152 -9155 (2012).

[9] Zhang, X., Yang, Y., Ding, J., Zhao, Y., Zhang, H., & Zhu, Y. Stereoselective gold(I)-catalyzed approach to the synthesis of complex α-glycosyl phosphosaccharides. *Nat. Commun.* **13**, 421 (2022).

[10] Smajlagic, I., Durán, R., Pilkington, M. & Dudding, T. Cyclopropenium enhanced thiourea catalysis. *J. Org. Chem.* **83**, 13973-13980 (2018).

[11] Wang, H. Y., Simmons, C. J., Blaszczyk, S. A., Balzer, P. G., Luo, R., Duan, X. & Tang, W. Isoquinoline-1-carboxylate as a traceless leaving group for chelation-assisted glycosylation under mild and neutral reaction conditions. *Angew. Chem. Int. Ed.* **56**, 15698-15702 (2017).

[12] Shaikh, A. Y., Sureshkumar, G., Pati, D., Gupta, S. S. & Hotha, S. Facile synthesis of unusual glycosyl carbamates and amino acid glycosides from propargyl 1,2-orthoesters as glycosyl donors. *Org. Biomol.Chem.* **9**, 5951-5959 (2011).

[13] Liu, M., Li, B. H., Xiong, D. C. & Ye, X. S. *O*-Glycosylation Enabled by *N*-(Glycosyloxy)acetamides. *J. Org. Chem.* **83**, 8292-8303 (2018).

[14] Mao, R.-Z., Xiong, D.-C., Guo, F., Li, Q., Duan, J. & Ye, X.-S. Light-driven highly efficient glycosylation reactions. *Org. Chem. Front.* **3**, 737-743 (2016).

[15] Singh, Y. & Demchenko, A. V. Defining the scope of the acid-catalyzed glycosidation of glycosyl bromides. *Chem. Eur. J.* **26**, 1042-1051 (2020).

[16] Sui, J.-J., Xiong, D.-C. & Ye, X.-S. Copper-mediated O-arylation of lactols with aryl boronic acids. *Chin. Chem. Lett.* **30**, 1533-1537 (2019).

[17] Liu, D.-K., Xiong, D.-C., Wu, X., Li, Q. & Ye, X.-S. Rapid glycosylation of 2′-benzoylphenyl glycosides promoted by TfOH. *Org. Chem. Front.* **6**, 2756-2759 (2019).

[18] Kamkhachorn, T., Parameswar, A. R. & Demchenko, A. V. Comparison of the armed/disarmed building blocks of the D-gluco and D-glucosamino series in the context of chemoselective oligosaccharide synthesis. *Org. Lett.* **12**, 3078-3081 (2010).

[19] Mukhopadhyay, B., Maurer, S. V., Rudolph, N., Well, R. M., Russell, D. A. & Field, R. A. From solution phase to "on-column" chemistry: trichloroacetimidate-based glycosylation promoted by perchloric acid-silica. *J. Org. Chem.* **70**, 9059-9062 (2005).

[20] Garcia, B. A. & Gin, D. Y. Synthesis of glycosyl-1-phosphates via dehydrative glycosylation. *Org. Lett.* **2**, 2135-2138 (2000).

[21] Koshiba, M., Suzuki, N., Arihara, R., Tsuda, T., Nambu, H., Nakamura, S. & Hashimoto, S. Catalytic stereoselective glycosidation with glycosyl diphenyl phosphates: rapid construction of 1,2-cis-α-glycosidic linkages. *Chem. Asian J.* **3**, 1664-1677 (2008).

[22] Tsuda, T., Nakamura, S. & Hashimoto, S. A highly stereoselective construction of 1,2-trans-β-glycosidic linkages capitalizing on 2-azido-2-deoxy-d-glycosyl diphenyl phosphates as glycosyl donors. *Tetrahedron* **60**, 10711-10737 (2004).

[23] Liu, G.-J., Zhang, X.-T. & Xing, G.-W. A general method for *N*-glycosylation of nucleobases promoted by (*p*-Tol)$_2$SO/Tf$_2$O with thioglycoside as donor. *Chem. Commun.* **51**, 12803-12806 (2015).

[24] Sniady, A., Bedore, M. W. & Jamison, T. F. One-flow, multistep synthesis of nucleosides by brønsted acid-catalyzed glycosylation. *Angew. Chem. Int. Ed.* **50**, 2155-2158 (2011).

[25] Liang, H., Ma, L., Li, C., Peng, Q., Wang, Z., Zhang, Z.-X., Yu, L., Liu, H., An, F. & Xue, W. Efficient glycosylation with glycosyl *ortho*-allylbenzoates as donors. *Tetrahedron Lett.* **60**, 84-87 (2019).

[26] Huang, X., Huang, L., Wang, H. & Ye, X.-S. Iterative one-pot synthesis of oligosaccharides. *Angew. Chem. Int. Ed.* **43**, 5221-5224 (2004).

[27] Premathilake, H. D., Mydock, L. K. & Demchenko, A.V. Superarming common glycosyl donors by simple 2-*O*-benzoyl-3,4,6-tri-*O*-benzyl protection. *J. Org. Chem.* **75**, 1095-1100 (2010).

[28] Shaw, M. & Kumar, A. Visible-light-mediated *β*-C(sp^3)-H amination of glycosylimidates: En Route to oxazoline-fused/spiro nonclassical bicyclic sugars. *Org. Lett.* **21**, 3108-3113 (2019).
